# Host suppression of quorum sensing during catheter-associated urinary tract infections

**DOI:** 10.1038/s41467-018-06882-y

**Published:** 2018-10-25

**Authors:** Stephanie J. Cole, Cherisse L. Hall, Maren Schniederberend, John M. Farrow III, Jonathan R. Goodson, Everett C. Pesci, Barbara I. Kazmierczak, Vincent T. Lee

**Affiliations:** 1 Department of Cell Biology and Molecular Genetics, University of Maryland, College Park, College Park, MD 20742 USA; 2 Department of Microbial Pathogenesis, Yale University School of Medicine, New Haven, CT 06520 USA; 3 Department of Microbiology and Immunology, The Brody School of Medicine at East Carolina University, Greenville, 27834 NC USA

## Abstract

Chronic bacterial infections on medical devices, including catheter-associated urinary tract infections (CAUTI), are associated with bacterial biofilm communities that are refractory to antibiotic therapy and resistant to host immunity. Previously, we have shown that *Pseudomonas aeruginosa* can cause CAUTI by forming a device-associated biofilm that is independent of known biofilm exopolysaccharides. Here, we show by RNA-seq that host urine alters the transcriptome of *P. aeruginosa* by suppressing quorum sensing regulated genes. *P. aeruginosa* produces acyl homoserine lactones (AHLs) in the presence of urea, but cannot perceive AHLs. Repression of quorum sensing by urine implies that quorum sensing should be dispensable during infection of the urinary tract. Indeed, mutants defective in quorum sensing are able to colonize similarly to wild-type in a murine model of CAUTI. Quorum sensing-regulated processes in clinical isolates are also inhibited by urea. These data show that urea in urine is a natural anti-quorum sensing mechanism in mammals.

## Abstract (web summary)

*Pseudomonas aeruginosa* can cause chronic infections on medical devices by forming biofilms, a process regulated by quorum sensing (QS). Here, the authors show that *P. aeruginosa* is unable to perceive QS signals in the presence of either host urine or urea, and clinical isolates are often defective in QS.

## Introduction

Chronic bacterial infections on medical devices are associated with bacterial biofilm communities that are refractory to antibiotic therapy and resistant to host immunity^1,2^. Bacteria use quorum sensing to coordinate their behavior at high cell density to produce biofilm matrix components^3–5^. Quorum-sensing pathways from diverse Gram-positive and Gram-negative bacteria are required for infections in mammalian hosts^6,7^.

*Pseudomonas aeruginosa* strain PAO1 defective in quorum sensing exhibited decreased biofilm formation in vitro^8,9^ and reduced virulence in murine models of acute urinary tract infection^10,11^, acute pulmonary infection^12^, and burn wound infection^13^. Strategies to inactivate or interfere with quorum sensing have therefore been targeted in order to control bacterial infections^14–16^. Currently, the ability of mammalian hosts to interfere with bacterial quorum sensing is the subject of intense investigation^17^. Mammalian hosts encode paraoxonases, which are lactonases that can inactivate acyl homoserine lactone (AHL) and decrease *P. aeruginosa* biofilm formation, pyocyanin production, and protease activity in vitro^18–20^. However, the role of quorum sensing in chronic infections is not well understood.

Here, we show that urea within host urine represses *Pseudomonas aeruginosa* quorum sensing in vitro and during catheter-associated urinary tract infections (CAUTI). Bacterial perception of homoserine lactone (HSL) quorum-sensing signals, 3-oxo-C_12_-HSL and C_4_-HSL, was disrupted, while ability to produce quorum molecules remained intact. These results imply that quorum sensing should be dispensable during infection of the urinary tract. This was tested by infecting quorum-sensing defective mutants of *P. aeruginosa* in a murine model of CAUTI. These mutants were able to colonize the catheter in a manner similar to wild type, indicating that quorum sensing is indeed dispensable during CAUTI. The contribution of quorum sensing was also assessed for human clinical CAUTI isolates. Quorum-sensing-regulated processes in a majority of human CAUTI isolates were inhibited by urea, while the remaining clinical isolates were found to be quorum defective, indicating that urine suppression of *P. aeruginosa* quorum sensing is a conserved process in mammalian systems. Overall, our data imply that urea in urine is a mammalian host factor that interferes with bacterial quorum sensing.

## Results

### RNA-seq reveals that urine and urea repress quorum sensing

**Fig. 1:**
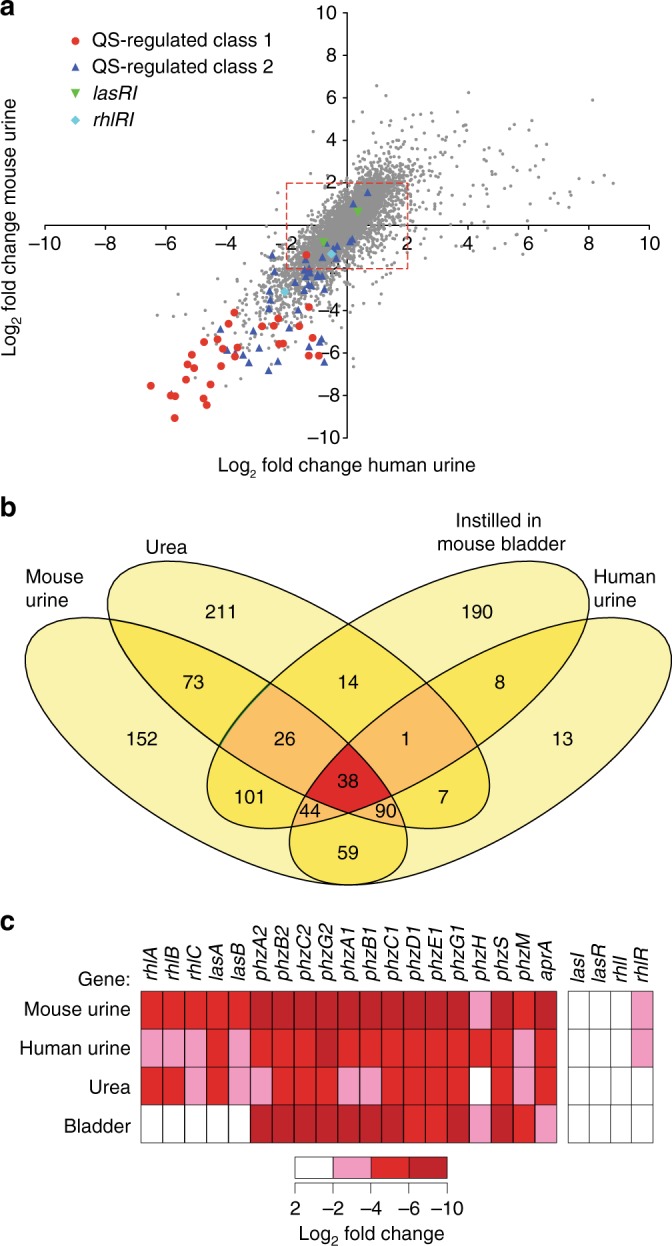
Urine and urea suppress the expression of quorum-regulated genes. **a** Comparisons of log_2_ fold changes in gene expression between LB vs mouse and human urine. Genes with less than four-fold change are within the box indicated by the red dashed line. The quorum-sensing-regulated genes were defined as those genes that had 20-fold decreased in Wagner et al.^25^ and 40-fold decreased in Schuster et al.^26^ in strains lacking *lasI rhlI* and *lasR rhlR*, respectively. Red circles indicate quorum-sensing-regulated genes that were identified in both studies (class 1), dark blue triangles indicate quorum-sensing-regulated genes that were identified in one of the two studies (class 2), green inverted triangles indicate *lasR* and *lasI* genes, and light blue diamonds indicate *rhlR* and *rhlI* genes. **b** Venn diagrams comparing the number of *P. aeruginosa* genes with four-fold reduction in expression when grown in mouse urine, human urine, media containing urea, and instilled in the mouse bladder as compared to bacteria grown in LB. **c** Heat map showing log_2_ fold gene expression changes of quorum-sensing-regulated genes in the presence of indicated urine, urea, and instillation into a mouse bladder

To investigate factors that are important during chronic infection, we established a murine model of CAUTI for the opportunistic pathogen *P. aeruginosa*. In this model, biofilm formation in the catheter lumen is dependent on extracellular DNA (eDNA)^21,22^ and independent of the exopolysaccharides required for abiotic biofilms^23,24^. Murine urine and urea were able to induce these eDNA-dependent biofilms, indicating that *P. aeruginosa* specifically responds to urine and urea. To characterize this response, the transcriptome of *P. aeruginosa* grown in media supplemented with mouse urine, human urine or urea, or instilled into the mouse bladder, was assayed by RNA-seq. Bacteria exposed to mouse and human urine showed similar changes in gene expression (Fig. 1a and complete RNA-seq data are available in Supplementary Data 1), and an overlapping transcriptional response to urine, urea, and incubation within mice bladder was observed. A Venn diagram of genes that were ≥4-fold down-regulated in each condition (Fig. 1b and Supplementary Table 1) shows that *P. aeruginosa* had a conserved response to mammalian urine and urea. Of note, *aprA, rhlAB, rhlC*, *lasA*, *lasB* genes, and *phz* operons, all genes known to be regulated by the *las* and *rhl* quorum-sensing systems, were among those most down-regulated in response to urea and urine (Fig. 1a, c)^25,26^. Microarray studies have reported positive-feedback regulation of the *lasI*/*lasR* and *rhlI*/*rhlR* quorum regulatory systems^25,26^. This was also observed in our RNA-seq data, with *lasI*/*lasR* and *rhlI*/*rhlR* genes being down-regulated, albeit by less than four-fold, in response to urine, urea, or growth within the murine bladder (Fig. 1c). Together, these results indicated that *P. aeruginosa* responds to growth in urine and media containing urea by altering its transcriptome in a manner similar to that of quorum-sensing mutants lacking AHL synthases or AHL receptors^25,26^.

### Urine and urea impair quorum-sensing-dependent phenotypes

**Fig. 2:**
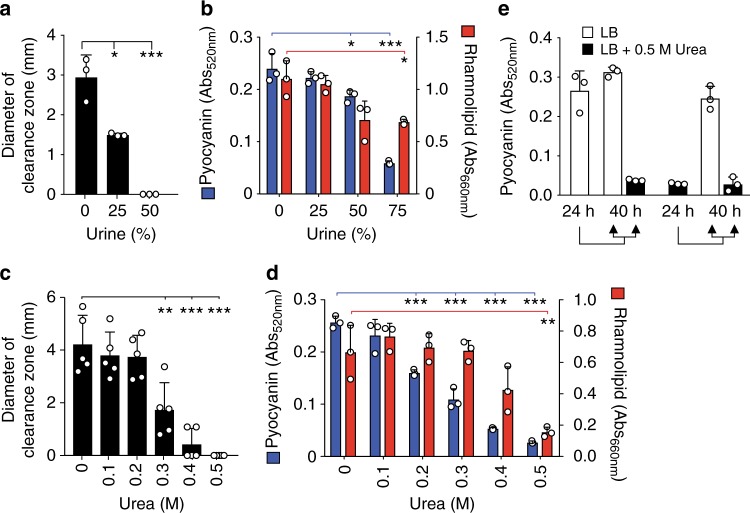
Urine and urea suppress quorum-regulated phenotypes. *P. aeruginosa* grown in 1× phosphate-buffered saline + 1% tryptone (PBS-T) supplemented with increasing concentrations of human urine was quantified for **a** zone of clearance around bacterial colony grown on milk plates, **b** pyocyanin (red bars) and rhamnolipid (blue bars) production. *P. aeruginosa* grown in LB supplemented with increasing amounts of urea was quantified for **c** zone of clearance around colonies grown on milk plates, **d** pyocyanin (red bars) and rhamnolipid (blue bars) production. **e** Quantification of pyocyanin produced by *P. aeruginosa* after 24-h growth in the presence or absence of 0.5 M urea followed by 16-h subculture growth in the presence or absence of 0.5 M urea. Data represent the mean and standard deviation of at least three independent replicates and were analyzed by unpaired *t*-test using GraphPad Prism software. Asterisk (*, **, and ***) indicates *p* < 0.05, <0.01, and <0.001, respectively

*P. aeruginosa* has several well-characterized quorum-regulated phenotypes, including elastase clearance on milk plates^27,28^, rhamnolipid production, and pyocyanin production^29–31^. We tested whether repression of quorum-regulated genes by urine and urea was significant enough to repress quorum-regulated phenotypes. Increasing amounts of mouse urine diminished elastase clearance zones on milk plates (Fig. 2a), and decreased both rhamnolipid and pyocyanin production (Fig. 2b). These phenotypes were not due to reduced growth, as *P. aeruginosa* grew to similar optical density in liquid cultures in the absence or presence of urine at the tested concentrations^21^. The concentration of urea in the pooled mouse and human urine was experimentally determined to be 786 ± 22 and 243 ± 14 mM, respectively. Addition of increasing concentrations of urea diminished elastase clearance zones on milk plates (Fig. 2c) and further inhibited rhamnolipid and pyocyanin production (Fig. 2d). The repression of quorum sensing by urine and urea was reversible as bacteria previously grown in media with 0.5 M urea were able to produce pyocyanin when subcultured in LB lacking urea (Fig. 2e). Together, these results indicate that urine and physiological concentrations of urea can repress LasR- and RhlR-mediated quorum-regulated phenotypes.

### Urea impairs perception of 3-oxo-C_12_-HSL by *P. aeruginosa*

**Fig. 3:**
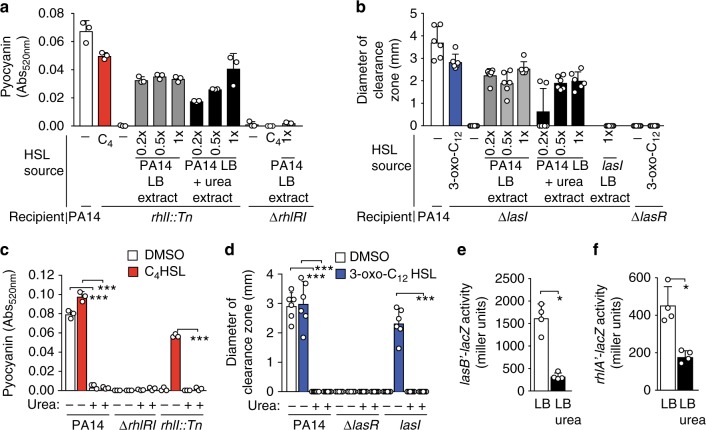
Urea affects perception, but not production, of autoinducers. **a** Production of C_4_-HSL by *P. aeruginosa* grown in the presence and absence of urea was assessed by the ability of spent media extracts to promote production of pyocyanin by PA14, *rhlI::Tn*, and *∆rhlRI*. **b** Production of 3-oxo-C_12_-HSL by *P. aeruginosa* grown in the presence and absence of urea was quantified by the ability of spent media extracts to promote protease activity by PA14, *∆lasI*, and *∆lasR* grown on milk plates. Extracts of culture supernatants from cells grown in the absence of urea (gray) and presence of urea (black) are indicated. **c** Perception of chemically synthesized C_4_-HSL (0.5 µM) as measured by pyocyanin production by PA14, *rhlI::Tn*, *∆rhlRI* grown in LB in the absence and presence of 0.5 M urea. **d** Perception of chemically synthesized 3-oxo-C_12_-HSL (1 µM) as quantified by PA14, ∆*lasI,* and *∆lasR* grown on milk plates in the absence or presence of 0.5 M urea. Samples, in which control HSL was added, are indicated in red for C_4_-HSL and blue for 3-oxo-C_12_-HSL. **e**, **f** Beta-gal activity was measured for *E. coli* strain DH5α carrying either **e** pECP64 (ptac-*lasR, lasB′-lacZ*) or **f** pECP61 (ptac-*rhlR rhlA’-lacZ*) grown with 5 nM 3-oxo-C_12_-HSL or 5 µM C_4_-HSL, respectively, in the presence (solid) or absence (open) of 0.5 M urea. Data represent the mean and standard deviation of at least three independent samples for each incubation time. Data were analyzed by unpaired *t*-test using GraphPad Prism software. Asterisk (*, **, and ***) indicates *p* < 0.05, <0.01, and <0.001, respectively

Quorum sensing can be repressed either by inhibiting the production of AHLs or by preventing the perception of AHLs by their receptors. To determine if urea inhibited the production of AHLs, we used ethyl acetate to extract AHLs from spent media of *P. aeruginosa* strain PA14 grown in the presence or absence of urea. The extracts were assayed for the ability to restore quorum-sensing-dependent phenotypes in ∆*lasI* or *rhlI:Tn* strains, which lack the respective synthases for 3-oxo-C_12_-HSL and C_4_-HSL quorum molecules. Extracts from PA14 grown either in the presence or in the absence of urea restored pyocyanin production in the *rhlI::tn* mutant similar to chemically synthesized C_4_-HSL (Fig. 3a). Likewise, extracts from PA14 grown in the presence or absence of urea induced protease production from a ∆*lasI* mutant similar to chemically synthesized 3-oxo-C_12_-HSL (Fig. 3b). This extract-mediated induction was due to the presence of secreted AHLs, as extracts from the spent media of ∆*lasI* did not induce protease activity by ∆*lasI* (Fig. 3b). While AHL concentration was lower when bacteria were grown in media containing urea, our data show that the levels of AHLs were above the threshold required to induce quorum sensing, suggesting that urea neither prevents secretion of quorum molecules nor chemically inactivates the quorum molecule.

To determine if urea disrupts the perception of AHLs, chemically synthesized AHLs were added to wild type and synthase mutant bacteria in media with or without 0.5 M urea. In the absence of urea, the addition of C_4_-HSL restored pyocyanin production to a *rhlI::tn* mutant (Fig. 3c) and addition of 3-oxo-C_12_-HSL restored elastase production in a ∆*lasI* mutant (Fig. 3d) to similar levels as observed for wild-type parental strains in the absence of urea. In contrast, the wild-type parental strain had significantly reduced pyocyanin and protease production in the presence of urea, and addition of excess AHLs failed to restore quorum-regulated responses. Likewise, the addition of C_4_-HSL or 3-oxo-C_12_-HSL to the respective synthase mutant strain failed to induce pyocyanin or elastase production in the presence of urea. Plasmid-derived over-expression of *rhlR* or *lasR* in the respective null mutants, while able to complement the respective mutations, did not overcome repression of quorum-regulated phenotypes by urea (Supplementary Figure 1). Since quorum regulation in *P. aeruginosa* is complex and involves many regulators, we asked whether urea repression of quorum sensing was also observed for *lasR*- and *rhlR*-regulated systems recapitulated in *Escherichia coli*^32^. *E. coli* strains expressing LasR can activate a *lasB′-lacZ* reporter when stimulated with exogenous 3-oxo-C_12_-HSL, but this activation was inhibited by the addition of 0.5 M urea (Fig. 3e). Urea also inhibited the activation of a *rhlA* reporter by exogenously added C_4_-HSL in *E. coli* (Fig. 3f), indicating that the process inhibited by urea is common to *P. aeruginosa* and *E. coli*. Together these results indicate that urea in urine disrupts quorum sensing by inhibiting bacterial perception of AHLs without chemically altering the AHL molecule.

### Urea does not prevent LasR-3-oxo-C_12_-HSL from binding to DNA

Urea could inhibit AHL perception by either inhibiting the quorum receptor-AHL complex from binding to DNA or preventing the AHLs from activating the quorum receptor. To test whether urea inhibited the LasR-3-oxo-C_12_-HSL complex from binding DNA, purified recombinant LasR bound to 3-oxo-C_12_-HSL was assayed for DNA binding in the presence and absence of 0.5 M urea in vitro^33,34^. The LasR-3-oxo-C_12_-HSL complex reduced the mobility of DNA fragments containing known *las* box but not control DNA sequences lacking *las* box (Supplementary Figure 2). The addition of urea did not alter the ability of this LasR-3-oxo-C_12_-HSL complex to bind DNA.

### Quorum-sensing genes are dispensable during CAUTI

**Fig. 4:**
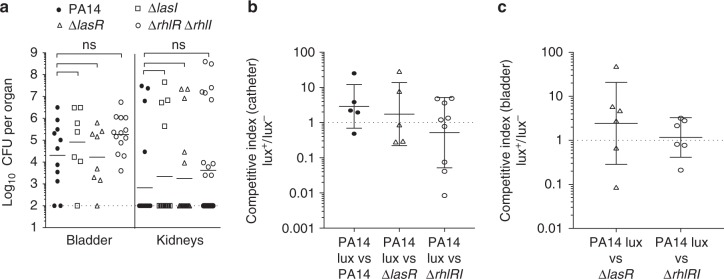
Quorum sensing is not required for catheter-associated urinary tract infection. **a** Bacterial load of the bladders and kidneys from mice infected with either PA14, ∆*lasR*,∆*lasI*, or ∆*rhlRI* using the CAUTI model of infection. The dashed line represents the limit of detection. Competitive indices (CI) for **b** biofilm formation in the catheter and **c** establishing infection in the bladder when mice were co-infected with PA14-lux and either unmarked PA14, ∆*lasR*, or ∆*rhlRI* in CAUTI. CI were calculated by first determining the ratio of lux+ and lux− CFU recovered from the catheter or infected bladder and then dividing by the ratio of lux+ and lux− CFU of the inoculum used for infection. Each symbol represents the CI measured for an individual bladder or a catheter. Dashed lines represent a CI value of 1 where no competition is observed. Horizontal line represents the geometric mean and error bars represent standard deviation

Based on the results showing urine inhibition of quorum sensing, we predicted that quorum sensing is not required to establish CAUTI and tested this hypothesis using a previously characterized murine model of CAUTI^21,35^. Bacterial persistence in this model results from catheter-associated colonization, as ≥10-fold higher numbers of bacteria were cultured from the catheter than from the bladder in 20 of 24 infected mice (Supplementary Figure 3). Mice infected with PA14 or the isogenic quorum-sensing mutants Δ*lasR*, Δ*lasI*, or Δ*rhlRI* had similar bacterial burdens in the bladder and kidneys, indicating that quorum sensing was dispensable for CAUTI (Fig. 4a). Likewise, competitive infections with PA14 and Δ*lasR* or Δ*rhlRI* showed that both Δ*lasR* and Δ*rhlRI* were as likely to form biofilms on catheter and to establish chronic infection within the bladder as wild-type PA14 (Fig. 4b, c). These results show that quorum sensing is not required for biofilm formation during CAUTI.

**Fig. 5:**
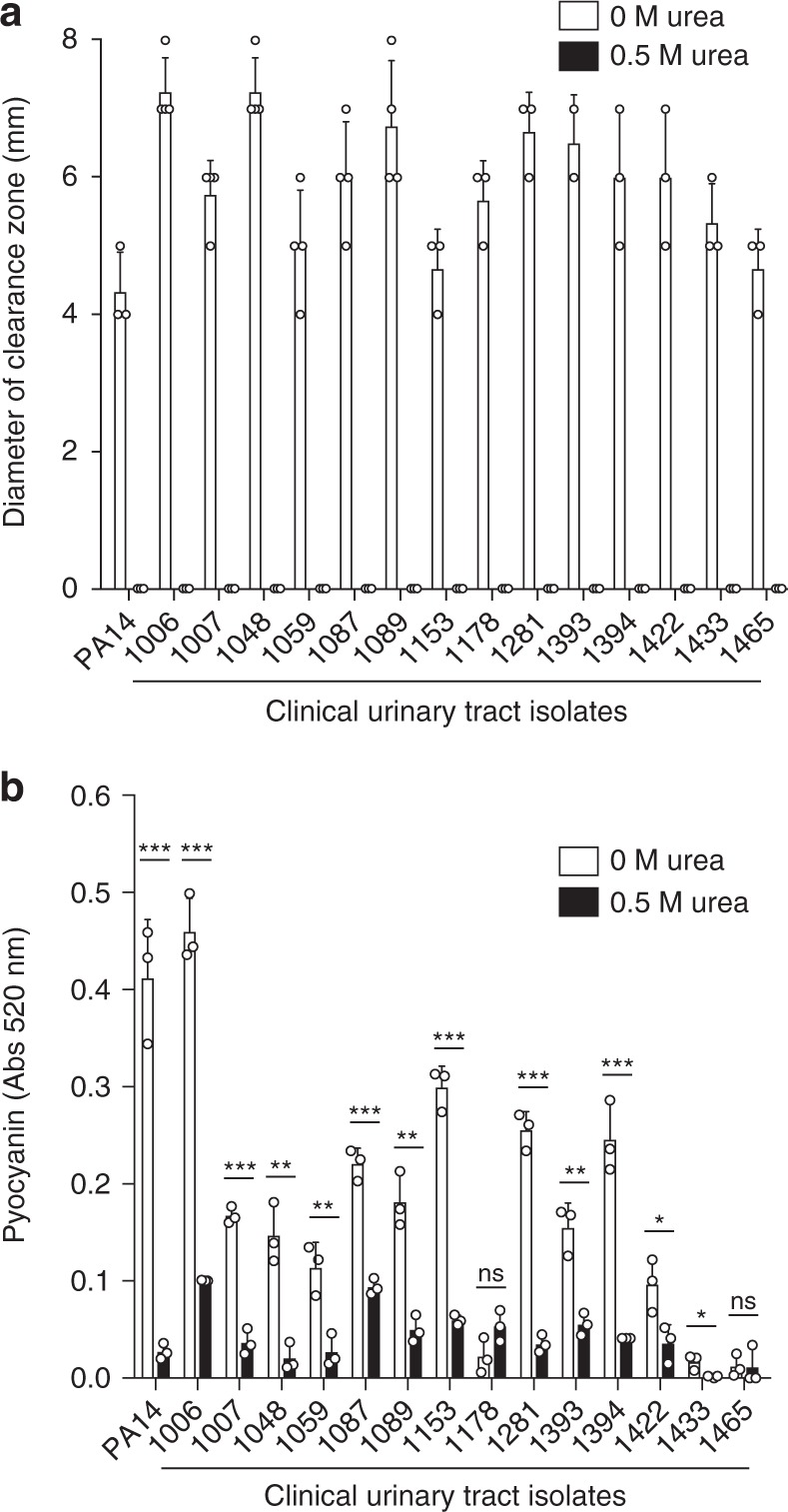
Urea suppresses quorum-sensing phenotypes in *P. aeruginosa* UTI clinical isolates. **a** Diameter of clearing around colonies for a selection of *P. aeruginosa* UTI clinical isolates grown on milk plates supplemented with either no urea (white bars) or 0.5 M urea (black bars). Under these conditions, none of the clinical isolates had elastase activity when grown in media containing 0.5 M urea. All comparisons between no urea and 0.5 M urea have a *p* < 0.001. **b** UTI strains grown in the presence and absence of 0.5 M urea were assessed for pyocyanin production. Data represent the mean and standard deviation of at least three independent samples. Data were analyzed by unpaired *t*-test using GraphPad Prism software by comparing samples with no urea or 0.5 M urea. Asterisk (*, **, and ***) indicates *p* < 0.05, 0.01, and <0.001, respectively and "ns" indicates not significant

If urine- and urea-mediated suppression of quorum sensing in *P. aeruginosa* also occurs during human CAUTI, we hypothesized that quorum sensing would be dispensable during human infections. To test this prediction, 29 clinical *P. aeruginosa* CAUTI isolates from human patients with urinary catheters or nephrostomy tubes were assessed for phenotypes that are indicative of quorum sensing^36^. Fifteen of these 29 isolates did not swarm, exhibited no or minimal protease activity, and were scored as “quorum negative”; the other 14 were considered “quorum competent” based on their protease activity and ability to swarm^36^. For quorum-negative strains, sequencing of the *lasRI* and *rhlRI* genes revealed one isolate with an early stop codon in *lasR* at position 186, two isolates with an early stop codon in *rhlR* at positions 67 and 74, respectively, and an isolate where *rhlR* had a substitution of the conserved P56 to alanine (Supplementary Table 2). About 0.5 M urea repressed the protease activity of the 14 quorum competent clinical isolates (Fig. 5a) and significantly reduced pyocyanin production in the 11 isolates that produced this phenazine (Fig. 5b). The suppression of quorum-sensing-dependent phenotypes by urea in human CAUTI isolates, and the loss of *lasR-* and *rhlR-*dependent phenotypes in other human CAUTI isolates suggests that quorum sensing is indeed dispensable for CAUTI. In this setting, quorum-sensing receptors become dispensable during chronic CAUTI, allowing *P. aeruginosa* to accumulate mutations in quorum-sensing genes. Missense and nonsense mutations in the quorum-sensing receptor *lasR* are commonly observed in chronic isolates from the lungs of cystic fibrosis (CF) patients^37^, suggesting that quorum sensing may be dispensable during other *P. aeruginosa* chronic infections.

## Discussion

This study establishes that urea within urine is a host factor that inhibits expression of quorum-regulated genes in *P. aeruginosa*. Transcriptome analysis showed that *P. aeruginosa* responds to urea and urine by down-regulating genes that are involved in quorum sensing, while phenotypic assays showed that *P. aeruginosa* phenocopied a quorum-sensing null mutant in the presence of urine and urea. Thus urea acts as a natural inhibitor of quorum sensing in *P. aeruginosa*. Future studies will determine the mechanistic basis for urine/urea inhibition of quorum sensing.

It is surprising that inhibition of AHL quorum sensing by urea or urine has not been previously reported, as urinary tract pathogens such as uropathogenic *E. coli* (UPEC), *Enterobacter* spp., *Proteus mirabilis,* and *Klebsiella pneumoniae* are well-studied^38^. Previous studies employing RNA-seq revealed the comprehensive transcriptome of UPEC isolates from human UTI patients^39^. *E. coli* is known to respond to AHL through the transcription factor SdiA^40^, a LuxR homolog^41^. Transcriptome analysis of these human UTI strains showed small (<4-fold) changes in expression levels of the SdiA regulon when UPEC isolates were grown in urine from healthy donors vs in LB media^39^. One reason for the absence of a urine-mediated effect on quorum-regulated genes in the UPEC study may be that enteric bacteria, such as UPEC, do not encode an endogenous AHL synthase gene^42^. The transcriptome study of UPEC isolates was performed in the absence of bacteria capable of producing AHLs that activate SdiA^39^. An effect of urea in urine on the SdiA regulon would not be expected in the absence of an exogenous source of AHL. Nonetheless, using the LasR system reconstituted in *E. coli*, we observed that growth in media containing urea reduced the expression of a *lasB*-lacZ reporter gene^31^. Future studies will determine if urine/urea inhibition of bacterial quorum sensing is a general process that affects all AHL utilizing systems and other bacterial quorum molecules such as autoinducer-2 and autoinducing peptides.^6,43^

During infection of mammalian host, quorum sensing is critical for acute infections of the lung^12^, urinary tract^10,11^ and burn wounds^44^ by *P. aeruginosa*. The role of quorum sensing during chronic infection by *P. aeruginosa* is still under active investigation. One of the key lines of evidence implicating quorum sensing in chronic *P. aeruginosa* infections comes from studies of CF isolates^37,45,46^. Sequencing of longitudinal strains from the same CF patient revealed that *P. aeruginosa* could cause a life-long infection and, during this prolonged infection, accumulate mutations in *mutS*, *mucA*, and *lasR* among many other genes^37,47,48^. Null mutations in *mutS* are found in 35–50% of CF isolates, explaining the often observed hypermutability of CF isolates^47,49^. Mutations in *mucA* leads to derepression of the AlgU/AlgT sigma factor resulting in the constitutive expression of the alginate biosynthesis (*algD-A*) operon^50,51^. As a consequence, these strains have increased production of the alginate exopolysaccharide^52^, which leads to reduced lung function of CF patients. While the effects of mutations in *mutS* and *mucA* on *P. aeruginosa* CF isolates have been well characterized, the effect of mutations in *lasR* is still debated. One hypothesis of why *lasR* function is lost during chronic lung infection is that *lasR* mutation provides an adaptive growth advantage in the low nutrient environment of the CF lung^45,53^. An alternative hypothesis posits that *lasR* mutants are social cheaters that benefit from quorum-sensing-regulated proteins made by neighboring bacteria without paying the metabolic cost of contributing to their production^54^. We would like to propose a third hypothesis in which the high frequency of *lasR* mutations present in clinical *P. aeruginosa* isolates reflects the absence of a requirement for quorum sensing during chronic infection. Similar to chronic CF isolates, *P. aeruginosa* isolates from human patients with CAUTI have acquired mutations in *lasR* and display null *lasR* quorum-sensing phenotypes. In murine chronic CAUTI infections, we have shown that quorum sensing is in fact dispensable as quorum-sensing null mutants successfully competed with wild-type *P. aeruginosa* in establishing chronic infection. The differences in the requirement of virulence factors between acute infections and chronic infections should change our view of chronic infections and lead to the development of treatment strategies that are specific for chronic infections.

## Methods

### Bacterial cultures and growth conditions

*rhlR*, *rhlI*, and *lasI* transposon mutants were obtained from the *P. aeruginosa* PA14 non-redundant transposon insertion mutant library provided by the Ausubel Lab at Harvard Medical School, Boston, MA^55^. In-frame deletion mutants and HA-tagged genes were made using primers listed (Supplementary Table 3). For in-frame deletion mutants, 1 kb regions upstream and downstream of the gene were cloned into pCR-Blunt. Then the two 1 kb fragments were subcloned into a pEX vector^56^. The in-frame deletion was introduced into wild-type *P. aeruginosa* PA14 by conjugation of the pEX deletion plasmid using the *E. coli* HB101 pRK2013 helper strain. Unmarked in-frame deletion mutations were selected by sucrose counter-selection, screened by PCR and confirmed by sequencing. Complementation strains were made by cloning the genes into pCR-Blunt vector and then transferring the gene to a pMMB expression vector. The *E. coli* strains expressing the plasmids were then mated with the corresponding *P. aeruginosa* knockout strain. All strains were grown in either LB or 1× PBS + 1% tryptone (PBS-T).

### Collection of human and mouse urine

Urine was collected from human volunteers with informed consent in accordance with the Institutional Review Board at the University of Maryland, College Park. Identifying information about the volunteers were not collected at the time of sample donation. Urine was sterile filtered through a 0.2 µm filter, pooled, and stored at −80 °C until use. Mouse urine was collected from anesthetized mice by insertion of sterile catheter tubing. Urine was collected from the catheter, pooled, sterile filtered, and stored at −80 °C until use.

### RNA sequencing library preparation and analysis

RNA was isolated from bacteria grown in LB, LB + 0.5 M urea, PBS-T, PBS-T + 75% human urine, or PBS-T + 75% mouse urine using Qiagen RNeasy Mini Kit (cat no. 74104). For the in vivo RNA-seq, *P. aeruginosa* was instilled into bladders of anesthetized mice for 90 min prior to collection and RNA isolation. Ribosomal RNA was removed from total RNA using the Epicentre Ribo-Zero Magnetic Kit (cat no. MRZGN126). Ribosomal-free RNA was then fragmented and cDNA library was prepared using Epicentre ScriptSeq v2 RNA-Seq Library Preparation Kit (cat no. SSV21106). RNA-seq libraries were sequenced using Illumina HiSeq 1000 at the Institute for Bioscience & Biotechnology Research (IBBR) at University of Maryland, College Park. Sequences were aligned to the PA14 reference genome using Bowtie2 (ref. ^57^). Interactive Genome Viewer was used to visualize the data. Fold changes were determined using DESeq.

### Quantification of pyocyanin

Pyocyanin was quantified using a spectrophometric method^58^. Indicated *P. aeruginosa* strains were grown in LB for 16 h while shaking at 37 °C. Overnight cultures were then diluted 1:1000 in LB supplemented with either 0, 0.1, 0.2, 0.3, 0.4, or 0.5 M urea or PBS-T supplemented with 25%, 50%, or 75% human urine and subcultured for an additional 16 h at 37 °C. Following subculturing, the optical density (OD_600_) was measured. Cultures were centrifuged at 16,100 *g* for 5 min. The supernatant (1.25 ml) was then extracted with 500 µl chloroform. Pyocyanin-containing chloroform extracts were extracted again with 250 µl 0.2 N HCl, which protonates pyocyanin changing the color from blue to red. Pyocyanin was quantified by measuring absorbance (*A*_520_) in a spectrophotometer^58^. When testing for reversibility of quorum phenotypes, pyocyanin was quantified for the overnight cultures grown in LB with or without 0.5 M urea. Each of the overnight cultures were then used for 1:1000 subcultures in media with or without 0.5 M urea. After an additional 16 h, pyocyanin was assayed.

### Protease assay

Protease activity was assessed using milk agar medium^59^. Overnight cultures of indicated *P. aeruginosa* strains were diluted to approximately 5 × 10^4^ bacteria per ml. One microliter of the diluted culture was spotted on the surface of protease agar plates containing 2% (w/v) nutrient agar and 2.5% (w/v) non-fat dried milk and supplemented with indicated amounts of urea or human urine. Protease agar plates were incubated at 37 °C for 16 h or just before protease activity was detected from the negative control ∆*lasI*. Pictures of plates were taken using a Fuji LAS3000 Imager and zone of clearance was determined by subtracting the diameter of colonies from the diameter of clearing using ImageJ software.

### Rhamnolipid assay

Rhamnolipids were quantified using a colorimetric assay^60^. A volume of 4 ml of supernatant was isolated from 5 ml of overnight *P. aeruginosa* cultures. Rhamnolipids were extracted from supernatants using equal volumes of ethyl acetate. The ethyl acetate layer was transferred to a new tube and the extraction was repeated three times. The ethyl acetate extractions were combined and evaporated. The remaining precipitant was then resuspended in 4 ml chloroform and 400 µl methylene blue solution, mixed vigorously, and then allowed to incubate at room temperature for 15 min to allow for color development. One milliliter of the methylene blue/chloroform layer was transferred to a clean 2 ml microfuge tube and 500 µl of 0.2 N HCl was added. When mixed with acid, the methylene blue in the chloroform layer shifts from a blue to a red color in the aqueous layer. Rhamnolipids are quantified by transferring 200 µl of the aqueous layer to a flat bottom 96-well plate and measuring the absorbance at 660 nm using a spectrophotometer.

### Urea assay

Urea was quantified using a colormetric method by measuring the absorption of the dye dimethylaminobenzaldehyde (DMAB) (see Method 967.07 in Official Method of Analysis by ACOC^61^). A volume of 100 µl of 0.1 M DMAB was added to 100 µl of standard concentrations (0, 5, 10, 25, 50, and 100 mM) of urea resuspended in artificial urine media. Standards were thoroughly mixed and allowed to incubate at room temperature for at least 5 min and absorbance was measured at 420 nm. Quantification of urea present in human and mouse urine samples was analyzed simultaneously with standards. Dilutions were made of the urine in order for absorbance to be read within range of the standard curve and the spectrophotometer.

### Effect of urea on *rhl* and *las* systems expressed in *E. coli*

Bioassays to test the activity of AHLs in *E. coli* were performed using a modified procedure^31^. Overnight cultures of *E. coli* strain DH5α carrying either pECP64 or pECP61.5 were diluted into fresh supplemented A medium^62^ to an OD_600_ of 0.1 and incubated with shaking at 37 °C until OD_600_ of 0.5. While *E. coli* were growing, assay tubes were prepared with AHL by drying under N_2_. After addition of IPTG to a final concentration of 1 mM to the *E. coli* cultures, 950 μl aliquots of cultures were transferred to tubes that contained each AHL with indicated concentrations of urea. Cultures in tubes were incubated at 37 °C with shaking for 90 min after which β-galactosidase (β-gal) activity was measured using the Miller assay^63^.

### Extraction of autoinducers

Cultures of wild-type PA14 or ∆*lasI* were grown shaking overnight at 37 °C in 100 ml LB or LB + 0.5 M urea. Prior to centrifugation, aliquots of the overnight culture were removed to measure OD_600_ and colony-forming units (CFU). The supernatants of overnight cultures were collected and extracted twice with 90 ml acidified ethyl acetate using a separating funnel. Ethyl acetate extracts were then dried and resuspended in a calculated volume of DMSO that allows for normalization of bacterial CFU. Chemically synthesized C_4_-HSL (#10007898) and 3-oxo-C_12_-HSL (#10007895) were purchased from Cayman Chemical.

### Murine CAUTI

All studies and procedures were approved by the University of Maryland Institutional Animal Care and Use Committee (IACUC) and complied with all relevant ethical regulations. Murine infections were performed using CF-1 mice^21^. Mice were injected with 1.5 × 10^7^ CFU *P. aeruginosa* strain transurethrally after a 5-mm catheter was inserted in the bladder of anesthetized mice. Bladders and kidneys were harvested 1–2 weeks post infection. Only mice whose bladders still retained the 5 mm catheter at the end of the experiment were considered. For some experiments, catheters were removed from the bladders by extrusion through the urethra. Bacterial burdens were determined by first homogenizing catheter, bladder, or each kidney in 1 ml sterile 1× PBS-T followed by plating serial dilutions on LB agar plates to determine CFU.

## Electronic supplementary material

Supplementary Information

Description of Additional Supplementary Files

Supplementary Data 1

## Data Availability

RNA-seq data are deposited at SRA under the accession number SRP159622.

## References

[1] Costerton JW, Stewart PS, Greenberg EP (1999). Bacterial biofilms: a common cause of persistent infections. Science.

[2] Parsek MR, Singh PK (2003). Bacterial biofilms: an emerging link to disease pathogenesis. Annu. Rev. Microbiol..

[3] Hammer BK, Bassler BL (2003). Quorum sensing controls biofilm formation in *Vibrio cholerae*. Mol. Microbiol..

[4] Zhu J, Mekalanos JJ (2003). Quorum sensing-dependent biofilms enhance colonization in *Vibrio cholerae*. Dev. Cell.

[5] Parsek MR, Greenberg EP (1999). Quorum sensing signals in development of *Pseudomonas aeruginosa* biofilms. Methods Enzymol..

[6] Rutherford S. T., Bassler B. L. (2012). Bacterial Quorum Sensing: Its Role in Virulence and Possibilities for Its Control. Cold Spring Harbor Perspectives in Medicine.

[7] Schuster M, Sexton DJ, Diggle SP, Greenberg EP (2013). Acyl-homoserine lactone quorum sensing: from evolution to application. Annu. Rev. Microbiol..

[8] Davies DG (1998). The involvement of cell-to-cell signals in the development of a bacterial biofilm. Science.

[9] De Kievit TR, Gillis R, Marx S, Brown C, Iglewski BH (2001). Quorum-sensing genes in *Pseudomonas aeruginosa* biofilms: their role and expression patterns. Appl. Environ. Microbiol..

[10] Gupta RK, Chhibber S, Harjai K (2013). Quorum sensing signal molecules cause renal tissue inflammation through local cytokine responses in experimental UTI caused by *Pseudomonas aeruginosa*. Immunobiology.

[11] Gupta RK, Harjai K, Chhibber S (2016). Rhl quorum sensing affects the virulence potential of *Pseudomonas aeruginosa* in an experimental urinary tract infection. Antonie Van Leeuwenhoek.

[12] Pearson JP, Feldman M, Iglewski BH, Prince A (2000). *Pseudomonas aeruginosa* cell-to-cell signaling is required for virulence in a model of acute pulmonary infection. Infect. Immun..

[13] Rumbaugh KP, Griswold JA, Hamood AN (1999). *Pseudomonas aeruginosa* strains obtained from patients with tracheal, urinary tract and wound infection: variations in virulence factors and virulence genes. J. Hosp. Infect..

[14] Gill EE, Franco OL, Hancock RE (2015). Antibiotic adjuvants: diverse strategies for controlling drug-resistant pathogens. Chem. Biol. Drug Des..

[15] Castillo-Juarez I (2015). Role of quorum sensing in bacterial infections. World J. Clin. Cases.

[16] Welsh MA, Blackwell HE (2016). Chemical probes of quorum sensing: from compound development to biological discovery. FEMS Microbiol. Rev..

[17] Teplitski M, Mathesius U, Rumbaugh KP (2011). Perception and degradation of N-acyl homoserine lactone quorum sensing signals by mammalian and plant cells. Chem. Rev..

[18] Aybey A, Demirkan E (2016). Inhibition of quorum sensing-controlled virulence factors in *Pseudomonas aeruginosa* by human serum paraoxonase. J. Med. Microbiol..

[19] Ozer EA (2005). Human and murine paraoxonase 1 are host modulators of *Pseudomonas aeruginosa* quorum-sensing. FEMS Microbiol. Lett..

[20] Stoltz DA (2007). Paraoxonase-2 deficiency enhances *Pseudomonas aeruginosa* quorum sensing in murine tracheal epithelia. Am. J. Physiol..

[21] Cole SJ, Records AR, Orr MW, Linden SB, Lee VT (2014). Catheter-associated urinary tract infection by *Pseudomonas aeruginosa* is mediated by exopolysaccharide-independent biofilms. Infect. Immun..

[22] Whitchurch CB, Tolker-Nielsen T, Ragas PC, Mattick JS (2002). Extracellular DNA required for bacterial biofilm formation. Science.

[23] Friedman L, Kolter R (2004). Genes involved in matrix formation in *Pseudomonas aeruginosa* PA14 biofilms. Mol. Microbiol..

[24] Wozniak DJ (2003). Alginate is not a significant component of the extracellular polysaccharide matrix of PA14 and PAO1 *Pseudomonas aeruginosa* biofilms. Proc. Natl. Acad. Sci. USA.

[25] Wagner VE, Bushnell D, Passador L, Brooks AI, Iglewski BH (2003). Microarray analysis of *Pseudomonas aeruginosa* quorum-sensing regulons: effects of growth phase and environment. J. Bacteriol..

[26] Schuster M, Lostroh CP, Ogi T, Greenberg EP (2003). Identification, timing, and signal specificity of *Pseudomonas aeruginosa* quorum-controlled genes: a transcriptome analysis. J. Bacteriol..

[27] Gambello MJ, Iglewski BH (1991). Cloning and characterization of the *Pseudomonas aeruginosa lasR* gene, a transcriptional activator of elastase expression. J. Bacteriol..

[28] Gambello MJ, Kaye S, Iglewski BH (1993). LasR of *Pseudomonas aeruginosa* is a transcriptional activator of the alkaline protease gene (*apr*) and an enhancer of exotoxin A expression. Infect. Immun..

[29] Latifi A (1995). Multiple homologues of LuxR and LuxI control expression of virulence determinants and secondary metabolites through quorum sensing in *Pseudomonas aeruginosa* PAO1. Mol. Microbiol..

[30] Brint JM, Ohman DE (1995). Synthesis of multiple exoproducts in *Pseudomonas aeruginosa* is under the control of RhlR-RhlI, another set of regulators in strain PAO1 with homology to the autoinducer-responsive LuxR-LuxI family. J. Bacteriol..

[31] Pearson JP, Pesci EC, Iglewski BH (1997). Roles of *Pseudomonas aeruginosa las* and *rhl* quorum-sensing systems in control of elastase and rhamnolipid biosynthesis genes. J. Bacteriol..

[32] Farrow JM (2008). PqsE functions independently of PqsR-*Pseudomonas* quinolone signal and enhances the *rhl* quorum-sensing system. J. Bacteriol..

[33] Schuster M, Urbanowski ML, Greenberg EP (2004). Promoter specificity in *Pseudomonas aeruginosa* quorum sensing revealed by DNA binding of purified LasR. Proc. Natl. Acad. Sci. USA.

[34] Gilbert KB, Kim TH, Gupta R, Greenberg EP, Schuster M (2009). Global position analysis of the *Pseudomonas aeruginosa* quorum-sensing transcription factor LasR. Mol. Microbiol..

[35] Cole SJ, Lee VT (2015). Cyclic di-GMP signaling contributes to *Pseudomonas aeruginosa*-mediated catheter-associated urinary tract infection. J. Bacteriol..

[36] Ledizet M (2012). The ability of virulence factor expression by *Pseudomonas aeruginosa* to predict clinical disease in hospitalized patients. PLoS ONE.

[37] Smith EE (2006). Genetic adaptation by *Pseudomonas aeruginosa* to the airways of cystic fibrosis patients. Proc. Natl. Acad. Sci. USA.

[38] Flores-Mireles AL, Walker JN, Caparon M, Hultgren SJ (2015). Urinary tract infections: epidemiology, mechanisms of infection and treatment options. Nat. Rev. Microbiol..

[39] Subashchandrabose S (2014). Host-specific induction of *Escherichia coli* fitness genes during human urinary tract infection. Proc. Natl. Acad. Sci. USA.

[40] Michael B, Smith JN, Swift S, Heffron F, Ahmer BM (2001). SdiA of *Salmonella enterica* is a LuxR homolog that detects mixed microbial communities. J. Bacteriol..

[41] Ahmer BM, van Reeuwijk J, Timmers CD, Valentine PJ, Heffron F (1998). *Salmonella typhimurium* encodes an SdiA homolog, a putative quorum sensor of the LuxR family, that regulates genes on the virulence plasmid. J. Bacteriol..

[42] Sabag-Daigle A, Ahmer BM (2012). ExpI and PhzI are descendants of the long lost cognate signal synthase for SdiA. PLoS ONE.

[43] Monnet V, Juillard V, Gardan R (2016). Peptide conversations in Gram-positive bacteria. Crit. Rev. Microbiol..

[44] Rumbaugh KP, Griswold JA, Iglewski BH, Hamood AN (1999). Contribution of quorum sensing to the virulence of *Pseudomonas aeruginosa* in burn wound infections. Infect. Immun..

[45] D’Argenio DA (2007). Growth phenotypes of *Pseudomonas aeruginosa lasR* mutants adapted to the airways of cystic fibrosis patients. Mol. Microbiol..

[46] Hoffman LR (2009). *Pseudomonas aeruginosa lasR* mutants are associated with cystic fibrosis lung disease progression. J. Cyst. Fibros..

[47] Feliziani Sofía, Luján Adela M., Moyano Alejandro J., Sola Claudia, Bocco José L., Montanaro Patricia, Canigia Liliana Fernández, Argaraña Carlos E., Smania Andrea M. (2010). Mucoidy, Quorum Sensing, Mismatch Repair and Antibiotic Resistance in Pseudomonas aeruginosa from Cystic Fibrosis Chronic Airways Infections. PLoS ONE.

[48] Feliziani S (2014). Coexistence and within-host evolution of diversified lineages of hypermutable *Pseudomonas aeruginosa* in long-term cystic fibrosis infections. PLoS Genet..

[49] Oliver A, Canton R, Campo P, Baquero F, Blazquez J (2000). High frequency of hypermutable *Pseudomonas aeruginosa* in cystic fibrosis lung infection. Science.

[50] Martin DW, Schurr MJ, Mudd MH, Deretic V (1993). Differentiation of *Pseudomonas aeruginosa* into the alginate-producing form: inactivation of *mucB* causes conversion to mucoidy. Mol. Microbiol..

[51] Goldberg JB, Gorman WL, Flynn JL, Ohman DE (1993). A mutation in *algN* permits trans activation of alginate production by *algT* in *Pseudomonas* species. J. Bacteriol..

[52] Martin DW (1993). Mechanism of conversion to mucoidy in *Pseudomonas aeruginosa* infecting cystic fibrosis patients. Proc. Natl. Acad. Sci. USA.

[53] Harrison F, Muruli A, Higgins S, Diggle SP (2014). Development of an ex vivo porcine lung model for studying growth, virulence, and signaling of *Pseudomonas aeruginosa*. Infect. Immun..

[54] Sandoz KM, Mitzimberg SM, Schuster M (2007). Social cheating in *Pseudomonas aeruginosa* quorum sensing. Proc. Natl. Acad. Sci. USA.

[55] Liberati NT (2006). An ordered, nonredundant library of *Pseudomonas aeruginosa* strain PA14 transposon insertion mutants. Proc. Natl. Acad. Sci. USA.

[56] Hoang TT, Karkhoff-Schweizer RR, Kutchma AJ, Schweizer HP (1998). A broad-host-range Flp-FRT recombination system for site-specific excision of chromosomally-located DNA sequences: application for isolation of unmarked *Pseudomonas aeruginosa* mutants. Gene.

[57] Langmead B, Trapnell C, Pop M, Salzberg SL (2009). Ultrafast and memory-efficient alignment of short DNA sequences to the human genome. Genome Biol..

[58] Essar DW, Eberly L, Hadero A, Crawford IP (1990). Identification and characterization of genes for a second anthranilate synthase in *Pseudomonas aeruginosa*: interchangeability of the two anthranilate synthases and evolutionary implications. J. Bacteriol..

[59] Brown MR, Foster JH (1970). A simple diagnostic milk medium for *Pseudomonas aeruginosa*. J. Clin. Pathol..

[60] Pinzon NM, Ju LK (2009). Analysis of rhamnolipid biosurfactants by methylene blue complexation. Appl. Microbiol. Biotechnol..

[61] International, A. *v. (loose-leaf)* (AOAC International, Arlington, VA, 1995).

[62] Pearson JP (1994). Structure of the autoinducer required for expression of *Pseudomonas aeruginosa* virulence genes. Proc. Natl. Acad. Sci. USA.

[63] Miller, J. H. *Experiments in Molecular Genetics* (Cold Spring Harbor Laboratory, Cold Spring Harbor, NY, 1972).

