# Supplementary material for: Host suppression of quorum sensing during catheter-associated urinary tract infections
Source: Nat Commun. 2018 Oct 25;9:4436. doi: 10.1038/s41467-018-06882-y (PMC6202348; doi:10.1038/s41467-018-06882-y)
Supplement: Supplementary file 1 — Supplementary Information [file 41467_2018_6882_MOESM1_ESM.pdf]

Supplemental information for

Host suppression of quorum sensing during catheter associated urinary tract infections

Stephanie J. Cole[1], Cherisse L. Hall[1], Maren Schniederberend[2], John M. Farrow III[3], Jonathan R. Goodson[1], Everett C. Pesci[3], Barbara I. Kazmierczak[2], Vincent T. Lee[1*]

Affiliations:

[1] Department of Cell Biology and Molecular Genetics, University of Maryland, College Park, College Park, MD 20742, USA

[2] Department of Microbial Pathogenesis, Yale University School of Medicine, New Haven, CT 06520, USA

[3] Department of Microbiology and Immunology, The Brody School of Medicine at East Carolina University, Greenville, North Carolina, USA.

Stephanie J. Cole and Cherisse L. Hall contributed equally to this work

[*]For correspondence: vtlee@umd.edu

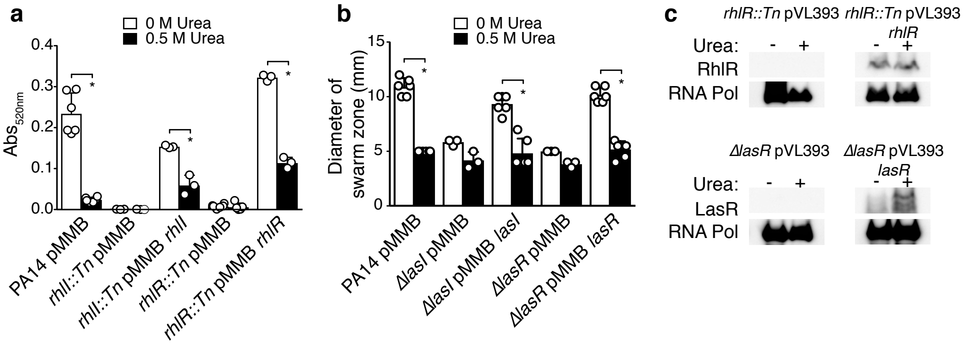

Supplementary Figure 1. **Overexpression of *las* and *rhl* genes does not restore quorum-related phenotypes in response to urea.** *lasR*, *lasI*, *rhlR* and *rhlI* genes were placed on pMMB plasmid under the control of an IPTG inducible promoter. All strains were induced with 1 mM IPTG. (**a**) Quantification of pyocyanin produced by indicated strains grown in LB with or without 0.5 M urea. (**b**) Diameter of swarm zone of indicated strains grown on 0.5% LB agar with and without 0.5 M urea. Data represent the mean and standard deviation of at least 3 independent replicates and analyzed by unpaired t-test. Asterisk (*) indicates $p < 0.05$. (**c**) Western blot to detect the presence of either RhlR or LasR protein in *rhlR::Tn* (empty vector) and *rhlR::Tn* (*rhlR*) or Δ*lasR* (empty vector) and Δ*lasR* (*lasR*), respectively, in the presence or absence of 0.5 M urea.

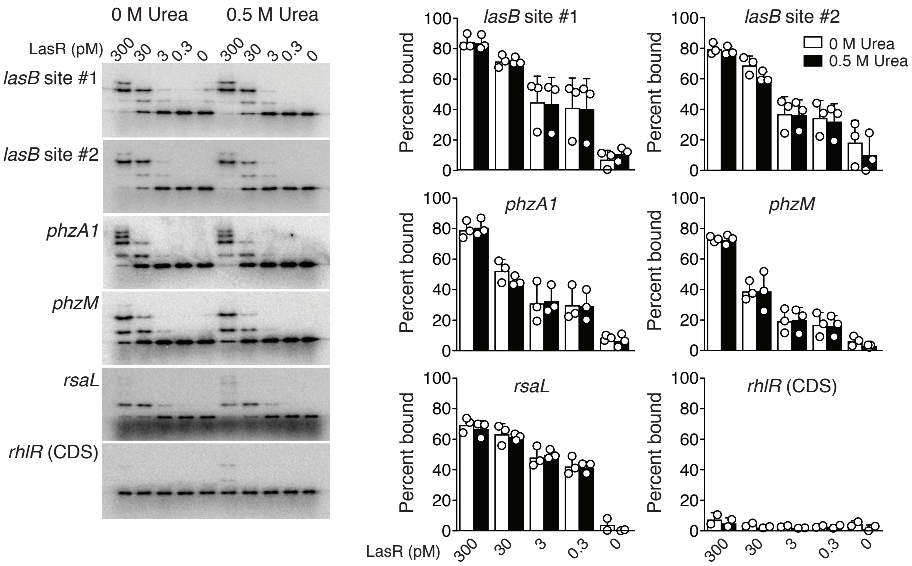

Supplementary Figure 2. **Urea does not affect LasR protein binding to the promoter regions of LasR regulated genes.** Electrophoretic mobility shift assay (EMSA) of LasR protein binding to ^{32}P labeled LasR-binding sites in the promoter regions of *lasB*, *rsaL*, *phzA1* and negative control sequence (coding sequence of *rhlR*). Reaction mixes contained 0.3-300 pM LasR protein, 5 μM 3-oxo-C_{12}-HSL, 1x binding buffer and either 0 M or 0.5 M urea. Radiolabeled DNA was added last to the mix and reactions were incubated for 10 minutes at room temperature prior to running samples on non-denaturing PAGE gels [1, 2]. Data represent the mean and standard deviation of at least 3 independent replicates.

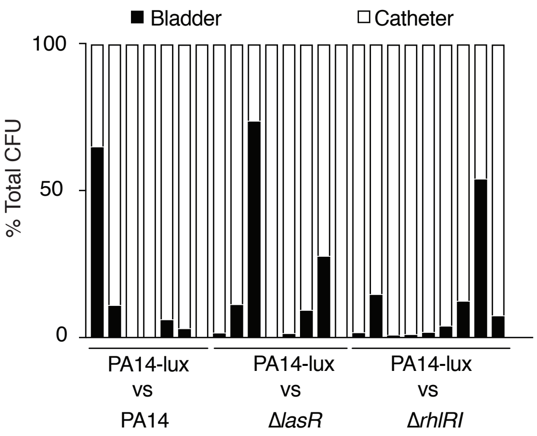

Supplementary Figure 3. ***P. aeruginosa*** **predominantly resides in the catheter during murine catheter associated urinary tract infections.** Colony forming units (CFU) per bladder or catheter were determined by isolating bladders and catheters from mice infected with PA14/PA14-lux, PA14-lux/Δ*lasR* or PA14-lux/Δ*rhlR*I as indicated. Bladders or catheters were individually macerated, serially diluted and plated on LB agar. Each bar represents the fraction of bacterial burden from the bladder and catheter that was isolated from one infected mouse.

Supplementary Table 1. Genes with a fold change ≤-4 when grown in human urine, mouse urine and LB with urea. Gene in red are those identified in Schuster *et al.* and Wagner *et al.*

Locus ID	Gene Name	Human Urine		Mouse Urine		Mouse Bladder		Urea	
		Log2 Fold Change	padj	Log2 Fold Change	padj	Log2 Fold Change	padj	Log2 Fold Change	padj
PA14_00630		-3.707	9.3E-18	-5.952	6.5E-34	-1.517	3.6E-03	-3.627	4.7E-10
PA14_00650		-4.325	2.5E-07	-5.581	7.8E-12	-2.264	2.1E-02	-5.150	5.4E-88
PA14_01330		-2.223	1.3E-16	-3.502	4.6E-39	-0.162	7.0E-01	-3.484	3.4E-23
PA14_01490		-4.169	9.2E-77	-6.613	3.5E-179	-0.563	7.1E-02	-4.279	2.8E-82
PA14_02220		-2.177	1.9E-18	-4.456	7.3E-68	0.648	4.8E-02	-2.660	3.6E-35
PA14_02230	*cheW*	-2.027	1.3E-10	-4.525	1.0E-34	0.638	1.1E-01	-2.488	2.8E-27
PA14_05840	*gcdH*	-2.592	2.7E-27	-5.770	1.2E-126	0.378	2.8E-01	-4.291	1.6E-17
PA14_07430		-2.595	1.6E-20	-3.946	3.7E-46	-4.236	1.0E-31	-2.700	2.4E-36
PA14_07660		-2.066	4.6E-22	-3.270	2.1E-54	1.961	3.2E-14	-3.023	1.4E-40
PA14_07680		-2.312	5.2E-25	-3.369	6.3E-53	1.592	1.2E-08	-3.060	5.1E-47
PA14_09400	*phzS*	-5.331	4.5E-124	-7.252	3.1E-215	-7.526	3.3E-128	-4.893	1.5E-99
PA14_09410	*phzG1*	-5.140	7.4E-54	-6.083	1.5E-68	-6.332	9.9E-33	-5.305	8.3E-74
PA14_09440	*phzE1*	-5.062	1.9E-49	-6.712	4.8E-71	-5.707	1.7E-33	-4.104	3.9E-71
PA14_09450	*phzD1*	-5.284	2.3E-15	-6.534	4.7E-17	-5.800	1.8E-10	-4.059	2.1E-58
PA14_09460	*phzC1*	-4.521	8.9E-32	-7.480	1.3E-58	-6.754	1.8E-29	-4.273	2.0E-59
PA14_09470	*phzB1*	-4.648	1.5E-95	-8.446	5.4E-204	-7.009	2.5E-99	-3.056	5.9E-44
PA14_09480	*phzA1*	-4.752	2.5E-43	-8.136	1.4E-57	-7.162	3.5E-29	-3.001	1.1E-36
PA14_09490	*phzM*	-3.733	1.2E-56	-4.099	2.9E-70	-4.041	4.1E-41	-2.285	3.4E-25
PA14_09900	*prpL*	-4.360	1.3E-60	-5.137	1.1E-83	-4.643	2.3E-42	-3.131	3.7E-43
PA14_10360		-3.628	5.1E-23	-5.738	6.3E-55	1.148	1.7E-02	-3.537	1.2E-13
PA14_10380		-3.629	1.5E-14	-4.291	3.6E-20	0.383	5.5E-01	-2.574	6.8E-05
PA14_10490		-2.590	5.6E-09	-3.895	8.7E-17	-3.418	7.1E-08	-5.492	1.1E-09
PA14_10500		-3.251	2.7E-22	-6.441	9.4E-84	-4.201	3.1E-25	-5.005	5.7E-22
PA14_10530		-3.238	2.8E-41	-5.882	4.1E-121	-5.814	3.3E-67	-4.089	3.3E-24
PA14_10540		-3.518	5.0E-48	-7.210	7.6E-167	-5.505	2.9E-69	-6.043	5.2E-129
PA14_11140		-2.992	2.4E-19	-3.331	4.9E-25	-1.152	4.2E-03	-3.081	2.8E-39
PA14_13210		-2.953	1.5E-07	-3.614	1.2E-10	-0.535	4.5E-01	-5.603	2.9E-15
PA14_13260	*moaB1*	-2.214	8.5E-06	-2.707	9.7E-09	-2.409	9.1E-05	-2.158	1.5E-12
PA14_14540		-2.518	1.8E-17	-3.820	4.3E-39	-3.627	2.3E-22	-6.442	9.9E-34
PA14_14550		-2.022	2.3E-15	-2.759	1.5E-28	-2.652	1.5E-17	-6.091	1.3E-22
PA14_16100		-2.823	4.0E-15	-3.039	1.2E-18	-1.776	5.0E-05	-4.842	1.5E-87
PA14_16250	*lasB*	-3.712	1.5E-67	-6.166	4.7E-181	-0.839	3.3E-03	-3.516	1.8E-59
PA14_16260		-2.911	6.0E-23	-4.758	9.9E-55	0.163	7.3E-01	-4.079	4.2E-73
PA14_16680		-2.004	8.5E-10	-4.133	5.3E-29	0.634	1.2E-01	-4.224	6.8E-69
PA14_18800		-3.104	6.4E-16	-4.949	1.3E-39	2.064	1.4E-05	-5.052	5.8E-47
PA14_19100	*rhlA*	-2.426	7.5E-24	-4.720	1.0E-81	1.147	1.5E-04	-4.417	1.0E-85
PA14_19110	*rhlB*	-2.813	1.0E-26	-4.741	3.9E-69	0.667	5.6E-02	-4.698	5.9E-94
PA14_19480		-2.697	1.3E-12	-3.556	1.3E-21	2.646	8.9E-10	-3.718	2.5E-11
PA14_19870	*ldh*	-2.352	7.6E-17	-3.541	5.0E-36	2.066	1.4E-10	-4.548	4.1E-18
PA14_19900		-2.100	9.1E-08	-2.627	7.8E-12	3.881	6.3E-26	-4.393	2.6E-81
PA14_20610	*lecB*	-4.290	5.7E-42	-5.359	1.3E-57	1.349	9.6E-05	-4.953	1.9E-86
PA14_21670		-2.339	5.7E-06	-3.303	1.4E-10	0.522	4.2E-01	-3.161	2.6E-34
PA14_24650	*rmf*	-3.708	3.3E-44	-5.058	6.1E-81	1.566	2.8E-06	-4.296	1.3E-06
PA14_24770		-2.117	8.9E-17	-3.035	2.3E-34	-0.198	6.2E-01	-2.303	1.2E-06
PA14_26020		-5.024	4.4E-87	-7.163	2.1E-155	-2.215	7.2E-13	-5.031	1.3E-100
PA14_26780		-2.038	5.4E-08	-2.145	1.8E-09	-0.972	4.1E-02	-2.254	8.6E-26

**PA14_28050**		-3.445	3.7E-35	-4.687	8.8E-62	0.352	3.7E-01	-3.735	1.5E-61
**PA14_28060**	*cpg2*	-2.872	4.9E-08	-4.425	6.5E-14	-0.057	9.4E-01	-3.689	8.6E-34
**PA14_28140**		-2.838	2.7E-12	-3.061	3.6E-15	0.338	5.2E-01	-3.736	7.7E-50
**PA14_28610**		-2.137	2.5E-05	-2.666	2.8E-08	0.149	8.5E-01	-3.236	3.2E-06
**PA14_29330**		-2.710	1.0E-25	-4.384	4.2E-60	-0.463	1.9E-01	-4.928	4.5E-94
**PA14_30620**		-2.545	3.8E-25	-3.488	1.1E-46	-2.065	1.1E-11	-2.084	7.9E-23
**PA14_30830**		-2.299	1.9E-21	-3.530	9.9E-48	1.532	6.6E-08	-4.163	1.7E-62
**PA14_31290**	*pa1L*	-4.734	3.0E-26	-5.482	3.2E-34	0.169	8.0E-01	-6.437	1.6E-12
**PA14_31350**		-2.993	3.2E-15	-3.171	5.5E-18	0.100	8.7E-01	-3.043	1.9E-42
**PA14_32950**		-2.336	1.1E-09	-3.910	9.6E-23	-2.180	6.1E-06	-2.239	7.5E-14
**PA14_33160**		-2.277	3.1E-16	-2.095	1.7E-15	1.845	1.6E-09	-3.911	3.8E-66
**PA14_33870**		-3.362	6.5E-21	-4.594	1.2E-37	-0.988	3.3E-02	-3.326	4.3E-13
**PA14_33970**		-2.124	1.3E-07	-2.044	5.8E-08	-0.138	8.2E-01	-2.411	6.5E-20
**PA14_33980**		-3.065	4.3E-09	-3.238	7.1E-11	-1.392	2.3E-02	-3.457	3.7E-10
**PA14_33990**		-2.792	1.1E-12	-3.391	1.0E-18	-0.874	7.5E-02	-3.987	2.6E-16
**PA14_34000**		-3.466	3.0E-08	-3.652	1.0E-09	-1.303	6.6E-02	-4.676	1.3E-30
**PA14_34010**		-2.666	8.5E-10	-3.385	3.7E-15	-0.914	8.9E-02	-3.611	4.7E-17
**PA14_34020**		-3.353	3.8E-03	-3.458	1.5E-03	-1.129	3.8E-01	-4.479	1.1E-05
**PA14_34030**		-2.894	3.1E-19	-4.265	6.5E-38	-1.063	8.5E-03	-3.616	2.7E-45
**PA14_34050**		-3.008	1.7E-27	-4.113	4.0E-49	-0.402	2.9E-01	-2.811	2.8E-22
**PA14_34070**		-2.322	6.8E-03	-3.039	1.2E-04	0.580	6.1E-01	-2.931	1.5E-30
**PA14_34140**		-2.654	6.5E-11	-3.835	4.1E-19	-1.186	1.6E-02	-3.084	2.0E-10
**PA14_34870**	*chiC*	-3.922	3.3E-47	-4.623	3.1E-65	1.776	1.3E-08	-2.861	6.3E-38
**PA14_36450**		-2.146	3.5E-12	-2.188	6.8E-14	1.003	4.9E-03	-2.647	6.0E-34
**PA14_36460**		-2.540	2.3E-11	-2.071	5.5E-09	1.387	1.3E-03	-2.901	6.8E-32
**PA14_36470**		-2.489	2.1E-15	-3.219	3.4E-25	0.535	1.8E-01	-3.463	9.1E-40
**PA14_36620**		-2.613	1.4E-05	-2.481	6.9E-06	2.000	6.2E-04	-3.729	8.3E-07
**PA14_36790**		-2.066	2.7E-02	-2.024	1.6E-02	0.356	7.5E-01	-2.319	1.2E-02
**PA14_36850**		-2.568	1.3E-10	-3.074	2.5E-15	2.010	2.5E-06	-2.948	3.1E-25
**PA14_36860**		-2.652	4.1E-06	-4.822	1.4E-11	1.182	4.7E-02	-5.626	1.3E-43
**PA14_37690**		-2.841	6.3E-24	-2.884	9.4E-27	-0.266	5.1E-01	-3.464	9.0E-53
**PA14_37745**		-5.817	5.9E-58	-7.925	5.0E-62	-3.256	4.0E-17	-3.240	1.8E-31
**PA14_37760**		-4.193	5.1E-38	-4.871	9.8E-49	-1.549	2.0E-05	-2.556	1.8E-24
**PA14_37770**		-4.753	1.8E-18	-4.842	1.1E-20	-3.435	3.3E-08	-2.366	5.0E-11
**PA14_37780**		-4.452	1.2E-28	-4.594	1.3E-32	-2.753	9.3E-10	-2.409	1.3E-03
**PA14_39270**		-2.075	2.7E-09	-3.341	6.9E-21	0.136	8.0E-01	-2.498	4.6E-23
**PA14_39560**		-2.979	8.2E-24	-3.679	6.3E-36	2.072	2.5E-10	-5.619	1.1E-117
**PA14_39780**		-2.404	1.1E-10	-2.154	3.8E-10	0.989	1.7E-02	-2.944	2.3E-31
**PA14_39880**	*phzG2*	-6.498	2.6E-36	-7.540	9.0E-36	-5.954	7.2E-19	-5.325	7.1E-55
**PA14_39945**	*phzC2*	-5.839	7.3E-52	-8.004	4.9E-53	-6.886	3.6E-27	-4.032	2.2E-62
**PA14_39960**	*phzB2*	-5.688	3.1E-139	-8.030	1.3E-236	-6.091	5.6E-100	-4.001	1.4E-68
**PA14_39970**	*phzA2*	-5.705	4.7E-101	-9.060	1.3E-142	-7.187	3.7E-74	-3.919	4.5E-33
**PA14_40010**		-3.199	1.4E-18	-5.321	1.1E-34	-3.714	1.8E-13	-2.096	5.4E-09
**PA14_40020**		-3.650	7.6E-28	-6.167	6.0E-64	-3.659	1.0E-18	-2.660	1.2E-23
**PA14_40030**		-3.445	6.1E-28	-6.080	1.1E-58	-4.188	8.3E-23	-2.328	3.8E-12
**PA14_40100**		-2.226	3.4E-09	-2.840	8.3E-15	-0.471	3.5E-01	-2.903	5.5E-35
**PA14_40110**		-2.033	1.1E-03	-2.391	3.9E-05	0.227	7.9E-01	-2.831	3.4E-20
**PA14_40290**	*lasA*	-4.111	2.8E-36	-5.804	1.1E-55	1.045	5.6E-03	-4.578	1.0E-47
**PA14_40750**		-2.507	7.5E-14	-3.642	3.7E-28	0.056	9.2E-01	-3.982	1.3E-64
**PA14_41500**		-2.697	4.4E-16	-2.867	3.6E-19	0.409	3.8E-01	-4.822	5.0E-22
**PA14_41980**		-2.502	1.7E-12	-2.288	7.2E-12	0.444	3.4E-01	-3.699	1.4E-09

**PA14_43250**		-2.502	1.5E-11	-3.347	1.0E-19	-0.678	1.6E-01	-3.390	3.8E-45
**PA14_46520**		-2.122	7.3E-16	-3.601	3.6E-44	-1.998	6.8E-10	-2.516	9.9E-33
**PA14_46760**		-2.045	8.0E-04	-2.509	1.8E-05	2.064	2.4E-04	-3.781	2.2E-26
**PA14_47120**		-2.489	1.8E-11	-3.472	6.1E-22	-0.560	2.8E-01	-2.677	2.9E-14
**PA14_47130**		-2.019	1.8E-14	-2.841	1.8E-28	0.041	9.3E-01	-3.034	1.9E-45
**PA14_48060**	*aprA*	-4.010	3.3E-71	-7.555	1.2E-240	-3.094	6.0E-29	-3.975	1.7E-72
**PA14_48760**		-2.316	1.7E-12	-3.089	1.3E-21	-1.859	5.9E-06	-2.801	3.9E-15
**PA14_49200**	*oprH*	-5.595	6.8E-86	-3.235	7.0E-30	-1.477	5.9E-05	-2.121	2.7E-10
**PA14_49210**	*napE*	-2.571	1.4E-15	-4.633	4.8E-42	0.227	6.4E-01	-3.024	1.2E-42
**PA14_49220**	*napF*	-2.285	1.0E-08	-3.441	2.0E-17	1.221	7.0E-03	-3.243	1.6E-33
**PA14_49230**	*napD*	-2.179	8.3E-13	-3.731	3.1E-34	0.461	2.7E-01	-2.331	1.4E-06
**PA14_49250**	*napA*	-2.310	2.0E-25	-3.941	1.4E-70	0.090	8.1E-01	-2.350	1.3E-28
**PA14_49260**	*napB*	-2.133	5.8E-11	-3.706	7.7E-28	-0.709	9.3E-02	-2.829	4.1E-34
**PA14_49270**	*napc*	-2.075	5.2E-10	-3.303	1.2E-23	-0.774	7.5E-02	-3.146	2.2E-20
**PA14_49330**		-2.170	1.9E-10	-3.455	6.2E-23	-0.551	2.2E-01	-3.674	7.2E-55
**PA14_49750**		-2.197	2.5E-07	-3.959	2.0E-17	0.691	1.8E-01	-2.456	4.4E-21
**PA14_49760**	*rhlC*	-3.562	1.9E-12	-4.147	1.3E-16	-1.186	4.7E-02	-2.803	3.6E-24
**PA14_50880**		-3.301	6.5E-32	-5.757	6.9E-87	-2.325	1.7E-11	-5.272	1.8E-15
**PA14_53250**	*cpbD*	-3.698	3.3E-59	-5.992	1.1E-144	-1.835	1.0E-10	-3.747	2.5E-64
**PA14_53530**		-3.328	3.1E-29	-5.024	1.2E-64	-4.477	1.0E-33	-3.538	1.4E-52
**PA14_54080**		-2.495	9.8E-16	-3.277	2.6E-26	0.826	2.7E-02	-4.739	4.4E-87
**PA14_55110**		-3.044	2.7E-24	-5.161	6.0E-65	-3.236	1.6E-18	-5.147	7.4E-53
**PA14_55790**		-2.261	1.5E-09	-2.857	5.5E-15	1.066	1.2E-02	-3.766	4.3E-58
**PA14_55850**		-2.501	2.1E-04	-2.904	6.1E-06	-1.271	1.1E-01	-3.250	1.3E-14
**PA14_55900**		-2.070	1.7E-05	-2.220	8.1E-07	-0.485	4.5E-01	-2.065	4.2E-11
**PA14_55940**		-3.975	1.4E-14	-5.854	1.2E-18	1.122	2.6E-02	-6.102	6.7E-102
**PA14_56730**		-2.025	1.3E-13	-3.362	6.0E-35	-0.277	4.9E-01	-2.689	1.4E-34
**PA14_60750**	*pra*	-2.445	3.0E-23	-5.165	1.6E-92	-1.300	3.0E-05	-3.458	2.9E-45
**PA14_60960**		-3.520	8.6E-19	-5.596	3.1E-46	-3.586	9.3E-14	-5.148	1.7E-107
**PA14_61190**		-2.257	2.6E-19	-2.336	8.3E-22	1.102	3.5E-04	-3.269	1.9E-49
**PA14_61200**		-2.532	1.4E-27	-2.444	6.2E-27	1.823	7.3E-11	-2.402	2.1E-29
**PA14_61380**		-3.423	1.1E-39	-4.730	8.3E-75	0.243	5.5E-01	-3.676	7.0E-21
**PA14_64480**	*osmE*	-2.344	1.1E-06	-3.156	7.3E-12	-1.323	2.7E-02	-3.021	1.8E-10
**PA14_65090**		-2.565	2.2E-16	-4.066	2.3E-35	0.696	7.4E-02	-3.594	7.1E-56
**PA14_66840**	*phaC2*	-2.736	7.4E-19	-3.557	6.3E-32	-1.748	5.3E-06	-2.425	1.0E-29
**PA14_67350**	*hutU*	-2.289	4.6E-20	-4.628	2.0E-74	-4.728	4.2E-44	-2.104	2.7E-19
**PA14_67370**		-2.529	8.3E-04	-2.029	2.9E-03	-0.567	5.5E-01	-3.860	1.4E-26
**PA14_68940**		-2.378	2.0E-23	-5.444	1.3E-92	-2.827	4.7E-21	-2.123	7.7E-23
**PA14_70740**		-2.210	1.2E-15	-3.297	1.4E-33	-0.264	5.2E-01	-3.063	5.6E-44

Supplementary Table 2. Comparison of sequences of *lasI*, *lasR*, *rhlR*, and *rhlI* genes from clinical isolates to PA14. Bolded cells indicate mutations that is not found in the coding sequence in the 1,636 strains of *P. aeruginosa* available at pseudomonas.com[3].

	**Amino acid sequence of LasR[a]**	**Amino acid sequence of LasI[a]**	**Amino acid sequence of RhlR[a]**	**Amino acid sequence of RhlI[b]**
# 1022	PA14	PA14	PA14	*rhlI (D83E, G127A)*
# 1052	PA14	PA14	PA14	*rhlI (D83E, G127A)*
# 1102	PA14	PA14	PA14	*rhlI (D83E, G127A)*
# 1181	PA14	PA14	***rhlR (P56A)***	*rhlI (D83E, G127A)*
# 1219	PA14	PA14	PA14	*rhlI (D83E, G127A)*
# 1229	PA14	PA14	PA14	*rhlI (D83E, G127A)*
# 1251	PA14	PA14	PA14	*rhlI (D83E, G127A)*
# 1254	PA14	PA14	PA14	*rhlI (D83E, G127A)*
# 1271	PA14	PA14	PA14	*rhlI (D83E, G127A)*
# 1275	***lasR (stop186)***	PA14	PA14	*rhlI (D83E, G127A)*
# 1277	PA14	PA14	PA14	*rhlI (D83E, G127A)*
# 1376	PA14	PA14	***rhlR (stop67)***	*rhlI (D83E, G127A)*
# 1441	PA14	PA14	PA14	*rhlI (D83E, G127A)*
# 1457	PA14	PA14	***rhlR (stop74)***	PA14
# 1473	PA14	PA14	PA14	*rhlI (D83E, G127A)*

[a] Legend: "PA14" denotes wild-type PA14 sequences. Numbers indicate last correct amino acid in indel or nonsense mutation

[b] PAO1 RhlI has alanine at position 127. RhlI from strains 8380, F23197, W60856, AES-1R, 19BR, 213BR, and SCV20265 have glutamate at position 83 and alanine at position 127.

Supplementary Table 3. Primer list**.**

Primer Sequence	Primer Name	Purpose	Source
AAcatatgATCGAATTGCTCTCTGAATCGC	sjc09	ORF *rhlI* Forward	This Study
AaggatccTCACACCGCCATCGACAGC	sjc10	ORF *rhlI* Reverse	This Study
AAcatatgAGGAATGACGGAGGCTTTTTGCTGTG	sjc12	ORF *rhlR* Forward	This Study
AActcgagTCAGATGAGGCCCAGCGCC	sjc13	ORF *rhlR* Reverse	This Study
AAcatatgATCGTACAAATTGGTCGGCGC	sjc15	ORF *lasI* Forward	This Study
AAggatccTCATGAAACCGCCAGTCGCT	sjc16	ORF *lasI* Reverse	This Study
AAggatccATGGCCTTGGTTGACGGTTTTC	sjc18	ORF *lasR* Forward	This Study
AActcgagTCAGAGAGTAATAAGACCCAAATTAAC	sjc19	ORF *lasR* Reverse	This Study
AAgaattcCTGGCGCGAGCATTACGA	KR102	KO *lasI* 5' US	This Study
AAtctagaTTGAGCACGCAACTTGTGCAT	sjc21	KO *lasI* 3' US	This Study
AAtctagaATCGAACTCAATGCCAAGACCC	sjc22	KO *lasI* 5' DS	This Study
TTggatccACTGGATCGACCAGGCCCTGA	KR105	KO *lasI* 3' DS	This Study
ATGGTTATGACGCACTCAGTCCTT	KR106	*lasI* KO Confirm 5'	This Study
ATCATCATCTTCTCCACCCCTACG	KR107	*lasI* KO Confirm 3'	This Study
AAgaattcTGCTCAACAGCCGGCATATCT	KR108	KO *lasR* 5' US	This Study
TTcatatgTCCAATTTTCCACTTGAGCGTTC	KR109	KO *lasR* 3' US	This Study
AAcatatgTAGCGGCCATTATGGCCGT	KR110	KO *lasR* 5' DS	This Study
TTggatccTAAAGCGCGATCTGGGTCTT	KR111	KO *lasR* 3' DS	This Study
TGTTGCCTAAGGACAGCCAGGAC	KR112	*lasR* KO Confirm 5'	This Study
ATCTCCCAACTGGTCTTGCCGATG	KR113	*lasR* KO Confirm 3'	This Study
AAgaattcGCTGAGCGACGAACTGAC	KR114	KO *rhlI-R* 5' US	This Study
TTcatatgTTTTCCAGGACGGCGAACAC	KR115	KO *rhlI-R* 3' US	This Study
AAcatatgATCAGCTTCCCGGCCTACCA	KR116	KO *rhlI-R* 5' DS	This Study
AAaagcttTCTGCTCAGCGATGTGCAA	sjc23	KO *rhlI_R* 3' DS	This Study
ATCCGATGCTGATGTCCAACC	KR118	*rhlI-R* KO Confirm 5'	This Study
GGCGACGATGTAGCGGGTTT	KR119	*rhlI-R* KO Confirm 3'	This Study
CTACAAGCTCGACGTCAACGAAT	sjc45	*lasB* (site 1 of 2)(EMSA)	This Study
AATCTGTATGTTTTCGCTGGAATAG	sjc46	*lasB* (site 1 of 2)(EMSA)	This Study
CGATCATCTTCACTTCCTCCAAA	sjc63	*rsaL* (EMSA) Forward	This Study
GACGTTTCTTCGAGCCTAGCAA	sjc64	*rsaL* (EMSA) Reverse	This Study
TGCAACTGATGATCGTCCACAT	sjc65	*lasB* (site 2 of 2) (EMSA) Forward	This Study
TCGCCGGAAATCTGTATGTTTT	sjc66	*lasB* (site 2 of 2)	This Study

		(EMSA) Reverse	
AGCTTAGCAATCCCGCATAC	sjc67	*phzA1* (EMSA) Forward	This Study
GAAGTGTTTCAAATAGCCAGCA	sjc68	*phzA1* (EMSA) Reverse	This Study
CGCTGATGTGGATTGCATAAAA	sjc69	*phzM* (EMSA) Forward	This Study
GCAGGAAGCATCAGCTTAGCA	sjc70	*phzM* (EMSA) Reverse	This Study
TCTTTTCGGACGTTTCTTCGAG	sjc71	*lasI* (EMSA) Forward	This Study
CACTTCCTCCAAATAGGAAGCTGA	sjc72	*lasI* (EMSA) Reverse	This Study

Supplementary References

1. Schuster M, Urbanowski ML, Greenberg EP. Promoter specificity in *Pseudomonas aeruginosa* quorum sensing revealed by DNA binding of purified LasR. *Proc Natl Acad Sci USA* **101**:15833 (2004)
2. Wurtzel O, Yoder-Himes DR, Han K, Dandekar AA, Edelheit S, Greenberg EP, Sorek R, Lory S. The single-nucleotide resolution transcriptome of *Pseudomonas aeruginosa* grown in body temperature. *PloS Pathog* **8**: e1002945 (2012)
3. Winsor GL, Griffiths EJ, Lo R, Dhillon BK, Shay JA, Brinkman FS. Enhanced annotations and features for comparing thousands of *Pseudomonas* genomes in the *Pseudomonas* genome database. *Nucleic Acids Res* **44:**D646-653. (2016)
