# Supplementary material for: Host suppression of quorum sensing during catheter-associated urinary tract infections
Source: Nat Commun. 2018 Oct 25;9:4436. doi: 10.1038/s41467-018-06882-y (PMC6202348; doi:10.1038/s41467-018-06882-y)
Supplement: Supplementary file 2 — Description of Additional Supplementary Files [file 41467_2018_6882_MOESM2_ESM.pdf]

Description of Additional Supplementary Files

File Name: Supplementary Data 1
Description: RNA Seq data of all genes in the indicated growth conditions.
