# Supplementary material for: Host suppression of quorum sensing during catheter-associated urinary tract infections
Source: Nat Commun. 2018 Oct 25;9:4436. doi: 10.1038/s41467-018-06882-y (PMC6202348; doi:10.1038/s41467-018-06882-y)
Supplement: Supplementary file 3 — Supplementary Data 1 [file 41467_2018_6882_MOESM3_ESM.pdf]

Supplementary Data 1. RNA Seq data of genes that are up- or down-regulated greater than 4-fold

		Human Urine vs. PBS-T		Mouse Urine vs. PBS-T		Mouse Bladder vs PBS-T				LB + 0.5 M Urea vs. LB	
Locus ID	Average normalized reads1	Log2 Fold Change	padj	Log2 Fold Change	padj	Log2 Fold Change	padj	Locus ID	Average normalized reads2	Log2 Fold Change	padj
PA14_00010	13996.561	0.946	0.037	2.248	0.000	0.704	0.203	PA14_00010	15256.997	0.631	0.031
PA14_00020	2137.829	-0.193	0.514	0.099	0.710	-1.169	0.000	PA14_00020	5479.859	0.758	0.000
PA14_00030	7791.740	0.499	0.104	1.168	0.000	0.918	0.007	PA14_00030	12136.858	1.173	0.000
PA14_00050	6836.600	0.042	0.916	0.329	0.204	-0.223	0.534	PA14_00050	8358.326	0.916	0.000
PA14_00060	240.780	-0.002	0.996	0.553	0.056	-0.566	0.167	PA14_00060	539.493	1.009	0.000
PA14_00070	365.644	0.175	0.628	0.442	0.101	0.149	0.721	PA14_00070	1153.872	0.782	0.000
PA14_00080	237.685	-1.305	0.000	-1.963	0.000	1.598	0.000	PA14_00080	640.661	-1.264	0.000
PA14_00090	779.434	0.178	0.624	0.016	0.963	-1.251	0.000	PA14_00090	695.596	1.192	0.000
PA14_00100	2854.589	0.651	0.014	0.908	0.000	0.424	0.206	PA14_00100	3358.239	1.461	0.000
PA14_00110	117.622	-0.032	0.959	-0.103	0.812	-0.064	0.917	PA14_00110	407.794	0.409	0.110
PA14_00120	1012.732	-0.176	0.595	0.066	0.826	0.127	0.744	PA14_00120	2218.594	0.541	0.012
PA14_00130	681.433	1.507	0.000	1.915	0.000	1.622	0.000	PA14_00130	799.160	-0.282	0.261
PA14_00140	48.670	0.073	0.923	0.921	0.038	0.752	0.216	PA14_00140	89.584	1.306	0.000
PA14_00150	33.233	-0.181	0.832	0.034	0.962	-0.719	0.380	PA14_00150	118.324	0.486	0.371
PA14_00160	746.102	0.397	0.380	1.219	0.001	0.910	0.049	PA14_00160	1340.398	-0.293	0.209
PA14_00170	1984.927	0.286	0.323	0.476	0.047	-0.460	0.147	PA14_00170	5226.191	0.274	0.215
PA14_00180	622.382	0.603	0.065	1.064	0.000	0.494	0.215	PA14_00180	1754.457	0.927	0.000
PA14_00190	843.423	-0.030	0.947	0.142	0.649	-0.223	0.584	PA14_00190	2418.228	0.057	0.792
PA14_00200	1234.075	-0.224	0.566	0.220	0.501	-0.279	0.516	PA14_00200	3442.939	-0.642	0.003
PA14_00210	1902.759	0.066	0.899	0.105	0.788	-1.752	0.000	PA14_00210	4021.642	0.805	0.000
PA14_00230	7.327	0.338	0.800	-0.113	0.921	0.244	0.858	PA14_00230	29.752	-0.130	0.873
PA14_00240	168.034	-0.107	0.816	0.401	0.198	-1.309	0.002	PA14_00240	577.908	0.036	1.000
PA14_00250	474.154	-0.134	0.767	-0.824	0.008	-2.734	0.000	PA14_00250	1117.827	0.209	0.395
PA14_00280	1073.548	0.543	0.035	0.653	0.005	-0.078	0.842	PA14_00280	3462.628	0.246	0.275
PA14_00290	176.333	0.423	0.230	0.608	0.039	0.166	0.727	PA14_00290	486.910	0.957	0.000
PA14_00300	399.288	-1.580	0.000	-2.504	0.000	-2.955	0.000	PA14_00300	3965.040	-1.141	0.000
PA14_00310	106.043	-1.699	0.000	-2.460	0.000	-3.201	0.000	PA14_00310	750.841	0.113	0.664
PA14_00320	81.587	-0.765	0.143	-1.726	0.000	-2.120	0.000	PA14_00320	380.349	-0.194	0.479
PA14_00340	11.247	0.717	0.447	0.778	0.318	-0.762	0.504	PA14_00340	24.742	0.905	0.170
PA14_00360	24.111	0.028	0.977	-0.128	0.860	-0.509	0.583	PA14_00360	70.962	1.156	0.003
PA14_00380	63.508	0.121	0.840	0.382	0.354	0.033	0.963	PA14_00380	165.499	0.817	0.007
PA14_00400	122.115	0.810	0.026	1.298	0.000	-0.368	0.496	PA14_00400	399.330	0.828	0.002
PA14_00410	27.991	-0.142	0.874	0.658	0.250	-0.001	0.999	PA14_00410	126.796	0.835	0.009
PA14_00420	31.274	0.593	0.418	1.360	0.014	-0.618	0.502	PA14_00420	45.326	0.813	0.190
PA14_00430	105.852	0.342	0.433	0.694	0.040	0.097	0.869	PA14_00430	224.246	0.732	0.009
PA14_00440	45.453	-1.050	0.047	-0.769	0.104	-2.832	0.000	PA14_00440	154.510	0.798	0.010
PA14_00450	538.606	-0.117	0.810	-0.568	0.090	-1.402	0.001	PA14_00450	1389.385	0.721	0.002
PA14_00460	353.023	1.062	0.019	2.402	0.000	0.297	0.645	PA14_00460	406.746	1.089	0.000
PA14_00470	1784.683	-1.446	0.000	-1.592	0.000	1.445	0.000	PA14_00470	8739.502	-1.925	0.000
PA14_00480	835.252	-1.914	0.000	-2.479	0.000	-0.979	0.016	PA14_00480	3345.766	-1.552	0.000
PA14_00490	233.984	0.882	0.006	1.374	0.000	1.353	0.000	PA14_00490	684.113	1.235	0.000
PA14_00510	773.118	0.216	0.678	-0.193	0.655	-1.387	0.003	PA14_00510	3566.617	-1.285	0.000
PA14_00520	203.757	0.515	0.273	1.342	0.000	1.485	0.001	PA14_00520	622.022	2.231	0.000
PA14_00530	124.662	0.246	0.581	0.846	0.008	0.228	0.661	PA14_00530	293.159	1.076	0.000
PA14_00550	137.919	0.087	0.885	-0.025	0.958	-0.516	0.351	PA14_00550	406.846	-0.588	0.017
PA14_00560	426.414	1.026	0.001	0.603	0.057	-1.828	0.000	PA14_00560	1792.486	-1.004	0.000
PA14_00570	528.748	0.414	0.282	0.401	0.235	-0.245	0.612	PA14_00570	588.218	1.235	0.000
PA14_00580	290.978	0.825	0.036	1.291	0.000	-0.688	0.171	PA14_00580	339.561	0.777	0.018
PA14_00590	53.501	-0.269	0.676	0.102	0.852	-0.871	0.185	PA14_00590	104.018	0.279	0.457
PA14_00600	13.288	-1.372	0.085	-1.433	0.041	-1.900	0.043	PA14_00600	74.157	-1.885	0.000
PA14_00620	190.008	-2.088	0.000	-3.858	0.000	-1.730	0.000	PA14_00620	300.036	-1.310	0.000
PA14_00630	186.744	-3.707	0.000	-5.952	0.000	-1.517	0.004	PA14_00630	426.796	-3.627	0.000
PA14_00640	186.956	-4.036	0.000	-3.591	0.000	-2.180	0.000	PA14_00640	930.422	-1.908	0.000
PA14_00650	184.392	-4.325	0.000	-5.581	0.000	-2.264	0.021	PA14_00650	1164.883	-5.150	0.000

PA14_00660	38.748	0.074	0.928	0.389	0.473	-1.173	0.123	PA14_00660	101.244	0.718	0.102
PA14_00670	1194.447	-0.002	0.997	-0.024	0.955	-0.687	0.125	PA14_00670	1799.534	-0.053	0.844
PA14_00680	27.074	1.293	0.055	2.247	0.000	0.320	0.749	PA14_00680	40.369	1.236	0.029
PA14_00690	102.501	-0.505	0.216	-0.027	0.949	-0.257	0.626	PA14_00690	102.209	-0.286	0.435
PA14_00700	16.549	-0.412	0.644	0.390	0.573	0.304	0.753	PA14_00700	18.865	0.148	0.876
PA14_00710	1009.501	-1.889	0.000	-2.375	0.000	0.058	0.902	PA14_00710	2652.287	-1.833	0.000
PA14_00720	558.264	-0.726	0.006	-0.568	0.023	0.097	0.808	PA14_00720	2036.440	-1.239	0.000
PA14_00730	48.071	0.228	0.715	0.409	0.382	0.029	0.970	PA14_00730	281.728	-1.376	0.000
PA14_00740	83.283	-0.298	0.539	0.511	0.161	-0.111	0.856	PA14_00740	335.914	-0.373	0.162
PA14_00750	180.104	-0.012	0.981	-0.304	0.374	-1.325	0.002	PA14_00750	627.302	0.365	0.152
PA14_00760	97.810	0.237	0.626	0.185	0.647	-0.973	0.062	PA14_00760	272.784	0.161	0.606
PA14_00770	102.790	0.369	0.406	0.152	0.708	-1.009	0.047	PA14_00770	350.311	0.356	0.177
PA14_00780	206.337	0.255	0.551	0.559	0.088	-1.193	0.007	PA14_00780	548.173	-0.293	0.383
PA14_00790	2565.827	-0.132	0.682	-0.575	0.013	-1.033	0.000	PA14_00790	7658.111	-0.697	0.001
PA14_00800	218.578	0.659	0.092	0.466	0.202	-1.062	0.026	PA14_00800	518.088	-0.196	0.470
PA14_00810	65.400	0.366	0.502	1.453	0.000	0.281	0.670	PA14_00810	167.596	-0.736	0.015
PA14_00820	1036.555	-1.043	0.004	-1.473	0.000	-1.909	0.000	PA14_00820	3444.896	0.550	0.010
PA14_00830	2295.664	-0.296	0.340	0.050	0.871	0.094	0.817	PA14_00830	6522.985	-0.657	0.001
PA14_00850	23.246	-0.180	0.834	0.105	0.874	-2.377	0.008	PA14_00850	90.118	-0.463	0.209
PA14_00860	5.629	-0.693	0.579	-0.685	0.506	-1.102	0.367	PA14_00860	41.569	-0.388	0.452
PA14_00875	356.730	-0.256	0.438	-0.333	0.220	-1.927	0.000	PA14_00875	999.476	0.079	0.767
PA14_00890	58.576	-0.204	0.719	-0.110	0.815	-2.847	0.000	PA14_00890	260.710	-0.278	0.342
PA14_00900	30.040	-0.378	0.578	-0.399	0.467	-0.959	0.198	PA14_00900	145.195	-0.506	0.104
PA14_00910	359.737	-0.320	0.404	0.016	0.967	-1.351	0.001	PA14_00910	905.051	0.267	0.294
PA14_00925	52.897	-0.761	0.116	-1.218	0.004	-2.392	0.000	PA14_00925	164.547	0.811	0.008
PA14_00940	84.467	-0.174	0.749	-0.554	0.158	-1.309	0.014	PA14_00940	335.267	1.391	0.000
PA14_00960	44.673	-0.543	0.408	-1.450	0.006	-2.186	0.003	PA14_00960	253.480	0.873	0.001
PA14_00970	88.378	-0.605	0.143	-1.645	0.000	-1.378	0.005	PA14_00970	485.714	-0.408	0.108
PA14_00980	726.421	-0.214	0.490	-0.376	0.128	0.076	0.847	PA14_00980	2197.924	-0.139	0.553
PA14_00990	299.382	-0.575	0.077	0.101	0.764	0.150	0.740	PA14_00990	712.112	0.710	0.079
PA14_01010	813.145	-0.589	0.030	-0.756	0.002	-1.045	0.001	PA14_01010	1822.817	0.675	0.002
PA14_01020	1521.063	-0.567	0.034	-0.800	0.001	-1.317	0.000	PA14_01020	3771.418	0.446	0.045
PA14_01030	320.115	-0.973	0.008	-1.087	0.001	-2.063	0.000	PA14_01030	1544.382	0.103	0.686
PA14_01040	48.762	-0.359	0.592	0.929	0.046	-0.364	0.642	PA14_01040	169.331	0.610	0.056
PA14_01060	121.792	0.456	0.483	0.710	0.172	0.388	0.601	PA14_01060	235.685	0.892	0.001
PA14_01070	59.562	-0.394	0.479	-0.694	0.114	-1.498	0.015	PA14_01070	193.244	0.820	0.005
PA14_01080	139.846	0.333	0.518	-0.164	0.723	-0.909	0.099	PA14_01080	380.625	-0.137	0.604
PA14_01100	380.627	-0.410	0.225	-0.793	0.005	-1.319	0.000	PA14_01100	1498.936	-0.334	0.146
PA14_01110	142.070	-0.793	0.029	-0.860	0.008	-0.749	0.097	PA14_01110	1008.732	-0.334	0.157
PA14_01120	38.694	-0.275	0.692	0.192	0.728	-1.606	0.029	PA14_01120	140.623	0.130	0.794
PA14_01130	37.476	0.131	0.861	-0.086	0.885	-0.429	0.575	PA14_01130	763.403	-0.427	0.189
PA14_01140	532.546	-0.355	0.423	0.128	0.756	-0.796	0.082	PA14_01140	1807.553	-0.147	0.537
PA14_01150	456.953	0.767	0.019	1.066	0.000	-0.318	0.482	PA14_01150	949.398	-0.171	0.508
PA14_01160	750.517	-0.622	0.024	-0.401	0.124	0.686	0.031	PA14_01160	3525.211	-1.123	0.000
PA14_01170	14.934	-0.316	0.740	0.006	0.994	-1.142	0.230	PA14_01170	92.160	-0.900	0.015
PA14_01180	15.240	-0.330	0.749	-1.038	0.174	0.464	0.650	PA14_01180	114.680	-0.840	0.012
PA14_01190	73.419	-0.698	0.143	-0.390	0.368	0.268	0.660	PA14_01190	245.753	-0.437	0.123
PA14_01200	590.054	-0.147	0.719	0.121	0.717	0.107	0.819	PA14_01200	1728.547	-0.720	0.001
PA14_01220	301.562	-0.128	0.821	0.243	0.564	-0.108	0.860	PA14_01220	1030.733	-0.698	0.002
PA14_01230	38.919	0.304	0.682	0.199	0.746	0.354	0.659	PA14_01230	163.641	-0.032	0.925
PA14_01240	966.478	-0.253	0.539	0.200	0.570	0.026	0.962	PA14_01240	2702.001	-1.091	0.047
PA14_01250	151.168	-0.644	0.083	0.497	0.122	0.220	0.656	PA14_01250	380.089	-0.278	0.313
PA14_01270	175.295	0.127	0.825	1.198	0.001	1.270	0.005	PA14_01270	403.268	-0.695	0.006
PA14_01290	862.622	0.035	0.936	-0.155	0.613	4.071	0.000	PA14_01290	4344.850	-2.612	0.000
PA14_01300	148.171	-0.928	0.052	-1.490	0.000	2.678	0.000	PA14_01300	1140.049	-2.848	0.000
PA14_01310	136.018	-0.305	0.576	-0.403	0.351	3.792	0.000	PA14_01310	760.508	-2.480	0.000
PA14_01320	116.750	-0.734	0.090	-0.779	0.040	3.063	0.000	PA14_01320	624.035	-2.474	0.000
PA14_01330	481.147	-2.223	0.000	-3.502	0.000	-0.162	0.700	PA14_01330	2644.107	-3.484	0.000
PA14_01340	27.724	-0.251	0.772	0.097	0.890	2.782	0.000	PA14_01340	350.988	-2.763	0.000

PA14_01350	55.561	0.106	0.899	-0.336	0.573	3.657	0.000	PA14_01350	431.278	-3.195	0.000
PA14_01360	44.958	0.293	0.725	0.034	0.963	4.207	0.000	PA14_01360	136.227	-2.307	0.000
PA14_01380	96.838	-0.914	0.037	-1.096	0.005	1.957	0.000	PA14_01380	454.207	-1.387	0.000
PA14_01390	854.414	-1.467	0.000	-2.046	0.000	0.217	0.592	PA14_01390	1482.363	-0.572	0.011
PA14_01400	340.977	0.395	0.330	0.351	0.322	-1.956	0.000	PA14_01400	459.804	1.474	0.000
PA14_01410	265.113	0.186	0.660	0.324	0.322	-0.844	0.041	PA14_01410	646.109	-0.700	0.003
PA14_01430	206.830	0.388	0.264	-0.017	0.963	-0.355	0.407	PA14_01430	328.762	0.204	0.507
PA14_01440	145.353	0.306	0.576	-0.277	0.552	-1.727	0.002	PA14_01440	220.661	-0.095	0.784
PA14_01460	337.718	0.551	0.291	1.305	0.002	-0.597	0.326	PA14_01460	652.537	0.965	0.010
PA14_01470	160.748	0.053	0.917	-0.070	0.855	-1.045	0.017	PA14_01470	1023.284	1.079	0.000
PA14_01480	65.293	0.205	0.707	-0.026	0.956	-0.835	0.152	PA14_01480	645.959	1.763	0.000
PA14_01490	6321.180	-4.169	0.000	-6.613	0.000	-0.563	0.071	PA14_01490	46443.974	-4.279	0.000
PA14_01500	201.792	1.299	0.000	2.272	0.000	-1.351	0.007	PA14_01500	406.880	2.104	0.000
PA14_01510	278.263	2.024	0.000	2.795	0.000	0.636	0.127	PA14_01510	427.219	1.649	0.000
PA14_01520	535.653	2.081	0.000	3.013	0.000	0.568	0.283	PA14_01520	570.335	1.013	0.000
PA14_01540	91.261	-0.890	0.069	-0.473	0.301	-0.928	0.113	PA14_01540	305.624	0.400	0.143
PA14_01550	221.681	-0.141	0.759	-0.049	0.900	-1.329	0.002	PA14_01550	429.363	0.343	0.431
PA14_01560	4850.732	-0.238	0.593	0.880	0.008	-0.727	0.094	PA14_01560	8406.012	2.269	0.000
PA14_01580	111.189	0.044	0.937	-1.983	0.000	-2.572	0.000	PA14_01580	218.593	-1.003	0.000
PA14_01600	1895.285	0.444	0.139	-1.012	0.000	-1.384	0.000	PA14_01600	8823.059	-2.153	0.000
PA14_01610	477.235	-0.059	0.902	-1.329	0.000	-1.427	0.000	PA14_01610	3712.452	-2.749	0.000
PA14_01620	484.832	-0.075	0.878	-1.718	0.000	-2.255	0.000	PA14_01620	4437.358	-2.773	0.000
PA14_01640	215.476	0.758	0.029	2.569	0.000	0.992	0.013	PA14_01640	353.756	1.599	0.000
PA14_01660	195.544	-0.442	0.210	-0.042	0.908	0.443	0.270	PA14_01660	1840.757	-1.746	0.000
PA14_01670	23.871	-0.127	0.896	0.685	0.267	-0.250	0.799	PA14_01670	65.077	0.663	0.124
PA14_01680	13.783	0.743	0.427	2.203	0.001	0.292	0.804	PA14_01680	32.753	0.201	0.792
PA14_01690	19.980	-0.152	0.874	0.806	0.181	0.019	0.985	PA14_01690	54.299	0.846	0.055
PA14_01710	16433.506	-0.618	0.011	-1.012	0.000	-0.944	0.001	PA14_01710	130185.854	-4.186	0.000
PA14_01720	652.866	-0.366	0.235	0.585	0.023	0.047	0.913	PA14_01720	7960.955	-4.399	0.000
PA14_01730	6100.045	0.026	0.946	-0.927	0.000	-2.616	0.000	PA14_01730	16258.941	1.286	0.000
PA14_01750	69.542	0.391	0.470	1.103	0.006	1.376	0.006	PA14_01750	158.118	-0.162	0.625
PA14_01760	626.407	-1.220	0.000	-2.099	0.000	-2.235	0.000	PA14_01760	1025.081	-0.405	0.096
PA14_01770	72.182	-0.720	0.104	-0.879	0.023	-2.022	0.000	PA14_01770	210.667	-0.084	0.834
PA14_01780	34.829	-1.773	0.001	-2.687	0.000	-2.057	0.002	PA14_01780	240.786	-2.384	0.000
PA14_01790	529.346	0.893	0.457	2.338	0.013	0.911	0.454	PA14_01790	391.946	-0.002	0.979
PA14_01800	163.917	0.578	0.284	0.897	0.046	-0.080	0.915	PA14_01800	339.285	-0.430	0.186
PA14_01810	65.630	-0.710	0.097	-1.205	0.001	-1.701	0.001	PA14_01810	244.215	-0.921	0.001
PA14_01830	590.703	0.691	0.011	0.860	0.001	0.664	0.043	PA14_01830	1105.146	-0.192	0.447
PA14_01840	18.658	-0.423	0.634	0.553	0.406	-0.095	0.928	PA14_01840	29.951	1.368	0.016
PA14_01860	19.391	-0.284	0.764	1.001	0.109	-0.609	0.537	PA14_01860	31.289	0.531	0.381
PA14_01870	47.681	0.367	0.521	0.850	0.045	0.406	0.532	PA14_01870	92.826	-0.293	0.453
PA14_01890	45.881	0.410	0.488	0.678	0.139	-0.060	0.941	PA14_01890	89.143	0.269	0.546
PA14_01900	93.776	1.505	0.000	0.988	0.008	0.431	0.444	PA14_01900	57.558	0.265	0.624
PA14_01910	29.483	0.807	0.175	0.154	0.804	-1.008	0.214	PA14_01910	32.646	-2.000	0.000
PA14_01930	315.795	0.107	0.824	0.434	0.191	-1.597	0.000	PA14_01930	418.202	0.926	0.000
PA14_01940	287.654	-1.015	0.001	-1.125	0.000	-1.547	0.000	PA14_01940	669.485	-1.342	0.000
PA14_01960	275.238	-1.080	0.000	-1.276	0.000	-1.839	0.000	PA14_01960	817.666	-1.497	0.000
PA14_01970	1379.384	-0.866	0.002	-1.148	0.000	-1.322	0.000	PA14_01970	3185.458	-1.270	0.000
PA14_01980	367.047	-0.864	0.002	-1.117	0.000	-2.028	0.000	PA14_01980	1620.791	-1.407	0.000
PA14_01990	130.958	1.133	0.009	2.791	0.000	-0.610	0.340	PA14_01990	118.139	-0.818	0.017
PA14_02010	996.811	2.163	0.000	3.614	0.000	-0.526	0.242	PA14_02010	551.027	-0.876	0.011
PA14_02020	2220.036	1.384	0.000	1.814	0.000	-2.252	0.000	PA14_02020	498.884	0.010	0.969
PA14_02030	49.483	0.404	0.477	0.543	0.231	-0.283	0.697	PA14_02030	134.479	0.751	0.206
PA14_02050	200.440	1.754	0.000	2.346	0.000	-0.577	0.230	PA14_02050	913.410	-1.826	0.000
PA14_02060	336.060	2.102	0.000	1.367	0.000	0.158	0.747	PA14_02060	255.327	0.821	0.002
PA14_02070	13.235	0.556	0.545	0.658	0.373	0.649	0.508	PA14_02070	18.863	0.248	0.758
PA14_02090	448.501	0.540	0.055	0.344	0.197	-1.473	0.000	PA14_02090	1417.042	0.505	0.035
PA14_02100	50.489	0.492	0.371	0.338	0.486	-2.041	0.005	PA14_02100	203.550	-0.318	0.277
PA14_02110	41.102	-0.601	0.352	-0.303	0.598	-2.465	0.001	PA14_02110	24.378	0.934	0.150

PA14_02130	28.962	-0.341	0.666	-0.863	0.146	-2.351	0.005	PA14_02130	48.251	0.681	0.165
PA14_02140	605.257	0.454	0.424	0.885	0.053	-0.831	0.164	PA14_02140	259.067	0.172	0.610
PA14_02150	250.320	0.030	0.954	0.790	0.009	0.131	0.793	PA14_02150	224.916	0.321	0.299
PA14_02180	10.572	-1.164	0.175	-2.923	0.000	-0.859	0.393	PA14_02180	77.681	-2.996	0.000
PA14_02190	52.638	-1.498	0.012	-3.282	0.000	1.064	0.111	PA14_02190	253.806	-2.692	0.000
PA14_02200	100.946	-1.989	0.000	-4.252	0.000	0.713	0.161	PA14_02200	757.136	-3.153	0.000
PA14_02220	947.148	-2.177	0.000	-4.456	0.000	0.648	0.048	PA14_02220	3578.260	-2.660	0.000
PA14_02230	183.323	-2.027	0.000	-4.525	0.000	0.638	0.106	PA14_02230	856.966	-2.488	0.000
PA14_02250	703.440	-1.673	0.000	-3.603	0.000	1.274	0.000	PA14_02250	2067.866	-2.196	0.000
PA14_02260	4924.944	-1.931	0.000	-3.767	0.000	2.225	0.000	PA14_02260	9960.547	-3.370	0.000
PA14_02270	552.572	-1.443	0.000	-2.533	0.000	-0.533	0.257	PA14_02270	2536.631	-1.968	0.000
PA14_02290	75.751	0.300	0.556	0.147	0.742	-0.110	0.868	PA14_02290	267.132	1.001	0.000
PA14_02300	22.136	-0.042	0.967	0.517	0.397	0.088	0.928	PA14_02300	54.891	0.523	0.258
PA14_02310	55.538	0.098	0.885	0.263	0.583	-0.473	0.477	PA14_02310	76.713	1.261	0.001
PA14_02330	44.073	0.187	0.807	0.750	0.140	0.384	0.614	PA14_02330	57.206	1.183	0.006
PA14_02340	43.661	0.998	0.082	1.161	0.021	0.239	0.776	PA14_02340	63.791	0.529	0.230
PA14_02360	46.768	0.527	0.390	0.897	0.063	0.227	0.773	PA14_02360	69.301	0.318	0.470
PA14_02370	11.332	-0.164	0.887	-0.075	0.933	-0.365	0.748	PA14_02370	26.247	0.759	0.251
PA14_02380	28.012	0.221	0.788	0.997	0.064	0.361	0.671	PA14_02380	47.260	0.911	0.052
PA14_02390	68.673	-0.799	0.086	-0.607	0.143	-1.158	0.043	PA14_02390	99.757	-0.296	0.449
PA14_02410	37.166	-0.302	0.650	0.252	0.631	0.727	0.245	PA14_02410	37.805	1.020	0.057
PA14_02420	16.897	-1.701	0.015	-1.397	0.025	-1.495	0.078	PA14_02420	27.625	-1.026	0.325
PA14_02435	9.882	0.172	0.900	-0.060	0.956	-0.167	0.901	PA14_02435	20.093	-0.634	0.413
PA14_02450	469.119	-1.608	0.000	-2.926	0.000	0.190	0.663	PA14_02450	2589.414	-1.328	0.000
PA14_02460	96.949	-1.867	0.000	-3.289	0.000	-0.828	0.218	PA14_02460	390.617	0.312	0.574
PA14_02470	141.067	-2.237	0.000	-4.156	0.000	-1.184	0.024	PA14_02470	420.807	0.051	0.870
PA14_02490	10.606	-1.982	0.018	-1.466	0.049	-1.343	0.172	PA14_02490	23.297	-0.870	0.216
PA14_02500	19.267	-0.970	0.171	-0.532	0.401	-0.142	0.882	PA14_02500	56.375	0.042	0.916
PA14_02510	43.249	-0.876	0.161	-0.192	0.751	-0.013	0.988	PA14_02510	162.392	-0.374	0.661
PA14_02520	2859.409	-0.632	0.012	-1.634	0.000	-3.067	0.000	PA14_02520	21526.805	-1.822	0.000
PA14_02530	1180.000	0.351	0.534	0.048	0.929	-1.841	0.001	PA14_02530	2075.632	0.263	0.560
PA14_02550	622.223	-2.563	0.000	-2.697	0.000	0.524	0.138	PA14_02550	466.333	-0.156	0.582
PA14_02560	116.691	-2.113	0.000	-2.597	0.000	1.508	0.000	PA14_02560	89.073	1.330	0.059
PA14_02570	4.655	-1.133	0.391	-1.311	0.239	1.603	0.172	PA14_02570	13.853	2.392	0.007
PA14_02580	31.324	-1.957	0.002	-2.902	0.000	1.888	0.002	PA14_02580	37.946	1.122	0.031
PA14_02590	23.376	-1.692	0.016	-1.405	0.024	2.233	0.000	PA14_02590	28.339	0.936	0.121
PA14_02610	44.921	-1.125	0.088	-0.724	0.215	3.067	0.000	PA14_02610	33.024	0.825	0.156
PA14_02620	86.324	-1.598	0.000	-1.958	0.000	2.432	0.000	PA14_02620	92.880	0.382	0.365
PA14_02630	19.445	0.510	0.585	0.886	0.215	2.165	0.006	PA14_02630	49.555	-0.519	0.294
PA14_02640	46.812	0.026	0.974	0.550	0.232	1.804	0.000	PA14_02640	94.425	0.913	0.010
PA14_02650	97.919	0.025	0.968	-0.161	0.700	-0.001	0.999	PA14_02650	244.474	0.730	0.008
PA14_02660	92.368	0.090	0.869	-0.056	0.897	-0.248	0.660	PA14_02660	292.711	-0.389	0.164
PA14_02680	35.180	0.328	0.614	0.255	0.635	0.027	0.974	PA14_02680	61.391	0.279	0.652
PA14_02690	27.924	0.084	0.928	0.177	0.792	-0.232	0.804	PA14_02690	38.183	-0.351	0.531
PA14_02700	45.948	0.602	0.256	-0.048	0.931	0.322	0.638	PA14_02700	69.345	0.219	0.601
PA14_02720	45.135	1.158	0.022	1.095	0.018	0.592	0.387	PA14_02720	84.149	0.333	0.410
PA14_02730	295.888	0.737	0.038	1.280	0.000	-0.242	0.633	PA14_02730	377.897	0.930	0.000
PA14_02740	87.303	1.179	0.002	1.315	0.000	1.322	0.004	PA14_02740	122.372	0.004	1.000
PA14_02750	158.531	-0.697	0.109	-0.755	0.050	-1.759	0.000	PA14_02750	488.945	0.624	0.011
PA14_02760	106.875	0.454	0.285	0.390	0.291	0.943	0.032	PA14_02760	263.101	0.699	0.011
PA14_02770	78.221	-0.271	0.618	0.034	0.945	1.164	0.014	PA14_02770	186.239	0.495	0.100
PA14_02790	88.301	0.197	0.738	-0.068	0.894	0.341	0.577	PA14_02790	306.782	0.454	0.092
PA14_02810	169.941	0.010	0.984	0.050	0.899	-0.258	0.597	PA14_02810	587.475	-0.738	0.002
PA14_02830	104.800	-0.181	0.707	-0.049	0.907	-0.492	0.326	PA14_02830	415.120	0.165	0.539
PA14_02840	149.519	-0.461	0.198	-0.796	0.008	-1.139	0.005	PA14_02840	638.528	0.407	0.093
PA14_02850	152.572	-0.206	0.646	-0.575	0.085	-1.367	0.002	PA14_02850	595.293	-0.288	0.254
PA14_02870	113.596	0.512	0.225	1.050	0.002	-0.569	0.292	PA14_02870	227.755	0.805	0.004
PA14_02890	199.398	3.070	0.000	1.567	0.000	1.245	0.002	PA14_02890	182.665	0.476	0.111
PA14_02900	14.531	-0.363	0.697	-1.048	0.134	0.587	0.516	PA14_02900	24.604	1.123	0.088

PA14_02910	58.034	-0.115	0.846	-0.258	0.551	-2.491	0.000	PA14_02910	117.659	0.104	0.806
PA14_02930	30.780	0.910	0.166	0.849	0.144	-0.733	0.412	PA14_02930	56.234	1.530	0.000
PA14_02960	13.605	0.162	0.892	0.722	0.362	-0.312	0.793	PA14_02960	37.292	0.704	0.201
PA14_02970	80.682	1.293	0.002	1.364	0.000	-1.144	0.075	PA14_02970	186.787	2.274	0.000
PA14_02980	42.273	1.584	0.001	0.108	0.857	1.054	0.092	PA14_02980	42.660	0.759	0.130
PA14_02990	68.263	2.177	0.000	0.197	0.699	-0.415	0.560	PA14_02990	68.946	0.570	0.184
PA14_03000	80.250	3.477	0.000	1.419	0.002	-0.458	0.581	PA14_03000	216.412	2.214	0.000
PA14_03010	137.479	-0.481	0.214	-0.337	0.329	-1.657	0.000	PA14_03010	465.200	0.247	0.358
PA14_03020	13.183	0.922	0.290	1.167	0.107	0.593	0.573	PA14_03020	27.209	0.407	0.536
PA14_03030	17.946	2.507	0.000	1.088	0.135	0.564	0.598	PA14_03030	41.126	0.551	0.285
PA14_03040	93.158	0.492	0.281	1.028	0.005	-0.714	0.206	PA14_03040	178.619	0.779	0.009
PA14_03050	63.924	0.921	0.074	0.883	0.057	0.933	0.122	PA14_03050	68.355	0.521	0.207
PA14_03070	86.005	-0.159	0.774	0.461	0.235	-2.018	0.001	PA14_03070	171.778	1.925	0.000
PA14_03080	69.608	-0.451	0.433	-0.520	0.274	-0.475	0.472	PA14_03080	931.438	-2.611	0.000
PA14_03090	271.597	-1.306	0.000	-1.498	0.000	-0.838	0.022	PA14_03090	7014.114	-4.253	0.000
PA14_03100	19.187	0.088	0.935	0.400	0.573	0.488	0.601	PA14_03100	20.177	0.839	0.254
PA14_03110	86.430	0.570	0.250	1.816	0.000	1.615	0.001	PA14_03110	122.806	1.455	0.000
PA14_03120	69.127	0.352	0.489	1.091	0.003	-0.792	0.181	PA14_03120	192.890	0.558	0.070
PA14_03130	1716.500	1.771	0.000	2.305	0.000	-2.007	0.000	PA14_03130	2644.296	1.478	0.000
PA14_03150	343.709	0.910	0.003	1.267	0.000	0.220	0.626	PA14_03150	841.286	0.873	0.006
PA14_03160	1503.407	-0.871	0.006	-1.007	0.001	0.742	0.055	PA14_03160	6983.307	-2.009	0.000
PA14_03163	13735.595	-0.260	0.417	-0.356	0.183	-1.280	0.000	PA14_03163	57000.016	-1.214	0.000
PA14_03166	401.202	0.151	0.729	0.332	0.309	-0.841	0.039	PA14_03166	5598.026	-1.125	0.000
PA14_03170	329.876	0.471	0.188	0.945	0.002	-0.221	0.645	PA14_03170	896.391	-0.915	0.000
PA14_03180	441.558	-0.051	0.925	-0.370	0.299	-1.219	0.004	PA14_03180	1450.486	-0.419	0.074
PA14_03190	834.807	-0.107	0.786	-0.501	0.069	-0.723	0.039	PA14_03190	3408.109	-0.741	0.001
PA14_03200	1034.153	0.084	0.859	-0.229	0.505	-0.759	0.058	PA14_03200	3524.250	-1.033	0.000
PA14_03210	43.117	-0.561	0.304	-1.192	0.008	-0.580	0.371	PA14_03210	249.044	-0.541	0.051
PA14_03220	339.862	-0.246	0.587	-0.271	0.468	-0.039	0.944	PA14_03220	1677.916	-0.892	0.000
PA14_03240	4.768	-0.285	0.848	-0.218	0.852	0.270	0.849	PA14_03240	684.195	-1.752	0.000
PA14_03250	790.672	0.043	0.909	-0.476	0.044	-0.558	0.070	PA14_03250	2397.548	0.043	0.870
PA14_03265	850.708	0.033	0.948	-0.432	0.179	-0.705	0.079	PA14_03265	2237.666	-0.037	0.918
PA14_03270	2157.793	-0.197	0.538	-0.469	0.060	-0.988	0.001	PA14_03270	6281.064	-0.217	0.347
PA14_03285	507.281	-0.041	0.930	-0.384	0.206	-0.671	0.080	PA14_03285	1664.565	-0.647	0.004
PA14_03290	317.699	0.345	0.314	0.357	0.227	-0.091	0.847	PA14_03290	2811.336	0.158	0.478
PA14_03300	50.778	1.087	0.029	1.432	0.001	0.145	0.857	PA14_03300	1135.676	0.006	0.983
PA14_03310	266.822	0.186	0.621	0.315	0.280	-0.176	0.689	PA14_03310	830.820	0.611	0.007
PA14_03320	641.164	0.178	0.609	0.567	0.027	-0.131	0.748	PA14_03320	1952.239	0.489	0.024
PA14_03330	282.995	0.188	0.681	0.046	0.910	-0.937	0.032	PA14_03330	857.106	1.214	0.001
PA14_03340	506.746	0.542	0.071	0.459	0.096	-0.564	0.127	PA14_03340	1372.372	1.170	0.000
PA14_03350	1706.150	-0.046	0.931	-0.307	0.389	-1.685	0.000	PA14_03350	4701.472	-0.825	0.000
PA14_03360	551.984	-0.104	0.802	-0.890	0.001	-1.898	0.000	PA14_03360	1555.533	-0.353	0.132
PA14_03370	412.031	0.006	0.987	-1.135	0.000	-2.385	0.000	PA14_03370	1019.357	-0.257	0.317
PA14_03380	1328.901	-0.090	0.805	-0.636	0.010	-0.882	0.004	PA14_03380	3983.229	-0.021	0.967
PA14_03390	132.235	0.643	0.138	1.355	0.000	0.943	0.050	PA14_03390	644.903	-0.542	0.023
PA14_03400	117.201	0.948	0.049	2.416	0.000	2.061	0.000	PA14_03400	536.843	-0.323	0.205
PA14_03410	40.716	1.073	0.112	3.132	0.000	2.798	0.000	#N/A	#N/A	#N/A	#N/A
PA14_03420	42.420	0.633	0.268	1.094	0.016	1.062	0.078	PA14_03420	171.323	-0.271	0.775
PA14_03430	1581.310	0.577	0.018	0.020	0.946	-0.298	0.355	PA14_03430	3091.308	0.806	0.000
PA14_03450	1658.035	0.253	0.470	-0.291	0.320	-1.527	0.000	PA14_03450	2375.862	0.737	0.011
PA14_03470	214.119	-0.136	0.736	0.167	0.595	-1.065	0.005	PA14_03470	325.823	0.240	0.405
PA14_03480	35.967	-0.040	0.965	-0.323	0.579	-0.529	0.499	PA14_03480	132.280	0.630	0.052
PA14_03490	107.608	-1.375	0.000	-2.400	0.000	-1.217	0.006	PA14_03490	474.116	-1.306	0.000
PA14_03510	155.210	-1.312	0.000	-2.270	0.000	-0.763	0.100	PA14_03510	773.670	-0.936	0.000
PA14_03520	86.735	-1.378	0.000	-1.793	0.000	-0.524	0.293	PA14_03520	355.819	-1.443	0.000
PA14_03530	17.513	-0.305	0.744	-0.680	0.329	-0.325	0.747	PA14_03530	54.147	0.646	0.171
PA14_03550	7.254	-0.134	0.920	-1.176	0.189	-1.217	0.282	PA14_03550	13.952	0.835	0.355
PA14_03560	34.966	0.305	0.695	0.486	0.407	1.779	0.005	PA14_03560	70.369	0.599	0.161
PA14_03580	78.776	-0.009	0.988	-1.446	0.000	-0.647	0.240	PA14_03580	405.044	-0.688	0.006

PA14_03590	38.356	-1.362	0.007	-1.937	0.000	-1.761	0.005	PA14_03590	1441.300	-3.449	0.000
PA14_03610	57.642	-0.002	0.997	-0.205	0.642	-1.421	0.013	PA14_03610	139.018	0.379	0.293
PA14_03620	25.973	-0.418	0.598	-0.489	0.442	0.036	0.970	PA14_03620	58.259	0.396	0.561
PA14_03630	20.760	-0.689	0.403	-0.005	0.995	-1.732	0.057	PA14_03630	69.101	-0.546	0.245
PA14_03650	172.752	-2.086	0.000	-1.870	0.000	-3.949	0.000	PA14_03650	171.366	-0.106	0.764
PA14_03670	223.487	-1.342	0.000	-1.291	0.000	-2.476	0.000	PA14_03670	181.718	0.284	0.357
PA14_03680	201.165	-1.832	0.000	-1.841	0.000	-2.195	0.000	PA14_03680	133.099	0.401	0.346
PA14_03700	1456.548	-1.816	0.000	-0.996	0.000	-1.218	0.000	PA14_03700	573.990	-0.386	0.196
PA14_03710	1646.362	-2.487	0.000	-2.395	0.000	-2.541	0.000	PA14_03710	661.979	-1.019	0.000
PA14_03720	784.707	0.658	0.012	1.319	0.000	0.757	0.015	PA14_03720	963.919	1.801	0.000
PA14_03730	4006.698	-0.505	0.061	0.228	0.388	0.512	0.108	PA14_03730	11693.345	-0.063	0.811
PA14_03760	53.368	-0.299	0.599	-0.178	0.711	-0.142	0.840	PA14_03760	77.496	-0.366	0.342
PA14_03770	52.527	0.032	0.967	0.390	0.417	-0.100	0.896	PA14_03770	70.374	0.036	0.931
PA14_03780	133.308	-0.590	0.111	-1.194	0.000	-0.710	0.108	PA14_03780	678.405	-1.151	0.000
PA14_03790	216.193	-1.240	0.000	-1.141	0.000	1.041	0.005	PA14_03790	887.242	-1.289	0.000
PA14_03800	3509.182	0.034	0.934	0.090	0.759	-0.117	0.764	PA14_03800	4642.698	0.434	0.063
PA14_03810	797.362	0.970	0.001	1.348	0.000	0.012	0.978	PA14_03810	691.468	0.278	0.257
PA14_03830	913.732	1.171	0.000	1.360	0.000	0.368	0.432	PA14_03830	1105.652	-0.158	0.684
PA14_03840	113.470	0.008	0.989	-0.276	0.486	-0.980	0.051	PA14_03840	277.244	0.678	0.012
PA14_03855	454.208	-0.610	0.033	-1.699	0.000	-0.704	0.038	PA14_03855	1104.659	1.230	0.000
PA14_03860	3533.192	1.390	0.000	0.469	0.089	1.352	0.000	PA14_03860	8271.546	1.256	0.000
PA14_03870	3072.260	2.400	0.000	0.866	0.014	1.223	0.005	PA14_03870	6391.474	0.295	0.378
PA14_03880	4325.542	1.553	0.000	0.112	0.667	1.055	0.000	PA14_03880	6804.742	2.143	0.000
PA14_03900	3998.335	0.576	0.034	-0.905	0.000	-0.017	0.968	PA14_03900	6112.345	1.362	0.000
PA14_03920	5319.892	0.017	0.966	-1.458	0.000	-0.884	0.002	PA14_03920	9738.487	0.301	0.205
PA14_03930	2067.905	0.790	0.002	0.078	0.795	0.095	0.814	PA14_03930	3892.944	0.561	0.008
PA14_03940	1388.700	0.514	0.047	-0.335	0.174	-0.722	0.017	PA14_03940	2319.154	1.062	0.006
PA14_03950	824.096	0.065	0.870	-0.720	0.004	-1.259	0.000	PA14_03950	1318.834	0.793	0.030
PA14_03960	842.428	-0.048	0.925	-0.434	0.190	-1.117	0.005	PA14_03960	869.738	0.283	0.271
PA14_03980	312.445	0.195	0.599	-0.159	0.612	-0.548	0.151	PA14_03980	763.230	0.326	0.170
PA14_04000	88.751	-0.055	0.927	-0.372	0.349	-1.146	0.029	PA14_04000	189.922	1.239	0.000
PA14_04010	2895.576	-1.710	0.000	-2.746	0.000	-1.413	0.000	PA14_04010	4965.327	-0.846	0.000
PA14_04020	69.758	-0.532	0.276	-0.293	0.502	-0.508	0.384	PA14_04020	122.450	1.196	0.000
PA14_04030	148.836	0.679	0.056	0.705	0.026	-0.012	0.983	PA14_04030	382.142	1.095	0.000
PA14_04040	198.158	-0.036	0.939	0.350	0.242	-0.114	0.812	PA14_04040	615.387	-0.016	0.940
PA14_04050	281.555	1.735	0.000	2.098	0.000	2.060	0.000	PA14_04050	803.738	0.758	0.001
PA14_04060	16.941	1.055	0.196	2.185	0.001	1.087	0.238	PA14_04060	32.806	0.914	0.109
PA14_04070	239.322	-0.339	0.342	-0.306	0.318	-0.412	0.314	PA14_04070	939.583	-0.141	0.553
PA14_04080	80.861	-0.315	0.505	-0.388	0.311	-0.531	0.310	PA14_04080	181.041	-0.155	0.671
PA14_04090	380.034	-1.133	0.000	-1.400	0.000	-0.800	0.038	PA14_04090	1256.433	-1.368	0.000
PA14_04100	2473.296	0.191	0.524	-0.086	0.751	-0.361	0.255	PA14_04100	4005.980	-0.016	1.000
PA14_04110	1667.353	0.273	0.475	0.445	0.149	-0.046	0.926	PA14_04110	2652.470	1.737	0.000
PA14_04140	1628.922	0.864	0.000	0.983	0.000	0.570	0.070	PA14_04140	2995.840	1.169	0.000
PA14_04150	474.280	0.069	0.866	-0.255	0.375	-0.820	0.018	PA14_04150	1331.139	0.471	0.036
PA14_04160	528.159	0.582	0.032	1.113	0.000	0.557	0.088	PA14_04160	1264.004	1.321	0.000
PA14_04180	127.562	-0.206	0.772	0.624	0.205	-0.046	0.955	PA14_04180	105.207	0.767	0.030
PA14_04190	106.848	3.602	0.000	-0.963	0.071	0.447	0.537	PA14_04190	74.517	-0.658	0.102
PA14_04210	60.671	2.433	0.000	-0.876	0.101	-0.399	0.616	PA14_04210	23.578	0.727	0.290
PA14_04220	158.175	2.069	0.000	-0.230	0.599	-0.241	0.696	PA14_04220	207.653	0.940	0.001
PA14_04230	93.274	1.777	0.000	-0.021	0.971	0.986	0.094	PA14_04230	126.721	1.590	0.000
PA14_04240	67.432	2.717	0.000	2.578	0.000	1.302	0.067	PA14_04240	89.662	0.128	0.762
PA14_04250	73.041	1.405	0.000	0.042	0.934	1.098	0.028	PA14_04250	110.202	0.500	0.143
PA14_04270	93.189	-0.650	0.165	-0.516	0.214	0.727	0.155	PA14_04270	376.720	-1.280	0.000
PA14_04290	308.736	0.822	0.011	0.732	0.015	0.492	0.236	PA14_04290	503.744	0.066	0.820
PA14_04300	1268.980	-0.794	0.004	-0.604	0.022	-0.536	0.123	PA14_04300	6104.620	-1.525	0.000
PA14_04310	739.128	0.336	0.323	0.924	0.001	0.927	0.007	PA14_04310	1237.360	1.405	0.000
PA14_04320	1772.965	0.752	0.011	1.165	0.000	-0.134	0.760	PA14_04320	2767.596	1.221	0.000
PA14_04330	292.529	-0.689	0.036	-0.873	0.003	-0.160	0.732	PA14_04330	1361.809	-1.079	0.000
PA14_04340	168.247	-0.132	0.772	0.582	0.058	0.901	0.019	PA14_04340	411.589	-0.263	0.321

PA14_04350	58.154	-0.069	0.915	-0.159	0.729	-0.456	0.451	PA14_04350	194.593	0.160	0.621
PA14_04370	76.467	0.585	0.225	1.010	0.010	-0.670	0.284	PA14_04370	137.105	0.784	0.014
PA14_04380	226.898	-0.022	0.967	0.287	0.396	-1.206	0.004	PA14_04380	538.089	0.513	0.036
PA14_04390	3292.005	0.253	0.418	0.438	0.084	-0.694	0.029	PA14_04390	10658.100	-0.197	0.487
PA14_04410	6069.734	0.513	0.276	0.735	0.066	-0.453	0.411	PA14_04410	8609.279	0.247	0.275
PA14_04420	746.968	0.905	0.000	1.714	0.000	2.159	0.000	PA14_04420	1535.294	0.023	0.977
PA14_04430	206.177	0.722	0.024	0.783	0.007	-1.182	0.005	PA14_04430	507.131	0.282	0.341
PA14_04440	467.199	0.805	0.152	2.198	0.000	1.365	0.024	PA14_04440	590.941	0.448	0.179
PA14_04460	187.718	-0.194	0.670	0.115	0.764	-1.391	0.001	PA14_04460	429.438	0.684	0.005
PA14_04480	1011.707	1.018	0.001	1.737	0.000	1.060	0.003	PA14_04480	1464.537	1.118	0.000
PA14_04490	440.015	0.573	0.118	1.061	0.001	-0.231	0.644	PA14_04490	1038.236	-0.722	0.002
PA14_04510	291.282	-0.222	0.576	-0.571	0.059	-1.431	0.000	PA14_04510	550.797	-0.529	0.031
PA14_04520	178.485	-0.339	0.344	-0.933	0.001	-0.756	0.053	PA14_04520	353.744	0.251	0.358
PA14_04530	53.115	-0.141	0.832	-0.515	0.263	-0.821	0.192	PA14_04530	134.794	0.107	0.798
PA14_04550	40.969	-0.396	0.507	-0.778	0.096	0.151	0.838	PA14_04550	128.319	-2.338	0.000
PA14_04560	60.997	0.926	0.045	1.520	0.000	-0.221	0.760	PA14_04560	121.566	0.933	0.026
PA14_04570	26.196	-0.179	0.833	0.060	0.929	-0.563	0.504	PA14_04570	62.202	0.654	0.122
PA14_04580	74.692	0.833	0.060	1.318	0.000	-0.714	0.255	PA14_04580	148.988	1.288	0.000
PA14_04590	122.299	1.180	0.001	0.919	0.006	-0.356	0.522	PA14_04590	465.048	-0.023	0.967
PA14_04610	52.994	0.059	0.935	1.169	0.005	-0.582	0.388	PA14_04610	86.089	1.756	0.000
PA14_04630	2244.871	0.359	0.185	0.187	0.469	-0.193	0.581	PA14_04630	6207.541	0.286	0.198
PA14_04640	218.572	0.573	0.119	2.011	0.000	1.671	0.000	PA14_04640	366.630	1.175	0.000
PA14_04650	519.141	-1.884	0.000	-2.748	0.000	-0.964	0.002	PA14_04650	3007.396	-3.025	0.000
PA14_04660	277.574	0.619	0.071	1.012	0.001	0.686	0.088	PA14_04660	509.478	0.188	0.538
PA14_04670	1362.948	0.883	0.000	1.228	0.000	-0.456	0.165	PA14_04670	2669.250	0.607	0.036
PA14_04680	269.293	0.921	0.006	0.781	0.013	0.469	0.291	PA14_04680	586.602	-0.280	0.317
PA14_04690	587.356	0.303	0.371	0.010	0.977	-1.340	0.000	PA14_04690	2387.095	-1.722	0.000
PA14_04700	738.642	0.546	0.053	0.766	0.002	0.133	0.742	PA14_04700	1854.262	0.910	0.000
PA14_04710	296.620	0.254	0.496	-0.849	0.003	-1.170	0.002	PA14_04710	309.064	-0.099	0.747
PA14_04730	414.346	-0.218	0.558	-0.316	0.291	-0.523	0.173	PA14_04730	838.312	-0.058	0.835
PA14_04750	631.130	0.537	0.079	1.020	0.000	-0.073	0.873	PA14_04750	1766.691	-0.091	0.752
PA14_04760	524.249	-0.334	0.343	-0.007	0.984	-1.284	0.000	PA14_04760	1102.629	1.045	0.000
PA14_04780	483.302	-0.733	0.055	0.128	0.752	-1.883	0.000	PA14_04780	808.960	0.020	0.927
PA14_04790	409.422	-0.663	0.027	-0.132	0.676	-1.997	0.000	PA14_04790	960.038	-0.260	0.291
PA14_04810	934.501	-0.899	0.001	-0.230	0.440	-1.609	0.000	PA14_04810	3619.714	-1.380	0.000
PA14_04820	480.661	0.268	0.433	0.581	0.030	-0.876	0.013	PA14_04820	1265.685	0.141	0.572
PA14_04830	106.373	-0.413	0.350	-0.450	0.226	-0.944	0.052	PA14_04830	467.147	-1.501	0.000
PA14_04840	284.446	0.208	0.585	0.397	0.178	-0.994	0.010	PA14_04840	577.987	0.118	0.694
PA14_04850	62.223	0.968	0.028	1.078	0.006	-0.654	0.321	PA14_04850	106.097	-0.122	0.789
PA14_04860	266.608	-0.334	0.312	-0.418	0.134	-1.804	0.000	PA14_04860	692.961	-1.096	0.002
PA14_04870	590.631	-0.320	0.296	-0.814	0.001	-1.137	0.000	PA14_04870	885.085	0.425	0.145
PA14_04890	710.527	-0.312	0.358	-0.371	0.196	-0.454	0.225	PA14_04890	1622.886	0.254	0.277
PA14_04900	3195.974	0.842	0.004	1.387	0.000	1.031	0.002	PA14_04900	4221.244	1.532	0.000
PA14_04910	1569.160	0.478	0.065	0.739	0.001	-0.278	0.408	PA14_04910	2283.329	1.623	0.000
PA14_04920	1392.239	0.055	0.904	0.642	0.025	0.273	0.513	PA14_04920	3021.222	-0.360	0.100
PA14_04930	4385.520	0.180	0.614	0.194	0.512	0.218	0.576	PA14_04930	19626.096	0.041	0.877
PA14_04940	299.388	0.327	0.390	0.578	0.058	0.261	0.563	PA14_04940	1045.380	0.843	0.000
PA14_04950	66.184	0.576	0.250	0.844	0.041	0.313	0.627	PA14_04950	153.748	1.414	0.000
PA14_04960	63.295	-0.156	0.816	0.396	0.397	-0.498	0.460	PA14_04960	258.112	0.878	0.001
PA14_04970	108.815	0.783	0.049	2.029	0.000	1.650	0.000	PA14_04970	211.606	1.599	0.000
PA14_04980	329.279	0.164	0.754	0.554	0.143	-0.670	0.179	PA14_04980	431.978	1.525	0.000
PA14_05000	275.563	0.911	0.003	1.809	0.000	0.795	0.035	PA14_05000	157.084	1.961	0.000
PA14_05010	118.410	0.558	0.167	2.112	0.000	0.845	0.060	PA14_05010	178.109	-0.014	0.995
PA14_05020	44.605	-0.043	0.965	0.041	0.954	0.692	0.364	PA14_05020	233.446	-1.675	0.000
PA14_05030	167.625	0.207	0.629	0.881	0.003	0.575	0.172	PA14_05030	402.638	0.192	0.506
PA14_05040	71.133	0.518	0.325	1.320	0.001	1.335	0.010	PA14_05040	137.252	2.069	0.000
PA14_05050	254.771	0.233	0.540	0.393	0.188	0.085	0.859	PA14_05050	1171.727	-0.653	0.035
PA14_05060	2412.263	1.069	0.008	1.479	0.000	1.052	0.029	PA14_05060	6631.143	-0.767	0.000
PA14_05070	555.454	0.513	0.071	0.827	0.001	-0.781	0.021	PA14_05070	1333.414	-0.458	0.270

PA14_05080	606.980	1.130	0.000	2.241	0.000	0.199	0.624
PA14_05110	392.749	-0.966	0.000	-1.048	0.000	-0.150	0.706
PA14_05120	96.395	0.631	0.125	1.031	0.003	0.088	0.889
PA14_05130	314.457	0.048	0.924	0.009	0.981	-1.136	0.005
PA14_05150	217.504	0.049	0.923	-0.571	0.077	-1.083	0.010
PA14_05160	2910.968	0.465	0.279	0.815	0.023	0.903	0.047
PA14_05180	1119.422	-0.391	0.161	-0.252	0.332	-0.138	0.713
PA14_05190	549.791	-0.728	0.036	-0.939	0.003	-0.654	0.118
PA14_05200	191.836	-1.028	0.005	-0.745	0.028	-0.387	0.427
PA14_05210	200.017	-0.144	0.756	-0.014	0.973	-1.330	0.002
PA14_05220	802.420	-1.779	0.000	-2.788	0.000	-2.745	0.000
PA14_05230	766.040	-1.486	0.000	-2.332	0.000	-2.458	0.000
PA14_05250	341.742	-0.298	0.438	-0.359	0.262	-1.229	0.002
PA14_05260	1389.446	-0.014	0.975	-0.251	0.353	-1.336	0.000
PA14_05270	192.539	-0.480	0.298	-0.861	0.024	-1.657	0.001
PA14_05280	155.740	1.093	0.001	1.413	0.000	0.845	0.045
PA14_05290	1117.584	0.732	0.016	1.054	0.000	-0.165	0.712
PA14_05300	680.935	0.129	0.746	0.409	0.154	-0.015	0.974
PA14_05310	1278.736	0.069	0.848	-0.473	0.051	-0.912	0.002
PA14_05320	3389.176	0.218	0.447	-0.551	0.014	-0.557	0.056
PA14_05330	3221.145	0.048	0.890	-0.658	0.002	-1.122	0.000
PA14_05340	489.482	0.283	0.416	0.216	0.475	-0.190	0.657
PA14_05360	7139.476	-0.141	0.668	-0.416	0.089	-1.471	0.000
PA14_05380	230.812	-0.502	0.181	-0.765	0.017	-1.234	0.003
PA14_05390	4546.982	-0.078	0.829	-0.046	0.873	-1.728	0.000
PA14_05400	338.057	-0.304	0.338	-0.514	0.047	-1.781	0.000
PA14_05410	109.793	-0.220	0.623	-0.233	0.520	-1.078	0.018
PA14_05420	85.223	0.081	0.887	0.449	0.233	-0.943	0.072
PA14_05430	26.314	0.616	0.357	1.002	0.059	-1.069	0.217
PA14_05440	381.235	0.659	0.091	0.860	0.013	-0.266	0.618
PA14_05450	109.761	0.823	0.063	1.980	0.000	0.723	0.178
PA14_05460	1111.210	0.426	0.116	0.454	0.062	-1.197	0.000
PA14_05480	302.957	-0.604	0.070	-0.944	0.001	-1.391	0.000
PA14_05500	3212.144	0.920	0.031	1.283	0.001	1.630	0.001
PA14_05510	1805.730	-1.088	0.001	-1.404	0.000	-1.859	0.000
PA14_05520	4736.241	1.940	0.000	2.765	0.000	2.093	0.000
PA14_05530	2897.280	0.970	0.001	1.665	0.000	1.395	0.000
PA14_05540	12052.797	1.041	0.003	2.184	0.000	1.167	0.005
PA14_05550	6883.634	0.891	0.001	1.669	0.000	0.783	0.015
PA14_05560	4828.490	0.960	0.001	0.967	0.001	2.265	0.000
PA14_05580	2550.325	0.273	0.360	0.796	0.001	2.075	0.000
PA14_05590	1162.797	0.112	0.783	0.058	0.866	0.247	0.546
PA14_05600	1336.723	-0.200	0.559	-0.112	0.710	0.702	0.032
PA14_05620	9356.674	-0.486	0.085	-0.652	0.010	-0.123	0.755
PA14_05630	26.828	0.916	0.190	1.700	0.003	1.045	0.184
PA14_05640	46.460	0.848	0.091	0.560	0.232	-0.184	0.816
PA14_05650	119.135	0.502	0.288	0.575	0.150	0.516	0.346
PA14_05660	2796.019	-0.509	0.074	-1.046	0.000	-0.647	0.050
PA14_05690	206.024	-0.013	0.980	-0.477	0.185	-3.281	0.000
PA14_05700	72.321	0.036	0.958	0.208	0.647	-1.486	0.010
PA14_05740	35.985	-0.033	0.976	2.113	0.000	-0.473	0.632
PA14_05750	33.304	0.066	0.939	1.871	0.000	0.270	0.747
PA14_05770	201.028	1.376	0.001	3.471	0.000	1.870	0.000
PA14_05775	2.328	1.064	0.470	0.591	0.654	0.582	0.686
PA14_05790	121.199	0.922	0.019	2.637	0.000	0.945	0.044
PA14_05810	90.260	0.361	0.472	1.717	0.000	-0.022	0.974
PA14_05820	848.260	-2.066	0.000	-4.299	0.000	1.791	0.000
PA14_05840	5499.491	-2.592	0.000	-5.770	0.000	0.378	0.279
PA14_05850	567.280	0.448	0.275	0.995	0.003	0.656	0.145

PA14_05080	791.918	2.179	0.000
PA14_05110	793.045	0.315	0.196
PA14_05120	302.562	1.897	0.000
PA14_05130	1359.289	1.905	0.000
PA14_05150	946.128	1.514	0.000
PA14_05160	5626.898	0.619	0.019
PA14_05180	2223.186	0.440	0.046
PA14_05190	2162.373	-1.015	0.000
PA14_05200	787.869	-1.601	0.000
PA14_05210	578.428	-0.363	0.305
PA14_05220	957.097	-0.384	0.091
PA14_05230	803.865	-0.579	0.013
PA14_05250	1140.589	1.167	0.000
PA14_05260	3195.195	1.026	0.000
PA14_05270	467.899	0.439	0.155
PA14_05280	340.355	0.270	0.320
PA14_05290	3045.218	0.336	0.123
PA14_05300	1334.504	1.083	0.000
PA14_05310	2478.425	0.122	0.601
PA14_05320	8892.912	-0.443	0.043
PA14_05330	8643.612	0.039	0.893
PA14_05340	1869.586	1.255	0.032
PA14_05360	18973.710	1.803	0.000
PA14_05380	1131.787	0.459	0.178
PA14_05390	11246.487	-0.046	0.859
PA14_05400	1164.729	-0.562	0.012
PA14_05410	518.384	-0.766	0.001
PA14_05420	232.630	-0.596	0.144
PA14_05430	58.239	0.272	0.609
PA14_05440	773.411	-0.061	0.818
PA14_05450	169.749	0.576	0.118
PA14_05460	1868.513	0.308	0.179
PA14_05480	916.300	-0.033	0.898
PA14_05500	10995.082	-0.585	0.077
PA14_05510	9397.884	-1.102	0.000
PA14_05520	4707.565	-1.074	0.000
PA14_05530	3305.071	-0.110	0.684
PA14_05540	8205.583	0.164	0.493
PA14_05550	5928.634	-0.207	0.353
PA14_05560	5045.856	1.907	0.000
PA14_05580	4738.512	0.183	0.463
PA14_05590	1451.428	-0.723	0.001
PA14_05600	936.069	1.076	0.012
PA14_05620	8109.012	0.686	0.009
PA14_05630	60.297	0.683	0.121
PA14_05640	42.803	0.230	0.689
PA14_05650	202.740	0.640	0.029
PA14_05660	10646.943	-1.001	0.000
PA14_05690	143.037	-0.365	0.263
PA14_05700	109.216	0.013	0.970
PA14_05740	60.637	0.132	0.776
PA14_05750	60.305	0.428	0.569
PA14_05770	480.246	-0.160	0.547
PA14_05775	24.220	-0.702	0.308
PA14_05790	216.410	-0.601	0.032
PA14_05810	300.640	-0.855	0.001
PA14_05820	385.995	-1.789	0.000
PA14_05840	3780.855	-4.291	0.000
PA14_05850	3751.758	-3.011	0.000

PA14_05860	23946.252	1.205	0.241	2.020	0.020	1.794	0.084	PA14_05860	17962.840	-1.464	0.006
PA14_05870	94.123	0.522	0.290	1.464	0.000	0.963	0.063	PA14_05870	205.645	0.019	0.931
PA14_05880	204.583	-0.116	0.838	0.772	0.029	4.425	0.000	PA14_05880	442.155	-1.705	0.000
PA14_05890	128.720	0.011	0.985	0.242	0.564	3.206	0.000	PA14_05890	355.397	-0.889	0.000
PA14_05910	100.448	-0.985	0.015	-0.788	0.032	1.100	0.012	PA14_05910	345.215	-0.378	0.160
PA14_05920	272.482	0.130	0.737	0.382	0.168	0.026	0.955	PA14_05920	746.497	0.282	0.266
PA14_05950	587.868	0.616	0.025	1.274	0.000	1.249	0.000	PA14_05950	907.237	0.899	0.000
PA14_05960	2353.982	-0.457	0.148	0.256	0.393	-0.924	0.007	PA14_05960	4547.298	0.427	0.165
PA14_05970	99.691	-0.346	0.486	-0.107	0.814	0.116	0.852	PA14_05970	213.046	0.529	0.067
PA14_05990	123.435	-0.434	0.280	-0.336	0.338	-1.924	0.000	PA14_05990	347.790	-0.019	0.923
PA14_06000	3851.591	-1.775	0.000	-3.557	0.000	-2.039	0.000	PA14_06000	11065.997	-0.717	0.007
PA14_06010	760.910	-0.975	0.001	-0.700	0.013	-0.623	0.092	PA14_06010	4754.199	-0.394	0.071
PA14_06030	490.897	0.242	0.478	0.414	0.127	-0.543	0.132	PA14_06030	1144.294	0.084	0.762
PA14_06040	1037.248	-1.032	0.000	-0.785	0.001	0.112	0.766	PA14_06040	6797.958	-0.727	0.000
PA14_06060	169.835	0.672	0.077	0.829	0.013	0.190	0.726	PA14_06060	481.930	0.141	0.623
PA14_06070	246.023	0.164	0.693	0.268	0.401	-0.826	0.040	PA14_06070	609.797	0.300	0.246
PA14_06080	79.333	0.881	0.028	1.259	0.000	0.397	0.478	PA14_06080	162.729	-0.153	0.645
PA14_06090	19.683	-0.091	0.927	0.443	0.495	1.127	0.132	PA14_06090	36.789	0.271	0.624
PA14_06120	62.942	-0.762	0.096	-0.402	0.340	-0.850	0.128	PA14_06120	352.582	-1.567	0.000
PA14_06130	69.034	-1.408	0.001	-1.448	0.000	-1.015	0.056	PA14_06130	374.658	-2.175	0.000
PA14_06150	295.095	-0.347	0.357	0.049	0.896	-0.469	0.267	PA14_06150	738.944	0.234	0.365
PA14_06160	390.626	-0.513	0.105	1.290	0.000	2.096	0.000	PA14_06160	324.530	-0.184	0.568
PA14_06170	90.960	-1.263	0.012	-0.571	0.204	1.900	0.000	PA14_06170	44.851	-1.099	0.026
PA14_06180	38.578	-0.692	0.316	-0.459	0.440	2.004	0.001	PA14_06180	37.558	-1.562	0.003
PA14_06190	778.714	0.649	0.151	0.379	0.375	-0.319	0.585	PA14_06190	615.730	1.242	0.000
PA14_06200	7.207	1.876	0.072	2.379	0.009	1.195	0.323	PA14_06200	7.725	-0.419	0.787
PA14_06210	96.595	-0.778	0.071	-0.751	0.050	-2.169	0.000	PA14_06210	284.022	-0.098	0.711
PA14_06230	264.301	5.495	0.000	2.741	0.000	0.462	0.519	PA14_06230	68.443	0.951	0.020
PA14_06240	72.160	0.677	0.167	1.321	0.001	0.545	0.365	PA14_06240	129.333	0.334	0.329
PA14_06250	989.420	1.159	0.006	1.743	0.000	-0.406	0.490	PA14_06250	1301.882	-0.142	0.749
PA14_06260	84.051	0.201	0.702	0.216	0.604	-1.021	0.061	PA14_06260	336.324	1.284	0.000
PA14_06270	51.566	-0.941	0.039	-1.152	0.004	-0.981	0.081	PA14_06270	232.387	-1.757	0.000
PA14_06280	88.272	-0.227	0.651	0.120	0.775	-1.874	0.001	PA14_06280	316.518	0.370	0.258
PA14_06290	7427.065	1.811	0.000	0.018	0.955	-0.231	0.531	PA14_06290	6901.274	0.372	0.092
PA14_06300	468.501	-0.141	0.910	-0.335	0.714	1.123	0.261	PA14_06300	1691.794	-2.941	0.000
PA14_06310	662.490	-1.571	0.000	-2.778	0.000	0.268	0.553	PA14_06310	4445.961	-3.105	0.000
PA14_06320	184.977	0.419	0.338	1.673	0.000	1.885	0.000	PA14_06320	549.881	1.385	0.000
PA14_06330	336.934	0.707	0.038	0.658	0.036	0.665	0.105	PA14_06330	768.672	-0.022	0.903
PA14_06340	76.216	-0.904	0.033	-1.001	0.008	-0.265	0.648	PA14_06340	324.040	-0.535	0.044
PA14_06350	22.632	0.121	0.899	0.526	0.402	1.513	0.030	PA14_06350	102.136	-0.065	0.868
PA14_06360	18.317	0.676	0.437	1.180	0.078	2.066	0.007	PA14_06360	72.012	1.379	0.001
PA14_06390	278.935	-0.233	0.579	0.018	0.963	1.433	0.000	PA14_06390	2593.577	-1.744	0.000
PA14_06400	141.663	0.565	0.169	0.545	0.134	0.701	0.135	PA14_06400	239.188	0.510	0.069
PA14_06420	158.997	2.897	0.000	0.769	0.107	6.197	0.000	PA14_06420	752.103	5.209	0.000
PA14_06430	72.922	3.111	0.000	-0.543	0.414	4.967	0.000	PA14_06430	641.067	4.628	0.000
PA14_06450	290.777	2.167	0.000	0.174	0.698	5.984	0.000	PA14_06450	874.032	5.171	0.000
PA14_06460	782.871	2.288	0.000	0.727	0.040	5.854	0.000	PA14_06460	1397.227	4.032	0.000
PA14_06480	280.964	1.918	0.000	0.361	0.318	5.245	0.000	PA14_06480	1201.028	3.180	0.000
PA14_06500	3739.263	-0.755	0.012	-1.535	0.000	0.684	0.059	PA14_06500	4985.545	2.743	0.000
PA14_06510	385.989	-0.380	0.246	-0.757	0.005	0.681	0.049	PA14_06510	996.528	1.119	0.000
PA14_06530	69.487	-0.815	0.065	-0.893	0.022	-0.320	0.587	PA14_06530	285.187	0.119	0.695
PA14_06540	406.093	-0.657	0.106	-0.782	0.030	0.761	0.099	PA14_06540	679.020	0.937	0.000
PA14_06570	82.780	-0.251	0.711	0.333	0.524	0.464	0.494	PA14_06570	163.016	0.746	0.014
PA14_06580	360.877	-1.095	0.000	-1.216	0.000	0.870	0.008	PA14_06580	2474.315	-2.617	0.000
PA14_06600	4433.286	-0.037	0.949	-2.713	0.000	-0.147	0.797	PA14_06600	35987.620	0.202	0.502
PA14_06620	86.977	-0.177	0.796	-0.722	0.132	0.215	0.763	PA14_06620	235.742	-0.464	0.324
PA14_06640	687.695	0.949	0.000	-0.174	0.547	-0.453	0.197	PA14_06640	5395.970	-0.077	0.833
PA14_06650	409.850	-1.530	0.000	-3.684	0.000	1.661	0.000	PA14_06650	1322.192	-0.997	0.000
PA14_06660	62.042	-1.174	0.013	-4.534	0.000	1.661	0.001	PA14_06660	240.037	-1.402	0.000

PA14_06670	187.069	-1.383	0.000	-4.283	0.000	1.588	0.000	PA14_06670	949.089	-1.499	0.000
PA14_06680	113.489	-0.899	0.063	-2.006	0.000	1.879	0.000	PA14_06680	622.275	-1.171	0.000
PA14_06690	73.259	-1.646	0.000	-4.376	0.000	1.775	0.000	PA14_06690	558.460	-1.561	0.000
PA14_06700	72.756	-1.767	0.001	-4.131	0.000	1.251	0.031	PA14_06700	433.312	-1.925	0.000
PA14_06710	122.627	-0.913	0.056	-3.689	0.000	1.543	0.002	PA14_06710	741.185	-1.580	0.000
PA14_06720	193.388	-0.993	0.017	-3.751	0.000	1.765	0.000	PA14_06720	1633.189	-1.792	0.000
PA14_06730	130.558	-0.571	0.244	-3.737	0.000	2.165	0.000	PA14_06730	1492.561	-2.249	0.000
PA14_06740	126.087	-1.026	0.025	-3.331	0.000	2.219	0.000	PA14_06740	1943.769	-2.705	0.000
PA14_06750	4965.893	-0.259	0.468	-3.789	0.000	2.309	0.000	PA14_06750	80362.271	-3.684	0.000
PA14_06770	359.601	0.209	0.561	-1.643	0.000	2.888	0.000	PA14_06770	7883.496	-5.182	0.000
PA14_06790	87.146	0.094	0.909	-0.717	0.202	4.912	0.000	PA14_06790	954.077	-4.562	0.000
PA14_06800	52.852	0.240	0.763	-0.779	0.206	4.384	0.000	PA14_06800	427.982	-3.595	0.000
PA14_06810	3049.813	4.177	0.000	-1.196	0.001	8.706	0.000	PA14_06810	79877.083	-10.234	0.000
PA14_06830	4601.717	2.607	0.000	-0.170	0.679	8.575	0.000	PA14_06830	33777.703	-8.086	0.000
PA14_06840	915.206	1.750	0.000	0.222	0.685	9.104	0.000	PA14_06840	2175.340	-6.104	0.000
PA14_06860	159.080	1.209	0.005	-1.602	0.000	3.107	0.000	PA14_06860	2476.004	-3.361	0.000
PA14_06870	2628.444	0.136	0.772	-0.821	0.012	-1.724	0.000	PA14_06870	8233.494	1.820	0.000
PA14_06875	3024.796	-1.006	0.326	-1.633	0.056	-0.514	0.664	#N/A	#N/A	#N/A	#N/A
PA14_06880	89.126	0.061	0.946	0.101	0.878	-0.011	0.990	PA14_06880	144.933	0.483	0.406
PA14_06890	227.242	-0.663	0.054	-1.390	0.000	0.103	0.838	PA14_06890	1451.347	-2.682	0.000
PA14_06900	33.844	-1.243	0.019	-1.099	0.021	-0.037	0.964	PA14_06900	718.830	-2.380	0.000
PA14_06920	30.702	-0.856	0.170	-1.081	0.044	0.857	0.195	PA14_06920	748.020	-1.730	0.000
PA14_06930	37.560	-0.285	0.668	-0.460	0.371	0.765	0.216	PA14_06930	416.277	-0.935	0.000
PA14_06940	96.647	0.140	0.786	0.385	0.293	1.096	0.010	PA14_06940	291.729	-1.348	0.000
PA14_06950	214.119	0.552	0.151	0.799	0.015	-0.476	0.328	PA14_06950	528.075	0.810	0.001
PA14_06960	366.229	-3.625	0.000	-3.873	0.000	2.010	0.000	PA14_06960	505.274	-1.894	0.000
PA14_06970	66.687	-0.996	0.033	-0.541	0.206	1.365	0.004	PA14_06970	344.705	-1.102	0.004
PA14_06980	96.420	-0.193	0.774	-0.276	0.599	-1.287	0.039	PA14_06980	621.655	0.172	0.487
PA14_06990	548.046	-0.352	0.328	-0.606	0.041	-0.300	0.484	PA14_06990	3341.452	-0.563	0.009
PA14_07000	142.990	-0.373	0.344	-0.569	0.077	-0.443	0.332	PA14_07000	511.292	0.534	0.033
PA14_07010	21.694	0.070	0.946	0.521	0.428	0.695	0.411	PA14_07010	90.893	-0.706	0.048
PA14_07020	113.699	-0.369	0.598	-0.140	0.824	-0.153	0.854	PA14_07020	189.248	-0.603	0.036
PA14_07030	240.224	-1.270	0.000	-1.470	0.000	-1.725	0.000	PA14_07030	491.041	0.026	0.933
PA14_07040	1469.106	0.003	0.993	-0.465	0.049	-1.246	0.000	PA14_07040	2828.007	1.608	0.000
PA14_07050	66.445	-0.978	0.025	-0.339	0.411	0.488	0.353	PA14_07050	466.937	-2.088	0.000
PA14_07060	219.592	0.850	0.007	0.868	0.003	0.189	0.696	PA14_07060	474.397	2.047	0.000
PA14_07070	1027.336	0.200	0.580	-0.385	0.175	-2.598	0.000	PA14_07070	3688.796	0.460	0.125
PA14_07090	2131.789	-1.939	0.000	-2.254	0.000	-0.017	0.974	PA14_07090	1579.758	-0.534	0.013
PA14_07110	3546.150	-1.522	0.000	-1.304	0.000	1.347	0.000	PA14_07110	2876.293	-0.485	0.021
PA14_07130	5217.992	-0.122	0.725	-0.075	0.796	-0.023	0.956	PA14_07130	4827.130	0.570	0.008
PA14_07140	462.217	0.494	0.113	0.921	0.001	0.233	0.572	PA14_07140	656.925	1.272	0.000
PA14_07150	65.232	-0.281	0.627	-0.535	0.232	-0.696	0.251	PA14_07150	155.828	-0.518	0.107
PA14_07170	429.092	0.444	0.233	0.945	0.002	-0.082	0.874	PA14_07170	1054.490	1.323	0.000
PA14_07190	650.701	-0.196	0.591	-0.079	0.811	-1.852	0.000	PA14_07190	1059.118	0.702	0.002
PA14_07200	632.140	-0.036	0.949	0.411	0.253	1.500	0.000	PA14_07200	1975.508	-1.946	0.000
PA14_07210	1452.620	0.144	0.726	0.584	0.047	0.610	0.108	PA14_07210	4570.237	-0.739	0.000
PA14_07230	5640.264	-0.229	0.408	-0.179	0.465	-0.867	0.001	PA14_07230	9431.053	-0.052	0.862
PA14_07240	161.302	0.970	0.004	1.133	0.000	0.972	0.018	PA14_07240	154.682	1.124	0.000
PA14_07250	54.200	-0.062	0.929	0.245	0.603	-0.112	0.876	PA14_07250	207.912	-1.460	0.000
PA14_07260	246.697	-0.178	0.686	0.055	0.885	0.044	0.934	PA14_07260	332.875	0.912	0.000
PA14_07280	107.549	0.708	0.059	0.807	0.015	-0.040	0.945	PA14_07280	231.725	-0.017	0.983
PA14_07290	785.500	0.679	0.138	1.295	0.001	0.962	0.059	PA14_07290	1099.310	0.747	0.001
PA14_07300	590.403	0.342	0.343	0.853	0.003	1.086	0.002	PA14_07300	1237.805	-0.106	0.680
PA14_07310	377.917	0.789	0.080	1.278	0.001	1.322	0.008	PA14_07310	530.002	0.293	0.371
PA14_07330	180.445	0.237	0.607	1.092	0.001	0.924	0.030	PA14_07330	311.892	0.237	0.590
PA14_07340	147.257	0.680	0.044	0.856	0.004	-0.341	0.478	PA14_07340	325.419	1.402	0.000
PA14_07355	132.504	-0.278	0.507	-2.320	0.000	-0.379	0.416	PA14_07355	415.850	-2.310	0.000
PA14_07360	76.803	-0.876	0.041	-0.724	0.060	-0.813	0.123	PA14_07360	276.516	-0.428	0.114
PA14_07370	117.337	-1.642	0.000	-2.141	0.000	-0.944	0.103	PA14_07370	426.721	-1.446	0.000

PA14_07380	64.236	-0.432	0.432	-1.637	0.000	-1.293	0.028	PA14_07380	257.389	-0.620	0.026
PA14_07400	96.506	-0.147	0.767	-0.406	0.264	-0.522	0.293	PA14_07400	235.699	-0.227	0.459
PA14_07410	69.040	0.440	0.350	-0.275	0.522	0.103	0.874	PA14_07410	206.313	-0.785	0.006
PA14_07420	132.113	0.401	0.319	0.151	0.690	-0.513	0.290	PA14_07420	417.416	-0.469	0.062
PA14_07430	823.337	-2.595	0.000	-3.946	0.000	-4.236	0.000	PA14_07430	4200.566	-2.700	0.000
PA14_07440	93.008	0.388	0.390	0.786	0.024	0.441	0.392	PA14_07440	229.144	0.516	0.075
PA14_07450	1415.501	0.732	0.015	1.479	0.000	0.046	0.923	PA14_07450	2164.805	1.320	0.000
PA14_07460	170.284	0.182	0.720	0.700	0.048	0.661	0.162	PA14_07460	466.493	-1.137	0.000
PA14_07470	161.774	0.821	0.143	2.800	0.000	2.979	0.000	#N/A	#N/A	#N/A	#N/A
PA14_07480	8341.438	0.157	0.683	2.042	0.000	3.331	0.000	PA14_07480	9157.174	-0.297	0.184
PA14_07500	613.617	-1.603	0.000	-1.833	0.000	1.617	0.000	PA14_07500	1818.306	-2.524	0.000
PA14_07520	19135.393	0.319	0.265	0.726	0.002	0.847	0.004	PA14_07520	33216.117	1.310	0.000
PA14_07530	4323.524	0.861	0.026	1.530	0.000	0.682	0.147	PA14_07530	7639.549	0.596	0.005
PA14_07550	312.086	0.951	0.010	2.493	0.000	2.938	0.000	PA14_07550	383.227	3.006	0.000
PA14_07560	3199.861	1.255	0.000	2.897	0.000	2.439	0.000	PA14_07560	3419.763	2.711	0.000
PA14_07570	530.442	1.180	0.000	2.437	0.000	2.514	0.000	PA14_07570	660.511	0.994	0.000
PA14_07580	66.079	-0.929	0.053	-0.467	0.295	-0.603	0.316	PA14_07580	505.455	-1.633	0.000
PA14_07590	137.566	0.463	0.256	0.966	0.003	0.751	0.090	PA14_07590	254.401	0.107	0.759
PA14_07600	208.445	0.743	0.059	1.598	0.000	1.566	0.000	PA14_07600	219.487	0.764	0.006
PA14_07620	236.790	0.222	0.573	0.282	0.371	0.979	0.007	PA14_07620	557.139	-0.464	0.060
PA14_07630	129.702	-0.956	0.021	-0.795	0.031	3.454	0.000	PA14_07630	562.697	-2.807	0.000
PA14_07650	2408.461	-1.885	0.000	-3.343	0.000	1.594	0.000	PA14_07650	13367.065	-3.019	0.000
PA14_07660	3706.182	-2.066	0.000	-3.270	0.000	1.961	0.000	PA14_07660	17616.856	-3.023	0.000
PA14_07680	20015.712	-2.312	0.000	-3.369	0.000	1.592	0.000	PA14_07680	77794.876	-3.060	0.000
PA14_07690	282.092	-0.244	0.579	-0.375	0.282	0.491	0.270	PA14_07690	934.214	-0.305	0.220
PA14_07700	775.406	0.358	0.238	0.140	0.629	0.622	0.058	PA14_07700	2046.442	-0.826	0.000
PA14_07710	332.111	-0.165	0.717	-0.522	0.116	-0.672	0.121	PA14_07710	2103.452	-2.073	0.000
PA14_07730	818.005	1.083	0.000	1.893	0.000	1.305	0.000	PA14_07730	1204.505	1.294	0.000
PA14_07740	235.171	0.226	0.528	0.160	0.604	-1.754	0.000	PA14_07740	350.537	0.485	0.125
PA14_07760	2095.644	0.103	0.838	-0.019	0.966	-1.784	0.000	PA14_07760	2963.963	-0.572	0.008
PA14_07770	23827.476	0.407	0.142	0.634	0.008	-0.209	0.559	PA14_07770	29528.433	0.655	0.001
PA14_07780	690.239	0.302	0.470	-0.588	0.077	-0.391	0.397	PA14_07780	4334.988	-1.763	0.000
PA14_07790	118.298	0.255	0.560	-0.166	0.662	0.026	0.964	PA14_07790	846.207	-0.270	0.253
PA14_07800	279.523	-0.100	0.833	-0.301	0.366	0.450	0.286	PA14_07800	1591.610	0.178	0.470
PA14_07810	224.849	-0.324	0.371	-0.726	0.012	-1.182	0.002	PA14_07810	680.067	-0.411	0.093
PA14_07820	510.232	0.028	0.949	-0.231	0.422	0.186	0.644	PA14_07820	1156.024	-0.414	0.071
PA14_07840	602.359	0.570	0.073	0.561	0.052	1.394	0.000	PA14_07840	956.758	-0.243	0.316
PA14_07850	364.694	-0.454	0.198	-1.065	0.000	-0.966	0.013	PA14_07850	953.339	-0.199	0.422
PA14_07860	446.695	0.001	0.999	-0.320	0.298	1.768	0.000	PA14_07860	350.500	-0.487	0.062
PA14_07870	666.948	-0.051	0.906	-1.294	0.000	0.910	0.006	PA14_07870	504.606	-0.363	0.161
PA14_07890	199.090	0.181	0.701	-0.385	0.281	1.322	0.001	PA14_07890	241.034	-0.355	0.196
PA14_07900	42.852	-0.123	0.867	-0.195	0.718	0.710	0.252	PA14_07900	95.078	-0.099	0.782
PA14_07910	403.581	0.317	0.405	1.414	0.000	0.701	0.072	PA14_07910	456.814	0.699	0.004
PA14_07930	603.557	0.207	0.578	0.444	0.122	0.015	0.974	PA14_07930	1504.791	0.335	0.164
PA14_07940	1315.158	-0.125	0.719	0.277	0.289	-0.247	0.490	PA14_07940	2300.685	0.689	0.001
PA14_07950	212.871	-0.273	0.447	-0.425	0.141	-1.612	0.000	PA14_07950	3748.459	-3.049	0.000
PA14_07960	1021.131	1.235	0.000	1.566	0.000	-0.703	0.040	PA14_07960	2593.334	0.352	0.129
PA14_07970	46.659	-1.186	0.012	-0.584	0.184	-2.777	0.000	PA14_07970	1608.733	-4.243	0.000
PA14_07980	169.286	-0.884	0.049	0.125	0.792	-0.789	0.145	PA14_07980	5330.578	-4.452	0.000
PA14_07990	256.950	-1.486	0.000	-1.243	0.001	-3.078	0.000	PA14_07990	7794.323	-4.682	0.000
PA14_08000	136.354	-1.057	0.002	-1.083	0.000	-1.453	0.001	PA14_08000	2864.298	-4.121	0.000
PA14_08010	161.529	-1.513	0.000	-1.086	0.001	-1.256	0.004	PA14_08010	4377.617	-4.655	0.000
PA14_08020	85.636	-0.953	0.026	-0.762	0.050	-1.430	0.006	PA14_08020	3040.976	-5.239	0.000
PA14_08030	170.130	-1.505	0.000	-1.497	0.000	-1.975	0.000	PA14_08030	6462.390	-5.108	0.000
PA14_08040	134.456	-1.462	0.000	-1.702	0.000	-2.729	0.000	PA14_08040	4390.781	-5.059	0.000
PA14_08050	881.576	-1.353	0.000	-1.595	0.000	-2.186	0.000	PA14_08050	22744.421	-5.077	0.000
PA14_08060	607.996	-0.873	0.011	-1.213	0.000	-1.849	0.000	PA14_08060	17448.859	-5.365	0.000
PA14_08070	1054.692	-1.966	0.000	-2.549	0.000	-3.596	0.000	PA14_08070	35511.406	-5.031	0.000
PA14_08090	158.755	-1.638	0.000	-2.337	0.000	-3.412	0.000	PA14_08090	8483.374	-4.989	0.000

PA14_08100	68.630	-1.726	0.000	-2.019	0.000	-2.352	0.000	PA14_08100	3234.653	-5.106	0.000
PA14_08110	55.630	-1.109	0.018	-0.411	0.356	-2.198	0.000	PA14_08110	1792.040	-5.241	0.000
PA14_08120	609.434	-1.671	0.000	-1.481	0.000	-2.541	0.000	PA14_08120	20218.112	-5.524	0.000
PA14_08130	98.985	-1.671	0.000	-1.582	0.000	-2.108	0.000	PA14_08130	4451.764	-5.014	0.000
PA14_08140	53.637	-1.282	0.006	-1.094	0.010	-1.419	0.014	PA14_08140	3263.735	-5.489	0.000
PA14_08150	333.809	-1.493	0.000	-1.510	0.000	-1.778	0.000	PA14_08150	10591.542	-5.240	0.000
PA14_08160	39.488	-1.319	0.021	-1.112	0.032	-1.933	0.006	PA14_08160	2028.587	-5.380	0.000
PA14_08180	5.609	-0.984	0.421	-2.212	0.028	-2.326	0.040	PA14_08180	629.668	-5.242	0.000
PA14_08190	12.374	-1.960	0.015	-1.588	0.028	-2.155	0.024	PA14_08190	1325.176	-5.361	0.000
PA14_08200	160.270	-1.807	0.000	-2.320	0.000	-3.673	0.000	PA14_08200	11769.877	-5.384	0.000
PA14_08210	318.857	-1.038	0.008	-1.365	0.000	-2.568	0.000	PA14_08210	16060.168	-5.611	0.000
PA14_08220	236.414	-1.681	0.000	-1.729	0.000	-1.919	0.000	PA14_08220	10719.864	-5.577	0.000
PA14_08230	53.277	-1.934	0.000	-1.655	0.000	-1.520	0.013	PA14_08230	2766.067	-5.053	0.000
PA14_08240	425.774	-1.832	0.000	-2.004	0.000	-2.446	0.000	PA14_08240	16273.061	-5.250	0.000
PA14_08250	54.374	-1.725	0.000	-1.834	0.000	-1.789	0.003	PA14_08250	2609.078	-5.094	0.000
PA14_08260	84.576	-2.045	0.000	-1.952	0.000	-2.751	0.000	PA14_08260	3675.287	-5.230	0.000
PA14_08270	426.671	-1.802	0.000	-1.581	0.000	-1.947	0.000	PA14_08270	13689.106	-5.313	0.000
PA14_08280	369.543	-1.865	0.000	-2.085	0.000	-3.122	0.000	PA14_08280	13072.081	-5.394	0.000
PA14_08300	925.080	-1.473	0.000	-1.526	0.000	-2.354	0.000	PA14_08300	24409.053	-4.923	0.000
PA14_08310	1213.049	-0.664	0.025	-1.012	0.000	-1.361	0.000	PA14_08310	18136.016	-3.945	0.000
PA14_08320	466.687	-0.827	0.014	-1.283	0.000	-1.116	0.005	PA14_08320	11078.761	-3.779	0.000
PA14_08330	149.566	-0.825	0.012	-1.099	0.000	-2.226	0.000	PA14_08330	3264.698	-3.318	0.000
PA14_08340	532.172	0.516	0.092	0.625	0.021	-0.041	0.929	PA14_08340	1605.432	-0.313	0.189
PA14_08350	2129.972	0.127	0.745	0.308	0.291	0.516	0.154	PA14_08350	4698.045	-0.541	0.012
PA14_08360	138.862	-0.659	0.091	-0.845	0.013	-0.578	0.225	PA14_08360	1179.126	-0.797	0.000
PA14_08370	4718.322	-0.323	0.294	-0.551	0.033	0.174	0.655	PA14_08370	9995.091	0.383	0.102
PA14_08380	315.282	-0.735	0.064	0.357	0.344	-0.153	0.785	PA14_08380	647.500	0.005	0.970
PA14_08390	4174.230	1.081	0.000	2.183	0.000	1.239	0.000	PA14_08390	2689.964	0.190	0.410
PA14_08400	2148.414	-0.419	0.299	-0.495	0.156	0.417	0.371	PA14_08400	7862.357	-2.047	0.000
PA14_08420	131.684	-1.543	0.000	-2.286	0.000	-0.647	0.134	PA14_08420	851.081	-2.537	0.000
PA14_08430	613.680	-0.068	0.876	-0.270	0.374	-0.334	0.404	PA14_08430	1793.547	-0.240	0.296
PA14_08440	190.897	-0.716	0.023	-0.451	0.122	-0.603	0.122	PA14_08440	736.585	-0.812	0.000
PA14_08450	1021.576	0.101	0.767	0.173	0.511	-0.565	0.070	PA14_08450	2603.469	0.339	0.139
PA14_08460	344.209	-0.475	0.199	-1.083	0.000	-1.005	0.013	PA14_08460	1142.690	0.320	0.160
PA14_08470	577.598	-0.191	0.591	-0.358	0.200	-0.493	0.172	PA14_08470	2126.193	-0.042	0.892
PA14_08480	305.507	0.464	0.171	0.621	0.034	-0.653	0.106	PA14_08480	648.955	0.292	0.258
PA14_08490	2279.405	0.575	0.240	1.298	0.001	0.321	0.598	PA14_08490	2412.167	0.213	0.369
PA14_08500	1708.590	0.101	0.846	0.335	0.367	0.433	0.364	PA14_08500	2464.820	-0.269	0.231
PA14_08510	267.911	0.656	0.106	0.964	0.006	0.872	0.058	PA14_08510	1654.179	-2.461	0.000
PA14_08520	626.474	0.268	0.415	0.238	0.400	0.407	0.252	PA14_08520	1263.165	0.954	0.000
PA14_08540	5791.375	0.522	0.031	0.268	0.259	0.706	0.011	PA14_08540	11794.666	0.844	0.000
PA14_08560	7205.032	0.713	0.009	1.438	0.000	0.730	0.026	PA14_08560	6325.062	0.127	0.553
PA14_08570	3.438	3.426	0.004	0.039	0.978	0.592	0.656	#N/A	#N/A	#N/A	#N/A
PA14_08580	0.000	NA	NA	NA	NA	NA	NA	#N/A	#N/A	#N/A	#N/A
PA14_08590	0.615	-0.837	0.559	-0.783	0.529	-0.946	0.399	#N/A	#N/A	#N/A	#N/A
PA14_08600	103.849	4.651	0.000	-0.120	0.934	0.212	0.891	#N/A	#N/A	#N/A	#N/A
PA14_08610	0.000	NA	NA	NA	NA	NA	NA	#N/A	#N/A	#N/A	#N/A
PA14_08620	3009.325	1.251	0.010	2.614	0.000	2.042	0.000	PA14_08620	3064.435	0.495	0.027
PA14_08630	684.739	0.342	0.483	1.260	0.001	0.834	0.087	PA14_08630	1344.969	-0.101	0.715
PA14_08640	1179.758	0.348	0.448	1.118	0.002	0.602	0.212	PA14_08640	2185.859	-0.466	0.041
PA14_08650	23051.428	1.219	0.003	3.384	0.000	1.861	0.000	#N/A	#N/A	#N/A	#N/A
PA14_08660	1717.821	0.984	0.004	2.505	0.000	1.564	0.000	#N/A	#N/A	#N/A	#N/A
PA14_08670	733.380	0.417	0.403	1.854	0.000	1.245	0.011	#N/A	#N/A	#N/A	#N/A
PA14_08680	9856.307	0.151	0.731	0.405	0.219	-0.759	0.058	PA14_08680	50779.026	1.873	0.000
PA14_08690	5204.701	0.928	0.047	2.347	0.000	3.537	0.000	#N/A	#N/A	#N/A	#N/A
PA14_08695	20904.469	0.718	0.040	1.404	0.000	2.063	0.000	PA14_08695	20798.669	2.023	0.000
PA14_08710	23222.814	0.444	0.167	1.479	0.000	1.456	0.000	PA14_08710	22979.218	1.688	0.000
PA14_08720	19059.866	0.876	0.005	0.951	0.001	1.458	0.000	PA14_08720	15181.175	2.845	0.000
PA14_08730	19067.651	0.660	0.042	0.964	0.001	1.811	0.000	PA14_08730	12227.799	2.890	0.000

PA14_08740	19634.587	0.799	0.009	1.128	0.000	1.744	0.000	PA14_08740	10503.959	2.539	0.000
PA14_08750	16339.173	0.289	0.431	0.365	0.236	-0.443	0.262	PA14_08750	9803.792	1.770	0.000
PA14_08760	68882.493	0.255	0.373	0.384	0.108	0.370	0.239	PA14_08760	41865.866	1.454	0.000
PA14_08780	81776.545	0.172	0.605	0.588	0.018	-0.109	0.782	PA14_08780	68389.652	1.494	0.000
PA14_08790	28265.583	0.960	0.009	1.097	0.001	1.719	0.000	PA14_08790	18537.398	1.930	0.000
PA14_08810	10993.269	0.317	0.298	0.528	0.039	0.375	0.278	PA14_08810	8600.895	1.748	0.000
PA14_08820	77250.627	0.099	0.753	0.289	0.215	-0.236	0.454	PA14_08820	49244.985	1.982	0.000
PA14_08830	19347.706	0.196	0.538	0.037	0.903	-0.934	0.002	PA14_08830	48224.813	1.885	0.000
PA14_08840	40247.902	1.508	0.000	2.361	0.000	1.601	0.000	PA14_08840	11759.347	1.394	0.000
PA14_08850	16591.857	0.980	0.000	1.054	0.000	0.482	0.181	PA14_08850	9506.770	1.592	0.000
PA14_08860	26937.052	1.018	0.001	1.053	0.000	1.052	0.005	PA14_08860	10810.649	2.036	0.000
PA14_08870	3577.820	0.433	0.249	0.165	0.649	0.059	0.911	PA14_08870	4518.904	2.252	0.000
PA14_08880	11359.885	0.238	0.519	0.391	0.190	0.919	0.009	PA14_08880	5891.409	1.616	0.000
PA14_08890	22511.558	0.691	0.028	0.555	0.061	0.466	0.238	PA14_08890	10514.435	1.321	0.000
PA14_08900	14957.755	0.674	0.014	0.614	0.018	0.436	0.211	PA14_08900	8929.435	1.644	0.000
PA14_08910	15980.400	0.724	0.018	0.540	0.064	0.166	0.706	PA14_08910	11841.290	1.557	0.000
PA14_08920	8754.809	0.107	0.799	-0.121	0.716	-0.695	0.059	PA14_08920	9452.574	1.316	0.000
PA14_08930	3044.083	0.666	0.010	0.625	0.010	-0.051	0.899	PA14_08930	3514.017	2.181	0.000
PA14_08940	8221.273	0.865	0.002	1.644	0.000	0.098	0.824	PA14_08940	4321.031	1.970	0.000
PA14_08950	10410.189	0.396	0.156	0.415	0.097	-0.105	0.785	PA14_08950	10910.772	1.517	0.000
PA14_08960	11840.328	0.440	0.115	0.360	0.164	-0.473	0.150	PA14_08960	11413.768	2.109	0.000
PA14_08970	16298.748	0.137	0.666	0.281	0.252	-0.831	0.004	PA14_08970	15743.467	1.752	0.000
PA14_08980	973.194	-0.063	0.914	-0.340	0.386	-1.268	0.005	PA14_08980	4101.736	0.969	0.001
PA14_08990	6233.657	0.380	0.171	1.063	0.000	1.258	0.000	PA14_08990	7287.432	1.801	0.000
PA14_09000	9189.710	0.334	0.303	0.379	0.178	1.170	0.000	PA14_09000	7887.508	1.902	0.000
PA14_09010	4802.231	0.316	0.413	0.119	0.742	0.631	0.117	PA14_09010	4656.091	2.045	0.000
PA14_09020	12654.390	0.097	0.790	0.294	0.268	0.328	0.344	PA14_09020	7866.038	2.010	0.000
PA14_09030	1382.438	0.102	0.845	-0.136	0.738	0.047	0.935	PA14_09030	4003.924	1.263	0.000
PA14_09040	8046.617	0.320	0.299	0.375	0.159	1.080	0.000	PA14_09040	7446.229	1.340	0.000
PA14_09050	43111.536	0.609	0.020	0.826	0.001	1.302	0.000	PA14_09050	29810.726	1.694	0.000
PA14_09070	14077.103	0.773	0.037	0.776	0.023	0.606	0.179	PA14_09070	12904.041	1.551	0.000
PA14_09080	14192.747	0.547	0.123	0.715	0.023	0.352	0.427	PA14_09080	15032.084	1.555	0.000
PA14_09090	3569.729	0.309	0.550	-0.109	0.819	0.159	0.796	PA14_09090	5988.910	1.683	0.002
PA14_09100	17145.509	0.490	0.139	0.327	0.292	0.445	0.259	PA14_09100	13010.292	2.013	0.000
PA14_09115	42653.143	0.419	0.099	0.594	0.008	0.572	0.049	PA14_09115	28883.573	1.852	0.000
PA14_09130	1527.025	0.306	0.531	-0.159	0.716	-0.923	0.055	PA14_09130	1973.265	1.256	0.000
PA14_09150	10541.788	-1.257	0.000	-2.403	0.000	-2.113	0.000	PA14_09150	43030.884	-2.189	0.000
PA14_09160	2118.089	-1.170	0.000	-1.874	0.000	-1.726	0.000	PA14_09160	10678.921	-0.190	0.387
PA14_09180	1659.429	0.637	0.032	0.414	0.145	0.055	0.902	PA14_09180	2111.187	1.540	0.000
PA14_09195	887.965	0.876	0.010	1.687	0.000	1.760	0.000	PA14_09195	1761.419	0.509	0.023
PA14_09200	1392.707	0.237	0.432	0.389	0.112	0.105	0.782	PA14_09200	2402.910	0.697	0.001
PA14_09210	2610.584	-2.035	0.000	-1.878	0.000	-0.115	0.828	PA14_09210	56.378	-0.321	0.508
PA14_09220	827.115	-1.933	0.000	-1.457	0.000	0.349	0.308	PA14_09220	23.363	-0.840	0.196
PA14_09230	6720.394	-1.167	0.005	-0.124	0.792	1.555	0.001	PA14_09230	39.901	-1.818	0.000
PA14_09240	2251.589	-1.838	0.000	-1.827	0.000	0.456	0.147	PA14_09240	38.064	-1.602	0.001
PA14_09260	237.551	-0.148	0.771	-0.653	0.068	0.383	0.443	PA14_09260	46.053	-0.792	0.104
PA14_09270	5021.130	-1.450	0.000	-0.950	0.000	-0.111	0.782	PA14_09270	91.310	-1.069	0.007
PA14_09280	5099.428	-1.121	0.000	-0.737	0.003	0.332	0.344	PA14_09280	107.667	-1.329	0.030
PA14_09290	530.569	-1.126	0.003	-1.059	0.003	0.080	0.891	PA14_09290	29.996	-2.012	0.134
PA14_09300	1571.143	-0.704	0.027	-0.403	0.188	0.518	0.184	PA14_09300	37.278	-0.509	0.371
PA14_09320	7527.156	0.117	0.830	0.530	0.159	2.193	0.000	PA14_09320	92.825	-1.218	0.004
PA14_09340	15673.439	-0.814	0.004	-0.499	0.073	1.313	0.000	PA14_09340	245.784	-0.943	0.000
PA14_09350	224.740	-0.977	0.002	-0.248	0.440	0.776	0.035	PA14_09350	15.236	-0.820	0.342
PA14_09370	797.695	-1.098	0.000	-0.422	0.111	0.642	0.051	PA14_09370	12.249	0.253	0.874
PA14_09380	1004.366	-1.108	0.000	-0.613	0.021	-0.050	0.909	PA14_09380	28.842	-0.861	0.165
PA14_09400	7182.845	-5.331	0.000	-7.252	0.000	-7.526	0.000	PA14_09400	13444.097	-4.893	0.000
PA14_09410	433.007	-5.140	0.000	-6.083	0.000	-6.332	0.000	PA14_09410	4839.969	-5.305	0.000
PA14_09420	0.000	NA	NA	NA	NA	NA	NA	PA14_09420	2444.843	-5.139	0.000
PA14_09440	560.778	-5.062	0.000	-6.712	0.000	-5.707	0.000	PA14_09440	2583.473	-4.104	0.000

PA14_09450	35.946	-5.284	0.000	-6.534	0.000	-5.800	0.000	PA14_09450	750.277	-4.059	0.000
PA14_09460	654.343	-4.521	0.000	-7.480	0.000	-6.754	0.000	PA14_09460	1637.654	-4.273	0.000
PA14_09470	4325.065	-4.648	0.000	-8.446	0.000	-7.009	0.000	PA14_09470	2779.661	-3.056	0.000
PA14_09480	521.741	-4.752	0.000	-8.136	0.000	-7.162	0.000	PA14_09480	774.654	-3.001	0.000
PA14_09490	1312.877	-3.733	0.000	-4.099	0.000	-4.041	0.000	PA14_09490	1285.067	-2.285	0.000
PA14_09500	54.032	-2.035	0.000	-0.881	0.027	-0.648	0.246	PA14_09500	612.391	-2.180	0.000
PA14_09520	397.894	-1.877	0.000	-0.677	0.007	-0.803	0.013	PA14_09520	1996.218	-0.900	0.000
PA14_09530	212.431	-2.125	0.000	-1.302	0.000	-0.670	0.093	PA14_09530	1786.336	-1.853	0.000
PA14_09540	39.912	-2.480	0.000	-1.385	0.002	-0.747	0.240	PA14_09540	559.440	-2.326	0.000
PA14_09550	293.623	-0.615	0.038	-1.131	0.000	0.088	0.841	PA14_09550	1529.936	-0.579	0.009
PA14_09570	46.893	0.846	0.090	1.386	0.001	0.413	0.543	PA14_09570	93.290	0.679	0.063
PA14_09580	201.254	0.234	0.549	0.074	0.834	-1.196	0.003	PA14_09580	420.530	1.415	0.000
PA14_09600	379.892	-0.022	0.969	-0.205	0.573	-1.708	0.000	PA14_09600	1288.096	-0.275	0.266
PA14_09610	121.901	0.473	0.278	0.670	0.064	0.478	0.346	PA14_09610	348.490	2.044	0.000
PA14_09630	399.567	-0.021	0.967	0.389	0.217	1.140	0.002	PA14_09630	882.233	0.672	0.003
PA14_09660	323.093	0.809	0.020	2.218	0.000	2.233	0.000	PA14_09660	574.421	2.035	0.000
PA14_09680	236.997	0.399	0.276	1.976	0.000	2.735	0.000	PA14_09680	386.748	1.999	0.000
PA14_09690	68.410	0.555	0.313	1.968	0.000	1.643	0.002	PA14_09690	237.723	2.506	0.000
PA14_09700	344.396	0.179	0.643	-0.475	0.104	-0.335	0.416	PA14_09700	476.618	-0.502	0.093
PA14_09710	56.700	0.711	0.149	-1.374	0.002	-1.313	0.037	PA14_09710	68.662	1.013	0.011
PA14_09730	100.510	2.272	0.000	-0.118	0.831	0.359	0.597	PA14_09730	66.807	1.550	0.000
PA14_09740	52.147	1.124	0.019	-0.850	0.080	0.584	0.358	PA14_09740	55.956	0.518	0.269
PA14_09750	129.819	0.678	0.054	-0.304	0.388	-0.676	0.147	PA14_09750	325.742	0.636	0.018
PA14_09760	244.795	1.400	0.000	0.993	0.003	-0.718	0.146	PA14_09760	468.982	1.246	0.000
PA14_09770	106.855	0.401	0.340	-0.385	0.297	-0.248	0.652	PA14_09770	233.189	0.930	0.001
PA14_09780	39.267	0.382	0.589	0.392	0.499	-1.504	0.059	PA14_09780	138.032	1.619	0.000
PA14_09790	297.023	-0.010	0.982	-0.774	0.004	-0.878	0.014	PA14_09790	598.169	0.402	0.099
PA14_09810	51.854	0.475	0.349	-0.379	0.404	-0.839	0.174	PA14_09810	90.281	0.688	0.174
PA14_09820	1083.218	-0.409	0.258	-0.588	0.056	-0.859	0.026	PA14_09820	2663.563	-0.042	0.868
PA14_09850	17.088	-0.067	0.954	0.965	0.157	-0.431	0.686	PA14_09850	33.830	0.096	0.924
PA14_09870	65.820	0.121	0.867	0.401	0.423	1.413	0.011	PA14_09870	85.462	0.098	0.870
PA14_09880	3.257	0.129	0.939	0.353	0.772	-1.118	0.383	PA14_09880	7.445	-0.357	0.857
PA14_09890	588.086	-0.273	0.429	-0.520	0.059	0.044	0.923	PA14_09890	975.080	0.265	0.287
PA14_09900	878.250	-4.360	0.000	-5.137	0.000	-4.643	0.000	PA14_09900	1341.772	-3.131	0.000
PA14_09910	51.561	-0.111	0.868	-0.162	0.744	0.094	0.896	PA14_09910	175.652	-0.295	0.345
PA14_09920	19.275	0.596	0.501	1.475	0.024	1.452	0.079	PA14_09920	49.950	0.579	0.224
PA14_09930	38.034	-0.715	0.308	1.104	0.031	2.341	0.000	PA14_09930	28.805	0.385	0.626
PA14_09940	157.589	-0.650	0.120	0.390	0.296	1.646	0.000	PA14_09940	149.454	-0.999	0.001
PA14_09950	41.416	1.304	0.017	2.039	0.000	0.062	0.948	PA14_09950	49.427	0.977	0.034
PA14_09960	41.591	0.523	0.433	1.918	0.000	0.505	0.521	PA14_09960	72.356	0.379	0.379
PA14_09970	117.510	-0.933	0.075	-0.866	0.064	0.940	0.104	PA14_09970	332.013	-1.969	0.000
PA14_09980	34.775	-0.223	0.770	0.369	0.511	1.009	0.121	PA14_09980	36.620	0.999	0.060
PA14_09990	77.668	0.407	0.437	1.018	0.010	-0.180	0.793	PA14_09990	220.982	0.163	0.701
PA14_10010	72.531	0.057	0.936	0.428	0.351	-1.697	0.008	PA14_10010	405.593	1.788	0.000
PA14_10020	160.418	0.087	0.901	0.276	0.574	-1.951	0.001	PA14_10020	246.070	-0.027	0.955
PA14_10040	637.804	-0.097	0.805	-0.376	0.172	-1.163	0.000	PA14_10040	864.658	0.566	0.016
PA14_10050	16.723	0.996	0.177	0.672	0.323	-0.407	0.700	PA14_10050	35.600	0.191	0.762
PA14_10070	12.360	-0.221	0.834	-0.224	0.780	-0.079	0.944	PA14_10070	44.600	0.440	0.386
PA14_10080	59.604	0.495	0.344	1.187	0.003	-0.213	0.764	PA14_10080	121.937	0.548	0.106
PA14_10090	95.930	-0.026	0.974	0.394	0.411	-0.690	0.275	PA14_10090	265.782	-0.195	0.497
PA14_10110	11.120	0.849	0.360	1.101	0.146	-0.912	0.428	PA14_10110	32.117	0.645	0.260
PA14_10120	122.627	0.052	0.930	0.174	0.676	-0.106	0.863	PA14_10120	359.137	-0.256	0.427
PA14_10130	31.466	1.208	0.041	1.852	0.000	0.411	0.630	PA14_10130	65.337	0.459	0.323
PA14_10140	6.465	1.148	0.300	1.458	0.113	0.038	0.978	PA14_10140	2.859	0.667	0.841
PA14_10160	3.802	1.027	0.441	1.034	0.356	-0.376	0.796	PA14_10160	3.868	2.574	0.317
PA14_10170	13.467	-0.136	0.921	0.492	0.602	0.391	0.749	PA14_10170	14.644	0.480	0.590
PA14_10180	51.355	-0.292	0.630	0.371	0.424	0.117	0.872	PA14_10180	37.828	1.115	0.031
PA14_10190	73.376	1.111	0.011	1.721	0.000	-0.254	0.724	PA14_10190	100.900	0.912	0.010
PA14_10200	157.161	0.341	0.441	-0.120	0.770	1.442	0.000	PA14_10200	209.876	0.183	0.554

PA14_10210	25.815	0.066	0.947	0.462	0.468	1.089	0.143	PA14_10210	24.826	1.125	0.075
PA14_10220	87.480	-0.226	0.708	1.274	0.001	0.764	0.165	PA14_10220	339.492	0.932	0.000
PA14_10230	39.133	0.780	0.185	0.059	0.927	0.572	0.430	PA14_10230	103.252	-0.018	0.998
PA14_10240	41.548	0.760	0.164	0.756	0.113	0.424	0.551	PA14_10240	90.751	0.314	0.436
PA14_10250	16.086	0.028	0.980	-0.946	0.205	-0.352	0.743	PA14_10250	41.284	-0.096	0.917
PA14_10260	56.713	1.986	0.000	2.343	0.000	2.228	0.000	PA14_10260	78.064	0.525	0.196
PA14_10270	5.867	0.876	0.483	-0.612	0.589	1.033	0.399	PA14_10270	5.634	1.240	0.405
PA14_10280	7.868	0.105	0.940	-0.558	0.570	1.028	0.341	PA14_10280	9.942	0.484	0.700
PA14_10290	466.480	0.146	0.700	0.180	0.548	3.046	0.000	PA14_10290	626.372	0.832	0.000
PA14_10300	23.306	0.426	0.614	-0.066	0.933	-0.303	0.761	PA14_10300	59.151	-0.338	0.477
PA14_10320	163.323	0.937	0.020	0.670	0.079	-0.453	0.421	PA14_10320	433.691	-0.373	0.145
PA14_10330	32.267	-2.377	0.000	-3.025	0.000	-1.101	0.092	PA14_10330	52.651	-1.379	0.002
PA14_10340	105.819	-2.295	0.000	-2.841	0.000	-0.766	0.116	PA14_10340	170.781	-1.327	0.000
PA14_10350	154.231	-2.281	0.000	-4.048	0.000	0.397	0.427	PA14_10350	310.718	-1.944	0.000
PA14_10360	3043.288	-3.628	0.000	-5.738	0.000	1.148	0.017	PA14_10360	10552.019	-3.537	0.000
PA14_10370	168.481	-2.748	0.000	-2.907	0.000	0.711	0.098	PA14_10370	124.689	-1.320	0.003
PA14_10380	163.169	-3.629	0.000	-4.291	0.000	0.383	0.550	PA14_10380	322.048	-2.574	0.000
PA14_10400	19.657	-0.508	0.535	-2.332	0.001	-0.375	0.692	PA14_10400	18.330	-0.635	0.449
PA14_10410	12.527	-0.713	0.421	-3.006	0.000	-0.285	0.789	PA14_10410	10.686	-2.082	0.033
PA14_10420	99.279	1.065	0.016	2.251	0.000	1.401	0.005	PA14_10420	104.055	0.689	0.051
PA14_10440	44.151	0.832	0.095	-0.635	0.195	0.712	0.240	PA14_10440	61.867	-0.412	0.345
PA14_10470	30.917	0.733	0.213	-0.161	0.791	-0.512	0.528	PA14_10470	64.893	-0.045	0.926
PA14_10480	149.005	0.376	0.320	-0.348	0.295	-0.357	0.443	PA14_10480	281.400	0.467	0.100
PA14_10490	64.724	-2.590	0.000	-3.895	0.000	-3.418	0.000	PA14_10490	234.531	-5.492	0.000
PA14_10500	5632.269	-3.251	0.000	-6.441	0.000	-4.201	0.000	PA14_10500	15673.100	-5.005	0.000
PA14_10530	2248.564	-3.238	0.000	-5.882	0.000	-5.814	0.000	PA14_10530	3205.250	-4.089	0.000
PA14_10540	3895.828	-3.518	0.000	-7.210	0.000	-5.505	0.000	PA14_10540	5085.481	-6.043	0.000
PA14_10550	2434.099	-1.959	0.000	-5.716	0.000	-3.828	0.000	PA14_10550	5700.087	-5.790	0.000
PA14_10560	726.137	-1.781	0.000	-4.093	0.000	-3.470	0.000	PA14_10560	1404.331	-6.215	0.000
PA14_10570	18.715	0.653	0.383	-1.053	0.113	-0.312	0.752	PA14_10570	49.749	-1.926	0.000
PA14_10590	46.679	2.237	0.000	-0.260	0.658	-0.938	0.239	PA14_10590	55.220	-0.224	0.671
PA14_10600	60.633	1.075	0.014	-0.149	0.767	0.727	0.196	PA14_10600	91.032	0.125	0.812
PA14_10610	52.380	2.233	0.000	0.977	0.035	1.178	0.047	PA14_10610	112.858	-0.568	0.128
PA14_10620	84.005	1.443	0.000	-0.318	0.455	-0.480	0.416	PA14_10620	169.506	0.007	0.946
PA14_10630	146.240	2.617	0.000	0.773	0.033	-0.705	0.213	PA14_10630	224.880	-0.860	0.002
PA14_10640	105.243	2.906	0.000	0.895	0.016	-0.382	0.554	PA14_10640	98.105	-0.236	0.581
PA14_10650	87.973	3.114	0.000	1.769	0.000	1.003	0.062	PA14_10650	68.000	0.030	0.931
PA14_10660	23.982	1.970	0.003	1.303	0.047	0.948	0.290	PA14_10660	81.899	-1.290	0.000
PA14_10670	196.420	1.361	0.000	2.087	0.000	1.302	0.005	PA14_10670	229.878	0.580	0.091
PA14_10680	186.069	-0.023	0.973	0.010	0.982	-0.974	0.055	PA14_10680	665.746	0.591	0.014
PA14_10700	1769.144	-1.125	0.000	-1.353	0.000	-0.544	0.083	PA14_10700	8164.587	-1.261	0.000
PA14_10710	1066.411	-0.366	0.246	0.184	0.536	1.023	0.001	PA14_10710	3554.673	-0.836	0.000
PA14_10730	3280.781	0.880	0.000	-0.050	0.864	-0.005	0.990	PA14_10730	14611.230	0.240	0.419
PA14_10740	298.800	0.540	0.096	0.783	0.005	0.856	0.018	PA14_10740	771.098	-0.709	0.002
PA14_10750	144.050	0.259	0.620	1.788	0.000	1.793	0.000	PA14_10750	276.538	0.114	0.731
PA14_10770	478.392	-1.577	0.000	-1.917	0.000	0.549	0.115	PA14_10770	1502.215	-2.129	0.000
PA14_10780	48.027	-0.743	0.156	-1.745	0.000	-0.959	0.123	PA14_10780	352.498	-1.367	0.000
PA14_10790	118.462	0.357	0.405	-0.428	0.241	0.587	0.199	PA14_10790	230.587	0.659	0.017
PA14_10800	228.770	1.084	0.000	1.268	0.000	-0.263	0.590	PA14_10800	474.566	0.350	0.162
PA14_10820	404.001	-1.200	0.000	-1.363	0.000	0.371	0.387	PA14_10820	1954.545	-2.850	0.000
PA14_10830	253.457	0.146	0.730	-0.122	0.726	0.246	0.581	PA14_10830	693.342	-0.015	0.998
PA14_10840	76.834	0.612	0.238	0.879	0.042	-0.432	0.524	PA14_10840	187.339	0.478	0.131
PA14_10850	9.987	-0.318	0.785	-0.122	0.899	-0.178	0.887	PA14_10850	29.882	0.204	0.752
PA14_10870	23.792	0.300	0.705	0.133	0.843	0.801	0.283	PA14_10870	51.832	0.288	0.571
PA14_10890	5.732	0.286	0.836	0.327	0.757	0.758	0.532	PA14_10890	8.236	1.985	0.097
PA14_10900	3.671	0.238	0.883	0.105	0.935	-1.051	0.415	PA14_10900	5.091	1.439	0.456
PA14_10910	17.856	-0.033	0.976	0.087	0.912	1.091	0.172	PA14_10910	52.779	-0.934	0.071
PA14_10920	8.741	-0.451	0.705	-1.748	0.050	-0.478	0.697	PA14_10920	23.379	0.491	0.603
PA14_10940	152.658	1.105	0.001	0.122	0.752	0.673	0.123	PA14_10940	391.246	0.174	0.566

PA14_10950	15.242	-0.155	0.887	-0.945	0.206	-0.387	0.717	PA14_10950	34.221	0.701	0.220
PA14_10960	96.975	1.944	0.000	-0.580	0.216	-0.182	0.800	PA14_10960	253.759	-0.057	0.914
PA14_10970	24.897	0.827	0.190	-0.998	0.095	0.333	0.696	PA14_10970	99.732	-1.401	0.000
PA14_10980	187.741	1.393	0.000	1.502	0.000	-0.435	0.472	PA14_10980	388.678	1.966	0.000
PA14_10990	13.118	3.075	0.000	2.435	0.002	2.634	0.005	PA14_10990	12.636	1.035	0.276
PA14_11000	138.223	2.111	0.000	1.150	0.000	1.412	0.000	PA14_11000	156.420	-0.301	0.370
PA14_11010	584.600	0.505	0.251	0.293	0.473	0.165	0.776	PA14_11010	1043.131	-0.966	0.022
PA14_11020	2.842	0.840	0.573	0.382	0.774	2.436	0.043	PA14_11020	11.002	0.571	0.629
PA14_11030	8.530	0.223	0.859	0.202	0.836	0.661	0.555	PA14_11030	25.310	1.233	0.048
PA14_11050	12.973	0.695	0.450	-0.422	0.617	0.745	0.452	PA14_11050	32.346	0.446	0.466
PA14_11060	59.763	0.408	0.436	0.784	0.051	-0.517	0.426	PA14_11060	112.335	-0.002	1.000
PA14_11070	19.957	-0.104	0.913	-0.941	0.131	-0.241	0.798	PA14_11070	53.894	0.640	0.172
PA14_11080	32.763	-0.047	0.959	0.186	0.760	1.845	0.002	PA14_11080	61.205	-0.660	0.123
PA14_11090	17.860	-0.350	0.705	-0.258	0.730	1.403	0.067	PA14_11090	14.883	-0.580	0.489
PA14_11100	188.365	-0.075	0.882	-0.080	0.835	0.334	0.468	PA14_11100	292.952	-0.362	0.204
PA14_11110	26.205	0.350	0.632	-0.056	0.933	0.600	0.425	PA14_11110	78.904	-0.402	0.371
PA14_11120	80.674	-0.928	0.049	-1.333	0.001	-1.804	0.001	PA14_11120	555.296	-0.720	0.002
PA14_11130	83.536	-0.853	0.103	-0.994	0.031	-1.793	0.003	PA14_11130	393.565	-0.602	0.021
PA14_11140	164.481	-2.992	0.000	-3.331	0.000	-1.152	0.004	PA14_11140	875.716	-3.081	0.000
PA14_11150	6.594	1.016	0.358	1.000	0.287	0.196	0.889	PA14_11150	36.672	0.405	0.474
PA14_11160	17.933	-0.112	0.914	0.731	0.256	-1.371	0.156	PA14_11160	64.728	0.569	0.202
PA14_11170	315.685	0.856	0.004	1.251	0.000	-0.417	0.330	PA14_11170	516.044	-0.186	0.467
PA14_11180	34.791	-0.336	0.634	-0.232	0.691	-0.168	0.842	PA14_11180	71.746	-0.015	0.957
PA14_11190	70.793	0.978	0.026	1.530	0.000	0.099	0.891	PA14_11190	136.887	0.026	0.953
PA14_11210	19.024	-0.275	0.754	-0.162	0.820	-0.574	0.534	PA14_11210	39.933	0.284	0.640
PA14_11230	3.643	-0.151	0.928	-0.184	0.885	0.844	0.521	PA14_11230	5.149	1.430	0.366
PA14_11240	55.704	1.268	0.005	0.259	0.600	0.622	0.309	PA14_11240	132.489	-0.687	0.031
PA14_11250	330.480	0.431	0.162	0.289	0.311	-0.066	0.884	PA14_11250	747.022	1.069	0.000
PA14_11260	354.848	-0.084	0.838	-0.215	0.471	-1.578	0.000	PA14_11260	578.025	1.249	0.000
PA14_11270	9011.334	-1.164	0.000	-2.604	0.000	-3.856	0.000	PA14_11270	50899.883	-0.157	0.741
PA14_11280	374.239	-0.589	0.099	-1.011	0.001	-1.471	0.000	PA14_11280	322.105	0.679	0.009
PA14_11290	231.543	-0.086	0.884	0.039	0.932	0.222	0.699	PA14_11290	219.565	0.871	0.002
PA14_11310	292.006	0.563	0.176	1.022	0.003	0.276	0.624	PA14_11310	382.380	-0.010	0.991
PA14_11320	3266.881	0.651	0.101	1.611	0.000	0.853	0.059	PA14_11320	2624.709	-1.185	0.000
PA14_11330	74.099	-0.571	0.271	-0.286	0.541	-0.475	0.454	PA14_11330	156.814	-0.576	0.057
PA14_11340	1905.812	0.172	0.617	0.056	0.856	-1.458	0.000	PA14_11340	4443.885	1.526	0.000
PA14_11350	111.030	0.058	0.918	0.069	0.870	-1.239	0.012	PA14_11350	298.734	0.383	0.159
PA14_11370	76.748	0.278	0.580	0.175	0.684	-1.165	0.035	PA14_11370	228.233	0.311	0.316
PA14_11380	362.574	0.626	0.051	0.951	0.001	0.261	0.548	PA14_11380	888.752	0.481	0.035
PA14_11400	259.328	-0.397	0.291	-0.299	0.371	-0.710	0.088	PA14_11400	513.882	0.254	0.359
PA14_11410	1104.934	0.854	0.007	1.482	0.000	1.053	0.004	PA14_11410	997.337	1.419	0.000
PA14_11420	1226.105	0.279	0.386	0.583	0.024	0.014	0.973	PA14_11420	1780.941	1.440	0.000
PA14_11430	2043.939	0.659	0.077	0.639	0.061	1.147	0.005	PA14_11430	2496.296	1.386	0.000
PA14_11450	413.342	1.318	0.000	1.396	0.000	0.773	0.033	PA14_11450	772.391	1.133	0.000
PA14_11460	347.078	0.622	0.047	1.245	0.000	0.898	0.011	PA14_11460	379.241	1.760	0.000
PA14_11470	257.789	0.771	0.011	1.192	0.000	0.040	0.935	PA14_11470	569.195	0.322	0.227
PA14_11480	372.057	0.003	0.995	0.587	0.054	0.823	0.031	PA14_11480	1234.378	-1.360	0.000
PA14_11490	115.550	-0.508	0.226	-0.207	0.596	-0.035	0.952	PA14_11490	467.518	-1.521	0.000
PA14_11510	386.036	0.105	0.863	0.099	0.836	0.195	0.750	PA14_11510	1328.600	0.687	0.002
PA14_11520	48.736	0.773	0.228	1.338	0.010	1.086	0.116	PA14_11520	192.187	1.841	0.000
PA14_11530	249.464	0.906	0.006	1.724	0.000	1.323	0.000	PA14_11530	458.548	2.178	0.000
PA14_11550	2160.762	0.880	0.000	1.123	0.000	0.615	0.049	PA14_11550	3926.594	1.131	0.000
PA14_11560	220.649	0.773	0.011	0.974	0.000	-0.819	0.046	PA14_11560	477.862	1.143	0.000
PA14_11570	596.360	1.396	0.000	1.786	0.000	-0.200	0.700	PA14_11570	1162.524	1.038	0.003
PA14_11580	108.200	-0.988	0.012	-1.382	0.000	0.379	0.452	PA14_11580	536.286	-2.044	0.000
PA14_11590	9.767	1.127	0.238	1.182	0.150	-0.177	0.895	PA14_11590	32.733	-0.108	0.901
PA14_11600	51.353	-0.306	0.640	-0.128	0.820	0.345	0.623	PA14_11600	87.113	0.322	0.394
PA14_11610	10.472	-0.072	0.960	0.931	0.277	0.932	0.386	PA14_11610	19.765	0.801	0.482
PA14_11620	40.818	-0.242	0.725	-0.933	0.061	-0.679	0.334	PA14_11620	207.069	-2.102	0.000

PA14_11630	80.207	-0.208	0.682	-1.208	0.001	-1.163	0.021	PA14_11630	198.235	-0.551	0.192
PA14_11650	739.151	0.658	0.138	0.717	0.070	-0.675	0.197	PA14_11650	955.998	0.058	0.898
PA14_11660	34.335	-0.450	0.479	0.015	0.980	0.239	0.748	PA14_11660	96.966	-1.633	0.000
PA14_11670	79.132	-0.714	0.129	-0.799	0.051	1.208	0.012	PA14_11670	116.850	-1.082	0.001
PA14_11680	521.374	0.459	0.111	0.715	0.004	-0.814	0.016	PA14_11680	1947.027	-0.673	0.002
PA14_11690	1561.494	-0.405	0.281	-0.268	0.436	-1.402	0.000	PA14_11690	2054.257	1.603	0.000
PA14_11700	349.202	0.780	0.013	1.367	0.000	1.668	0.000	PA14_11700	471.984	1.698	0.000
PA14_11720	265.948	-0.291	0.458	0.846	0.004	1.339	0.000	PA14_11720	717.663	1.150	0.000
PA14_11730	62.017	0.888	0.130	1.612	0.001	0.292	0.718	PA14_11730	172.901	0.353	0.294
PA14_11740	185.031	-0.868	0.014	-1.003	0.002	0.639	0.128	PA14_11740	1525.237	-2.115	0.000
PA14_11750	408.603	-0.098	0.818	-0.416	0.156	-1.119	0.002	PA14_11750	1791.012	-0.761	0.000
PA14_11760	166.604	0.548	0.112	-3.665	0.000	-1.718	0.000	PA14_11760	221.902	-1.595	0.000
PA14_11770	763.201	1.128	0.000	-3.227	0.000	-1.355	0.000	PA14_11770	784.072	-1.667	0.000
PA14_11790	489.742	1.297	0.001	-2.303	0.000	-1.517	0.001	PA14_11790	559.765	-1.949	0.000
PA14_11810	1515.177	0.213	0.523	-6.646	0.000	-3.771	0.000	PA14_11810	935.423	-1.743	0.000
PA14_11830	2069.811	0.246	0.540	-0.116	0.747	0.161	0.735	PA14_11830	2767.015	0.017	0.939
PA14_11845	612.176	0.456	0.277	0.251	0.520	-0.772	0.094	PA14_11845	1772.795	0.052	0.814
PA14_11860	143.316	0.374	0.357	0.651	0.045	-0.313	0.537	PA14_11860	392.665	-0.059	0.846
PA14_11880	22.300	0.588	0.484	0.568	0.416	0.906	0.291	PA14_11880	63.961	0.209	0.671
PA14_11890	472.963	-0.892	0.002	-1.610	0.000	0.386	0.301	PA14_11890	3548.185	-3.053	0.000
PA14_11900	1376.831	0.453	0.270	0.092	0.825	0.084	0.880	PA14_11900	3651.279	-1.396	0.000
PA14_11910	476.006	-2.151	0.000	-2.950	0.000	-1.655	0.000	PA14_11910	1104.561	-1.156	0.000
PA14_11920	81.380	0.631	0.180	0.881	0.027	-0.246	0.714	PA14_11920	235.803	1.617	0.000
PA14_11930	114.669	0.583	0.132	0.612	0.071	-0.599	0.240	PA14_11930	551.220	-0.772	0.001
PA14_11940	266.622	-0.226	0.613	-0.501	0.142	-0.177	0.732	PA14_11940	1271.815	-1.511	0.000
PA14_11960	247.957	-1.559	0.000	-0.576	0.064	-1.917	0.000	PA14_11960	734.769	0.644	0.006
PA14_11970	360.934	-0.414	0.323	0.876	0.009	0.282	0.575	PA14_11970	1627.753	0.083	0.735
PA14_11980	105.070	0.918	0.031	1.662	0.000	0.711	0.180	PA14_11980	256.364	0.014	1.000
PA14_11990	40.549	0.293	0.683	0.630	0.234	0.304	0.700	PA14_11990	117.014	-0.465	0.408
PA14_12010	1875.945	0.787	0.017	0.796	0.010	1.334	0.000	PA14_12010	2465.094	1.440	0.000
PA14_12020	704.028	0.739	0.013	0.886	0.001	1.531	0.000	PA14_12020	934.031	1.389	0.000
PA14_12030	1060.527	0.474	0.103	0.906	0.000	0.908	0.004	PA14_12030	1495.613	-0.260	0.267
PA14_12050	793.344	0.447	0.215	1.030	0.001	1.502	0.000	PA14_12050	749.865	1.161	0.000
PA14_12060	815.084	0.553	0.055	0.967	0.000	0.796	0.015	PA14_12060	971.919	1.654	0.000
PA14_12070	3725.939	0.771	0.029	1.777	0.000	1.063	0.009	PA14_12070	5217.500	0.831	0.000
PA14_12080	1425.228	0.737	0.046	1.458	0.000	0.301	0.541	PA14_12080	2080.223	0.634	0.004
PA14_12090	9293.926	0.147	0.802	0.185	0.688	0.420	0.456	PA14_12090	8469.515	0.191	0.408
PA14_12100	3834.152	-0.148	0.622	-0.327	0.158	-0.705	0.012	PA14_12100	6370.332	0.031	0.911
PA14_12110	604.888	0.217	0.627	-0.058	0.888	-1.001	0.018	PA14_12110	1871.448	0.385	0.094
PA14_12120	3318.644	0.012	0.976	-0.102	0.716	-0.137	0.712	PA14_12120	7230.233	0.262	0.266
PA14_12130	753.829	-0.271	0.467	-0.603	0.039	-0.788	0.034	PA14_12130	3149.737	-0.087	0.706
PA14_12140	196.486	-0.321	0.405	-0.194	0.567	1.215	0.001	PA14_12140	499.350	-1.301	0.000
PA14_12150	29.124	0.655	0.357	1.820	0.000	0.569	0.498	PA14_12150	87.318	-0.124	0.728
PA14_12160	864.158	0.309	0.290	0.666	0.005	0.450	0.164	PA14_12160	1634.081	0.607	0.005
PA14_12170	1.390	0.522	0.749	1.742	0.148	1.623	0.182	PA14_12170	2.023	1.400	0.660
PA14_12180	25.573	0.290	0.721	1.238	0.023	0.578	0.478	PA14_12180	115.704	-0.973	0.003
PA14_12200	742.278	0.733	0.022	0.553	0.067	-0.212	0.641	PA14_12200	885.482	0.651	0.005
PA14_12210	1519.019	-0.106	0.782	-0.640	0.015	-0.424	0.236	PA14_12210	1657.645	0.678	0.002
PA14_12230	4189.301	0.460	0.092	0.641	0.008	0.147	0.697	PA14_12230	5634.584	1.169	0.000
PA14_12260	389.480	-1.958	0.000	-3.117	0.000	1.111	0.028	PA14_12260	3676.274	-4.623	0.000
PA14_12270	239.057	0.385	0.391	1.613	0.000	1.728	0.000	PA14_12270	443.317	0.681	0.063
PA14_12280	197.498	0.034	0.944	0.604	0.036	-1.000	0.012	PA14_12280	346.837	0.779	0.002
PA14_12300	1391.286	0.594	0.025	1.199	0.000	0.402	0.229	PA14_12300	2197.584	1.407	0.000
PA14_12310	4502.207	0.991	0.001	1.297	0.000	0.836	0.021	PA14_12310	6219.027	0.689	0.001
PA14_12330	6358.530	0.518	0.076	0.556	0.036	0.407	0.257	PA14_12330	16204.607	-0.010	0.997
PA14_12350	3158.766	1.195	0.000	2.111	0.000	2.214	0.000	PA14_12350	2384.773	1.430	0.000
PA14_12360	1644.527	0.674	0.036	2.682	0.000	2.294	0.000	PA14_12360	1640.459	1.677	0.000
PA14_12370	4147.885	0.319	0.420	0.664	0.036	-0.043	0.935	PA14_12370	7141.652	-0.337	0.136
PA14_12390	728.698	0.319	0.378	-0.417	0.172	-0.636	0.100	PA14_12390	834.985	0.889	0.000

PA14_12400	418.724	1.242	0.000	0.897	0.004	0.995	0.012	PA14_12400	532.643	0.753	0.002
PA14_12410	899.284	0.990	0.001	1.461	0.000	0.108	0.818	PA14_12410	1599.442	0.677	0.006
PA14_12420	929.523	0.400	0.189	0.050	0.876	1.330	0.000	#N/A	#N/A	#N/A	#N/A
PA14_12430	353.032	1.376	0.000	2.196	0.000	3.014	0.000	#N/A	#N/A	#N/A	#N/A
PA14_12440	329.297	1.041	0.001	-0.361	0.264	2.786	0.000	PA14_12440	1248.922	-2.152	0.000
PA14_12450	1135.924	0.760	0.009	-0.237	0.437	3.005	0.000	PA14_12450	2752.106	-0.407	0.064
PA14_12470	252.385	0.534	0.142	-0.143	0.700	3.097	0.000	PA14_12470	656.160	-0.711	0.002
PA14_12490	411.599	-0.376	0.274	-0.822	0.003	-0.260	0.535	PA14_12490	1390.434	-0.169	0.487
PA14_12530	305.367	-0.256	0.544	-0.579	0.075	0.753	0.063	PA14_12530	2400.385	-0.783	0.000
PA14_12540	46.138	0.863	0.101	1.347	0.002	-0.108	0.896	PA14_12540	87.318	0.618	0.110
PA14_12550	117.844	0.275	0.681	0.024	0.969	-0.815	0.221	PA14_12550	238.602	2.053	0.000
PA14_12560	430.970	0.434	0.241	1.210	0.000	0.552	0.186	PA14_12560	1184.391	0.809	0.000
PA14_12570	589.460	0.424	0.171	-0.272	0.349	0.031	0.945	PA14_12570	1363.472	-0.128	0.690
PA14_12590	27.351	0.591	0.377	0.788	0.145	0.817	0.257	PA14_12590	87.166	-1.144	0.001
PA14_12610	50.616	0.102	0.889	-0.071	0.900	-0.573	0.408	PA14_12610	155.490	0.306	0.357
PA14_12620	567.940	-1.189	0.000	-0.974	0.002	0.203	0.672	PA14_12620	2547.573	-0.785	0.000
PA14_12630	549.778	0.496	0.098	1.052	0.000	0.422	0.248	PA14_12630	741.385	0.412	0.097
PA14_12640	22.965	0.606	0.417	-0.123	0.866	-2.847	0.003	PA14_12640	32.910	1.193	0.029
PA14_12650	88.508	0.441	0.343	0.714	0.057	-0.019	0.977	PA14_12650	215.364	0.091	0.787
PA14_12670	341.674	0.424	0.337	0.724	0.046	0.152	0.792	PA14_12670	632.575	0.574	0.018
PA14_12680	105.348	-1.776	0.000	-1.010	0.007	1.564	0.000	PA14_12680	472.735	-2.189	0.000
PA14_12690	76.889	-0.853	0.063	-0.657	0.112	-0.804	0.151	PA14_12690	243.343	-0.493	0.088
PA14_12700	260.808	0.926	0.004	1.605	0.000	-0.742	0.096	PA14_12700	382.300	1.603	0.000
PA14_12710	81.182	0.297	0.528	-0.205	0.617	-0.304	0.591	PA14_12710	154.788	0.471	0.153
PA14_12730	76.056	0.601	0.205	0.547	0.191	0.327	0.597	PA14_12730	190.612	0.900	0.002
PA14_12740	156.922	-0.829	0.047	-0.009	0.984	-1.765	0.000	PA14_12740	724.550	1.117	0.000
PA14_12750	349.092	0.482	0.164	1.523	0.000	1.308	0.000	PA14_12750	841.631	0.794	0.001
PA14_12760	519.912	0.897	0.001	1.285	0.000	0.478	0.174	PA14_12760	872.162	0.964	0.000
PA14_12770	103.599	0.115	0.835	0.495	0.181	-0.295	0.602	PA14_12770	277.562	0.973	0.008
PA14_12780	497.984	0.461	0.125	0.029	0.930	-0.310	0.422	PA14_12780	1565.434	0.711	0.001
PA14_12810	183.435	-0.317	0.491	-0.763	0.033	-1.144	0.014	PA14_12810	672.398	-0.638	0.006
PA14_12820	144.701	0.276	0.483	0.178	0.602	-0.527	0.236	PA14_12820	412.946	0.395	0.125
PA14_12840	552.438	-1.221	0.000	-0.867	0.000	0.787	0.011	PA14_12840	3741.642	-2.101	0.000
PA14_12850	435.181	-0.489	0.166	0.194	0.567	0.398	0.343	PA14_12850	1841.439	-1.329	0.000
PA14_12860	275.498	0.098	0.822	-0.234	0.460	0.347	0.393	PA14_12860	730.748	-0.256	0.297
PA14_12870	859.929	0.862	0.002	0.980	0.000	0.347	0.364	PA14_12870	1542.147	0.199	0.380
PA14_12890	30.643	1.112	0.065	0.878	0.113	-0.107	0.912	PA14_12890	117.963	0.334	0.334
PA14_12900	984.601	-0.397	0.712	-0.751	0.368	-2.374	0.008	PA14_12900	3107.365	0.237	0.389
PA14_12910	23.643	0.149	0.876	0.645	0.305	-0.151	0.885	PA14_12910	31.849	0.684	0.237
PA14_12920	161.037	-2.242	0.000	-0.981	0.006	-2.047	0.000	PA14_12920	146.733	0.324	0.346
PA14_12940	37.272	-2.599	0.000	-0.540	0.249	-2.047	0.002	PA14_12940	29.541	1.099	0.209
PA14_12960	93.545	-2.130	0.000	-0.904	0.036	-2.071	0.000	PA14_12960	56.652	1.467	0.001
PA14_12970	88.922	-1.481	0.000	-0.282	0.475	-1.316	0.007	PA14_12970	140.898	-0.331	0.337
PA14_12980	755.220	-0.489	0.122	-2.164	0.000	-2.847	0.000	PA14_12980	708.455	-1.253	0.000
PA14_12990	1720.754	1.068	0.000	2.803	0.000	4.977	0.000	PA14_12990	453.022	1.479	0.000
PA14_13000	34.376	-2.706	0.000	-2.213	0.000	-0.353	0.667	PA14_13000	53.507	-0.950	0.032
PA14_13010	260.875	-2.887	0.000	-4.139	0.000	-0.666	0.245	PA14_13010	172.248	-1.803	0.000
PA14_13030	1086.300	-2.770	0.000	-2.800	0.000	1.637	0.000	PA14_13030	1296.135	-1.482	0.000
PA14_13040	379.241	-2.238	0.000	-2.117	0.000	1.059	0.003	PA14_13040	771.875	-1.621	0.000
PA14_13050	5.293	-1.240	0.282	-3.079	0.003	0.213	0.876	PA14_13050	27.541	-2.456	0.000
PA14_13060	77.895	0.086	0.899	-0.278	0.561	-0.329	0.619	PA14_13060	350.938	0.941	0.000
PA14_13070	17.117	0.020	0.985	0.359	0.610	0.818	0.332	PA14_13070	46.344	0.579	0.247
PA14_13090	371.379	1.200	0.000	0.001	0.998	-0.112	0.811	PA14_13090	1501.972	0.514	0.019
PA14_13110	359.137	-1.138	0.000	-2.423	0.000	-0.431	0.252	PA14_13110	644.026	-1.231	0.000
PA14_13130	1439.231	-0.572	0.038	-4.259	0.000	-0.466	0.167	PA14_13130	1597.030	-1.107	0.000
PA14_13140	750.440	-0.472	0.126	-4.480	0.000	-1.091	0.001	PA14_13140	1504.167	-1.745	0.000
PA14_13150	453.304	-0.663	0.056	-2.146	0.000	0.515	0.217	PA14_13150	882.395	-0.932	0.000
PA14_13170	3440.795	-0.024	0.954	-1.382	0.000	-0.244	0.496	PA14_13170	16236.686	-1.873	0.000
PA14_13190	1710.059	-1.504	0.000	-2.963	0.000	-0.299	0.391	PA14_13190	24189.179	-3.192	0.000

PA14_13200	242.635	0.149	0.767	0.369	0.320	1.675	0.000	PA14_13200	1185.503	-0.223	0.366
PA14_13210	32.279	-2.953	0.000	-3.614	0.000	-0.535	0.447	PA14_13210	297.415	-5.603	0.000
PA14_13220	47.792	-0.289	0.683	-0.657	0.216	0.230	0.766	PA14_13220	556.213	-3.546	0.000
PA14_13230	974.924	0.163	0.656	-0.225	0.444	0.266	0.493	PA14_13230	3072.123	-1.192	0.000
PA14_13240	69.774	-0.865	0.061	-0.806	0.049	-0.301	0.625	PA14_13240	466.542	-0.709	0.004
PA14_13250	216.311	-0.034	0.942	-0.451	0.132	-0.244	0.579	PA14_13250	704.496	-0.782	0.001
PA14_13260	68.962	-2.214	0.000	-2.707	0.000	-2.409	0.000	PA14_13260	166.901	-2.158	0.000
PA14_13280	28.578	-1.873	0.001	-2.124	0.000	-1.713	0.017	PA14_13280	236.964	-3.217	0.000
PA14_13290	58.526	-0.735	0.107	-2.230	0.000	-0.216	0.727	PA14_13290	478.975	-0.140	0.623
PA14_13300	59.142	-1.030	0.020	-1.727	0.000	-0.234	0.706	PA14_13300	291.553	-0.121	0.667
PA14_13320	52.327	-0.380	0.499	-1.346	0.002	-0.047	0.947	PA14_13320	179.236	-0.150	0.631
PA14_13330	61.121	-0.229	0.696	0.451	0.285	0.949	0.063	PA14_13330	408.755	-1.737	0.000
PA14_13340	58.071	-0.952	0.039	-0.736	0.075	-0.328	0.596	PA14_13340	478.258	-3.589	0.000
PA14_13350	422.891	-1.364	0.000	-2.383	0.000	-3.815	0.000	PA14_13350	1036.652	-0.704	0.007
PA14_13360	1647.685	-1.225	0.000	-2.217	0.000	-3.463	0.000	PA14_13360	2702.994	-0.537	0.114
PA14_13370	165.685	-1.262	0.000	-2.010	0.000	-2.780	0.000	PA14_13370	603.301	-0.245	0.312
PA14_13380	234.460	-1.375	0.000	-1.593	0.000	-2.342	0.000	PA14_13380	720.231	0.358	0.209
PA14_13390	384.256	-0.819	0.055	-1.486	0.000	-3.078	0.000	PA14_13390	2549.469	0.182	0.474
PA14_13410	1000.691	0.493	0.066	0.457	0.064	-0.243	0.496	PA14_13410	1256.542	1.289	0.000
PA14_13420	718.308	-0.152	0.695	0.167	0.592	-1.309	0.000	PA14_13420	2296.215	0.070	0.866
PA14_13430	658.983	1.166	0.000	1.686	0.000	1.067	0.007	PA14_13430	209.610	1.170	0.000
PA14_13450	34.525	-0.660	0.364	-0.546	0.373	0.876	0.222	PA14_13450	26.334	-0.991	0.116
PA14_13460	125.388	0.239	0.664	0.799	0.041	1.696	0.000	PA14_13460	87.964	-0.445	0.430
PA14_13470	296.424	0.688	0.023	0.606	0.030	0.607	0.101	PA14_13470	741.263	0.130	0.632
PA14_13490	18.802	0.715	0.329	0.366	0.586	-0.635	0.503	PA14_13490	33.130	-0.363	0.553
PA14_13500	173.645	0.331	0.574	-0.137	0.795	-1.242	0.035	PA14_13500	312.765	0.911	0.000
PA14_13510	754.239	1.689	0.000	1.720	0.000	0.944	0.010	PA14_13510	1176.442	-0.342	0.355
PA14_13520	440.299	1.146	0.000	2.160	0.000	-1.491	0.000	PA14_13520	210.402	0.804	0.005
PA14_13530	273.264	0.719	0.015	1.572	0.000	-2.060	0.000	PA14_13530	74.374	1.059	0.007
PA14_13560	262.329	0.285	0.526	0.716	0.038	-3.300	0.000	PA14_13560	125.202	0.494	0.159
PA14_13580	696.593	-0.865	0.002	-1.578	0.000	-0.824	0.018	PA14_13580	1455.712	-0.212	0.401
PA14_13590	612.470	-0.496	0.130	-0.350	0.247	0.712	0.050	PA14_13590	996.517	-0.119	0.696
PA14_13600	333.161	-0.893	0.006	-0.720	0.017	0.652	0.093	PA14_13600	754.193	-0.233	0.355
PA14_13610	156.080	-0.793	0.044	-1.082	0.002	0.283	0.585	PA14_13610	442.337	-0.695	0.005
PA14_13620	209.967	1.133	0.000	1.571	0.000	1.025	0.010	PA14_13620	265.489	1.844	0.000
PA14_13630	43.574	-1.140	0.029	-1.305	0.005	0.156	0.832	PA14_13630	169.354	-1.579	0.000
PA14_13650	170.336	0.436	0.332	0.245	0.552	-0.244	0.671	PA14_13650	279.752	0.742	0.005
PA14_13660	128.502	-0.621	0.471	-0.020	0.980	6.762	0.000	PA14_13660	22.072	1.974	0.003
PA14_13670	13.656	0.431	0.656	1.413	0.035	0.292	0.793	PA14_13670	48.332	0.443	0.409
PA14_13680	60.941	0.189	0.779	0.361	0.472	-0.564	0.414	PA14_13680	149.030	0.600	0.063
PA14_13690	105.607	0.062	0.916	-0.991	0.006	-0.449	0.397	PA14_13690	613.558	0.430	0.123
PA14_13710	137.403	0.110	0.835	-0.128	0.752	0.326	0.522	PA14_13710	724.756	0.066	0.846
PA14_13720	793.583	-0.341	0.585	-1.039	0.028	-1.174	0.049	PA14_13720	2263.509	-1.415	0.000
PA14_13730	395.962	-1.074	0.004	-2.363	0.000	-3.468	0.000	PA14_13730	2957.332	-0.688	0.175
PA14_13740	1024.785	-1.227	0.000	-1.594	0.000	-2.294	0.000	PA14_13740	5287.093	-0.123	0.624
PA14_13750	553.467	-1.690	0.000	-4.721	0.000	-4.697	0.000	PA14_13750	518.420	-0.984	0.001
PA14_13770	140.192	-1.146	0.001	-5.192	0.000	-3.846	0.000	PA14_13770	1130.786	-1.509	0.000
PA14_13780	545.663	-0.779	0.011	-3.294	0.000	-2.975	0.000	PA14_13780	14301.201	-2.195	0.000
PA14_13800	252.723	-0.271	0.507	-1.532	0.000	-1.229	0.002	PA14_13800	7734.876	-2.096	0.000
PA14_13810	22.006	-0.621	0.362	-1.571	0.005	-1.189	0.121	PA14_13810	745.430	-2.137	0.000
PA14_13830	71.301	-0.724	0.090	-1.723	0.000	-0.938	0.067	PA14_13830	1462.483	-2.011	0.000
PA14_13840	23.427	-0.504	0.484	-1.588	0.005	-2.408	0.004	PA14_13840	588.722	-1.833	0.000
PA14_13850	19.459	-0.059	0.952	-0.543	0.401	-0.349	0.707	PA14_13850	367.726	-1.531	0.000
PA14_13860	21.199	-0.134	0.887	-0.469	0.475	0.068	0.944	PA14_13860	95.120	0.744	0.040
PA14_13870	339.169	1.023	0.001	1.527	0.000	1.313	0.000	PA14_13870	815.590	0.081	0.790
PA14_13880	29.068	-0.086	0.920	-0.341	0.560	-0.139	0.874	PA14_13880	85.832	-0.061	0.876
PA14_13890	140.895	0.324	0.449	0.903	0.005	1.251	0.002	PA14_13890	500.097	-0.433	0.081
PA14_13900	273.762	0.378	0.237	0.071	0.827	0.126	0.770	#N/A	#N/A	#N/A	#N/A
PA14_13910	175.867	0.826	0.067	0.993	0.013	0.133	0.843	#N/A	#N/A	#N/A	#N/A

PA14_13920	72.160	1.274	0.002	1.122	0.004	1.885	0.000
PA14_13940	2302.194	0.267	0.579	0.111	0.798	0.107	0.855
PA14_13950	4006.462	0.184	0.707	-0.089	0.834	-0.235	0.659
PA14_13960	16.483	-0.440	0.656	-0.811	0.289	0.955	0.290
PA14_13970	214.060	-0.997	0.035	-0.897	0.039	-0.177	0.793
PA14_13990	294.523	0.904	0.010	1.017	0.002	0.472	0.308
PA14_14000	83.026	1.012	0.011	1.136	0.001	-0.191	0.765
PA14_14010	209.296	1.005	0.009	0.680	0.068	-1.224	0.014
PA14_14020	66.926	1.030	0.056	0.873	0.078	-0.081	0.924
PA14_14040	3678.243	0.600	0.029	0.761	0.002	0.663	0.040
PA14_14060	392.400	-0.604	0.064	-2.036	0.000	-0.249	0.567
PA14_14080	231.997	-0.257	0.511	-1.237	0.000	-2.513	0.000
PA14_14100	524.976	-1.238	0.000	-1.591	0.000	0.826	0.013
PA14_14110	1105.162	-0.134	0.772	0.181	0.621	0.381	0.398
PA14_14130	104.931	-0.456	0.316	-0.471	0.222	0.075	0.901
PA14_14140	195.709	-0.254	0.535	0.275	0.406	0.769	0.049
PA14_14150	425.299	0.471	0.298	1.490	0.000	1.486	0.001
PA14_14160	230.337	-0.228	0.584	0.049	0.897	0.079	0.877
PA14_14170	129.891	-0.904	0.031	-1.083	0.004	-0.902	0.074
PA14_14200	80.573	-0.523	0.259	-0.460	0.249	0.045	0.944
PA14_14210	386.554	0.294	0.353	0.295	0.277	-0.432	0.230
PA14_14220	350.193	0.410	0.274	0.569	0.071	0.091	0.861
PA14_14230	495.485	-0.252	0.454	-1.023	0.000	-0.056	0.896
PA14_14250	191.106	-0.015	0.977	-0.035	0.929	-0.296	0.536
PA14_14270	286.337	-1.006	0.005	-0.966	0.004	-0.181	0.729
PA14_14280	35.316	0.789	0.166	1.162	0.014	-1.037	0.197
PA14_14290	177.786	-0.210	0.627	-0.338	0.318	0.280	0.547
PA14_14300	90.406	-0.043	0.943	-0.289	0.467	-1.943	0.000
PA14_14310	82.313	-0.052	0.936	0.945	0.013	-2.224	0.000
PA14_14320	16.164	-0.550	0.525	-0.828	0.232	-1.122	0.222
PA14_14330	134.012	1.863	0.000	1.437	0.000	-0.396	0.536
PA14_14340	131.164	-0.371	0.419	-1.090	0.002	-0.977	0.043
PA14_14360	764.443	-0.647	0.028	-1.704	0.000	-2.350	0.000
PA14_14370	224.663	0.235	0.614	-1.508	0.000	-2.960	0.000
PA14_14380	417.453	0.511	0.084	-1.091	0.000	-2.473	0.000
PA14_14390	3378.939	-0.098	0.767	-1.647	0.000	-2.419	0.000
PA14_14400	1.785	-1.583	0.249	-0.301	0.830	-1.051	0.412
PA14_14420	48.185	0.916	0.058	0.560	0.221	0.969	0.087
PA14_14430	47.591	0.608	0.269	0.344	0.496	-0.819	0.242
PA14_14440	3621.341	0.335	0.251	-0.224	0.403	0.209	0.563
PA14_14450	853.390	-0.041	0.927	-0.090	0.785	0.538	0.141
PA14_14460	650.880	0.158	0.736	-0.113	0.772	-0.292	0.547
PA14_14470	1796.677	0.408	0.156	-0.042	0.892	-0.039	0.926
PA14_14480	115.848	0.044	0.936	0.288	0.411	0.390	0.402
PA14_14490	32.930	-0.452	0.490	-0.528	0.320	-0.413	0.589
PA14_14500	1345.251	0.416	0.157	0.557	0.030	0.916	0.004
PA14_14510	442.880	0.097	0.822	0.488	0.093	0.464	0.230
PA14_14520	90.541	0.118	0.835	0.831	0.019	0.673	0.167
PA14_14530	259.310	-0.184	0.657	-0.053	0.885	1.163	0.001
PA14_14540	646.844	-2.518	0.000	-3.820	0.000	-3.627	0.000
PA14_14550	1969.667	-2.022	0.000	-2.759	0.000	-2.652	0.000
PA14_14560	201.458	1.556	0.000	3.237	0.000	3.339	0.000
PA14_14570	195.178	0.512	0.359	2.523	0.000	0.923	0.112
PA14_14590	636.927	1.066	0.000	1.863	0.000	1.765	0.000
PA14_14600	1058.290	0.554	0.144	1.320	0.000	1.040	0.011
PA14_14610	1177.806	-0.010	0.980	0.443	0.099	0.019	0.967
PA14_14630	5996.608	0.353	0.358	0.274	0.422	0.121	0.808
PA14_14650	689.432	0.140	0.729	0.171	0.596	-0.211	0.626
PA14_14660	4612.998	-0.031	0.962	0.751	0.046	1.951	0.000

PA14_13920	253.733	0.760	0.031
PA14_13940	16277.627	-1.418	0.000
PA14_13950	12995.491	-0.443	0.370
PA14_13960	88.353	-0.798	0.125
PA14_13970	1572.904	-2.422	0.000
PA14_13990	1381.416	1.492	0.000
PA14_14000	241.031	0.053	0.870
PA14_14010	283.486	1.025	0.000
PA14_14020	132.671	0.152	0.662
PA14_14040	6324.765	0.923	0.000
PA14_14060	564.266	-0.982	0.000
PA14_14080	554.335	1.518	0.000
PA14_14100	1717.803	-2.332	0.000
PA14_14110	2634.138	-0.287	0.215
PA14_14130	339.170	-0.001	0.986
PA14_14140	835.847	-0.531	0.024
PA14_14150	1096.876	-0.288	0.233
PA14_14160	664.229	-0.181	0.489
PA14_14170	427.742	-0.470	0.067
PA14_14200	234.703	-0.705	0.183
PA14_14210	749.281	0.216	0.414
PA14_14220	1880.528	1.236	0.000
PA14_14230	993.541	-0.894	0.000
PA14_14250	395.941	-0.394	0.125
PA14_14270	2006.179	-2.049	0.000
PA14_14280	140.959	0.851	0.008
PA14_14290	494.190	-0.398	0.122
PA14_14300	105.812	0.356	0.338
PA14_14310	389.491	-0.770	0.002
PA14_14320	40.638	-1.112	0.030
PA14_14330	396.766	1.197	0.000
PA14_14340	972.030	1.613	0.000
PA14_14360	2467.067	-0.027	0.905
PA14_14370	272.903	1.215	0.000
PA14_14380	539.043	2.137	0.000
PA14_14390	5845.857	0.605	0.004
PA14_14400	3.650	-1.131	0.574
PA14_14420	89.666	0.109	0.769
PA14_14430	212.978	-1.514	0.000
PA14_14440	3369.925	1.381	0.000
PA14_14450	3098.966	-1.930	0.000
PA14_14460	1413.747	-0.470	0.204
PA14_14470	2852.582	0.562	0.008
PA14_14480	263.973	-0.525	0.059
PA14_14490	168.756	-0.856	0.030
PA14_14500	2284.248	1.177	0.000
PA14_14510	691.642	1.684	0.000
PA14_14520	229.965	1.188	0.000
PA14_14530	583.310	-1.082	0.000
PA14_14540	79217.305	-6.442	0.000
PA14_14550	133552.455	-6.091	0.000
PA14_14560	322.823	2.277	0.000
#N/A	#N/A	#N/A	#N/A
PA14_14590	1174.772	0.339	0.133
PA14_14600	1855.317	0.854	0.000
PA14_14610	2060.960	1.260	0.000
PA14_14630	5373.080	1.606	0.000
PA14_14650	1273.421	0.916	0.000
PA14_14660	21670.103	-1.218	0.000

PA14_14680	1570.214	0.781	0.025	1.818	0.000	2.417	0.000	PA14_14680	2695.644	2.472	0.000
PA14_14690	1092.517	1.058	0.000	2.008	0.000	1.663	0.000	PA14_14690	1640.971	1.480	0.000
PA14_14700	501.303	0.635	0.025	1.132	0.000	1.014	0.001	PA14_14700	780.949	0.557	0.018
PA14_14710	6981.717	1.385	0.001	2.943	0.000	6.323	0.000	PA14_14710	4635.850	-2.434	0.000
PA14_14730	14918.202	0.723	0.022	1.057	0.000	3.497	0.000	PA14_14730	11916.521	-1.756	0.000
PA14_14740	5233.377	0.792	0.008	1.285	0.000	3.864	0.000	PA14_14740	3191.111	-0.833	0.000
PA14_14750	1578.933	0.163	0.723	0.753	0.020	4.181	0.000	PA14_14750	1310.958	-0.804	0.000
PA14_14770	3893.560	1.221	0.000	2.004	0.000	4.357	0.000	PA14_14770	1926.082	-0.457	0.041
PA14_14780	3671.507	0.711	0.007	1.718	0.000	3.976	0.000	PA14_14780	1850.975	0.948	0.000
PA14_14800	2043.136	0.813	0.003	1.787	0.000	3.326	0.000	PA14_14800	1600.462	0.686	0.014
PA14_14810	294.562	0.317	0.506	1.700	0.000	2.871	0.000	PA14_14810	621.581	-0.162	0.591
PA14_14820	1409.314	0.546	0.098	0.900	0.002	1.447	0.000	PA14_14820	1708.990	0.711	0.001
PA14_14830	3055.856	0.117	0.758	0.547	0.041	2.154	0.000	PA14_14830	3778.070	0.918	0.000
PA14_14850	3466.004	0.348	0.248	0.557	0.029	2.039	0.000	PA14_14850	3101.077	0.396	0.067
PA14_14860	912.471	-0.070	0.878	-0.180	0.586	0.784	0.033	PA14_14860	1486.578	0.171	0.482
PA14_14880	3007.469	0.028	0.944	-0.007	0.982	0.987	0.001	PA14_14880	3026.619	0.174	0.472
PA14_14890	4069.853	0.184	0.591	-0.026	0.935	0.526	0.118	PA14_14890	5045.239	0.232	0.315
PA14_14900	2969.342	-0.112	0.778	-0.185	0.549	0.565	0.114	PA14_14900	4419.731	0.478	0.027
PA14_14910	3029.062	-0.035	0.920	0.405	0.071	0.470	0.103	PA14_14910	3905.497	0.402	0.070
PA14_14930	4449.181	0.463	0.126	1.157	0.000	1.234	0.000	PA14_14930	5268.464	0.131	0.584
PA14_14940	637.336	0.351	0.344	0.743	0.013	0.207	0.653	PA14_14940	1145.251	0.024	0.900
PA14_14960	435.821	0.173	0.621	-0.569	0.029	0.374	0.294	PA14_14960	2407.117	-1.491	0.000
PA14_14975	186.369	-0.197	0.667	-0.349	0.322	-1.190	0.006	PA14_14975	1166.069	-1.883	0.000
PA14_14990	1326.809	-0.988	0.000	-1.127	0.000	-0.319	0.346	PA14_14990	5062.144	-1.333	0.000
PA14_15000	214.602	0.688	0.039	0.938	0.001	0.225	0.634	PA14_15000	475.053	0.585	0.015
PA14_15020	348.789	0.138	0.718	0.244	0.405	-0.462	0.221	PA14_15020	1156.686	-0.124	0.757
PA14_15030	18897.059	0.664	0.042	0.837	0.005	-0.461	0.257	PA14_15030	13658.510	0.742	0.005
PA14_15050	36.226	-2.554	0.000	-3.012	0.000	-0.579	0.419	PA14_15050	87.731	-0.968	0.008
PA14_15070	260.595	-2.864	0.000	-3.878	0.000	-2.266	0.000	PA14_15070	248.903	-0.975	0.000
PA14_15080	155.673	-2.371	0.000	-2.536	0.000	-2.783	0.000	PA14_15080	197.155	-1.079	0.000
PA14_15090	100.438	-1.378	0.000	-2.155	0.000	1.025	0.022	PA14_15090	616.620	-1.899	0.000
PA14_15100	236.209	-0.760	0.014	-0.814	0.004	0.517	0.165	PA14_15100	832.734	-1.058	0.000
PA14_15110	2206.899	0.510	0.259	0.030	0.951	1.556	0.001	PA14_15110	4777.264	-1.368	0.000
PA14_15120	525.079	0.053	0.917	-0.400	0.227	0.928	0.017	PA14_15120	1765.734	-1.066	0.000
PA14_15130	195.440	0.119	0.788	-0.260	0.429	0.322	0.459	PA14_15130	773.326	-0.613	0.009
PA14_15140	208.297	3.634	0.000	2.774	0.000	0.613	0.230	PA14_15140	135.047	1.333	0.000
PA14_15150	33.394	1.002	0.078	1.369	0.005	0.609	0.411	PA14_15150	129.211	0.920	0.017
PA14_15160	117.044	1.860	0.000	2.224	0.000	0.382	0.500	PA14_15160	135.978	1.106	0.001
PA14_15180	261.214	1.730	0.000	2.363	0.000	0.807	0.116	PA14_15180	273.977	0.571	0.042
PA14_15190	19.454	1.567	0.030	2.735	0.000	1.233	0.158	PA14_15190	19.796	2.021	0.005
PA14_15200	248.736	2.335	0.000	3.104	0.000	2.404	0.000	PA14_15200	461.508	1.686	0.000
PA14_15210	104.326	0.934	0.020	1.461	0.000	0.162	0.796	PA14_15210	156.452	0.544	0.078
PA14_15230	99.475	0.085	0.880	0.593	0.095	-0.679	0.196	PA14_15230	305.223	0.228	0.464
PA14_15240	44.513	1.011	0.038	1.079	0.013	0.015	0.985	PA14_15240	67.392	2.051	0.000
PA14_15250	6.309	-0.642	0.596	-0.025	0.981	-0.408	0.754	PA14_15250	17.451	0.882	0.275
PA14_15260	4.820	-0.762	0.559	0.604	0.563	-0.796	0.538	PA14_15260	7.427	0.512	0.714
PA14_15270	4.619	-0.982	0.420	-0.956	0.350	-1.550	0.190	PA14_15270	6.406	0.659	0.711
PA14_15280	16.265	-1.486	0.061	-2.197	0.002	0.254	0.804	PA14_15280	56.975	-1.968	0.043
PA14_15290	66.527	-0.632	0.184	-0.960	0.018	-0.156	0.811	PA14_15290	269.854	-1.672	0.000
PA14_15310	5831.739	0.192	0.556	0.468	0.062	1.143	0.000	PA14_15310	7491.085	0.989	0.000
PA14_15340	3182.258	0.197	0.587	-0.182	0.555	0.640	0.070	PA14_15340	2007.466	0.364	0.103
PA14_15350	6047.750	1.745	0.000	1.928	0.000	0.712	0.013	PA14_15350	7335.259	2.488	0.000
PA14_15360	3859.896	0.998	0.000	0.893	0.000	-0.397	0.211	PA14_15360	10050.309	-0.971	0.000
PA14_15370	59.569	1.407	0.017	2.211	0.000	0.360	0.680	PA14_15370	59.049	1.475	0.000
PA14_15380	166.671	0.985	0.005	2.332	0.000	2.474	0.000	PA14_15380	401.266	1.129	0.000
PA14_15400	231.110	0.178	0.685	0.550	0.080	0.831	0.035	PA14_15400	973.924	-1.164	0.000
PA14_15410	29.780	1.270	0.038	0.672	0.263	1.700	0.011	#N/A	#N/A	#N/A	#N/A
PA14_15420	16.742	0.687	0.429	1.032	0.132	1.132	0.196	#N/A	#N/A	#N/A	#N/A
PA14_15430	84.472	0.308	0.559	0.348	0.415	0.587	0.279	PA14_15430	245.719	0.283	0.338

PA14_15435	189.849	0.225	0.585	0.143	0.686	0.043	0.935	PA14_15435	492.250	0.642	0.008
PA14_15445	74.680	0.427	0.459	0.689	0.130	0.537	0.394	PA14_15445	272.237	-0.431	0.118
PA14_15450	75.184	1.462	0.005	1.841	0.000	1.835	0.002	PA14_15450	171.659	0.096	0.794
PA14_15460	289.171	0.694	0.024	0.398	0.178	0.358	0.378	PA14_15460	745.946	1.071	0.001
PA14_15470	74.832	0.812	0.069	-0.069	0.891	0.132	0.847	PA14_15470	270.567	0.380	0.172
PA14_15475	308.962	1.352	0.000	0.574	0.134	1.316	0.004	PA14_15475	933.617	-0.168	0.493
PA14_15480	620.080	0.227	0.701	-0.166	0.738	-1.500	0.004	PA14_15480	1722.463	0.551	0.030
PA14_15490	122.482	0.860	0.032	1.638	0.000	1.957	0.000	PA14_15490	390.809	0.844	0.001
PA14_15500	150.478	0.459	0.220	0.631	0.045	0.671	0.107	PA14_15500	662.786	-0.476	0.051
PA14_15510	326.874	0.676	0.082	0.835	0.015	1.541	0.000	PA14_15510	827.510	-0.053	0.853
PA14_15520	135.643	1.115	0.002	2.067	0.000	2.280	0.000	PA14_15520	395.270	0.239	0.380
PA14_15530	82.234	-1.570	0.002	-1.477	0.002	-1.662	0.006	PA14_15530	860.313	-1.385	0.033
PA14_15540	397.310	-0.164	0.705	-0.362	0.271	-0.504	0.236	PA14_15540	2199.185	-0.463	0.037
PA14_15560	563.585	0.697	0.009	0.371	0.157	-0.108	0.793	PA14_15560	1690.307	-0.456	0.171
PA14_15570	6683.222	0.831	0.050	1.780	0.000	0.337	0.548	PA14_15570	9742.533	-0.369	0.088
PA14_15580	12015.971	0.875	0.001	1.027	0.000	-0.241	0.527	PA14_15580	24775.080	1.299	0.000
PA14_15590	8429.906	0.453	0.117	0.467	0.074	-0.754	0.020	PA14_15590	15540.172	0.580	0.005
PA14_15600	8367.035	0.650	0.143	1.176	0.002	-0.431	0.431	PA14_15600	9941.866	-0.023	0.908
PA14_15610	3592.510	-0.066	0.851	-0.120	0.652	-1.128	0.000	PA14_15610	8954.270	-0.262	0.236
PA14_15620	43.023	1.732	0.003	2.618	0.000	1.588	0.024	PA14_15620	94.177	0.470	0.213
PA14_15630	114.671	0.599	0.137	1.013	0.002	1.078	0.013	PA14_15630	322.737	-0.082	0.781
PA14_15650	114.188	0.011	0.985	-0.138	0.758	-1.053	0.047	PA14_15650	415.740	0.312	0.239
PA14_15660	169.159	0.629	0.126	1.819	0.000	0.686	0.153	PA14_15660	386.728	0.263	0.331
PA14_15670	1738.262	0.448	0.093	0.674	0.004	-0.784	0.010	PA14_15670	3153.888	0.535	0.013
PA14_15680	352.036	-0.162	0.700	0.032	0.932	0.080	0.872	PA14_15680	1586.563	-0.504	0.026
PA14_15700	77.626	1.241	0.010	1.978	0.000	0.948	0.115	PA14_15700	151.501	0.907	0.003
PA14_15710	116.337	0.423	0.406	1.099	0.004	0.387	0.516	PA14_15710	254.123	-0.760	0.005
PA14_15720	1713.241	0.496	0.119	0.546	0.056	-0.125	0.779	PA14_15720	2582.286	0.256	0.295
PA14_15740	3470.874	0.582	0.029	0.850	0.000	0.828	0.007	PA14_15740	3375.717	0.901	0.000
PA14_15750	33.193	0.704	0.363	3.507	0.000	2.580	0.000	PA14_15750	22.467	-0.468	0.539
PA14_15770	79.552	-0.841	0.040	-0.813	0.026	-1.299	0.010	PA14_15770	854.779	-1.141	0.001
PA14_15780	89.155	1.074	0.004	0.068	0.877	0.064	0.918	PA14_15780	201.369	0.185	0.651
PA14_15790	356.374	1.765	0.000	1.073	0.000	1.770	0.000	PA14_15790	579.119	0.643	0.007
PA14_15810	109.100	1.066	0.010	0.241	0.589	1.652	0.000	PA14_15810	158.679	0.884	0.003
PA14_15820	141.420	1.420	0.000	0.004	0.994	1.901	0.000	PA14_15820	217.166	1.380	0.000
PA14_15830	263.935	1.420	0.000	0.372	0.305	3.170	0.000	PA14_15830	225.398	1.042	0.000
PA14_15840	687.053	-0.219	0.517	0.121	0.682	1.212	0.000	PA14_15840	2531.602	-0.566	0.008
PA14_15850	440.084	0.461	0.129	0.497	0.063	1.412	0.000	PA14_15850	1654.002	-0.354	0.135
PA14_15860	1297.786	-0.613	0.018	-1.713	0.000	-0.987	0.001	PA14_15860	5595.776	-1.038	0.001
PA14_15870	679.108	-0.794	0.002	-1.586	0.000	-0.877	0.005	PA14_15870	2347.308	-0.794	0.001
PA14_15880	67.294	-0.637	0.154	-1.801	0.000	-1.234	0.021	PA14_15880	455.258	-1.005	0.000
PA14_15890	352.600	-0.536	0.112	-1.512	0.000	-2.168	0.000	PA14_15890	1114.991	-0.175	0.460
PA14_15910	157.735	0.951	0.006	1.384	0.000	0.233	0.659	PA14_15910	318.472	0.982	0.000
PA14_15920	73.291	1.627	0.000	2.766	0.000	2.086	0.000	PA14_15920	99.340	2.281	0.000
PA14_15930	384.490	0.276	0.462	0.507	0.088	-2.129	0.000	PA14_15930	472.416	0.754	0.002
PA14_15940	436.522	0.503	0.114	0.749	0.007	-0.580	0.134	PA14_15940	1188.729	1.862	0.000
PA14_15960	6140.364	0.195	0.540	0.573	0.019	0.419	0.200	PA14_15960	7669.415	1.243	0.000
PA14_15970	4147.824	0.377	0.204	1.249	0.000	2.141	0.000	PA14_15970	3172.830	2.032	0.000
PA14_15980	6326.431	0.689	0.009	1.806	0.000	1.928	0.000	PA14_15980	4703.018	0.979	0.000
PA14_15990	19443.435	1.295	0.000	2.698	0.000	2.377	0.000	PA14_15990	6751.131	1.640	0.000
PA14_16000	1970.018	0.793	0.014	1.313	0.000	0.373	0.382	PA14_16000	1374.293	1.304	0.000
PA14_16010	325.226	1.836	0.000	2.868	0.000	1.871	0.000	PA14_16010	157.506	2.026	0.000
PA14_16020	608.937	-1.438	0.000	-3.042	0.000	0.564	0.155	PA14_16020	2033.700	-2.372	0.000
PA14_16030	391.397	-1.377	0.000	-1.818	0.000	-0.348	0.381	PA14_16030	526.072	-0.494	0.045
PA14_16040	364.664	0.418	0.262	0.690	0.026	0.731	0.068	PA14_16040	567.326	0.911	0.000
PA14_16050	3705.552	0.380	0.280	1.032	0.000	0.988	0.006	PA14_16050	5643.095	0.256	0.364
PA14_16070	1200.359	0.457	0.104	0.895	0.000	0.152	0.695	PA14_16070	1515.338	0.329	0.155
PA14_16090	485.435	0.171	0.701	-0.283	0.415	-0.992	0.015	PA14_16090	474.705	0.325	0.213
PA14_16100	130.846	-2.823	0.000	-3.039	0.000	-1.776	0.000	PA14_16100	1852.818	-4.842	0.000

PA14_16110	276.141	-0.998	0.002	-0.327	0.320	1.574	0.000	PA14_16110	1142.035	-1.821	0.000
PA14_16130	207.155	-0.417	0.243	-0.405	0.189	-0.482	0.251	PA14_16130	624.486	-0.107	0.672
PA14_16140	3268.286	1.201	0.000	0.052	0.868	-0.440	0.211	PA14_16140	4237.247	2.309	0.000
PA14_16150	3899.304	1.008	0.001	0.603	0.037	-0.688	0.070	PA14_16150	4550.627	2.341	0.000
PA14_16160	1199.243	0.995	0.000	0.678	0.012	0.100	0.824	PA14_16160	2445.016	2.596	0.000
PA14_16180	3144.260	0.294	0.302	-0.047	0.871	-0.946	0.001	PA14_16180	8177.476	2.449	0.000
PA14_16190	1605.621	0.549	0.048	0.614	0.015	-0.581	0.083	PA14_16190	3227.622	0.418	0.055
PA14_16200	135.297	0.312	0.447	-0.187	0.607	-2.613	0.000	PA14_16200	491.331	-1.297	0.000
PA14_16210	442.738	0.653	0.052	1.952	0.000	0.708	0.073	PA14_16210	421.041	1.189	0.000
PA14_16220	243.558	0.229	0.586	1.054	0.000	0.626	0.130	PA14_16220	259.780	1.111	0.000
PA14_16250	12982.002	-3.712	0.000	-6.166	0.000	-0.839	0.003	PA14_16250	19521.836	-3.516	0.000
PA14_16260	650.134	-2.911	0.000	-4.758	0.000	0.163	0.727	PA14_16260	4855.395	-4.079	0.000
PA14_16270	3907.022	0.416	0.327	0.497	0.171	2.822	0.000	PA14_16270	5486.712	-0.172	0.424
PA14_16280	669.363	-0.860	0.001	-0.667	0.007	-1.426	0.000	PA14_16280	3162.248	-2.283	0.000
PA14_16290	328.481	0.291	0.562	-0.060	0.899	-0.632	0.223	PA14_16290	1961.586	-1.545	0.000
PA14_16300	42.770	0.851	0.118	1.155	0.013	0.967	0.122	PA14_16300	225.396	-1.147	0.002
PA14_16310	38.905	0.433	0.560	2.193	0.000	1.485	0.025	PA14_16310	282.917	-1.294	0.000
PA14_16320	360.257	-0.029	0.948	-0.152	0.617	-1.486	0.000	PA14_16320	789.930	0.490	0.041
PA14_16330	631.470	0.225	0.604	0.329	0.343	-0.785	0.063	PA14_16330	1458.318	-0.040	0.875
PA14_16340	96.649	-1.203	0.005	-1.871	0.000	-1.690	0.001	PA14_16340	562.330	-1.915	0.000
PA14_16350	43.961	-0.211	0.752	-0.952	0.046	0.565	0.372	PA14_16350	219.672	-1.937	0.000
PA14_16360	139.406	-0.467	0.251	-0.889	0.008	-0.698	0.130	PA14_16360	299.041	-0.880	0.001
PA14_16370	705.708	-2.335	0.000	-2.655	0.000	-0.583	0.124	PA14_16370	5071.838	-1.541	0.000
PA14_16380	59.169	0.006	0.992	-0.711	0.094	-1.510	0.011	PA14_16380	150.565	0.924	0.002
PA14_16390	539.193	-1.124	0.001	-2.660	0.000	-0.140	0.787	PA14_16390	346.858	0.438	0.109
PA14_16410	69.351	-1.359	0.002	-3.099	0.000	-1.229	0.025	PA14_16410	62.670	-0.302	0.726
PA14_16430	1100.230	0.029	0.938	0.036	0.901	-0.861	0.003	PA14_16430	4048.678	-0.957	0.000
PA14_16440	93.959	0.310	0.514	0.217	0.595	-0.683	0.204	PA14_16440	523.656	-0.338	0.159
PA14_16450	213.344	0.296	0.442	0.265	0.416	-0.616	0.146	PA14_16450	646.696	0.577	0.016
PA14_16460	506.750	0.753	0.055	0.917	0.009	0.386	0.449	PA14_16460	863.703	0.631	0.006
PA14_16470	1112.330	0.456	0.088	0.253	0.322	-0.408	0.213	PA14_16470	2333.399	-0.275	0.232
PA14_16480	598.697	-0.095	0.821	-0.080	0.812	0.268	0.507	PA14_16480	1628.301	-0.723	0.001
PA14_16500	1314.409	0.477	0.316	1.076	0.005	0.234	0.696	PA14_16500	1758.384	-0.281	0.216
PA14_16510	3034.424	0.336	0.197	0.689	0.002	1.105	0.000	PA14_16510	4342.550	0.899	0.000
PA14_16530	7706.949	0.810	0.000	1.266	0.000	0.483	0.105	PA14_16530	6762.443	1.073	0.000
PA14_16550	334.600	0.551	0.059	0.444	0.098	0.096	0.826	PA14_16550	1135.509	0.251	0.302
PA14_16560	475.400	-0.254	0.470	-1.122	0.000	-0.706	0.048	PA14_16560	1994.463	-0.251	0.293
PA14_16580	1629.743	0.047	0.899	-0.349	0.153	-0.281	0.398	PA14_16580	3329.524	0.383	0.076
PA14_16590	242.850	-0.298	0.397	-0.968	0.000	-1.580	0.000	PA14_16590	511.417	0.121	0.686
PA14_16600	1809.505	0.744	0.002	0.831	0.000	-0.209	0.549	PA14_16600	1758.068	0.514	0.020
PA14_16610	127.290	-0.306	0.574	-0.465	0.284	-1.248	0.021	PA14_16610	388.148	-0.117	0.711
PA14_16620	156.147	0.468	0.284	0.890	0.012	-0.480	0.369	PA14_16620	269.013	0.005	0.975
PA14_16630	1914.527	-1.593	0.000	-2.268	0.000	1.201	0.002	PA14_16630	7166.676	-1.724	0.000
PA14_16640	2250.899	-1.742	0.000	-2.663	0.000	0.848	0.003	PA14_16640	10825.753	-2.260	0.000
PA14_16660	580.105	-1.317	0.000	-2.215	0.000	-0.298	0.396	PA14_16660	1501.153	-1.829	0.000
PA14_16670	203.218	0.215	0.560	-0.273	0.366	-1.112	0.003	PA14_16670	353.737	-0.866	0.009
PA14_16680	146.749	-2.004	0.000	-4.133	0.000	0.634	0.117	PA14_16680	1320.185	-4.224	0.000
PA14_16690	1006.855	-0.279	0.345	-0.685	0.004	-0.896	0.003	PA14_16690	2974.590	-0.393	0.080
PA14_16700	2221.166	-0.536	0.098	-0.928	0.001	-1.710	0.000	PA14_16700	3428.635	1.313	0.000
PA14_16710	284.460	1.057	0.000	2.026	0.000	1.462	0.000	PA14_16710	405.340	1.779	0.000
PA14_16720	249.002	0.070	0.898	0.297	0.425	-0.705	0.134	PA14_16720	1800.919	-0.450	0.041
PA14_16730	160.928	0.213	0.630	0.556	0.087	-0.276	0.586	PA14_16730	602.919	0.586	0.014
PA14_16740	49.828	-0.399	0.516	0.422	0.382	-0.159	0.838	PA14_16740	157.047	0.038	0.927
PA14_16750	25.755	-0.286	0.726	-0.386	0.546	-0.385	0.663	PA14_16750	75.159	0.285	0.541
PA14_16770	549.032	1.517	0.000	2.158	0.000	1.202	0.019	PA14_16770	524.338	1.313	0.000
PA14_16780	98.747	0.720	0.087	1.018	0.005	-0.477	0.410	PA14_16780	150.351	0.810	0.010
PA14_16790	344.997	-0.142	0.819	-0.512	0.243	-1.815	0.000	PA14_16790	1065.771	-0.649	0.004
PA14_16800	449.680	-1.575	0.000	-0.501	0.063	-2.820	0.000	PA14_16800	1424.973	-1.920	0.000
PA14_16820	1039.558	-1.249	0.000	-0.558	0.033	-2.509	0.000	PA14_16820	3608.729	0.148	0.537

PA14_16830	743.109	0.337	0.334	0.306	0.314	-0.592	0.119	PA14_16830	957.315	1.294	0.000
PA14_16840	431.030	-0.514	0.097	-0.656	0.017	-2.089	0.000	PA14_16840	1348.988	-1.019	0.000
PA14_16860	1516.984	0.847	0.020	1.330	0.000	1.016	0.016	PA14_16860	2017.729	-0.014	0.966
PA14_16870	45.115	-0.728	0.203	-0.391	0.451	-0.644	0.349	PA14_16870	219.781	-0.372	0.227
PA14_16880	91.238	-0.733	0.103	-0.760	0.056	-0.777	0.148	PA14_16880	248.525	-0.290	0.366
PA14_16890	248.776	-0.236	0.507	-0.734	0.007	-2.196	0.000	PA14_16890	559.083	-0.643	0.006
PA14_16910	123.199	0.017	0.975	-0.763	0.021	-0.633	0.171	PA14_16910	364.805	-1.030	0.000
PA14_16920	66.901	0.202	0.773	0.425	0.413	0.953	0.123	PA14_16920	199.580	-0.121	0.812
PA14_16930	340.601	-0.034	0.946	0.062	0.866	0.711	0.066	PA14_16930	914.701	-0.663	0.004
PA14_16950	922.736	0.379	0.359	-0.469	0.184	-0.653	0.143	PA14_16950	1887.467	1.742	0.000
PA14_16960	135.088	0.118	0.834	0.356	0.364	-0.118	0.849	PA14_16960	564.865	0.824	0.000
PA14_16970	198.479	0.014	0.978	-0.123	0.732	-0.380	0.402	PA14_16970	833.810	0.331	0.172
PA14_16980	271.544	1.048	0.002	1.610	0.000	0.929	0.026	PA14_16980	339.667	0.716	0.006
PA14_16990	526.846	-0.213	0.603	-1.314	0.000	-0.111	0.822	PA14_16990	2429.256	-1.619	0.000
PA14_17000	52.491	-0.193	0.772	-0.636	0.185	1.321	0.014	PA14_17000	76.169	-0.413	0.425
PA14_17010	977.424	0.472	0.091	0.380	0.141	-0.343	0.331	PA14_17010	2993.702	0.516	0.016
PA14_17030	360.556	0.181	0.623	0.000	0.999	0.096	0.829	PA14_17030	585.039	0.572	0.016
PA14_17040	2660.105	0.621	0.011	0.994	0.000	1.317	0.000	PA14_17040	3451.573	1.193	0.000
PA14_17050	4714.843	0.885	0.001	0.964	0.000	0.850	0.011	PA14_17050	9381.572	0.232	0.283
PA14_17060	10956.222	0.945	0.001	1.449	0.000	0.908	0.009	PA14_17060	7838.715	1.828	0.000
PA14_17070	5235.375	0.549	0.090	0.804	0.005	0.634	0.092	PA14_17070	4502.946	1.527	0.000
PA14_17080	8487.274	0.506	0.067	0.773	0.002	0.647	0.042	PA14_17080	8312.218	0.500	0.018
PA14_17100	13408.091	0.204	0.526	0.069	0.819	-0.671	0.033	PA14_17100	13368.262	-0.087	0.694
PA14_17110	1055.898	0.662	0.020	0.903	0.000	0.379	0.302	PA14_17110	2012.974	0.403	0.071
PA14_17120	497.621	0.263	0.425	0.504	0.055	-0.300	0.430	PA14_17120	438.716	0.942	0.000
PA14_17130	1460.990	0.283	0.458	0.936	0.001	0.515	0.197	PA14_17130	1479.012	0.371	0.286
PA14_17140	3214.028	-0.190	0.622	0.867	0.002	0.206	0.632	PA14_17140	7058.057	0.693	0.001
PA14_17150	9993.974	0.243	0.447	0.451	0.079	-0.133	0.734	PA14_17150	13330.028	1.484	0.000
PA14_17170	1844.616	-0.109	0.775	-0.539	0.045	-1.377	0.000	PA14_17170	2883.405	0.480	0.027
PA14_17180	3220.236	0.264	0.333	0.454	0.044	-0.402	0.180	PA14_17180	4347.052	0.748	0.000
PA14_17190	3035.490	0.641	0.025	1.104	0.000	0.781	0.019	PA14_17190	4104.453	1.287	0.000
PA14_17210	2305.720	0.464	0.229	0.849	0.009	-0.667	0.121	PA14_17210	2754.487	0.833	0.000
PA14_17220	845.671	0.642	0.024	0.920	0.000	-0.327	0.388	PA14_17220	1023.641	1.626	0.000
PA14_17230	356.644	0.421	0.190	0.800	0.003	-1.078	0.004	PA14_17230	454.930	1.027	0.000
PA14_17250	842.311	0.885	0.007	1.129	0.000	-1.731	0.000	PA14_17250	534.280	1.480	0.000
PA14_17260	7067.984	0.876	0.002	1.600	0.000	0.833	0.016	PA14_17260	6537.442	0.737	0.000
PA14_17270	3896.089	1.066	0.004	1.375	0.000	0.992	0.028	PA14_17270	6458.112	0.445	0.205
PA14_17280	207.011	0.450	0.183	0.751	0.008	-0.070	0.889	PA14_17280	338.760	0.147	0.630
PA14_17290	10777.001	0.973	0.000	1.778	0.000	2.685	0.000	PA14_17290	9372.039	0.695	0.001
PA14_17310	5773.742	0.898	0.008	1.291	0.000	2.224	0.000	PA14_17310	8221.070	-0.038	0.971
PA14_17320	8490.687	-0.176	0.542	-0.125	0.619	0.725	0.008	PA14_17320	6561.524	0.191	0.415
PA14_17330	535.626	0.953	0.008	1.590	0.000	2.806	0.000	PA14_17330	643.918	-0.961	0.014
PA14_17340	1222.960	0.852	0.020	2.059	0.000	2.539	0.000	PA14_17340	902.636	0.494	0.037
PA14_17350	64.410	-1.250	0.006	-1.940	0.000	-2.059	0.000	PA14_17350	487.681	1.158	0.000
PA14_17370	803.692	0.222	0.478	-0.059	0.842	-0.281	0.423	PA14_17370	2058.569	1.350	0.000
PA14_17380	224.933	0.594	0.078	0.585	0.053	-0.378	0.405	PA14_17380	786.081	-0.210	0.390
PA14_17400	943.481	-0.321	0.291	-0.729	0.003	-0.247	0.504	PA14_17400	2298.971	-0.108	0.674
PA14_17410	249.783	0.168	0.704	0.370	0.262	1.155	0.002	PA14_17410	488.197	0.218	0.423
PA14_17420	321.183	-0.387	0.246	-0.453	0.112	-0.446	0.248	PA14_17420	1109.026	-0.073	0.759
PA14_17440	370.539	0.434	0.296	0.506	0.155	0.462	0.334	PA14_17440	1326.852	0.481	0.034
PA14_17450	523.268	0.112	0.749	-0.233	0.378	-0.544	0.099	PA14_17450	1262.434	1.248	0.000
PA14_17460	1987.670	-0.068	0.886	-0.381	0.235	-0.655	0.098	PA14_17460	4700.029	-0.090	0.694
PA14_17470	24447.986	-1.371	0.000	-1.308	0.000	-0.085	0.866	PA14_17470	37834.465	-1.140	0.000
PA14_17480	12512.598	-1.163	0.000	-1.842	0.000	-0.363	0.322	PA14_17480	25817.098	-1.575	0.000
PA14_17490	2467.323	-0.394	0.341	-0.504	0.151	-1.912	0.000	PA14_17490	6573.296	0.562	0.009
PA14_17500	1253.083	0.569	0.047	0.644	0.013	0.165	0.683	PA14_17500	2178.110	1.378	0.000
PA14_17510	115.234	-0.081	0.881	-0.657	0.060	1.149	0.005	PA14_17510	210.764	-0.327	0.322
PA14_17520	346.987	0.535	0.088	0.556	0.048	0.657	0.070	PA14_17520	796.977	0.151	0.596
PA14_17530	3035.302	0.669	0.010	0.937	0.000	1.836	0.000	PA14_17530	4703.562	-0.987	0.000

PA14_17540	77.031	0.861	0.083	1.473	0.000	1.604	0.002	PA14_17540	225.801	-0.392	0.188
PA14_17550	1046.018	-1.302	0.000	-2.081	0.000	-1.949	0.000	PA14_17550	9948.388	-0.379	0.233
PA14_17570	2679.302	-2.046	0.000	-3.688	0.000	-2.826	0.000	PA14_17570	13083.459	-0.672	0.001
PA14_17580	4005.929	-1.949	0.000	-3.203	0.000	-3.626	0.000	PA14_17580	19485.121	0.340	0.266
PA14_17590	335.869	0.288	0.527	0.982	0.004	0.472	0.329	PA14_17590	836.977	-0.886	0.000
PA14_17600	713.025	-0.565	0.082	-0.724	0.012	-0.450	0.257	PA14_17600	2578.160	-1.311	0.000
PA14_17610	125.119	0.214	0.631	0.544	0.099	-0.445	0.361	PA14_17610	158.176	-0.133	0.705
PA14_17620	38.923	0.644	0.269	1.148	0.014	1.325	0.025	PA14_17620	33.509	0.550	0.328
PA14_17630	58.433	0.772	0.088	0.920	0.020	-0.024	0.973	PA14_17630	59.334	0.946	0.026
PA14_17640	33.959	0.064	0.937	0.605	0.229	-0.480	0.531	PA14_17640	32.133	0.968	0.085
PA14_17650	59.793	0.631	0.216	0.929	0.029	-0.755	0.257	PA14_17650	174.459	1.006	0.001
PA14_17660	65.562	0.779	0.078	1.186	0.002	-0.476	0.455	PA14_17660	130.115	1.626	0.000
PA14_17670	263.358	-0.253	0.505	-0.031	0.932	-1.408	0.000	PA14_17670	866.235	-0.103	0.710
PA14_17675	62.452	0.207	0.741	-0.005	0.993	1.293	0.014	PA14_17675	152.306	0.784	0.012
PA14_17690	1421.793	0.953	0.000	0.520	0.051	-0.128	0.757	PA14_17690	2886.124	0.325	0.143
PA14_17700	1461.994	-0.149	0.710	-0.026	0.940	1.225	0.000	PA14_17700	4359.840	-4.256	0.000
PA14_17710	108.912	0.312	0.705	1.550	0.003	1.460	0.032	PA14_17710	218.890	-3.684	0.000
PA14_17720	141.950	0.316	0.473	-0.233	0.545	0.215	0.686	PA14_17720	444.788	0.191	0.506
PA14_17730	42.976	-0.412	0.534	-0.352	0.521	0.953	0.122	PA14_17730	189.179	-0.031	0.981
PA14_17740	39.629	-0.114	0.883	-0.685	0.191	0.231	0.764	PA14_17740	85.558	1.166	0.001
PA14_17760	44.239	-0.290	0.717	0.051	0.942	0.323	0.700	PA14_17760	80.730	-0.988	0.009
PA14_17780	77.898	1.596	0.000	1.766	0.000	1.749	0.000	PA14_17780	97.501	0.123	0.792
PA14_17790	70.382	1.025	0.034	0.134	0.804	0.341	0.617	PA14_17790	208.581	1.122	0.000
PA14_17810	101.135	3.019	0.000	1.459	0.000	0.751	0.175	PA14_17810	86.055	1.353	0.000
PA14_17820	32.800	3.102	0.000	1.055	0.080	0.716	0.410	PA14_17820	32.974	0.825	0.187
PA14_17850	16.556	4.790	0.000	2.535	0.003	2.106	0.049	PA14_17850	11.754	0.571	0.607
PA14_17860	42.045	1.529	0.002	0.677	0.187	-1.013	0.199	PA14_17860	69.602	0.222	0.666
PA14_17880	10.835	2.130	0.008	0.758	0.375	0.153	0.907	PA14_17880	16.964	0.144	0.940
PA14_17890	52.753	0.881	0.072	0.625	0.169	-0.257	0.731	PA14_17890	166.626	0.153	0.647
PA14_17900	1232.867	2.337	0.000	1.619	0.000	1.329	0.012	PA14_17900	2296.980	-0.795	0.003
PA14_17910	32.703	1.146	0.073	1.126	0.050	-0.094	0.926	PA14_17910	256.269	-3.503	0.000
PA14_17920	189.514	0.708	0.037	0.849	0.005	1.334	0.000	PA14_17920	460.448	-0.366	0.141
PA14_17930	3664.866	0.508	0.168	5.333	0.000	7.795	0.000	PA14_17930	9391.702	-4.344	0.000
PA14_17940	941.935	0.685	0.011	1.735	0.000	4.181	0.000	PA14_17940	1327.487	0.646	0.003
PA14_17960	2473.612	0.428	0.158	3.011	0.000	4.918	0.000	PA14_17960	4209.529	-0.088	0.730
PA14_17980	9914.474	1.926	0.000	5.022	0.000	6.736	0.000	PA14_17980	8849.011	-1.329	0.000
PA14_17990	375.228	0.520	0.124	3.143	0.000	3.328	0.000	PA14_17990	674.060	0.559	0.018
PA14_18010	955.914	0.569	0.046	3.453	0.000	2.782	0.000	PA14_18010	988.011	1.828	0.000
PA14_18020	493.363	0.179	0.660	1.522	0.000	2.262	0.000	PA14_18020	1092.687	-1.682	0.000
PA14_18040	238.586	1.333	0.000	1.947	0.000	3.413	0.000	PA14_18040	627.306	-0.771	0.001
PA14_18050	78.350	-1.342	0.005	-0.985	0.026	-0.584	0.345	PA14_18050	332.085	-1.457	0.000
PA14_18060	63.036	-0.176	0.798	0.402	0.411	-1.750	0.010	PA14_18060	265.102	0.934	0.001
PA14_18070	5617.892	-0.100	0.822	-0.860	0.003	-0.809	0.031	PA14_18070	21386.313	-2.307	0.000
PA14_18080	148.202	-0.092	0.866	0.561	0.110	0.889	0.042	PA14_18080	773.165	0.013	0.944
PA14_18090	189.465	0.811	0.029	1.433	0.000	1.284	0.002	PA14_18090	322.306	1.080	0.000
PA14_18100	1115.603	-1.587	0.000	-2.532	0.000	-2.732	0.000	PA14_18100	6334.120	-0.959	0.077
PA14_18110	174.524	0.226	0.635	0.056	0.898	-0.823	0.088	PA14_18110	587.490	0.333	0.189
PA14_18120	2080.214	-3.242	0.000	-5.592	0.000	-3.080	0.000	PA14_18120	337.706	-0.468	0.087
PA14_18140	480.941	-3.452	0.000	-5.756	0.000	-3.915	0.000	PA14_18140	112.006	-1.243	0.000
PA14_18150	311.388	-2.534	0.000	-2.041	0.000	-1.581	0.000	PA14_18150	183.857	0.164	0.613
PA14_18160	800.725	1.494	0.000	2.812	0.000	-0.859	0.023	PA14_18160	585.032	1.047	0.000
PA14_18180	2455.475	2.556	0.000	4.340	0.000	1.308	0.002	PA14_18180	1068.635	0.400	0.348
PA14_18200	92.979	0.869	0.032	0.940	0.009	-1.361	0.021	PA14_18200	244.986	1.099	0.000
PA14_18210	31.582	1.827	0.002	1.887	0.001	0.624	0.479	PA14_18210	74.465	1.332	0.001
PA14_18230	153.630	0.878	0.017	1.176	0.000	-0.234	0.678	PA14_18230	372.754	0.732	0.004
PA14_18250	701.513	2.283	0.000	4.042	0.000	-0.168	0.709	PA14_18250	1280.742	1.744	0.000
PA14_18260	94.604	1.692	0.000	3.610	0.000	-0.059	0.944	PA14_18260	256.433	1.621	0.000
PA14_18275	127.480	3.656	0.000	5.341	0.000	1.698	0.006	PA14_18275	404.086	1.561	0.000
PA14_18300	620.943	-3.414	0.000	-4.495	0.000	-3.462	0.000	PA14_18300	2082.010	0.522	0.162

PA14_18310	324.699	-2.773	0.000	-3.963	0.000	-2.734	0.000	PA14_18310	1482.825	-0.110	0.718
PA14_18320	46.631	-2.751	0.000	-3.158	0.000	-2.018	0.000	PA14_18320	215.527	0.885	0.166
PA14_18330	65.363	-3.335	0.000	-3.725	0.000	-3.776	0.000	PA14_18330	367.838	0.659	0.242
PA14_18340	30.402	-3.321	0.000	-3.249	0.000	-2.948	0.000	PA14_18340	293.968	-0.264	0.349
PA14_18350	576.473	-3.246	0.000	-4.415	0.000	-3.260	0.000	PA14_18350	3545.721	0.073	0.795
PA14_18360	247.024	-3.394	0.000	-3.518	0.000	-2.910	0.000	PA14_18360	2586.780	0.117	0.779
PA14_18370	319.114	-3.044	0.000	-3.467	0.000	-2.211	0.000	PA14_18370	5728.981	-0.707	0.001
PA14_18380	129.458	-0.594	0.165	-0.654	0.078	-0.256	0.650	PA14_18380	253.697	-0.949	0.001
PA14_18410	61.980	-0.045	0.956	0.016	0.978	0.253	0.734	PA14_18410	124.050	-0.233	0.533
PA14_18430	40.605	-0.563	0.376	0.026	0.966	0.161	0.842	PA14_18430	57.132	-0.238	0.606
PA14_18450	39.503	-0.350	0.572	-1.390	0.003	-0.234	0.749	PA14_18450	89.531	-0.707	0.053
PA14_18470	24.322	-0.908	0.163	-0.772	0.173	-0.871	0.272	PA14_18470	43.820	-0.041	0.946
PA14_18480	57.175	0.171	0.800	0.451	0.344	1.048	0.064	PA14_18480	89.447	-0.474	0.209
PA14_18500	58.341	-0.749	0.176	-0.501	0.310	0.801	0.179	PA14_18500	133.921	0.107	0.752
PA14_18510	18.939	-1.108	0.149	-1.275	0.056	-0.895	0.328	PA14_18510	50.681	-0.312	0.546
PA14_18520	28.710	0.324	0.675	-0.157	0.816	1.115	0.107	PA14_18520	40.251	0.710	0.170
PA14_18550	27.385	-0.711	0.285	-0.660	0.243	0.116	0.893	PA14_18550	50.317	0.466	0.343
PA14_18565	35.481	-0.162	0.837	-0.246	0.676	0.051	0.952	PA14_18565	66.654	-0.153	0.732
PA14_18580	81.766	-0.416	0.458	0.045	0.931	0.560	0.337	PA14_18580	102.109	0.144	0.723
PA14_18590	446.950	-0.271	0.460	0.046	0.896	-0.229	0.593	PA14_18590	1680.510	-1.152	0.000
PA14_18600	755.255	0.601	0.078	1.197	0.000	0.678	0.088	PA14_18600	1514.089	0.344	0.135
PA14_18610	1110.809	1.152	0.000	1.650	0.000	1.205	0.000	PA14_18610	1481.121	0.724	0.001
PA14_18620	365.363	1.283	0.007	1.742	0.000	1.458	0.008	PA14_18620	606.852	-0.688	0.040
PA14_18630	741.988	-1.363	0.000	-3.700	0.000	-3.060	0.000	PA14_18630	975.159	0.395	0.082
PA14_18640	125.972	-0.120	0.811	0.079	0.842	0.977	0.017	PA14_18640	263.261	0.005	0.981
PA14_18650	2190.707	-0.171	0.673	0.393	0.203	0.660	0.083	PA14_18650	6889.223	-1.653	0.000
PA14_18660	93.857	0.472	0.344	1.773	0.000	0.887	0.088	PA14_18660	202.191	0.685	0.044
PA14_18670	104977.510	0.392	0.489	0.890	0.047	1.440	0.008	PA14_18670	185181.531	-0.558	0.087
PA14_18680	109.839	-0.855	0.104	-0.816	0.079	1.796	0.001	PA14_18680	383.851	-3.593	0.000
PA14_18690	6712.421	0.016	0.976	-0.198	0.583	-1.366	0.000	PA14_18690	13541.136	0.588	0.005
PA14_18700	171.325	-0.361	0.416	-0.947	0.006	-0.466	0.346	PA14_18700	450.359	0.527	0.036
PA14_18710	1217.507	0.616	0.090	0.486	0.150	1.305	0.001	PA14_18710	2686.724	0.090	0.831
PA14_18720	189.796	-0.564	0.161	-1.261	0.000	0.315	0.529	PA14_18720	1515.858	-1.733	0.000
PA14_18740	3010.969	-0.061	0.881	0.242	0.398	0.739	0.026	PA14_18740	5760.619	1.009	0.000
PA14_18750	1576.308	0.671	0.016	0.492	0.064	-0.221	0.569	PA14_18750	4906.664	-0.595	0.008
PA14_18760	1255.830	-0.592	0.087	-2.127	0.000	-3.405	0.000	PA14_18760	5853.419	-2.220	0.000
PA14_18780	600.728	-0.664	0.082	-2.467	0.000	-2.322	0.000	PA14_18780	4908.902	-2.610	0.000
PA14_18790	118.800	-1.143	0.001	-2.485	0.000	-0.880	0.046	PA14_18790	1098.974	-4.640	0.000
PA14_18800	7726.217	-3.104	0.000	-4.949	0.000	2.064	0.000	PA14_18800	18088.736	-5.052	0.000
PA14_18810	5231.700	-0.084	0.876	-2.660	0.000	-0.007	0.990	PA14_18810	8408.546	0.271	0.492
PA14_18820	1103.548	0.093	0.837	-2.772	0.000	-0.201	0.657	PA14_18820	1474.781	1.864	0.000
PA14_18830	301.820	-0.534	0.113	-3.161	0.000	-0.102	0.832	PA14_18830	473.913	0.059	0.852
PA14_18850	989.380	-0.331	0.492	-3.014	0.000	0.712	0.147	PA14_18850	963.499	-1.025	0.000
PA14_18860	208.664	-0.514	0.141	-2.376	0.000	-0.567	0.167	PA14_18860	356.014	-0.695	0.007
PA14_18870	100.709	-0.027	0.971	-1.014	0.021	-2.637	0.000	PA14_18870	1300.926	-1.583	0.000
PA14_18880	936.925	0.551	0.073	0.290	0.330	-0.705	0.051	PA14_18880	1340.248	1.192	0.000
PA14_18890	328.867	0.289	0.506	-0.045	0.915	-0.248	0.626	PA14_18890	520.525	0.956	0.002
PA14_18900	99.448	0.037	0.946	0.124	0.750	-0.786	0.102	PA14_18900	138.563	0.751	0.020
PA14_18910	74.648	-0.353	0.501	-0.129	0.784	-1.277	0.021	PA14_18910	123.345	0.967	0.003
PA14_18920	307.646	0.581	0.099	0.373	0.263	-0.523	0.235	PA14_18920	540.145	1.442	0.000
PA14_18930	132.443	1.055	0.002	1.186	0.000	1.109	0.007	PA14_18930	296.610	1.570	0.000
PA14_18950	214.972	1.457	0.000	1.806	0.000	1.185	0.003	PA14_18950	466.523	1.350	0.000
PA14_18960	183.698	-0.354	0.417	-0.957	0.005	0.151	0.780	PA14_18960	384.816	-0.198	0.883
PA14_18970	720.501	-0.960	0.000	-1.575	0.000	-0.754	0.020	PA14_18970	3326.087	-1.637	0.000
PA14_18985	62.925	-0.548	0.250	-0.865	0.029	-0.654	0.245	PA14_18985	461.975	-0.773	0.001
PA14_19010	46.742	-0.053	0.941	-0.387	0.416	-0.363	0.599	PA14_19010	125.940	-0.620	0.056
PA14_19020	297.532	0.536	0.090	0.966	0.000	0.577	0.122	PA14_19020	371.500	0.489	0.059
PA14_19030	246.739	0.859	0.007	1.216	0.000	0.985	0.009	PA14_19030	306.612	0.678	0.010
PA14_19050	5829.602	0.580	0.016	0.509	0.025	0.367	0.233	PA14_19050	9426.994	1.239	0.000

PA14_19065	647.950	0.259	0.483	0.208	0.516	-0.385	0.341	PA14_19065	1346.645	0.306	0.189
PA14_19090	868.042	0.174	0.668	0.320	0.312	0.046	0.926	PA14_19090	2329.748	0.528	0.015
PA14_19100	2202.909	-2.426	0.000	-4.720	0.000	1.147	0.000	PA14_19100	16394.034	-4.417	0.000
PA14_19110	1403.217	-2.813	0.000	-4.741	0.000	0.667	0.056	PA14_19110	11974.327	-4.698	0.000
PA14_19120	3612.459	-2.065	0.000	-3.117	0.000	-0.825	0.010	PA14_19120	16741.818	-1.551	0.000
PA14_19130	827.081	-0.520	0.074	-1.333	0.000	0.180	0.648	PA14_19130	3417.387	-0.574	0.007
PA14_19140	438.372	0.328	0.399	-0.006	0.988	-0.038	0.942	PA14_19140	919.846	-0.015	0.927
PA14_19150	116.308	1.022	0.007	1.859	0.000	0.976	0.035	PA14_19150	175.115	1.305	0.000
PA14_19160	437.525	0.887	0.012	2.131	0.000	0.862	0.042	PA14_19160	387.691	0.611	0.016
PA14_19170	2881.410	1.357	0.000	2.014	0.000	3.236	0.000	PA14_19170	2174.885	0.683	0.002
PA14_19190	5235.147	1.540	0.000	0.218	0.407	-0.897	0.003	PA14_19190	4919.413	0.939	0.000
PA14_19205	71.313	0.581	0.219	0.497	0.235	-0.028	0.968	PA14_19205	91.113	1.312	0.000
PA14_19210	216.161	1.017	0.001	1.031	0.000	0.521	0.220	PA14_19210	295.765	1.315	0.000
PA14_19230	442.667	0.932	0.000	0.785	0.002	0.396	0.267	PA14_19230	377.341	1.431	0.000
PA14_19270	12.306	-0.242	0.832	-1.439	0.068	-0.111	0.926	PA14_19270	31.889	0.322	0.607
PA14_19290	1098.287	0.431	0.139	0.426	0.102	0.242	0.516	PA14_19290	1394.463	0.441	0.054
PA14_19310	792.931	-1.616	0.000	-2.897	0.000	-1.875	0.000	PA14_19310	4261.368	-1.122	0.000
PA14_19320	70.914	-0.417	0.430	-0.036	0.944	-0.443	0.471	PA14_19320	185.859	-0.202	0.516
PA14_19330	62.243	0.158	0.810	0.013	0.981	-0.901	0.157	PA14_19330	223.290	-0.287	0.321
PA14_19340	217.220	-1.279	0.000	-1.600	0.000	0.341	0.451	PA14_19340	823.266	-1.530	0.000
PA14_19350	543.815	-0.927	0.001	-1.254	0.000	1.844	0.000	PA14_19350	1594.288	-1.559	0.000
PA14_19360	1868.602	-0.547	0.034	-0.711	0.002	2.391	0.000	PA14_19360	3604.897	-1.061	0.000
PA14_19370	2029.212	-0.502	0.078	-0.919	0.000	1.893	0.000	PA14_19370	8428.064	-1.967	0.000
PA14_19380	788.672	-0.495	0.148	-2.100	0.000	-2.909	0.000	PA14_19380	4642.253	0.096	0.705
PA14_19390	39.428	0.633	0.319	1.031	0.043	0.263	0.751	PA14_19390	118.578	0.572	0.095
PA14_19400	118.143	0.057	0.916	0.103	0.793	-0.690	0.148	PA14_19400	269.076	0.352	0.222
PA14_19410	1291.920	-0.297	0.295	-1.393	0.000	-0.742	0.013	PA14_19410	4433.033	-1.117	0.000
PA14_19430	143.332	0.312	0.499	-0.069	0.874	0.653	0.165	PA14_19430	338.191	0.337	0.209
PA14_19450	116.688	-0.265	0.570	-0.025	0.954	-0.793	0.100	PA14_19450	203.474	1.240	0.000
PA14_19470	2341.408	2.911	0.000	3.219	0.000	4.304	0.000	PA14_19470	918.417	0.622	0.044
PA14_19480	687.174	-2.697	0.000	-3.556	0.000	2.646	0.000	PA14_19480	949.385	-3.718	0.000
PA14_19490	2210.514	-3.374	0.000	-2.995	0.000	-1.039	0.010	PA14_19490	1026.606	-1.753	0.000
PA14_19500	13.075	-2.172	0.018	-2.283	0.006	-0.283	0.810	PA14_19500	7.303	-0.604	0.764
PA14_19510	9.457	-1.138	0.219	-1.409	0.077	0.130	0.912	PA14_19510	15.608	0.026	1.000
PA14_19520	5.777	-1.269	0.288	-1.061	0.309	-1.292	0.288	PA14_19520	10.394	-1.371	0.172
PA14_19530	171.889	-3.637	0.000	-2.624	0.000	-2.325	0.000	PA14_19530	101.181	-1.900	0.013
PA14_19540	62.699	-3.640	0.000	-2.536	0.000	-1.926	0.000	PA14_19540	55.976	-1.383	0.009
PA14_19560	53.247	-3.552	0.000	-3.541	0.000	-2.232	0.000	PA14_19560	36.879	-0.839	0.123
PA14_19570	34.852	-2.252	0.000	-1.488	0.008	-0.736	0.344	PA14_19570	40.482	0.393	0.466
PA14_19580	148.442	-2.821	0.000	-2.138	0.000	-1.585	0.001	PA14_19580	207.329	-1.532	0.000
PA14_19590	192.398	-2.714	0.000	-1.407	0.000	-1.789	0.000	PA14_19590	249.474	-1.462	0.000
PA14_19600	419.647	0.982	0.000	0.968	0.000	-0.123	0.782	PA14_19600	970.339	0.658	0.004
PA14_19610	5526.914	0.768	0.062	1.036	0.005	-0.041	0.946	PA14_19610	9487.413	0.039	0.899
PA14_19620	1022.787	0.083	0.835	-0.132	0.663	-0.385	0.294	PA14_19620	3028.772	-0.147	0.549
PA14_19630	1365.411	0.017	0.974	-0.128	0.717	0.031	0.950	PA14_19630	3187.408	-0.224	0.325
PA14_19640	469.160	0.574	0.082	0.812	0.005	0.247	0.577	PA14_19640	1129.716	-0.300	0.195
PA14_19650	18.653	1.387	0.130	3.387	0.000	1.483	0.140	PA14_19650	58.000	2.214	0.000
PA14_19660	1023.657	-0.064	0.920	-0.463	0.276	-0.180	0.771	PA14_19660	1304.034	1.075	0.002
PA14_19670	37.557	0.010	0.989	0.177	0.751	0.144	0.859	PA14_19670	97.248	0.432	0.260
PA14_19680	258.643	1.649	0.000	2.809	0.000	2.059	0.000	PA14_19680	525.644	-2.416	0.000
PA14_19690	114.976	1.877	0.000	2.517	0.000	1.779	0.000	PA14_19690	246.648	-1.324	0.000
PA14_19700	287.502	0.117	0.789	1.022	0.000	2.221	0.000	PA14_19700	937.219	-2.113	0.000
PA14_19710	84.388	0.937	0.055	1.622	0.000	2.520	0.000	PA14_19710	297.796	-1.357	0.000
PA14_19720	13.796	-0.028	0.981	0.692	0.354	2.177	0.006	PA14_19720	48.901	-1.106	0.017
PA14_19730	146.733	0.309	0.471	0.014	0.973	1.691	0.000	PA14_19730	377.373	-0.248	0.356
PA14_19740	180.592	-1.070	0.001	-1.384	0.000	0.782	0.044	PA14_19740	447.250	-1.065	0.000
PA14_19750	46.897	0.759	0.131	-1.084	0.021	-0.530	0.435	PA14_19750	40.439	-0.739	0.140
PA14_19770	142.856	1.688	0.000	0.365	0.325	0.825	0.066	PA14_19770	100.048	-0.440	0.233
PA14_19800	215.285	0.125	0.799	-0.024	0.955	-1.912	0.000	PA14_19800	723.320	-0.171	0.655

PA14_19810	76.981	-0.226	0.693	-0.100	0.836	1.374	0.004	PA14_19810	146.994	-0.810	0.010
PA14_19830	57.830	-0.201	0.737	-0.947	0.027	0.340	0.578	PA14_19830	226.962	-0.594	0.036
PA14_19850	65.917	1.528	0.001	2.249	0.000	2.893	0.000	PA14_19850	130.927	0.452	0.179
PA14_19860	55.597	-0.951	0.044	-1.092	0.009	-1.494	0.011	PA14_19860	254.146	-0.537	0.049
PA14_19870	654.416	-2.352	0.000	-3.541	0.000	2.066	0.000	PA14_19870	5823.824	-4.548	0.000
PA14_19900	256.833	-2.100	0.000	-2.627	0.000	3.881	0.000	PA14_19900	4090.846	-4.393	0.000
PA14_19910	498.759	-1.652	0.001	-2.020	0.000	4.981	0.000	PA14_19910	2369.700	-3.479	0.000
PA14_19920	120.114	-1.435	0.007	-2.154	0.000	4.676	0.000	PA14_19920	879.535	-3.386	0.000
PA14_19930	19.156	1.313	0.078	2.247	0.000	1.792	0.024	PA14_19930	52.375	0.490	0.553
PA14_19940	66.312	0.548	0.277	0.387	0.389	0.567	0.331	PA14_19940	217.581	-0.957	0.001
PA14_19950	1364.086	0.371	0.270	0.310	0.302	-0.200	0.639	PA14_19950	6317.920	-1.292	0.000
PA14_19960	16.549	0.355	0.722	1.095	0.111	1.454	0.084	PA14_19960	45.191	-0.811	0.282
PA14_19970	16.417	0.213	0.868	2.895	0.000	2.718	0.002	PA14_19970	20.635	2.513	0.001
PA14_19990	218.303	0.381	0.352	2.460	0.000	2.335	0.000	PA14_19990	117.399	1.103	0.001
PA14_20000	30.625	-0.662	0.402	0.423	0.514	1.910	0.005	PA14_20000	35.072	0.002	1.000
PA14_20010	73.936	-0.291	0.604	0.101	0.834	2.496	0.000	PA14_20010	60.030	0.082	0.918
PA14_20020	94.231	-1.427	0.009	-0.485	0.315	4.434	0.000	PA14_20020	25.185	0.408	0.531
PA14_20030	8.779	-0.908	0.416	-0.327	0.740	1.694	0.079	PA14_20030	9.859	0.291	0.818
PA14_20040	9.893	-0.204	0.885	0.724	0.440	1.417	0.178	PA14_20040	11.867	0.456	0.708
PA14_20050	30.585	0.143	0.857	-0.197	0.745	1.666	0.005	PA14_20050	75.198	0.072	0.897
PA14_20060	48.543	-0.594	0.278	-0.332	0.494	1.264	0.016	PA14_20060	409.176	-1.390	0.000
PA14_20070	53.384	0.509	0.349	1.383	0.001	-0.053	0.945	PA14_20070	88.978	0.675	0.072
PA14_20080	141.618	0.138	0.804	0.236	0.572	0.230	0.689	PA14_20080	379.622	0.522	0.050
PA14_20100	62.292	-0.001	0.999	-0.184	0.685	-1.042	0.071	PA14_20100	108.680	1.146	0.001
PA14_20110	163.184	0.093	0.848	-0.130	0.724	-0.588	0.190	PA14_20110	275.588	-0.502	0.062
PA14_20120	151.989	-1.246	0.006	-1.564	0.000	-1.830	0.001	PA14_20120	605.197	-1.658	0.000
PA14_20130	116.583	-0.373	0.475	-0.206	0.651	-0.674	0.228	PA14_20130	327.183	0.526	0.044
PA14_20140	19070.468	0.377	0.724	1.324	0.088	2.178	0.015	PA14_20140	30583.636	-2.376	0.001
PA14_20150	4.808	0.833	0.546	0.497	0.682	2.856	0.010	PA14_20150	30.327	-3.155	0.000
PA14_20170	32.261	0.671	0.435	0.536	0.467	4.608	0.000	PA14_20170	64.468	-3.022	0.000
PA14_20180	68.071	0.070	0.938	-1.835	0.006	5.147	0.000	PA14_20180	114.377	-4.684	0.000
PA14_20190	121.204	1.512	0.005	-0.607	0.327	5.875	0.000	PA14_20190	357.616	-4.507	0.000
PA14_20200	682.997	1.987	0.000	-1.272	0.000	5.754	0.000	PA14_20200	4528.008	-6.054	0.000
PA14_20230	1702.450	2.589	0.000	-1.538	0.000	6.373	0.000	PA14_20230	10512.885	-6.920	0.000
PA14_20240	270.729	5.416	0.000	1.853	0.000	0.680	0.235	PA14_20240	65.125	-0.267	0.565
PA14_20250	73.115	4.671	0.000	0.895	0.136	0.971	0.221	PA14_20250	38.179	0.201	0.767
PA14_20260	44.032	0.761	0.187	1.573	0.001	2.261	0.000	PA14_20260	64.522	1.460	0.000
PA14_20270	50.905	0.367	0.572	1.251	0.006	2.789	0.000	PA14_20270	86.565	0.522	0.176
PA14_20280	52.978	0.492	0.450	0.924	0.064	3.260	0.000	PA14_20280	250.541	-0.058	0.923
PA14_20290	4664.692	-1.010	0.001	-1.002	0.000	1.044	0.003	PA14_20290	23850.327	-1.248	0.000
PA14_20300	54.835	-1.085	0.048	-0.937	0.057	-1.013	0.126	PA14_20300	624.578	-3.548	0.000
PA14_20320	68.261	-0.380	0.470	-0.618	0.139	0.250	0.684	PA14_20320	2984.548	-4.918	0.000
PA14_20330	8.742	-0.098	0.941	-0.523	0.569	0.986	0.335	PA14_20330	298.842	-4.108	0.000
PA14_20350	10.573	-0.089	0.947	0.270	0.775	0.334	0.783	PA14_20350	159.484	-3.172	0.000
PA14_20360	7.244	-0.328	0.808	0.446	0.654	0.252	0.851	PA14_20360	116.472	-3.548	0.000
PA14_20370	4.806	-0.277	0.848	-1.925	0.061	-0.103	0.943	PA14_20370	82.907	-3.259	0.000
PA14_20380	14.456	0.262	0.792	0.077	0.927	1.046	0.222	PA14_20380	113.591	-3.071	0.000
PA14_20390	19.545	-1.001	0.233	-1.173	0.101	0.563	0.551	PA14_20390	246.961	-3.402	0.000
PA14_20400	7.008	0.064	0.966	0.115	0.912	0.020	0.988	PA14_20400	122.454	-3.471	0.000
PA14_20420	11.522	0.162	0.885	-0.181	0.834	0.428	0.684	PA14_20420	120.717	-3.174	0.000
PA14_20430	110.317	0.307	0.574	0.058	0.910	0.509	0.372	PA14_20430	548.037	-1.869	0.000
PA14_20440	100.595	0.171	0.719	-0.300	0.412	-0.369	0.470	PA14_20440	555.875	-1.449	0.000
PA14_20450	76.660	0.140	0.806	-0.019	0.969	0.592	0.249	PA14_20450	244.910	-0.751	0.006
PA14_20460	70.506	-0.726	0.231	-1.622	0.001	1.363	0.024	PA14_20460	58.574	0.204	0.684
PA14_20470	103.937	-1.659	0.000	-2.821	0.000	0.808	0.053	PA14_20470	170.472	-1.817	0.000
PA14_20480	2604.259	-0.549	0.036	-2.642	0.000	1.783	0.000	PA14_20480	1761.268	-1.876	0.000
PA14_20491	54.059	-0.106	0.876	-0.003	0.995	0.334	0.603	PA14_20491	184.521	-1.402	0.000
PA14_20500	41.958	-0.943	0.157	0.617	0.283	0.093	0.918	#N/A	#N/A	#N/A	#N/A
PA14_20510	3299.540	0.502	0.110	0.298	0.320	1.402	0.000	PA14_20510	6644.072	-0.029	0.920

PA14_20520	2056.961	-0.705	0.002	-1.841	0.000	-1.441	0.000	PA14_20520	12388.977	0.208	0.333
PA14_20530	3305.587	-0.313	0.462	-0.857	0.010	-0.724	0.095	PA14_20530	12773.926	0.735	0.001
PA14_20550	113.981	-0.010	0.987	-0.538	0.183	1.168	0.012	PA14_20550	188.876	-0.265	0.481
PA14_20560	785.375	-0.344	0.357	-0.237	0.476	1.728	0.000	PA14_20560	1212.150	-1.840	0.000
PA14_20570	277.859	0.847	0.031	0.692	0.058	1.608	0.000	PA14_20570	303.222	-1.194	0.000
PA14_20580	136.534	0.290	0.522	0.205	0.598	1.326	0.001	PA14_20580	268.092	-0.793	0.003
PA14_20590	61.516	-0.706	0.233	-1.375	0.005	0.237	0.751	PA14_20590	206.401	-1.123	0.000
PA14_20600	432.512	-0.053	0.902	0.043	0.897	-0.697	0.050	PA14_20600	1111.613	-0.782	0.000
PA14_20610	530.583	-4.290	0.000	-5.359	0.000	1.349	0.000	PA14_20610	1361.866	-4.953	0.000
PA14_20620	82.219	1.321	0.002	3.059	0.000	-0.195	0.796	PA14_20620	36.415	0.651	0.240
PA14_20630	86.335	1.518	0.001	2.510	0.000	-0.030	0.970	PA14_20630	64.695	0.856	0.040
PA14_20640	158.562	0.093	0.866	0.551	0.126	-0.223	0.691	PA14_20640	177.862	0.322	0.494
PA14_20650	152.825	-0.089	0.850	-0.566	0.071	-0.826	0.050	PA14_20650	225.226	0.763	0.006
PA14_20670	1145.786	0.920	0.000	-0.154	0.601	-0.087	0.839	PA14_20670	2148.004	0.428	0.053
PA14_20680	432.839	0.217	0.572	-0.203	0.532	0.038	0.937	PA14_20680	1113.711	-0.378	0.100
PA14_20690	137.442	-0.167	0.749	-0.361	0.356	0.639	0.182	PA14_20690	461.612	-1.095	0.000
PA14_20700	963.899	-0.043	0.928	-0.573	0.060	1.067	0.003	PA14_20700	2762.618	-1.082	0.000
PA14_20720	3027.021	0.112	0.757	-0.818	0.001	1.037	0.001	PA14_20720	9055.376	-1.209	0.000
PA14_20730	4814.993	-0.010	0.983	-1.182	0.000	0.368	0.368	PA14_20730	17315.256	-1.670	0.000
PA14_20740	1121.096	0.865	0.008	1.579	0.000	2.023	0.000	PA14_20740	1566.174	-0.062	0.844
PA14_20750	7273.699	-0.260	0.534	-0.715	0.027	0.918	0.022	PA14_20750	19739.267	-0.931	0.014
PA14_20760	616.244	-0.465	0.165	-1.506	0.000	-0.146	0.747	PA14_20760	3025.234	-0.748	0.000
PA14_20770	1080.395	-1.355	0.000	-2.145	0.000	-0.858	0.062	PA14_20770	5425.448	-2.083	0.000
PA14_20780	944.676	-1.340	0.000	-1.683	0.000	-0.097	0.839	PA14_20780	4461.125	-1.342	0.000
PA14_20800	127.098	-0.039	0.952	0.291	0.504	-0.364	0.542	PA14_20800	396.557	0.285	0.315
PA14_20810	1005.382	0.796	0.003	1.166	0.000	1.377	0.000	PA14_20810	1216.075	1.352	0.000
PA14_20820	267.548	0.240	0.567	0.117	0.752	0.530	0.212	PA14_20820	1397.137	-0.923	0.000
PA14_20840	159.742	-0.805	0.018	-1.530	0.000	-0.589	0.167	PA14_20840	1010.825	-1.131	0.000
PA14_20850	389.557	0.395	0.246	0.040	0.911	-0.119	0.796	PA14_20850	1224.312	-1.066	0.000
PA14_20860	1736.968	-0.651	0.179	0.007	0.991	1.230	0.018	PA14_20860	5837.479	-3.761	0.000
PA14_20870	195.347	0.142	0.728	0.256	0.406	0.481	0.215	PA14_20870	274.722	1.377	0.000
PA14_20880	54.067	0.549	0.339	0.657	0.167	0.627	0.332	PA14_20880	171.480	0.240	0.463
PA14_20890	2256.605	-1.263	0.000	-2.524	0.000	-4.058	0.000	PA14_20890	9232.018	0.135	0.565
PA14_20900	203.423	-1.499	0.000	-4.318	0.000	-2.717	0.000	PA14_20900	1176.910	-1.809	0.000
PA14_20920	405.049	-0.622	0.037	-3.883	0.000	-1.766	0.000	PA14_20920	2287.898	-2.102	0.000
PA14_20940	649.685	-1.143	0.000	-5.289	0.000	-3.754	0.000	PA14_20940	8368.810	-2.578	0.000
PA14_20950	2445.822	-1.268	0.000	-6.121	0.000	-3.589	0.000	PA14_20950	14514.705	-2.664	0.000
PA14_20960	556.058	-1.262	0.000	-5.688	0.000	-4.313	0.000	PA14_20960	4713.160	-2.307	0.000
PA14_20970	1619.613	-0.910	0.001	-5.472	0.000	-3.340	0.000	PA14_20970	9617.350	-2.135	0.000
PA14_20980	682.326	-0.844	0.015	-5.320	0.000	-3.933	0.000	PA14_20980	5208.129	-2.507	0.000
PA14_21000	547.300	-0.934	0.001	-6.121	0.000	-4.252	0.000	PA14_21000	4639.727	-2.390	0.000
PA14_21010	337.539	-0.755	0.018	-6.409	0.000	-4.547	0.000	PA14_21010	2754.739	-2.323	0.000
PA14_21020	2350.866	-0.695	0.014	-5.548	0.000	-3.456	0.000	PA14_21020	8103.574	-1.515	0.000
PA14_21030	7555.409	-1.545	0.000	-4.558	0.000	-1.019	0.000	PA14_21030	17506.992	-1.431	0.000
PA14_21040	30.676	-0.824	0.195	-1.308	0.017	0.470	0.526	PA14_21040	166.821	0.186	0.564
PA14_21050	33.488	-0.753	0.207	-0.258	0.639	-0.485	0.518	PA14_21050	193.647	-0.171	0.585
PA14_21060	30.381	-0.168	0.834	0.329	0.561	-0.374	0.652	PA14_21060	192.288	-1.256	0.000
PA14_21070	158.302	0.623	0.063	1.049	0.000	-0.652	0.144	PA14_21070	318.438	0.505	0.063
PA14_21080	74.736	0.676	0.148	0.755	0.063	-1.101	0.075	PA14_21080	174.989	0.359	0.363
PA14_21090	68.784	0.900	0.176	2.232	0.000	1.392	0.049	PA14_21090	125.894	-0.280	0.780
PA14_21110	74.845	0.041	0.951	0.352	0.407	0.684	0.196	PA14_21110	430.183	-2.063	0.000
PA14_21120	61.419	-0.129	0.838	0.205	0.655	1.351	0.005	PA14_21120	207.277	-0.750	0.011
PA14_21130	65.459	-0.049	0.942	-0.250	0.582	0.452	0.428	PA14_21130	412.161	-0.909	0.000
PA14_21140	83.247	0.129	0.833	0.658	0.099	-0.720	0.221	PA14_21140	83.754	-0.710	0.055
PA14_21150	33.719	0.251	0.726	0.325	0.559	-1.152	0.132	PA14_21150	46.642	0.878	0.065
PA14_21160	33.380	-0.358	0.652	-0.334	0.605	-1.791	0.026	PA14_21160	84.820	-0.307	0.457
PA14_21175	867.278	-1.451	0.000	-1.484	0.000	-2.728	0.000	PA14_21175	1075.952	-0.591	0.009
PA14_21180	82.382	0.523	0.285	0.929	0.019	-0.101	0.885	PA14_21180	133.787	1.112	0.001
PA14_21190	275.905	-2.539	0.000	-3.445	0.000	1.178	0.000	PA14_21190	1865.148	-1.349	0.000

PA14_21210	257.671	-0.504	0.113	-0.458	0.106	0.688	0.050	PA14_21210	507.236	0.356	0.322
PA14_21220	6664.873	-1.925	0.000	-3.834	0.000	-4.513	0.000	PA14_21220	68239.812	-0.489	0.096
PA14_21230	1941.559	0.857	0.003	1.253	0.000	1.118	0.001	PA14_21230	1931.032	1.721	0.000
PA14_21240	667.621	-0.485	0.292	-0.274	0.520	1.158	0.014	PA14_21240	2266.850	-2.227	0.000
PA14_21250	406.995	-0.028	0.960	-0.314	0.374	0.400	0.380	PA14_21250	2565.078	-1.567	0.000
PA14_21260	34865.374	-1.392	0.000	-2.442	0.000	-5.467	0.000	PA14_21260	138235.233	-1.826	0.000
PA14_21280	698.015	1.758	0.000	2.780	0.000	1.628	0.000	PA14_21280	1042.486	0.327	0.187
PA14_21290	461.823	1.087	0.000	2.011	0.000	0.900	0.010	PA14_21290	850.544	1.109	0.000
PA14_21300	63.642	0.172	0.766	0.397	0.342	0.234	0.709	PA14_21300	152.659	-0.642	0.035
PA14_21310	270.354	-0.511	0.214	-1.298	0.000	-1.533	0.001	PA14_21310	733.593	-0.169	0.517
PA14_21320	746.026	0.424	0.253	0.286	0.399	0.458	0.282	PA14_21320	1210.859	-0.182	0.457
PA14_21340	1211.395	-0.325	0.265	-1.412	0.000	-0.874	0.004	PA14_21340	2197.554	-0.753	0.000
PA14_21370	2447.994	-0.194	0.556	-1.174	0.000	-1.474	0.000	PA14_21370	6294.921	-0.127	0.610
PA14_21380	33.245	1.266	0.028	1.880	0.000	0.735	0.330	PA14_21380	29.571	1.157	0.051
PA14_21400	1275.599	0.605	0.019	0.922	0.000	0.197	0.586	PA14_21400	1951.882	1.034	0.000
PA14_21410	13.717	-0.133	0.905	-0.302	0.707	0.956	0.279	PA14_21410	288.656	-4.019	0.000
PA14_21440	537.185	-0.038	0.949	-0.538	0.162	-0.819	0.091	PA14_21440	1627.851	-0.670	0.003
PA14_21450	45.133	0.037	0.962	0.297	0.541	-0.638	0.344	PA14_21450	364.331	-0.478	0.065
PA14_21460	17.482	-0.504	0.550	-0.341	0.629	-0.375	0.700	PA14_21460	66.999	-1.166	0.004
PA14_21470	56.807	-0.955	0.032	-2.125	0.000	-1.293	0.018	PA14_21470	219.405	-2.265	0.000
PA14_21480	332.443	-0.235	0.655	-1.117	0.003	-0.724	0.157	PA14_21480	762.552	-2.041	0.000
PA14_21490	2068.164	0.245	0.703	-0.698	0.144	-0.265	0.703	PA14_21490	11995.996	-1.442	0.000
PA14_21510	35.216	-1.959	0.000	-1.886	0.000	-0.234	0.754	PA14_21510	167.826	-2.626	0.000
PA14_21520	46.262	-0.127	0.870	0.526	0.305	0.113	0.890	PA14_21520	136.904	0.104	0.796
PA14_21530	55.538	-1.252	0.008	-1.625	0.000	-0.072	0.918	PA14_21530	5103.077	-7.059	0.000
PA14_21540	381.530	-0.001	0.999	-0.552	0.162	-0.107	0.863	PA14_21540	892.655	0.097	0.665
PA14_21550	266.454	0.007	0.989	-0.287	0.490	1.548	0.000	PA14_21550	537.573	-0.081	0.777
PA14_21560	11.802	0.398	0.692	0.018	0.983	0.509	0.624	PA14_21560	62.422	-0.408	0.345
PA14_21570	91.850	1.382	0.001	2.136	0.000	-0.457	0.498	PA14_21570	124.932	1.712	0.000
PA14_21580	100.889	0.950	0.026	2.347	0.000	1.419	0.003	PA14_21580	94.387	0.441	0.262
PA14_21590	40.471	0.950	0.105	2.306	0.000	0.526	0.498	PA14_21590	46.548	0.877	0.072
PA14_21600	22.554	0.050	0.960	0.832	0.144	-0.401	0.665	PA14_21600	39.705	0.593	0.262
PA14_21610	51.352	0.654	0.190	0.838	0.047	1.170	0.027	PA14_21610	800.755	-3.327	0.000
PA14_21620	10.101	-0.704	0.478	-1.011	0.209	0.445	0.678	PA14_21620	69.619	-2.698	0.000
PA14_21630	231.821	0.424	0.486	-0.284	0.597	-3.679	0.000	PA14_21630	262.338	-0.676	0.120
PA14_21640	369.423	0.447	0.156	0.054	0.871	0.031	0.946	PA14_21640	823.557	-0.287	0.235
PA14_21650	76.462	0.771	0.177	0.622	0.231	1.045	0.098	PA14_21650	206.097	0.849	0.182
PA14_21660	13.556	-0.791	0.370	0.131	0.873	-0.629	0.535	PA14_21660	25.904	1.296	0.037
PA14_21670	64.764	-2.339	0.000	-3.303	0.000	0.522	0.417	PA14_21670	422.192	-3.161	0.000
PA14_21680	55.450	-0.887	0.075	-0.175	0.722	0.190	0.777	PA14_21680	197.462	-1.357	0.000
PA14_21690	381.269	0.032	0.947	0.838	0.003	-0.529	0.189	PA14_21690	959.825	0.915	0.000
PA14_21700	511.257	0.220	0.566	0.411	0.172	-0.224	0.611	PA14_21700	1071.035	0.083	0.779
PA14_21710	668.773	0.706	0.095	1.188	0.001	0.704	0.157	PA14_21710	1520.936	-0.904	0.000
PA14_21720	256.307	1.427	0.000	2.132	0.000	1.260	0.001	PA14_21720	590.615	0.214	0.455
PA14_21730	384.123	-1.508	0.000	-0.357	0.189	-2.091	0.000	PA14_21730	216.561	0.103	0.741
PA14_21750	234.679	-0.958	0.007	-0.003	0.995	0.084	0.876	PA14_21750	455.429	0.258	0.325
PA14_21760	304.425	-0.508	0.416	-0.279	0.619	-0.153	0.847	PA14_21760	492.526	0.579	0.022
PA14_21770	17.123	0.296	0.767	1.433	0.027	0.668	0.487	PA14_21770	62.337	0.941	0.027
PA14_21780	103.942	0.548	0.229	1.357	0.000	-0.443	0.460	PA14_21780	245.333	0.467	0.097
PA14_21790	1145.492	0.744	0.072	0.396	0.323	-1.666	0.000	PA14_21790	1983.113	1.521	0.000
PA14_21800	0.320	0.919	0.340	0.009	0.993	0.135	0.874	#N/A	#N/A	#N/A	#N/A
PA14_21810	16.646	-0.729	0.470	1.856	0.005	2.430	0.002	#N/A	#N/A	#N/A	#N/A
PA14_21820	3268.069	0.084	0.827	0.051	0.870	-0.912	0.004	PA14_21820	8649.725	0.495	0.019
PA14_21830	112.290	-0.769	0.046	-0.585	0.092	0.406	0.392	PA14_21830	520.876	-1.395	0.000
PA14_21840	1391.334	-0.710	0.139	-0.252	0.597	1.255	0.016	PA14_21840	3987.602	-1.495	0.000
PA14_21850	357.240	-0.242	0.538	-0.170	0.614	0.358	0.393	PA14_21850	2271.783	-1.787	0.000
PA14_21860	117.194	0.440	0.316	1.048	0.002	1.968	0.000	PA14_21860	407.420	-1.203	0.000
PA14_21870	103.089	0.089	0.876	0.937	0.006	0.971	0.030	PA14_21870	296.968	0.464	0.094
PA14_21880	9959.185	-0.614	0.020	-0.839	0.000	-0.379	0.257	PA14_21880	29039.556	-0.368	0.096

PA14_21890	491.611	-0.739	0.013	-1.132	0.000	-1.305	0.000	PA14_21890	1542.720	-0.733	0.001
PA14_21900	154.645	-0.634	0.200	-0.378	0.405	-1.283	0.020	PA14_21900	507.669	-0.452	0.172
PA14_21910	251.177	-0.147	0.726	0.323	0.296	-0.360	0.402	PA14_21910	704.963	-1.253	0.000
PA14_21920	39.089	-1.360	0.007	-0.562	0.232	0.444	0.481	PA14_21920	164.623	-2.239	0.000
PA14_21930	20.867	-1.436	0.041	-0.846	0.174	1.184	0.096	PA14_21930	97.998	-1.645	0.016
PA14_21940	110.664	-0.692	0.077	-0.581	0.096	0.971	0.020	PA14_21940	332.369	-1.725	0.000
PA14_21960	252.471	-1.687	0.000	-1.837	0.000	0.120	0.818	PA14_21960	2458.849	-4.086	0.000
PA14_21970	26.258	-1.376	0.033	-1.779	0.002	0.093	0.918	PA14_21970	106.040	-1.446	0.000
PA14_21980	124.560	0.160	0.761	0.514	0.169	-1.228	0.016	PA14_21980	295.006	0.012	1.000
PA14_21990	1004.903	0.426	0.154	0.862	0.001	0.027	0.950	PA14_21990	1858.435	-0.333	0.158
PA14_22000	826.224	0.527	0.047	0.977	0.000	-0.134	0.726	PA14_22000	1181.381	1.548	0.000
PA14_22010	406.206	0.169	0.759	0.466	0.252	-1.506	0.002	PA14_22010	1851.571	0.328	0.156
PA14_22020	3987.289	0.197	0.643	0.388	0.242	-0.176	0.714	PA14_22020	8623.789	0.263	0.382
PA14_22040	1267.980	0.588	0.066	1.088	0.000	-0.237	0.585	PA14_22040	3124.425	0.049	0.873
PA14_22050	433.969	0.436	0.298	1.496	0.000	2.011	0.000	PA14_22050	718.602	1.749	0.000
PA14_22060	749.377	-0.570	0.054	-0.490	0.072	-0.486	0.178	PA14_22060	1754.520	-0.795	0.000
PA14_22075	440.078	0.149	0.704	0.713	0.009	-0.566	0.135	PA14_22075	844.258	-0.184	0.482
PA14_22080	1398.951	1.716	0.000	1.510	0.000	-0.054	0.900	PA14_22080	3221.411	1.240	0.000
PA14_22090	301.614	-0.297	0.420	0.280	0.361	2.090	0.000	PA14_22090	652.974	-0.735	0.021
PA14_22100	144.883	0.283	0.581	-0.738	0.064	1.745	0.000	PA14_22100	177.216	0.356	0.300
PA14_22110	190.801	-0.426	0.251	-1.379	0.000	1.314	0.000	PA14_22110	348.195	-0.198	0.706
PA14_22120	372.865	-0.014	0.976	-1.065	0.000	0.300	0.454	PA14_22120	1145.540	-1.066	0.000
PA14_22130	80.914	-0.753	0.094	-2.361	0.000	0.667	0.192	PA14_22130	89.932	0.557	0.129
PA14_22140	166.873	-0.062	0.920	-0.494	0.223	1.110	0.018	PA14_22140	245.229	-0.269	0.383
PA14_22160	38.230	-0.218	0.755	-0.570	0.268	0.824	0.183	PA14_22160	73.188	0.134	0.839
PA14_22180	143.359	2.090	0.000	2.112	0.000	1.022	0.078	PA14_22180	188.949	-0.250	0.462
PA14_22190	577.449	0.495	0.104	0.852	0.001	1.350	0.000	PA14_22190	1336.616	-0.432	0.059
PA14_22210	112.742	-0.090	0.875	0.184	0.651	1.804	0.000	PA14_22210	211.948	0.674	0.018
PA14_22220	149.886	-0.535	0.155	-1.305	0.000	0.891	0.024	PA14_22220	274.118	0.771	0.003
PA14_22230	257.749	0.129	0.784	0.193	0.594	0.179	0.722	PA14_22230	449.206	0.749	0.140
PA14_22240	143.933	-0.582	0.142	-0.814	0.017	1.289	0.001	PA14_22240	203.555	0.616	0.057
PA14_22250	210.495	0.038	0.939	0.276	0.403	1.670	0.000	PA14_22250	291.642	0.289	0.296
PA14_22260	161.736	0.289	0.544	0.961	0.005	3.015	0.000	PA14_22260	102.796	0.917	0.007
PA14_22270	2158.432	0.290	0.446	0.854	0.004	1.025	0.005	PA14_22270	4773.184	0.381	0.369
PA14_22280	116.711	-0.083	0.888	-0.338	0.405	1.162	0.010	PA14_22280	324.626	-0.291	0.323
PA14_22290	517.148	0.282	0.538	0.437	0.235	1.507	0.000	PA14_22290	1058.481	-0.373	0.125
PA14_22310	961.705	0.043	0.924	-0.413	0.156	0.313	0.431	PA14_22310	2936.120	-0.656	0.053
PA14_22320	6.156	-1.086	0.347	-0.559	0.581	0.977	0.384	PA14_22320	548.149	-7.555	0.000
PA14_22330	232.156	1.278	0.000	0.407	0.169	0.879	0.014	PA14_22330	458.539	-0.097	0.740
PA14_22340	411.926	-0.197	0.598	-0.675	0.015	-0.599	0.109	PA14_22340	815.495	2.382	0.000
PA14_22350	207.645	0.260	0.631	-0.852	0.036	-0.233	0.706	PA14_22350	322.889	0.138	0.634
PA14_22370	338.073	0.801	0.064	0.402	0.339	-0.178	0.774	PA14_22370	810.077	0.103	0.736
PA14_22380	83.651	1.048	0.059	0.265	0.647	0.059	0.944	PA14_22380	235.781	0.830	0.003
PA14_22400	125.027	-1.211	0.003	-2.399	0.000	1.399	0.001	PA14_22400	479.470	-3.038	0.000
PA14_22410	435.843	0.568	0.049	1.138	0.000	-0.195	0.635	PA14_22410	1150.570	0.252	0.512
PA14_22420	31.422	-0.326	0.640	0.041	0.948	0.049	0.954	PA14_22420	121.437	-1.174	0.000
PA14_22440	716.054	0.340	0.268	0.066	0.831	0.916	0.004	PA14_22440	1446.476	1.308	0.000
PA14_22450	592.817	-0.057	0.919	-0.807	0.020	-0.188	0.727	PA14_22450	2729.890	1.847	0.001
PA14_22460	942.549	0.332	0.300	0.173	0.564	1.293	0.000	PA14_22460	3860.241	1.050	0.000
PA14_22470	369.758	0.001	0.998	-0.390	0.178	0.417	0.270	PA14_22470	1665.197	-0.410	0.067
PA14_22480	533.687	-0.511	0.127	-0.623	0.035	-0.479	0.233	PA14_22480	1657.218	-0.192	0.617
PA14_22490	126.948	1.157	0.004	1.459	0.000	1.126	0.020	PA14_22490	219.414	0.416	0.152
PA14_22500	43.090	0.570	0.319	0.648	0.174	1.052	0.075	PA14_22500	117.007	0.736	0.204
PA14_22510	15.672	0.209	0.834	0.210	0.782	0.712	0.423	PA14_22510	52.003	0.549	0.230
PA14_22520	77.310	0.284	0.639	0.789	0.073	0.350	0.598	PA14_22520	185.296	0.431	0.401
PA14_22530	124.838	1.045	0.009	1.031	0.005	0.797	0.112	PA14_22530	256.244	0.546	0.100
PA14_22540	269.030	1.128	0.003	0.671	0.070	-0.030	0.963	PA14_22540	434.914	-0.633	0.140
PA14_22550	403.140	1.101	0.000	1.751	0.000	-0.078	0.869	PA14_22550	654.144	0.699	0.003
PA14_22560	47.706	-0.537	0.337	-0.373	0.441	-1.968	0.003	PA14_22560	222.706	-1.059	0.000

PA14_22570	131.434	-0.675	0.130	-1.310	0.001	-2.832	0.000	PA14_22570	1166.488	-1.981	0.000
PA14_22580	731.679	1.523	0.000	1.854	0.000	1.127	0.037	PA14_22580	1457.609	-0.417	0.282
PA14_22590	27.742	-0.626	0.422	0.035	0.963	0.258	0.779	PA14_22590	125.022	-1.665	0.003
PA14_22600	24.406	1.033	0.139	2.075	0.000	1.184	0.132	PA14_22600	90.098	-1.327	0.000
PA14_22620	539.808	0.551	0.052	0.853	0.001	0.559	0.099	PA14_22620	1122.483	0.930	0.000
PA14_22630	64.509	-0.588	0.244	-0.943	0.025	0.732	0.171	PA14_22630	360.706	-1.490	0.000
PA14_22640	389.456	1.223	0.000	1.782	0.000	1.526	0.000	PA14_22640	382.054	0.651	0.011
PA14_22650	163.898	0.294	0.435	-0.139	0.685	-1.804	0.000	PA14_22650	401.690	-0.090	0.722
PA14_22660	308.372	-0.193	0.618	-0.620	0.031	-2.368	0.000	PA14_22660	646.876	0.765	0.001
PA14_22670	141.269	-0.399	0.281	-0.864	0.004	-1.698	0.000	PA14_22670	479.735	0.416	0.113
PA14_22680	528.393	0.088	0.822	-0.209	0.467	-1.289	0.000	PA14_22680	1740.527	0.157	0.540
PA14_22690	370.803	0.295	0.402	0.850	0.002	-0.065	0.891	PA14_22690	706.099	1.368	0.000
PA14_22700	13.284	-0.476	0.641	0.492	0.536	0.146	0.899	PA14_22700	33.770	2.170	0.000
PA14_22710	181.061	-0.192	0.630	-0.004	0.992	-0.868	0.030	PA14_22710	276.378	0.518	0.096
PA14_22720	55.550	0.472	0.338	-0.384	0.386	0.392	0.508	PA14_22720	256.242	1.538	0.000
PA14_22730	192.677	0.519	0.137	0.201	0.557	0.584	0.152	PA14_22730	680.761	0.793	0.001
PA14_22740	469.917	0.435	0.151	0.810	0.001	-0.209	0.605	PA14_22740	1022.966	-0.676	0.003
PA14_22760	280.692	0.356	0.303	0.198	0.533	0.394	0.323	PA14_22760	664.002	0.588	0.013
PA14_22770	375.480	0.144	0.724	0.169	0.604	-0.245	0.575	PA14_22770	862.709	0.519	0.024
PA14_22780	174.333	0.074	0.890	0.004	0.993	-0.497	0.307	PA14_22780	1104.541	0.958	0.000
PA14_22800	863.795	-0.079	0.844	0.074	0.814	-0.521	0.140	PA14_22800	3014.695	0.505	0.019
PA14_22820	400.411	0.287	0.463	0.727	0.016	1.281	0.000	PA14_22820	901.396	-0.323	0.178
PA14_22830	677.282	0.080	0.834	-0.144	0.612	0.497	0.134	PA14_22830	1482.099	0.212	0.346
PA14_22840	508.100	-0.246	0.467	-0.327	0.238	0.050	0.909	PA14_22840	1249.677	1.087	0.000
PA14_22860	1129.365	1.336	0.001	1.815	0.000	2.424	0.000	PA14_22860	1097.105	1.065	0.005
PA14_22870	205.505	0.358	0.377	0.675	0.036	0.662	0.121	PA14_22870	388.457	1.369	0.000
PA14_22880	59.581	0.462	0.460	1.209	0.009	1.617	0.005	PA14_22880	223.520	-1.663	0.000
PA14_22890	1668.989	3.889	0.000	3.951	0.000	3.453	0.000	PA14_22890	2508.542	3.966	0.000
PA14_22910	2334.198	3.240	0.000	3.442	0.000	2.464	0.000	PA14_22910	3141.118	2.878	0.000
PA14_22930	866.285	2.184	0.000	2.634	0.000	1.457	0.000	PA14_22930	1020.859	2.136	0.000
PA14_22940	465.060	2.746	0.000	3.181	0.000	0.535	0.224	PA14_22940	413.021	2.975	0.000
PA14_22960	747.453	1.900	0.000	2.085	0.000	0.438	0.334	PA14_22960	740.762	2.580	0.000
PA14_22980	5266.437	3.789	0.000	-0.985	0.000	3.931	0.000	PA14_22980	1731.347	-2.293	0.000
PA14_22990	326.053	4.882	0.000	-1.080	0.026	3.714	0.000	PA14_22990	127.489	-3.141	0.000
PA14_23000	1162.388	5.626	0.000	-0.419	0.198	4.475	0.000	PA14_23000	264.071	-2.134	0.000
PA14_23010	3586.433	5.597	0.000	-0.442	0.161	4.767	0.000	PA14_23010	771.595	-2.419	0.000
PA14_23030	5147.452	5.817	0.000	-1.354	0.000	4.700	0.000	PA14_23030	2209.776	-1.663	0.000
PA14_23050	2186.677	0.996	0.000	1.268	0.000	1.361	0.000	PA14_23050	4731.450	0.152	0.534
PA14_23060	432.423	1.029	0.002	1.318	0.000	0.367	0.435	PA14_23060	879.555	0.844	0.000
PA14_23070	4383.130	3.931	0.000	4.728	0.000	4.001	0.000	PA14_23070	3970.418	3.676	0.000
PA14_23080	1731.111	3.955	0.000	4.472	0.000	3.918	0.000	PA14_23080	1964.591	3.755	0.000
PA14_23090	357.073	3.713	0.000	3.759	0.000	2.157	0.000	PA14_23090	1063.532	3.325	0.000
PA14_23100	90.233	-1.177	0.005	-1.444	0.000	0.858	0.073	PA14_23100	286.336	-0.923	0.000
PA14_23110	741.652	0.560	0.047	1.406	0.000	0.629	0.058	PA14_23110	907.837	-0.382	0.279
PA14_23120	80.324	0.408	0.385	0.393	0.322	0.370	0.505	PA14_23120	227.151	-0.191	0.671
PA14_23130	128.963	-1.043	0.021	-0.724	0.082	0.280	0.647	PA14_23130	439.915	-0.242	0.366
PA14_23160	45.314	0.471	0.485	1.698	0.000	-0.281	0.743	PA14_23160	28.057	1.044	0.079
PA14_23170	209.335	1.621	0.000	3.667	0.000	1.680	0.000	PA14_23170	85.880	0.695	0.059
PA14_23190	156.613	0.605	0.136	2.755	0.000	1.196	0.005	PA14_23190	134.339	-0.088	0.790
PA14_23200	539.839	-0.347	0.270	-0.583	0.026	-0.146	0.721	PA14_23200	776.398	0.920	0.000
PA14_23210	474.261	-0.518	0.097	-1.019	0.000	0.072	0.874	PA14_23210	868.943	-0.251	0.297
PA14_23220	415.672	-0.283	0.508	-1.331	0.000	-0.607	0.170	PA14_23220	960.631	-0.551	0.013
PA14_23240	1520.440	0.671	0.093	0.713	0.049	1.263	0.004	PA14_23240	2187.081	0.323	0.159
PA14_23250	552.334	0.464	0.126	0.935	0.000	0.392	0.288	PA14_23250	769.304	1.283	0.000
PA14_23260	7833.804	0.400	0.109	0.567	0.010	0.890	0.001	PA14_23260	10283.382	0.926	0.000
PA14_23270	2664.267	0.079	0.850	-0.011	0.974	0.669	0.060	PA14_23270	4028.147	0.325	0.139
PA14_23280	1720.503	-0.257	0.490	-0.458	0.125	-0.144	0.749	PA14_23280	3347.776	0.298	0.182
PA14_23290	1043.577	0.322	0.284	1.034	0.000	0.694	0.028	PA14_23290	1421.204	1.341	0.000
PA14_23310	2027.307	0.238	0.438	0.655	0.006	0.618	0.046	PA14_23310	2156.128	0.788	0.000

PA14_23320	504.593	-0.123	0.762	-0.055	0.874	-0.386	0.331	PA14_23320	685.483	0.662	0.005
PA14_23330	47892.295	0.926	0.002	1.047	0.000	1.519	0.000	PA14_23330	26458.287	2.455	0.000
PA14_23340	4285.643	-0.142	0.818	-0.245	0.601	-1.771	0.000	PA14_23340	18274.800	-0.172	0.463
PA14_23350	4364.601	-0.005	0.989	0.413	0.162	0.113	0.802	PA14_23350	11789.525	-0.619	0.084
PA14_23360	13113.025	0.256	0.438	0.227	0.426	-0.417	0.238	PA14_23360	36165.870	0.906	0.001
PA14_23370	9577.876	0.011	0.980	-0.058	0.867	-0.956	0.006	PA14_23370	20236.814	0.886	0.001
PA14_23380	11014.393	0.231	0.663	-0.041	0.932	-2.168	0.000	PA14_23380	12358.045	-0.132	0.768
PA14_23390	6663.972	0.549	0.560	0.376	0.647	-0.815	0.394	PA14_23390	10034.246	-0.112	0.833
PA14_23400	5247.101	-0.520	0.332	-0.712	0.116	-1.558	0.004	PA14_23400	12114.418	0.511	0.285
PA14_23410	8821.324	0.083	0.906	-0.049	0.929	-0.958	0.089	PA14_23410	11465.702	-0.004	0.976
PA14_23420	34244.612	-0.565	0.081	-1.256	0.000	-1.064	0.003	PA14_23420	56894.633	-0.076	0.794
PA14_23430	16118.954	-0.258	0.535	-0.492	0.137	-0.851	0.035	PA14_23430	29665.458	0.660	0.013
PA14_23440	10712.297	-0.633	0.077	-1.101	0.000	-1.862	0.000	PA14_23440	27580.743	0.473	0.100
PA14_23450	4771.003	-0.517	0.219	-0.948	0.008	-1.357	0.002	PA14_23450	15590.319	0.367	0.339
PA14_23460	5771.525	-0.094	0.936	-0.479	0.560	-0.702	0.480	PA14_23460	11764.101	0.123	0.767
PA14_23470	6108.744	-0.118	0.759	0.125	0.688	-0.389	0.291	PA14_23470	10229.399	0.695	0.001
PA14_23480	98.198	-0.344	0.524	0.080	0.871	-0.560	0.341	PA14_23480	476.206	-0.822	0.001
PA14_23490	10.590	-0.945	0.294	-0.724	0.352	-1.494	0.135	#N/A	#N/A	#N/A	#N/A
PA14_23500	849.318	-0.662	0.022	-0.887	0.001	-0.598	0.088	PA14_23500	1730.768	0.522	0.017
PA14_23510	1471.093	0.382	0.169	0.163	0.550	-0.052	0.897	PA14_23510	2930.647	0.410	0.061
PA14_23520	50.399	0.700	0.179	0.590	0.207	-0.832	0.225	PA14_23520	124.338	0.268	0.467
PA14_23530	37.254	1.821	0.000	1.664	0.001	0.311	0.715	PA14_23530	125.795	-0.368	0.318
PA14_23540	161.121	0.620	0.071	1.121	0.000	0.705	0.081	PA14_23540	519.948	0.278	0.295
PA14_23560	1143.151	0.126	0.748	0.014	0.967	-0.595	0.099	PA14_23560	1961.351	0.815	0.000
PA14_23570	28.008	-0.021	0.983	1.509	0.012	0.309	0.748	#N/A	#N/A	#N/A	#N/A
PA14_23580	15.026	2.135	0.042	4.636	0.000	2.245	0.042	#N/A	#N/A	#N/A	#N/A
PA14_23590	138.987	0.528	0.173	1.897	0.000	0.420	0.384	PA14_23590	156.144	-0.545	0.096
PA14_23610	18.614	-0.654	0.453	0.014	0.986	0.052	0.963	PA14_23610	63.325	-1.584	0.000
PA14_23620	280.658	0.528	0.135	0.555	0.076	-0.380	0.402	PA14_23620	721.868	0.085	0.730
PA14_23630	85.428	0.134	0.807	0.019	0.967	-0.639	0.234	PA14_23630	342.401	0.270	0.346
PA14_23640	163.267	0.424	0.295	1.589	0.000	-0.492	0.327	PA14_23640	260.702	0.644	0.020
PA14_23650	58.682	-0.616	0.216	-1.736	0.000	-1.433	0.014	PA14_23650	367.003	-0.317	0.246
PA14_23670	316.795	-0.037	0.949	-0.618	0.090	-0.821	0.082	PA14_23670	936.917	0.049	0.871
PA14_23680	615.215	-1.597	0.000	-1.594	0.000	-4.157	0.000	PA14_23680	2876.300	-1.204	0.000
PA14_23690	38.807	0.902	0.118	0.423	0.449	0.119	0.893	PA14_23690	77.469	0.750	0.059
PA14_23700	319.262	0.378	0.254	0.554	0.046	-1.117	0.003	PA14_23700	827.750	0.570	0.015
PA14_23720	607.235	-0.859	0.001	-0.866	0.000	-0.792	0.015	PA14_23720	2517.661	-0.948	0.000
PA14_23730	149.143	0.170	0.742	0.010	0.981	0.470	0.348	PA14_23730	188.099	0.302	0.323
PA14_23750	4818.851	0.200	0.495	0.095	0.719	-0.062	0.869	PA14_23750	2162.323	0.208	0.385
PA14_23760	2934.451	1.231	0.003	1.627	0.000	0.134	0.839	PA14_23760	1095.630	-0.487	0.032
PA14_23770	2832.808	0.554	0.072	0.808	0.003	-0.080	0.863	PA14_23770	738.750	0.112	0.698
PA14_23790	2200.129	0.250	0.404	-0.082	0.772	0.143	0.700	PA14_23790	1021.863	0.652	0.003
PA14_23800	2125.493	-0.183	0.567	-0.214	0.416	-0.997	0.001	PA14_23800	2816.004	1.276	0.000
PA14_23810	2626.404	-0.706	0.026	-1.327	0.000	-2.458	0.000	PA14_23810	6871.776	-1.044	0.000
PA14_23830	11123.634	-0.388	0.126	-0.687	0.002	-1.877	0.000	PA14_23830	36410.128	-0.203	0.351
PA14_23840	302.207	0.117	0.808	1.156	0.000	-0.534	0.242	PA14_23840	1054.616	1.742	0.000
PA14_23850	1078.886	0.616	0.027	1.447	0.000	2.356	0.000	PA14_23850	1685.150	0.666	0.002
PA14_23860	1920.296	0.202	0.545	0.686	0.006	0.172	0.657	PA14_23860	3552.572	1.255	0.000
PA14_23880	1717.282	0.196	0.544	0.701	0.004	0.464	0.155	PA14_23880	2581.094	0.933	0.000
PA14_23890	754.814	0.217	0.501	0.324	0.215	-0.327	0.356	PA14_23890	1351.668	0.574	0.009
PA14_23900	322.067	1.045	0.010	2.174	0.000	1.845	0.000	PA14_23900	912.882	0.774	0.001
PA14_23920	4654.935	0.518	0.101	1.215	0.000	1.088	0.002	PA14_23920	4656.612	0.728	0.001
PA14_23930	605.745	0.002	0.997	0.510	0.072	0.134	0.759	PA14_23930	1499.307	0.529	0.017
PA14_23950	259.095	-0.389	0.248	0.302	0.302	-0.121	0.788	PA14_23950	576.416	0.472	0.071
PA14_23970	3369.737	-0.990	0.000	-1.727	0.000	-1.372	0.000	PA14_23970	5613.395	0.813	0.000
PA14_23980	3502.441	-0.459	0.077	-0.399	0.095	0.961	0.001	PA14_23980	6688.840	-0.470	0.027
PA14_23990	1073.375	-1.323	0.000	-2.330	0.000	-2.069	0.000	PA14_23990	2662.397	-0.681	0.002
PA14_24010	238.581	-1.171	0.001	-1.888	0.000	-2.219	0.000	PA14_24010	1259.518	-0.328	0.166
PA14_24020	891.083	-1.847	0.000	-2.713	0.000	-3.143	0.000	PA14_24020	2140.959	-0.097	0.692

PA14_24040	338.743	-1.112	0.000	-1.837	0.000	-1.968	0.000	PA14_24040	885.243	-0.375	0.105
PA14_24050	141.430	-0.894	0.009	-1.639	0.000	-2.712	0.000	PA14_24050	359.980	-0.156	0.623
PA14_24060	532.674	-0.578	0.039	-1.340	0.000	-1.572	0.000	PA14_24060	1068.645	-0.210	0.387
PA14_24070	1486.343	-0.243	0.581	-0.292	0.422	-0.667	0.127	PA14_24070	2004.570	0.250	0.289
PA14_24080	435.329	-1.059	0.000	-1.645	0.000	-1.844	0.000	PA14_24080	1118.848	-0.599	0.007
PA14_24100	134.411	-0.996	0.004	-1.356	0.000	-2.561	0.000	PA14_24100	452.690	-1.271	0.000
PA14_24110	2.502	-0.702	0.643	-0.835	0.499	-1.203	0.345	#N/A	#N/A	#N/A	#N/A
PA14_24120	50.130	0.208	0.777	1.265	0.008	0.885	0.176	#N/A	#N/A	#N/A	#N/A
PA14_24130	33.321	0.091	0.936	0.833	0.243	-0.204	0.852	#N/A	#N/A	#N/A	#N/A
PA14_24140	443.954	0.634	0.034	0.960	0.000	0.099	0.827	PA14_24140	672.007	1.059	0.000
PA14_24150	240.018	1.211	0.000	1.565	0.000	0.261	0.578	PA14_24150	734.110	0.879	0.000
PA14_24170	462.960	-0.406	0.261	-0.997	0.001	0.798	0.035	PA14_24170	1519.540	-0.366	0.202
PA14_24180	282.101	-1.109	0.000	-1.756	0.000	-0.355	0.395	PA14_24180	991.728	-1.256	0.000
PA14_24190	63.460	0.328	0.565	1.131	0.005	0.280	0.677	PA14_24190	131.710	0.762	0.021
PA14_24210	73.000	-0.706	0.123	-1.580	0.000	0.456	0.401	PA14_24210	779.844	-3.200	0.000
PA14_24220	676.458	0.296	0.370	0.174	0.561	-1.074	0.002	PA14_24220	1755.904	-0.219	0.361
PA14_24230	144.528	0.194	0.686	-0.031	0.943	-0.683	0.159	PA14_24230	394.264	0.436	0.096
PA14_24240	723.442	0.343	0.501	0.755	0.059	-0.667	0.210	PA14_24240	1169.320	-0.552	0.015
PA14_24245	2400.141	0.341	0.289	0.614	0.022	-0.028	0.950	PA14_24245	4190.995	-0.489	0.331
PA14_24260	1979.707	0.444	0.167	0.598	0.033	1.065	0.002	PA14_24260	3638.267	-0.290	0.201
PA14_24270	1909.484	-0.363	0.281	-0.833	0.003	-0.418	0.275	PA14_24270	4369.400	0.184	0.397
PA14_24290	1199.292	0.954	0.001	0.288	0.333	-0.413	0.282	PA14_24290	1018.789	1.043	0.000
PA14_24300	506.936	1.074	0.000	0.489	0.060	-0.487	0.173	PA14_24300	480.814	1.330	0.000
PA14_24310	277.258	-0.305	0.449	-0.652	0.040	0.903	0.020	PA14_24310	554.898	0.377	0.128
PA14_24330	877.306	0.293	0.297	-0.382	0.112	-0.120	0.745	PA14_24330	1003.068	0.288	0.236
PA14_24340	156.809	0.403	0.281	0.159	0.651	-0.131	0.797	PA14_24340	254.557	0.805	0.015
PA14_24350	90.419	1.064	0.005	1.208	0.000	0.781	0.107	PA14_24350	184.170	1.626	0.000
PA14_24360	126.939	-0.253	0.563	0.096	0.803	0.750	0.069	PA14_24360	277.744	-0.131	0.658
PA14_24370	149.194	-0.263	0.572	-1.067	0.002	-1.428	0.002	PA14_24370	751.497	-1.150	0.000
PA14_24380	181.845	-0.001	0.999	-0.243	0.473	-0.878	0.035	PA14_24380	357.214	0.359	0.196
PA14_24390	205.800	-0.262	0.480	-0.749	0.008	-0.947	0.013	PA14_24390	273.255	0.736	0.024
PA14_24400	60.651	-0.310	0.546	-0.797	0.043	-1.388	0.012	PA14_24400	127.769	1.771	0.000
PA14_24410	10.533	-0.669	0.483	-0.406	0.616	-0.510	0.637	PA14_24410	37.410	1.995	0.044
PA14_24420	158.519	0.165	0.729	0.083	0.836	-0.161	0.765	PA14_24420	420.290	0.353	0.191
PA14_24430	1117.999	0.437	0.143	0.347	0.204	-0.105	0.802	PA14_24430	2609.236	-0.254	0.291
PA14_24440	300.678	-0.624	0.048	-0.054	0.875	0.887	0.011	PA14_24440	829.549	0.371	0.127
PA14_24445	10667.970	-0.515	0.040	-2.682	0.000	-3.762	0.000	PA14_24445	14701.537	-1.563	0.000
PA14_24480	166.663	-0.371	0.379	0.070	0.862	0.790	0.062	PA14_24480	156.113	0.880	0.005
PA14_24490	154.703	-0.555	0.114	-0.615	0.046	0.020	0.970	PA14_24490	179.624	0.622	0.055
PA14_24500	1.589	-0.926	0.538	-1.117	0.370	-0.692	0.620	PA14_24500	5.598	1.593	0.286
PA14_24510	13.452	-0.375	0.687	-1.172	0.094	-0.473	0.638	PA14_24510	26.118	1.025	0.102
PA14_24530	39.185	-0.901	0.112	-0.595	0.244	-0.321	0.667	PA14_24530	49.535	0.417	0.385
PA14_24550	77.792	-0.645	0.120	-0.981	0.006	-0.347	0.518	PA14_24550	115.494	0.415	0.236
PA14_24560	47.303	-0.899	0.070	-0.271	0.569	-0.089	0.900	PA14_24560	96.336	0.171	0.659
PA14_24570	65.827	0.078	0.899	-0.339	0.419	-0.593	0.299	PA14_24570	323.803	0.637	0.014
PA14_24580	521.728	0.215	0.601	-0.036	0.927	-1.734	0.000	PA14_24580	1807.829	-0.104	0.891
PA14_24590	362.980	0.110	0.775	-0.031	0.925	-1.008	0.004	PA14_24590	1197.537	-0.090	0.770
PA14_24600	421.586	-0.933	0.001	-1.867	0.000	-2.239	0.000	PA14_24600	1055.878	0.666	0.005
PA14_24610	675.498	-0.004	0.993	-0.173	0.649	-0.670	0.128	PA14_24610	1253.773	-0.216	0.365
PA14_24620	413.157	0.079	0.866	0.042	0.911	-0.143	0.764	PA14_24620	1088.846	-0.171	0.495
PA14_24630	24.989	0.201	0.823	0.252	0.709	1.884	0.005	PA14_24630	74.359	-0.208	0.741
PA14_24640	1334.911	0.924	0.000	1.524	0.000	1.527	0.000	PA14_24640	1979.406	1.612	0.000
PA14_24650	5927.658	-3.708	0.000	-5.058	0.000	1.566	0.000	PA14_24650	26891.430	-4.296	0.000
PA14_24665	226.066	0.431	0.265	1.124	0.000	1.847	0.000	PA14_24665	298.589	0.458	0.097
PA14_24675	651.857	0.334	0.330	1.107	0.000	1.433	0.000	PA14_24675	586.786	0.840	0.000
PA14_24690	1163.634	-0.071	0.875	-0.604	0.039	0.209	0.630	PA14_24690	2693.132	-0.252	0.296
PA14_24700	145.546	-0.375	0.548	-0.328	0.535	-0.727	0.261	PA14_24700	425.342	0.252	0.361
PA14_24710	126.725	0.731	0.053	0.285	0.449	-0.175	0.758	PA14_24710	390.423	-0.931	0.000
PA14_24720	136.855	0.540	0.159	0.657	0.046	1.019	0.013	PA14_24720	385.727	1.204	0.000

PA14_24730	1056.470	-0.653	0.011	-1.097	0.000	0.139	0.708
PA14_24740	147.983	-2.148	0.000	-3.006	0.000	0.403	0.450
PA14_24760	277.688	-2.134	0.000	-3.026	0.000	-0.045	0.923
PA14_24770	5310.667	-2.117	0.000	-3.035	0.000	-0.198	0.622
PA14_24780	79.860	0.296	0.566	1.072	0.003	0.583	0.272
PA14_24790	1275.522	0.893	0.005	-0.920	0.002	-0.855	0.029
PA14_24810	18.959	-0.120	0.896	-0.485	0.440	-1.944	0.025
PA14_24820	5.006	-0.178	0.910	0.417	0.707	0.682	0.598
PA14_24830	111.036	0.652	0.080	0.513	0.132	-0.705	0.153
PA14_24840	159.687	-0.099	0.857	0.334	0.378	0.479	0.328
PA14_24850	46.158	0.607	0.418	1.051	0.076	0.811	0.307
PA14_24860	177.218	-0.835	0.011	0.029	0.937	0.825	0.026
PA14_24870	3.729	-0.890	0.516	0.219	0.858	0.126	0.933
PA14_24880	2139.052	-1.253	0.000	-1.079	0.000	-0.633	0.091
PA14_24890	353.400	0.723	0.034	0.415	0.205	-0.708	0.100
PA14_24900	338.589	-0.167	0.787	-0.682	0.119	-1.629	0.002
PA14_24910	869.839	0.662	0.039	0.650	0.027	-1.073	0.004
PA14_24920	304.500	0.713	0.015	0.514	0.064	0.049	0.916
PA14_24940	215.321	-0.106	0.803	0.153	0.631	-0.081	0.865
PA14_24950	154.005	-0.087	0.867	0.181	0.629	-0.379	0.444
PA14_24960	254.637	0.294	0.458	0.385	0.234	-0.112	0.826
PA14_24970	152.423	-1.517	0.000	-1.720	0.000	-0.902	0.078
PA14_24980	359.374	-1.404	0.000	-1.566	0.000	-2.369	0.000
PA14_24990	256.778	-0.669	0.044	-1.304	0.000	-0.338	0.434
PA14_25000	2262.318	0.266	0.439	-0.119	0.708	0.789	0.021
PA14_25020	784.296	0.602	0.033	0.948	0.000	0.729	0.026
PA14_25030	591.906	0.548	0.093	0.855	0.003	-0.928	0.015
PA14_25040	225.644	-1.389	0.000	-1.555	0.000	-0.517	0.175
PA14_25050	64.029	1.201	0.009	1.923	0.000	1.341	0.013
PA14_25060	217.781	0.393	0.344	0.571	0.093	0.780	0.071
PA14_25080	6415.882	0.395	0.229	-0.315	0.290	0.199	0.634
PA14_25090	1748.869	0.012	0.975	-0.739	0.001	-0.141	0.697
PA14_25100	934.569	0.310	0.294	0.601	0.013	1.238	0.000
PA14_25110	11371.226	0.269	0.323	0.439	0.053	-0.321	0.297
PA14_25130	1353.860	0.101	0.773	0.273	0.289	0.506	0.111
PA14_25140	1159.498	1.319	0.000	2.257	0.000	3.264	0.000
PA14_25150	116.262	-0.031	0.964	0.242	0.582	-0.818	0.138
PA14_25160	467.994	0.812	0.015	1.052	0.000	-0.837	0.047
PA14_25180	1901.018	-0.120	0.767	-1.424	0.000	-0.237	0.569
PA14_25195	1208.596	0.442	0.154	0.500	0.068	0.918	0.006
PA14_25210	1414.443	0.108	0.843	0.521	0.162	0.253	0.637
PA14_25220	596.235	-0.774	0.003	-1.068	0.000	-0.860	0.007
PA14_25230	2410.216	0.055	0.884	0.059	0.840	-0.052	0.896
PA14_25250	8986.301	-0.124	0.731	0.599	0.019	0.620	0.059
PA14_25270	873.684	-0.092	0.849	0.729	0.021	1.083	0.005
PA14_25280	1200.403	0.494	0.091	0.963	0.000	0.252	0.509
PA14_25305	1135.790	0.296	0.321	0.479	0.052	0.272	0.436
PA14_25320	1035.405	0.268	0.463	0.308	0.314	0.006	0.990
PA14_25330	476.946	-0.031	0.942	-0.214	0.462	-0.641	0.067
PA14_25340	1306.562	0.296	0.495	0.420	0.239	0.302	0.538
PA14_25350	1200.378	-0.160	0.654	-0.169	0.565	-0.685	0.042
PA14_25360	648.697	0.142	0.679	0.364	0.153	-0.215	0.561
PA14_25370	605.073	-0.314	0.451	-0.282	0.430	-1.091	0.008
PA14_25390	4655.077	0.444	0.125	1.147	0.000	-0.132	0.741
PA14_25400	1148.374	-0.352	0.473	-0.196	0.654	-0.074	0.906
PA14_25410	116.481	-0.530	0.173	-0.352	0.313	0.833	0.042
PA14_25420	339.835	-0.519	0.121	-0.066	0.852	-0.248	0.570
PA14_25430	402.699	-0.619	0.062	-0.315	0.320	0.075	0.877
PA14_25440	747.343	0.061	0.935	1.338	0.003	1.169	0.040

PA14_24730	2981.937	-0.179	0.468
PA14_24740	689.394	-1.191	0.000
PA14_24760	968.438	-1.553	0.000
PA14_24770	17127.962	-2.303	0.000
PA14_24780	153.074	1.249	0.000
PA14_24790	1370.573	1.560	0.000
PA14_24810	26.387	0.384	0.572
PA14_24820	18.557	0.641	0.406
PA14_24830	191.580	-0.516	0.076
PA14_24840	499.168	0.214	0.439
PA14_24850	158.795	-0.522	0.367
PA14_24860	2409.663	-4.193	0.000
#N/A	#N/A	#N/A	#N/A
PA14_24880	8684.007	-0.945	0.000
PA14_24890	780.818	1.156	0.000
PA14_24900	1382.647	0.574	0.009
PA14_24910	1150.321	1.260	0.000
PA14_24920	588.644	2.223	0.000
PA14_24940	505.118	-0.642	0.010
PA14_24950	275.577	-0.070	0.880
PA14_24960	508.413	-0.473	0.058
PA14_24970	1571.075	-2.396	0.000
PA14_24980	1515.796	-0.877	0.000
PA14_24990	553.021	0.241	0.353
PA14_25000	5323.592	-0.271	0.342
PA14_25020	908.887	1.392	0.000
PA14_25030	1478.873	3.612	0.000
PA14_25040	1371.444	-2.698	0.000
PA14_25050	157.279	0.775	0.293
PA14_25060	460.940	1.105	0.000
PA14_25080	26904.099	0.661	0.044
PA14_25090	7682.955	0.510	0.122
PA14_25100	2840.857	-0.045	0.947
PA14_25110	13956.301	0.759	0.000
PA14_25130	2038.295	-0.723	0.001
PA14_25140	1769.835	-0.706	0.018
PA14_25150	356.156	-0.236	0.415
PA14_25160	1358.876	-0.706	0.001
PA14_25180	14428.503	-2.231	0.000
PA14_25195	2825.562	0.518	0.080
PA14_25210	2269.270	0.350	0.135
PA14_25220	1087.505	-0.466	0.042
PA14_25230	3646.482	0.092	0.714
PA14_25250	9714.271	1.445	0.000
PA14_25270	1091.769	1.708	0.000
PA14_25280	1918.517	0.874	0.000
PA14_25305	1136.805	0.816	0.000
PA14_25320	983.678	0.164	0.513
PA14_25330	625.972	0.230	0.383
PA14_25340	1258.590	0.270	0.275
PA14_25350	2050.887	0.313	0.180
PA14_25360	1340.374	0.175	0.487
PA14_25370	1389.422	-0.085	0.759
PA14_25390	5350.510	0.567	0.008
PA14_25400	2331.316	-0.037	0.916
PA14_25410	780.150	-0.188	0.568
PA14_25420	1438.180	-0.963	0.000
PA14_25430	1690.593	0.521	0.020
PA14_25440	1022.872	1.351	0.000

PA14_25450	759.850	-0.090	0.809	0.580	0.019	-0.154	0.691	PA14_25450	1158.610	1.214	0.000
PA14_25470	223.599	-0.811	0.012	0.213	0.503	1.473	0.000	PA14_25470	1063.390	-0.808	0.000
PA14_25480	163.694	0.390	0.340	0.842	0.009	0.508	0.266	PA14_25480	481.730	-0.124	0.648
PA14_25490	638.555	0.000	0.999	0.659	0.012	-0.312	0.416	PA14_25490	1951.651	0.927	0.000
PA14_25500	115.541	0.045	0.936	0.426	0.227	0.035	0.951	PA14_25500	278.299	1.021	0.000
PA14_25510	272.029	0.346	0.321	1.121	0.000	0.896	0.011	PA14_25510	911.204	0.729	0.001
PA14_25520	479.425	0.589	0.043	0.998	0.000	0.161	0.700	PA14_25520	922.304	0.719	0.001
PA14_25530	449.643	0.441	0.212	0.778	0.008	0.182	0.697	PA14_25530	741.010	0.850	0.000
PA14_25540	298.654	0.873	0.012	1.152	0.000	0.697	0.104	PA14_25540	452.141	0.626	0.012
PA14_25550	702.318	0.592	0.037	1.347	0.000	0.445	0.208	PA14_25550	771.874	0.692	0.003
PA14_25560	29751.476	1.067	0.000	1.055	0.000	2.484	0.000	PA14_25560	17290.116	1.788	0.000
PA14_25580	2047.653	0.852	0.002	0.864	0.001	0.934	0.005	PA14_25580	5342.354	0.430	0.049
PA14_25590	2028.127	0.612	0.027	0.978	0.000	0.544	0.105	PA14_25590	5833.139	1.557	0.000
PA14_25600	2100.980	0.037	0.937	0.129	0.707	0.105	0.825	PA14_25600	5618.596	1.976	0.000
PA14_25610	186.749	0.857	0.035	1.405	0.000	1.223	0.007	PA14_25610	251.457	1.331	0.000
PA14_25620	3590.457	0.660	0.070	1.708	0.000	1.112	0.006	PA14_25620	9043.833	2.414	0.000
PA14_25630	239.545	0.295	0.621	1.278	0.003	0.798	0.167	PA14_25630	1462.295	1.697	0.000
PA14_25640	1516.818	0.738	0.007	2.069	0.000	1.774	0.000	PA14_25640	1545.360	1.998	0.000
PA14_25650	3490.617	0.074	0.835	0.451	0.062	-0.142	0.699	PA14_25650	2235.235	1.227	0.000
PA14_25660	9050.270	-0.638	0.042	-0.304	0.324	0.138	0.757	PA14_25660	14699.048	-0.044	0.843
PA14_25670	14487.577	-0.047	0.910	0.215	0.446	-0.507	0.134	PA14_25670	37891.604	-0.110	0.624
PA14_25690	1793.560	-0.066	0.881	0.055	0.872	-0.585	0.112	PA14_25690	3693.563	1.060	0.000
PA14_25710	605.027	0.293	0.357	0.881	0.000	0.417	0.234	PA14_25710	1446.129	0.309	0.176
PA14_25730	1611.070	0.213	0.538	0.909	0.000	0.435	0.223	PA14_25730	2766.514	0.391	0.080
PA14_25740	153.715	-0.117	0.807	0.267	0.437	-0.016	0.976	PA14_25740	406.920	0.615	0.017
PA14_25760	564.361	0.429	0.180	1.240	0.000	0.583	0.108	PA14_25760	922.019	0.690	0.003
PA14_25770	2131.563	-0.224	0.530	0.222	0.462	0.426	0.250	PA14_25770	7387.898	-0.549	0.008
PA14_25780	299.715	0.147	0.754	0.221	0.546	-0.192	0.708	PA14_25780	947.666	1.049	0.000
PA14_25790	201.575	-0.327	0.411	-0.293	0.384	0.642	0.112	PA14_25790	920.369	-0.451	0.053
PA14_25800	641.113	0.429	0.172	1.222	0.000	1.752	0.000	PA14_25800	976.401	0.235	0.330
PA14_25810	368.819	-0.586	0.052	-0.448	0.106	-0.593	0.102	PA14_25810	1004.462	-0.299	0.217
PA14_25820	267.654	-0.036	0.936	0.218	0.467	0.128	0.770	PA14_25820	597.649	0.432	0.127
PA14_25830	49.484	-0.715	0.176	-1.292	0.004	-1.245	0.045	PA14_25830	173.711	0.033	0.944
PA14_25840	5891.762	0.112	0.741	-0.789	0.001	-0.833	0.005	PA14_25840	24901.639	0.341	0.106
PA14_25860	8528.279	-0.416	0.084	-1.282	0.000	-1.473	0.000	PA14_25860	21524.137	-0.062	0.835
PA14_25880	3301.938	0.070	0.851	-0.676	0.005	-0.937	0.002	PA14_25880	10015.382	0.091	0.669
PA14_25900	2594.084	-0.150	0.693	-0.034	0.923	-0.645	0.067	PA14_25900	3082.954	0.988	0.000
PA14_25910	249.758	-0.407	0.235	-0.398	0.182	-0.351	0.395	PA14_25910	614.253	-0.601	0.200
PA14_25920	121.317	0.200	0.682	-2.887	0.000	-1.997	0.000	PA14_25920	196.606	2.383	0.000
PA14_25930	57.870	0.831	0.096	-1.768	0.000	-1.150	0.088	PA14_25930	112.484	3.152	0.000
PA14_25940	1254.026	0.033	0.949	-1.292	0.000	-0.752	0.070	PA14_25940	2993.199	0.547	0.011
PA14_25960	206.197	0.031	0.964	-1.321	0.001	-1.256	0.014	PA14_25960	243.070	0.771	0.005
PA14_25970	489.685	0.174	0.616	-2.058	0.000	-2.011	0.000	PA14_25970	444.985	0.589	0.019
PA14_25980	73.187	0.287	0.621	0.604	0.165	0.226	0.738	PA14_25980	127.277	1.183	0.000
PA14_25990	175.092	0.834	0.011	1.574	0.000	0.866	0.028	PA14_25990	267.468	1.580	0.000
PA14_26000	34.347	0.260	0.721	1.233	0.010	-0.149	0.868	PA14_26000	53.098	0.704	0.136
PA14_26010	84.770	0.339	0.468	0.638	0.076	-0.617	0.261	PA14_26010	197.086	-0.143	0.632
PA14_26020	2483.435	-5.024	0.000	-7.163	0.000	-2.215	0.000	PA14_26020	5624.422	-5.031	0.000
PA14_26050	130.947	4.170	0.000	1.890	0.000	0.840	0.162	PA14_26050	40.570	0.238	0.661
PA14_26060	837.320	-1.437	0.000	-1.644	0.000	2.294	0.000	PA14_26060	5042.120	-4.036	0.000
PA14_26070	23.343	-0.812	0.254	-0.572	0.355	-0.550	0.522	PA14_26070	127.311	-0.550	0.083
PA14_26080	22.708	0.686	0.358	1.006	0.091	1.026	0.190	PA14_26080	104.851	0.384	0.311
PA14_26090	32.112	0.443	0.496	0.567	0.278	-0.518	0.516	PA14_26090	80.155	1.931	0.000
PA14_26110	22.663	0.897	0.212	1.300	0.028	1.619	0.026	PA14_26110	56.140	2.113	0.000
PA14_26130	84.158	2.733	0.000	2.911	0.000	0.014	0.987	PA14_26130	141.638	2.673	0.000
PA14_26140	79.677	-0.106	0.864	0.181	0.689	-1.390	0.015	PA14_26140	466.408	1.246	0.000
PA14_26150	79.125	0.429	0.361	1.242	0.000	-0.676	0.249	PA14_26150	376.410	-1.104	0.002
PA14_26160	16.765	0.526	0.566	1.030	0.135	0.161	0.887	PA14_26160	44.481	3.074	0.000
PA14_26165	99.619	1.020	0.015	2.202	0.000	-0.761	0.223	PA14_26165	118.078	1.264	0.000

PA14_26190	53.435	-1.337	0.004	-1.622	0.000	-1.491	0.010	PA14_26190	294.536	-1.456	0.000
PA14_26200	4.084	1.208	0.358	0.770	0.519	0.696	0.618	PA14_26200	7.989	0.692	0.567
PA14_26210	19.302	0.511	0.557	0.702	0.307	0.645	0.486	PA14_26210	52.072	-0.464	0.402
PA14_26220	20.278	-0.397	0.650	-0.922	0.168	0.726	0.387	PA14_26220	26.547	0.472	0.467
PA14_26230	5.471	-0.154	0.922	-2.083	0.046	0.062	0.966	PA14_26230	9.360	0.648	0.566
PA14_26240	8.232	-0.379	0.745	-1.506	0.082	0.286	0.812	PA14_26240	11.905	0.321	0.776
PA14_26260	8.837	0.600	0.584	-0.332	0.737	0.796	0.474	PA14_26260	14.159	0.294	0.794
PA14_26270	25.024	0.290	0.721	0.982	0.078	-0.267	0.779	PA14_26270	75.151	2.185	0.000
PA14_26280	804.670	-0.847	0.013	-1.004	0.001	1.472	0.000	PA14_26280	2452.484	-1.842	0.000
PA14_26300	3.802	0.431	0.786	1.094	0.342	1.238	0.338	PA14_26300	9.520	-0.672	0.592
PA14_26310	150.053	-0.375	0.459	-0.251	0.569	-0.102	0.874	PA14_26310	351.997	-0.937	0.000
PA14_26330	153.516	-0.776	0.029	-1.169	0.000	-0.147	0.773	PA14_26330	773.297	-2.297	0.000
PA14_26340	44.286	-1.105	0.036	-2.968	0.000	-3.672	0.000	PA14_26340	215.331	-4.442	0.000
PA14_26350	361.794	-1.649	0.000	-2.073	0.000	-1.243	0.001	PA14_26350	2304.464	-1.824	0.000
PA14_26360	23.443	0.496	0.548	0.552	0.406	-0.585	0.570	PA14_26360	26.624	0.481	0.466
PA14_26390	40.056	1.408	0.009	0.663	0.222	0.107	0.909	PA14_26390	29.313	1.188	0.043
PA14_26400	111.571	1.335	0.011	0.568	0.286	-0.384	0.627	PA14_26400	43.297	0.554	0.279
PA14_26420	104.771	0.874	0.028	-0.002	0.997	-0.443	0.461	PA14_26420	63.546	0.087	0.866
PA14_26450	16.602	0.761	0.428	1.408	0.053	3.613	0.000	PA14_26450	15.365	0.605	0.515
PA14_26460	21.571	0.820	0.269	1.031	0.093	0.191	0.855	PA14_26460	18.688	-0.112	0.915
PA14_26470	71.547	0.396	0.450	0.291	0.522	-1.067	0.078	PA14_26470	101.238	1.006	0.004
PA14_26480	101.787	0.952	0.019	0.831	0.027	0.006	0.992	PA14_26480	131.904	0.603	0.069
PA14_26485	130.306	1.042	0.004	0.085	0.842	0.200	0.721	PA14_26485	219.814	2.507	0.000
PA14_26500	135.726	1.206	0.000	-0.107	0.782	-1.444	0.003	PA14_26500	126.797	2.083	0.000
PA14_26510	100.633	0.063	0.935	-1.933	0.000	-1.901	0.003	PA14_26510	165.449	1.486	0.000
PA14_26530	123.678	-0.055	0.916	-1.452	0.000	-1.366	0.002	PA14_26530	140.085	1.558	0.000
PA14_26540	903.592	0.775	0.005	1.089	0.000	0.705	0.038	PA14_26540	2483.117	1.225	0.000
PA14_26550	737.574	0.010	0.982	0.495	0.074	0.265	0.505	PA14_26550	2690.780	1.108	0.000
PA14_26560	322.297	0.201	0.700	0.502	0.195	0.028	0.965	PA14_26560	939.750	0.763	0.001
PA14_26570	247.463	0.468	0.212	0.724	0.022	0.296	0.529	PA14_26570	524.698	-0.518	0.032
PA14_26580	12.238	-0.072	0.958	1.344	0.079	-0.289	0.819	PA14_26580	24.852	-0.669	0.309
PA14_26590	3112.185	0.291	0.306	0.694	0.003	0.296	0.371	PA14_26590	13508.787	0.328	0.141
PA14_26600	643.071	0.557	0.071	1.426	0.000	2.621	0.000	PA14_26600	1753.725	0.063	0.792
PA14_26610	249.604	-0.086	0.860	0.336	0.314	1.919	0.000	PA14_26610	661.793	0.338	0.172
PA14_26620	260.705	-0.366	0.308	-0.015	0.967	0.828	0.023	PA14_26620	700.514	-0.297	0.217
PA14_26640	255.001	-0.252	0.505	-0.494	0.094	1.498	0.000	PA14_26640	340.813	-0.672	0.020
PA14_26650	119.433	0.815	0.038	0.126	0.767	0.311	0.567	PA14_26650	129.901	-0.213	0.563
PA14_26670	230.115	0.085	0.853	-0.382	0.234	-0.284	0.532	PA14_26670	232.439	-0.476	0.104
PA14_26690	14.976	0.006	0.996	-0.842	0.240	-0.845	0.386	PA14_26690	23.552	-0.029	1.000
PA14_26700	117.756	0.115	0.822	-0.391	0.280	-1.018	0.031	PA14_26700	147.055	0.140	0.673
PA14_26720	173.106	0.151	0.748	-0.565	0.094	-0.344	0.480	PA14_26720	172.260	0.055	0.906
PA14_26730	97.430	0.524	0.230	0.341	0.390	0.012	0.985	PA14_26730	86.812	1.259	0.001
PA14_26750	145.159	0.537	0.176	0.364	0.319	0.529	0.261	PA14_26750	161.043	0.999	0.001
PA14_26760	502.224	0.166	0.772	0.813	0.042	-1.494	0.003	PA14_26760	1997.200	-0.473	0.030
PA14_26770	177.406	-0.052	0.920	0.735	0.017	1.045	0.007	PA14_26770	587.634	0.176	0.568
PA14_26780	200.852	-2.038	0.000	-2.145	0.000	-0.972	0.041	PA14_26780	1836.083	-2.254	0.000
PA14_26810	17.442	-0.153	0.880	0.871	0.164	0.236	0.815	PA14_26810	142.642	-1.437	0.000
PA14_26830	38.742	-0.195	0.809	0.628	0.252	0.317	0.697	PA14_26830	259.442	-1.631	0.000
PA14_26850	16.810	-0.127	0.906	-0.279	0.717	1.865	0.012	PA14_26850	32.167	0.974	0.090
PA14_26860	725.807	0.245	0.441	0.814	0.001	-0.688	0.038	PA14_26860	2584.226	1.024	0.000
PA14_26870	5.317	-0.810	0.508	0.122	0.913	-0.668	0.602	PA14_26870	21.638	1.165	0.277
PA14_26880	492.174	1.108	0.000	1.775	0.000	1.257	0.000	PA14_26880	1748.348	1.801	0.000
PA14_26890	88.996	0.316	0.549	0.971	0.012	0.437	0.445	PA14_26890	198.450	1.026	0.000
PA14_26910	142.714	-0.695	0.138	0.223	0.619	1.062	0.032	PA14_26910	335.828	-0.952	0.000
PA14_26920	18.475	-0.325	0.714	-0.739	0.268	0.027	0.978	PA14_26920	50.113	-0.473	0.325
PA14_26930	263.767	-0.257	0.591	-0.199	0.623	-0.382	0.459	PA14_26930	349.068	-0.700	0.006
PA14_26940	69.085	-0.702	0.123	-0.559	0.165	0.662	0.192	PA14_26940	208.754	-1.520	0.000
PA14_26960	253.390	0.193	0.624	-0.017	0.963	-1.250	0.001	PA14_26960	498.146	-0.262	0.306
PA14_26970	124.726	-0.697	0.085	-1.081	0.002	1.836	0.000	PA14_26970	415.654	-1.757	0.000

PA14_26980	101.519	-0.659	0.143	-0.314	0.445	2.508	0.000	PA14_26980	527.448	-2.295	0.000
PA14_26990	27.753	-0.225	0.798	-0.661	0.298	-0.156	0.872	PA14_26990	670.191	-2.988	0.000
PA14_27000	1658.953	-0.263	0.381	-1.934	0.000	-2.333	0.000	PA14_27000	6470.636	0.069	0.792
PA14_27020	226.159	0.532	0.135	0.503	0.113	-0.330	0.485	PA14_27020	576.289	0.210	0.421
PA14_27050	547.895	-0.160	0.677	0.345	0.233	0.638	0.072	PA14_27050	1256.874	-1.019	0.000
PA14_27070	274.161	-0.760	0.120	-1.158	0.006	-1.219	0.025	PA14_27070	1358.856	-2.336	0.000
PA14_27090	30.255	1.236	0.040	-1.027	0.091	-1.466	0.086	PA14_27090	49.192	1.458	0.015
PA14_27100	118.406	1.018	0.024	-1.046	0.016	-1.097	0.060	PA14_27100	212.113	0.339	0.274
PA14_27110	20.468	0.176	0.851	0.021	0.978	0.909	0.251	PA14_27110	100.070	-1.591	0.001
PA14_27120	441.066	0.674	0.019	1.376	0.000	0.035	0.939	PA14_27120	1758.289	-0.799	0.001
PA14_27130	279.239	-0.236	0.595	-0.028	0.946	-0.117	0.828	PA14_27130	555.271	0.051	0.858
PA14_27140	349.591	-0.273	0.482	-0.334	0.296	-0.467	0.263	PA14_27140	620.709	0.548	0.041
PA14_27150	65.867	0.272	0.675	0.306	0.555	0.122	0.874	PA14_27150	321.062	-0.362	0.301
PA14_27160	228.132	-0.537	0.175	-0.707	0.039	-1.353	0.002	PA14_27160	941.556	0.051	0.846
PA14_27170	107.225	0.468	0.302	1.186	0.001	0.825	0.087	PA14_27170	318.475	1.123	0.005
PA14_27180	2244.754	-0.033	0.938	0.207	0.468	0.842	0.009	PA14_27180	5897.893	-0.202	0.356
PA14_27190	144.476	0.457	0.427	2.736	0.000	1.535	0.004	#N/A	#N/A	#N/A	#N/A
PA14_27200	607.145	0.304	0.471	0.346	0.326	0.016	0.976	PA14_27200	980.503	0.060	0.814
PA14_27210	7791.161	0.195	0.618	0.457	0.128	-0.128	0.780	PA14_27210	7724.986	2.222	0.000
PA14_27220	63.575	-0.785	0.101	-0.823	0.051	-2.753	0.000	PA14_27220	112.359	-0.628	0.097
PA14_27230	348.295	-0.006	0.991	-0.462	0.173	-1.099	0.009	PA14_27230	967.756	-1.743	0.000
PA14_27250	57.855	0.315	0.555	0.408	0.333	-0.773	0.207	PA14_27250	92.918	0.175	0.679
PA14_27270	66.955	0.866	0.041	-0.051	0.917	-0.811	0.172	PA14_27270	160.657	-0.521	0.089
PA14_27280	77.463	-0.016	0.979	0.311	0.438	0.508	0.327	PA14_27280	232.492	0.532	0.064
PA14_27290	19.609	-0.032	0.975	-0.337	0.623	-0.781	0.385	PA14_27290	132.105	-0.685	0.028
PA14_27310	137.377	-0.133	0.774	0.329	0.320	0.299	0.521	PA14_27310	294.739	-0.518	0.052
PA14_27330	289.099	-0.179	0.688	-0.668	0.038	-1.072	0.009	PA14_27330	1424.449	-1.351	0.000
PA14_27350	70.100	0.339	0.553	0.892	0.034	-0.404	0.551	PA14_27350	157.684	1.187	0.000
PA14_27360	34.255	-0.651	0.288	-0.721	0.164	-0.289	0.709	PA14_27360	154.424	-1.593	0.000
PA14_27370	2300.072	1.139	0.000	2.676	0.000	4.212	0.000	PA14_27370	655.647	2.127	0.000
PA14_27390	48.760	-0.219	0.731	-0.120	0.819	0.277	0.682	PA14_27390	174.400	-0.347	0.268
PA14_27400	21.941	0.037	0.970	-0.204	0.759	-0.137	0.887	PA14_27400	54.340	-0.545	0.213
PA14_27410	12.796	0.189	0.864	0.268	0.742	0.029	0.979	PA14_27410	14.937	0.642	0.470
PA14_27420	7.970	-0.567	0.623	-0.082	0.937	-0.336	0.790	PA14_27420	12.195	0.954	0.328
PA14_27430	50.645	0.899	0.124	1.960	0.000	1.241	0.053	PA14_27430	29.949	0.702	0.319
PA14_27440	476.749	0.875	0.012	0.943	0.003	0.872	0.037	PA14_27440	650.890	0.548	0.021
PA14_27450	73.601	0.064	0.920	0.194	0.662	-1.540	0.008	PA14_27450	217.144	-0.317	0.331
PA14_27460	238.544	-0.085	0.867	-0.053	0.896	-1.922	0.000	PA14_27460	323.642	-0.511	0.052
PA14_27470	423.460	-0.386	0.230	-0.176	0.564	-0.730	0.039	PA14_27470	666.815	0.566	0.016
PA14_27480	778.853	-1.395	0.000	-0.640	0.011	-1.153	0.000	PA14_27480	6362.161	0.037	0.938
PA14_27490	130.333	0.373	0.376	0.913	0.005	0.688	0.120	PA14_27490	201.559	2.973	0.000
PA14_27500	857.500	0.456	0.107	0.756	0.002	0.393	0.252	PA14_27500	1540.364	1.939	0.000
PA14_27510	431.055	-0.496	0.081	-0.688	0.006	-1.006	0.002	PA14_27510	1857.363	-1.188	0.000
PA14_27520	1013.990	0.026	0.947	-0.462	0.057	-1.711	0.000	PA14_27520	3539.059	-0.437	0.041
PA14_27530	140.142	-0.266	0.568	-0.609	0.088	-1.379	0.003	PA14_27530	991.534	-0.147	0.622
PA14_27550	623.377	-1.290	0.000	-1.785	0.000	-0.570	0.093	PA14_27550	2186.103	-1.608	0.000
PA14_27560	1332.537	0.735	0.122	1.621	0.000	-0.482	0.414	PA14_27560	1152.091	0.937	0.000
PA14_27570	268.470	0.180	0.636	0.264	0.379	-0.522	0.183	PA14_27570	477.143	0.426	0.094
PA14_27580	306.130	0.067	0.874	-0.546	0.048	-0.140	0.746	PA14_27580	866.071	-0.126	0.610
PA14_27590	1253.653	-0.057	0.885	-0.563	0.027	0.354	0.309	PA14_27590	4608.996	-1.227	0.000
PA14_27600	325.609	0.299	0.648	1.801	0.000	1.073	0.078	#N/A	#N/A	#N/A	#N/A
PA14_27610	172.650	0.812	0.178	1.968	0.000	1.196	0.069	#N/A	#N/A	#N/A	#N/A
PA14_27620	38.480	0.189	0.804	1.461	0.002	-0.629	0.427	#N/A	#N/A	#N/A	#N/A
PA14_27630	27.581	-0.647	0.369	0.236	0.707	1.633	0.010	PA14_27630	68.245	-0.902	0.028
PA14_27640	104.754	-1.153	0.027	-0.690	0.137	3.991	0.000	PA14_27640	105.910	-1.158	0.001
PA14_27650	44.206	-0.155	0.839	-0.558	0.298	0.932	0.142	PA14_27650	161.911	-1.346	0.000
PA14_27660	43.613	-0.258	0.704	-0.420	0.418	0.505	0.450	PA14_27660	167.454	-1.280	0.000
PA14_27675	72.866	-0.485	0.333	-0.886	0.030	0.521	0.341	PA14_27675	199.563	-0.605	0.039
PA14_27680	18.886	-0.103	0.920	0.026	0.973	1.442	0.054	PA14_27680	44.802	-1.250	0.144

PA14_27690	3.625	-0.416	0.799	-1.289	0.279	-0.250	0.871	PA14_27690	6.330	0.863	0.568
PA14_27700	26.263	0.814	0.266	2.160	0.000	2.375	0.000	PA14_27700	38.659	0.117	0.873
PA14_27710	199.793	-0.081	0.880	0.996	0.002	1.632	0.000	PA14_27710	342.418	-0.001	0.989
PA14_27720	125.164	-0.857	0.019	-1.593	0.000	0.394	0.391	PA14_27720	528.036	-1.455	0.000
PA14_27730	964.740	-1.810	0.000	-2.266	0.000	-0.140	0.715	PA14_27730	3600.029	-1.117	0.000
PA14_27740	115.722	-1.132	0.001	-2.778	0.000	-0.691	0.108	PA14_27740	718.144	-2.982	0.000
PA14_27755	350.493	-0.309	0.365	0.205	0.494	-0.632	0.087	PA14_27755	862.090	-0.490	0.040
PA14_27770	699.921	-0.119	0.744	0.244	0.372	-1.284	0.000	PA14_27770	1245.755	0.900	0.000
PA14_27780	478.206	-0.626	0.032	-0.385	0.164	-2.130	0.000	PA14_27780	655.650	0.977	0.000
PA14_27800	91.894	-0.045	0.936	-0.005	0.990	-0.110	0.851	PA14_27800	183.408	0.867	0.004
PA14_27810	54.290	-0.717	0.171	-0.807	0.075	-1.568	0.012	PA14_27810	212.080	0.006	0.997
PA14_27830	4.814	-0.650	0.635	-1.059	0.329	-0.660	0.624	PA14_27830	23.159	-1.706	0.016
PA14_27840	15.449	-1.003	0.181	-1.191	0.066	-0.475	0.611	PA14_27840	74.981	0.151	0.782
PA14_27850	27.064	0.116	0.897	1.057	0.049	-0.010	0.991	PA14_27850	91.568	0.559	0.137
PA14_27870	502.532	-1.032	0.001	-1.697	0.000	-2.222	0.000	PA14_27870	3081.472	-1.870	0.000
PA14_27880	12.425	0.001	0.999	-0.507	0.586	1.917	0.038	PA14_27880	119.946	-4.355	0.000
PA14_27890	6.531	-0.931	0.437	-0.571	0.581	-0.270	0.845	PA14_27890	120.493	-4.203	0.000
PA14_27900	98.431	0.374	0.477	0.234	0.608	0.508	0.372	PA14_27900	474.028	-0.764	0.001
PA14_27910	285.087	0.011	0.980	-0.320	0.279	0.151	0.729	PA14_27910	611.918	0.689	0.004
PA14_27920	662.548	-0.864	0.035	-0.810	0.032	0.005	0.992	PA14_27920	2795.206	-0.417	0.065
PA14_27930	166.107	-0.965	0.013	-1.605	0.000	-0.520	0.294	PA14_27930	1295.232	-2.931	0.000
PA14_27940	1979.476	-0.447	0.118	-0.039	0.902	0.728	0.022	PA14_27940	12847.220	-1.154	0.000
PA14_27950	642.051	-0.514	0.084	-0.326	0.244	0.688	0.040	PA14_27950	6972.119	-1.738	0.000
PA14_27960	2135.371	-0.980	0.000	-1.715	0.000	-1.063	0.000	PA14_27960	6052.214	-0.422	0.053
PA14_27980	477.740	1.172	0.000	1.803	0.000	2.242	0.000	PA14_27980	565.212	2.329	0.000
PA14_27990	1695.759	0.431	0.316	-0.280	0.475	0.665	0.155	PA14_27990	4698.648	-1.110	0.002
PA14_28000	368.306	-0.144	0.705	-0.412	0.143	0.258	0.516	PA14_28000	896.591	0.416	0.083
PA14_28010	36.528	-0.824	0.213	-0.540	0.361	-0.971	0.204	PA14_28010	123.889	-0.090	0.816
PA14_28020	2.470	-0.410	0.805	-1.710	0.149	0.485	0.741	PA14_28020	9.561	-0.475	0.705
PA14_28030	907.747	-0.278	0.471	-0.956	0.001	3.226	0.000	PA14_28030	1386.796	-1.478	0.000
PA14_28040	40.626	0.341	0.616	0.288	0.607	1.428	0.017	PA14_28040	80.258	0.766	0.050
PA14_28050	843.124	-3.445	0.000	-4.687	0.000	0.352	0.375	PA14_28050	2720.803	-3.735	0.000
PA14_28060	42.655	-2.872	0.000	-4.425	0.000	-0.057	0.941	PA14_28060	224.124	-3.689	0.000
PA14_28070	26.788	-0.966	0.134	-0.982	0.078	-1.653	0.042	PA14_28070	40.619	0.505	0.545
PA14_28080	9.590	0.366	0.760	0.615	0.494	1.016	0.340	PA14_28080	24.309	0.244	0.734
PA14_28090	15.634	-0.384	0.700	-0.027	0.974	-1.357	0.169	PA14_28090	47.942	1.170	0.012
PA14_28100	17.698	-0.231	0.818	-0.838	0.243	1.490	0.054	PA14_28100	29.589	0.503	0.421
PA14_28110	4.499	1.472	0.211	1.169	0.272	0.298	0.842	PA14_28110	37.641	-0.703	0.198
PA14_28120	32.030	-0.679	0.303	-0.329	0.574	-0.094	0.915	PA14_28120	505.115	-1.767	0.000
PA14_28130	137.362	-0.418	0.285	-0.893	0.005	0.211	0.666	PA14_28130	1305.008	-1.444	0.000
PA14_28140	78.384	-2.838	0.000	-3.061	0.000	0.338	0.517	PA14_28140	635.184	-3.736	0.000
PA14_28150	34.431	-1.503	0.006	-2.249	0.000	0.915	0.131	PA14_28150	253.881	-2.323	0.000
PA14_28170	449.874	-1.177	0.000	-0.952	0.000	0.483	0.166	PA14_28170	2496.488	-1.747	0.000
PA14_28180	974.591	1.644	0.000	0.413	0.120	-0.383	0.290	PA14_28180	1837.916	0.238	0.292
PA14_28190	24.245	0.854	0.351	2.665	0.000	1.114	0.242	#N/A	#N/A	#N/A	#N/A
PA14_28200	76.552	-0.092	0.885	0.375	0.381	0.026	0.970	PA14_28200	304.188	0.719	0.013
PA14_28210	138.719	0.264	0.574	0.621	0.078	-0.797	0.112	PA14_28210	304.913	0.493	0.275
PA14_28220	80.268	-0.134	0.833	-0.014	0.978	0.919	0.076	PA14_28220	172.443	-0.121	0.690
PA14_28230	43.412	-0.370	0.559	-0.493	0.327	0.287	0.688	PA14_28230	83.624	0.854	0.023
PA14_28240	263.738	0.507	0.154	1.143	0.000	-0.100	0.849	PA14_28240	594.828	0.815	0.001
PA14_28250	325.734	-1.157	0.000	-2.746	0.000	-0.200	0.657	PA14_28250	1295.996	-2.198	0.000
PA14_28260	647.790	0.632	0.045	1.312	0.000	1.319	0.000	PA14_28260	1270.336	-1.006	0.000
PA14_28280	654.410	0.432	0.143	1.033	0.000	-0.081	0.849	PA14_28280	591.158	0.519	0.032
PA14_28290	438.383	1.087	0.005	3.040	0.000	1.554	0.000	PA14_28290	236.922	0.549	0.211
PA14_28300	5.172	-0.173	0.916	1.443	0.147	0.695	0.598	PA14_28300	9.194	-0.247	0.884
PA14_28310	42.731	0.090	0.916	0.748	0.157	-0.408	0.620	PA14_28310	73.156	-0.271	0.506
PA14_28320	88.186	0.711	0.087	0.802	0.028	0.109	0.864	PA14_28320	252.040	-0.088	0.773
PA14_28330	26.486	0.656	0.342	1.625	0.002	0.013	0.989	PA14_28330	63.722	0.453	0.316
PA14_28340	597.552	-0.017	0.969	-0.242	0.392	-1.279	0.000	PA14_28340	824.241	0.107	0.667

PA14_28350	186.617	-0.259	0.515	0.100	0.777	0.499	0.218	PA14_28350	489.685	-0.723	0.003
PA14_28360	388.083	-0.601	0.105	-0.827	0.010	2.157	0.000	PA14_28360	469.285	0.673	0.006
PA14_28370	37.259	-0.178	0.835	0.209	0.739	3.009	0.000	PA14_28370	49.052	1.507	0.001
PA14_28380	96.194	-0.060	0.930	-0.298	0.527	1.439	0.004	PA14_28380	154.616	-0.284	0.395
PA14_28390	50.984	-0.206	0.756	0.434	0.366	0.253	0.719	PA14_28390	61.952	1.344	0.001
PA14_28400	6116.069	0.701	0.006	0.156	0.569	-0.868	0.004	PA14_28400	6902.913	1.575	0.000
PA14_28410	10.727	0.704	0.507	0.935	0.273	1.129	0.277	PA14_28410	154.135	-2.773	0.000
PA14_28420	163.020	0.374	0.366	0.569	0.090	0.462	0.320	PA14_28420	301.380	0.292	0.317
PA14_28430	124.899	1.408	0.000	1.541	0.000	1.598	0.000	PA14_28430	324.027	2.243	0.000
PA14_28440	557.274	-0.526	0.081	-0.679	0.011	0.221	0.579	PA14_28440	1810.011	-0.821	0.000
PA14_28450	2640.852	-0.607	0.061	-0.526	0.079	0.276	0.517	PA14_28450	7385.699	0.643	0.002
PA14_28460	1453.502	-1.310	0.000	-1.711	0.000	0.403	0.198	PA14_28460	8655.561	0.363	0.096
PA14_28470	113.952	2.143	0.000	3.631	0.000	1.087	0.047	PA14_28470	84.685	-0.895	0.300
PA14_28490	893.895	-1.670	0.000	-2.866	0.000	-1.239	0.002	PA14_28490	4594.067	-0.836	0.000
PA14_28500	597.851	-1.384	0.000	-3.333	0.000	-2.762	0.000	PA14_28500	3076.355	-0.744	0.020
PA14_28510	10.333	0.865	0.447	2.198	0.009	1.713	0.102	PA14_28510	24.692	0.419	0.564
PA14_28520	254.589	-0.575	0.159	0.396	0.259	4.150	0.000	PA14_28520	629.049	-0.943	0.000
PA14_28530	129.757	-1.035	0.010	-0.888	0.015	0.700	0.139	PA14_28530	387.233	-0.984	0.000
PA14_28540	9.701	0.018	0.989	0.785	0.366	1.550	0.114	PA14_28540	57.937	-1.917	0.000
PA14_28560	29.027	0.174	0.857	-0.346	0.633	-0.907	0.314	PA14_28560	85.872	0.032	0.990
PA14_28570	71.776	0.866	0.038	1.321	0.000	0.456	0.417	PA14_28570	102.071	1.512	0.000
PA14_28580	901.471	1.650	0.000	2.074	0.000	1.111	0.000	PA14_28580	1065.384	0.963	0.000
PA14_28590	138.596	0.963	0.005	1.109	0.000	0.688	0.120	PA14_28590	229.688	0.948	0.001
PA14_28600	2166.772	-1.040	0.000	-1.758	0.000	1.842	0.000	PA14_28600	1196.898	-1.315	0.000
PA14_28610	135.105	-2.137	0.000	-2.666	0.000	0.149	0.849	PA14_28610	1003.636	-3.236	0.000
PA14_28620	135.979	-1.581	0.001	-2.066	0.000	1.885	0.000	PA14_28620	473.860	-3.337	0.000
PA14_28630	58.979	0.600	0.354	1.671	0.000	3.632	0.000	PA14_28630	56.052	1.307	0.003
PA14_28650	14557.762	0.290	0.401	1.049	0.000	1.676	0.000	PA14_28650	25869.287	0.621	0.003
PA14_28660	149289.745	0.148	0.759	0.588	0.090	0.718	0.102	PA14_28660	93672.749	0.631	0.003
PA14_28670	13069.696	0.558	0.189	0.365	0.359	0.466	0.360	PA14_28670	15426.123	0.847	0.000
PA14_28680	2726.214	0.019	0.977	0.048	0.925	-0.019	0.977	PA14_28680	4110.677	1.003	0.000
PA14_28690	1192.102	0.244	0.493	0.338	0.247	0.803	0.020	PA14_28690	1155.627	1.731	0.000
PA14_28710	2105.478	-0.235	0.527	-0.504	0.084	0.293	0.477	PA14_28710	21948.083	-1.549	0.000
PA14_28720	5032.342	-1.015	0.000	-1.337	0.000	-1.266	0.000	PA14_28720	42686.238	-1.642	0.000
PA14_28730	979.536	-1.120	0.004	-1.545	0.000	-1.328	0.004	PA14_28730	7903.020	-1.441	0.000
PA14_28740	158.332	0.745	0.067	2.451	0.000	1.669	0.000	#N/A	#N/A	#N/A	#N/A
PA14_28750	176.160	-0.214	0.690	0.435	0.275	0.979	0.038	PA14_28750	418.708	-0.562	0.205
PA14_28760	100.340	0.395	0.400	0.394	0.318	-0.235	0.697	PA14_28760	373.109	-0.513	0.047
PA14_28770	4186.696	-0.237	0.441	0.923	0.000	0.593	0.056	PA14_28770	6915.295	0.821	0.013
PA14_28780	615.836	-0.344	0.761	-0.445	0.620	-0.182	0.880	PA14_28780	1949.894	-1.490	0.072
PA14_28790	250.260	0.024	0.974	0.584	0.178	1.021	0.052	PA14_28790	1010.923	0.306	0.212
PA14_28800	1484.699	0.084	0.851	0.683	0.021	0.041	0.933	PA14_28800	4521.301	0.154	0.611
PA14_28810	12003.815	-0.268	0.417	-0.427	0.115	-0.863	0.008	PA14_28810	34600.939	0.357	0.098
PA14_28820	6126.974	-0.598	0.013	-0.822	0.000	-1.448	0.000	PA14_28820	19474.589	-0.417	0.055
PA14_28830	1892.525	-1.172	0.000	-1.997	0.000	-2.968	0.000	PA14_28830	5527.155	-0.608	0.004
PA14_28840	13028.922	-0.429	0.239	-1.032	0.001	-1.863	0.000	PA14_28840	30988.514	-0.691	0.044
PA14_28850	922.361	-1.196	0.000	-2.293	0.000	-2.769	0.000	PA14_28850	5126.567	-1.296	0.000
PA14_28870	37.928	-0.538	0.351	-0.481	0.322	-0.530	0.440	PA14_28870	728.423	-1.147	0.001
PA14_28880	131.901	-0.276	0.522	-1.256	0.000	-2.855	0.000	PA14_28880	682.034	-1.829	0.000
PA14_28895	592.525	-0.442	0.121	-1.079	0.000	-2.210	0.000	PA14_28895	1323.360	-0.689	0.002
PA14_28910	48.233	-0.250	0.704	-1.210	0.011	-1.126	0.086	PA14_28910	136.835	0.275	0.409
PA14_28920	396.984	-0.290	0.405	-0.706	0.010	-1.228	0.000	PA14_28920	930.073	-0.036	0.891
PA14_28930	24.357	0.250	0.748	-0.947	0.104	0.477	0.542	PA14_28930	71.349	-0.562	0.179
PA14_28940	232.515	-0.531	0.123	-1.203	0.000	-0.234	0.605	PA14_28940	596.465	-1.691	0.000
PA14_28950	152.047	-1.409	0.004	-1.145	0.011	1.647	0.002	PA14_28950	287.038	-2.687	0.000
PA14_28960	85.218	-1.739	0.000	-3.451	0.000	0.985	0.029	PA14_28960	304.396	-3.191	0.000
PA14_28970	15.039	-0.294	0.792	0.543	0.494	1.360	0.126	PA14_28970	14.936	0.983	0.240
PA14_28980	5.069	0.061	0.974	0.273	0.829	-0.073	0.963	PA14_28980	11.236	0.602	0.553
PA14_28990	116.787	0.323	0.494	0.350	0.368	0.998	0.027	PA14_28990	258.163	0.271	0.366

PA14_29000	85.073	-0.226	0.684	-0.709	0.079	0.123	0.849	PA14_29000	331.727	-0.593	0.023
PA14_29010	255.364	-0.816	0.009	-0.873	0.002	-0.840	0.028	PA14_29010	923.454	-1.001	0.000
PA14_29020	154.500	-1.620	0.000	-1.400	0.000	0.580	0.247	PA14_29020	603.463	-1.855	0.000
PA14_29030	67.122	-0.100	0.870	-0.511	0.220	-0.209	0.743	PA14_29030	219.835	-0.436	0.147
PA14_29040	8.804	1.071	0.263	0.465	0.609	-0.547	0.660	PA14_29040	27.526	-0.312	0.671
PA14_29050	73.102	0.902	0.028	0.861	0.021	-0.029	0.966	PA14_29050	135.941	0.613	0.054
PA14_29060	204.326	-0.171	0.692	0.027	0.944	0.349	0.423	PA14_29060	574.603	-0.179	0.463
PA14_29070	34.792	0.710	0.233	1.168	0.016	-0.041	0.964	PA14_29070	118.940	-0.380	0.292
PA14_29090	399.517	1.288	0.000	-0.636	0.020	-0.133	0.758	PA14_29090	353.143	0.775	0.002
PA14_29100	51.697	-0.211	0.740	-0.689	0.134	-0.312	0.652	PA14_29100	136.998	0.968	0.002
PA14_29110	1376.641	-0.259	0.564	-1.026	0.002	-1.890	0.000	PA14_29110	4236.712	-0.120	0.635
PA14_29120	230.948	-0.560	0.139	-0.280	0.430	0.894	0.027	PA14_29120	836.871	-1.207	0.000
PA14_29130	1321.323	-0.364	0.296	-0.955	0.001	-1.409	0.000	PA14_29130	5636.116	-0.699	0.001
PA14_29150	413.909	0.315	0.337	0.580	0.029	-0.023	0.962	PA14_29150	1183.002	-0.099	0.731
PA14_29160	585.075	-0.517	0.096	-0.681	0.013	-1.593	0.000	PA14_29160	2131.694	0.225	0.335
PA14_29180	50.496	0.838	0.076	0.782	0.065	-0.242	0.743	PA14_29180	101.475	0.351	0.517
PA14_29190	19.068	-0.669	0.437	-1.165	0.093	-0.633	0.515	PA14_29190	76.759	-0.312	0.463
PA14_29200	93.020	-0.154	0.773	-0.116	0.788	1.502	0.000	PA14_29200	194.342	-0.088	0.818
PA14_29210	9.314	-1.509	0.094	-4.211	0.000	-0.707	0.513	PA14_29210	51.056	-0.914	0.056
PA14_29220	24.408	-0.834	0.251	-1.392	0.023	0.959	0.195	PA14_29220	129.359	-1.919	0.000
PA14_29230	206.322	-1.809	0.000	-2.338	0.000	0.252	0.541	PA14_29230	1021.656	-1.964	0.000
PA14_29240	286.141	-0.625	0.060	-1.196	0.000	-2.941	0.000	PA14_29240	785.806	-0.546	0.021
PA14_29250	22.789	-0.170	0.855	-0.326	0.633	-1.692	0.057	PA14_29250	62.858	-0.135	0.880
PA14_29260	207.566	-0.125	0.804	0.191	0.612	0.683	0.117	PA14_29260	657.661	-0.248	0.326
PA14_29270	479.942	0.549	0.243	0.974	0.013	1.234	0.011	PA14_29270	506.687	0.292	0.267
PA14_29280	47.806	-0.035	0.964	0.506	0.263	0.803	0.160	PA14_29280	93.844	-0.032	0.955
PA14_29290	100.954	0.958	0.096	1.520	0.002	1.415	0.024	PA14_29290	192.332	-0.231	0.559
PA14_29300	110.392	1.389	0.003	2.279	0.000	3.611	0.000	PA14_29300	206.249	-0.927	0.001
PA14_29320	64.548	-0.127	0.879	-0.123	0.845	2.880	0.000	PA14_29320	142.662	-2.062	0.000
PA14_29330	485.241	-2.710	0.000	-4.384	0.000	-0.463	0.191	PA14_29330	2921.156	-4.928	0.000
PA14_29340	4.166	0.052	0.975	-0.280	0.814	0.171	0.907	PA14_29340	13.493	0.152	0.917
PA14_29350	117.719	-0.154	0.766	1.115	0.001	1.839	0.000	PA14_29350	114.433	-0.371	0.296
PA14_29360	365.576	-0.201	0.615	1.569	0.000	1.862	0.000	PA14_29360	155.393	1.092	0.000
PA14_29375	128.799	-0.427	0.366	0.882	0.013	1.710	0.000	#N/A	#N/A	#N/A	#N/A
PA14_29390	872.728	-0.982	0.000	-0.882	0.000	-1.087	0.000	PA14_29390	3349.660	-0.721	0.001
PA14_29400	665.038	-1.216	0.000	-1.356	0.000	-2.128	0.000	PA14_29400	2202.037	-1.354	0.000
PA14_29410	331.283	-0.721	0.047	-0.760	0.020	-0.735	0.090	PA14_29410	1251.437	-1.491	0.000
PA14_29420	176.512	-0.339	0.361	-0.949	0.001	-0.082	0.869	PA14_29420	311.167	-0.716	0.006
PA14_29440	47.659	-0.164	0.816	0.195	0.712	-0.235	0.755	PA14_29440	221.565	-0.268	0.363
PA14_29460	19.853	-0.263	0.769	-0.287	0.682	-0.626	0.501	PA14_29460	93.322	-0.633	0.085
PA14_29470	70.327	-0.397	0.430	-0.479	0.245	-0.122	0.851	PA14_29470	324.445	-0.801	0.002
PA14_29480	77.799	-0.007	0.991	0.991	0.025	0.804	0.176	PA14_29480	130.000	0.089	0.843
PA14_29490	126.669	0.025	0.966	0.398	0.247	-0.086	0.879	PA14_29490	358.142	0.002	0.999
PA14_29500	56.460	0.292	0.618	0.594	0.173	1.110	0.036	PA14_29500	130.219	0.344	0.331
PA14_29510	19.881	-0.109	0.919	0.405	0.573	-0.432	0.672	PA14_29510	78.437	0.395	0.359
PA14_29520	18.742	0.834	0.303	1.898	0.002	1.081	0.215	PA14_29520	79.318	0.216	0.626
PA14_29530	5.994	-1.476	0.171	-0.202	0.847	-1.031	0.386	PA14_29530	24.424	-0.194	0.803
PA14_29540	16.016	0.034	0.975	0.356	0.616	0.180	0.863	PA14_29540	29.386	0.781	0.204
PA14_29550	91.614	0.231	0.672	0.783	0.040	1.496	0.001	PA14_29550	198.101	-0.133	0.647
PA14_29560	97.974	-0.006	0.991	-0.379	0.326	0.210	0.707	PA14_29560	326.360	-0.286	0.316
PA14_29570	55.855	-0.492	0.382	-0.446	0.347	0.291	0.662	PA14_29570	202.119	-0.630	0.055
PA14_29575	483.829	-0.997	0.000	-1.335	0.000	-0.866	0.007	PA14_29575	1861.401	-1.524	0.001
PA14_29590	1253.292	0.177	0.681	0.600	0.056	-1.433	0.000	PA14_29590	2562.417	-0.110	0.670
PA14_29600	1893.227	1.762	0.000	2.915	0.000	3.361	0.000	PA14_29600	1798.200	2.143	0.000
PA14_29620	332.897	0.859	0.004	0.700	0.012	-0.053	0.914	PA14_29620	695.396	-0.368	0.116
PA14_29640	2704.060	2.545	0.000	-0.610	0.063	6.585	0.000	PA14_29640	3613.182	-4.320	0.000
PA14_29650	81.577	-1.004	0.027	-2.983	0.000	2.761	0.000	PA14_29650	389.082	-3.029	0.000
PA14_29660	408.716	-1.035	0.004	-2.108	0.000	4.932	0.000	PA14_29660	682.476	-2.014	0.000
PA14_29680	234.144	-0.035	0.938	-0.079	0.814	-1.085	0.004	PA14_29680	336.521	0.301	0.269

PA14_29690	149.534	0.647	0.056	0.404	0.206	-0.682	0.129	PA14_29690	298.754	1.127	0.000
PA14_29710	1634.244	-1.004	0.000	-1.610	0.000	-0.047	0.906	PA14_29710	3320.675	-0.855	0.014
PA14_29720	404.840	-0.844	0.007	-0.976	0.001	0.352	0.390	PA14_29720	1206.176	-0.638	0.025
PA14_29730	346.521	-0.042	0.926	0.001	0.998	1.531	0.000	PA14_29730	753.477	0.221	0.362
PA14_29740	541.801	0.384	0.267	0.795	0.005	2.137	0.000	PA14_29740	695.669	-0.051	0.867
PA14_29750	89.902	0.047	0.948	0.447	0.322	2.292	0.000	PA14_29750	229.309	0.416	0.780
PA14_29760	623.535	0.043	0.918	-0.789	0.002	0.614	0.066	PA14_29760	1874.131	-0.619	0.005
PA14_29770	58.607	0.428	0.435	1.454	0.000	-1.366	0.040	PA14_29770	68.630	1.166	0.003
PA14_29800	1158.696	-0.749	0.009	-0.334	0.241	-0.682	0.050	PA14_29800	2642.679	-0.391	0.094
PA14_29820	2353.552	-0.950	0.000	-0.595	0.016	0.121	0.754	PA14_29820	6995.624	-0.151	0.492
PA14_29830	39.953	0.149	0.834	0.723	0.115	0.435	0.523	PA14_29830	49.867	0.816	0.081
PA14_29850	1092.065	-0.110	0.778	-0.318	0.271	-1.320	0.000	PA14_29850	1453.127	0.442	0.055
PA14_29860	973.268	-0.342	0.240	-0.499	0.044	-1.106	0.000	PA14_29860	1566.724	0.364	0.109
PA14_29880	1680.159	-0.394	0.161	-0.772	0.001	-1.046	0.000	PA14_29880	3188.796	0.147	0.539
PA14_29890	254.956	-0.753	0.060	-1.194	0.001	-1.355	0.003	PA14_29890	1036.351	-0.339	0.160
PA14_29900	305.724	-0.452	0.169	-0.781	0.005	-0.881	0.017	PA14_29900	1092.348	-0.540	0.019
PA14_29920	952.302	-0.294	0.398	-0.714	0.009	-1.280	0.000	PA14_29920	2431.472	-0.014	0.970
PA14_29930	998.598	-0.417	0.135	-0.702	0.003	-0.782	0.011	PA14_29930	1974.843	0.319	0.163
PA14_29940	6204.534	0.277	0.392	0.023	0.943	-0.359	0.317	PA14_29940	11095.152	-0.214	0.352
PA14_29970	2881.599	-0.014	0.975	0.092	0.774	-0.276	0.484	PA14_29970	3727.541	0.470	0.033
PA14_29980	601.972	0.255	0.486	-0.190	0.554	-0.828	0.024	PA14_29980	1254.145	0.352	0.122
PA14_29990	7242.369	0.317	0.261	0.456	0.058	0.095	0.802	PA14_29990	9197.077	1.133	0.000
PA14_30010	5073.024	0.639	0.053	0.936	0.001	1.031	0.005	PA14_30010	6640.882	0.753	0.000
PA14_30020	4935.528	0.731	0.010	1.010	0.000	0.420	0.248	PA14_30020	14381.594	-0.363	0.094
PA14_30030	39.272	-0.133	0.875	-1.222	0.029	1.611	0.010	PA14_30030	105.835	-1.570	0.004
PA14_30040	147.152	0.249	0.621	0.270	0.509	2.000	0.000	PA14_30040	468.647	-1.283	0.004
PA14_30050	7666.870	-0.445	0.143	-1.235	0.000	0.515	0.142	PA14_30050	15671.089	-0.303	0.247
PA14_30070	735.942	-1.126	0.000	-1.469	0.000	1.361	0.000	PA14_30070	3565.864	-0.192	0.426
PA14_30080	442.526	-0.812	0.004	-0.729	0.005	1.663	0.000	PA14_30080	2480.623	-0.200	0.379
PA14_30090	491.192	-0.990	0.037	-1.704	0.000	-0.664	0.257	PA14_30090	3400.781	-0.302	0.180
PA14_30100	2504.390	-0.243	0.471	-0.153	0.607	0.901	0.005	PA14_30100	8305.899	-0.050	0.832
PA14_30110	3973.400	0.287	0.487	1.374	0.000	3.052	0.000	PA14_30110	1479.570	0.970	0.000
PA14_30130	160.777	0.181	0.744	0.806	0.035	1.393	0.002	PA14_30130	264.019	0.972	0.000
PA14_30140	75.999	0.934	0.067	1.377	0.002	0.977	0.101	PA14_30140	263.385	1.602	0.000
PA14_30150	1036.189	0.430	0.166	1.173	0.000	1.139	0.000	PA14_30150	2146.491	1.273	0.000
PA14_30160	150.852	0.032	0.954	-0.075	0.852	1.671	0.000	PA14_30160	220.683	1.729	0.000
PA14_30180	8330.881	0.535	0.063	0.399	0.141	1.533	0.000	PA14_30180	6463.691	1.775	0.000
PA14_30190	26912.383	-0.369	0.164	-0.900	0.000	-0.836	0.003	PA14_30190	37305.283	0.072	0.738
PA14_30200	4330.372	-1.355	0.000	-2.185	0.000	-1.638	0.000	PA14_30200	12247.393	-1.215	0.000
PA14_30210	11930.046	-1.178	0.000	-1.542	0.000	1.088	0.004	PA14_30210	79147.424	-3.109	0.000
PA14_30230	10653.695	-1.126	0.000	-1.361	0.000	1.009	0.000	PA14_30230	80362.281	-2.601	0.000
PA14_30240	659.850	0.342	0.360	1.847	0.000	2.222	0.000	PA14_30240	1692.791	0.729	0.001
PA14_30260	1098.539	-1.751	0.000	-2.267	0.000	0.279	0.501	PA14_30260	8110.376	-3.541	0.000
PA14_30270	266.070	1.000	0.002	1.287	0.000	-0.543	0.228	PA14_30270	545.984	2.172	0.000
PA14_30280	5142.560	0.046	0.920	-0.224	0.481	-1.735	0.000	PA14_30280	7389.703	0.630	0.003
PA14_30290	4085.500	0.167	0.578	0.648	0.004	-0.210	0.527	PA14_30290	7900.154	1.399	0.000
PA14_30310	3012.118	0.628	0.073	0.764	0.015	0.360	0.421	PA14_30310	4831.045	1.199	0.000
PA14_30320	5128.600	0.590	0.144	1.241	0.000	0.852	0.059	PA14_30320	6332.458	1.296	0.000
PA14_30330	3039.951	0.622	0.012	1.249	0.000	-0.367	0.254	PA14_30330	4591.334	1.763	0.000
PA14_30340	1192.467	0.421	0.102	0.906	0.000	-0.355	0.267	PA14_30340	2060.305	1.424	0.000
PA14_30350	194.808	-0.499	0.171	-0.087	0.815	0.914	0.017	PA14_30350	505.165	-0.316	0.211
PA14_30360	1327.259	-0.211	0.554	-0.248	0.398	0.320	0.399	PA14_30360	2214.728	0.450	0.159
PA14_30370	215.578	-0.218	0.597	0.137	0.693	0.811	0.033	PA14_30370	828.696	-1.037	0.006
PA14_30380	153.975	-0.348	0.368	-0.315	0.337	0.525	0.198	PA14_30380	850.322	-0.625	0.034
PA14_30390	196.879	-0.423	0.264	0.043	0.911	0.344	0.438	PA14_30390	742.378	-1.221	0.000
PA14_30400	152.442	-0.386	0.347	0.043	0.916	0.721	0.086	PA14_30400	671.701	-1.286	0.000
PA14_30410	718.791	-1.299	0.001	-1.477	0.000	-0.258	0.640	PA14_30410	6168.675	-1.152	0.000
PA14_30420	34.348	0.814	0.360	1.540	0.029	0.620	0.539	#N/A	#N/A	#N/A	#N/A
PA14_30430	316.020	-1.098	0.001	-1.359	0.000	-0.958	0.017	PA14_30430	470.525	-0.225	0.432

PA14_30440	108.121	-0.550	0.195	-1.240	0.001	-0.047	0.938	PA14_30440	250.583	-0.795	0.004
PA14_30450	679.243	-0.381	0.396	0.128	0.760	1.198	0.006	PA14_30450	720.962	-0.207	0.459
PA14_30460	33.140	-1.579	0.006	-0.967	0.059	0.309	0.677	PA14_30460	55.206	-0.394	0.369
PA14_30470	136.354	-0.099	0.853	0.368	0.313	0.762	0.087	PA14_30470	222.271	-0.231	0.426
PA14_30490	36.387	-0.987	0.070	-0.978	0.041	-1.222	0.067	PA14_30490	57.204	0.741	0.091
PA14_30500	33.505	-0.851	0.164	-0.069	0.912	-0.019	0.982	PA14_30500	64.012	0.340	0.474
PA14_30520	19.333	-0.488	0.545	-0.447	0.501	0.288	0.751	PA14_30520	39.566	0.385	0.493
PA14_30540	14.910	-1.176	0.177	0.031	0.972	0.461	0.651	PA14_30540	21.345	0.565	0.451
PA14_30550	58.125	-0.720	0.185	-0.520	0.279	-0.002	0.998	PA14_30550	66.551	0.449	0.305
PA14_30560	58.409	0.089	0.903	0.158	0.767	0.612	0.328	PA14_30560	210.110	-0.843	0.003
PA14_30570	1880.601	-0.504	0.147	-1.302	0.000	0.334	0.437	PA14_30570	3804.154	-1.616	0.000
PA14_30580	2704.339	-0.325	0.328	-0.957	0.000	-0.937	0.006	PA14_30580	14879.540	-0.032	0.940
PA14_30590	162.072	0.045	0.936	0.660	0.049	2.483	0.000	PA14_30590	407.414	-0.203	0.441
PA14_30600	17.425	-1.376	0.060	-1.230	0.057	-0.397	0.675	PA14_30600	44.326	-0.188	0.829
PA14_30620	736.827	-2.545	0.000	-3.488	0.000	-2.065	0.000	PA14_30620	2675.505	-2.084	0.000
PA14_30630	1089.653	-1.535	0.000	-2.620	0.000	-3.180	0.000	PA14_30630	2183.622	0.698	0.001
PA14_30650	9580.251	0.289	0.594	0.982	0.016	1.246	0.013	PA14_30650	14181.780	-0.881	0.108
PA14_30660	1085.766	-0.189	0.656	-0.474	0.140	-0.411	0.341	PA14_30660	3109.330	0.426	0.054
PA14_30670	1837.243	0.155	0.726	1.597	0.000	0.521	0.214	PA14_30670	2952.092	1.109	0.000
PA14_30680	224.606	1.136	0.013	3.678	0.000	1.976	0.000	#N/A	#N/A	#N/A	#N/A
PA14_30690	1440.759	-1.310	0.000	-1.672	0.000	0.125	0.821	PA14_30690	6905.710	-2.334	0.000
PA14_30700	1496.261	0.116	0.756	0.410	0.128	-0.384	0.286	PA14_30700	2806.561	-0.253	0.270
PA14_30710	840.394	-0.338	0.401	-0.336	0.329	-0.416	0.351	PA14_30710	4281.128	-0.626	0.030
PA14_30720	25.352	0.771	0.457	2.389	0.002	1.451	0.140	#N/A	#N/A	#N/A	#N/A
PA14_30730	836.098	0.020	0.963	-0.194	0.468	-0.205	0.572	PA14_30730	2269.141	0.178	0.449
PA14_30740	56.133	0.932	0.065	1.542	0.000	2.407	0.000	PA14_30740	130.962	-0.391	0.254
PA14_30750	1049.950	0.188	0.693	0.057	0.894	0.370	0.446	PA14_30750	1783.563	0.572	0.008
PA14_30760	140.083	-0.634	0.091	-1.156	0.000	-1.137	0.010	PA14_30760	664.963	-0.059	0.838
PA14_30770	78.481	-0.910	0.054	-1.677	0.000	-1.353	0.016	PA14_30770	440.945	-0.523	0.038
PA14_30790	173.552	-0.064	0.901	0.551	0.089	-0.308	0.531	PA14_30790	729.529	0.522	0.031
PA14_30800	848.464	-0.071	0.874	-0.300	0.331	-2.262	0.000	PA14_30800	1570.062	-0.665	0.002
PA14_30810	114.357	-0.589	0.133	-0.675	0.048	-0.115	0.838	PA14_30810	371.630	1.007	0.000
PA14_30820	1315.605	-1.483	0.000	-2.286	0.000	1.709	0.000	PA14_30820	6392.121	-3.452	0.000
PA14_30830	754.731	-2.299	0.000	-3.530	0.000	1.532	0.000	PA14_30830	4601.107	-4.163	0.000
PA14_30840	978.476	-1.688	0.000	-1.792	0.000	1.432	0.000	PA14_30840	2543.821	-2.268	0.000
PA14_30850	78.355	-0.325	0.581	0.731	0.085	1.567	0.002	PA14_30850	187.721	-0.639	0.031
PA14_30860	8.040	-0.293	0.822	0.895	0.297	-0.415	0.747	PA14_30860	23.547	-0.246	0.776
PA14_30870	3.574	0.338	0.823	-0.447	0.706	-1.335	0.281	PA14_30870	11.539	0.116	0.966
PA14_30880	12.251	-1.370	0.115	-1.349	0.076	0.033	0.977	PA14_30880	25.514	-0.901	0.168
PA14_30890	0.636	0.095	0.957	0.945	0.431	1.065	0.343	PA14_30890	1.005	-0.308	1.000
PA14_30900	16.065	0.159	0.880	-0.127	0.876	0.879	0.314	PA14_30900	57.451	-2.075	0.000
PA14_30910	28.648	-0.853	0.150	-0.363	0.504	-0.900	0.212	PA14_30910	85.171	-1.381	0.000
PA14_30930	1.843	-1.156	0.423	-2.583	0.025	-1.257	0.327	PA14_30930	5.810	-2.095	0.141
PA14_30940	7.436	0.114	0.936	-0.380	0.708	-0.371	0.775	PA14_30940	19.035	-0.856	0.270
PA14_30950	3.761	0.689	0.633	1.295	0.241	0.700	0.613	PA14_30950	10.602	0.615	0.606
PA14_30960	93.490	0.287	0.704	1.728	0.001	2.007	0.001	PA14_30960	111.748	1.146	0.001
PA14_30970	758.313	0.088	0.809	0.032	0.917	0.246	0.490	PA14_30970	2810.127	-0.852	0.000
PA14_30980	1025.288	-0.130	0.839	-0.725	0.097	-1.390	0.008	PA14_30980	1571.371	0.316	0.258
PA14_30990	147.286	-0.159	0.736	-0.305	0.390	0.285	0.555	PA14_30990	269.281	0.700	0.009
PA14_31000	70.247	-0.063	0.924	-0.078	0.874	0.028	0.968	PA14_31000	219.418	1.163	0.000
PA14_31010	929.676	0.179	0.578	0.599	0.012	0.698	0.022	PA14_31010	1857.196	1.117	0.000
PA14_31030	206.658	-0.154	0.701	-0.147	0.648	-1.513	0.000	PA14_31030	652.265	1.802	0.000
PA14_31040	625.065	0.950	0.007	1.367	0.000	0.034	0.952	PA14_31040	1133.072	1.484	0.000
PA14_31050	1039.495	0.254	0.494	0.399	0.184	-0.635	0.095	PA14_31050	2493.968	0.485	0.025
PA14_31060	8258.640	1.527	0.000	1.680	0.000	0.601	0.087	PA14_31060	8864.639	0.200	0.376
PA14_31070	20.220	0.729	0.364	1.478	0.016	1.039	0.218	PA14_31070	63.540	1.269	0.002
PA14_31080	2.465	-0.462	0.778	0.607	0.632	0.552	0.703	PA14_31080	3.791	2.519	0.213
PA14_31090	4.358	1.414	0.268	1.908	0.073	1.573	0.207	PA14_31090	34.588	0.289	0.630
PA14_31100	3.512	-1.313	0.305	-0.213	0.862	-0.101	0.945	PA14_31100	21.100	0.537	0.476

PA14_31110	9.484	-0.240	0.851	-0.194	0.845	1.631	0.088	PA14_31110	20.445	0.544	0.488
PA14_31130	23.418	1.422	0.073	1.989	0.003	3.685	0.000	PA14_31130	92.146	0.706	0.063
PA14_31150	243.390	-0.099	0.844	0.134	0.721	1.569	0.000	PA14_31150	1082.131	-1.437	0.000
PA14_31160	76.745	1.071	0.013	1.549	0.000	0.782	0.153	PA14_31160	207.384	1.399	0.000
PA14_31170	2.870	1.775	0.177	2.558	0.023	2.592	0.033	PA14_31170	6.838	2.841	0.037
PA14_31180	88.157	-0.389	0.494	-0.589	0.197	-0.475	0.456	PA14_31180	451.811	-0.767	0.002
PA14_31190	77.407	0.028	0.966	0.873	0.017	1.178	0.011	PA14_31190	120.166	-0.117	0.758
PA14_31200	25.391	0.864	0.197	1.536	0.004	0.758	0.345	PA14_31200	25.997	-0.013	1.000
PA14_31220	3.113	-0.934	0.492	-1.004	0.375	-1.113	0.386	PA14_31220	12.920	-0.098	1.000
PA14_31230	98.262	1.209	0.012	1.864	0.000	2.232	0.000	PA14_31230	140.398	0.316	0.372
PA14_31240	534.758	0.016	0.974	-0.555	0.054	-0.372	0.352	PA14_31240	2932.561	-2.633	0.000
PA14_31250	2.377	0.494	0.757	0.082	0.954	0.557	0.700	PA14_31250	4.003	1.392	0.473
PA14_31260	1.658	-0.240	0.896	-1.668	0.168	-0.043	0.977	PA14_31260	4.562	-0.622	0.756
PA14_31270	45.430	-0.524	0.344	-0.980	0.030	-0.478	0.470	PA14_31270	195.997	-0.996	0.000
PA14_31280	110.540	-0.173	0.730	-0.579	0.111	1.022	0.017	PA14_31280	235.681	-0.437	0.147
PA14_31290	286.535	-4.734	0.000	-5.482	0.000	0.169	0.796	PA14_31290	3615.368	-6.437	0.000
PA14_31300	1207.876	-0.273	0.392	-0.283	0.299	-0.014	0.974	PA14_31300	5505.118	-0.772	0.000
PA14_31310	502.743	-0.111	0.766	0.052	0.870	0.724	0.026	PA14_31310	1629.987	-0.293	0.383
PA14_31330	180.679	-0.530	0.151	-1.585	0.000	-1.363	0.001	PA14_31330	785.486	1.236	0.000
PA14_31340	15.285	-1.169	0.154	-1.107	0.121	-0.526	0.601	PA14_31340	32.269	-1.088	0.054
PA14_31350	409.133	-2.993	0.000	-3.171	0.000	0.100	0.872	PA14_31350	1654.714	-3.043	0.000
PA14_31360	68.221	-1.320	0.002	-1.845	0.000	0.662	0.195	PA14_31360	369.088	-2.177	0.000
PA14_31370	356.298	-0.828	0.046	-0.453	0.252	0.837	0.079	PA14_31370	1711.723	-1.982	0.000
PA14_31380	77.885	0.507	0.301	1.231	0.001	2.482	0.000	PA14_31380	78.736	-0.268	0.504
PA14_31390	208.287	-1.957	0.000	-2.353	0.000	-0.555	0.176	PA14_31390	1056.833	-1.788	0.000
PA14_31400	150.812	-0.023	0.966	0.175	0.626	-0.137	0.793	PA14_31400	269.589	0.240	0.411
PA14_31420	686.606	0.465	0.148	1.279	0.000	2.656	0.000	PA14_31420	1217.563	0.194	0.421
PA14_31430	242.401	0.012	0.981	1.690	0.000	1.633	0.000	PA14_31430	697.202	1.118	0.000
PA14_31440	1972.952	-0.233	0.618	0.525	0.143	0.809	0.068	PA14_31440	2218.288	0.243	0.321
PA14_31450	210.637	0.520	0.178	1.830	0.000	3.107	0.000	PA14_31450	570.090	-1.530	0.000
PA14_31460	310.158	0.861	0.009	1.519	0.000	1.579	0.000	PA14_31460	627.260	1.401	0.000
PA14_31470	909.283	-1.310	0.000	-2.684	0.000	-2.391	0.000	PA14_31470	1426.643	1.721	0.000
PA14_31480	121.610	-1.140	0.001	-1.381	0.000	-1.977	0.000	PA14_31480	280.235	0.296	0.316
PA14_31500	1693.513	-1.580	0.000	-3.123	0.000	0.051	0.916	PA14_31500	1552.097	1.107	0.001
PA14_31510	1215.176	-1.844	0.000	-4.574	0.000	-1.318	0.002	PA14_31510	1339.111	-0.674	0.002
PA14_31530	1113.395	-2.066	0.000	-5.845	0.000	-2.140	0.000	PA14_31530	1535.010	-0.739	0.001
PA14_31540	1041.278	-1.835	0.000	-4.838	0.000	-2.331	0.000	PA14_31540	1564.376	-0.511	0.038
PA14_31560	840.945	1.442	0.000	1.795	0.000	1.440	0.000	PA14_31560	1030.463	1.005	0.000
PA14_31580	321.274	-0.383	0.224	-0.548	0.040	0.670	0.045	PA14_31580	502.698	-0.228	0.377
PA14_31610	237.093	-0.253	0.534	0.077	0.834	0.308	0.490	PA14_31610	980.838	-1.073	0.000
PA14_31620	31.514	0.132	0.870	0.257	0.656	0.604	0.398	PA14_31620	100.745	-1.302	0.000
PA14_31630	47.363	0.210	0.797	0.131	0.842	0.304	0.717	PA14_31630	67.134	0.994	0.015
PA14_31640	105.435	0.371	0.593	0.968	0.063	0.285	0.720	PA14_31640	122.284	0.186	0.739
PA14_31650	976.075	0.542	0.064	0.769	0.003	-0.554	0.119	PA14_31650	1889.960	1.823	0.000
PA14_31660	137.072	-1.484	0.000	-1.736	0.000	-0.165	0.761	PA14_31660	529.094	-1.034	0.000
PA14_31680	335.719	0.250	0.454	0.301	0.275	-0.893	0.011	PA14_31680	760.514	0.331	0.192
PA14_31690	482.264	0.203	0.580	0.656	0.016	-1.066	0.003	PA14_31690	534.937	0.140	0.630
PA14_31700	175.876	-0.329	0.485	-0.101	0.816	-0.158	0.784	PA14_31700	461.092	-0.053	0.862
PA14_31720	81.211	0.101	0.867	-0.130	0.774	-0.052	0.937	PA14_31720	199.088	-0.010	0.979
PA14_31730	15.462	-0.167	0.874	-0.496	0.506	-0.641	0.521	PA14_31730	57.770	0.149	0.841
PA14_31740	70.592	-0.253	0.637	0.277	0.510	0.549	0.298	PA14_31740	234.430	-1.057	0.000
PA14_31750	59.962	-0.523	0.318	-0.783	0.070	0.083	0.905	PA14_31750	178.086	-0.516	0.178
PA14_31760	112.147	-0.628	0.113	-0.368	0.312	-0.534	0.274	PA14_31760	232.776	-0.074	0.866
PA14_31770	41.135	0.030	0.971	-0.205	0.702	-0.228	0.760	PA14_31770	99.109	-0.193	0.638
PA14_31780	144.375	0.846	0.019	1.227	0.000	1.388	0.000	PA14_31780	320.826	1.315	0.000
PA14_31800	2409.378	0.652	0.066	0.172	0.640	-0.335	0.467	PA14_31800	2936.289	1.304	0.000
PA14_31810	758.942	1.509	0.000	0.412	0.108	-0.336	0.348	PA14_31810	1006.978	-0.489	0.481
PA14_31820	20.198	0.124	0.910	0.394	0.601	0.728	0.430	PA14_31820	25.458	1.034	0.102
PA14_31840	134.633	-0.995	0.004	-1.851	0.000	-2.072	0.000	PA14_31840	214.481	0.337	0.397

PA14_31850	329.332	-0.367	0.268	-0.681	0.013	-1.540	0.000	PA14_31850	622.687	0.513	0.029
PA14_31870	415.050	-0.128	0.744	0.534	0.050	0.730	0.034	PA14_31870	1115.651	0.399	0.092
PA14_31890	318.543	-0.169	0.652	0.753	0.004	0.012	0.978	PA14_31890	548.041	0.858	0.000
PA14_31900	581.669	0.279	0.424	0.797	0.003	-0.355	0.370	PA14_31900	690.933	0.090	0.765
PA14_31920	331.534	-0.012	0.979	0.548	0.038	-0.887	0.012	PA14_31920	574.500	-0.404	0.089
PA14_31930	20.162	-0.090	0.926	0.728	0.221	-0.155	0.876	PA14_31930	42.585	-0.125	0.834
PA14_31950	31.841	0.418	0.556	0.601	0.280	1.357	0.036	PA14_31950	133.818	0.756	0.019
PA14_31960	45.100	0.104	0.881	-0.055	0.920	0.931	0.094	PA14_31960	224.089	-0.256	0.389
PA14_31970	7.527	0.430	0.749	1.302	0.161	2.605	0.009	PA14_31970	15.511	2.200	0.008
PA14_31990	10.419	0.994	0.304	0.924	0.270	0.437	0.717	PA14_31990	29.309	0.191	0.806
PA14_32015	18.672	-0.314	0.767	-0.136	0.877	1.349	0.124	#N/A	#N/A	#N/A	#N/A
PA14_32025	47.383	0.393	0.498	0.842	0.053	0.859	0.139	#N/A	#N/A	#N/A	#N/A
PA14_32060	48.855	-0.013	0.987	0.218	0.707	0.699	0.301	PA14_32060	110.499	1.329	0.000
PA14_32080	37.300	0.220	0.784	-1.005	0.084	1.151	0.092	PA14_32080	51.524	-0.104	0.814
PA14_32100	26.312	0.781	0.316	-0.354	0.637	1.583	0.040	PA14_32100	28.488	-0.322	0.608
PA14_32110	5.917	0.105	0.945	-0.987	0.337	-0.047	0.974	PA14_32110	16.803	0.385	0.763
PA14_32130	8.110	-0.562	0.617	-1.700	0.055	-0.490	0.686	PA14_32130	22.499	0.469	0.517
PA14_32140	29.282	-1.131	0.142	-2.225	0.001	-2.066	0.026	PA14_32140	42.541	-1.499	0.001
PA14_32150	221.414	-0.434	0.485	-1.601	0.001	-0.257	0.727	PA14_32150	146.843	-1.413	0.000
PA14_32160	243.853	-0.636	0.062	-2.009	0.000	-1.040	0.012	PA14_32160	280.758	-1.113	0.000
PA14_32190	138.651	0.231	0.632	-0.237	0.553	2.126	0.000	PA14_32190	190.029	-0.638	0.027
PA14_32200	68.598	1.140	0.016	1.021	0.019	0.875	0.137	PA14_32200	99.998	1.182	0.001
PA14_32220	183.190	-0.948	0.061	-1.919	0.000	1.059	0.059	PA14_32220	247.736	0.049	0.894
PA14_32230	208.323	-1.452	0.002	-2.392	0.000	-0.941	0.102	PA14_32230	780.327	-0.507	0.032
PA14_32240	95.073	-1.019	0.010	-2.782	0.000	0.021	0.972	PA14_32240	388.386	0.286	0.381
PA14_32250	4.584	-0.851	0.556	-0.746	0.542	1.442	0.248	PA14_32250	7.075	-1.555	0.214
PA14_32270	228.919	0.581	0.094	0.852	0.004	0.799	0.043	PA14_32270	304.646	-0.375	0.181
PA14_32280	224.989	-1.134	0.003	-1.411	0.000	1.573	0.000	PA14_32280	774.904	-2.124	0.000
PA14_32290	107.722	0.530	0.282	0.972	0.015	0.280	0.657	PA14_32290	177.708	1.836	0.000
PA14_32300	44.239	-0.163	0.818	0.236	0.649	0.068	0.931	PA14_32300	78.560	0.914	0.019
PA14_32310	1986.347	-0.666	0.018	-1.436	0.000	-1.205	0.000	PA14_32310	3800.555	-0.543	0.013
PA14_32330	248.513	1.055	0.001	2.265	0.000	-0.263	0.605	PA14_32330	296.074	-0.357	0.378
PA14_32340	50.396	3.565	0.000	4.415	0.000	1.184	0.157	PA14_32340	26.447	-0.403	0.555
PA14_32350	1443.621	4.019	0.000	6.247	0.000	2.270	0.000	PA14_32350	214.993	-0.200	0.499
PA14_32360	67.722	0.241	0.656	0.304	0.475	-0.250	0.697	PA14_32360	162.790	-0.153	0.633
PA14_32370	44.886	0.315	0.633	0.428	0.410	-1.848	0.013	PA14_32370	127.857	1.019	0.002
PA14_32380	46.675	0.274	0.695	1.827	0.000	0.341	0.654	PA14_32380	73.823	0.843	0.033
PA14_32390	159.425	-0.011	0.982	1.441	0.000	0.366	0.414	PA14_32390	198.756	1.267	0.000
PA14_32400	47.670	0.842	0.116	2.207	0.000	0.860	0.176	PA14_32400	105.818	0.418	0.238
PA14_32410	317.921	0.098	0.802	0.549	0.036	-0.929	0.009	PA14_32410	926.768	0.328	0.181
PA14_32420	1148.832	-0.405	0.132	-0.152	0.567	-1.458	0.000	PA14_32420	2984.577	-0.591	0.005
PA14_32440	20.442	-0.537	0.486	-1.068	0.080	-0.829	0.327	PA14_32440	90.307	-1.516	0.000
PA14_32450	61.006	-0.287	0.596	-0.424	0.320	0.034	0.961	PA14_32450	253.310	-0.252	0.374
PA14_32460	44.040	-0.600	0.288	-0.650	0.174	-1.065	0.103	PA14_32460	231.670	0.160	0.612
PA14_32470	36.525	1.141	0.045	1.754	0.000	0.966	0.162	PA14_32470	87.585	0.042	0.912
PA14_32480	177.313	-0.372	0.414	-0.748	0.038	1.649	0.000	PA14_32480	1751.593	-1.510	0.001
PA14_32490	779.162	-0.642	0.053	-0.531	0.082	2.293	0.000	PA14_32490	7537.212	-1.785	0.000
PA14_32500	406.958	0.718	0.027	0.702	0.018	1.010	0.006	PA14_32500	914.459	-0.524	0.021
PA14_32520	293.022	1.416	0.000	2.658	0.000	3.042	0.000	PA14_32520	472.282	0.925	0.000
PA14_32530	1418.398	3.094	0.000	3.439	0.000	2.153	0.000	PA14_32530	2433.370	1.027	0.000
PA14_32540	554.999	3.168	0.000	3.324	0.000	1.868	0.000	PA14_32540	1118.045	0.808	0.000
PA14_32570	25.938	-1.131	0.116	-1.703	0.007	-0.776	0.369	PA14_32570	138.101	0.494	0.296
PA14_32580	64.685	-0.972	0.029	-1.326	0.001	-0.137	0.832	PA14_32580	233.252	-0.526	0.061
PA14_32590	80.110	-0.332	0.506	0.873	0.015	0.430	0.420	PA14_32590	161.124	0.416	0.199
PA14_32600	348.286	-1.489	0.000	-1.119	0.000	-1.068	0.006	PA14_32600	660.695	0.700	0.003
PA14_32610	426.269	-0.526	0.126	0.137	0.691	-0.930	0.016	PA14_32610	479.356	-0.059	0.799
PA14_32630	231.889	-1.044	0.024	0.220	0.644	1.250	0.015	PA14_32630	378.175	-1.797	0.000
PA14_32640	8.959	-1.596	0.090	-2.673	0.002	0.503	0.647	PA14_32640	8.085	1.167	0.342
PA14_32650	3.321	0.458	0.767	-1.177	0.324	0.582	0.682	PA14_32650	3.437	0.060	1.000

PA14_32660	3.527	-1.565	0.213	-2.680	0.015	0.147	0.922
PA14_32670	22.265	0.004	0.997	-1.377	0.025	-0.554	0.528
PA14_32690	24.556	-0.758	0.278	-0.868	0.141	-0.042	0.965
PA14_32700	143.344	1.235	0.001	1.347	0.000	0.001	0.999
PA14_32710	78.298	-0.766	0.128	-0.057	0.913	-0.926	0.125
PA14_32720	22.844	-0.861	0.262	-0.042	0.955	0.296	0.748
PA14_32740	173.002	-1.109	0.003	0.252	0.497	0.186	0.727
PA14_32750	11.394	-0.164	0.895	0.381	0.654	0.388	0.733
PA14_32770	406.933	1.728	0.001	1.957	0.000	1.241	0.044
PA14_32780	137.784	0.108	0.818	-0.435	0.185	-0.595	0.179
PA14_32790	6626.136	-0.345	0.405	-0.734	0.027	-0.503	0.261
PA14_32810	61.673	1.775	0.000	0.436	0.428	2.102	0.000
PA14_32820	475.929	0.413	0.477	-0.516	0.286	0.577	0.351
PA14_32830	1405.401	-0.977	0.005	-1.843	0.000	-0.961	0.021
PA14_32840	207.383	-1.237	0.000	-2.154	0.000	-0.146	0.750
PA14_32850	98.813	-1.152	0.002	-2.416	0.000	-0.257	0.620
PA14_32860	84.289	-0.771	0.155	-2.063	0.000	-1.330	0.029
PA14_32880	80.563	-0.148	0.801	-0.692	0.086	-0.405	0.491
PA14_32890	210.423	0.087	0.885	-0.539	0.183	-1.907	0.000
PA14_32905	71.840	-0.752	0.172	-0.362	0.467	2.902	0.000
PA14_32930	66.581	0.491	0.405	1.212	0.006	1.840	0.001
PA14_32940	497.145	0.687	0.043	1.287	0.000	0.599	0.146
PA14_32950	109.771	-2.336	0.000	-3.910	0.000	-2.180	0.000
PA14_32970	33.099	-0.165	0.830	-0.438	0.422	-1.535	0.041
PA14_32985	593.319	-1.120	0.002	-2.829	0.000	-2.668	0.000
PA14_33000	6400.250	-0.870	0.001	-3.229	0.000	-3.850	0.000
PA14_33010	1301.017	-0.644	0.010	-2.792	0.000	-4.518	0.000
PA14_33030	2963.248	-0.478	0.109	-2.210	0.000	-1.754	0.000
PA14_33040	1281.992	-0.556	0.064	-2.540	0.000	-2.377	0.000
PA14_33050	30.665	-2.628	0.000	-2.651	0.000	0.474	0.505
PA14_33060	21.255	-1.764	0.017	-2.712	0.000	1.858	0.007
PA14_33070	181.278	0.260	0.631	0.878	0.023	2.677	0.000
PA14_33080	82.223	0.062	0.926	0.137	0.773	0.263	0.681
PA14_33110	235.371	0.432	0.439	1.071	0.012	2.306	0.000
PA14_33120	261.792	-0.537	0.111	-0.305	0.330	-0.234	0.599
PA14_33130	505.528	-0.158	0.742	0.520	0.129	1.618	0.000
PA14_33150	149.000	-0.399	0.422	-0.202	0.647	0.253	0.667
PA14_33160	417.984	-2.277	0.000	-2.095	0.000	1.845	0.000
PA14_33170	80.936	0.416	0.366	0.183	0.662	0.244	0.677
PA14_33190	45.246	-1.722	0.001	-1.827	0.000	1.059	0.050
PA14_33200	10.885	-1.048	0.277	-0.995	0.228	0.899	0.362
PA14_33220	49.704	-1.677	0.001	-2.295	0.000	0.436	0.498
PA14_33230	4.384	0.285	0.853	-0.076	0.953	0.985	0.435
PA14_33240	25.973	-0.232	0.772	-0.674	0.252	0.968	0.158
PA14_33250	113.757	-0.475	0.622	0.829	0.198	7.037	0.000
PA14_33260	138.941	-2.383	0.000	-1.625	0.000	1.688	0.000
PA14_33270	56.103	-0.349	0.734	-0.394	0.625	5.601	0.000
PA14_33280	709.306	-0.650	0.144	0.076	0.864	6.075	0.000
PA14_33290	328.485	-1.760	0.000	-2.578	0.000	-0.324	0.386
PA14_33300	827.312	-1.136	0.001	-1.983	0.000	-2.681	0.000
PA14_33310	1743.327	-1.016	0.001	-2.143	0.000	-2.386	0.000
PA14_33320	1855.228	-1.405	0.000	-2.920	0.000	-3.033	0.000
PA14_33330	1628.949	-1.974	0.000	-3.727	0.000	-4.058	0.000
PA14_33340	2259.588	-0.963	0.004	-1.821	0.000	-2.184	0.000
PA14_33350	486.502	-0.589	0.068	-0.780	0.007	-0.850	0.023
PA14_33360	78.684	-0.095	0.875	-0.488	0.232	1.321	0.003
PA14_33370	4.635	-0.596	0.666	-2.962	0.005	-0.410	0.769
PA14_33380	23.820	1.689	0.010	1.618	0.007	2.442	0.000
PA14_33410	41.170	0.889	0.152	0.976	0.070	2.133	0.000

PA14_32660	6.777	1.338	0.321
PA14_32670	72.775	-0.238	0.586
PA14_32690	80.824	-0.760	0.047
PA14_32700	278.921	0.821	0.071
PA14_32710	91.614	0.506	0.174
PA14_32720	21.878	1.366	0.047
PA14_32740	117.607	0.390	0.275
PA14_32750	13.239	0.337	0.763
PA14_32770	502.901	0.361	0.270
PA14_32780	720.819	1.039	0.000
PA14_32790	15274.271	-0.321	0.146
PA14_32810	199.762	-0.815	0.052
PA14_32820	1215.361	-0.416	0.543
PA14_32830	5825.606	-0.106	0.673
PA14_32840	700.680	-1.094	0.000
PA14_32850	387.750	-0.413	0.127
PA14_32860	375.527	-1.007	0.000
PA14_32880	232.465	-0.619	0.025
PA14_32890	390.883	-0.767	0.002
PA14_32905	88.283	-0.486	0.209
PA14_32930	103.558	0.687	0.053
PA14_32940	865.540	1.534	0.000
PA14_32950	313.460	-2.239	0.000
PA14_32970	113.346	0.640	0.064
PA14_32985	1595.928	-0.651	0.004
PA14_33000	7279.122	-0.787	0.000
PA14_33010	2212.726	-1.458	0.000
PA14_33030	2992.530	-0.921	0.000
PA14_33040	984.716	-0.259	0.274
PA14_33050	31.717	-0.394	0.583
PA14_33060	13.894	-1.069	0.314
PA14_33070	99.698	0.042	0.931
PA14_33080	63.628	0.185	0.718
PA14_33110	110.348	0.042	0.966
PA14_33120	514.920	-0.605	0.011
PA14_33130	595.298	-0.014	0.942
PA14_33150	334.325	0.030	0.937
PA14_33160	2750.243	-3.911	0.000
PA14_33170	239.825	-0.299	0.324
PA14_33190	140.298	-1.385	0.000
PA14_33200	85.801	-1.892	0.000
PA14_33220	182.365	-1.617	0.000
PA14_33230	10.574	0.171	0.985
PA14_33240	235.936	-2.757	0.000
PA14_33250	34.897	-2.328	0.000
PA14_33260	76.150	-1.726	0.000
PA14_33270	7.465	-0.331	0.857
PA14_33280	145.815	-0.336	0.325
PA14_33290	1989.768	-1.606	0.000
PA14_33300	2685.819	-1.032	0.000
PA14_33310	5375.885	-0.962	0.000
PA14_33320	5489.594	-0.503	0.022
PA14_33330	4531.977	-0.757	0.000
PA14_33340	4202.308	-1.369	0.000
PA14_33350	1086.137	0.372	0.121
PA14_33360	176.240	0.036	0.906
PA14_33370	19.748	-1.445	0.041
PA14_33380	32.596	1.530	0.006
PA14_33410	40.398	0.311	0.578

PA14_33420	170.741	-0.554	0.147	-1.091	0.001	-0.074	0.892	PA14_33420	299.784	-0.427	0.123
PA14_33430	37.634	-0.152	0.833	-0.511	0.312	0.917	0.121	PA14_33430	167.653	-1.356	0.000
PA14_33440	208.167	0.148	0.729	0.404	0.191	0.378	0.372	PA14_33440	708.712	-0.332	0.155
PA14_33450	97.719	-1.810	0.000	-2.065	0.000	0.294	0.559	PA14_33450	753.772	-2.003	0.000
PA14_33460	19.781	-2.240	0.001	-2.047	0.001	0.239	0.790	PA14_33460	205.131	-1.269	0.020
PA14_33480	106.676	-1.512	0.000	-1.110	0.003	0.786	0.097	PA14_33480	477.003	-0.951	0.000
PA14_33500	78.986	-0.589	0.391	-0.287	0.629	4.162	0.000	PA14_33500	80.784	-1.298	0.000
PA14_33510	51.832	-0.549	0.602	-0.948	0.259	4.749	0.000	PA14_33510	14.713	-2.537	0.003
PA14_33520	35.792	0.341	0.793	-0.065	0.954	5.438	0.000	PA14_33520	9.917	-1.641	0.116
PA14_33530	322.908	-0.921	0.020	-0.096	0.817	4.452	0.000	PA14_33530	137.581	-1.016	0.001
PA14_33540	517.796	-0.248	0.699	1.037	0.017	5.594	0.000	PA14_33540	102.125	-1.601	0.000
PA14_33550	41.006	-0.142	0.904	1.319	0.046	4.937	0.000	PA14_33550	26.464	-2.596	0.002
PA14_33560	97.374	-1.065	0.096	-0.767	0.176	3.546	0.000	PA14_33560	56.253	-2.091	0.000
PA14_33570	55.979	-0.908	0.260	-0.383	0.587	4.540	0.000	PA14_33570	42.280	-3.617	0.000
PA14_33580	60.850	0.122	0.907	0.885	0.161	5.145	0.000	PA14_33580	69.644	-2.556	0.000
PA14_33590	498.249	-1.259	0.002	-0.660	0.081	4.887	0.000	PA14_33590	181.027	-2.491	0.000
PA14_33600	831.518	-1.035	0.013	-0.202	0.632	5.683	0.000	PA14_33600	383.089	-2.928	0.000
PA14_33610	2897.973	-0.196	0.622	0.620	0.034	5.403	0.000	PA14_33610	1493.450	0.018	0.981
PA14_33630	2285.128	-0.513	0.098	-0.219	0.465	5.005	0.000	PA14_33630	1622.392	-0.107	0.648
PA14_33650	2559.880	-0.087	0.814	0.392	0.121	4.947	0.000	PA14_33650	1945.622	-0.147	0.516
PA14_33680	1192.392	-0.582	0.124	-0.318	0.376	2.486	0.000	PA14_33680	582.703	-1.390	0.000
PA14_33690	1135.813	0.375	0.422	0.981	0.006	6.328	0.000	PA14_33690	175.309	-0.001	1.000
PA14_33700	235.474	-0.344	0.490	0.206	0.629	2.857	0.000	PA14_33700	475.462	0.138	0.612
PA14_33710	42.668	0.484	0.678	0.656	0.466	5.660	0.000	PA14_33710	14.610	0.357	0.715
PA14_33720	188.898	-0.089	0.916	0.234	0.685	5.551	0.000	PA14_33720	20.697	-0.455	0.581
PA14_33730	445.328	-0.764	0.196	-0.571	0.273	5.883	0.000	PA14_33730	53.750	-0.578	0.201
PA14_33740	116.179	-0.133	0.848	0.457	0.322	3.861	0.000	PA14_33740	109.665	0.221	0.553
PA14_33750	35.313	0.386	0.634	0.946	0.109	2.283	0.001	PA14_33750	25.487	2.599	0.000
PA14_33760	206.270	0.711	0.057	1.955	0.000	2.927	0.000	PA14_33760	148.311	2.379	0.000
PA14_33770	248.195	0.647	0.067	1.463	0.000	3.267	0.000	PA14_33770	229.161	1.442	0.000
PA14_33780	1159.721	0.249	0.462	0.209	0.475	0.582	0.092	PA14_33780	2077.975	-0.881	0.000
PA14_33800	494.501	0.328	0.412	0.556	0.084	-0.002	0.998	PA14_33800	678.382	-0.503	0.032
PA14_33810	701.676	0.268	0.622	1.505	0.000	5.794	0.000	PA14_33810	142.848	-0.512	0.407
PA14_33820	239.711	-0.741	0.170	0.855	0.040	5.246	0.000	PA14_33820	48.149	0.317	0.570
PA14_33830	23.005	-0.866	0.348	-1.324	0.085	2.634	0.001	PA14_33830	22.538	-1.912	0.004
PA14_33840	95.572	0.423	0.366	0.832	0.023	1.595	0.000	PA14_33840	164.505	-0.025	0.936
PA14_33860	529.505	1.735	0.000	2.200	0.000	1.473	0.001	PA14_33860	570.731	-1.297	0.000
PA14_33870	563.031	-3.362	0.000	-4.594	0.000	-0.988	0.033	PA14_33870	1082.762	-3.326	0.000
PA14_33880	136.348	-0.012	0.984	0.896	0.010	1.829	0.000	PA14_33880	201.568	-0.040	0.868
PA14_33890	116.683	-0.777	0.075	-0.980	0.011	-0.588	0.274	PA14_33890	465.872	-0.121	0.716
PA14_33900	317.784	-0.467	0.196	-0.544	0.084	-0.892	0.026	PA14_33900	642.960	-0.637	0.007
PA14_33910	22.704	0.521	0.541	0.205	0.790	2.068	0.005	PA14_33910	52.966	-1.240	0.005
PA14_33920	5.942	-0.024	0.987	0.363	0.742	2.143	0.042	PA14_33920	12.771	-0.136	0.926
PA14_33930	19.181	-1.496	0.054	-1.846	0.008	0.630	0.485	PA14_33930	94.984	-2.888	0.000
PA14_33940	21.928	-0.367	0.653	0.160	0.814	0.182	0.847	PA14_33940	125.682	-1.471	0.001
PA14_33960	93.700	-1.558	0.000	-1.795	0.000	0.183	0.738	PA14_33960	339.587	-1.891	0.000
PA14_33970	91.283	-2.124	0.000	-2.044	0.000	-0.138	0.822	PA14_33970	320.532	-2.411	0.000
PA14_33980	38.106	-3.065	0.000	-3.238	0.000	-1.392	0.023	PA14_33980	267.476	-3.457	0.000
PA14_33990	108.753	-2.792	0.000	-3.391	0.000	-0.874	0.075	PA14_33990	526.420	-3.987	0.000
PA14_34000	25.978	-3.466	0.000	-3.652	0.000	-1.303	0.066	PA14_34000	112.814	-4.676	0.000
PA14_34010	68.076	-2.666	0.000	-3.385	0.000	-0.914	0.089	PA14_34010	218.229	-3.611	0.000
PA14_34020	3.299	-3.353	0.004	-3.458	0.002	-1.129	0.381	PA14_34020	14.237	-4.479	0.000
PA14_34030	245.835	-2.894	0.000	-4.265	0.000	-1.063	0.009	PA14_34030	545.550	-3.616	0.000
PA14_34050	437.480	-3.008	0.000	-4.113	0.000	-0.402	0.295	PA14_34050	599.422	-2.811	0.000
PA14_34070	217.036	-2.322	0.007	-3.039	0.000	0.580	0.605	PA14_34070	426.423	-2.931	0.000
PA14_34080	216.089	-1.047	0.001	-1.055	0.000	1.116	0.002	PA14_34080	550.220	-0.891	0.000
PA14_34100	186.024	-1.533	0.000	-1.882	0.000	0.435	0.291	PA14_34100	626.338	-0.264	0.292
PA14_34110	26.236	-1.792	0.004	-2.581	0.000	-0.751	0.335	PA14_34110	224.430	-0.806	0.076
PA14_34130	177.395	-1.948	0.000	-2.839	0.000	-0.833	0.043	PA14_34130	479.947	-1.468	0.000

PA14_34140	68.514	-2.654	0.000	-3.835	0.000	-1.186	0.016	PA14_34140	467.307	-3.084	0.000
PA14_34150	145.038	-1.472	0.000	-1.208	0.000	-0.820	0.073	PA14_34150	100.206	-0.670	0.054
PA14_34170	26.338	-2.828	0.000	-1.321	0.025	1.947	0.002	PA14_34170	183.423	0.167	0.969
PA14_34180	9.197	-1.051	0.282	-0.132	0.888	0.369	0.744	PA14_34180	17.639	0.800	0.718
PA14_34190	5.066	-1.383	0.234	-0.825	0.422	0.013	0.992	PA14_34190	17.968	1.848	0.268
PA14_34200	11.255	-0.283	0.799	-0.369	0.663	0.739	0.456	PA14_34200	27.627	0.112	1.000
PA14_34210	8.623	-0.255	0.833	-0.639	0.468	-0.215	0.863	PA14_34210	23.467	0.639	0.359
PA14_34230	159.063	0.171	0.749	0.680	0.068	-0.647	0.220	PA14_34230	175.603	0.804	0.007
PA14_34250	96.787	0.806	0.072	0.965	0.015	-0.959	0.103	PA14_34250	79.728	1.744	0.000
PA14_34260	12.415	0.015	0.989	-0.050	0.956	1.289	0.143	PA14_34260	19.933	-0.096	0.935
PA14_34270	6.076	0.437	0.740	-0.449	0.681	0.942	0.427	PA14_34270	11.003	0.285	0.866
PA14_34280	16.081	-0.532	0.564	0.135	0.866	1.282	0.114	PA14_34280	20.785	-0.200	0.844
PA14_34290	16.040	-0.534	0.548	-0.712	0.318	1.137	0.160	PA14_34290	19.791	0.800	0.275
PA14_34300	7.322	0.084	0.952	0.120	0.909	0.419	0.734	PA14_34300	11.923	0.620	0.535
PA14_34320	19.638	-0.109	0.916	0.061	0.937	0.692	0.419	PA14_34320	23.378	1.162	0.071
PA14_34330	160.309	-1.744	0.000	-1.973	0.000	-1.304	0.002	PA14_34330	393.771	-1.764	0.000
PA14_34340	32.747	-0.496	0.452	-0.048	0.939	-1.362	0.061	PA14_34340	60.610	2.490	0.000
PA14_34350	13.770	-0.553	0.524	-3.215	0.000	-0.687	0.471	PA14_34350	5.564	0.907	0.548
PA14_34360	17.533	-0.609	0.432	-3.019	0.000	-0.337	0.716	PA14_34360	15.109	1.023	0.236
PA14_34370	22.001	-1.392	0.025	-3.245	0.000	-1.874	0.014	PA14_34370	20.535	0.794	0.284
PA14_34390	26.082	-0.638	0.345	-2.440	0.000	0.448	0.555	PA14_34390	34.292	-0.689	0.459
PA14_34410	30.374	-0.719	0.289	-1.627	0.004	0.061	0.945	PA14_34410	21.776	-0.213	0.798
PA14_34420	132.799	-1.080	0.005	-1.428	0.000	0.265	0.621	PA14_34420	50.014	0.252	0.630
PA14_34440	58.467	-0.123	0.845	-0.827	0.052	-1.398	0.020	PA14_34440	116.410	-0.652	0.057
PA14_34450	34.638	-0.415	0.520	-0.957	0.057	-1.479	0.038	PA14_34450	81.831	-0.110	0.799
PA14_34460	106.636	-1.405	0.000	-3.449	0.000	-1.959	0.000	PA14_34460	161.993	-1.640	0.000
PA14_34490	49.228	-1.230	0.024	-2.503	0.000	-2.169	0.001	PA14_34490	95.631	-1.084	0.002
PA14_34500	86.992	-0.543	0.358	-2.797	0.000	-0.745	0.251	PA14_34500	166.957	0.204	0.512
PA14_34510	46.555	-0.573	0.268	-1.944	0.000	-0.844	0.156	PA14_34510	112.153	-0.307	0.403
PA14_34520	10.939	-1.070	0.273	-0.831	0.325	-0.309	0.795	PA14_34520	28.885	-0.443	0.512
PA14_34540	75.159	-0.079	0.899	-0.782	0.049	0.109	0.864	PA14_34540	223.526	-1.745	0.000
PA14_34550	19.131	0.706	0.426	2.499	0.000	2.055	0.009	PA14_34550	78.983	2.615	0.000
PA14_34580	70.484	1.644	0.002	4.006	0.000	3.123	0.000	PA14_34580	123.189	2.136	0.000
PA14_34600	1348.880	0.115	0.807	0.153	0.676	-0.270	0.563	PA14_34600	5194.818	1.613	0.000
PA14_34610	90.585	3.253	0.000	4.007	0.000	4.848	0.000	PA14_34610	149.058	3.801	0.000
PA14_34630	221.330	3.287	0.000	4.157	0.000	4.479	0.000	PA14_34630	441.493	4.445	0.000
PA14_34640	463.578	3.895	0.000	4.722	0.000	6.080	0.000	PA14_34640	617.553	4.667	0.000
PA14_34660	393.407	1.592	0.000	2.054	0.000	1.625	0.000	PA14_34660	1069.655	1.891	0.000
PA14_34670	21.973	-0.877	0.289	-1.717	0.013	-1.447	0.110	PA14_34670	122.722	-0.921	0.005
PA14_34680	246.665	0.161	0.822	0.872	0.070	0.712	0.264	PA14_34680	359.892	0.094	0.726
PA14_34690	62.653	0.358	0.496	0.430	0.314	-0.409	0.522	PA14_34690	142.329	1.970	0.000
PA14_34700	6.068	-0.526	0.695	0.514	0.615	0.525	0.684	PA14_34700	17.967	2.630	0.001
PA14_34710	5.919	-1.287	0.277	0.455	0.656	1.301	0.239	PA14_34710	13.225	1.563	0.080
PA14_34720	39.229	-0.951	0.066	-1.146	0.012	-0.525	0.428	PA14_34720	139.293	-0.906	0.004
PA14_34730	16.902	-4.284	0.000	-2.510	0.000	-2.726	0.003	PA14_34730	8.789	0.525	0.704
PA14_34740	17.320	-3.181	0.000	-2.624	0.001	-1.368	0.167	PA14_34740	7.944	-0.962	0.422
PA14_34750	11.737	-2.431	0.005	-1.199	0.111	0.095	0.933	PA14_34750	16.530	-0.120	0.909
PA14_34770	13.151	-2.328	0.004	-1.541	0.030	0.217	0.837	PA14_34770	12.741	0.001	1.000
PA14_34780	9.230	-0.857	0.406	-0.928	0.280	0.493	0.660	PA14_34780	13.240	0.861	0.463
PA14_34790	17.205	-0.748	0.405	-1.861	0.011	-0.550	0.589	PA14_34790	38.307	-1.096	0.029
PA14_34800	396.605	-0.176	0.611	-1.078	0.000	-1.493	0.000	PA14_34800	2613.209	-0.952	0.001
PA14_34810	1271.280	-0.452	0.184	-1.096	0.000	-1.815	0.000	PA14_34810	2928.871	-0.291	0.178
PA14_34820	132.575	-1.613	0.000	-3.488	0.000	-2.775	0.000	PA14_34820	700.027	-2.310	0.000
PA14_34830	186.154	-1.421	0.000	-3.038	0.000	-1.692	0.000	PA14_34830	880.661	-2.373	0.000
PA14_34840	635.651	-1.725	0.000	-2.661	0.000	-0.699	0.059	PA14_34840	3191.121	-2.590	0.000
PA14_34850	57.617	-0.571	0.261	-1.070	0.011	0.761	0.151	PA14_34850	273.825	-1.952	0.000
PA14_34870	1263.517	-3.922	0.000	-4.623	0.000	1.776	0.000	PA14_34870	1554.410	-2.861	0.000
PA14_34880	652.493	1.130	0.001	2.180	0.000	0.098	0.859	PA14_34880	564.356	0.659	0.006
PA14_34900	198.849	0.278	0.478	0.675	0.023	-0.517	0.239	PA14_34900	804.074	1.046	0.000

PA14_34920	23.663	-0.530	0.488	0.600	0.305	0.022	0.981	PA14_34920	73.840	1.846	0.000
PA14_34930	22.605	0.429	0.598	1.185	0.043	-0.072	0.944	PA14_34930	66.788	1.677	0.000
PA14_34940	4.182	-0.196	0.910	2.079	0.044	1.511	0.222	PA14_34940	11.759	1.162	0.235
PA14_34960	375.898	0.903	0.016	1.544	0.000	0.685	0.139	PA14_34960	1288.804	0.143	0.570
PA14_34970	2008.558	0.543	0.061	1.205	0.000	0.605	0.075	PA14_34970	1389.363	0.382	0.093
PA14_34990	991.113	1.001	0.000	1.853	0.000	0.920	0.004	PA14_34990	803.512	1.240	0.000
PA14_35000	92.002	0.744	0.085	1.374	0.000	0.192	0.760	PA14_35000	184.080	-0.619	0.035
PA14_35010	12.086	0.797	0.393	0.261	0.768	1.393	0.128	PA14_35010	21.026	0.218	0.801
PA14_35020	12.415	0.482	0.686	1.527	0.068	0.819	0.471	PA14_35020	16.245	1.423	0.070
PA14_35030	9.122	-1.399	0.173	-1.274	0.159	-1.804	0.100	PA14_35030	26.609	0.546	0.390
PA14_35040	7.522	0.782	0.483	1.023	0.252	1.007	0.369	PA14_35040	18.931	1.258	0.085
PA14_35050	9.792	-0.034	0.979	0.854	0.288	-0.055	0.966	PA14_35050	24.047	1.640	0.011
PA14_35060	49.683	0.126	0.865	0.788	0.092	0.677	0.290	PA14_35060	128.934	-1.464	0.000
PA14_35070	164.626	0.367	0.454	0.834	0.028	0.912	0.061	PA14_35070	316.305	1.268	0.000
PA14_35080	48.264	0.148	0.823	-0.113	0.829	-1.337	0.039	PA14_35080	69.400	0.036	0.922
PA14_35100	43.995	0.792	0.213	0.882	0.108	-2.288	0.007	PA14_35100	57.752	0.446	0.333
PA14_35110	23.670	0.161	0.852	0.870	0.118	-0.608	0.493	PA14_35110	38.926	0.849	0.113
PA14_35130	11.201	0.833	0.374	0.881	0.263	-1.172	0.293	PA14_35130	33.011	-0.464	0.449
PA14_35140	113.340	-0.433	0.302	-0.510	0.148	0.279	0.580	PA14_35140	276.300	0.511	0.062
PA14_35150	13.374	0.084	0.944	0.273	0.741	1.140	0.202	PA14_35150	24.583	-0.320	0.695
PA14_35160	23.499	-1.495	0.025	-0.536	0.375	1.293	0.052	PA14_35160	321.397	-2.633	0.000
PA14_35170	31.871	-0.970	0.091	-0.494	0.345	-1.397	0.047	PA14_35170	124.738	-1.019	0.006
PA14_35190	379.316	0.724	0.012	0.486	0.079	-0.369	0.349	PA14_35190	523.585	1.491	0.000
PA14_35200	32.840	0.986	0.114	1.957	0.000	-0.358	0.706	PA14_35200	71.036	2.000	0.000
PA14_35210	174.907	0.341	0.423	0.913	0.005	-0.306	0.551	PA14_35210	352.323	1.845	0.000
PA14_35230	144.639	0.781	0.094	2.479	0.000	-0.199	0.774	PA14_35230	229.963	1.833	0.000
PA14_35240	16.995	-0.067	0.949	0.701	0.278	0.473	0.607	PA14_35240	27.528	0.093	0.923
PA14_35250	87.644	0.465	0.303	0.850	0.019	-1.525	0.009	PA14_35250	224.476	1.520	0.000
PA14_35270	259.786	2.763	0.000	2.340	0.000	0.958	0.029	PA14_35270	368.477	-0.028	0.977
PA14_35290	1187.977	3.015	0.000	2.273	0.000	1.097	0.005	PA14_35290	1246.799	-0.701	0.008
PA14_35300	631.401	2.935	0.000	2.325	0.000	1.168	0.017	PA14_35300	664.524	-0.912	0.000
PA14_35320	147.538	4.603	0.000	3.032	0.000	1.943	0.000	PA14_35320	64.548	0.712	0.086
PA14_35330	136.493	4.539	0.000	3.558	0.000	1.713	0.002	PA14_35330	81.304	0.185	0.630
PA14_35340	145.863	5.378	0.000	4.548	0.000	3.430	0.000	PA14_35340	110.286	-0.059	0.898
PA14_35360	80.071	5.536	0.000	4.747	0.000	3.149	0.000	PA14_35360	40.210	0.368	0.500
PA14_35370	219.550	3.463	0.000	3.201	0.000	2.911	0.000	PA14_35370	304.130	0.605	0.041
PA14_35380	32.742	0.339	0.650	1.030	0.049	0.954	0.173	PA14_35380	135.689	1.887	0.000
PA14_35390	4.292	-0.649	0.628	-0.533	0.631	-0.217	0.880	PA14_35390	12.353	0.299	0.792
PA14_35400	6.554	0.490	0.693	0.389	0.702	0.768	0.517	PA14_35400	20.510	0.543	0.465
PA14_35420	3.986	0.462	0.765	1.217	0.274	1.684	0.167	PA14_35420	4.454	1.135	0.521
PA14_35430	22.440	-0.148	0.875	-0.159	0.824	0.695	0.386	PA14_35430	26.601	0.108	0.906
PA14_35440	38.610	-0.707	0.320	-1.665	0.004	-2.141	0.006	PA14_35440	53.364	1.467	0.001
PA14_35460	97.304	0.181	0.699	-1.748	0.000	-0.451	0.353	PA14_35460	53.580	0.568	0.282
PA14_35470	358.440	0.646	0.095	0.922	0.006	-1.510	0.001	PA14_35470	515.626	-0.118	0.674
PA14_35490	1669.800	-6.282	0.000	-4.225	0.000	-1.736	0.000	PA14_35490	7961.645	0.863	0.006
PA14_35500	2398.331	-5.748	0.000	-4.275	0.000	-0.952	0.004	PA14_35500	14179.032	0.941	0.000
PA14_35520	1631.513	-5.344	0.000	-4.180	0.000	-1.447	0.000	PA14_35520	12708.892	0.237	0.301
PA14_35530	3439.553	-4.698	0.000	-3.996	0.000	-0.683	0.021	PA14_35530	16081.365	-0.187	0.449
PA14_35540	523.533	0.293	0.509	1.038	0.002	-1.283	0.003	PA14_35540	1055.546	0.798	0.001
PA14_35550	4.490	-1.322	0.295	-1.263	0.249	0.774	0.548	PA14_35550	13.909	-0.956	0.303
PA14_35570	61.539	-2.169	0.000	-1.595	0.000	1.869	0.000	PA14_35570	221.137	-2.019	0.000
PA14_35590	4.978	-0.286	0.849	0.212	0.856	1.051	0.385	PA14_35590	13.389	1.025	0.252
PA14_35600	22.055	-1.237	0.088	-1.186	0.063	0.619	0.457	PA14_35600	58.472	0.184	0.813
PA14_35620	23.974	-0.996	0.151	-1.822	0.003	1.855	0.003	PA14_35620	33.696	-0.385	0.642
PA14_35630	18.005	-2.186	0.004	-1.022	0.119	1.482	0.040	PA14_35630	31.940	-0.060	0.958
PA14_35640	16.027	-1.050	0.192	-1.321	0.058	1.590	0.034	PA14_35640	39.595	-0.773	0.128
PA14_35650	22.950	-1.492	0.055	-2.368	0.001	1.085	0.192	PA14_35650	42.174	-0.126	0.861
PA14_35670	58.001	-1.071	0.034	-2.534	0.000	1.864	0.000	PA14_35670	183.679	-1.395	0.000
PA14_35680	34.882	-1.005	0.100	-0.998	0.062	2.005	0.000	PA14_35680	125.030	-1.184	0.001

PA14_35690	91.858	-0.377	0.441	-0.438	0.274	2.055	0.000	PA14_35690	171.883	0.486	0.136
PA14_35700	291.240	-0.477	0.152	-0.665	0.021	-0.023	0.963	PA14_35700	1367.889	-1.853	0.000
PA14_35710	3730.578	0.664	0.082	0.904	0.008	0.213	0.684	PA14_35710	6287.715	0.240	0.526
PA14_35720	2412.398	0.532	0.075	-0.019	0.955	-0.806	0.018	PA14_35720	4324.086	-0.585	0.006
PA14_35730	171.518	0.200	0.635	0.627	0.039	1.833	0.000	PA14_35730	402.226	0.503	0.106
PA14_35740	1612.098	-1.070	0.000	-1.527	0.000	-0.691	0.032	PA14_35740	5184.478	-0.438	0.050
PA14_35750	38.919	-2.414	0.000	-3.635	0.000	-2.307	0.000	PA14_35750	247.610	-1.474	0.000
PA14_35760	1872.434	-0.435	0.237	-1.876	0.000	-1.186	0.002	PA14_35760	5803.828	0.168	0.610
PA14_35770	1936.441	-0.255	0.422	-0.801	0.001	-0.082	0.841	PA14_35770	5125.216	0.462	0.030
PA14_35780	1048.406	-0.789	0.013	-2.164	0.000	-1.500	0.000	PA14_35780	4079.906	0.746	0.012
PA14_35790	7151.455	-0.562	0.066	-1.311	0.000	-0.849	0.014	PA14_35790	18861.663	0.111	0.742
PA14_35800	4681.311	-0.469	0.077	-1.083	0.000	-0.707	0.018	PA14_35800	16514.919	0.664	0.001
PA14_35810	5230.258	-0.966	0.000	-1.439	0.000	-0.155	0.696	PA14_35810	17418.710	0.146	0.521
PA14_35820	4902.526	-0.444	0.152	-0.364	0.202	0.188	0.647	PA14_35820	13060.140	-1.163	0.000
PA14_35830	5569.421	0.866	0.000	1.021	0.000	-1.265	0.000	PA14_35830	9421.170	0.953	0.000
PA14_35840	2096.694	-0.092	0.833	-0.393	0.191	-0.958	0.008	PA14_35840	9468.915	-0.661	0.001
PA14_35850	2745.222	-0.227	0.486	-0.262	0.337	0.493	0.140	PA14_35850	9627.712	-1.131	0.000
PA14_35860	404.411	0.479	0.288	1.096	0.002	2.713	0.000	PA14_35860	1040.407	0.321	0.397
PA14_35880	370.267	-0.044	0.931	0.027	0.946	2.442	0.000	PA14_35880	1087.969	0.483	0.033
PA14_35890	109.588	-0.476	0.286	-0.547	0.146	0.824	0.072	PA14_35890	470.311	0.662	0.047
PA14_35900	45.529	-0.265	0.692	-0.366	0.476	1.725	0.001	PA14_35900	130.618	0.417	0.209
PA14_35920	59.180	0.208	0.750	0.658	0.151	0.939	0.105	PA14_35920	128.570	-0.185	0.605
PA14_35930	1.236	-0.453	0.788	-0.283	0.841	-1.087	0.375	PA14_35930	6.917	1.637	0.498
PA14_35940	26.003	0.046	0.962	0.449	0.440	0.822	0.253	PA14_35940	48.551	0.324	0.692
PA14_35950	7.941	-0.601	0.600	-0.417	0.662	-0.542	0.657	PA14_35950	13.295	0.250	0.839
PA14_35970	25.718	-0.288	0.717	-0.292	0.643	1.158	0.087	PA14_35970	43.894	0.466	0.521
PA14_35980	9.746	0.378	0.750	-0.236	0.815	1.766	0.066	PA14_35980	16.796	0.214	0.825
PA14_35990	13.699	-0.387	0.717	-0.321	0.708	0.676	0.501	PA14_35990	32.941	0.153	0.803
PA14_36000	230.873	0.303	0.393	0.470	0.099	-0.937	0.018	PA14_36000	599.254	1.083	0.000
PA14_36010	660.825	-0.273	0.478	1.343	0.000	-0.720	0.067	PA14_36010	1166.348	0.614	0.006
PA14_36020	556.202	0.345	0.458	2.640	0.000	1.772	0.000	PA14_36020	1426.110	0.712	0.001
PA14_36030	61.189	-0.204	0.789	1.973	0.000	2.206	0.000	PA14_36030	303.298	0.384	0.177
PA14_36050	23.623	0.028	0.977	-0.586	0.357	0.103	0.914	PA14_36050	35.052	0.840	0.118
PA14_36060	21.751	0.189	0.863	0.546	0.474	0.653	0.504	PA14_36060	59.051	-0.048	0.922
PA14_36070	118.004	0.355	0.412	0.282	0.449	0.365	0.464	PA14_36070	189.187	0.031	0.933
PA14_36080	5.506	-0.174	0.914	-0.179	0.883	0.316	0.825	PA14_36080	9.008	0.390	0.822
PA14_36090	17.352	-0.573	0.519	-0.241	0.753	1.248	0.118	PA14_36090	30.181	0.285	0.684
PA14_36100	22.456	0.109	0.906	-0.741	0.235	-0.483	0.591	PA14_36100	54.679	1.330	0.003
PA14_36110	15.280	0.452	0.657	0.172	0.847	1.050	0.268	PA14_36110	36.925	1.321	0.011
PA14_36120	22.945	-0.628	0.411	-0.712	0.259	0.005	0.996	PA14_36120	41.122	1.634	0.001
PA14_36130	53.434	0.251	0.717	0.127	0.828	0.896	0.155	PA14_36130	76.429	0.033	0.960
PA14_36150	2.148	-0.871	0.563	-1.493	0.218	0.392	0.793	PA14_36150	4.868	0.609	0.783
PA14_36170	16.396	-0.559	0.516	-0.974	0.156	1.096	0.167	PA14_36170	18.367	1.525	0.037
PA14_36180	50.380	0.457	0.465	0.052	0.932	1.078	0.078	PA14_36180	116.587	0.164	0.672
PA14_36190	18.782	-0.200	0.855	0.079	0.929	0.018	0.988	PA14_36190	31.076	0.512	0.613
PA14_36200	1103.275	-4.608	0.000	-5.163	0.000	-2.082	0.000	PA14_36200	259.516	0.778	0.004
PA14_36220	21.522	-3.462	0.000	-3.992	0.000	-1.005	0.203	PA14_36220	9.887	0.307	0.817
PA14_36230	31.591	-2.519	0.000	-1.633	0.002	-0.426	0.587	PA14_36230	15.205	1.539	0.065
PA14_36250	128.321	0.677	0.069	0.846	0.010	-0.495	0.331	PA14_36250	181.149	1.337	0.000
PA14_36260	106.729	1.061	0.008	2.304	0.000	0.551	0.313	PA14_36260	182.758	1.810	0.000
PA14_36270	1473.904	0.715	0.035	2.060	0.000	-0.319	0.483	PA14_36270	1851.786	2.282	0.000
PA14_36280	1665.282	1.778	0.031	3.687	0.000	0.994	0.315	PA14_36280	828.231	2.812	0.000
PA14_36290	219.962	1.098	0.000	2.378	0.000	-0.507	0.272	PA14_36290	444.378	2.579	0.000
PA14_36300	375.877	0.092	0.860	1.302	0.000	-0.804	0.077	PA14_36300	1058.304	0.837	0.000
PA14_36310	1679.341	-2.120	0.000	-5.553	0.000	-3.285	0.000	PA14_36310	1700.175	-0.927	0.000
PA14_36320	1817.179	-2.252	0.000	-5.572	0.000	-3.193	0.000	PA14_36320	1761.552	-0.165	0.475
PA14_36330	205.259	-1.586	0.000	-4.735	0.000	-3.196	0.000	PA14_36330	414.360	0.381	0.178
PA14_36345	678.075	0.580	0.115	0.082	0.835	1.410	0.000	PA14_36345	1626.248	-1.094	0.000
PA14_36350	30.291	-0.880	0.152	-1.136	0.032	0.916	0.156	PA14_36350	123.206	-1.963	0.000

PA14_36360	30.600	-1.309	0.049	-1.661	0.005	2.123	0.001	PA14_36360	56.280	-1.205	0.005
PA14_36370	11.056	-0.968	0.331	-1.783	0.035	1.559	0.084	PA14_36370	32.834	-0.896	0.103
PA14_36375	37.764	-1.670	0.003	-2.074	0.000	1.021	0.093	PA14_36375	70.457	-1.495	0.000
PA14_36390	15.509	-2.027	0.006	-1.904	0.004	-0.574	0.535	PA14_36390	37.644	-0.213	0.733
PA14_36400	6.451	-0.437	0.754	0.112	0.925	2.416	0.017	PA14_36400	18.425	0.108	0.970
PA14_36410	3.303	-1.135	0.421	-0.518	0.682	1.406	0.264	PA14_36410	29.093	-1.933	0.001
PA14_36420	399.882	-1.152	0.000	-1.191	0.000	1.090	0.002	PA14_36420	1542.480	-1.743	0.000
PA14_36450	232.789	-2.146	0.000	-2.188	0.000	1.003	0.005	PA14_36450	1937.220	-2.647	0.000
PA14_36460	237.048	-2.540	0.000	-2.071	0.000	1.387	0.001	PA14_36460	2083.213	-2.901	0.000
PA14_36470	221.522	-2.489	0.000	-3.219	0.000	0.535	0.178	PA14_36470	1899.423	-3.463	0.000
PA14_36480	18.963	-0.522	0.617	-0.417	0.625	3.082	0.000	PA14_36480	28.492	-1.158	0.051
PA14_36490	23.209	-0.538	0.545	-0.844	0.239	3.114	0.000	PA14_36490	41.436	-2.027	0.000
PA14_36500	70.002	-0.276	0.679	-0.035	0.952	3.582	0.000	PA14_36500	44.396	-1.445	0.002
PA14_36520	150.537	-1.023	0.028	-1.062	0.011	2.318	0.000	PA14_36520	241.851	-1.997	0.000
PA14_36530	166.194	-1.623	0.000	-1.323	0.001	3.043	0.000	PA14_36530	119.031	-1.746	0.000
PA14_36540	30.615	-0.148	0.885	-0.460	0.532	3.133	0.000	PA14_36540	53.953	-0.988	0.025
PA14_36550	106.288	-0.732	0.085	-1.212	0.001	2.743	0.000	PA14_36550	163.361	-1.287	0.000
PA14_36560	239.201	-0.900	0.009	-1.902	0.000	1.785	0.000	PA14_36560	244.509	-2.723	0.000
PA14_36570	197.669	-1.753	0.000	-2.204	0.000	2.850	0.000	PA14_36570	132.130	-2.194	0.000
PA14_36580	288.266	-1.557	0.001	-0.991	0.028	3.183	0.000	PA14_36580	76.613	-1.799	0.000
PA14_36590	36.645	-2.261	0.000	-2.558	0.000	2.204	0.000	PA14_36590	23.799	-1.555	0.014
PA14_36605	117.614	-1.858	0.000	-2.029	0.000	2.529	0.000	PA14_36605	107.375	-2.727	0.000
PA14_36620	52.136	-2.613	0.000	-2.481	0.000	2.000	0.001	PA14_36620	71.057	-3.729	0.000
PA14_36630	224.151	-1.653	0.000	-1.458	0.000	2.930	0.000	PA14_36630	258.449	-2.507	0.000
PA14_36650	67.466	-1.586	0.004	-1.692	0.001	1.560	0.006	PA14_36650	200.185	-2.797	0.000
PA14_36660	75.905	-1.019	0.037	-0.136	0.777	2.769	0.000	PA14_36660	86.178	-0.966	0.009
PA14_36670	9.409	-1.161	0.268	-0.813	0.372	1.003	0.339	PA14_36670	24.519	-1.156	0.067
PA14_36680	6.184	-1.096	0.357	-0.530	0.611	1.427	0.186	PA14_36680	10.668	0.030	1.000
PA14_36690	8.719	0.318	0.808	-0.621	0.545	2.441	0.010	PA14_36690	17.442	-0.349	0.724
PA14_36700	12.321	-1.300	0.190	-0.076	0.937	2.100	0.015	PA14_36700	9.403	0.321	0.798
PA14_36710	42.509	-1.207	0.038	-1.089	0.035	1.673	0.003	PA14_36710	94.248	-2.100	0.000
PA14_36730	173.558	-1.486	0.000	-1.557	0.000	1.760	0.000	PA14_36730	287.795	-1.916	0.000
PA14_36740	264.229	-1.818	0.000	-1.574	0.000	2.508	0.000	PA14_36740	216.832	-1.607	0.000
PA14_36760	98.874	-0.505	0.443	-0.176	0.766	2.831	0.000	PA14_36760	180.494	-1.820	0.000
PA14_36770	15.981	-0.831	0.389	-1.102	0.166	1.738	0.041	PA14_36770	117.294	-2.204	0.000
PA14_36780	19.752	-2.127	0.008	-1.089	0.115	2.051	0.004	PA14_36780	147.357	-2.808	0.000
PA14_36790	10.620	-2.066	0.027	-2.024	0.016	0.356	0.752	PA14_36790	40.867	-2.319	0.012
PA14_36810	211.536	-1.652	0.000	-1.161	0.003	1.673	0.000	PA14_36810	128.518	-1.317	0.000
PA14_36820	117.104	-1.863	0.000	-1.637	0.000	1.330	0.005	PA14_36820	113.558	-2.479	0.000
PA14_36830	19.302	0.725	0.360	1.110	0.078	0.436	0.657	PA14_36830	58.518	-0.301	0.501
PA14_36840	202.994	-2.048	0.000	-2.241	0.000	2.026	0.000	PA14_36840	190.240	-1.818	0.000
PA14_36850	189.185	-2.568	0.000	-3.074	0.000	2.010	0.000	PA14_36850	245.123	-2.948	0.000
PA14_36860	35.596	-2.652	0.000	-4.822	0.000	1.182	0.047	PA14_36860	497.845	-5.626	0.000
PA14_36870	30.200	-0.052	0.956	-0.526	0.391	2.099	0.001	PA14_36870	57.924	-0.383	0.427
PA14_36880	14.187	-0.825	0.437	-0.384	0.676	2.813	0.001	PA14_36880	27.149	-0.487	0.464
PA14_36890	4.178	-1.211	0.358	-1.819	0.101	1.655	0.157	PA14_36890	13.882	-0.237	0.814
PA14_36900	14.421	-0.735	0.470	-1.131	0.174	2.732	0.001	PA14_36900	37.528	-1.659	0.126
PA14_36910	51.489	-0.676	0.251	-0.911	0.066	2.483	0.000	PA14_36910	218.862	1.461	0.000
PA14_36920	56.495	-0.654	0.260	-1.003	0.039	2.740	0.000	PA14_36920	170.687	1.118	0.000
PA14_36930	12.782	-0.283	0.842	0.684	0.474	4.191	0.000	PA14_36930	14.280	-0.956	0.274
PA14_36940	30.564	0.181	0.843	0.658	0.281	2.521	0.000	PA14_36940	39.987	0.562	0.294
PA14_36960	11.651	-0.567	0.581	0.239	0.779	1.388	0.117	PA14_36960	25.390	0.176	0.823
PA14_36980	79.049	-1.600	0.000	-1.004	0.012	1.087	0.023	PA14_36980	270.190	-1.940	0.000
PA14_36990	8.002	0.043	0.979	1.177	0.229	2.347	0.025	PA14_36990	9.503	1.499	0.161
PA14_37000	6.173	-0.165	0.912	-0.987	0.332	0.190	0.891	PA14_37000	16.441	0.650	0.441
PA14_37010	23.052	0.193	0.828	-0.333	0.617	1.122	0.122	PA14_37010	30.209	0.593	0.487
PA14_37030	23.507	-0.526	0.486	-0.214	0.746	0.635	0.412	PA14_37030	39.957	1.430	0.004
PA14_37040	4.447	-0.049	0.977	0.116	0.927	0.990	0.430	PA14_37040	13.982	1.393	0.115
PA14_37060	28.374	-0.340	0.646	-1.642	0.003	-1.215	0.114	PA14_37060	83.969	1.108	0.003

PA14_37070	2981.062	0.219	0.476	-1.107	0.000	-2.973	0.000	PA14_37070	9077.722	1.646	0.000
PA14_37080	66.998	-0.781	0.130	-1.883	0.000	-3.940	0.000	PA14_37080	677.124	1.515	0.000
PA14_37090	18.361	0.071	0.945	0.230	0.749	0.283	0.770	PA14_37090	51.787	0.979	0.088
PA14_37100	26.399	-0.367	0.631	-0.221	0.729	1.330	0.042	PA14_37100	43.447	0.724	0.149
PA14_37120	31.804	-0.450	0.547	-0.964	0.097	-0.569	0.491	PA14_37120	103.568	0.878	0.105
PA14_37130	79.217	-0.534	0.248	-1.261	0.001	0.012	0.985	PA14_37130	169.484	-1.392	0.000
PA14_37140	82.100	-0.179	0.740	-0.855	0.024	-1.502	0.004	PA14_37140	200.062	-1.240	0.028
PA14_37150	8.157	-0.234	0.855	-0.517	0.582	0.083	0.948	PA14_37150	54.774	-2.009	0.000
PA14_37170	21.827	-0.448	0.567	-0.115	0.867	0.540	0.503	PA14_37170	78.895	-1.044	0.005
PA14_37190	67.882	-0.870	0.041	-1.550	0.000	-1.958	0.000	PA14_37190	216.250	-0.801	0.004
PA14_37200	148.319	0.372	0.363	0.622	0.058	0.775	0.067	PA14_37200	237.738	0.894	0.001
PA14_37210	155.120	-2.477	0.000	-5.200	0.000	-4.108	0.000	PA14_37210	595.935	-1.853	0.000
PA14_37220	73.342	-0.546	0.232	-0.231	0.583	-0.026	0.967	PA14_37220	154.563	0.245	0.460
PA14_37250	615.119	-2.834	0.000	-5.127	0.000	-3.568	0.000	PA14_37250	1346.947	-1.522	0.000
PA14_37260	452.294	-1.705	0.000	-5.472	0.000	-4.464	0.000	PA14_37260	310.075	-0.709	0.006
PA14_37270	135.380	-2.264	0.000	-5.736	0.000	-5.143	0.000	PA14_37270	91.283	-0.929	0.010
PA14_37290	70.406	-1.519	0.001	-4.667	0.000	-5.989	0.000	PA14_37290	49.324	-0.628	0.248
PA14_37310	85.753	-2.505	0.000	-6.528	0.000	-6.587	0.000	PA14_37310	72.126	-0.587	0.139
PA14_37320	41.208	-1.201	0.025	-4.323	0.000	-3.662	0.000	PA14_37320	24.463	0.813	0.210
PA14_37340	49.845	-0.653	0.211	-0.416	0.368	0.319	0.617	PA14_37340	141.068	-1.198	0.000
PA14_37350	16.598	-1.036	0.216	-1.403	0.052	1.417	0.075	PA14_37350	50.851	-1.539	0.001
PA14_37360	4.770	1.333	0.268	0.914	0.403	1.108	0.381	PA14_37360	10.935	-0.041	1.000
PA14_37370	6.411	0.014	0.991	-0.537	0.599	-0.461	0.727	PA14_37370	11.818	0.591	0.570
PA14_37380	30.568	0.252	0.734	-0.423	0.466	0.051	0.954	PA14_37380	50.059	-0.389	0.449
PA14_37400	23.538	0.129	0.884	0.208	0.745	-0.662	0.446	PA14_37400	65.292	1.387	0.001
PA14_37410	18.834	-0.474	0.578	-0.131	0.860	-0.883	0.341	PA14_37410	55.915	0.452	0.359
PA14_37420	9.955	-1.183	0.201	-0.956	0.238	-0.912	0.389	PA14_37420	26.199	0.697	0.282
PA14_37430	102.550	0.130	0.827	-0.480	0.248	0.464	0.397	PA14_37430	163.400	1.031	0.001
PA14_37440	24.167	-1.431	0.016	-1.419	0.008	-0.472	0.542	PA14_37440	59.129	-1.063	0.013
PA14_37460	22.170	-1.572	0.016	-1.307	0.026	-3.384	0.000	PA14_37460	37.465	0.020	1.000
PA14_37470	7.452	-1.260	0.224	-1.607	0.072	-0.690	0.559	PA14_37470	27.431	-0.451	0.517
PA14_37490	100.561	-1.084	0.003	-0.916	0.006	-1.136	0.013	PA14_37490	173.506	-0.158	0.650
PA14_37510	11.970	-1.938	0.026	-1.228	0.115	-2.709	0.007	PA14_37510	16.932	0.719	0.384
PA14_37520	14.297	-1.094	0.188	-0.868	0.235	-1.772	0.063	PA14_37520	18.967	0.977	0.197
PA14_37530	23.143	-1.415	0.022	-1.243	0.024	-0.932	0.221	PA14_37530	35.084	0.764	0.166
PA14_37550	11.029	-1.109	0.196	-1.268	0.087	-0.973	0.330	PA14_37550	15.954	0.169	0.852
PA14_37560	73.693	-1.464	0.000	-1.232	0.001	-2.250	0.000	PA14_37560	82.013	1.136	0.002
PA14_37570	242.350	-0.223	0.620	-0.828	0.012	-2.079	0.000	PA14_37570	640.759	0.877	0.016
PA14_37580	739.648	-0.082	0.844	-0.742	0.006	-2.261	0.000	PA14_37580	2604.974	-0.449	0.042
PA14_37590	1050.072	0.072	0.906	-0.799	0.040	-2.513	0.000	PA14_37590	1428.624	1.796	0.000
PA14_37610	827.306	-0.589	0.036	-2.035	0.000	-3.635	0.000	PA14_37610	1280.834	1.955	0.000
PA14_37630	136.570	-0.270	0.578	-1.521	0.000	-2.475	0.000	PA14_37630	162.466	1.474	0.000
PA14_37640	24.469	0.125	0.901	-0.512	0.467	0.956	0.238	PA14_37640	27.598	0.212	0.741
PA14_37650	11.465	0.034	0.977	-0.606	0.453	0.824	0.387	PA14_37650	23.356	0.292	0.741
PA14_37660	223.453	-0.611	0.070	-0.990	0.001	-0.242	0.591	PA14_37660	487.557	0.484	0.208
PA14_37670	719.397	-0.538	0.203	-3.898	0.000	-1.826	0.000	PA14_37670	3499.307	-4.602	0.000
PA14_37680	255.783	-0.503	0.183	-1.378	0.000	-0.680	0.115	PA14_37680	1133.064	-0.872	0.000
PA14_37690	287.805	-2.841	0.000	-2.884	0.000	-0.266	0.507	PA14_37690	2025.413	-3.464	0.000
PA14_37710	1738.306	-2.059	0.000	-2.324	0.000	-0.589	0.045	PA14_37710	8223.294	-1.587	0.000
PA14_37730	39.120	-0.480	0.428	-0.788	0.105	1.511	0.005	PA14_37730	78.499	-0.876	0.020
PA14_37745	391.037	-5.817	0.000	-7.925	0.000	-3.256	0.000	PA14_37745	292.690	-3.240	0.000
PA14_37760	250.677	-4.193	0.000	-4.871	0.000	-1.549	0.000	PA14_37760	421.408	-2.556	0.000
PA14_37770	67.939	-4.753	0.000	-4.842	0.000	-3.435	0.000	PA14_37770	94.275	-2.366	0.000
PA14_37780	130.927	-4.452	0.000	-4.594	0.000	-2.753	0.000	PA14_37780	172.813	-2.409	0.001
PA14_37790	19.533	-2.330	0.001	-3.030	0.000	-0.521	0.577	PA14_37790	37.635	-0.790	0.146
PA14_37810	11.545	-1.978	0.015	-1.956	0.007	-0.875	0.370	PA14_37810	12.940	-0.210	0.872
PA14_37820	75.427	0.930	0.035	1.594	0.000	-0.002	0.998	PA14_37820	120.956	1.530	0.000
PA14_37830	67.687	-2.673	0.000	-2.203	0.000	-2.043	0.000	PA14_37830	66.688	-0.077	0.898
PA14_37840	32.147	-0.097	0.915	0.308	0.616	-0.544	0.520	PA14_37840	102.328	-0.540	0.124

PA14_37850	3.730	-0.539	0.705	-0.819	0.465	-0.588	0.669
PA14_37870	3.125	-1.534	0.240	-1.017	0.386	-0.152	0.921
PA14_37880	11.557	-0.056	0.966	0.541	0.513	1.389	0.130
PA14_37900	47.548	-0.102	0.893	0.981	0.027	2.060	0.000
PA14_37910	73.509	0.993	0.017	1.326	0.000	-0.252	0.707
PA14_37915	13.187	1.239	0.152	1.438	0.054	0.718	0.504
PA14_37940	84.364	0.660	0.167	1.263	0.001	-0.976	0.116
PA14_37950	28.525	-0.337	0.686	0.320	0.621	-0.507	0.571
PA14_37965	179.087	1.160	0.001	0.655	0.055	-0.042	0.944
PA14_37980	6.173	0.108	0.943	0.940	0.325	0.534	0.679
PA14_37990	7.962	-0.734	0.486	-2.845	0.002	-1.529	0.158
PA14_38000	38.100	-0.776	0.191	-1.506	0.003	-0.304	0.694
PA14_38010	90.504	-1.217	0.006	-0.789	0.056	0.532	0.335
PA14_38020	29.072	-0.222	0.807	0.802	0.178	1.830	0.007
PA14_38040	174.798	-0.808	0.014	-0.343	0.278	-0.206	0.657
PA14_38050	11.876	-2.600	0.002	-2.641	0.001	-0.860	0.393
PA14_38060	120.968	-0.570	0.152	-0.244	0.511	1.467	0.000
PA14_38080	760.884	0.344	0.281	0.622	0.018	0.387	0.289
PA14_38090	67.224	-0.063	0.924	-0.014	0.978	0.782	0.132
PA14_38110	526.500	-0.469	0.114	-0.621	0.017	1.037	0.001
PA14_38130	207.243	1.004	0.003	-0.673	0.046	0.450	0.324
PA14_38140	986.067	1.707	0.000	0.404	0.186	1.063	0.003
PA14_38160	90.310	0.022	0.975	0.015	0.975	-1.038	0.063
PA14_38170	121.315	-0.177	0.722	-1.146	0.001	-1.578	0.001
PA14_38180	1875.878	0.002	0.997	-0.576	0.085	0.888	0.031
PA14_38190	42.615	-0.495	0.405	-1.554	0.001	0.525	0.411
PA14_38200	135.893	-0.223	0.649	-0.562	0.126	0.109	0.852
PA14_38210	23.187	-0.339	0.764	0.945	0.205	3.668	0.000
PA14_38220	61.522	-0.665	0.310	0.548	0.290	3.290	0.000
PA14_38250	199.407	1.072	0.001	1.810	0.000	-0.018	0.973
PA14_38260	160.860	-2.424	0.000	-1.649	0.000	-0.450	0.446
PA14_38270	97.500	-1.176	0.007	-1.170	0.003	2.550	0.000
PA14_38290	17.934	0.537	0.494	0.090	0.906	0.166	0.869
PA14_38300	114.233	0.259	0.608	0.088	0.845	0.245	0.670
PA14_38310	37.502	-0.293	0.705	-1.140	0.045	0.286	0.730
PA14_38320	482.893	1.936	0.000	1.729	0.000	0.512	0.184
PA14_38330	2823.801	-0.226	0.619	0.004	0.993	0.690	0.116
PA14_38340	181.786	-1.821	0.000	-2.086	0.000	2.276	0.000
PA14_38350	3032.253	-0.969	0.000	-0.823	0.002	-0.367	0.327
PA14_38360	1226.306	-0.440	0.428	0.678	0.135	2.132	0.000
PA14_38370	152.074	-1.249	0.016	-1.378	0.003	1.383	0.016
PA14_38380	315.207	0.574	0.056	1.129	0.000	0.101	0.823
PA14_38395	25.007	0.652	0.436	2.409	0.000	2.645	0.000
PA14_38410	76.964	0.016	0.979	0.801	0.027	1.207	0.007
PA14_38420	129.523	0.207	0.652	0.566	0.089	1.175	0.003
PA14_38430	11636.837	-0.457	0.235	-0.917	0.004	-1.436	0.000
PA14_38440	7439.550	-1.019	0.000	-2.701	0.000	-1.883	0.000
PA14_38460	4228.818	-1.132	0.002	-2.890	0.000	-1.914	0.000
PA14_38470	1646.248	-1.028	0.016	-2.754	0.000	-1.688	0.000
PA14_38480	1845.101	-1.598	0.000	-3.549	0.000	-2.702	0.000
PA14_38490	568.477	-1.694	0.000	-3.089	0.000	-2.530	0.000
PA14_38500	337.650	0.227	0.549	-1.243	0.000	0.488	0.206
PA14_38510	3575.156	0.189	0.621	-3.766	0.000	-0.635	0.085
PA14_38530	1161.196	-0.185	0.594	-4.806	0.000	-1.801	0.000
PA14_38550	1870.466	0.101	0.829	-4.085	0.000	-0.168	0.727
PA14_38560	179.148	0.847	0.008	-2.759	0.000	-0.395	0.386
PA14_38570	193.952	1.132	0.000	-0.433	0.204	0.042	0.939
PA14_38580	304.345	-2.195	0.000	-3.867	0.000	0.430	0.357
PA14_38590	166.142	-3.352	0.000	-4.209	0.000	-1.113	0.011

PA14_37850	7.321	0.942	0.463
PA14_37870	5.235	0.131	1.000
PA14_37880	22.018	0.579	0.506
PA14_37900	99.683	-0.403	0.509
PA14_37910	103.618	2.090	0.000
PA14_37915	21.883	0.389	0.603
PA14_37940	206.026	1.404	0.000
PA14_37950	31.828	-0.763	0.172
PA14_37965	186.170	-0.826	0.005
PA14_37980	19.882	0.808	0.274
PA14_37990	12.433	2.440	0.011
PA14_38000	141.098	0.999	0.002
PA14_38010	154.174	0.954	0.002
PA14_38020	78.504	-1.561	0.000
PA14_38040	487.548	-1.090	0.020
PA14_38050	77.104	-0.875	0.154
PA14_38060	282.451	0.182	0.568
PA14_38080	815.032	0.716	0.002
PA14_38090	75.127	1.811	0.000
PA14_38110	478.515	0.450	0.076
PA14_38130	229.618	1.955	0.000
PA14_38140	2513.294	1.743	0.000
PA14_38160	272.889	-0.003	0.985
PA14_38170	170.793	0.242	0.443
PA14_38180	4785.367	-0.088	0.878
PA14_38190	110.586	-0.239	0.556
PA14_38200	524.449	-0.742	0.002
PA14_38210	30.385	-0.125	0.870
PA14_38220	74.030	-0.562	0.148
PA14_38250	437.977	1.273	0.000
PA14_38260	836.377	-2.382	0.000
PA14_38270	397.981	-2.336	0.000
PA14_38290	70.757	0.038	0.965
PA14_38300	241.631	-0.816	0.003
PA14_38310	39.013	0.122	0.860
PA14_38320	2461.160	-2.615	0.000
PA14_38330	8207.073	-1.000	0.011
PA14_38340	850.367	-4.234	0.000
PA14_38350	9096.648	0.554	0.009
PA14_38360	2730.324	0.536	0.156
PA14_38370	1905.729	-4.320	0.000
PA14_38380	765.808	0.656	0.234
PA14_38395	196.151	2.854	0.083
PA14_38410	441.139	2.251	0.207
PA14_38420	595.908	0.185	0.489
PA14_38430	24263.820	1.641	0.000
PA14_38440	16997.321	2.409	0.000
PA14_38460	6638.062	1.441	0.000
PA14_38470	2781.583	1.227	0.000
PA14_38480	4188.245	1.247	0.000
PA14_38490	1724.500	1.084	0.000
PA14_38500	1079.078	-2.048	0.000
PA14_38510	4768.250	-1.630	0.000
PA14_38530	1532.203	-1.579	0.000
PA14_38550	2146.811	-2.267	0.000
PA14_38560	169.150	-1.169	0.001
PA14_38570	147.322	0.734	0.019
PA14_38580	100.687	0.867	0.014
PA14_38590	114.273	0.077	0.873

PA14_38610	317.548	-0.552	0.195	-2.928	0.000	-0.744	0.123	PA14_38610	323.485	1.430	0.000
PA14_38630	909.761	-1.917	0.000	-4.805	0.000	-1.518	0.000	PA14_38630	1129.196	0.371	0.104
PA14_38640	1257.043	-2.292	0.000	-6.379	0.000	-2.113	0.000	PA14_38640	1651.817	0.261	0.258
PA14_38660	2983.528	-2.598	0.000	-6.813	0.000	-2.182	0.000	PA14_38660	4828.523	-0.265	0.237
PA14_38680	82.007	0.679	0.143	1.564	0.000	2.031	0.000	PA14_38680	114.879	1.356	0.000
PA14_38690	200.212	0.093	0.867	-0.751	0.039	-0.441	0.391	PA14_38690	555.482	-0.262	0.329
PA14_38700	136.588	-0.199	0.665	0.187	0.609	-0.599	0.204	PA14_38700	557.345	0.169	0.816
PA14_38710	24.353	-0.821	0.239	-1.515	0.010	-0.757	0.357	PA14_38710	213.463	-1.230	0.000
PA14_38720	82.945	-1.297	0.004	-0.573	0.177	-0.849	0.132	PA14_38720	367.789	-1.081	0.000
PA14_38730	62.652	0.752	0.148	1.791	0.000	1.647	0.002	PA14_38730	96.918	1.756	0.001
PA14_38740	283.653	0.922	0.007	-0.271	0.455	-0.730	0.105	PA14_38740	280.258	0.848	0.001
PA14_38750	105.747	0.847	0.027	0.612	0.087	0.056	0.928	PA14_38750	201.539	0.306	0.328
PA14_38770	90.647	-1.079	0.006	-0.633	0.079	0.175	0.750	PA14_38770	226.159	-0.738	0.008
PA14_38780	56.368	-1.254	0.005	-1.347	0.001	-0.123	0.852	PA14_38780	201.065	-0.910	0.001
PA14_38790	36.794	-0.022	0.978	-0.798	0.119	-0.731	0.309	PA14_38790	124.862	-0.720	0.039
PA14_38800	58.138	-1.017	0.039	-1.637	0.000	0.165	0.812	PA14_38800	233.608	-0.790	0.005
PA14_38820	69.262	-1.579	0.000	-1.677	0.000	0.393	0.485	PA14_38820	300.580	-0.945	0.002
PA14_38825	77.619	-1.336	0.001	-1.423	0.000	0.583	0.236	PA14_38825	316.070	-1.372	0.000
PA14_38840	13.823	-0.461	0.648	-0.711	0.369	-0.095	0.935	PA14_38840	384.853	-1.547	0.000
PA14_38850	10.799	0.282	0.809	-0.036	0.971	2.026	0.021	PA14_38850	16.272	0.435	0.667
PA14_38860	43.287	0.446	0.526	0.426	0.467	1.023	0.136	PA14_38860	65.176	-0.170	0.684
PA14_38880	6.197	0.932	0.418	0.927	0.337	1.657	0.124	PA14_38880	8.872	1.345	0.226
PA14_38900	6.525	-0.813	0.494	-0.367	0.723	0.083	0.951	PA14_38900	16.432	0.760	0.369
PA14_38910	4.577	1.358	0.285	1.019	0.373	2.754	0.015	PA14_38910	9.476	-0.176	0.937
PA14_38920	4.111	1.096	0.402	0.484	0.690	1.314	0.294	PA14_38920	8.320	0.176	0.981
PA14_38930	75.493	0.686	0.126	0.280	0.524	0.584	0.284	PA14_38930	97.625	0.712	0.047
PA14_38950	0.574	-0.074	0.967	-0.744	0.533	-0.429	0.719	PA14_38950	1.300	0.387	1.000
PA14_38970	23.583	-0.341	0.651	-0.687	0.235	-1.925	0.018	PA14_38970	90.431	-0.096	0.859
PA14_38990	32.032	0.283	0.695	1.021	0.038	0.737	0.285	PA14_38990	68.429	0.479	0.275
PA14_39000	21.000	-0.382	0.644	-0.048	0.948	0.301	0.740	PA14_39000	26.572	-0.379	0.585
PA14_39010	46.739	0.040	0.962	-0.240	0.657	-0.944	0.168	PA14_39010	82.723	0.040	0.970
PA14_39020	31.833	0.644	0.339	-0.407	0.523	2.487	0.000	PA14_39020	54.110	0.272	0.599
PA14_39050	36.656	0.401	0.542	1.536	0.001	0.581	0.408	PA14_39050	55.991	1.413	0.001
PA14_39060	1.948	0.606	0.709	1.765	0.141	1.402	0.271	PA14_39060	13.624	-2.105	0.128
PA14_39070	105.096	-0.634	0.102	-0.474	0.175	-1.568	0.001	PA14_39070	217.280	0.366	0.231
PA14_39080	377.541	0.154	0.788	0.651	0.105	-0.167	0.789	PA14_39080	655.743	0.254	0.636
PA14_39090	372.895	-0.706	0.052	-0.737	0.025	-1.874	0.000	PA14_39090	872.406	-1.969	0.000
PA14_39100	131.939	0.018	0.974	0.506	0.123	-0.581	0.217	PA14_39100	253.317	0.719	0.326
PA14_39110	58.390	-0.073	0.923	0.696	0.132	-0.179	0.816	PA14_39110	97.091	1.681	0.007
PA14_39130	2111.856	0.971	0.000	1.274	0.000	1.812	0.000	PA14_39130	1974.912	1.861	0.000
PA14_39140	88.632	-0.657	0.118	-0.537	0.150	0.260	0.630	PA14_39140	351.938	-0.104	0.723
PA14_39150	36.279	-0.054	0.958	1.587	0.004	-0.770	0.386	PA14_39150	63.582	1.127	0.007
PA14_39160	148.789	0.203	0.656	0.865	0.006	1.269	0.001	PA14_39160	301.999	0.563	0.215
PA14_39180	44.506	0.207	0.766	1.219	0.007	0.558	0.405	PA14_39180	79.896	-0.429	0.270
PA14_39190	573.305	0.358	0.402	2.043	0.000	1.361	0.001	PA14_39190	795.770	0.995	0.000
PA14_39200	59.548	0.493	0.357	0.920	0.029	0.556	0.360	PA14_39200	108.337	0.696	0.045
PA14_39210	29.407	0.618	0.352	0.901	0.091	0.582	0.448	PA14_39210	37.302	1.450	0.005
PA14_39220	2.683	-0.385	0.817	0.084	0.952	0.228	0.882	PA14_39220	2.633	1.925	0.480
PA14_39230	4.656	-0.039	0.980	-0.479	0.674	0.658	0.614	PA14_39230	8.587	-1.101	0.379
PA14_39240	48.225	-0.121	0.862	-0.278	0.579	1.297	0.014	PA14_39240	128.214	-1.190	0.000
PA14_39250	3.632	-0.053	0.975	-0.280	0.818	0.126	0.933	PA14_39250	7.338	-0.159	0.982
PA14_39260	10.916	1.332	0.215	2.218	0.012	1.978	0.062	PA14_39260	12.242	1.584	0.082
PA14_39270	119.456	-2.075	0.000	-3.341	0.000	0.136	0.796	PA14_39270	413.725	-2.498	0.000
PA14_39280	221.142	0.336	0.400	0.035	0.930	-0.292	0.543	PA14_39280	1148.449	1.916	0.000
PA14_39300	385.765	0.720	0.010	0.818	0.001	0.203	0.618	PA14_39300	1929.373	1.561	0.000
PA14_39320	320.657	0.028	0.958	0.662	0.028	-0.222	0.634	PA14_39320	1969.290	2.405	0.000
PA14_39330	629.814	0.340	0.307	0.948	0.000	-0.181	0.670	PA14_39330	4107.335	2.033	0.000
PA14_39350	3372.793	0.856	0.000	1.360	0.000	0.772	0.009	PA14_39350	17854.170	1.105	0.000
PA14_39360	83.442	0.026	0.972	0.198	0.666	1.407	0.003	PA14_39360	206.756	0.439	0.144

PA14_39390	754.912	-0.575	0.066	-0.881	0.001	0.825	0.019	PA14_39390	2185.828	-0.033	0.955
PA14_39410	127.080	-1.012	0.003	-1.294	0.000	0.424	0.330	PA14_39410	560.617	-1.086	0.000
PA14_39420	46.369	-0.018	0.980	-0.429	0.390	-0.783	0.241	PA14_39420	778.385	-3.848	0.000
PA14_39440	396.284	-0.359	0.268	-1.162	0.000	-1.786	0.000	PA14_39440	955.641	-0.351	0.127
PA14_39460	399.936	-0.332	0.522	-1.712	0.000	-1.559	0.002	PA14_39460	905.961	-0.853	0.067
PA14_39470	3417.401	-0.963	0.000	-1.912	0.000	-1.767	0.000	PA14_39470	8850.749	-0.265	0.427
PA14_39480	5027.967	-1.008	0.000	-2.428	0.000	-1.919	0.000	PA14_39480	17537.409	-0.678	0.001
PA14_39500	49.964	-0.748	0.159	-1.061	0.020	-0.869	0.168	PA14_39500	133.201	0.010	1.000
PA14_39520	94.537	-0.708	0.154	-0.823	0.056	1.535	0.002	PA14_39520	143.098	-0.926	0.015
PA14_39530	68.272	-0.868	0.083	-0.332	0.475	2.149	0.000	PA14_39530	88.193	-0.046	0.937
PA14_39540	61.660	-1.041	0.040	-1.477	0.001	2.568	0.000	PA14_39540	259.242	-2.647	0.000
PA14_39560	523.108	-2.979	0.000	-3.679	0.000	2.072	0.000	PA14_39560	5844.175	-5.619	0.000
PA14_39570	16.611	0.433	0.637	0.333	0.662	1.091	0.203	PA14_39570	34.615	0.818	0.141
PA14_39580	34.389	-0.478	0.478	-0.913	0.086	0.608	0.380	PA14_39580	60.490	0.380	0.507
PA14_39590	187.761	0.204	0.642	1.032	0.001	1.289	0.001	PA14_39590	258.026	0.163	0.629
PA14_39610	1005.584	0.856	0.009	1.848	0.000	1.878	0.000	PA14_39610	668.100	1.793	0.000
PA14_39620	16.974	-0.402	0.655	-1.635	0.020	-1.042	0.274	PA14_39620	19.892	-0.247	0.830
PA14_39630	2.963	-0.740	0.636	0.865	0.495	-0.131	0.932	PA14_39630	3.898	1.869	0.322
PA14_39640	28.445	-0.560	0.538	0.318	0.668	0.335	0.740	PA14_39640	22.896	0.150	0.873
PA14_39650	22.945	-0.667	0.516	0.201	0.818	-0.159	0.897	PA14_39650	20.671	-0.700	0.354
PA14_39660	53.314	-1.263	0.014	-0.788	0.092	-0.164	0.824	PA14_39660	109.337	-0.845	0.013
PA14_39670	4.052	-1.054	0.426	-0.974	0.385	-0.572	0.680	PA14_39670	32.533	-0.940	0.093
PA14_39690	639.476	-0.443	0.274	-0.089	0.826	-1.497	0.000	PA14_39690	2892.641	0.951	0.000
PA14_39700	216.307	-1.578	0.000	-3.657	0.000	-3.579	0.000	PA14_39700	1949.766	-0.776	0.001
PA14_39710	20.056	-1.095	0.129	-1.075	0.086	-0.686	0.438	PA14_39710	188.015	1.375	0.000
PA14_39720	55.748	1.135	0.116	4.853	0.000	3.307	0.000	PA14_39720	35.588	1.585	0.003
PA14_39730	15.812	0.720	0.503	3.384	0.000	2.043	0.028	PA14_39730	12.391	1.278	0.161
PA14_39750	35.511	0.948	0.197	3.905	0.000	2.117	0.003	PA14_39750	17.539	2.600	0.001
PA14_39770	230.154	-0.467	0.154	-0.413	0.156	0.209	0.621	PA14_39770	828.504	-0.744	0.001
PA14_39780	100.978	-2.404	0.000	-2.154	0.000	0.989	0.017	PA14_39780	452.696	-2.944	0.000
PA14_39790	206.496	-1.077	0.000	-1.934	0.000	-1.354	0.000	PA14_39790	383.886	-0.888	0.000
PA14_39800	99.724	-0.559	0.281	-0.807	0.062	1.120	0.031	PA14_39800	75.705	-0.242	0.782
PA14_39810	12.499	-0.868	0.373	-0.944	0.247	0.243	0.835	PA14_39810	5.642	0.332	0.899
PA14_39820	57.731	-0.917	0.064	-1.493	0.001	0.984	0.063	PA14_39820	39.471	0.327	0.561
PA14_39830	30.383	-0.779	0.234	-0.273	0.648	0.960	0.148	PA14_39830	33.598	0.305	0.611
PA14_39850	4.905	0.711	0.593	0.943	0.373	1.010	0.422	PA14_39850	7.131	0.659	0.636
PA14_39860	34.436	-0.642	0.359	-0.529	0.373	1.387	0.033	PA14_39860	31.660	0.831	0.341
PA14_39870	29.140	0.278	0.708	0.162	0.794	0.038	0.967	PA14_39870	59.700	0.869	0.044
PA14_39880	134.769	-6.498	0.000	-7.540	0.000	-5.954	0.000	PA14_39880	3827.968	-5.325	0.000
PA14_39890	0.000	NA	NA	NA	NA	NA	NA	PA14_39890	2447.945	-5.281	0.000
PA14_39910	0.379	-1.349	0.214	-1.389	0.160	-1.193	0.178	PA14_39910	2130.217	-4.122	0.000
PA14_39925	0.000	NA	NA	NA	NA	NA	NA	PA14_39925	697.231	-4.147	0.000
PA14_39945	279.902	-5.839	0.000	-8.004	0.000	-6.886	0.000	PA14_39945	1065.860	-4.032	0.000
PA14_39960	4610.695	-5.688	0.000	-8.030	0.000	-6.091	0.000	PA14_39960	2919.984	-4.001	0.000
PA14_39970	1783.438	-5.705	0.000	-9.060	0.000	-7.187	0.000	PA14_39970	1364.579	-3.919	0.000
PA14_39980	123.908	-1.436	0.000	-2.342	0.000	-0.851	0.044	PA14_39980	378.174	-0.200	0.471
PA14_39990	221.298	-2.908	0.000	-5.756	0.000	-3.779	0.000	PA14_39990	112.206	-1.760	0.000
PA14_40010	104.772	-3.199	0.000	-5.321	0.000	-3.714	0.000	PA14_40010	93.047	-2.096	0.000
PA14_40020	478.769	-3.650	0.000	-6.167	0.000	-3.659	0.000	PA14_40020	319.472	-2.660	0.000
PA14_40030	263.812	-3.445	0.000	-6.080	0.000	-4.188	0.000	PA14_40030	163.571	-2.328	0.000
PA14_40040	115.132	-2.686	0.000	-4.136	0.000	-2.757	0.000	PA14_40040	72.319	-1.118	0.005
PA14_40050	36.841	-2.598	0.000	-3.821	0.000	-3.506	0.000	PA14_40050	42.247	-1.159	0.016
PA14_40060	4.119	-2.119	0.068	-2.283	0.029	-1.134	0.368	PA14_40060	10.122	-0.281	0.866
PA14_40070	110.349	-0.709	0.098	-0.408	0.302	-0.810	0.115	PA14_40070	298.273	0.070	0.841
PA14_40080	244.919	-1.111	0.000	-0.701	0.016	-0.333	0.429	PA14_40080	1817.557	-0.332	0.361
PA14_40100	102.773	-2.226	0.000	-2.840	0.000	-0.471	0.351	PA14_40100	816.918	-2.903	0.000
PA14_40110	26.901	-2.033	0.001	-2.391	0.000	0.227	0.788	PA14_40110	177.873	-2.831	0.000
PA14_40120	151.418	-0.207	0.660	-0.039	0.927	0.547	0.231	PA14_40120	499.980	0.275	0.303
PA14_40130	97.085	-0.173	0.748	-0.154	0.720	-0.920	0.078	PA14_40130	400.224	0.347	0.338

PA14_40150	51.515	-0.516	0.370	-0.408	0.407	-0.433	0.526	PA14_40150	149.490	-0.250	0.427
PA14_40160	25.937	-0.968	0.148	-0.038	0.955	0.122	0.892	PA14_40160	137.838	-0.716	0.104
PA14_40170	9.024	-0.770	0.498	0.678	0.439	0.441	0.714	PA14_40170	79.255	-1.532	0.000
PA14_40180	508.927	-1.702	0.000	-2.247	0.000	0.656	0.123	PA14_40180	1311.246	-2.832	0.000
PA14_40200	364.821	-1.972	0.000	-2.228	0.000	-0.438	0.268	PA14_40200	1071.427	-2.370	0.000
PA14_40210	21.148	-0.256	0.772	0.200	0.770	1.560	0.023	PA14_40210	46.855	0.843	0.091
PA14_40220	32.875	-0.355	0.635	0.124	0.845	0.175	0.841	PA14_40220	71.416	0.426	0.323
PA14_40230	337.867	-1.069	0.001	-1.173	0.000	3.082	0.000	PA14_40230	324.074	-0.755	0.003
PA14_40240	134.245	-1.511	0.000	-1.870	0.000	2.102	0.000	PA14_40240	168.812	-0.910	0.003
PA14_40250	180.099	-1.300	0.000	-0.983	0.002	2.350	0.000	PA14_40250	471.450	-1.618	0.000
PA14_40260	733.960	-2.440	0.000	-2.632	0.000	1.783	0.000	PA14_40260	2300.570	-1.844	0.000
PA14_40270	30.285	0.170	0.843	0.533	0.360	0.135	0.887	PA14_40270	65.012	0.974	0.021
PA14_40280	364.030	0.023	0.965	0.098	0.779	-0.025	0.962	PA14_40280	854.978	0.306	0.207
PA14_40290	463.115	-4.111	0.000	-5.804	0.000	1.045	0.006	PA14_40290	2547.914	-4.578	0.000
PA14_40300	132.742	-1.594	0.000	-2.849	0.000	0.906	0.062	PA14_40300	554.193	-2.149	0.000
PA14_40310	263.045	-2.278	0.000	-4.371	0.000	-1.645	0.000	PA14_40310	1114.469	-1.600	0.000
PA14_40320	21.765	-0.237	0.799	0.095	0.899	0.783	0.338	PA14_40320	55.705	0.936	0.037
PA14_40330	3.220	-1.500	0.236	-0.999	0.375	-0.311	0.833	PA14_40330	21.566	1.071	0.119
PA14_40340	402.378	1.044	0.003	1.953	0.000	3.102	0.000	PA14_40340	525.063	1.233	0.001
PA14_40350	98.632	-0.475	0.288	-0.092	0.830	-0.070	0.910	PA14_40350	205.688	0.397	0.195
PA14_40370	123.154	-0.055	0.937	0.650	0.129	0.915	0.089	PA14_40370	311.222	0.905	0.001
PA14_40380	49.663	1.685	0.000	1.705	0.000	0.422	0.563	PA14_40380	120.847	1.200	0.000
PA14_40390	211.948	1.727	0.000	2.417	0.000	1.289	0.006	PA14_40390	1222.783	1.227	0.000
PA14_40410	83.500	0.262	0.603	1.026	0.004	-0.161	0.797	PA14_40410	436.531	1.881	0.000
PA14_40420	116.007	0.127	0.816	0.489	0.186	-0.857	0.099	PA14_40420	514.346	2.801	0.000
PA14_40430	206.394	-1.808	0.000	-2.377	0.000	1.099	0.001	PA14_40430	740.073	-2.895	0.000
PA14_40440	79.161	-0.246	0.655	-1.094	0.007	-1.005	0.071	PA14_40440	322.239	-0.308	0.277
PA14_40450	117.912	-0.189	0.691	-0.406	0.255	0.011	0.984	PA14_40450	258.334	0.510	0.062
PA14_40470	83.946	-0.256	0.668	-0.018	0.973	0.101	0.885	PA14_40470	259.136	0.195	0.609
PA14_40490	283.158	-0.527	0.152	-0.253	0.467	0.571	0.172	PA14_40490	678.098	-0.290	0.249
PA14_40510	236.518	0.530	0.173	2.616	0.000	3.719	0.000	PA14_40510	390.903	-1.265	0.000
PA14_40520	4.551	-0.477	0.748	0.666	0.549	1.636	0.159	PA14_40520	3.807	1.842	0.327
PA14_40540	113.092	1.899	0.000	3.942	0.000	5.354	0.000	PA14_40540	30.227	1.897	0.001
PA14_40550	234.222	0.974	0.007	1.768	0.000	1.288	0.002	PA14_40550	318.695	1.243	0.000
PA14_40560	311.383	-1.956	0.000	-1.985	0.000	-0.258	0.541	PA14_40560	983.451	0.327	0.313
PA14_40570	103.103	-0.761	0.067	-0.866	0.018	0.984	0.026	PA14_40570	281.728	-0.799	0.002
PA14_40600	43.446	-0.244	0.717	-0.368	0.476	-0.492	0.491	PA14_40600	125.293	-0.036	0.915
PA14_40610	3.097	0.032	0.985	0.541	0.654	-0.847	0.527	PA14_40610	10.871	0.522	0.632
PA14_40620	13.728	0.133	0.904	-0.927	0.219	-1.488	0.138	PA14_40620	37.120	0.079	0.938
PA14_40630	4194.281	-0.038	0.928	0.231	0.422	1.518	0.000	PA14_40630	16756.480	-2.108	0.000
PA14_40640	2074.203	0.779	0.061	1.709	0.000	0.810	0.096	PA14_40640	1710.215	1.266	0.000
PA14_40650	38.975	0.096	0.904	0.538	0.291	1.720	0.002	PA14_40650	68.697	0.935	0.021
PA14_40660	1.827	-0.804	0.605	-1.078	0.395	0.065	0.967	PA14_40660	7.891	0.646	0.610
PA14_40670	2323.691	-0.059	0.867	-0.297	0.220	-0.491	0.104	PA14_40670	2850.556	0.587	0.007
PA14_40690	125.920	-0.820	0.036	-0.965	0.006	-1.791	0.000	PA14_40690	394.025	-0.406	0.119
PA14_40700	33.236	0.861	0.196	1.059	0.061	0.501	0.554	PA14_40700	94.591	1.178	0.022
PA14_40710	507.380	1.008	0.002	1.794	0.000	0.698	0.098	PA14_40710	652.396	1.715	0.000
PA14_40730	496.269	0.259	0.501	1.220	0.000	0.735	0.053	PA14_40730	490.360	1.175	0.000
PA14_40740	650.020	-0.058	0.926	0.446	0.276	1.181	0.013	PA14_40740	2226.511	-2.021	0.000
PA14_40750	396.184	-2.507	0.000	-3.642	0.000	0.056	0.921	PA14_40750	1549.260	-3.982	0.000
PA14_40770	1946.015	-1.860	0.000	-1.682	0.000	-2.052	0.000	PA14_40770	1501.436	0.937	0.000
PA14_40780	106.657	-2.407	0.000	-2.847	0.000	-3.232	0.000	PA14_40780	100.436	0.648	0.069
PA14_40790	48.662	0.900	0.111	1.595	0.001	-0.542	0.505	PA14_40790	182.389	0.639	0.030
PA14_40800	91.864	-0.642	0.162	-0.577	0.155	-0.069	0.915	PA14_40800	191.280	-0.646	0.027
PA14_40820	22.152	-0.329	0.705	-0.412	0.547	0.394	0.661	PA14_40820	68.860	0.657	0.234
PA14_40830	736.214	-0.969	0.000	-1.864	0.000	-1.461	0.000	PA14_40830	4013.655	-0.182	0.434
PA14_40840	3163.351	-0.262	0.494	-0.646	0.031	0.105	0.826	PA14_40840	9869.925	-0.405	0.060
PA14_40850	4404.156	0.213	0.660	-0.952	0.007	-1.490	0.000	PA14_40850	9341.930	0.091	0.794
PA14_40860	733.176	-0.129	0.916	-1.146	0.145	-1.758	0.055	PA14_40860	2402.338	0.085	0.714

PA14_40880	903.896	0.391	0.214	0.091	0.774	1.837	0.000	PA14_40880	1755.942	0.397	0.075
PA14_40890	413.183	-0.355	0.290	-1.262	0.000	-0.461	0.225	PA14_40890	1098.556	-0.166	0.535
PA14_40900	43.706	0.812	0.159	1.847	0.000	1.540	0.010	PA14_40900	117.910	0.188	0.626
PA14_40910	56.519	-0.268	0.668	-0.817	0.077	-0.172	0.814	PA14_40910	269.365	-1.616	0.000
PA14_40930	147.038	0.474	0.261	1.710	0.000	-0.056	0.928	PA14_40930	317.640	0.813	0.002
PA14_40940	21.175	0.453	0.585	1.461	0.013	-0.524	0.599	PA14_40940	45.154	2.207	0.000
PA14_40950	819.769	0.191	0.592	0.405	0.142	-0.695	0.047	PA14_40950	931.405	0.810	0.000
PA14_40960	1101.824	-0.030	0.944	-0.105	0.731	-1.078	0.001	PA14_40960	2273.311	1.211	0.000
PA14_40980	2891.789	0.563	0.068	0.964	0.000	0.273	0.502	PA14_40980	5119.786	0.819	0.000
PA14_41000	143.646	-0.429	0.378	-0.759	0.054	-1.919	0.000	PA14_41000	388.891	1.002	0.000
PA14_41010	603.619	-0.113	0.761	-0.336	0.218	-0.608	0.076	PA14_41010	1264.332	-0.852	0.000
PA14_41020	1799.292	0.540	0.070	-0.546	0.046	-1.569	0.000	PA14_41020	2276.412	0.600	0.005
PA14_41030	1156.323	-0.262	0.400	-0.166	0.550	0.504	0.121	PA14_41030	2498.178	-0.307	0.326
PA14_41050	181.988	-0.149	0.748	-0.091	0.814	-0.363	0.443	PA14_41050	485.609	0.151	0.604
PA14_41060	945.417	0.483	0.191	1.166	0.000	1.089	0.005	PA14_41060	2162.875	-0.364	0.194
PA14_41070	673.363	0.282	0.531	1.196	0.000	0.782	0.077	PA14_41070	1622.319	-0.298	0.191
PA14_41080	624.724	-0.318	0.522	-0.561	0.158	0.294	0.601	PA14_41080	1161.240	-0.860	0.060
PA14_41090	4266.028	0.325	0.291	0.957	0.000	0.458	0.181	PA14_41090	9119.065	0.659	0.016
PA14_41110	1696.201	0.209	0.539	0.014	0.967	-0.857	0.009	PA14_41110	5120.695	0.411	0.062
PA14_41130	1316.510	0.072	0.876	-0.444	0.153	-1.195	0.001	PA14_41130	3225.371	0.983	0.000
PA14_41140	498.563	-0.247	0.557	-0.224	0.529	-1.695	0.000	PA14_41140	974.490	0.549	0.018
PA14_41150	219.498	-0.211	0.645	-0.525	0.128	-2.242	0.000	PA14_41150	762.738	0.480	0.046
PA14_41160	1067.938	-0.301	0.371	-0.881	0.001	-2.669	0.000	PA14_41160	2300.640	0.096	0.725
PA14_41170	454.247	-0.791	0.010	-1.447	0.000	-3.057	0.000	PA14_41170	1648.274	-0.355	0.122
PA14_41190	3959.919	-0.455	0.083	-0.240	0.344	-1.028	0.000	PA14_41190	4450.076	1.247	0.000
PA14_41200	266.131	0.311	0.406	0.609	0.039	-0.005	0.992	#N/A	#N/A	#N/A	#N/A
PA14_41210	4248.645	-0.098	0.859	-0.254	0.534	-1.876	0.000	PA14_41210	12355.757	-0.046	0.869
PA14_41220	53958.157	0.049	0.909	0.587	0.030	0.318	0.400	PA14_41220	82906.815	0.629	0.002
PA14_41230	29438.945	-0.122	0.724	0.136	0.625	0.840	0.005	PA14_41230	75922.159	-0.051	0.856
PA14_41240	10557.169	0.304	0.371	0.654	0.017	1.558	0.000	PA14_41240	13695.723	0.999	0.000
PA14_41250	6226.239	0.059	0.901	0.510	0.098	0.153	0.744	PA14_41250	10173.251	1.185	0.000
PA14_41260	340.222	-0.012	0.981	0.385	0.255	1.029	0.009	PA14_41260	636.046	0.774	0.001
PA14_41270	337.027	0.381	0.281	0.544	0.065	0.577	0.136	PA14_41270	412.212	1.440	0.000
PA14_41280	190.805	-1.103	0.018	-0.278	0.543	4.032	0.000	PA14_41280	638.812	2.032	0.381
PA14_41290	7.637	-0.126	0.934	0.004	0.997	2.493	0.011	PA14_41290	55.431	0.828	0.703
PA14_41300	116.298	-0.053	0.928	1.365	0.000	0.184	0.748	PA14_41300	343.870	0.851	0.001
PA14_41320	137.368	-0.070	0.909	1.345	0.000	-0.198	0.744	#N/A	#N/A	#N/A	#N/A
PA14_41330	473.296	0.684	0.075	2.353	0.000	0.348	0.489	#N/A	#N/A	#N/A	#N/A
PA14_41340	1116.571	0.253	0.607	1.375	0.000	-0.120	0.841	#N/A	#N/A	#N/A	#N/A
PA14_41350	1183.555	0.583	0.046	1.313	0.000	1.241	0.000	PA14_41350	1862.547	1.161	0.000
PA14_41360	2868.338	0.486	0.137	0.728	0.010	0.112	0.807	PA14_41360	2768.794	0.363	0.095
PA14_41380	3766.723	0.211	0.472	0.165	0.520	-0.346	0.271	PA14_41380	5393.995	1.113	0.000
PA14_41390	3025.116	0.205	0.706	-0.170	0.708	-2.088	0.000	PA14_41390	6897.674	0.572	0.351
PA14_41400	79.230	0.131	0.843	0.050	0.927	-1.048	0.086	PA14_41400	178.133	0.743	0.012
PA14_41420	646.021	0.614	0.037	0.977	0.000	-0.900	0.011	PA14_41420	864.503	2.489	0.000
PA14_41430	52.858	0.767	0.210	1.782	0.000	1.193	0.068	PA14_41430	153.619	2.793	0.000
PA14_41440	5470.921	-1.479	0.000	-3.195	0.000	-3.223	0.000	PA14_41440	24035.355	-0.313	0.272
PA14_41450	211.875	0.254	0.585	0.712	0.040	1.256	0.003	PA14_41450	368.257	0.243	0.406
PA14_41470	7807.007	0.356	0.258	-0.063	0.843	-1.556	0.000	PA14_41470	5956.348	0.926	0.000
PA14_41480	26.818	0.366	0.650	0.371	0.572	0.120	0.901	PA14_41480	41.024	0.743	0.149
PA14_41490	15.231	0.772	0.366	1.057	0.126	1.223	0.159	PA14_41490	26.967	-0.029	1.000
PA14_41500	385.849	-2.697	0.000	-2.867	0.000	0.409	0.378	PA14_41500	3239.787	-4.822	0.000
PA14_41510	2.647	-1.847	0.146	-2.980	0.008	-1.095	0.402	PA14_41510	8.300	-1.292	0.555
PA14_41520	14.988	0.146	0.899	0.245	0.770	2.001	0.013	PA14_41520	27.787	-0.241	0.739
PA14_41530	47.800	-0.404	0.491	-0.323	0.511	1.163	0.029	PA14_41530	163.206	-1.848	0.000
PA14_41540	5.478	0.881	0.463	0.003	0.998	-0.538	0.694	PA14_41540	18.698	-0.457	0.556
PA14_41560	27.703	-0.336	0.695	-0.219	0.753	0.003	0.998	PA14_41560	47.458	0.148	0.802
PA14_41563	26.547	-0.255	0.770	-0.320	0.636	0.615	0.453	PA14_41563	67.433	-0.501	0.250
PA14_41570	50010.412	-0.921	0.000	-1.375	0.000	-1.316	0.000	PA14_41570	77194.679	-0.140	0.548

PA14_41575	43823.199	-0.752	0.034	-0.697	0.034	0.491	0.268	PA14_41575	51695.911	-0.023	0.886
PA14_41590	5907.074	-0.601	0.060	-0.054	0.880	1.204	0.001	PA14_41590	7520.023	0.410	0.057
PA14_41610	718.241	-1.483	0.000	-0.544	0.029	1.297	0.000	PA14_41610	2015.730	1.592	0.000
PA14_41630	2223.755	0.383	0.428	1.393	0.000	0.242	0.676	PA14_41630	3352.029	0.467	0.054
PA14_41640	819.087	-0.924	0.001	-0.410	0.151	-0.991	0.004	PA14_41640	2769.619	-1.438	0.000
PA14_41650	177.104	1.179	0.000	1.857	0.000	0.412	0.375	PA14_41650	169.345	1.925	0.000
PA14_41670	9603.088	-0.279	0.329	-0.141	0.596	-0.228	0.503	PA14_41670	9711.895	0.427	0.046
PA14_41680	1407.510	-0.425	0.105	-0.570	0.014	-0.005	0.990	PA14_41680	3156.304	-0.652	0.003
PA14_41690	1277.248	0.785	0.006	1.273	0.000	2.460	0.000	PA14_41690	2035.485	1.100	0.000
PA14_41710	1667.751	-0.192	0.556	-0.138	0.625	0.292	0.403	PA14_41710	2584.832	1.020	0.000
PA14_41730	3173.721	-0.104	0.822	0.218	0.529	0.585	0.151	PA14_41730	3551.156	0.150	0.514
PA14_41740	103.693	-0.563	0.152	-0.907	0.007	-0.031	0.957	PA14_41740	657.443	-1.810	0.000
PA14_41750	112.118	-0.111	0.838	-0.426	0.259	0.923	0.036	PA14_41750	520.281	-1.151	0.000
PA14_41760	315.489	0.607	0.064	-0.314	0.326	1.620	0.000	PA14_41760	616.078	-0.787	0.001
PA14_41780	470.309	0.677	0.113	-0.330	0.429	2.396	0.000	PA14_41780	704.142	-0.694	0.003
PA14_41790	203.023	0.152	0.712	-1.520	0.000	1.098	0.002	PA14_41790	545.439	-0.830	0.015
PA14_41800	2334.701	0.387	0.284	-0.790	0.008	1.355	0.000	PA14_41800	3953.055	0.437	0.043
PA14_41810	2133.034	0.390	0.149	-0.319	0.200	0.575	0.059	PA14_41810	4616.831	1.234	0.000
PA14_41820	500.316	0.140	0.691	0.282	0.288	0.135	0.732	PA14_41820	1049.491	1.724	0.000
PA14_41830	180.532	-0.432	0.285	-0.143	0.710	-0.939	0.034	PA14_41830	298.678	0.824	0.002
PA14_41840	453.274	-0.749	0.034	-0.688	0.034	-0.535	0.224	PA14_41840	596.123	0.585	0.014
PA14_41860	18.123	-0.945	0.225	-0.480	0.487	-0.961	0.296	PA14_41860	81.943	-0.244	0.568
PA14_41870	3755.786	0.025	0.944	-0.419	0.061	0.408	0.164	PA14_41870	9275.351	-1.161	0.000
PA14_41880	878.416	-1.284	0.000	-1.118	0.000	1.237	0.000	PA14_41880	3927.155	-2.048	0.000
PA14_41900	772.072	-0.358	0.332	0.043	0.909	0.344	0.419	PA14_41900	1918.964	-0.168	0.491
PA14_41910	105.298	-0.531	0.189	-0.668	0.053	-0.334	0.516	PA14_41910	428.959	-0.313	0.228
PA14_41920	2760.718	0.080	0.835	-0.456	0.081	-0.746	0.022	PA14_41920	3529.670	2.012	0.000
PA14_41930	513.716	-1.207	0.000	-1.047	0.000	-1.446	0.000	PA14_41930	2837.314	0.113	0.674
PA14_41950	263.559	-0.454	0.281	-0.632	0.075	-1.105	0.014	PA14_41950	1142.915	0.186	0.454
PA14_41960	22.530	0.185	0.837	0.993	0.086	-0.786	0.383	PA14_41960	65.212	0.264	0.662
PA14_41970	1689.112	-1.240	0.000	-2.501	0.000	-3.169	0.000	PA14_41970	16315.914	-0.460	0.032
PA14_41980	190.667	-2.502	0.000	-2.288	0.000	0.444	0.343	PA14_41980	1179.195	-3.699	0.000
PA14_41990	41.268	0.372	0.556	0.516	0.297	2.095	0.000	PA14_41990	46.951	-0.621	0.295
PA14_42000	4.695	0.518	0.708	-0.585	0.611	1.672	0.143	PA14_42000	11.008	-1.655	0.089
PA14_42010	1240.755	0.309	0.401	-0.655	0.026	-0.359	0.385	PA14_42010	3435.559	-0.586	0.006
PA14_42020	67.023	-0.362	0.528	-0.275	0.569	0.125	0.860	PA14_42020	199.673	1.056	0.000
PA14_42030	6.615	-0.283	0.849	1.935	0.032	0.757	0.546	PA14_42030	24.414	0.873	0.174
PA14_42050	28.271	0.430	0.609	1.583	0.006	1.642	0.024	PA14_42050	51.661	0.690	0.146
PA14_42060	120.317	1.172	0.001	1.257	0.000	0.798	0.088	PA14_42060	201.125	0.635	0.028
PA14_42080	260.724	-1.192	0.000	-1.820	0.000	0.743	0.038	PA14_42080	1224.607	-1.333	0.000
PA14_42090	381.169	-1.221	0.000	-2.243	0.000	-0.400	0.305	PA14_42090	2837.227	-3.022	0.000
PA14_42100	68.871	0.353	0.559	0.458	0.342	0.810	0.174	PA14_42100	148.460	-0.284	0.474
PA14_42120	51.377	-0.423	0.473	-1.300	0.005	-0.276	0.696	PA14_42120	138.506	0.492	0.139
PA14_42130	277.161	-0.807	0.010	-0.910	0.001	2.094	0.000	PA14_42130	718.038	-1.300	0.000
PA14_42140	600.008	-0.088	0.876	0.181	0.660	3.228	0.000	PA14_42140	898.200	-0.297	0.224
PA14_42150	627.223	-0.329	0.409	-0.086	0.819	3.512	0.000	PA14_42150	927.211	0.254	0.287
PA14_42160	483.611	-0.484	0.191	-0.140	0.700	3.247	0.000	PA14_42160	855.892	0.506	0.029
PA14_42180	325.310	0.258	0.508	0.840	0.004	2.578	0.000	PA14_42180	933.704	0.100	0.747
PA14_42200	456.470	-1.357	0.000	-2.056	0.000	2.304	0.000	PA14_42200	1741.121	-2.608	0.000
PA14_42220	434.969	-0.134	0.741	-0.193	0.543	1.892	0.000	PA14_42220	794.644	0.919	0.000
PA14_42230	460.664	0.033	0.942	-0.316	0.279	0.554	0.124	PA14_42230	605.406	0.347	0.165
PA14_42250	265.192	2.324	0.000	2.567	0.000	-0.341	0.513	PA14_42250	644.019	2.593	0.000
PA14_42260	48.768	2.118	0.000	2.380	0.000	-0.544	0.536	PA14_42260	257.678	2.336	0.000
PA14_42270	730.545	2.154	0.000	2.548	0.000	-0.256	0.577	PA14_42270	1612.319	1.660	0.000
PA14_42280	355.698	2.436	0.000	2.327	0.000	-0.685	0.144	PA14_42280	1288.353	1.988	0.000
PA14_42290	181.205	2.413	0.000	2.769	0.000	-0.892	0.104	PA14_42290	444.331	2.063	0.000
PA14_42300	52.769	1.706	0.000	1.962	0.000	-2.047	0.012	PA14_42300	299.857	2.049	0.000
PA14_42310	1015.874	2.252	0.000	2.280	0.000	-1.757	0.000	PA14_42310	2499.031	2.416	0.000
PA14_42320	161.707	2.530	0.000	2.414	0.000	-0.363	0.534	PA14_42320	5049.524	2.269	0.000

PA14_42340	607.269	2.422	0.000	2.733	0.000	-0.545	0.145	PA14_42340	5187.623	2.278	0.000
PA14_42350	606.471	2.115	0.000	2.604	0.000	-0.735	0.059	PA14_42350	1141.894	2.841	0.000
PA14_42360	54.445	2.468	0.000	2.764	0.000	-1.385	0.112	PA14_42360	301.952	1.326	0.001
PA14_42380	1462.545	2.237	0.000	2.911	0.000	-0.077	0.899	PA14_42380	2484.750	1.260	0.000
PA14_42390	3491.073	2.027	0.000	1.922	0.000	0.084	0.855	PA14_42390	7022.964	0.581	0.006
PA14_42400	635.140	1.953	0.000	2.341	0.000	-0.372	0.382	PA14_42400	2028.578	-0.376	0.113
PA14_42410	28.853	1.471	0.012	1.336	0.013	-0.352	0.709	PA14_42410	142.221	0.041	0.988
PA14_42430	290.209	0.953	0.005	1.025	0.001	-1.735	0.000	PA14_42430	1291.169	0.423	0.070
PA14_42440	349.391	1.535	0.000	0.176	0.571	-2.346	0.000	PA14_42440	1318.875	-0.219	0.393
PA14_42450	3793.686	1.798	0.000	1.187	0.000	-2.399	0.000	PA14_42450	6975.835	-0.470	0.029
PA14_42460	938.784	1.836	0.000	0.959	0.000	-2.633	0.000	PA14_42460	2372.865	-0.312	0.351
PA14_42470	523.066	1.645	0.000	1.497	0.000	-0.901	0.014	PA14_42470	1600.549	-0.323	0.178
PA14_42480	112.918	2.090	0.000	2.506	0.000	0.903	0.073	PA14_42480	414.063	-0.456	0.097
PA14_42490	60.447	1.601	0.001	2.081	0.000	1.423	0.013	PA14_42490	87.666	1.066	0.004
PA14_42500	427.635	2.126	0.000	2.164	0.000	0.530	0.207	PA14_42500	578.944	0.275	0.350
PA14_42510	5.503	2.811	0.008	2.546	0.010	0.187	0.902	PA14_42510	18.299	1.002	0.187
PA14_42520	9.402	2.694	0.002	1.645	0.063	0.838	0.494	PA14_42520	29.277	-0.005	1.000
PA14_42530	8.890	1.701	0.065	1.912	0.019	-1.137	0.349	PA14_42530	39.119	-0.057	0.985
PA14_42540	14.086	2.115	0.003	1.111	0.121	-1.487	0.176	PA14_42540	68.630	0.091	0.883
PA14_42550	103.194	2.514	0.000	2.553	0.000	-1.638	0.018	PA14_42550	221.652	1.081	0.000
PA14_42570	170.713	2.361	0.000	2.042	0.000	-2.084	0.000	PA14_42570	251.327	0.114	0.785
PA14_42580	18.259	0.759	0.325	0.486	0.484	-2.281	0.019	PA14_42580	62.565	-1.551	0.000
PA14_42600	51.064	1.703	0.000	0.783	0.072	-2.185	0.004	PA14_42600	128.531	-1.277	0.001
PA14_42610	82.389	1.216	0.001	0.618	0.099	-1.343	0.017	PA14_42610	138.987	-0.288	0.401
PA14_42620	126.006	1.488	0.000	1.497	0.000	-0.564	0.296	PA14_42620	181.649	-0.200	0.553
PA14_42630	54.211	1.618	0.000	0.975	0.022	-1.534	0.031	PA14_42630	115.118	-0.785	0.183
PA14_42640	52.240	1.669	0.000	1.887	0.000	-0.029	0.972	PA14_42640	92.642	0.045	0.961
PA14_42660	113.419	1.679	0.000	1.560	0.000	-0.776	0.170	PA14_42660	185.722	1.005	0.013
PA14_42670	1667.965	-0.549	0.109	-0.651	0.034	0.037	0.942	PA14_42670	929.698	0.852	0.000
PA14_42680	2233.953	-0.945	0.058	-0.615	0.195	1.507	0.006	PA14_42680	1742.046	0.624	0.146
PA14_42690	529.572	0.047	0.914	0.047	0.887	-0.323	0.403	PA14_42690	541.849	0.344	0.179
PA14_42700	120.865	0.568	0.166	0.416	0.266	0.416	0.417	PA14_42700	383.427	0.297	0.296
PA14_42710	43.162	0.404	0.497	0.648	0.161	0.329	0.642	PA14_42710	233.095	-1.039	0.000
PA14_42720	342.825	0.087	0.848	0.482	0.113	-0.549	0.185	PA14_42720	776.490	1.334	0.000
PA14_42730	468.518	-0.661	0.058	-0.813	0.009	-0.860	0.033	PA14_42730	1326.978	-0.325	0.359
PA14_42740	956.512	-0.457	0.121	-0.102	0.738	-0.956	0.003	PA14_42740	1584.034	0.092	0.759
PA14_42750	224.000	0.781	0.026	1.137	0.000	0.481	0.285	PA14_42750	423.029	1.039	0.000
PA14_42760	418.571	0.161	0.695	0.292	0.354	-0.475	0.246	PA14_42760	994.225	1.115	0.000
PA14_42770	362.937	1.178	0.006	1.456	0.000	2.012	0.000	PA14_42770	1238.451	0.324	0.201
PA14_42780	370.831	-0.617	0.045	-1.119	0.000	-0.230	0.580	PA14_42780	2175.150	-0.686	0.001
PA14_42790	207.200	0.084	0.865	0.753	0.016	0.244	0.612	PA14_42790	351.250	0.598	0.022
PA14_42820	1880.311	0.795	0.007	-0.528	0.064	-1.502	0.000	PA14_42820	5674.964	-1.791	0.000
PA14_42830	103.051	0.420	0.324	0.838	0.013	0.019	0.974	PA14_42830	350.506	-0.733	0.118
PA14_42840	138.283	0.219	0.693	0.649	0.104	0.188	0.760	PA14_42840	422.542	1.097	0.185
PA14_42850	914.348	0.337	0.316	1.374	0.000	-0.201	0.633	PA14_42850	2051.564	-0.116	0.666
PA14_42860	17776.260	-1.087	0.002	-1.953	0.000	-4.461	0.000	PA14_42860	92844.167	0.088	0.704
PA14_42870	306.129	0.466	0.261	0.925	0.006	0.169	0.755	PA14_42870	757.927	-0.253	0.309
PA14_42880	31.054	-1.017	0.085	-1.507	0.004	-0.981	0.173	PA14_42880	120.616	-0.463	0.194
PA14_42890	41.179	-2.168	0.000	-1.581	0.002	-1.398	0.048	PA14_42890	231.217	-1.223	0.000
PA14_42900	307.830	-2.116	0.000	-2.271	0.000	-1.336	0.000	PA14_42900	916.373	-0.369	0.109
PA14_42910	135.158	-2.033	0.000	-2.613	0.000	-1.185	0.006	PA14_42910	428.641	-0.700	0.004
PA14_42920	423.160	-1.846	0.000	-2.695	0.000	-1.274	0.000	PA14_42920	1224.426	-1.263	0.000
PA14_42940	410.394	-2.030	0.000	-2.823	0.000	-1.257	0.005	PA14_42940	841.472	-0.776	0.001
PA14_42950	309.744	-2.502	0.000	-3.951	0.000	-2.161	0.000	PA14_42950	583.860	-0.378	0.124
PA14_42960	18.049	-2.884	0.000	-4.058	0.000	-2.077	0.015	PA14_42960	81.947	0.161	0.679
PA14_42970	241.456	-1.597	0.000	-2.955	0.000	-0.383	0.401	PA14_42970	837.612	-0.984	0.001
PA14_42980	565.836	-1.875	0.000	-3.222	0.000	-1.398	0.000	PA14_42980	1410.600	-0.517	0.021
PA14_42990	369.737	-2.249	0.000	-3.471	0.000	-1.413	0.000	PA14_42990	1384.505	0.147	0.545
PA14_43000	732.344	-1.487	0.000	-2.505	0.000	-0.427	0.307	PA14_43000	2856.526	0.298	0.183

PA14_43020	152.460	-2.195	0.000	-2.900	0.000	-1.001	0.013	PA14_43020	670.109	0.384	0.105
PA14_43030	1816.311	-1.648	0.000	-2.446	0.000	-0.905	0.002	PA14_43030	5536.857	1.216	0.000
PA14_43040	1964.949	-1.622	0.000	-1.987	0.000	-0.912	0.008	PA14_43040	4699.000	1.112	0.000
PA14_43050	8113.151	-0.615	0.048	-0.973	0.000	-0.187	0.665	PA14_43050	17333.644	0.880	0.000
PA14_43070	113.972	-1.957	0.000	-3.734	0.000	-1.983	0.000	PA14_43070	1378.111	-1.560	0.000
PA14_43080	5.284	-0.378	0.789	-0.489	0.655	0.014	0.992	PA14_43080	174.380	0.156	0.657
PA14_43090	56.410	-1.122	0.015	-2.044	0.000	-0.226	0.727	PA14_43090	243.804	0.283	0.322
PA14_43100	6024.116	-0.377	0.468	-0.090	0.855	1.348	0.006	PA14_43100	11258.102	-1.380	0.000
PA14_43110	1190.134	1.289	0.000	1.358	0.000	-2.002	0.000	PA14_43110	2583.321	0.530	0.019
PA14_43130	1237.581	1.638	0.000	1.341	0.000	-1.452	0.000	PA14_43130	3149.197	0.555	0.009
PA14_43140	64.813	1.912	0.000	1.618	0.000	0.572	0.372	PA14_43140	191.475	0.173	0.702
PA14_43150	40.340	-0.350	0.589	-0.475	0.355	-1.322	0.056	PA14_43150	109.577	0.207	0.610
PA14_43160	285.373	1.167	0.000	1.613	0.000	-0.556	0.190	PA14_43160	687.314	0.340	0.181
PA14_43170	170.944	-1.328	0.001	-0.636	0.085	0.023	0.970	PA14_43170	514.845	-1.028	0.000
PA14_43180	68.319	-0.471	0.428	-0.036	0.950	0.280	0.689	PA14_43180	97.828	-0.325	0.576
PA14_43190	129.034	-0.582	0.130	-0.716	0.031	0.460	0.307	PA14_43190	293.014	0.223	0.430
PA14_43200	242.614	-1.129	0.005	-1.617	0.000	-1.936	0.000	PA14_43200	965.570	0.655	0.004
PA14_43220	215.386	1.358	0.000	1.048	0.000	-0.228	0.647	PA14_43220	381.130	0.235	0.386
PA14_43230	73.165	-0.394	0.427	-0.609	0.126	-0.396	0.495	PA14_43230	306.131	0.262	0.345
PA14_43240	148.510	0.066	0.908	0.469	0.197	-0.138	0.813	PA14_43240	296.558	0.847	0.001
PA14_43250	123.788	-2.502	0.000	-3.347	0.000	-0.678	0.156	PA14_43250	824.319	-3.390	0.000
PA14_43270	368.852	-0.145	0.740	0.092	0.800	-0.731	0.073	PA14_43270	410.802	0.759	0.004
PA14_43280	420.932	0.036	0.935	0.437	0.103	-0.487	0.183	PA14_43280	752.248	0.294	0.241
PA14_43290	249.058	-1.362	0.001	-1.091	0.003	-0.920	0.061	PA14_43290	913.174	-0.709	0.019
PA14_43300	454.752	0.505	0.114	0.239	0.440	-0.716	0.058	PA14_43300	1026.830	0.688	0.002
PA14_43310	36.023	-0.147	0.855	0.569	0.277	2.076	0.000	PA14_43310	68.683	1.312	0.001
PA14_43320	15.218	-0.076	0.947	0.265	0.738	-1.183	0.236	PA14_43320	57.433	0.357	0.558
PA14_43340	54.523	0.181	0.761	-0.351	0.440	-1.676	0.008	PA14_43340	184.502	-0.137	0.654
PA14_43350	96.903	-0.463	0.288	-1.044	0.003	-1.292	0.008	PA14_43350	259.920	-1.008	0.000
PA14_43370	7.845	-0.387	0.746	0.058	0.955	-0.118	0.928	PA14_43370	13.524	0.660	0.507
PA14_43380	53.070	0.138	0.836	0.429	0.350	1.667	0.001	PA14_43380	69.727	1.116	0.005
PA14_43400	23.110	-0.086	0.929	0.306	0.643	1.425	0.043	PA14_43400	42.861	0.568	0.272
PA14_43405	10.128	0.401	0.736	0.125	0.903	1.544	0.117	PA14_43405	18.262	0.513	0.533
PA14_43420	292.760	-0.297	0.413	-0.627	0.029	0.658	0.072	PA14_43420	790.312	-1.244	0.000
PA14_43430	426.533	1.514	0.000	1.133	0.001	-0.695	0.161	PA14_43430	611.543	0.543	0.022
PA14_43440	38.413	1.581	0.002	0.754	0.144	-0.504	0.552	PA14_43440	64.773	1.342	0.001
PA14_43460	45.316	0.718	0.199	0.849	0.075	-0.099	0.907	PA14_43460	102.207	1.459	0.000
PA14_43480	413.153	1.832	0.000	2.051	0.000	0.827	0.083	PA14_43480	590.789	0.528	0.173
PA14_43490	88.986	2.031	0.000	2.684	0.000	0.089	0.905	PA14_43490	159.426	1.491	0.000
PA14_43510	338.829	-0.575	0.055	-0.180	0.548	-0.047	0.916	PA14_43510	1376.915	-1.026	0.000
PA14_43520	220.998	-0.171	0.682	0.053	0.882	-0.087	0.861	PA14_43520	809.820	-0.654	0.004
PA14_43530	1101.181	0.126	0.742	0.239	0.414	0.395	0.284	PA14_43530	3920.514	-0.900	0.000
PA14_43540	68.339	-0.320	0.557	-0.429	0.324	-0.089	0.896	PA14_43540	249.134	-0.462	0.094
PA14_43550	111.361	-0.776	0.048	-0.850	0.015	-0.179	0.746	PA14_43550	470.541	-1.561	0.000
PA14_43570	35.058	0.389	0.569	1.866	0.000	-0.751	0.364	PA14_43570	59.803	1.175	0.006
PA14_43580	291.719	0.678	0.081	1.598	0.000	-1.078	0.023	PA14_43580	561.907	-0.412	0.325
PA14_43600	156.389	-0.304	0.587	-0.154	0.752	-1.369	0.013	PA14_43600	384.230	-0.514	0.046
PA14_43610	529.015	-0.804	0.004	-0.335	0.229	0.561	0.103	PA14_43610	1805.545	-1.529	0.000
PA14_43620	142.464	0.125	0.782	0.303	0.354	-0.237	0.626	PA14_43620	347.279	0.420	0.114
PA14_43630	313.406	-0.058	0.893	-0.432	0.121	-0.714	0.049	PA14_43630	849.320	-0.071	0.811
PA14_43640	1373.771	0.145	0.704	-0.085	0.793	0.215	0.597	PA14_43640	3849.477	-0.480	0.027
PA14_43650	232.366	0.150	0.740	0.024	0.951	-0.159	0.752	PA14_43650	416.081	0.481	0.061
PA14_43660	48.747	-0.326	0.614	-1.231	0.011	-0.729	0.281	PA14_43660	91.028	0.182	0.756
PA14_43670	1034.991	-0.024	0.950	-0.142	0.599	0.469	0.137	PA14_43670	2055.719	-0.101	0.675
PA14_43680	1764.102	-1.235	0.000	-1.211	0.000	-0.290	0.364	PA14_43680	3821.178	0.249	0.262
PA14_43690	4278.060	-1.033	0.000	-1.678	0.000	-0.814	0.011	PA14_43690	8249.765	0.260	0.320
PA14_43710	426.533	0.148	0.692	-0.498	0.066	0.789	0.017	PA14_43710	1185.459	-0.657	0.003
PA14_43720	186.817	1.275	0.001	1.648	0.000	0.481	0.385	PA14_43720	347.472	-0.318	0.368
PA14_43730	34.744	-0.837	0.158	-0.418	0.436	0.127	0.874	PA14_43730	310.232	-3.476	0.000

PA14_43740	343.223	-0.865	0.015	-0.773	0.019	0.350	0.455	PA14_43740	717.426	-1.333	0.000
PA14_43760	788.365	-2.232	0.000	-3.376	0.000	-3.105	0.000	PA14_43760	2998.170	-1.748	0.001
PA14_43770	76.753	-0.847	0.082	-0.726	0.094	0.510	0.380	PA14_43770	454.005	-1.422	0.011
PA14_43780	282.045	1.363	0.000	2.146	0.000	2.412	0.000	PA14_43780	1828.216	-0.596	0.007
PA14_43790	522.463	1.174	0.000	1.878	0.000	2.303	0.000	PA14_43790	4176.923	0.403	0.238
PA14_43810	70.753	0.445	0.437	1.014	0.020	1.063	0.059	PA14_43810	966.222	1.003	0.000
PA14_43820	45.427	0.248	0.736	0.830	0.104	-0.318	0.700	PA14_43820	98.731	-0.226	0.602
PA14_43830	32.814	0.156	0.847	0.127	0.840	-1.171	0.131	PA14_43830	73.334	0.531	0.191
PA14_43840	413.995	-1.134	0.000	-0.146	0.606	-2.163	0.000	PA14_43840	647.589	1.574	0.000
PA14_43850	9170.826	-0.137	0.672	0.670	0.004	-3.936	0.000	PA14_43850	21377.731	2.741	0.000
PA14_43870	138.745	0.097	0.857	-0.021	0.963	-0.844	0.083	PA14_43870	240.775	0.985	0.000
PA14_43880	63.862	0.325	0.557	0.434	0.325	-1.244	0.043	PA14_43880	108.753	0.973	0.051
PA14_43890	64.921	0.299	0.618	1.173	0.005	1.079	0.049	PA14_43890	107.846	1.319	0.000
PA14_43900	3686.152	-0.992	0.003	-0.939	0.003	-0.159	0.750	PA14_43900	26680.803	-1.134	0.000
PA14_43910	68.467	0.093	0.892	1.271	0.002	0.844	0.130	PA14_43910	86.888	1.067	0.004
PA14_43920	425.885	-0.418	0.261	0.142	0.689	1.542	0.000	PA14_43920	415.730	1.419	0.000
PA14_43940	4338.586	-0.121	0.761	0.215	0.485	-1.135	0.001	PA14_43940	3636.747	1.086	0.000
PA14_43950	16833.932	-0.245	0.528	-0.112	0.750	-1.210	0.001	PA14_43950	15263.608	1.157	0.000
PA14_43970	5611.022	-0.136	0.749	-0.408	0.191	-1.064	0.004	PA14_43970	6546.920	-0.079	0.762
PA14_44000	10966.253	0.329	0.383	0.217	0.524	-0.531	0.190	PA14_44000	10198.108	-0.385	0.074
PA14_44010	21417.391	0.274	0.422	-0.130	0.680	-0.237	0.549	PA14_44010	22697.393	-0.399	0.070
PA14_44020	5640.856	-0.713	0.034	-1.252	0.000	-1.258	0.001	PA14_44020	5759.307	0.026	0.919
PA14_44030	8437.347	-0.192	0.563	-0.188	0.504	-0.422	0.214	PA14_44030	5735.493	0.846	0.018
PA14_44050	2766.061	-0.445	0.260	-0.231	0.535	-0.205	0.685	PA14_44050	4934.024	1.638	0.000
PA14_44060	3489.359	0.109	0.802	0.681	0.021	0.956	0.009	PA14_44060	6443.348	1.253	0.000
PA14_44070	8856.156	-0.041	0.916	-0.155	0.571	-1.590	0.000	PA14_44070	9264.021	0.484	0.020
PA14_44080	1432.282	-1.588	0.000	-1.610	0.000	-0.694	0.019	PA14_44080	3160.952	0.275	0.219
PA14_44090	201.636	0.610	0.165	1.596	0.000	2.356	0.000	PA14_44090	188.320	0.352	0.265
PA14_44100	128.024	-0.275	0.557	0.417	0.248	0.110	0.849	PA14_44100	279.351	0.783	0.003
PA14_44110	77.649	0.914	0.048	1.780	0.000	2.431	0.000	PA14_44110	91.274	1.477	0.000
PA14_44120	415.350	-0.437	0.233	-0.135	0.704	0.641	0.107	PA14_44120	521.852	0.404	0.116
PA14_44130	981.010	-0.222	0.531	-0.208	0.486	0.603	0.085	PA14_44130	4552.729	-2.495	0.000
PA14_44140	153.926	-1.154	0.037	-1.924	0.000	-2.687	0.000	PA14_44140	437.087	0.099	0.852
PA14_44150	236.359	0.116	0.785	0.414	0.162	0.810	0.026	PA14_44150	707.334	0.655	0.005
PA14_44160	1068.450	-0.321	0.331	-0.339	0.233	0.246	0.531	PA14_44160	3080.067	0.299	0.187
PA14_44170	252.041	-1.077	0.004	-1.724	0.000	-0.091	0.874	PA14_44170	617.151	-1.772	0.000
PA14_44180	189.797	0.714	0.042	1.376	0.000	1.420	0.000	PA14_44180	249.568	1.575	0.000
PA14_44190	12.493	0.166	0.885	0.655	0.387	-0.151	0.900	PA14_44190	21.404	1.154	0.138
PA14_44200	2.988	-0.160	0.928	-0.172	0.899	0.510	0.725	PA14_44200	4.383	1.544	0.370
PA14_44210	44.005	-0.071	0.926	0.186	0.723	1.907	0.000	PA14_44210	91.357	-0.391	0.333
PA14_44230	195.270	0.236	0.661	-0.875	0.028	0.766	0.134	PA14_44230	2651.065	-4.210	0.000
PA14_44240	43.484	-0.157	0.805	-1.148	0.008	-0.562	0.374	PA14_44240	226.857	-2.323	0.000
PA14_44260	88.540	-0.184	0.707	-0.431	0.237	-0.488	0.336	PA14_44260	833.607	-3.173	0.000
PA14_44270	291.050	-0.480	0.251	0.059	0.890	-0.041	0.944	PA14_44270	1125.589	-1.110	0.000
PA14_44280	125.869	-0.353	0.513	-0.145	0.764	-0.954	0.087	PA14_44280	471.128	1.041	0.000
PA14_44290	2783.707	-1.427	0.000	-1.853	0.000	-0.624	0.039	PA14_44290	11870.283	-1.471	0.000
PA14_44300	4271.900	-0.589	0.094	-1.772	0.000	-1.754	0.000	PA14_44300	18428.984	-0.336	0.137
PA14_44311	321.536	-2.278	0.000	-2.830	0.000	0.295	0.498	PA14_44311	579.552	1.217	0.030
PA14_44320	38.114	0.412	0.503	0.175	0.751	0.454	0.515	PA14_44320	78.311	0.433	0.294
PA14_44340	5666.228	-1.507	0.000	-2.933	0.000	-4.418	0.000	PA14_44340	15179.726	1.392	0.000
PA14_44350	1558.728	-1.320	0.000	-2.660	0.000	-4.311	0.000	PA14_44350	4891.274	1.060	0.000
PA14_44360	2707.766	-1.420	0.000	-2.908	0.000	-5.214	0.000	PA14_44360	8191.006	1.038	0.000
PA14_44370	6697.876	-0.514	0.099	-1.034	0.000	-0.296	0.456	PA14_44370	4331.699	-0.132	0.604
PA14_44380	946.895	-0.630	0.054	-1.290	0.000	-1.318	0.000	PA14_44380	943.008	-0.569	0.018
PA14_44390	253.537	-0.177	0.767	-1.200	0.004	-1.967	0.000	PA14_44390	575.397	-0.311	0.457
PA14_44400	2355.587	-0.778	0.007	-1.507	0.000	-1.971	0.000	PA14_44400	1165.428	-0.627	0.014
PA14_44420	6771.666	-1.346	0.000	-2.083	0.000	-0.073	0.878	PA14_44420	10201.354	-0.920	0.000
PA14_44430	1150.178	-1.099	0.000	-1.444	0.000	-0.851	0.018	PA14_44430	2521.403	-0.033	0.866
PA14_44440	759.682	-1.111	0.000	-1.322	0.000	-0.022	0.964	PA14_44440	1916.805	0.600	0.006

PA14_44450	28.637	0.268	0.773	0.960	0.135	0.009	0.993	PA14_44450	158.762	-0.477	0.130
PA14_44460	44.509	-1.004	0.074	-0.745	0.138	-0.150	0.850	PA14_44460	94.799	0.261	0.510
PA14_44470	9931.441	-0.440	0.071	-1.270	0.000	-0.935	0.000	PA14_44470	29788.316	0.768	0.000
PA14_44480	4250.949	-0.584	0.104	-0.912	0.004	0.207	0.669	PA14_44480	16108.426	-2.377	0.000
PA14_44490	5943.864	-1.040	0.000	-1.026	0.000	0.764	0.006	PA14_44490	11952.463	-0.640	0.002
PA14_44500	199.459	-0.673	0.185	-0.324	0.501	0.183	0.787	PA14_44500	419.303	0.579	0.019
PA14_44510	335.319	-0.242	0.545	0.526	0.082	1.232	0.001	PA14_44510	1089.348	-0.822	0.000
PA14_44520	93.263	1.578	0.001	1.941	0.000	3.601	0.000	PA14_44520	85.318	1.160	0.002
PA14_44530	161.014	3.154	0.000	4.054	0.000	5.888	0.000	PA14_44530	84.005	2.364	0.003
PA14_44540	182.137	0.397	0.319	1.057	0.001	0.913	0.025	PA14_44540	390.948	-0.125	0.679
PA14_44560	291.150	0.170	0.676	0.510	0.082	2.004	0.000	PA14_44560	415.118	1.060	0.000
PA14_44570	90.392	-0.627	0.157	-1.114	0.003	0.365	0.500	PA14_44570	367.337	-0.950	0.000
PA14_44580	72.072	-0.241	0.660	-0.755	0.063	-0.124	0.851	PA14_44580	200.227	0.385	0.201
PA14_44590	558.236	0.760	0.023	2.250	0.000	0.691	0.088	PA14_44590	550.516	0.277	0.296
PA14_44610	281.566	0.920	0.005	1.940	0.000	0.500	0.245	PA14_44610	174.307	0.552	0.136
PA14_44620	1696.756	-0.035	0.943	-0.415	0.182	-1.622	0.000	PA14_44620	2373.254	0.010	0.969
PA14_44630	3714.171	0.400	0.185	0.547	0.038	-0.198	0.613	PA14_44630	5987.875	0.454	0.039
PA14_44640	98.687	-0.495	0.235	-1.277	0.000	-0.692	0.152	PA14_44640	363.480	-0.774	0.002
PA14_44650	658.199	-0.649	0.039	-0.198	0.540	-0.841	0.022	PA14_44650	1108.886	0.551	0.016
PA14_44660	1857.602	0.535	0.073	0.766	0.004	0.398	0.280	PA14_44660	3038.019	0.763	0.002
PA14_44670	10606.417	0.133	0.754	0.355	0.265	-0.016	0.973	PA14_44670	20571.381	-0.862	0.000
PA14_44680	5341.164	-0.152	0.718	0.403	0.200	0.858	0.022	PA14_44680	10800.720	-0.498	0.253
PA14_44690	1564.111	0.361	0.405	0.519	0.148	0.725	0.106	PA14_44690	4587.256	0.185	0.514
PA14_44700	139.267	-0.479	0.338	-0.821	0.045	-0.567	0.316	PA14_44700	190.559	0.823	0.005
PA14_44710	251.495	0.138	0.740	-1.195	0.000	0.823	0.024	PA14_44710	1028.335	-0.422	0.079
PA14_44740	437.523	0.480	0.115	-1.281	0.000	1.788	0.000	PA14_44740	2204.355	0.134	0.554
PA14_44760	151.743	0.490	0.255	0.726	0.041	2.540	0.000	PA14_44760	469.628	0.523	0.035
PA14_44770	156.686	0.197	0.666	-0.016	0.969	1.851	0.000	PA14_44770	722.158	0.556	0.017
PA14_44780	658.006	0.165	0.747	-0.292	0.459	-0.636	0.185	PA14_44780	2170.359	-0.519	0.120
PA14_44800	121.011	2.901	0.000	1.011	0.004	1.336	0.002	PA14_44800	128.818	0.729	0.042
PA14_44820	1667.491	4.632	0.000	1.478	0.000	0.873	0.032	PA14_44820	721.441	-0.004	0.962
PA14_44830	2393.192	4.497	0.000	1.898	0.000	1.583	0.000	PA14_44830	1608.318	0.157	0.497
PA14_44840	504.088	4.607	0.000	1.719	0.000	1.187	0.008	PA14_44840	666.187	0.451	0.056
PA14_44850	3158.941	4.912	0.000	2.417	0.000	1.944	0.000	PA14_44850	3334.925	0.718	0.001
PA14_44860	7522.034	6.231	0.000	2.803	0.000	1.877	0.000	PA14_44860	4320.610	1.993	0.000
PA14_44880	7440.960	6.055	0.000	2.611	0.000	1.339	0.000	PA14_44880	3473.709	2.748	0.000
PA14_44890	6.817	2.098	0.034	0.230	0.841	0.447	0.742	PA14_44890	686.680	-1.765	0.000
PA14_44900	119.031	-0.381	0.358	-0.739	0.027	0.138	0.793	PA14_44900	806.902	-0.625	0.006
PA14_44910	2168.577	0.022	0.966	0.017	0.963	-0.089	0.853	PA14_44910	8599.884	-0.687	0.001
PA14_44920	2489.552	-0.215	0.609	-0.295	0.387	-0.771	0.055	PA14_44920	10171.860	-0.390	0.076
PA14_44930	45.419	-0.327	0.648	-0.623	0.259	0.012	0.989	PA14_44930	258.372	0.052	0.852
PA14_44950	187.594	3.081	0.000	0.550	0.192	0.219	0.737	PA14_44950	144.018	1.048	0.001
PA14_44960	102.823	0.620	0.139	1.963	0.000	0.352	0.529	PA14_44960	314.489	1.679	0.000
PA14_44970	1198.763	0.267	0.358	0.492	0.037	-0.953	0.001	PA14_44970	3770.229	-0.088	0.693
PA14_44980	287.030	-0.008	0.987	0.638	0.051	0.439	0.334	PA14_44980	769.788	1.766	0.000
PA14_44990	277.619	5.211	0.000	1.743	0.000	0.209	0.784	PA14_44990	67.141	0.356	0.438
PA14_45000	2420.470	7.677	0.000	2.205	0.000	1.130	0.011	PA14_45000	102.799	1.277	0.000
PA14_45010	8794.387	8.515	0.000	2.361	0.000	2.199	0.000	PA14_45010	171.068	0.238	0.441
PA14_45020	3877.323	8.809	0.000	1.984	0.000	1.006	0.017	PA14_45020	77.416	0.031	0.931
PA14_45030	2052.169	8.137	0.000	2.032	0.000	-0.016	0.981	PA14_45030	68.638	1.580	0.000
PA14_45050	1056.717	7.373	0.000	1.581	0.000	0.187	0.789	PA14_45050	62.114	1.980	0.000
PA14_45060	99.327	6.062	0.000	1.010	0.101	1.612	0.029	PA14_45060	17.213	1.527	0.049
PA14_45070	176.016	1.696	0.000	0.536	0.168	0.200	0.739	PA14_45070	303.879	0.146	0.611
PA14_45090	67.769	1.643	0.000	1.333	0.001	1.243	0.018	PA14_45090	86.881	0.043	0.928
PA14_45100	261.446	0.667	0.029	0.153	0.632	1.966	0.000	PA14_45100	329.530	-0.065	0.887
PA14_45110	1652.307	-0.956	0.001	-1.038	0.000	-1.838	0.000	PA14_45110	2883.971	0.365	0.101
PA14_45120	115.855	0.224	0.749	0.890	0.069	0.690	0.294	PA14_45120	193.431	-0.283	0.344
PA14_45130	24.339	0.933	0.175	1.179	0.043	1.622	0.022	PA14_45130	58.953	0.780	0.069
PA14_45150	180.756	1.139	0.001	1.257	0.000	1.079	0.012	PA14_45150	315.908	1.276	0.000

PA14_45170	100.406	0.797	0.035	1.220	0.000	-0.241	0.677	PA14_45170	130.299	0.943	0.003
PA14_45180	97.393	0.789	0.112	0.743	0.095	0.355	0.589	PA14_45180	102.488	0.053	0.930
PA14_45190	48.028	0.975	0.063	0.853	0.073	0.405	0.573	PA14_45190	85.902	0.198	0.658
PA14_45210	24.525	-0.168	0.844	-1.194	0.042	-1.848	0.026	PA14_45210	54.819	-0.396	0.434
PA14_45240	17.166	-0.189	0.843	-0.196	0.788	0.157	0.874	PA14_45240	43.622	-0.893	0.071
PA14_45250	78.276	-0.052	0.931	0.051	0.911	0.010	0.987	PA14_45250	282.302	0.006	1.000
PA14_45260	80.791	-0.993	0.020	-0.118	0.791	-0.857	0.107	PA14_45260	178.351	-0.938	0.123
PA14_45280	967.379	-0.639	0.037	-0.172	0.588	-1.352	0.000	PA14_45280	874.744	0.082	0.877
PA14_45290	261.357	-0.953	0.001	-0.681	0.013	-1.834	0.000	PA14_45290	352.177	-0.534	0.089
PA14_45300	533.075	-0.904	0.001	-1.203	0.000	-1.455	0.000	PA14_45300	672.030	-0.649	0.045
PA14_45310	1058.070	-1.473	0.000	-1.609	0.000	-1.891	0.000	PA14_45310	977.458	-0.277	0.229
PA14_45330	685.990	-0.924	0.000	-0.935	0.000	-1.429	0.000	PA14_45330	765.362	-0.037	0.898
PA14_45340	141.214	-1.117	0.001	-1.471	0.000	-1.270	0.002	PA14_45340	381.055	0.353	0.188
PA14_45350	917.449	-0.642	0.109	-0.237	0.553	0.446	0.364	PA14_45350	821.314	0.845	0.000
PA14_45370	199.122	-0.501	0.174	0.288	0.388	0.638	0.116	PA14_45370	303.417	0.388	0.162
PA14_45380	128.653	0.321	0.518	1.467	0.000	1.172	0.011	PA14_45380	259.838	1.727	0.000
PA14_45400	429.022	-0.809	0.013	-1.609	0.000	0.528	0.188	PA14_45400	1343.598	-1.174	0.000
PA14_45410	124.904	-0.242	0.639	-0.944	0.013	0.603	0.229	PA14_45410	778.203	-1.279	0.003
PA14_45430	31.640	-1.074	0.092	-1.243	0.026	0.068	0.940	PA14_45430	128.255	-1.296	0.000
PA14_45440	34.521	-0.262	0.708	-0.204	0.718	-0.564	0.452	PA14_45440	83.985	0.021	0.995
PA14_45450	48.699	0.094	0.898	0.894	0.045	-0.340	0.648	PA14_45450	109.808	0.468	0.201
PA14_45460	122.855	0.368	0.415	1.128	0.001	0.196	0.732	PA14_45460	256.906	1.408	0.000
PA14_45470	45.383	-0.077	0.926	0.263	0.644	0.146	0.859	PA14_45470	208.646	-1.755	0.000
PA14_45480	174.754	-0.087	0.870	-0.230	0.546	0.264	0.603	PA14_45480	716.396	-0.450	0.069
PA14_45500	2316.430	-0.162	0.593	-0.229	0.350	0.177	0.601	PA14_45500	5430.239	-0.468	0.028
PA14_45510	2478.263	0.105	0.797	0.293	0.322	1.548	0.000	PA14_45510	6123.386	-0.398	0.069
PA14_45520	834.245	-0.404	0.162	-0.449	0.078	0.223	0.543	PA14_45520	1731.295	0.312	0.179
PA14_45540	1118.395	-0.259	0.446	-0.310	0.277	0.089	0.840	PA14_45540	2878.905	-0.002	0.969
PA14_45560	843.419	0.057	0.880	0.213	0.416	0.452	0.159	PA14_45560	1803.972	0.384	0.088
PA14_45580	760.058	-0.360	0.217	-0.436	0.084	-0.756	0.017	PA14_45580	1879.471	0.208	0.398
PA14_45590	7249.655	0.077	0.832	0.163	0.544	-0.031	0.937	PA14_45590	14921.998	0.748	0.000
PA14_45610	3841.571	-0.092	0.799	-0.035	0.910	0.176	0.636	PA14_45610	12361.020	0.718	0.000
PA14_45620	3942.833	-0.414	0.303	-1.095	0.001	-1.195	0.003	PA14_45620	10453.295	0.361	0.107
PA14_45630	5005.640	-0.112	0.740	-0.187	0.474	-0.471	0.132	PA14_45630	14441.550	-0.832	0.000
PA14_45640	2267.553	0.115	0.719	0.066	0.811	0.728	0.010	PA14_45640	5051.002	-0.404	0.066
PA14_45660	4241.352	0.283	0.495	0.430	0.204	1.312	0.001	PA14_45660	6135.363	-0.243	0.277
PA14_45680	1212.864	0.419	0.121	1.147	0.000	1.075	0.000	PA14_45680	1836.959	-0.081	0.735
PA14_45700	63.586	-0.274	0.628	0.200	0.663	0.026	0.971	PA14_45700	149.752	0.132	0.731
PA14_45710	191.174	-0.132	0.799	0.335	0.368	0.135	0.810	PA14_45710	426.934	0.440	0.097
PA14_45720	348.722	-0.094	0.832	0.629	0.027	0.176	0.693	PA14_45720	735.163	0.710	0.002
PA14_45740	194.934	-0.054	0.913	-0.201	0.556	-1.151	0.005	PA14_45740	671.067	0.146	0.646
PA14_45760	98.564	-0.334	0.520	-0.712	0.077	-0.500	0.381	PA14_45760	352.520	-0.196	0.746
PA14_45770	583.786	-0.073	0.865	0.091	0.783	-0.872	0.015	PA14_45770	1130.789	0.814	0.000
PA14_45780	96.904	0.197	0.717	0.595	0.123	-0.088	0.892	PA14_45780	251.445	0.298	0.331
PA14_45790	273.982	-0.185	0.607	-0.421	0.125	-1.022	0.003	PA14_45790	735.448	-0.673	0.003
PA14_45800	2768.126	-0.312	0.457	-0.007	0.987	0.344	0.465	PA14_45800	5250.276	-0.594	0.005
PA14_45810	614.914	-0.582	0.041	-0.511	0.051	-0.190	0.626	PA14_45810	2344.779	-0.738	0.001
PA14_45830	658.516	-0.158	0.761	0.261	0.519	-0.407	0.429	PA14_45830	1595.896	-0.331	0.164
PA14_45840	5729.228	0.012	0.975	0.621	0.008	1.094	0.000	PA14_45840	9600.571	0.175	0.479
PA14_45850	202.520	0.975	0.002	1.975	0.000	1.986	0.000	PA14_45850	356.330	0.974	0.000
PA14_45870	81.826	-0.029	0.966	-0.296	0.490	-0.537	0.351	PA14_45870	462.471	-0.426	0.091
PA14_45880	68.814	-0.478	0.368	-0.192	0.689	-0.226	0.735	PA14_45880	416.855	-1.428	0.000
PA14_45890	104.640	-0.636	0.156	0.360	0.368	1.058	0.022	PA14_45890	242.195	-0.281	0.339
PA14_45910	110.338	-0.647	0.197	1.203	0.003	0.342	0.578	PA14_45910	146.703	-0.353	0.289
PA14_45920	90.430	-0.208	0.693	-0.204	0.627	0.143	0.812	PA14_45920	307.092	-0.748	0.004
PA14_45930	185.369	-0.767	0.024	-0.946	0.002	-0.915	0.025	PA14_45930	437.752	0.037	0.906
PA14_45940	4156.690	0.360	0.315	0.624	0.036	-1.300	0.000	PA14_45940	10941.867	-0.789	0.000
PA14_45950	10431.089	-1.412	0.000	-2.083	0.000	-2.706	0.000	PA14_45950	45197.169	-1.256	0.000
PA14_45960	3350.921	-0.776	0.005	-0.825	0.001	0.682	0.043	PA14_45960	11254.801	-1.078	0.000

PA14_45970	958.430	-2.550	0.000	-3.264	0.000	-4.269	0.000	PA14_45970	10059.134	-0.255	0.494
PA14_45980	18.288	0.995	0.239	2.113	0.001	2.416	0.002	PA14_45980	49.457	1.836	0.000
PA14_46000	23.921	-0.208	0.834	0.695	0.298	-0.987	0.292	PA14_46000	44.868	0.023	0.984
PA14_46010	46.123	0.784	0.160	1.926	0.000	-0.073	0.934	PA14_46010	60.697	0.327	0.509
PA14_46020	44.747	1.173	0.019	1.339	0.003	0.342	0.647	PA14_46020	97.743	0.208	0.639
PA14_46030	168.087	-0.583	0.105	-0.683	0.031	-0.830	0.049	PA14_46030	893.587	-1.819	0.000
PA14_46060	138.578	-0.415	0.363	0.658	0.068	0.315	0.553	PA14_46060	296.884	-0.457	0.100
PA14_46070	990.479	1.284	0.003	6.107	0.000	3.474	0.000	PA14_46070	77.830	1.666	0.000
PA14_46080	619.468	0.980	0.036	6.574	0.000	4.412	0.000	PA14_46080	65.343	0.584	0.179
PA14_46100	215.631	0.209	0.729	4.891	0.000	2.398	0.000	PA14_46100	43.554	0.974	0.048
PA14_46110	227.046	1.443	0.003	4.925	0.000	1.429	0.013	PA14_46110	55.519	0.794	0.067
PA14_46120	131.262	1.403	0.002	4.705	0.000	0.932	0.129	PA14_46120	53.398	1.072	0.110
PA14_46140	249.976	0.864	0.030	4.344	0.000	1.904	0.000	PA14_46140	135.134	1.776	0.000
PA14_46150	158.185	-1.177	0.002	0.691	0.045	-0.370	0.471	PA14_46150	984.062	-2.718	0.000
PA14_46160	2077.407	-0.469	0.121	-1.008	0.000	0.171	0.674	PA14_46160	16918.072	-1.718	0.003
PA14_46170	194.404	0.387	0.337	1.547	0.000	2.227	0.000	PA14_46170	276.402	0.433	0.134
PA14_46180	27.449	0.296	0.739	2.677	0.000	2.114	0.002	PA14_46180	22.928	-0.327	0.643
PA14_46200	116.643	1.812	0.000	1.093	0.003	0.544	0.320	PA14_46200	174.004	0.197	0.569
PA14_46220	432.304	4.532	0.000	0.178	0.711	0.427	0.478	PA14_46220	112.360	-0.436	0.295
PA14_46230	224.144	4.127	0.000	0.112	0.834	0.663	0.272	PA14_46230	51.511	0.176	0.779
PA14_46240	151.391	-1.475	0.000	-1.576	0.000	-0.245	0.597	PA14_46240	953.881	-1.353	0.000
PA14_46250	24.846	-0.304	0.693	0.318	0.592	-1.076	0.180	PA14_46250	89.715	0.378	0.337
PA14_46260	179.415	-0.068	0.896	-0.069	0.863	0.409	0.375	PA14_46260	552.451	-1.253	0.000
PA14_46270	232.362	0.732	0.025	1.199	0.000	-0.898	0.038	PA14_46270	383.540	1.686	0.000
PA14_46280	624.733	-1.406	0.000	-1.240	0.000	3.246	0.000	PA14_46280	905.079	-3.189	0.000
PA14_46290	60.912	0.890	0.081	1.489	0.000	-0.171	0.833	PA14_46290	133.924	0.938	0.004
PA14_46300	80.436	0.515	0.285	2.099	0.000	0.268	0.674	PA14_46300	114.903	1.001	0.003
PA14_46310	97.066	0.073	0.904	0.638	0.089	-0.264	0.665	PA14_46310	304.138	-0.868	0.001
PA14_46320	154.126	0.395	0.327	1.132	0.000	1.090	0.006	PA14_46320	302.741	0.356	0.215
PA14_46330	65.875	0.385	0.494	0.657	0.135	0.248	0.716	PA14_46330	133.074	0.525	0.119
PA14_46340	114.791	0.261	0.580	0.462	0.205	0.314	0.549	PA14_46340	217.898	0.762	0.008
PA14_46360	179.348	0.937	0.004	1.292	0.000	1.421	0.000	PA14_46360	274.559	0.584	0.027
PA14_46370	78.965	-0.486	0.370	0.167	0.733	0.423	0.489	PA14_46370	207.140	1.015	0.000
PA14_46380	92.827	0.412	0.362	1.016	0.003	0.950	0.038	PA14_46380	146.936	0.916	0.003
PA14_46390	34.585	1.001	0.065	0.924	0.059	-0.041	0.964	PA14_46390	85.837	1.283	0.001
PA14_46400	132.329	0.167	0.720	-0.014	0.973	-0.372	0.454	PA14_46400	432.570	0.071	0.807
PA14_46420	25.080	-0.696	0.377	-0.817	0.211	0.071	0.944	PA14_46420	90.349	0.312	0.422
PA14_46430	171.069	0.252	0.553	0.391	0.241	0.640	0.129	PA14_46430	400.304	-0.175	0.554
PA14_46440	801.271	0.460	0.146	0.396	0.167	0.081	0.859	PA14_46440	1695.228	0.150	0.537
PA14_46450	764.830	-0.427	0.167	-1.386	0.000	-0.656	0.060	PA14_46450	2975.470	-0.030	0.916
PA14_46460	1478.042	0.154	0.635	0.053	0.856	-0.076	0.847	PA14_46460	3218.777	0.997	0.001
PA14_46470	162.775	-0.092	0.852	0.353	0.292	0.606	0.150	PA14_46470	235.521	1.213	0.000
PA14_46480	78.851	1.548	0.000	2.069	0.000	1.602	0.001	PA14_46480	148.772	1.063	0.001
PA14_46490	137.230	1.111	0.002	1.161	0.001	1.308	0.002	PA14_46490	204.688	1.067	0.005
PA14_46510	1485.434	-1.489	0.000	-2.162	0.000	-0.265	0.463	PA14_46510	7175.509	-2.360	0.000
PA14_46520	1103.830	-2.122	0.000	-3.601	0.000	-1.998	0.000	PA14_46520	6240.911	-2.516	0.000
PA14_46530	577.223	-1.924	0.000	-3.040	0.000	-1.324	0.000	PA14_46530	5184.405	-3.726	0.000
PA14_46540	1809.447	-1.324	0.000	-2.382	0.000	-0.973	0.000	PA14_46540	10742.340	-1.801	0.000
PA14_46550	2196.140	-0.837	0.002	-1.901	0.000	-0.151	0.704	PA14_46550	11749.794	-1.239	0.000
PA14_46560	2.193	-0.258	0.884	-0.352	0.793	-0.149	0.924	PA14_46560	6.314	-0.698	0.666
PA14_46570	582.841	0.712	0.009	1.397	0.000	-0.212	0.595	PA14_46570	1852.010	0.825	0.000
PA14_46580	67.325	0.631	0.195	1.648	0.000	0.509	0.398	PA14_46580	114.967	2.018	0.000
PA14_46590	256.310	0.430	0.207	1.066	0.000	0.272	0.529	PA14_46590	387.514	0.302	0.260
PA14_46600	134.885	0.664	0.175	0.767	0.073	0.058	0.935	PA14_46600	151.291	0.661	0.039
PA14_46610	100.163	1.797	0.000	2.642	0.000	0.555	0.375	PA14_46610	212.305	1.512	0.000
PA14_46620	740.875	1.326	0.000	1.895	0.000	0.336	0.460	PA14_46620	1113.886	0.778	0.000
PA14_46630	207.668	0.687	0.054	1.998	0.000	-0.148	0.785	PA14_46630	409.979	1.604	0.000
PA14_46640	241.398	-0.743	0.026	0.438	0.152	-0.873	0.031	PA14_46640	160.660	0.282	0.408
PA14_46650	28.868	-0.211	0.810	-0.577	0.361	-0.172	0.857	PA14_46650	28.629	1.415	0.017

PA14_46660	198.956	0.278	0.549	0.479	0.189	-0.068	0.910	PA14_46660	339.715	0.805	0.005
PA14_46670	22.277	-0.216	0.815	0.007	0.993	0.728	0.369	PA14_46670	109.942	-0.654	0.065
PA14_46680	316.922	0.908	0.002	1.915	0.000	1.165	0.001	PA14_46680	643.158	2.220	0.000
PA14_46700	144.762	1.116	0.005	1.841	0.000	0.767	0.132	PA14_46700	205.287	2.178	0.000
PA14_46710	348.110	0.384	0.238	0.880	0.001	1.434	0.000	PA14_46710	439.371	1.088	0.001
PA14_46720	295.735	0.395	0.325	0.523	0.120	1.919	0.000	PA14_46720	940.711	-1.685	0.000
PA14_46740	173.584	0.070	0.881	-0.739	0.014	-1.209	0.003	PA14_46740	231.337	0.264	0.470
PA14_46750	41.955	-1.750	0.004	-1.836	0.001	2.841	0.000	PA14_46750	82.343	-2.258	0.000
PA14_46760	32.887	-2.045	0.001	-2.509	0.000	2.064	0.000	PA14_46760	136.522	-3.781	0.000
PA14_46770	167.557	-1.371	0.000	-2.585	0.000	0.754	0.064	PA14_46770	209.017	-1.140	0.000
PA14_46780	83.063	-1.507	0.000	-2.315	0.000	0.095	0.882	PA14_46780	222.647	-2.455	0.000
PA14_46800	3.029	0.812	0.570	0.310	0.813	0.967	0.471	PA14_46800	7.456	0.305	0.846
PA14_46810	35.055	-1.011	0.075	-0.930	0.063	1.040	0.079	PA14_46810	106.406	-0.914	0.008
PA14_46820	50.134	-1.223	0.036	-1.433	0.006	1.187	0.056	PA14_46820	175.424	-1.092	0.000
PA14_46830	56.257	-1.058	0.029	-1.383	0.001	1.138	0.026	PA14_46830	180.530	-2.179	0.000
PA14_46840	93.262	-1.124	0.007	-1.822	0.000	-0.869	0.093	PA14_46840	605.803	-2.102	0.000
PA14_46850	113.896	-0.994	0.006	-2.393	0.000	-0.127	0.815	PA14_46850	868.501	-2.430	0.000
PA14_46860	15.235	-0.112	0.914	-0.605	0.388	-0.061	0.954	PA14_46860	86.827	-0.943	0.033
PA14_46880	96.838	-1.154	0.008	-0.165	0.715	0.605	0.248	PA14_46880	758.320	0.176	0.481
PA14_46890	295.630	-0.840	0.009	0.184	0.581	-0.401	0.341	PA14_46890	2164.291	-0.844	0.000
PA14_46900	575.622	-2.671	0.000	-0.037	0.942	-2.499	0.000	PA14_46900	10094.435	-1.021	0.000
PA14_46910	12038.319	-0.469	0.192	-2.417	0.000	-0.428	0.316	PA14_46910	13191.986	-0.847	0.002
PA14_46920	479.461	-0.852	0.031	-1.368	0.000	0.376	0.464	PA14_46920	678.379	-0.318	0.196
PA14_46930	473.713	-1.250	0.000	-2.259	0.000	-0.430	0.335	PA14_46930	907.410	-0.398	0.086
PA14_46950	2098.858	-0.847	0.004	-1.407	0.000	0.387	0.324	PA14_46950	1997.737	-0.355	0.125
PA14_46960	2840.037	-0.253	0.504	-0.904	0.002	0.912	0.012	PA14_46960	1835.951	0.328	0.146
PA14_46970	4347.278	-1.597	0.000	-3.190	0.000	-1.519	0.000	PA14_46970	5537.934	-0.875	0.000
PA14_46980	811.476	0.408	0.170	0.534	0.039	1.646	0.000	PA14_46980	831.398	0.910	0.000
PA14_46990	82.611	-1.101	0.008	-1.432	0.000	-0.112	0.857	PA14_46990	105.033	0.667	0.055
PA14_47000	50.118	-0.313	0.583	-0.644	0.143	-0.626	0.315	PA14_47000	98.569	-0.459	0.233
PA14_47010	28.447	-0.296	0.699	0.189	0.758	-1.020	0.192	PA14_47010	304.836	-3.039	0.000
PA14_47030	6.747	-0.568	0.646	0.577	0.547	0.229	0.863	PA14_47030	29.522	-2.431	0.018
PA14_47040	191.220	-0.975	0.012	0.404	0.264	0.498	0.294	PA14_47040	404.416	2.094	0.000
PA14_47060	27.946	-0.293	0.701	-0.683	0.233	-0.927	0.235	PA14_47060	74.283	-0.506	0.217
PA14_47070	7.769	0.448	0.705	0.809	0.361	1.274	0.219	PA14_47070	19.927	-0.250	0.779
PA14_47080	101.332	-0.314	0.520	0.090	0.837	-0.304	0.593	PA14_47080	404.174	-0.362	0.180
PA14_47090	191.314	-1.582	0.000	-1.634	0.000	0.772	0.051	PA14_47090	1187.734	-3.131	0.000
PA14_47100	102.256	-0.031	0.966	1.102	0.007	0.234	0.722	PA14_47100	124.848	0.771	0.017
PA14_47110	114.531	0.883	0.033	1.633	0.000	0.677	0.190	PA14_47110	249.752	1.223	0.000
PA14_47120	722.494	-2.489	0.000	-3.472	0.000	-0.560	0.278	PA14_47120	3486.041	-2.677	0.000
PA14_47130	1406.976	-2.019	0.000	-2.841	0.000	0.041	0.928	PA14_47130	6287.953	-3.034	0.000
PA14_47140	75.508	-0.430	0.415	-0.259	0.573	0.408	0.487	PA14_47140	177.723	-0.671	0.180
PA14_47150	44.100	0.692	0.256	1.556	0.001	-1.593	0.043	PA14_47150	27.883	0.301	0.671
PA14_47160	127.985	1.077	0.003	1.690	0.000	-0.996	0.054	PA14_47160	120.933	-0.471	0.160
PA14_47180	312.134	0.894	0.002	1.168	0.000	-0.988	0.010	PA14_47180	173.859	-0.225	0.472
PA14_47190	1845.526	1.386	0.000	1.948	0.000	-0.649	0.056	PA14_47190	340.326	0.037	0.896
PA14_47210	1441.183	2.040	0.000	3.101	0.000	0.132	0.776	PA14_47210	177.110	0.372	0.244
PA14_47230	40.419	0.105	0.896	0.262	0.644	-0.667	0.380	PA14_47230	83.535	-0.178	0.744
PA14_47240	209.229	0.197	0.637	0.992	0.001	-0.096	0.852	PA14_47240	628.084	-0.654	0.005
PA14_47250	36.923	-0.244	0.749	0.028	0.966	-0.496	0.533	PA14_47250	128.955	0.169	0.754
PA14_47260	63.278	0.173	0.778	0.858	0.033	1.304	0.009	PA14_47260	124.457	-0.027	0.961
PA14_47270	43.174	-0.482	0.395	-0.893	0.050	-0.487	0.467	PA14_47270	119.275	0.469	0.184
PA14_47280	71.721	0.093	0.888	0.040	0.939	0.343	0.581	PA14_47280	145.971	1.175	0.000
PA14_47300	82.714	0.023	0.973	0.621	0.097	1.054	0.023	PA14_47300	188.229	0.483	0.112
PA14_47310	323.612	1.453	0.001	0.687	0.135	-0.721	0.243	PA14_47310	539.707	1.240	0.000
PA14_47320	155.772	-1.246	0.001	-1.644	0.000	-0.937	0.050	PA14_47320	932.127	-0.665	0.003
PA14_47330	494.463	-0.423	0.152	-0.504	0.051	0.039	0.928	PA14_47330	2306.682	-0.639	0.003
PA14_47340	660.020	0.048	0.949	0.427	0.398	1.384	0.015	PA14_47340	1919.700	-0.528	0.198
PA14_47350	338.490	0.294	0.416	1.133	0.000	1.775	0.000	PA14_47350	613.266	0.683	0.005

PA14_47360	478.306	0.539	0.075	0.541	0.048	0.254	0.527	PA14_47360	583.984	1.277	0.000
PA14_47370	27.107	1.134	0.065	1.656	0.002	0.955	0.200	PA14_47370	31.392	1.157	0.040
PA14_47380	179.707	-0.691	0.150	-0.215	0.642	2.686	0.000	PA14_47380	128.836	-0.356	0.302
PA14_47390	11.172	-0.638	0.595	0.679	0.449	2.561	0.004	PA14_47390	12.026	0.207	0.839
PA14_47400	38.957	0.727	0.357	1.379	0.025	2.740	0.000	PA14_47400	41.551	-0.399	0.452
PA14_47410	44.154	0.334	0.596	1.030	0.023	0.693	0.275	PA14_47410	106.855	0.872	0.014
PA14_47420	33.405	0.390	0.570	-0.396	0.497	-1.317	0.087	PA14_47420	290.730	-1.229	0.000
PA14_47430	61.867	0.302	0.618	0.318	0.520	-0.523	0.440	PA14_47430	351.575	-0.966	0.000
PA14_47440	251.607	0.025	0.963	0.411	0.193	0.561	0.167	PA14_47440	727.147	0.475	0.140
PA14_47450	169.488	0.097	0.838	-0.582	0.070	0.304	0.506	PA14_47450	413.970	-0.401	0.124
PA14_47460	522.566	-0.140	0.684	0.041	0.893	0.979	0.001	PA14_47460	1254.416	0.075	0.800
PA14_47490	786.362	-0.274	0.352	-0.848	0.000	-2.543	0.000	PA14_47490	820.691	1.350	0.000
PA14_47500	582.683	-0.294	0.385	-0.558	0.041	-1.452	0.000	PA14_47500	1409.390	-0.176	0.766
PA14_47510	64.605	-0.208	0.697	-0.710	0.067	-3.117	0.000	PA14_47510	252.059	0.321	0.304
PA14_47520	337.368	0.611	0.045	0.135	0.673	-1.373	0.000	PA14_47520	1635.175	-1.320	0.000
PA14_47530	241.487	0.094	0.845	0.176	0.621	1.185	0.002	PA14_47530	1166.175	-0.931	0.000
PA14_47540	13614.737	1.499	0.000	0.258	0.274	-0.805	0.003	PA14_47540	18673.549	1.049	0.001
PA14_47550	623.949	-1.603	0.000	-2.337	0.000	-1.266	0.000	PA14_47550	1874.329	-1.153	0.000
PA14_47560	14.059	-0.479	0.634	-0.042	0.963	-1.740	0.084	PA14_47560	32.690	-0.417	0.500
PA14_47580	39.825	-1.001	0.098	-0.947	0.076	-2.591	0.001	PA14_47580	202.140	-0.250	0.393
PA14_47600	66.852	-0.803	0.076	-0.942	0.018	0.403	0.470	PA14_47600	387.781	-0.360	0.439
PA14_47610	152.268	-0.269	0.506	0.090	0.804	0.686	0.083	PA14_47610	4848.772	-0.768	0.000
PA14_47640	51.798	0.432	0.500	1.827	0.000	2.935	0.000	PA14_47640	1039.025	0.738	0.001
PA14_47650	61.425	0.506	0.387	-0.142	0.800	-1.137	0.094	PA14_47650	153.049	1.742	0.000
PA14_47660	103.993	0.584	0.127	-0.570	0.108	-2.350	0.000	PA14_47660	168.217	1.429	0.000
PA14_47670	121.800	0.221	0.600	-1.423	0.000	-3.011	0.000	PA14_47670	199.112	1.287	0.000
PA14_47680	95.625	0.216	0.649	-1.143	0.001	-2.267	0.000	PA14_47680	162.464	1.295	0.000
PA14_47690	344.052	0.710	0.013	-0.759	0.005	-1.898	0.000	PA14_47690	306.302	2.086	0.000
PA14_47720	157.693	0.859	0.006	-0.823	0.007	-3.096	0.000	PA14_47720	172.169	1.885	0.000
PA14_47730	108.136	1.035	0.003	-0.219	0.577	-1.520	0.004	PA14_47730	162.398	2.073	0.000
PA14_47750	77.147	0.995	0.024	-0.714	0.102	-1.508	0.015	PA14_47750	88.258	1.359	0.000
PA14_47760	140.266	0.898	0.009	-0.635	0.062	-1.697	0.000	PA14_47760	165.765	1.524	0.000
PA14_47790	420.810	0.256	0.459	-1.843	0.000	-2.673	0.000	PA14_47790	586.518	0.610	0.011
PA14_47800	1439.644	0.964	0.000	-0.386	0.149	-1.028	0.001	PA14_47800	1276.597	1.121	0.000
PA14_47810	67.702	3.847	0.000	4.681	0.000	1.224	0.134	PA14_47810	55.234	0.640	0.181
PA14_47820	233.847	0.348	0.331	0.930	0.001	0.556	0.153	PA14_47820	733.585	0.314	0.195
PA14_47840	12.567	0.153	0.889	0.135	0.871	-1.433	0.157	PA14_47840	23.627	0.803	0.229
PA14_47850	16.675	0.557	0.503	0.095	0.905	-0.420	0.681	PA14_47850	12.990	0.681	0.480
PA14_47860	9.576	0.831	0.405	-0.034	0.973	-0.322	0.798	PA14_47860	10.072	1.205	0.268
PA14_47870	12.977	1.357	0.154	2.806	0.000	0.603	0.624	PA14_47870	19.532	1.340	0.064
PA14_47880	81.733	-0.486	0.284	-0.697	0.064	-0.788	0.131	PA14_47880	271.441	-0.204	0.493
PA14_47890	1151.450	1.692	0.000	2.321	0.000	-1.034	0.004	PA14_47890	2351.442	2.338	0.000
PA14_47900	3.586	0.606	0.683	1.859	0.077	0.214	0.889	PA14_47900	7.660	0.374	0.792
PA14_47910	16.530	-0.405	0.644	-0.407	0.565	-0.180	0.862	PA14_47910	44.094	-0.317	0.553
PA14_47920	30.643	1.747	0.011	0.405	0.586	-0.170	0.877	PA14_47920	39.525	0.663	0.305
PA14_47930	3.834	0.587	0.672	-0.380	0.752	-0.417	0.769	PA14_47930	11.732	0.723	0.476
PA14_47940	2.732	1.237	0.357	-0.151	0.913	0.136	0.931	PA14_47940	5.951	0.481	0.779
PA14_47950	7.518	0.291	0.810	-0.847	0.350	-1.442	0.200	PA14_47950	14.762	-0.172	0.892
PA14_47960	22.138	0.124	0.890	-0.841	0.169	0.434	0.601	PA14_47960	41.167	0.129	0.836
PA14_47970	35.803	1.212	0.016	-0.706	0.180	-0.599	0.432	PA14_47970	66.104	-0.346	0.428
PA14_48000	25.393	0.623	0.365	0.040	0.955	0.812	0.276	PA14_48000	37.182	0.213	0.730
PA14_48010	13.852	-0.085	0.943	0.625	0.409	0.427	0.687	PA14_48010	43.347	0.076	0.919
PA14_48020	130.959	0.529	0.150	0.245	0.485	-0.613	0.191	PA14_48020	288.471	-0.151	0.616
PA14_48030	18.564	-1.042	0.133	-1.501	0.013	-0.560	0.516	PA14_48030	54.077	-0.797	0.274
PA14_48040	606.725	-1.267	0.000	-1.905	0.000	-3.177	0.000	PA14_48040	1339.386	-1.113	0.000
PA14_48060	16932.150	-4.010	0.000	-7.555	0.000	-3.094	0.000	PA14_48060	28788.365	-3.975	0.000
PA14_48090	793.856	-2.136	0.000	-4.426	0.000	-3.606	0.000	PA14_48090	2323.824	-1.683	0.000
PA14_48100	487.615	-2.419	0.000	-4.728	0.000	-3.958	0.000	PA14_48100	1088.572	-1.436	0.000
PA14_48115	398.535	-2.398	0.000	-4.137	0.000	-3.754	0.000	PA14_48115	927.080	-0.381	0.115

PA14_48140	413.849	-2.212	0.000	-3.913	0.000	-1.802	0.000	PA14_48140	1348.750	-0.768	0.000
PA14_48150	186.032	0.473	0.175	0.025	0.949	-0.360	0.423	PA14_48150	356.980	0.635	0.078
PA14_48160	380.700	-1.262	0.000	-1.152	0.000	0.167	0.702	PA14_48160	1377.269	-1.140	0.000
PA14_48170	92.048	-0.223	0.675	0.548	0.146	1.509	0.000	PA14_48170	221.434	-1.577	0.000
PA14_48190	77.295	0.983	0.022	1.567	0.000	1.232	0.012	PA14_48190	168.534	0.087	0.829
PA14_48200	125.046	1.355	0.000	2.101	0.000	0.239	0.667	PA14_48200	63.142	0.161	0.847
PA14_48210	34.928	0.774	0.203	1.462	0.003	1.107	0.094	PA14_48210	47.179	0.552	0.282
PA14_48230	4.869	1.160	0.401	2.684	0.012	1.167	0.378	PA14_48230	1.102	Inf	0.453
PA14_48240	11.013	0.666	0.516	1.813	0.014	1.202	0.224	PA14_48240	15.178	1.668	0.037
PA14_48280	9.553	0.249	0.844	1.756	0.023	0.137	0.917	PA14_48280	27.633	-0.485	0.435
PA14_48300	13.875	-0.060	0.963	1.250	0.075	1.288	0.147	PA14_48300	21.445	1.062	0.136
PA14_48310	57.154	0.277	0.660	0.433	0.375	-1.497	0.028	PA14_48310	165.254	0.274	0.400
PA14_48320	107.126	0.026	0.966	0.121	0.764	-0.175	0.757	PA14_48320	190.878	0.114	0.755
PA14_48330	4.082	-0.018	0.991	-0.279	0.824	-0.183	0.903	PA14_48330	10.213	1.219	0.244
PA14_48340	21.588	-0.238	0.787	0.527	0.394	-0.461	0.619	PA14_48340	38.108	1.181	0.021
PA14_48350	6.593	0.653	0.629	2.171	0.021	1.661	0.151	PA14_48350	20.136	0.513	0.517
PA14_48380	14.649	0.850	0.329	1.912	0.004	1.385	0.114	PA14_48380	23.997	1.538	0.071
PA14_48390	57.892	0.686	0.146	0.443	0.310	-1.395	0.028	PA14_48390	166.368	-0.475	0.128
PA14_48400	91.313	1.181	0.003	2.180	0.000	-2.367	0.001	PA14_48400	77.395	1.925	0.000
PA14_48410	209.339	0.780	0.137	2.954	0.000	2.177	0.000	PA14_48410	155.659	0.638	0.044
PA14_48420	119.289	-0.109	0.849	0.890	0.013	1.398	0.001	PA14_48420	239.723	-0.180	0.607
PA14_48440	1538.539	2.186	0.000	5.205	0.000	5.471	0.000	PA14_48440	215.648	0.767	0.059
PA14_48450	117.994	-0.107	0.871	0.313	0.496	0.399	0.509	PA14_48450	253.934	-0.395	0.165
PA14_48460	102.784	0.658	0.210	0.722	0.113	0.677	0.264	PA14_48460	150.465	0.059	0.865
PA14_48470	11.615	-0.352	0.748	0.575	0.468	-0.488	0.669	PA14_48470	24.202	0.922	0.162
PA14_48490	17.656	0.001	0.999	-0.476	0.541	0.655	0.488	PA14_48490	60.927	0.664	0.130
PA14_48500	443.558	1.990	0.000	1.849	0.000	0.050	0.926	PA14_48500	568.665	1.837	0.000
PA14_48510	5.595	1.038	0.402	1.259	0.218	0.448	0.748	PA14_48510	721.731	0.981	0.000
PA14_48520	378.756	1.334	0.000	1.765	0.000	1.539	0.000	PA14_48520	1229.591	1.163	0.000
PA14_48530	84.782	-0.659	0.116	-2.748	0.000	-0.976	0.047	PA14_48530	116.870	-0.753	0.024
PA14_48540	17.054	-1.460	0.040	-2.945	0.000	-1.225	0.148	PA14_48540	18.773	-2.118	0.003
PA14_48550	7.110	-1.120	0.276	-3.987	0.000	-1.539	0.159	PA14_48550	18.851	-1.967	0.006
PA14_48560	150.794	-0.754	0.035	-2.989	0.000	-1.249	0.003	PA14_48560	181.555	-2.153	0.000
PA14_48570	165.547	-1.265	0.000	-3.842	0.000	-2.719	0.000	PA14_48570	203.134	-1.571	0.000
PA14_48590	296.332	-1.618	0.000	-3.961	0.000	-3.235	0.000	PA14_48590	334.641	-1.737	0.000
PA14_48600	130.142	-0.825	0.151	-2.369	0.000	-1.222	0.056	PA14_48600	130.305	-1.790	0.000
PA14_48610	25.550	-0.692	0.270	-2.475	0.000	-1.438	0.047	PA14_48610	83.167	-2.574	0.000
PA14_48620	24.768	-1.046	0.084	-3.542	0.000	-3.788	0.000	PA14_48620	70.390	-2.168	0.000
PA14_48630	31.455	-1.761	0.001	-3.620	0.000	-1.010	0.137	PA14_48630	85.926	-1.550	0.001
PA14_48640	9.480	0.668	0.541	-0.453	0.643	0.339	0.786	PA14_48640	27.429	-1.068	0.077
PA14_48650	41.181	-0.265	0.705	-0.592	0.260	-0.158	0.846	PA14_48650	120.506	-0.743	0.047
PA14_48660	37.893	0.353	0.585	-0.134	0.819	0.113	0.890	PA14_48660	89.312	-0.316	0.470
PA14_48680	99.566	-0.634	0.147	-0.184	0.661	0.554	0.267	PA14_48680	488.306	-2.197	0.000
PA14_48700	494.758	0.044	0.920	0.342	0.237	-0.673	0.066	PA14_48700	891.791	0.379	0.109
PA14_48710	57.488	0.016	0.980	0.139	0.777	-0.537	0.400	PA14_48710	218.775	-0.455	0.125
PA14_48730	442.089	-0.297	0.440	-0.156	0.649	0.082	0.868	PA14_48730	798.408	-0.615	0.007
PA14_48740	294.261	0.020	0.965	-0.490	0.075	-0.522	0.161	PA14_48740	818.797	-0.278	0.292
PA14_48750	205.708	0.095	0.869	0.091	0.838	-0.403	0.455	PA14_48750	430.590	-0.259	0.367
PA14_48760	132.724	-2.316	0.000	-3.089	0.000	-1.859	0.000	PA14_48760	512.184	-2.801	0.000
PA14_48770	126.769	0.106	0.835	0.235	0.523	0.272	0.588	PA14_48770	289.930	0.702	0.009
PA14_48780	182.596	-0.388	0.319	0.230	0.502	-1.153	0.007	PA14_48780	391.095	-0.310	0.232
PA14_48790	1702.748	-0.430	0.169	0.250	0.396	-0.859	0.012	PA14_48790	3442.735	0.959	0.000
PA14_48800	2500.701	0.825	0.004	1.073	0.000	0.471	0.203	PA14_48800	7869.253	-0.107	0.779
PA14_48810	1848.730	0.750	0.080	1.100	0.004	-1.192	0.014	PA14_48810	6598.009	0.786	0.008
PA14_48830	1627.691	0.969	0.000	1.107	0.000	-1.521	0.000	PA14_48830	3509.844	1.918	0.000
PA14_48840	46.994	-0.072	0.920	-0.753	0.098	0.834	0.134	PA14_48840	177.386	-1.022	0.072
PA14_48850	27.210	-0.080	0.929	0.617	0.271	0.880	0.215	PA14_48850	83.407	0.236	0.565
PA14_48860	548.109	0.224	0.490	0.679	0.006	0.867	0.005	PA14_48860	907.214	1.568	0.000
PA14_48870	630.238	0.848	0.003	0.958	0.000	1.453	0.000	PA14_48870	1619.639	0.736	0.001

PA14_48880	739.180	0.191	0.584	-0.334	0.227	1.726	0.000	PA14_48880	2211.984	-1.175	0.000
PA14_48890	132.017	-0.380	0.392	-0.451	0.219	0.351	0.485	PA14_48890	304.477	-0.491	0.077
PA14_48900	0.852	-0.046	0.980	-0.851	0.499	-0.411	0.753	PA14_48900	3.639	0.688	0.792
PA14_48910	80.690	-0.374	0.430	-0.964	0.010	0.498	0.323	PA14_48910	154.100	-0.318	0.483
PA14_48920	3.315	-0.476	0.757	-2.565	0.022	-0.360	0.808	PA14_48920	18.159	-1.572	0.035
PA14_48930	58.081	-0.731	0.161	-1.206	0.007	-0.666	0.289	PA14_48930	169.485	-0.451	0.169
PA14_48940	102.573	-0.892	0.074	-1.558	0.000	-2.092	0.000	PA14_48940	717.410	-2.069	0.000
PA14_48950	580.195	-0.486	0.107	-1.274	0.000	-1.504	0.000	PA14_48950	2711.343	-2.551	0.001
PA14_48960	10.136	-0.576	0.621	-0.532	0.578	-1.071	0.348	PA14_48960	50.440	-0.355	0.450
PA14_48970	76.008	0.023	0.979	-0.206	0.740	0.066	0.940	PA14_48970	97.103	-0.330	0.584
PA14_48980	13.541	0.357	0.722	0.123	0.887	-0.323	0.775	PA14_48980	61.869	-1.087	0.009
PA14_48990	4.138	-0.830	0.562	-0.829	0.486	1.780	0.132	PA14_48990	30.840	-0.121	0.890
PA14_49000	7.244	-1.284	0.214	-2.079	0.022	0.184	0.885	PA14_49000	85.232	-0.513	0.176
PA14_49010	155.106	0.316	0.526	0.354	0.388	0.844	0.084	PA14_49010	531.453	-1.190	0.000
PA14_49020	1311.486	0.212	0.652	-0.151	0.707	-0.391	0.424	PA14_49020	3333.852	0.055	0.796
PA14_49030	11651.365	0.155	0.670	0.139	0.646	-0.228	0.555	PA14_49030	19424.375	1.034	0.005
PA14_49040	356.576	-0.084	0.855	-0.151	0.660	0.975	0.008	PA14_49040	882.011	-0.692	0.003
PA14_49050	85.471	0.182	0.730	-0.197	0.640	2.070	0.000	PA14_49050	645.936	-2.522	0.000
PA14_49060	79.069	0.873	0.049	1.053	0.007	2.596	0.000	PA14_49060	72.228	0.130	0.840
PA14_49070	108.393	-0.666	0.092	-1.530	0.000	-0.477	0.335	PA14_49070	251.715	-0.901	0.001
PA14_49080	106.560	-0.541	0.168	-1.174	0.000	-0.314	0.532	PA14_49080	492.731	-1.604	0.000
PA14_49090	6.584	0.726	0.545	0.112	0.923	1.797	0.088	PA14_49090	20.209	-0.297	0.702
PA14_49100	17.112	-1.368	0.092	-0.315	0.686	0.008	0.994	PA14_49100	33.109	-0.018	1.000
PA14_49110	92.766	0.756	0.051	1.105	0.001	0.471	0.352	PA14_49110	191.706	0.997	0.001
PA14_49130	226.602	-2.295	0.000	1.537	0.000	0.294	0.587	PA14_49130	697.730	1.260	0.000
PA14_49150	130.435	0.586	0.189	-0.002	0.996	0.121	0.849	PA14_49150	280.623	0.544	0.050
PA14_49160	578.284	-1.018	0.000	-0.747	0.002	-0.686	0.033	PA14_49160	3126.657	0.177	0.436
PA14_49170	1330.945	-2.380	0.000	-0.353	0.382	-2.073	0.000	PA14_49170	21154.749	0.039	0.858
PA14_49180	545.071	-3.398	0.000	-1.783	0.000	-2.994	0.000	PA14_49180	13209.197	-0.487	0.027
PA14_49200	13780.871	-5.595	0.000	-3.235	0.000	-1.477	0.000	PA14_49200	328359.885	-2.121	0.000
PA14_49210	351.398	-2.571	0.000	-4.633	0.000	0.227	0.639	PA14_49210	2097.058	-3.024	0.000
PA14_49220	125.970	-2.285	0.000	-3.441	0.000	1.221	0.007	PA14_49220	969.809	-3.243	0.000
PA14_49230	412.141	-2.179	0.000	-3.731	0.000	0.461	0.268	PA14_49230	2138.563	-2.331	0.000
PA14_49250	1509.174	-2.310	0.000	-3.941	0.000	0.090	0.809	PA14_49250	4570.941	-2.350	0.000
PA14_49260	145.180	-2.133	0.000	-3.706	0.000	-0.709	0.093	PA14_49260	989.299	-2.829	0.000
PA14_49270	239.281	-2.075	0.000	-3.303	0.000	-0.774	0.075	PA14_49270	1516.692	-3.146	0.000
PA14_49280	247.896	-0.128	0.798	-0.308	0.401	-0.618	0.181	PA14_49280	506.367	0.664	0.005
PA14_49290	220.925	1.266	0.000	1.878	0.000	1.995	0.000	PA14_49290	373.392	-0.553	0.031
PA14_49300	342.804	-2.149	0.000	-2.378	0.000	-0.769	0.023	PA14_49300	1115.790	-1.701	0.000
PA14_49310	115.060	-3.057	0.000	-5.241	0.000	-1.889	0.000	PA14_49310	159.079	-1.682	0.000
PA14_49320	295.536	-1.883	0.000	-2.447	0.000	0.144	0.734	PA14_49320	2618.027	-3.101	0.000
PA14_49330	126.289	-2.170	0.000	-3.455	0.000	-0.551	0.219	PA14_49330	1241.929	-3.674	0.000
PA14_49340	46.256	-0.073	0.936	-0.880	0.131	-1.155	0.133	PA14_49340	267.132	-0.184	0.578
PA14_49350	127.226	-0.519	0.355	-0.050	0.929	0.001	0.999	PA14_49350	511.085	0.029	0.938
PA14_49360	78.542	-0.866	0.035	-0.776	0.034	0.306	0.564	PA14_49360	229.455	-1.816	0.000
PA14_49380	458.586	0.620	0.034	0.805	0.002	0.700	0.042	PA14_49380	849.028	1.559	0.000
PA14_49390	209.933	0.223	0.557	0.238	0.444	0.457	0.242	PA14_49390	377.891	0.244	0.372
PA14_49400	142.042	-0.371	0.348	-0.356	0.289	-0.881	0.042	PA14_49400	599.448	-0.991	0.000
PA14_49410	661.206	-1.314	0.000	-0.976	0.005	-2.283	0.000	PA14_49410	2009.485	-0.178	0.454
PA14_49420	203.292	0.009	0.985	0.500	0.096	-0.113	0.820	PA14_49420	479.885	-0.116	0.689
PA14_49440	186.471	0.738	0.026	1.154	0.000	0.073	0.889	PA14_49440	462.427	0.452	0.078
PA14_49460	8238.970	-0.018	0.967	-0.219	0.442	1.369	0.000	PA14_49460	11336.994	0.694	0.127
PA14_49470	9490.203	-0.261	0.328	-0.241	0.304	0.825	0.002	PA14_49470	11945.497	0.611	0.050
PA14_49480	17109.345	-0.581	0.074	-1.980	0.000	-0.268	0.529	PA14_49480	22701.951	-0.729	0.001
PA14_49500	70.284	-1.214	0.010	-1.705	0.000	-0.633	0.290	PA14_49500	121.319	-0.769	0.292
PA14_49510	1898.238	0.442	0.300	-0.627	0.084	-1.540	0.000	PA14_49510	22318.094	-1.596	0.000
PA14_49520	4307.798	0.159	0.684	-0.391	0.185	-0.532	0.155	PA14_49520	58758.979	-2.569	0.000
PA14_49530	63.006	-0.456	0.412	-1.389	0.002	-0.259	0.703	PA14_49530	265.473	-0.282	0.342
PA14_49540	111.343	-0.843	0.027	-2.253	0.000	-1.467	0.001	PA14_49540	432.499	-0.383	0.123

PA14_49560	28.329	-0.855	0.229	-0.127	0.854	0.974	0.181
PA14_49570	7.971	-0.132	0.929	0.486	0.629	1.264	0.250
PA14_49580	3.959	-0.080	0.965	0.459	0.700	0.067	0.966
PA14_49590	76.144	0.621	0.152	0.642	0.089	0.264	0.658
PA14_49610	26.274	0.128	0.879	0.244	0.686	0.439	0.575
PA14_49620	9.781	-0.254	0.833	-0.492	0.581	-0.021	0.986
PA14_49630	83.321	0.867	0.034	1.235	0.000	0.387	0.495
PA14_49640	109.129	1.438	0.000	0.662	0.060	-0.249	0.669
PA14_49650	3974.128	6.338	0.000	2.271	0.000	2.689	0.000
PA14_49660	81.170	0.070	0.910	0.502	0.200	0.070	0.915
PA14_49680	309.692	0.826	0.149	1.931	0.000	-0.334	0.660
PA14_49690	477.303	-0.246	0.464	-2.387	0.000	1.474	0.000
PA14_49700	30.248	-0.611	0.389	-0.933	0.105	-0.162	0.858
PA14_49710	178.866	-1.033	0.002	-2.159	0.000	0.415	0.341
PA14_49720	3.909	-0.727	0.634	0.735	0.550	2.240	0.061
PA14_49730	33.626	-0.679	0.285	-0.560	0.304	0.829	0.204
PA14_49740	604.094	0.287	0.340	0.150	0.587	0.643	0.040
PA14_49750	100.460	-2.197	0.000	-3.959	0.000	0.691	0.179
PA14_49760	77.158	-3.562	0.000	-4.147	0.000	-1.186	0.047
PA14_49780	40.076	0.638	0.260	1.013	0.027	-0.224	0.784
PA14_49790	112.389	0.873	0.014	0.726	0.027	-0.337	0.532
PA14_49800	657.841	-0.558	0.185	0.615	0.096	-1.441	0.001
PA14_49810	326.981	0.953	0.007	1.850	0.000	1.000	0.017
PA14_49820	94.882	-0.237	0.617	-0.343	0.355	-0.293	0.585
PA14_49840	759.059	-0.484	0.154	-0.757	0.010	-1.456	0.000
PA14_49850	354.158	-2.511	0.000	-2.454	0.000	-0.431	0.324
PA14_49860	27.338	-1.795	0.009	-1.260	0.043	0.201	0.832
PA14_49870	683.682	-0.582	0.078	-1.270	0.000	-1.507	0.000
PA14_49880	412.078	-0.197	0.592	-0.390	0.170	1.058	0.001
PA14_49890	721.208	-0.618	0.045	-1.298	0.000	0.443	0.242
PA14_49900	318.874	-0.979	0.002	-2.369	0.000	-0.273	0.530
PA14_49910	559.894	0.361	0.262	0.592	0.027	1.646	0.000
PA14_49920	322.533	0.804	0.012	1.235	0.000	2.032	0.000
PA14_49930	305.422	0.676	0.049	1.589	0.000	1.901	0.000
PA14_49940	759.917	-0.327	0.261	-0.429	0.083	0.979	0.001
PA14_49960	91.333	0.198	0.708	0.337	0.398	1.578	0.000
PA14_49970	117.074	0.323	0.525	0.860	0.024	0.067	0.918
PA14_49990	894.008	-0.071	0.920	-0.520	0.275	0.652	0.277
PA14_50000	944.668	1.625	0.000	2.243	0.000	4.355	0.000
PA14_50010	215.800	-1.100	0.013	-0.099	0.839	1.117	0.026
PA14_50020	126.032	-1.073	0.008	-0.264	0.514	0.912	0.045
PA14_50030	98.825	1.526	0.000	1.338	0.001	0.908	0.101
PA14_50040	92.482	-0.034	0.959	0.938	0.009	0.793	0.102
PA14_50050	6.399	1.262	0.255	0.743	0.469	1.488	0.186
PA14_50060	129.905	-1.246	0.001	-1.622	0.000	0.708	0.132
PA14_50070	313.428	0.118	0.780	0.174	0.591	2.141	0.000
PA14_50080	398.027	1.082	0.000	1.496	0.000	1.056	0.003
PA14_50100	390.907	0.632	0.027	1.049	0.000	0.539	0.124
PA14_50110	326.355	0.556	0.080	0.919	0.001	0.640	0.085
PA14_50130	1343.614	0.101	0.763	0.451	0.056	0.457	0.140
PA14_50140	1364.474	0.241	0.416	0.631	0.006	0.770	0.008
PA14_50160	373.854	-0.430	0.250	0.467	0.141	0.625	0.122
PA14_50180	1022.344	0.512	0.169	1.044	0.001	0.964	0.017
PA14_50200	933.073	0.613	0.050	1.161	0.000	0.695	0.058
PA14_50220	6872.682	0.647	0.009	0.675	0.003	-0.745	0.011
PA14_50240	673.383	-0.992	0.003	-1.739	0.000	-0.861	0.037
PA14_50250	5239.748	-1.146	0.000	-1.998	0.000	-0.285	0.506
PA14_50270	5942.901	-0.585	0.014	-1.008	0.000	0.347	0.254
PA14_50280	2038.939	-0.526	0.068	-1.643	0.000	0.505	0.141

PA14_49560	42.459	0.555	0.286
PA14_49570	14.207	0.521	0.604
PA14_49580	14.649	1.778	0.032
PA14_49590	153.294	0.334	0.316
PA14_49610	46.168	-0.530	0.312
PA14_49620	6.130	2.174	0.120
PA14_49630	191.009	0.782	0.007
PA14_49640	218.021	0.302	0.314
PA14_49650	410.675	-0.723	0.004
PA14_49660	336.399	-0.053	0.868
PA14_49680	282.282	-0.027	0.945
PA14_49690	7451.334	-0.610	0.004
PA14_49700	208.644	-0.492	0.126
PA14_49710	1239.958	0.842	0.098
PA14_49720	8.091	1.380	0.439
PA14_49730	106.570	-0.144	0.710
PA14_49740	1082.269	0.340	0.139
PA14_49750	326.475	-2.456	0.000
PA14_49760	270.236	-2.803	0.000
PA14_49780	95.013	1.835	0.000
PA14_49790	281.080	1.178	0.000
PA14_49800	571.292	-0.212	0.450
PA14_49810	517.274	0.193	0.493
PA14_49820	226.137	-0.147	0.647
PA14_49840	1767.363	0.063	0.843
PA14_49850	422.496	-0.005	0.978
PA14_49860	134.004	-1.761	0.000
PA14_49870	2362.636	-1.309	0.000
PA14_49880	1188.856	-1.400	0.000
PA14_49890	2305.270	0.488	0.039
PA14_49900	1681.696	0.810	0.033
PA14_49910	1115.422	-2.422	0.000
PA14_49920	665.345	-0.580	0.012
PA14_49930	414.047	0.254	0.354
PA14_49940	3164.062	-0.563	0.009
PA14_49960	468.897	-0.564	0.020
PA14_49970	173.106	0.741	0.014
PA14_49990	1936.672	0.099	0.880
PA14_50000	429.976	1.477	0.000
PA14_50010	1134.484	-1.848	0.000
PA14_50020	1210.199	-2.384	0.000
PA14_50030	221.154	0.471	0.404
PA14_50040	216.939	1.119	0.000
PA14_50050	8.983	-0.317	0.831
PA14_50060	892.771	-2.796	0.000
PA14_50070	671.279	-0.230	0.351
PA14_50080	512.298	0.533	0.030
PA14_50100	570.025	0.835	0.000
PA14_50110	773.393	-0.044	0.892
PA14_50130	2886.854	-0.051	0.859
PA14_50140	2610.901	0.358	0.106
PA14_50160	890.515	0.481	0.085
PA14_50180	1181.976	0.644	0.004
PA14_50200	1584.654	0.533	0.017
PA14_50220	10817.303	0.558	0.007
PA14_50240	5683.292	-1.866	0.000
PA14_50250	24288.009	-1.907	0.000
PA14_50270	21360.644	-1.364	0.000
PA14_50280	6402.799	-1.746	0.000

PA14_50290	15387.466	-0.537	0.052	-1.850	0.000	0.723	0.022	PA14_50290	45319.388	-1.608	0.000
PA14_50300	2612.083	-0.246	0.572	-0.185	0.622	-0.472	0.295	PA14_50300	3305.349	-0.387	0.077
PA14_50310	229.152	-0.129	0.794	-0.387	0.277	-0.489	0.299	PA14_50310	623.078	-0.129	0.658
PA14_50320	249.322	-0.542	0.104	0.067	0.848	-0.350	0.406	PA14_50320	758.477	-0.467	0.049
PA14_50330	534.019	-0.448	0.146	-0.654	0.014	-0.846	0.014	PA14_50330	1709.869	-0.752	0.001
PA14_50340	2255.262	-0.082	0.809	-0.425	0.068	-0.641	0.028	PA14_50340	5901.310	-0.892	0.000
PA14_50360	1886.718	-0.077	0.832	-0.427	0.079	-0.632	0.039	PA14_50360	3610.904	-0.289	0.210
PA14_50380	872.239	0.356	0.254	0.323	0.243	0.179	0.656	PA14_50380	1631.124	-0.099	0.690
PA14_50410	343.564	0.168	0.671	0.250	0.420	0.017	0.972	PA14_50410	603.603	0.221	0.393
PA14_50420	1495.757	0.454	0.214	0.769	0.013	0.918	0.019	PA14_50420	1718.108	0.472	0.033
PA14_50430	1057.821	-0.228	0.499	-0.368	0.176	-0.210	0.590	PA14_50430	2487.502	0.187	0.407
PA14_50440	751.685	-0.140	0.701	0.248	0.376	-0.572	0.098	PA14_50440	2567.862	-0.221	0.331
PA14_50450	2290.634	-0.175	0.614	-0.555	0.033	-0.611	0.067	PA14_50450	3978.960	0.501	0.138
PA14_50460	819.828	-0.237	0.550	-0.511	0.099	-0.698	0.076	PA14_50460	2808.698	-0.041	0.900
PA14_50470	1143.039	-0.291	0.477	-0.353	0.300	-0.601	0.155	PA14_50470	3674.302	-0.318	0.171
PA14_50480	1508.898	-0.049	0.913	0.109	0.740	-0.312	0.436	PA14_50480	4236.559	-0.388	0.093
PA14_50500	2151.434	-1.666	0.000	-2.940	0.000	-4.801	0.000	PA14_50500	13500.982	-0.332	0.313
PA14_50510	134.815	0.512	0.157	0.632	0.042	-0.246	0.626	PA14_50510	542.983	-0.303	0.296
PA14_50520	4880.184	-0.076	0.835	-2.120	0.000	-1.680	0.000	PA14_50520	5643.081	-0.008	1.000
PA14_50530	98.607	-0.371	0.518	-2.313	0.000	-1.892	0.001	PA14_50530	162.655	0.873	0.093
PA14_50540	311.986	-0.270	0.450	-2.260	0.000	-2.094	0.000	PA14_50540	385.605	1.077	0.000
PA14_50550	781.885	-0.015	0.976	-1.863	0.000	-2.207	0.000	PA14_50550	938.034	0.819	0.005
PA14_50560	288.061	0.103	0.844	-1.578	0.000	-2.678	0.000	PA14_50560	647.306	-1.161	0.000
PA14_50570	1963.718	0.004	0.991	0.108	0.700	-0.771	0.012	PA14_50570	3147.537	0.261	0.262
PA14_50590	899.693	0.493	0.112	0.730	0.007	0.481	0.192	PA14_50590	1306.719	0.159	0.548
PA14_50600	176.450	1.445	0.002	2.568	0.000	0.572	0.378	PA14_50600	268.918	0.185	0.519
PA14_50610	51.921	-0.966	0.063	-1.092	0.018	-0.890	0.158	PA14_50610	176.402	-0.451	0.149
PA14_50620	123.232	-0.069	0.897	0.015	0.972	0.393	0.403	PA14_50620	470.960	-0.549	0.029
PA14_50630	181.449	0.137	0.822	-0.601	0.154	-1.370	0.010	PA14_50630	507.882	0.678	0.031
PA14_50640	110.676	0.090	0.881	-0.459	0.264	-1.950	0.000	PA14_50640	830.842	-0.484	0.039
PA14_50650	654.724	0.035	0.951	0.140	0.733	-1.086	0.015	PA14_50650	1808.176	0.510	0.021
PA14_50660	1128.644	0.388	0.198	0.163	0.577	-1.035	0.001	PA14_50660	2343.042	0.432	0.054
PA14_50670	212.279	0.528	0.101	0.630	0.026	0.072	0.881	PA14_50670	963.406	0.455	0.053
PA14_50680	90.525	-0.740	0.062	-1.272	0.000	-1.438	0.003	PA14_50680	459.408	-0.659	0.007
PA14_50690	168.139	-1.069	0.001	-1.079	0.000	-1.173	0.002	PA14_50690	653.376	-1.083	0.000
PA14_50700	259.086	-0.338	0.361	0.119	0.729	-1.025	0.009	PA14_50700	773.699	-0.122	0.603
PA14_50710	285.407	-0.688	0.026	-0.244	0.423	-0.864	0.019	PA14_50710	857.858	-0.007	0.955
PA14_50720	53.346	-0.662	0.235	-0.027	0.963	-0.781	0.231	PA14_50720	236.241	-0.007	0.969
PA14_50730	506.862	-0.653	0.049	-0.022	0.954	-0.499	0.223	PA14_50730	1526.378	0.235	0.344
PA14_50740	7693.658	-1.644	0.000	-0.851	0.004	-2.235	0.000	PA14_50740	63054.388	-1.615	0.000
PA14_50750	36.909	-0.709	0.275	-0.217	0.718	-1.736	0.023	PA14_50750	122.712	0.859	0.008
PA14_50760	38.851	0.285	0.697	0.190	0.752	2.448	0.000	PA14_50760	127.250	1.495	0.000
PA14_50770	141.232	1.390	0.000	0.382	0.304	2.794	0.000	PA14_50770	815.033	3.040	0.000
PA14_50790	203.865	-0.689	0.033	-0.357	0.243	-0.915	0.019	PA14_50790	655.871	1.461	0.000
PA14_50800	1297.079	-0.918	0.000	-1.225	0.000	-0.345	0.292	PA14_50800	3210.442	1.480	0.000
PA14_50810	1822.722	-0.696	0.041	-0.236	0.496	1.005	0.009	PA14_50810	3001.896	1.033	0.000
PA14_50820	407.689	-0.292	0.358	-0.777	0.002	-0.932	0.005	PA14_50820	1323.751	-0.358	0.119
PA14_50830	134.084	0.069	0.898	0.397	0.256	-0.375	0.459	PA14_50830	200.514	-0.042	0.918
PA14_50840	1100.114	1.240	0.000	1.776	0.000	1.431	0.000	PA14_50840	1199.547	1.183	0.000
PA14_50850	34.576	-0.411	0.580	0.424	0.468	-0.764	0.344	PA14_50850	95.796	1.381	0.001
PA14_50860	274.277	-0.690	0.029	-1.361	0.000	-1.449	0.000	PA14_50860	825.504	-1.122	0.000
PA14_50870	192.488	-1.206	0.000	-1.374	0.000	-0.827	0.052	PA14_50870	477.384	-0.697	0.004
PA14_50880	1203.542	-3.301	0.000	-5.757	0.000	-2.325	0.000	PA14_50880	9919.643	-5.272	0.000
PA14_50890	365.657	-0.235	0.659	-0.363	0.388	0.196	0.744	PA14_50890	1679.392	-1.207	0.000
PA14_50900	733.958	-0.563	0.190	-0.849	0.022	-0.153	0.793	PA14_50900	1947.717	-0.663	0.002
PA14_50910	55.390	0.912	0.144	0.239	0.708	-0.927	0.251	PA14_50910	325.432	-0.744	0.005
PA14_50920	59.229	0.798	0.113	1.225	0.004	0.432	0.516	PA14_50920	150.559	0.783	0.011
PA14_50930	292.756	-0.225	0.581	-0.512	0.102	0.477	0.246	PA14_50930	1094.993	-0.892	0.000
PA14_50940	1092.445	0.431	0.143	1.001	0.000	-0.780	0.020	PA14_50940	3522.090	-0.280	0.215

PA14_50950	102.646	0.331	0.604	0.729	0.131	0.193	0.796	PA14_50950	772.583	0.256	0.360
PA14_50970	449.390	-0.381	0.199	-0.318	0.231	-1.066	0.001	PA14_50970	1082.950	-0.448	0.053
PA14_50980	1229.058	0.284	0.441	0.328	0.290	1.134	0.001	PA14_50980	1621.564	0.161	0.491
PA14_51000	1014.780	0.643	0.013	1.223	0.000	0.006	0.989	PA14_51000	2439.042	1.151	0.000
PA14_51010	814.961	0.568	0.126	1.199	0.000	0.700	0.100	PA14_51010	3360.704	0.220	0.348
PA14_51020	378.170	-0.417	0.179	-0.509	0.059	-1.141	0.001	PA14_51020	1500.705	-0.181	0.422
PA14_51040	52.509	-0.610	0.216	-1.030	0.013	-0.827	0.156	PA14_51040	201.736	-1.355	0.000
PA14_51050	83.568	-0.635	0.167	-0.802	0.043	-1.262	0.017	PA14_51050	246.952	-1.196	0.000
PA14_51060	135.199	0.130	0.799	0.245	0.519	-0.576	0.244	PA14_51060	267.957	0.331	0.266
PA14_51070	52.734	0.937	0.042	0.902	0.030	0.325	0.626	PA14_51070	102.895	0.587	0.112
PA14_51080	102.020	0.835	0.048	0.978	0.009	0.516	0.343	PA14_51080	142.321	0.824	0.010
PA14_51090	96.909	0.262	0.581	-0.424	0.268	-0.853	0.093	PA14_51090	161.626	0.590	0.055
PA14_51100	380.070	0.608	0.061	0.047	0.897	-0.365	0.392	PA14_51100	562.198	-0.473	0.045
PA14_51110	98.445	-0.219	0.737	-0.291	0.569	-0.545	0.403	PA14_51110	93.509	0.348	0.365
PA14_51120	70.705	-0.012	0.985	-0.184	0.679	-0.405	0.496	PA14_51120	95.375	0.789	0.030
PA14_51150	116.396	1.550	0.000	1.647	0.000	1.284	0.005	PA14_51150	123.942	0.870	0.009
PA14_51160	101.807	1.127	0.004	1.909	0.000	0.330	0.573	PA14_51160	124.995	0.424	0.205
PA14_51170	173.229	0.464	0.254	1.083	0.001	-0.616	0.209	PA14_51170	249.035	0.855	0.002
PA14_51190	221.935	0.705	0.026	1.003	0.000	0.892	0.015	PA14_51190	361.128	0.900	0.000
PA14_51200	95.694	0.584	0.151	1.017	0.002	0.591	0.221	PA14_51200	205.399	1.008	0.000
PA14_51205	126.963	0.512	0.185	-0.079	0.844	-0.756	0.113	PA14_51205	217.262	0.236	0.421
PA14_51220	1846.658	0.227	0.526	1.113	0.000	1.275	0.000	PA14_51220	4191.315	-0.528	0.015
PA14_51230	94.334	0.040	0.965	1.297	0.010	0.405	0.595	#N/A	#N/A	#N/A	#N/A
PA14_51240	1998.668	-0.095	0.786	0.168	0.534	-1.443	0.000	PA14_51240	2490.879	0.895	0.000
PA14_51250	1407.951	0.933	0.007	1.615	0.000	0.809	0.053	PA14_51250	1761.345	0.074	0.757
PA14_51260	4158.485	0.195	0.621	0.133	0.695	-0.475	0.228	PA14_51260	4262.410	1.157	0.000
PA14_51270	2412.984	0.456	0.092	0.602	0.012	-0.460	0.155	PA14_51270	5493.922	0.856	0.000
PA14_51280	846.318	0.240	0.634	-0.046	0.922	-0.826	0.090	PA14_51280	2514.175	0.713	0.025
PA14_51290	351.878	-0.389	0.288	-0.103	0.772	-0.473	0.259	PA14_51290	748.458	0.402	0.101
PA14_51300	491.671	0.058	0.889	0.756	0.003	0.068	0.876	PA14_51300	597.911	0.993	0.000
PA14_51310	49.331	-0.095	0.907	0.360	0.508	0.579	0.402	PA14_51310	131.195	1.188	0.000
PA14_51320	1762.491	0.514	0.161	0.416	0.218	0.106	0.840	PA14_51320	3230.367	0.598	0.005
PA14_51330	1155.602	0.079	0.844	0.126	0.680	-0.069	0.874	PA14_51330	2793.032	-0.531	0.153
PA14_51340	1636.967	0.172	0.719	-0.633	0.068	1.035	0.014	PA14_51340	5228.963	-0.648	0.057
PA14_51350	1450.480	-0.730	0.025	-1.925	0.000	-2.535	0.000	PA14_51350	1464.461	-1.095	0.000
PA14_51360	8883.293	-0.980	0.004	-2.392	0.000	-3.487	0.000	PA14_51360	5580.142	-1.014	0.000
PA14_51380	4824.425	-0.361	0.435	-1.526	0.000	-3.042	0.000	PA14_51380	3824.791	-1.107	0.000
PA14_51390	2893.599	-1.276	0.000	-2.767	0.000	-4.453	0.000	PA14_51390	3294.840	-1.400	0.000
PA14_51410	3493.312	-0.972	0.000	-2.300	0.000	-4.389	0.000	PA14_51410	2703.110	-0.519	0.019
PA14_51420	2599.663	-1.190	0.000	-2.815	0.000	-4.641	0.000	PA14_51420	2989.688	-0.291	0.204
PA14_51430	5807.129	-0.474	0.059	-1.113	0.000	-3.018	0.000	PA14_51430	9134.772	0.054	0.840
PA14_51440	324.027	0.278	0.457	0.044	0.903	0.185	0.683	PA14_51440	815.255	0.497	0.034
PA14_51450	1105.653	-0.274	0.441	-0.654	0.020	-0.091	0.842	PA14_51450	2717.879	-1.203	0.000
PA14_51460	38.669	0.312	0.622	-0.565	0.265	-0.039	0.962	PA14_51460	90.530	-0.072	0.947
PA14_51470	72.125	0.011	0.987	-0.690	0.133	-2.145	0.001	PA14_51470	140.454	0.377	0.254
PA14_51480	109.224	-1.351	0.000	-1.562	0.000	-0.782	0.079	PA14_51480	297.968	-0.489	0.069
PA14_51490	98.092	-1.446	0.000	-2.185	0.000	-0.971	0.035	PA14_51490	1328.567	-2.744	0.000
PA14_51500	81.934	-0.637	0.163	0.136	0.758	-0.123	0.848	PA14_51500	190.048	0.686	0.020
PA14_51510	424.288	0.236	0.520	0.364	0.218	-0.724	0.056	PA14_51510	1161.931	0.557	0.013
PA14_51520	1582.420	-0.468	0.203	-0.965	0.002	-2.001	0.000	PA14_51520	7896.179	-1.593	0.000
PA14_51530	2415.652	0.779	0.001	-0.248	0.305	-1.241	0.000	PA14_51530	9627.571	-1.024	0.000
PA14_51540	206.484	0.981	0.003	1.881	0.000	1.287	0.001	PA14_51540	450.376	0.722	0.003
PA14_51550	379.861	0.453	0.157	0.945	0.000	1.327	0.000	PA14_51550	1651.113	-0.101	0.679
PA14_51560	914.785	0.339	0.243	-0.085	0.770	0.340	0.317	PA14_51560	4424.911	-0.585	0.006
PA14_51570	1855.088	0.420	0.504	-1.435	0.003	-1.937	0.001	PA14_51570	8455.287	-0.806	0.001
PA14_51580	228.494	0.136	0.807	-0.236	0.575	1.993	0.000	PA14_51580	800.789	-1.201	0.014
PA14_51590	924.370	0.008	0.987	-0.565	0.076	0.694	0.085	PA14_51590	3727.343	-0.725	0.001
PA14_51600	596.429	-0.167	0.766	-1.056	0.006	-0.670	0.197	PA14_51600	1399.379	-1.376	0.015
PA14_51610	109.851	0.258	0.722	-0.789	0.143	0.052	0.950	PA14_51610	448.439	-2.431	0.000

PA14_51620	5.677	-1.588	0.147	-0.832	0.399	-1.586	0.171	PA14_51620	1871.102	-0.323	0.170
PA14_51630	10.014	1.020	0.295	0.388	0.680	1.435	0.148	PA14_51630	750.747	0.015	0.964
PA14_51640	83.198	-0.032	0.964	-0.244	0.593	0.647	0.230	PA14_51640	519.804	-1.246	0.000
PA14_51650	108.358	-0.135	0.791	-0.796	0.023	0.482	0.306	PA14_51650	866.016	-0.990	0.000
PA14_51660	73.285	1.020	0.039	2.972	0.000	0.282	0.704	#N/A	#N/A	#N/A	#N/A
PA14_51670	506.987	0.428	0.398	1.735	0.000	1.354	0.006	PA14_51670	902.559	-0.691	0.002
PA14_51680	544.851	1.439	0.000	3.215	0.000	2.834	0.000	PA14_51680	660.251	1.347	0.000
PA14_51690	1122.330	0.392	0.290	0.997	0.001	0.988	0.010	PA14_51690	2727.683	0.890	0.000
PA14_51710	16664.108	-0.753	0.022	-0.991	0.001	-1.688	0.000	PA14_51710	17799.500	1.213	0.000
PA14_51720	6685.523	-0.358	0.211	-0.490	0.048	-0.986	0.001	PA14_51720	10814.358	1.276	0.000
PA14_51730	3521.343	0.094	0.782	0.646	0.005	0.440	0.157	PA14_51730	8013.486	-0.405	0.071
PA14_51740	2606.492	-0.532	0.034	-0.074	0.788	-0.897	0.002	PA14_51740	3115.823	0.408	0.064
PA14_51750	1568.418	-0.523	0.065	-0.202	0.475	-0.598	0.073	PA14_51750	2269.137	0.588	0.006
PA14_51770	1160.552	0.048	0.911	0.574	0.031	-0.181	0.657	PA14_51770	2142.794	0.419	0.059
PA14_51780	1361.952	0.265	0.490	0.543	0.075	0.538	0.176	PA14_51780	2753.959	0.339	0.124
PA14_51790	768.462	0.230	0.522	0.318	0.279	0.149	0.730	PA14_51790	2107.404	0.049	0.858
PA14_51800	1318.183	0.543	0.055	0.721	0.004	0.617	0.062	PA14_51800	2474.841	0.644	0.007
PA14_51810	2353.983	-0.034	0.936	-0.282	0.315	-1.000	0.002	PA14_51810	2742.138	1.115	0.000
PA14_51820	4942.829	0.358	0.230	0.534	0.037	0.430	0.206	PA14_51820	6240.749	1.494	0.000
PA14_51830	6895.170	-1.149	0.000	-1.786	0.000	-0.734	0.064	PA14_51830	43535.644	-1.560	0.000
PA14_51840	462.072	0.538	0.294	0.798	0.064	0.853	0.121	PA14_51840	1331.211	1.382	0.000
PA14_51850	42.088	-1.771	0.004	-2.085	0.000	0.014	0.987	PA14_51850	916.358	-2.853	0.000
PA14_51860	317.345	-1.782	0.000	-2.268	0.000	-0.261	0.542	PA14_51860	2932.892	-2.367	0.000
PA14_51880	24425.913	-0.289	0.357	-1.960	0.000	-4.014	0.000	PA14_51880	32844.227	-0.263	0.555
PA14_51890	54.858	-0.327	0.550	-0.091	0.852	-0.740	0.216	PA14_51890	172.070	-0.174	0.583
PA14_51900	5198.634	0.336	0.184	0.558	0.010	0.565	0.043	PA14_51900	7685.791	0.749	0.000
PA14_51910	1244.595	0.136	0.693	0.106	0.713	-0.561	0.085	PA14_51910	1289.691	1.228	0.000
PA14_51920	62.211	0.196	0.760	0.991	0.020	0.268	0.699	PA14_51920	283.705	-0.423	0.136
PA14_51930	322.086	-0.139	0.775	-0.127	0.746	-0.519	0.264	PA14_51930	982.017	-0.058	0.866
PA14_51940	53.269	-0.855	0.105	-1.075	0.020	-1.920	0.003	PA14_51940	200.791	-0.449	0.553
PA14_51950	263.105	-0.492	0.116	-1.190	0.000	-1.826	0.000	PA14_51950	749.931	0.503	0.034
PA14_51960	316.083	-0.379	0.238	-0.645	0.016	-0.272	0.492	PA14_51960	891.155	-0.295	0.222
PA14_51980	491.699	-0.065	0.890	0.213	0.524	-0.132	0.782	PA14_51980	1903.327	-0.106	0.669
PA14_51990	472.177	0.750	0.012	0.991	0.000	0.361	0.365	PA14_51990	1045.014	0.069	0.782
PA14_52000	219.094	0.794	0.027	0.876	0.007	1.049	0.011	PA14_52000	318.684	0.811	0.002
PA14_52010	2922.427	1.348	0.000	2.351	0.000	0.517	0.190	PA14_52010	3717.375	1.561	0.000
PA14_52020	723.148	0.326	0.255	0.501	0.037	-0.768	0.014	PA14_52020	1338.618	0.693	0.002
PA14_52040	1265.424	0.609	0.043	1.617	0.000	1.595	0.000	PA14_52040	1314.911	1.346	0.000
PA14_52050	2139.216	0.978	0.029	1.328	0.001	0.965	0.067	PA14_52050	7649.506	-0.527	0.131
PA14_52060	2444.801	-0.078	0.828	-0.096	0.731	-0.548	0.077	PA14_52060	7632.386	-0.500	0.019
PA14_52070	321.084	-0.469	0.163	-0.509	0.084	-0.243	0.575	PA14_52070	1761.099	-1.179	0.000
PA14_52080	551.643	0.535	0.068	0.598	0.023	-1.074	0.002	PA14_52080	1049.861	-0.732	0.001
PA14_52090	114.721	0.874	0.034	1.589	0.000	0.470	0.390	PA14_52090	281.082	-0.696	0.011
PA14_52110	91.765	1.379	0.001	1.680	0.000	0.734	0.185	PA14_52110	167.822	-0.042	0.894
PA14_52120	202.517	0.588	0.143	1.168	0.001	-0.436	0.399	PA14_52120	520.976	-1.976	0.000
PA14_52130	4000.628	-0.517	0.036	-0.237	0.329	0.344	0.265	PA14_52130	5582.267	0.320	0.145
PA14_52140	1328.460	0.756	0.003	0.726	0.002	0.055	0.892	PA14_52140	1859.155	0.194	0.380
PA14_52150	338.776	-0.500	0.275	-0.813	0.034	0.372	0.495	PA14_52150	1139.331	0.069	0.781
PA14_52160	1734.750	0.274	0.447	0.318	0.294	-0.857	0.017	PA14_52160	2611.109	-0.426	0.172
PA14_52180	5921.184	0.248	0.534	0.320	0.329	0.179	0.702	PA14_52180	7138.528	-0.136	0.571
PA14_52190	1919.082	0.327	0.414	0.282	0.418	1.263	0.001	PA14_52190	3214.999	-0.511	0.017
PA14_52210	2711.778	0.948	0.002	1.163	0.000	1.732	0.000	PA14_52210	4226.006	0.949	0.000
PA14_52230	414.234	-0.799	0.007	0.991	0.000	0.469	0.195	PA14_52230	402.525	-1.214	0.000
PA14_52240	455.588	-0.421	0.345	1.092	0.002	0.476	0.334	PA14_52240	291.022	-0.246	0.389
PA14_52250	149.176	-0.943	0.068	-0.066	0.908	-0.204	0.775	PA14_52250	200.976	-1.296	0.008
PA14_52260	707.457	-0.559	0.047	-0.430	0.099	-0.041	0.924	PA14_52260	1909.007	-0.877	0.000
PA14_52270	114.194	-1.409	0.000	-1.859	0.000	-1.098	0.013	PA14_52270	312.972	-1.259	0.003
PA14_52280	623.449	0.719	0.025	1.199	0.000	1.444	0.000	PA14_52280	939.447	0.412	0.097
PA14_52290	135.368	0.079	0.885	0.013	0.976	0.526	0.264	PA14_52290	628.905	-0.866	0.000

PA14_52300	159.742	0.053	0.920	0.575	0.077	2.532	0.000	PA14_52300	227.284	0.713	0.012
PA14_52310	142.314	0.367	0.373	1.710	0.000	-0.125	0.825	PA14_52310	339.449	0.987	0.000
PA14_52320	58.878	-0.004	0.996	2.236	0.000	2.303	0.000	#N/A	#N/A	#N/A	#N/A
PA14_52330	621.136	0.511	0.064	1.141	0.000	1.015	0.001	PA14_52330	1831.987	-0.991	0.000
PA14_52340	305.007	-2.335	0.000	0.743	0.097	-3.824	0.000	PA14_52340	1063.527	0.280	0.283
PA14_52350	509.282	-1.080	0.000	-0.564	0.050	-0.981	0.006	PA14_52350	4126.912	0.204	0.369
PA14_52370	285.278	-1.258	0.000	-0.674	0.026	-1.368	0.000	PA14_52370	920.002	-0.361	0.346
PA14_52380	281.211	-1.400	0.000	-2.222	0.000	-0.863	0.023	PA14_52380	1437.388	-2.050	0.000
PA14_52400	358.975	-0.338	0.289	-0.032	0.923	0.171	0.671	PA14_52400	1573.606	-1.617	0.000
PA14_52420	661.877	0.433	0.161	1.144	0.000	1.680	0.000	PA14_52420	933.361	-0.640	0.004
PA14_52430	115.060	0.976	0.009	1.289	0.000	1.202	0.005	PA14_52430	145.837	-0.489	0.525
PA14_52440	14.060	0.751	0.382	1.069	0.120	1.392	0.099	PA14_52440	13.051	0.959	0.317
PA14_52460	1286.280	0.340	0.288	0.656	0.013	0.243	0.532	PA14_52460	5707.470	-2.119	0.000
PA14_52465	84.607	1.052	0.048	0.804	0.105	0.385	0.597	PA14_52465	237.002	-0.734	0.133
PA14_52480	47.794	-1.361	0.002	-1.847	0.000	-1.590	0.005	PA14_52480	2226.376	-5.298	0.000
PA14_52490	94.360	-0.847	0.047	-0.322	0.432	-2.623	0.000	PA14_52490	2356.109	-4.961	0.000
PA14_52500	9.985	-1.471	0.095	-0.184	0.831	-1.304	0.202	PA14_52500	423.946	-4.858	0.000
PA14_52510	7.120	-0.780	0.511	-0.022	0.984	0.599	0.619	PA14_52510	255.110	-3.855	0.000
PA14_52520	175.926	-0.001	0.999	0.845	0.003	-0.464	0.291	PA14_52520	1544.385	-3.320	0.000
PA14_52530	2421.671	0.807	0.449	1.432	0.097	0.198	0.876	PA14_52530	4189.132	-0.445	0.424
PA14_52540	38.667	1.324	0.030	2.569	0.000	1.128	0.125	#N/A	#N/A	#N/A	#N/A
PA14_52550	686.421	0.613	0.085	2.367	0.000	2.013	0.000	#N/A	#N/A	#N/A	#N/A
PA14_52560	5657.039	0.505	0.222	2.283	0.000	1.675	0.000	#N/A	#N/A	#N/A	#N/A
PA14_52570	19747.009	-0.731	0.500	-0.097	0.927	0.535	0.650	PA14_52570	67831.732	-1.258	0.000
PA14_52580	3845.640	0.299	0.379	0.447	0.113	-0.532	0.143	PA14_52580	3485.583	0.382	0.092
PA14_52600	2194.529	0.314	0.248	0.402	0.086	-0.339	0.285	PA14_52600	2620.853	0.798	0.000
PA14_52610	273.628	0.787	0.041	1.563	0.000	0.056	0.926	PA14_52610	450.496	1.252	0.000
PA14_52630	1063.585	-0.257	0.421	-0.824	0.001	-1.194	0.000	PA14_52630	3672.490	-1.060	0.000
PA14_52640	1541.915	-0.434	0.107	-1.074	0.000	-1.420	0.000	PA14_52640	5133.781	-1.012	0.000
PA14_52660	3435.875	-0.518	0.064	-1.522	0.000	-1.123	0.000	PA14_52660	8676.897	-1.377	0.000
PA14_52670	865.386	-0.112	0.760	-0.823	0.001	-2.533	0.000	PA14_52670	2109.190	-0.985	0.000
PA14_52690	1954.459	-0.333	0.235	-1.571	0.000	-3.044	0.000	PA14_52690	3329.014	-0.515	0.019
PA14_52700	1597.618	0.489	0.060	-0.265	0.292	-1.771	0.000	PA14_52700	2899.205	-0.581	0.008
PA14_52720	3745.822	-0.418	0.102	-1.404	0.000	-2.551	0.000	PA14_52720	7732.912	-0.104	0.647
PA14_52730	19.703	-0.562	0.488	-0.327	0.635	0.374	0.678	PA14_52730	104.837	-1.604	0.000
PA14_52740	808.456	0.531	0.082	0.089	0.785	-0.093	0.838	PA14_52740	1360.772	-0.482	0.029
PA14_52750	1599.804	0.506	0.061	-0.156	0.573	-0.382	0.257	PA14_52750	2107.301	-0.049	0.847
PA14_52760	4033.063	1.082	0.001	0.933	0.003	0.940	0.018	PA14_52760	4177.117	-0.410	0.053
PA14_52770	980.707	0.491	0.071	0.436	0.081	0.302	0.384	PA14_52770	1566.666	-0.524	0.018
PA14_52780	557.448	-0.041	0.920	0.299	0.269	0.528	0.116	PA14_52780	1282.698	-0.138	0.588
PA14_52790	7023.674	0.353	0.341	0.197	0.564	0.669	0.088	PA14_52790	7335.837	0.361	0.163
PA14_52800	1400.279	1.557	0.000	0.294	0.491	-0.958	0.052	PA14_52800	1638.529	0.338	0.131
PA14_52810	159.083	3.880	0.000	0.992	0.007	1.035	0.035	PA14_52810	105.129	0.580	0.108
PA14_52820	28.286	4.080	0.000	1.375	0.042	1.513	0.077	PA14_52820	87.757	-1.068	0.308
PA14_52840	216.913	5.233	0.000	-0.850	0.072	0.707	0.220	PA14_52840	80.013	-0.215	0.609
PA14_52850	302.969	6.723	0.000	1.274	0.011	2.286	0.000	PA14_52850	37.536	0.534	0.328
PA14_52870	432.331	7.345	0.000	0.849	0.076	1.806	0.001	PA14_52870	57.766	2.160	0.000
PA14_52880	213.322	6.574	0.000	-0.231	0.729	0.995	0.159	PA14_52880	58.869	2.388	0.000
PA14_52890	438.109	7.409	0.000	1.423	0.002	2.975	0.000	PA14_52890	92.561	1.609	0.000
PA14_52900	1738.119	6.983	0.000	1.042	0.001	1.968	0.000	PA14_52900	171.231	1.264	0.000
PA14_52910	1711.017	6.854	0.000	1.516	0.000	2.794	0.000	PA14_52910	188.925	0.452	0.155
PA14_52920	138.082	1.866	0.000	1.863	0.000	1.478	0.001	PA14_52920	300.819	0.384	0.162
PA14_52930	176.528	0.637	0.082	0.070	0.862	-1.176	0.010	PA14_52930	431.224	0.745	0.003
PA14_52940	41.407	-0.229	0.746	-0.432	0.416	-0.286	0.709	PA14_52940	88.941	0.097	0.836
PA14_52960	101.988	2.173	0.000	2.837	0.000	0.608	0.351	PA14_52960	116.572	-0.175	0.663
PA14_52980	438.273	0.481	0.170	0.229	0.499	-1.072	0.007	PA14_52980	889.884	0.838	0.000
PA14_52990	8862.790	-0.509	0.133	-3.075	0.000	-0.379	0.361	PA14_52990	24710.250	-1.507	0.000
PA14_53000	5435.284	0.591	0.057	-2.158	0.000	-0.185	0.667	PA14_53000	12675.343	-0.449	0.039
PA14_53010	2676.768	-0.022	0.963	-2.789	0.000	-0.749	0.035	PA14_53010	7303.216	0.224	0.334

PA14_53020	2022.200	0.350	0.369	0.101	0.788	0.410	0.348	PA14_53020	5008.317	-0.906	0.000
PA14_53030	152.086	0.745	0.038	0.769	0.017	1.043	0.011	PA14_53030	176.487	0.601	0.052
PA14_53040	632.142	-1.119	0.000	-1.752	0.000	-3.662	0.000	PA14_53040	1844.960	-1.158	0.000
PA14_53050	447.654	1.073	0.000	-3.669	0.000	-0.829	0.031	PA14_53050	231.189	-0.442	0.135
PA14_53070	4462.056	0.206	0.646	-6.263	0.000	-1.165	0.004	PA14_53070	5536.121	-1.767	0.000
PA14_53090	27.667	0.558	0.455	0.660	0.277	1.439	0.040	PA14_53090	34.094	-0.549	0.334
PA14_53100	112.153	0.854	0.050	-0.875	0.033	0.429	0.454	PA14_53100	136.671	-0.950	0.055
PA14_53110	60.313	-0.104	0.862	-1.257	0.001	-0.219	0.721	PA14_53110	107.903	-0.449	0.237
PA14_53120	1117.074	-0.292	0.370	-0.963	0.000	-0.115	0.783	PA14_53120	4566.623	-0.438	0.044
PA14_53140	1954.623	-0.413	0.197	-1.062	0.000	-0.049	0.915	PA14_53140	8026.038	-0.587	0.005
PA14_53150	710.759	0.029	0.951	0.421	0.160	1.096	0.002	PA14_53150	1288.835	-0.071	0.795
PA14_53160	141.681	0.072	0.902	0.654	0.075	1.778	0.000	PA14_53160	587.344	-0.759	0.020
PA14_53180	1905.517	1.077	0.000	1.411	0.000	2.618	0.000	PA14_53180	2948.957	0.449	0.041
PA14_53190	1087.890	-1.111	0.000	-1.062	0.000	-0.686	0.075	PA14_53190	4128.269	0.375	0.100
PA14_53200	4081.920	-0.465	0.130	-0.490	0.076	0.077	0.862	PA14_53200	12313.256	0.338	0.198
PA14_53210	330.437	-0.395	0.260	-1.316	0.000	-0.217	0.623	PA14_53210	1010.849	-0.532	0.044
PA14_53220	922.778	-0.992	0.000	-1.435	0.000	-0.632	0.049	PA14_53220	3793.602	-1.166	0.000
PA14_53230	101.468	-0.850	0.030	-1.108	0.001	-0.620	0.204	PA14_53230	412.860	-0.668	0.013
PA14_53250	3204.880	-3.698	0.000	-5.992	0.000	-1.835	0.000	PA14_53250	6678.584	-3.747	0.000
PA14_53260	182.613	0.260	0.501	-0.155	0.648	-0.830	0.043	PA14_53260	1196.651	-0.113	0.686
PA14_53270	12.729	-0.652	0.514	-0.230	0.794	-1.134	0.275	PA14_53270	24.541	-0.384	0.627
PA14_53290	116.668	-1.190	0.011	-1.420	0.001	-0.330	0.607	PA14_53290	3263.962	-4.615	0.000
PA14_53300	99.196	-0.862	0.028	-0.288	0.447	0.305	0.549	PA14_53300	8340.511	-6.431	0.000
PA14_53310	227.703	-0.776	0.010	-1.177	0.000	0.575	0.108	PA14_53310	886.199	-0.950	0.000
PA14_53330	179.574	-0.136	0.761	0.067	0.857	-0.326	0.479	PA14_53330	572.956	0.180	0.497
PA14_53340	70.480	-0.603	0.185	-0.819	0.035	0.111	0.859	PA14_53340	180.744	0.318	0.389
PA14_53360	57.855	-0.576	0.234	-1.231	0.002	0.542	0.314	PA14_53360	414.346	-1.371	0.000
PA14_53370	92.404	-0.610	0.138	-0.942	0.007	0.932	0.030	PA14_53370	762.667	-1.381	0.000
PA14_53380	12.196	-0.276	0.794	-0.691	0.373	0.107	0.925	PA14_53380	1576.692	-6.250	0.000
PA14_53390	380.875	1.131	0.001	0.650	0.051	0.731	0.089	PA14_53390	548.410	0.397	0.390
PA14_53400	197.813	0.905	0.016	1.694	0.000	3.519	0.000	PA14_53400	158.273	1.591	0.000
PA14_53410	504.078	2.588	0.000	3.572	0.000	4.230	0.000	PA14_53410	401.038	0.182	0.521
PA14_53420	1226.142	-0.232	0.674	0.738	0.068	2.243	0.000	PA14_53420	1882.254	-1.636	0.000
PA14_53430	3441.044	-0.699	0.025	0.110	0.745	0.968	0.007	PA14_53430	5308.204	-0.126	0.681
PA14_53450	2113.603	0.515	0.274	-1.557	0.000	-1.978	0.000	PA14_53450	10207.294	-2.290	0.000
PA14_53470	1429.867	-0.925	0.000	-2.498	0.000	-2.125	0.000	PA14_53470	8541.655	-0.913	0.000
PA14_53480	1315.182	-0.889	0.000	-2.139	0.000	-1.927	0.000	PA14_53480	11556.183	0.017	0.980
PA14_53490	463.806	0.157	0.660	-0.307	0.265	-0.409	0.258	PA14_53490	3847.652	1.789	0.000
PA14_53500	2447.881	-0.003	0.995	0.316	0.302	0.682	0.063	PA14_53500	9661.039	0.943	0.000
PA14_53510	152.355	0.627	0.098	0.469	0.180	-0.386	0.453	PA14_53510	327.613	0.026	0.910
PA14_53520	161.742	1.175	0.001	1.414	0.000	0.126	0.825	PA14_53520	300.233	2.243	0.000
PA14_53530	1635.431	-3.328	0.000	-5.024	0.000	-4.477	0.000	PA14_53530	6842.428	-3.538	0.000
PA14_53540	37.376	0.859	0.164	1.101	0.036	0.175	0.848	PA14_53540	108.013	-0.042	0.945
PA14_53550	64.330	0.733	0.123	1.209	0.002	1.539	0.002	PA14_53550	190.039	-0.062	0.842
PA14_53560	254.080	0.211	0.584	0.347	0.253	0.143	0.755	PA14_53560	704.377	-0.068	0.849
PA14_53570	297.422	0.046	0.928	0.196	0.583	0.013	0.980	PA14_53570	1290.700	-0.503	0.025
PA14_53580	1505.469	-0.395	0.156	-1.086	0.000	-1.417	0.000	PA14_53580	9862.304	-0.871	0.000
PA14_53590	3803.716	-0.712	0.016	-2.274	0.000	-2.984	0.000	PA14_53590	13180.320	-0.240	0.470
PA14_53600	551.450	-0.680	0.036	-2.371	0.000	-1.917	0.000	PA14_53600	2276.692	-0.272	0.275
PA14_53610	857.657	0.137	0.755	-1.128	0.000	-0.748	0.061	PA14_53610	2633.514	-0.720	0.220
PA14_53620	104.730	-0.910	0.033	-2.436	0.000	-0.119	0.849	PA14_53620	271.056	-0.542	0.195
PA14_53630	15.951	-0.483	0.578	-0.789	0.250	-0.062	0.952	PA14_53630	68.217	-0.022	0.952
PA14_53640	13.114	1.463	0.080	0.936	0.238	1.987	0.023	PA14_53640	27.922	0.775	0.191
PA14_53650	259.096	0.337	0.447	0.157	0.696	-1.138	0.013	PA14_53650	912.374	-0.996	0.000
PA14_53660	549.546	0.098	0.823	-0.485	0.107	-0.832	0.028	PA14_53660	2111.483	-0.737	0.001
PA14_53670	2492.968	-0.049	0.923	-0.498	0.127	-0.912	0.022	PA14_53670	9110.666	-0.771	0.000
PA14_53680	21.272	0.833	0.260	1.197	0.048	0.095	0.929	PA14_53680	186.344	-0.456	0.297
PA14_53690	8.158	-0.630	0.597	0.287	0.770	0.620	0.597	PA14_53690	22.698	-0.294	0.746
PA14_53700	52.457	-0.172	0.778	-0.210	0.655	-0.670	0.280	PA14_53700	367.888	-0.378	0.156

PA14_53720	104.490	-0.042	0.943	0.469	0.203	-0.736	0.153	PA14_53720	300.686	0.114	0.744
PA14_53730	211.281	-0.716	0.062	-0.969	0.004	-1.605	0.000	PA14_53730	1234.464	-1.552	0.000
PA14_53740	4.201	-1.761	0.125	-1.487	0.145	-1.246	0.307	PA14_53740	21.858	-0.723	0.303
PA14_53750	21.051	-0.830	0.250	-0.576	0.357	-0.367	0.685	PA14_53750	74.959	-0.238	0.625
PA14_53770	45.568	-0.086	0.917	0.489	0.357	0.690	0.309	PA14_53770	109.357	-0.177	0.630
PA14_53780	287.141	-0.489	0.171	-0.341	0.295	-0.939	0.020	PA14_53780	1067.577	-0.734	0.001
PA14_53790	38.046	-0.542	0.358	-0.791	0.101	-0.393	0.586	PA14_53790	189.076	-1.025	0.002
PA14_53800	40.221	0.554	0.351	0.399	0.443	0.495	0.480	PA14_53800	107.336	0.225	0.567
PA14_53810	27.788	-0.872	0.231	-1.344	0.029	-1.348	0.101	PA14_53810	202.868	-2.121	0.000
PA14_53820	1532.584	-0.222	0.565	-0.746	0.010	-2.945	0.000	PA14_53820	10154.296	-1.948	0.000
PA14_53830	40.151	0.030	0.974	-0.132	0.831	1.212	0.053	PA14_53830	155.547	-0.110	0.755
PA14_53840	2851.001	-0.680	0.175	0.301	0.539	1.048	0.057	PA14_53840	9187.671	0.401	0.060
PA14_53850	184.093	-1.126	0.003	-1.490	0.000	-1.018	0.027	PA14_53850	776.987	-1.322	0.000
PA14_53860	76.774	-1.426	0.002	-0.920	0.029	0.368	0.539	PA14_53860	128.485	-2.051	0.000
PA14_53870	18.909	-0.251	0.789	0.673	0.289	0.197	0.847	PA14_53870	8.646	1.088	0.366
PA14_53880	122.077	-0.589	0.176	0.065	0.884	-2.066	0.000	PA14_53880	90.224	-0.019	0.996
PA14_53890	5.839	0.476	0.721	0.932	0.348	-0.089	0.951	PA14_53890	4.779	0.519	0.787
PA14_53900	470.382	0.545	0.059	0.825	0.001	0.295	0.434	PA14_53900	460.317	0.863	0.000
PA14_53910	274.472	-1.582	0.000	-2.306	0.000	1.152	0.001	PA14_53910	356.357	-0.252	0.521
PA14_53920	308.185	-1.030	0.012	-0.255	0.550	0.528	0.307	PA14_53920	871.243	2.456	0.000
PA14_53940	3282.252	-1.309	0.000	-2.194	0.000	-1.480	0.001	PA14_53940	2304.291	0.657	0.002
PA14_53950	1703.462	-0.837	0.065	-2.635	0.000	-2.226	0.000	PA14_53950	1429.541	0.479	0.167
PA14_53970	1467.150	-0.864	0.036	-2.196	0.000	-2.044	0.000	PA14_53970	1520.940	-0.096	0.728
PA14_53980	651.390	-0.833	0.056	-1.749	0.000	-1.692	0.000	PA14_53980	1032.032	0.224	0.336
PA14_54000	514.168	-0.993	0.034	-2.317	0.000	-1.113	0.041	PA14_54000	738.050	-0.442	0.076
PA14_54010	41.123	-0.273	0.677	-0.239	0.650	-1.295	0.061	PA14_54010	135.513	-0.737	0.021
PA14_54030	8.306	0.026	0.985	0.777	0.407	-0.957	0.434	PA14_54030	12.104	1.073	0.258
PA14_54040	1036.131	1.705	0.000	1.207	0.000	0.910	0.021	PA14_54040	860.619	3.136	0.000
PA14_54050	69.668	-0.247	0.668	0.378	0.374	1.476	0.002	PA14_54050	405.277	-0.821	0.001
PA14_54070	44.172	0.085	0.910	0.201	0.705	0.154	0.842	PA14_54070	382.979	-1.463	0.000
PA14_54080	210.361	-2.495	0.000	-3.277	0.000	0.826	0.027	PA14_54080	2253.688	-4.739	0.000
PA14_54090	108.205	-0.018	0.976	-0.295	0.456	-1.460	0.004	PA14_54090	177.234	-0.003	0.985
PA14_54110	7.215	1.127	0.315	1.835	0.041	1.333	0.242	PA14_54110	12.001	1.735	0.067
PA14_54120	13.013	0.173	0.878	0.597	0.429	-0.045	0.970	PA14_54120	25.802	1.684	0.007
PA14_54130	319.288	0.293	0.428	-0.061	0.865	-0.800	0.041	PA14_54130	923.789	0.622	0.006
PA14_54150	730.698	0.366	0.218	0.002	0.995	0.237	0.523	PA14_54150	2452.438	4.644	0.000
PA14_54170	661.412	-1.076	0.000	-0.924	0.000	1.151	0.000	PA14_54170	3262.664	3.011	0.000
PA14_54180	433.151	-0.208	0.699	0.300	0.446	-0.170	0.793	PA14_54180	186.002	-2.512	0.000
PA14_54190	69.811	-0.574	0.269	-0.946	0.028	-0.178	0.793	PA14_54190	243.170	0.026	0.984
PA14_54210	4934.911	-0.812	0.006	-0.070	0.837	-2.441	0.000	PA14_54210	14925.250	1.454	0.000
PA14_54220	271.571	-0.250	0.568	0.685	0.036	-1.402	0.001	PA14_54220	620.930	2.819	0.000
PA14_54230	161.595	0.013	0.980	0.711	0.019	0.001	0.998	PA14_54230	343.473	0.225	0.435
PA14_54240	30.719	-2.145	0.000	-1.512	0.004	-0.186	0.820	PA14_54240	252.119	-3.489	0.000
PA14_54260	163.502	1.194	0.002	1.540	0.000	1.256	0.007	PA14_54260	172.707	1.074	0.000
PA14_54270	105.432	0.020	0.974	0.438	0.243	-0.705	0.169	PA14_54270	83.572	1.615	0.000
PA14_54290	259.989	-0.651	0.105	-0.949	0.007	-1.506	0.001	PA14_54290	665.995	0.607	0.012
PA14_54300	440.728	0.774	0.104	1.607	0.000	1.163	0.027	PA14_54300	716.817	0.714	0.022
PA14_54320	4480.783	0.647	0.049	1.019	0.000	0.861	0.022	PA14_54320	3863.932	0.010	0.989
PA14_54330	709.619	0.162	0.622	0.442	0.073	1.007	0.001	PA14_54330	1724.235	-0.835	0.000
PA14_54340	2494.816	-0.642	0.041	-0.245	0.440	0.256	0.542	PA14_54340	10490.975	-2.373	0.000
PA14_54350	1732.278	0.181	0.639	1.151	0.000	0.388	0.324	PA14_54350	1533.622	-0.129	0.641
PA14_54370	8151.634	0.549	0.098	1.292	0.000	1.205	0.001	PA14_54370	8560.547	0.522	0.014
PA14_54390	6945.778	-0.336	0.239	-0.065	0.825	-0.495	0.119	PA14_54390	9780.327	1.396	0.000
PA14_54400	1932.725	0.299	0.491	0.873	0.009	0.822	0.056	PA14_54400	4609.827	1.051	0.000
PA14_54410	1259.596	-0.600	0.076	-0.292	0.374	0.823	0.031	PA14_54410	9334.260	0.135	0.583
PA14_54420	4946.398	0.213	0.602	0.895	0.003	2.399	0.000	PA14_54420	28799.484	-1.227	0.000
PA14_54430	9971.223	-0.364	0.316	0.012	0.973	1.733	0.000	PA14_54430	58733.701	-0.600	0.004
PA14_54450	1258.541	0.091	0.802	0.417	0.093	0.678	0.028	PA14_54450	3428.918	-0.262	0.243
PA14_54460	74.500	0.626	0.217	1.577	0.000	1.929	0.000	PA14_54460	112.949	-0.672	0.050

PA14_54470	203.265	-0.069	0.896	0.640	0.052	-0.355	0.471	PA14_54470	434.371	-0.534	0.029
PA14_54480	427.816	0.424	0.214	0.388	0.202	0.365	0.372	PA14_54480	1119.000	0.018	0.955
PA14_54490	1818.022	0.269	0.526	0.324	0.359	2.595	0.000	PA14_54490	1402.574	1.572	0.000
PA14_54500	305.486	1.422	0.000	1.552	0.000	0.560	0.163	PA14_54500	227.394	0.495	0.097
PA14_54510	116.660	1.008	0.011	1.583	0.000	0.078	0.906	PA14_54510	168.094	0.766	0.041
PA14_54520	7561.828	8.126	0.000	5.897	0.000	4.269	0.000	PA14_54520	119.904	0.170	0.658
PA14_54540	2489.442	6.266	0.000	3.748	0.000	1.022	0.023	PA14_54540	88.302	-0.359	0.515
PA14_54550	219.222	6.500	0.000	3.099	0.000	1.616	0.028	PA14_54550	11.646	1.303	0.176
PA14_54570	923.649	6.602	0.000	3.815	0.000	1.403	0.009	PA14_54570	49.977	-0.442	0.494
PA14_54580	277.141	5.628	0.000	2.587	0.000	1.278	0.060	PA14_54580	11.217	1.418	0.144
PA14_54590	693.514	-0.339	0.360	-0.005	0.990	-1.523	0.000	PA14_54590	1119.398	0.232	0.342
PA14_54600	85.588	-0.023	0.972	-0.372	0.342	-0.186	0.752	PA14_54600	227.858	0.492	0.090
PA14_54610	35.517	0.944	0.088	0.676	0.186	-0.143	0.872	PA14_54610	139.390	0.477	0.150
PA14_54620	1753.471	-0.908	0.014	-2.306	0.000	-2.230	0.000	PA14_54620	1274.924	-1.084	0.000
PA14_54630	1407.799	-0.914	0.000	-3.605	0.000	-3.421	0.000	PA14_54630	1240.927	-0.782	0.002
PA14_54640	1869.151	-0.778	0.009	-3.074	0.000	-3.557	0.000	PA14_54640	1299.852	-0.514	0.027
PA14_54660	889.851	-0.967	0.000	-3.437	0.000	-3.833	0.000	PA14_54660	567.187	-0.154	0.605
PA14_54670	185.340	-0.791	0.012	-2.288	0.000	0.016	0.973	PA14_54670	1059.089	-3.937	0.000
PA14_54680	56.122	-0.768	0.158	-0.964	0.040	0.794	0.179	PA14_54680	300.553	-2.288	0.000
PA14_54690	16.747	-0.634	0.496	-1.284	0.084	-0.223	0.842	PA14_54690	84.473	-1.951	0.000
PA14_54700	105.599	1.681	0.001	1.296	0.006	2.735	0.000	PA14_54700	170.383	2.414	0.000
PA14_54710	53.732	0.916	0.047	0.813	0.052	0.640	0.276	PA14_54710	183.472	-0.706	0.017
PA14_54720	1.092	1.064	0.464	0.033	0.981	1.611	0.173	PA14_54720	14.338	-1.077	0.215
PA14_54730	7.142	-0.224	0.871	0.036	0.973	0.748	0.520	PA14_54730	19.699	-0.710	0.325
PA14_54740	7.817	-0.594	0.632	-0.838	0.398	2.145	0.027	PA14_54740	97.414	-1.960	0.000
PA14_54750	16.782	-0.248	0.834	-0.138	0.883	2.870	0.000	PA14_54750	163.665	-2.399	0.000
PA14_54760	108.640	0.047	0.929	-0.220	0.553	-1.221	0.009	PA14_54760	374.473	-0.643	0.014
PA14_54770	79.899	0.096	0.866	-0.554	0.149	-0.418	0.452	PA14_54770	195.184	-0.348	0.257
PA14_54790	113.184	-0.116	0.843	0.286	0.481	2.321	0.000	PA14_54790	192.163	0.522	0.082
PA14_54800	147.461	0.204	0.655	0.425	0.213	2.538	0.000	PA14_54800	252.442	0.545	0.046
PA14_54810	99.638	-1.396	0.001	-2.287	0.000	-0.377	0.502	PA14_54810	1083.641	-2.826	0.000
PA14_54820	355.257	-0.675	0.024	-1.355	0.000	0.880	0.008	PA14_54820	907.901	-0.255	0.282
PA14_54830	2073.193	-0.058	0.885	-0.001	0.998	1.296	0.000	PA14_54830	1157.084	0.497	0.172
PA14_54840	542.482	1.438	0.001	3.700	0.000	2.454	0.000	#N/A	#N/A	#N/A	#N/A
PA14_54850	839.277	1.420	0.000	2.976	0.000	2.375	0.000	PA14_54850	1749.980	0.274	0.226
PA14_54860	71.330	-0.474	0.489	-0.761	0.164	0.647	0.359	PA14_54860	318.790	-0.948	0.000
PA14_54870	193.009	0.036	0.949	-0.873	0.012	0.164	0.760	PA14_54870	632.085	-1.051	0.000
PA14_54880	103.286	-0.301	0.566	-0.282	0.517	-0.172	0.785	PA14_54880	643.220	-0.671	0.005
PA14_54890	498.084	0.496	0.163	0.568	0.070	0.777	0.049	PA14_54890	1542.361	0.060	0.812
PA14_54900	287.565	0.758	0.021	1.000	0.001	0.543	0.188	PA14_54900	1105.653	0.367	0.112
PA14_54910	176.798	0.136	0.778	0.136	0.723	0.523	0.244	PA14_54910	748.945	-0.638	0.006
PA14_54920	374.348	-0.138	0.705	-0.717	0.005	-0.270	0.479	PA14_54920	1587.798	-0.985	0.000
PA14_54930	217.641	-0.488	0.158	-0.235	0.468	0.110	0.818	PA14_54930	799.701	-1.076	0.000
PA14_54940	429.079	0.179	0.596	-0.165	0.561	0.479	0.153	PA14_54940	1501.264	-0.937	0.000
PA14_54950	209.239	0.019	0.972	0.111	0.758	0.341	0.441	PA14_54950	741.218	-0.577	0.013
PA14_54960	78.587	0.361	0.478	-0.093	0.845	-0.200	0.754	PA14_54960	299.536	-0.839	0.002
PA14_54970	78.758	0.149	0.786	-0.192	0.652	0.532	0.298	PA14_54970	281.514	-0.331	0.253
PA14_54980	181.284	-0.148	0.746	-0.158	0.660	0.541	0.198	PA14_54980	542.380	-0.266	0.289
PA14_55000	25.697	-0.048	0.961	0.219	0.735	0.926	0.202	PA14_55000	75.492	-0.608	0.141
PA14_55020	22.009	-0.533	0.515	-0.838	0.201	1.456	0.040	PA14_55020	73.354	-0.165	0.760
PA14_55030	15.214	0.002	0.999	0.175	0.837	0.252	0.820	PA14_55030	45.062	0.146	0.798
PA14_55040	41.548	-0.392	0.521	-0.292	0.568	1.308	0.015	PA14_55040	77.830	-0.572	0.237
PA14_55050	2246.722	0.283	0.368	0.371	0.161	0.315	0.379	PA14_55050	5229.311	-0.081	0.728
PA14_55060	407.767	0.124	0.741	0.255	0.366	0.395	0.274	PA14_55060	1400.941	-0.540	0.015
PA14_55070	206.571	0.106	0.837	-0.441	0.219	-0.122	0.826	PA14_55070	874.014	-0.360	0.136
PA14_55080	1907.441	-1.151	0.000	-2.033	0.000	-1.170	0.000	PA14_55080	11990.330	-1.954	0.000
PA14_55090	202.607	-0.408	0.298	-0.864	0.007	-0.478	0.291	PA14_55090	1379.148	-1.315	0.000
PA14_55100	164.227	-0.768	0.111	-0.573	0.193	0.731	0.184	PA14_55100	427.016	-1.440	0.000
PA14_55110	939.221	-3.044	0.000	-5.161	0.000	-3.236	0.000	PA14_55110	3518.479	-5.147	0.000

PA14_55117	79.375	0.773	0.111	0.783	0.068	1.026	0.059
PA14_55130	22.098	0.652	0.396	0.764	0.226	0.762	0.363
PA14_55140	15.256	0.057	0.960	-0.139	0.860	-2.662	0.007
PA14_55150	192.326	0.983	0.004	1.297	0.000	1.297	0.001
PA14_55160	43.519	1.085	0.113	1.968	0.000	4.359	0.000
PA14_55170	183.359	0.312	0.461	0.754	0.019	0.868	0.036
PA14_55180	571.741	0.576	0.185	0.834	0.026	0.199	0.733
PA14_55200	86.119	-1.134	0.005	-0.859	0.020	-0.112	0.852
PA14_55220	37.893	-0.427	0.512	-0.787	0.126	0.076	0.926
PA14_55230	12.652	0.030	0.980	0.306	0.705	0.445	0.669
PA14_55240	25.254	0.333	0.654	0.311	0.606	-0.979	0.239
PA14_55250	55.433	0.646	0.238	1.444	0.001	0.342	0.631
PA14_55260	5.641	0.509	0.701	-0.273	0.814	0.729	0.563
PA14_55280	30.173	0.818	0.203	0.804	0.150	0.068	0.944
PA14_55290	11.288	-0.258	0.816	-0.096	0.915	-1.349	0.193
PA14_55300	72.001	0.378	0.505	0.656	0.139	0.968	0.081
PA14_55320	45.999	0.132	0.848	0.438	0.353	-0.028	0.972
PA14_55330	35.950	0.611	0.336	1.672	0.000	0.958	0.154
PA14_55340	15.715	-0.110	0.920	-0.168	0.837	-0.579	0.574
PA14_55360	12.655	0.391	0.726	0.919	0.253	2.023	0.023
PA14_55380	57.569	1.092	0.046	1.784	0.000	1.695	0.004
PA14_55390	25.908	-0.186	0.814	-1.026	0.061	0.058	0.946
PA14_55400	642.958	0.304	0.418	0.789	0.007	1.200	0.001
PA14_55410	39.845	-0.639	0.278	-1.411	0.004	0.445	0.512
PA14_55430	4.909	-0.489	0.734	1.443	0.136	-1.030	0.419
PA14_55440	45.726	0.376	0.544	0.280	0.595	0.258	0.729
PA14_55450	17.136	0.582	0.492	0.357	0.629	1.314	0.103
PA14_55460	1.152	-0.311	0.860	-1.358	0.267	0.579	0.680
PA14_55470	2.579	-0.226	0.900	-0.138	0.923	0.546	0.706
PA14_55480	2.181	0.708	0.644	-0.438	0.744	0.071	0.965
PA14_55490	12.936	-0.725	0.423	-1.691	0.023	-0.695	0.496
PA14_55500	10.893	-0.230	0.834	-0.838	0.279	-0.328	0.767
PA14_55510	10.526	-0.516	0.615	-1.290	0.105	0.152	0.896
PA14_55520	4.630	1.377	0.241	0.403	0.732	-0.706	0.602
PA14_55530	25.229	-0.203	0.811	-0.363	0.569	-0.425	0.627
PA14_55540	25.093	-0.283	0.756	0.701	0.260	2.422	0.000
PA14_55550	29.724	0.050	0.965	1.488	0.011	2.947	0.000
PA14_55560	64.862	-0.237	0.738	0.386	0.450	2.741	0.000
PA14_55570	101.996	-0.229	0.627	-0.987	0.004	-1.715	0.001
PA14_55580	284.890	-1.183	0.022	0.270	0.586	4.134	0.000
PA14_55590	1.739	-0.600	0.712	-0.068	0.963	-0.907	0.492
PA14_55600	11.724	0.836	0.387	0.524	0.546	0.201	0.872
PA14_55610	124.690	0.016	0.976	0.232	0.520	0.005	0.992
PA14_55620	0.064	0.008	0.989	0.001	0.998	0.035	0.944
PA14_55631	6528.230	3.767	0.001	0.182	0.899	-0.174	0.912
PA14_55634	0.000	NA	NA	NA	NA	NA	NA
PA14_55635	0.000	NA	NA	NA	NA	NA	NA
PA14_55637	0.000	NA	NA	NA	NA	NA	NA
PA14_55640	362.750	0.631	0.033	-0.138	0.661	-0.064	0.889
PA14_55650	636.457	0.194	0.539	-0.114	0.682	-0.604	0.059
PA14_55660	500.340	-0.190	0.615	0.147	0.643	-0.863	0.017
PA14_55670	1105.205	0.025	0.949	-0.140	0.608	-0.486	0.131
PA14_55690	1224.254	0.215	0.479	-0.080	0.774	-0.225	0.517
PA14_55710	199.256	0.174	0.697	0.489	0.130	0.414	0.352
PA14_55720	45.718	0.961	0.076	2.273	0.000	0.179	0.832
PA14_55730	443.972	1.082	0.001	1.138	0.000	-0.268	0.574
PA14_55740	63.995	0.004	0.995	0.210	0.642	-0.075	0.914
PA14_55750	258.985	0.914	0.058	0.990	0.023	0.470	0.451
PA14_55760	2079.687	0.282	0.447	1.162	0.000	2.468	0.000

PA14_55117	153.371	0.242	0.468
PA14_55130	49.406	-0.951	0.049
PA14_55140	47.564	-0.087	0.907
PA14_55150	374.750	1.199	0.000
PA14_55160	42.023	0.979	0.049
PA14_55170	457.122	0.682	0.006
PA14_55180	1303.621	0.019	0.917
PA14_55200	388.822	-2.079	0.000
PA14_55220	94.184	0.088	0.832
PA14_55230	30.652	0.605	0.351
PA14_55240	57.795	-0.029	1.000
PA14_55250	113.950	0.016	1.000
PA14_55260	42.597	-0.775	0.124
PA14_55280	77.686	-0.451	0.432
PA14_55290	50.929	-1.399	0.002
PA14_55300	133.306	-1.283	0.000
PA14_55320	114.049	-0.884	0.009
PA14_55330	71.664	-1.052	0.006
PA14_55340	32.596	-0.779	0.174
PA14_55360	40.762	-0.374	0.495
PA14_55380	101.595	-0.367	0.325
PA14_55390	100.131	-1.969	0.000
PA14_55400	1918.529	-0.764	0.000
PA14_55410	221.616	-2.213	0.000
PA14_55430	16.642	-1.095	0.278
PA14_55440	112.235	0.133	0.756
PA14_55450	41.097	-1.426	0.004
PA14_55460	4.897	0.943	0.565
PA14_55470	19.344	-0.183	0.865
PA14_55480	9.752	-1.100	0.303
PA14_55490	73.659	-1.153	0.003
PA14_55500	22.325	0.230	0.818
PA14_55510	43.448	-2.306	0.000
PA14_55520	19.715	-0.802	0.272
PA14_55530	113.427	-0.060	0.899
PA14_55540	78.351	-2.273	0.000
PA14_55550	122.755	-1.308	0.000
PA14_55560	289.016	-2.129	0.000
PA14_55570	727.053	-1.979	0.000
PA14_55580	53.557	-0.453	0.430
PA14_55590	14.430	-1.303	0.126
PA14_55600	46.838	-1.065	0.023
PA14_55610	428.457	-0.830	0.001
#N/A	#N/A	#N/A	#N/A
#N/A	#N/A	#N/A	#N/A
#N/A	#N/A	#N/A	#N/A
#N/A	#N/A	#N/A	#N/A
#N/A	#N/A	#N/A	#N/A
PA14_55640	1302.501	-0.341	0.146
PA14_55650	1895.986	1.146	0.000
PA14_55660	882.868	-0.906	0.000
PA14_55670	1492.687	-0.214	0.383
PA14_55690	1656.640	0.544	0.014
PA14_55710	484.423	0.436	0.081
PA14_55720	71.809	1.481	0.000
PA14_55730	559.762	-0.002	0.998
PA14_55740	194.845	-0.403	0.298
PA14_55750	474.433	-0.084	0.779
PA14_55760	4144.263	0.042	0.939

PA14_55770	2056.278	0.838	0.000	1.483	0.000	1.908	0.000	PA14_55770	1945.778	2.215	0.000
PA14_55780	139.347	-1.174	0.001	-1.036	0.002	1.488	0.000	PA14_55780	1329.307	-1.824	0.000
PA14_55790	117.629	-2.261	0.000	-2.857	0.000	1.066	0.012	PA14_55790	1306.848	-3.766	0.000
PA14_55800	153.615	-0.289	0.479	-0.590	0.064	1.030	0.006	PA14_55800	1049.919	-1.030	0.000
PA14_55810	2668.278	-1.397	0.000	-2.899	0.000	-0.983	0.001	PA14_55810	20202.748	-2.019	0.000
PA14_55820	187.316	-0.517	0.147	-0.755	0.014	-0.231	0.621	PA14_55820	1157.525	-2.115	0.000
PA14_55840	3.707	-0.484	0.750	-1.308	0.251	-1.705	0.161	PA14_55840	95.979	-4.105	0.000
PA14_55850	18.119	-2.501	0.000	-2.904	0.000	-1.271	0.114	PA14_55850	160.061	-3.250	0.000
PA14_55860	60.611	-1.289	0.003	-2.551	0.000	0.127	0.841	PA14_55860	259.456	-2.537	0.000
PA14_55880	14.115	-1.272	0.112	-1.270	0.070	-0.399	0.696	PA14_55880	99.900	-2.856	0.000
PA14_55890	30.551	-1.663	0.002	-2.518	0.000	-0.516	0.461	PA14_55890	143.035	-2.450	0.000
PA14_55900	44.142	-2.070	0.000	-2.220	0.000	-0.485	0.446	PA14_55900	233.120	-2.065	0.000
PA14_55920	161.676	-1.426	0.000	-1.034	0.003	0.915	0.034	PA14_55920	663.389	-1.535	0.000
PA14_55930	188.832	-1.164	0.000	-1.389	0.000	1.940	0.000	PA14_55930	972.191	-2.363	0.000
PA14_55940	78.990	-3.975	0.000	-5.854	0.000	1.122	0.026	PA14_55940	927.233	-6.102	0.000
PA14_55960	610.204	1.173	0.000	0.227	0.427	-0.448	0.218	PA14_55960	1279.796	0.388	0.098
PA14_55980	355.250	-0.750	0.028	-1.190	0.000	-1.709	0.000	PA14_55980	1147.996	0.131	0.607
PA14_56000	821.064	-0.978	0.000	-2.821	0.000	0.064	0.874	PA14_56000	4684.039	-1.229	0.000
PA14_56010	917.091	-0.437	0.096	-1.172	0.000	-0.631	0.036	PA14_56010	3611.533	-1.363	0.000
PA14_56030	450.613	-1.791	0.000	-1.785	0.000	0.793	0.026	PA14_56030	3823.273	-2.286	0.000
PA14_56040	771.758	-0.868	0.063	-0.029	0.956	0.583	0.307	PA14_56040	3069.826	-0.774	0.000
PA14_56050	86.692	-0.701	0.139	0.340	0.425	0.372	0.521	PA14_56050	470.900	-0.624	0.012
PA14_56060	1043.453	0.321	0.284	0.458	0.070	-0.600	0.067	PA14_56060	1784.051	0.852	0.000
PA14_56070	35008.005	-0.718	0.011	-1.357	0.000	-3.095	0.000	PA14_56070	182946.968	-1.365	0.000
PA14_56080	1458.883	0.444	0.220	0.775	0.011	-0.047	0.926	PA14_56080	2887.248	-0.546	0.010
PA14_56090	355.784	-0.154	0.792	0.086	0.858	-0.830	0.117	PA14_56090	1759.272	0.547	0.016
PA14_56100	145.370	0.967	0.011	0.863	0.014	-0.376	0.498	PA14_56100	463.013	0.940	0.000
PA14_56110	267.124	0.146	0.786	0.796	0.029	-0.495	0.342	PA14_56110	378.694	1.417	0.000
PA14_56130	476.265	0.469	0.323	0.759	0.054	0.317	0.580	PA14_56130	585.579	1.239	0.009
PA14_56140	355.243	0.046	0.916	-0.372	0.184	-1.257	0.000	PA14_56140	504.307	0.364	0.166
PA14_56160	156.867	-0.228	0.580	-1.252	0.000	-2.501	0.000	PA14_56160	400.389	-0.022	0.917
PA14_56170	166.376	-0.273	0.523	-1.406	0.000	-2.413	0.000	PA14_56170	376.182	0.934	0.000
PA14_56180	465.447	-0.706	0.052	-1.636	0.000	0.321	0.498	PA14_56180	1801.530	-1.595	0.000
PA14_56190	225.424	0.497	0.151	1.032	0.000	2.335	0.000	PA14_56190	427.431	0.295	0.278
PA14_56200	187.460	-0.752	0.019	-2.020	0.000	-0.348	0.412	PA14_56200	1154.021	-1.037	0.000
PA14_56210	580.333	0.425	0.152	0.036	0.911	-0.051	0.907	PA14_56210	1431.509	-0.711	0.002
PA14_56220	1374.710	-1.454	0.000	-3.247	0.000	-2.816	0.000	PA14_56220	4135.276	0.108	0.665
PA14_56240	3810.868	0.025	0.954	-0.588	0.025	-2.176	0.000	PA14_56240	6083.208	0.393	0.067
PA14_56250	149.961	-0.884	0.017	-0.863	0.011	-0.505	0.288	PA14_56250	548.064	-1.757	0.000
PA14_56260	99.632	-0.629	0.153	-1.347	0.000	-1.234	0.014	PA14_56260	466.031	-1.741	0.000
PA14_56280	147.662	-1.560	0.000	-2.904	0.000	-2.092	0.000	PA14_56280	716.504	-1.357	0.000
PA14_56300	2470.109	0.006	0.988	-0.194	0.470	-1.094	0.000	PA14_56300	2323.651	0.843	0.000
PA14_56340	136.392	0.008	0.987	1.089	0.000	0.008	0.988	PA14_56340	428.054	0.258	0.370
PA14_56360	35.585	-0.085	0.936	0.901	0.168	-0.519	0.587	PA14_56360	275.208	-0.799	0.003
PA14_56370	1399.994	-0.157	0.713	0.915	0.002	-1.607	0.000	PA14_56370	6492.691	-1.336	0.000
PA14_56380	68.267	0.974	0.032	0.873	0.035	1.668	0.001	PA14_56380	197.749	-0.817	0.004
PA14_56390	418.838	-0.369	0.234	-0.697	0.007	0.581	0.084	PA14_56390	1318.505	-1.166	0.000
PA14_56410	199.744	-0.263	0.487	-0.266	0.396	-0.541	0.181	PA14_56410	564.125	-1.012	0.000
PA14_56420	272.624	0.605	0.065	0.746	0.010	-0.372	0.394	PA14_56420	948.272	-0.401	0.085
PA14_56430	38.982	0.725	0.187	1.128	0.013	0.489	0.486	PA14_56430	163.470	-0.143	0.642
PA14_56450	39.709	0.079	0.918	-0.162	0.771	-0.369	0.623	PA14_56450	209.481	-0.290	0.351
PA14_56470	46.300	-0.123	0.855	-0.257	0.602	0.034	0.964	PA14_56470	324.676	-1.475	0.000
PA14_56480	45.022	0.391	0.545	1.053	0.025	1.449	0.012	PA14_56480	196.809	-0.518	0.232
PA14_56510	198.985	-0.720	0.033	-0.393	0.215	-0.002	0.998	PA14_56510	819.474	-0.928	0.000
PA14_56520	173.519	-0.713	0.034	-0.744	0.013	0.639	0.098	PA14_56520	907.776	-1.514	0.055
PA14_56530	150.499	0.009	0.987	-0.186	0.624	-1.321	0.004	PA14_56530	765.316	1.350	0.000
PA14_56540	1911.042	-0.754	0.015	-2.210	0.000	-3.488	0.000	PA14_56540	9464.029	0.404	0.063
PA14_56550	101.768	-0.924	0.020	-1.596	0.000	-1.702	0.000	PA14_56550	1018.080	0.702	0.002
PA14_56560	8.065	-0.318	0.794	-1.500	0.090	-0.751	0.522	PA14_56560	104.573	-1.060	0.098

PA14_56570	21.096	-1.608	0.026	-1.724	0.008	-0.834	0.342	PA14_56570	374.175	-0.522	0.544
PA14_56590	14811.299	-2.238	0.000	-3.507	0.000	-3.261	0.000	PA14_56590	105271.477	-1.285	0.000
PA14_56600	123.188	0.302	0.486	0.574	0.087	0.100	0.860	PA14_56600	674.157	-1.154	0.000
PA14_56620	132.386	0.155	0.758	1.264	0.000	-0.851	0.087	PA14_56620	612.748	-0.960	0.000
PA14_56640	14.962	0.416	0.679	1.346	0.054	-0.455	0.688	PA14_56640	97.724	-0.068	0.905
PA14_56660	387.135	0.177	0.643	0.981	0.000	-2.559	0.000	PA14_56660	579.477	0.364	0.136
PA14_56670	56.350	-2.552	0.000	-2.505	0.000	-1.253	0.022	PA14_56670	92.346	-0.446	0.252
PA14_56680	798.495	-2.358	0.000	-2.313	0.000	-0.975	0.002	PA14_56680	1060.468	0.017	0.908
PA14_56690	242.375	-2.180	0.000	-2.805	0.000	-1.439	0.006	PA14_56690	633.518	-0.594	0.198
PA14_56700	881.990	1.010	0.003	2.037	0.000	0.917	0.028	PA14_56700	1304.793	1.005	0.000
PA14_56720	303.311	0.142	0.744	-0.369	0.255	-1.561	0.000	PA14_56720	1034.048	-0.091	0.725
PA14_56730	401.740	-2.025	0.000	-3.362	0.000	-0.277	0.491	PA14_56730	1759.681	-2.689	0.000
PA14_56740	87.703	0.657	0.119	0.772	0.035	-0.658	0.249	PA14_56740	217.927	0.353	0.245
PA14_56750	20.343	-0.256	0.782	-0.002	0.998	0.902	0.261	PA14_56750	47.150	-0.178	0.768
PA14_56770	27.015	0.082	0.925	-0.710	0.223	1.591	0.010	PA14_56770	28.440	0.586	0.367
PA14_56780	81669.834	0.143	0.808	-0.425	0.318	-2.417	0.000	PA14_56780	128670.384	-1.122	0.000
PA14_56790	1155.218	-0.516	0.041	-1.437	0.000	-0.651	0.028	PA14_56790	5624.398	-1.851	0.000
PA14_56800	315.364	-0.141	0.746	-0.187	0.582	-0.086	0.863	PA14_56800	1262.661	-0.864	0.000
PA14_56810	134.123	-0.971	0.004	-1.092	0.000	-0.082	0.872	PA14_56810	984.585	-1.801	0.000
PA14_56830	5240.957	-1.084	0.000	-1.491	0.000	-3.725	0.000	PA14_56830	2531.954	0.256	0.574
PA14_56840	629.973	-0.199	0.577	0.598	0.025	-1.561	0.000	PA14_56840	447.257	0.514	0.205
PA14_56850	691.674	-0.574	0.048	-0.293	0.295	-2.020	0.000	PA14_56850	440.246	0.243	0.346
PA14_56870	430.299	-0.542	0.071	0.170	0.570	-2.391	0.000	PA14_56870	187.663	-0.043	0.936
PA14_56880	1019.767	0.915	0.034	1.432	0.000	-0.582	0.286	PA14_56880	2112.928	0.832	0.006
PA14_56890	844.743	-0.153	0.675	-0.498	0.063	-1.409	0.000	PA14_56890	2263.322	0.411	0.066
PA14_56900	434.538	-0.213	0.605	-0.148	0.674	-0.689	0.089	PA14_56900	1564.232	-0.469	0.037
PA14_56910	15.235	-0.862	0.293	-0.627	0.375	-1.026	0.275	PA14_56910	259.394	-1.541	0.000
PA14_56920	1337.673	0.714	0.035	1.179	0.000	1.489	0.000	PA14_56920	3609.728	-0.280	0.220
PA14_56930	436.039	0.598	0.156	1.448	0.000	2.073	0.000	PA14_56930	1271.926	-0.273	0.244
PA14_56940	290.855	0.416	0.289	1.273	0.000	1.221	0.002	PA14_56940	545.274	0.594	0.017
PA14_56950	238.220	0.168	0.692	0.748	0.011	0.623	0.115	PA14_56950	707.773	-0.174	0.529
PA14_56960	29.981	0.476	0.566	1.352	0.024	1.702	0.020	PA14_56960	84.489	-0.421	0.288
PA14_56970	779.673	1.053	0.001	1.950	0.000	2.221	0.000	PA14_56970	1968.299	-0.938	0.000
PA14_56980	202.721	0.867	0.008	1.482	0.000	2.497	0.000	PA14_56980	643.137	-0.598	0.011
PA14_56990	147.971	-2.447	0.000	-1.919	0.000	-1.667	0.001	PA14_56990	797.953	-3.212	0.000
PA14_57010	74992.356	-0.077	0.812	0.864	0.000	-2.979	0.000	PA14_57010	59113.969	2.535	0.000
PA14_57020	70306.622	0.954	0.002	2.411	0.000	-2.791	0.000	PA14_57020	54347.756	2.271	0.000
PA14_57030	480.575	0.022	0.974	0.594	0.137	0.533	0.315	PA14_57030	1701.813	2.676	0.000
PA14_57040	142.238	-0.159	0.766	-0.214	0.606	-0.439	0.414	PA14_57040	441.527	0.769	0.001
PA14_57050	872.681	0.231	0.592	-0.300	0.394	-1.687	0.000	PA14_57050	2393.023	1.027	0.000
PA14_57060	985.719	0.199	0.575	0.558	0.038	1.103	0.001	PA14_57060	1503.016	0.904	0.000
PA14_57070	259.185	0.619	0.066	0.883	0.003	-0.100	0.846	PA14_57070	458.001	1.254	0.000
PA14_57080	316.139	0.130	0.783	-0.045	0.911	0.331	0.479	PA14_57080	1213.652	-0.781	0.000
PA14_57100	174.408	-0.459	0.290	-0.559	0.129	-0.548	0.271	PA14_57100	499.941	0.535	0.035
PA14_57110	519.127	0.628	0.065	0.556	0.075	0.972	0.011	PA14_57110	990.738	0.570	0.023
PA14_57130	1594.396	-0.210	0.521	0.158	0.578	-0.369	0.288	PA14_57130	2251.221	-0.386	0.094
PA14_57140	265.732	0.294	0.505	0.314	0.391	0.297	0.551	PA14_57140	412.079	0.911	0.004
PA14_57160	65.934	-1.555	0.005	-1.964	0.000	-0.182	0.820	PA14_57160	253.586	-2.072	0.000
PA14_57170	968.105	-0.095	0.837	-0.320	0.327	-0.078	0.874	PA14_57170	1783.906	-0.916	0.000
PA14_57180	1207.672	0.306	0.589	-0.183	0.712	-0.089	0.897	PA14_57180	1729.532	-1.371	0.000
PA14_57190	366.779	0.438	0.194	0.753	0.008	-1.247	0.001	PA14_57190	714.580	0.549	0.021
PA14_57200	1529.105	0.833	0.016	1.355	0.000	0.804	0.051	PA14_57200	2407.944	0.276	0.387
PA14_57210	1953.198	-0.343	0.199	-0.954	0.000	-1.052	0.000	PA14_57210	2563.857	0.483	0.028
PA14_57220	16187.660	0.286	0.297	0.185	0.463	0.126	0.721	PA14_57220	21226.574	1.329	0.000
PA14_57240	500.195	0.364	0.295	1.189	0.000	0.868	0.016	PA14_57240	583.594	1.471	0.000
PA14_57250	74.504	-0.349	0.513	-0.088	0.856	0.828	0.106	PA14_57250	248.264	-0.545	0.049
PA14_57260	15804.461	0.538	0.263	1.582	0.000	1.594	0.001	PA14_57260	16952.697	0.541	0.011
PA14_57275	8212.897	-0.452	0.152	-0.739	0.007	-1.814	0.000	PA14_57275	26171.399	-1.214	0.000
PA14_57290	11094.478	0.516	0.107	0.745	0.008	-0.254	0.541	PA14_57290	20333.059	-0.667	0.001

PA14_57300	2905.415	-0.139	0.754	0.258	0.451	-0.622	0.128	PA14_57300	5986.416	-0.439	0.038
PA14_57320	881.377	-0.297	0.316	0.198	0.459	-1.023	0.001	PA14_57320	2590.279	-0.641	0.002
PA14_57330	1236.393	0.147	0.701	0.220	0.467	-1.030	0.003	PA14_57330	3919.564	-0.470	0.029
PA14_57340	874.671	0.093	0.845	0.652	0.038	-0.718	0.080	PA14_57340	2617.041	-0.489	0.021
PA14_57360	1417.007	0.158	0.736	0.356	0.314	-0.508	0.259	PA14_57360	4601.275	-1.004	0.000
PA14_57370	2709.946	0.431	0.184	0.651	0.020	-0.325	0.413	PA14_57370	5635.107	-0.600	0.003
PA14_57380	2931.777	0.332	0.357	0.587	0.047	-0.412	0.306	PA14_57380	6172.688	-0.289	0.175
PA14_57390	1557.098	-0.163	0.607	0.248	0.326	-0.545	0.078	PA14_57390	3725.009	0.057	0.860
PA14_57410	1650.972	0.036	0.926	0.421	0.083	-0.930	0.002	PA14_57410	4404.022	-0.111	0.680
PA14_57425	2851.269	0.442	0.097	0.986	0.000	-0.319	0.337	PA14_57425	6818.554	0.861	0.000
PA14_57440	130.227	0.144	0.770	0.898	0.006	-0.649	0.186	PA14_57440	555.287	-0.004	0.983
PA14_57450	3041.388	-0.292	0.438	0.312	0.328	-1.921	0.000	PA14_57450	9900.046	-0.571	0.006
PA14_57460	5192.575	0.153	0.734	1.051	0.001	-0.734	0.075	PA14_57460	15151.816	-0.501	0.017
PA14_57470	536.011	0.547	0.073	1.066	0.000	0.679	0.054	PA14_57470	1079.873	0.946	0.000
PA14_57480	2399.141	0.218	0.459	0.269	0.274	0.407	0.189	PA14_57480	3870.594	0.809	0.000
PA14_57490	277.495	-0.088	0.835	-0.384	0.185	0.577	0.111	PA14_57490	1086.313	-0.501	0.031
PA14_57500	1493.859	0.104	0.756	-0.539	0.021	-0.317	0.332	PA14_57500	3750.431	0.232	0.306
PA14_57510	1436.791	0.361	0.210	0.249	0.346	-0.432	0.195	PA14_57510	2697.565	0.607	0.004
PA14_57520	726.164	0.288	0.334	0.385	0.124	-1.552	0.000	PA14_57520	793.891	-0.412	0.078
PA14_57530	839.718	0.333	0.268	0.630	0.011	-0.945	0.003	PA14_57530	763.854	-0.513	0.034
PA14_57540	2319.172	-0.415	0.251	-0.694	0.023	-2.498	0.000	PA14_57540	2538.188	-0.310	0.366
PA14_57560	4509.651	-0.819	0.001	-1.543	0.000	-2.586	0.000	PA14_57560	5158.861	0.672	0.001
PA14_57570	4943.533	-0.688	0.030	-0.808	0.005	-1.789	0.000	PA14_57570	10702.127	0.320	0.157
PA14_57580	460.497	0.113	0.854	0.176	0.707	-0.627	0.251	PA14_57580	1460.516	1.753	0.000
PA14_57590	7553.870	0.647	0.026	1.256	0.000	0.897	0.007	PA14_57590	8376.388	1.924	0.000
PA14_57600	977.919	0.171	0.636	0.084	0.790	-0.411	0.263	PA14_57600	1657.034	0.513	0.019
PA14_57610	705.818	-0.688	0.027	-1.755	0.000	-1.428	0.000	#N/A	#N/A	#N/A	#N/A
PA14_57630	236.610	0.940	0.004	0.527	0.100	0.731	0.074	PA14_57630	294.163	1.267	0.000
PA14_57640	68.069	0.401	0.467	-0.100	0.847	0.148	0.836	PA14_57640	108.454	0.274	0.479
PA14_57650	4440.895	2.834	0.000	4.906	0.000	3.413	0.000	PA14_57650	1608.904	3.269	0.000
PA14_57670	899.519	0.417	0.135	0.085	0.767	-0.896	0.004	PA14_57670	2295.202	1.110	0.000
PA14_57680	514.543	0.733	0.007	1.021	0.000	-0.589	0.097	PA14_57680	1428.571	0.885	0.000
PA14_57690	1443.500	-0.569	0.093	-1.047	0.000	-1.516	0.000	PA14_57690	4465.349	-0.432	0.043
PA14_57710	1954.244	-1.960	0.000	-2.082	0.000	-3.554	0.000	PA14_57710	1343.844	0.465	0.397
PA14_57720	5202.184	-1.252	0.000	-1.173	0.000	-2.393	0.000	PA14_57720	3290.551	0.917	0.000
PA14_57730	1098.039	0.018	0.972	0.051	0.889	1.066	0.003	PA14_57730	2558.018	-0.121	0.619
PA14_57740	157.235	0.104	0.827	0.404	0.212	-1.160	0.008	PA14_57740	403.879	-0.109	0.699
PA14_57760	554.670	-0.151	0.717	-0.098	0.777	0.146	0.751	PA14_57760	1516.659	0.485	0.032
PA14_57770	270.124	0.015	0.974	0.273	0.343	-0.902	0.013	PA14_57770	498.558	-0.285	0.276
PA14_57780	644.660	0.244	0.449	0.806	0.001	0.231	0.534	PA14_57780	1323.030	-0.691	0.002
PA14_57800	269.031	-0.003	0.996	0.408	0.261	0.006	0.991	PA14_57800	394.698	1.245	0.000
PA14_57810	2915.435	0.709	0.004	1.034	0.000	0.195	0.577	PA14_57810	3204.500	1.155	0.000
PA14_57820	1009.806	0.893	0.001	1.532	0.000	1.016	0.002	PA14_57820	1617.416	0.761	0.000
PA14_57830	384.586	-0.072	0.875	0.674	0.019	-0.585	0.141	PA14_57830	700.392	-0.190	0.470
PA14_57840	2240.771	-0.733	0.014	-0.605	0.031	-1.815	0.000	PA14_57840	4084.831	0.114	0.674
PA14_57850	729.262	-0.735	0.033	-0.211	0.552	-0.841	0.038	PA14_57850	2175.356	0.214	0.393
PA14_57870	805.343	-0.239	0.621	1.571	0.000	-0.432	0.392	PA14_57870	1654.635	1.967	0.000
PA14_57880	313.349	-0.357	0.285	0.681	0.011	-0.537	0.155	PA14_57880	1413.041	1.240	0.000
PA14_57890	2987.822	0.770	0.015	1.422	0.000	1.256	0.000	PA14_57890	6664.469	0.771	0.000
PA14_57900	1768.869	0.751	0.064	1.177	0.001	1.248	0.006	PA14_57900	3105.239	1.160	0.000
PA14_57910	3681.672	0.735	0.005	1.216	0.000	1.148	0.000	PA14_57910	8327.608	0.872	0.000
PA14_57920	2853.661	0.498	0.088	0.496	0.062	0.644	0.055	PA14_57920	6312.194	0.689	0.001
PA14_57930	1210.446	0.240	0.407	0.506	0.028	0.611	0.037	PA14_57930	3047.934	0.620	0.003
PA14_57940	8401.496	0.328	0.272	-0.098	0.738	0.428	0.202	PA14_57940	23587.261	-0.101	0.725
PA14_57950	37881.445	-1.150	0.000	-2.705	0.000	-1.726	0.000	PA14_57950	242502.769	-0.853	0.000
PA14_57960	5818.796	0.531	0.099	-0.297	0.338	0.257	0.539	PA14_57960	21775.865	-0.191	0.563
PA14_57970	4460.895	-0.157	0.624	-0.370	0.133	-0.455	0.149	PA14_57970	18326.085	0.387	0.067
PA14_57980	814.640	0.078	0.833	0.072	0.805	0.183	0.619	PA14_57980	5639.454	0.591	0.008
PA14_57990	71.266	-0.181	0.839	1.127	0.038	4.318	0.000	PA14_57990	77.353	-0.101	0.912

PA14_58000	710.540	-0.453	0.278	0.642	0.050	5.714	0.000	PA14_58000	246.719	-0.486	0.105
PA14_58010	350.294	-0.810	0.108	-0.373	0.421	5.098	0.000	PA14_58010	134.215	-0.318	0.346
PA14_58030	777.102	-0.376	0.437	0.887	0.015	4.842	0.000	PA14_58030	310.338	-0.667	0.013
PA14_58040	444.828	0.921	0.065	2.400	0.000	6.048	0.000	PA14_58040	139.319	-0.607	0.067
PA14_58050	246.102	-0.119	0.810	0.457	0.181	1.238	0.002	PA14_58050	779.354	-0.141	0.586
PA14_58060	332.700	0.567	0.179	0.827	0.023	0.857	0.067	PA14_58060	1199.841	0.147	0.517
PA14_58070	1671.490	-0.260	0.379	0.268	0.289	1.113	0.000	PA14_58070	4692.862	0.538	0.010
PA14_58080	935.434	-0.047	0.906	0.522	0.039	0.947	0.002	PA14_58080	2987.322	0.002	0.983
PA14_58090	1783.165	0.171	0.623	0.586	0.023	0.444	0.199	PA14_58090	3227.016	0.059	0.843
PA14_58100	6581.354	0.425	0.431	1.180	0.005	1.681	0.001	PA14_58100	8857.710	-0.273	0.403
PA14_58110	1584.382	0.230	0.478	0.635	0.011	0.582	0.075	PA14_58110	4319.181	-0.321	0.156
PA14_58120	227.252	0.430	0.208	0.997	0.000	0.410	0.314	PA14_58120	200.811	0.963	0.007
PA14_58130	735.506	0.438	0.255	0.913	0.004	1.039	0.009	PA14_58130	816.706	1.801	0.000
PA14_58150	2487.383	1.055	0.000	1.534	0.000	1.066	0.000	PA14_58150	3503.479	1.657	0.000
PA14_58170	1539.215	1.081	0.000	1.329	0.000	1.042	0.001	PA14_58170	1934.374	2.151	0.000
PA14_58180	2394.101	0.833	0.001	0.812	0.001	0.944	0.002	PA14_58180	1870.415	1.958	0.000
PA14_58190	5185.451	0.796	0.001	1.080	0.000	0.692	0.024	PA14_58190	3185.236	1.741	0.000
PA14_58210	202.918	1.178	0.001	1.397	0.000	1.639	0.000	PA14_58210	302.425	0.337	0.216
PA14_58220	595.522	0.487	0.130	0.461	0.111	-0.138	0.758	PA14_58220	1252.896	0.807	0.001
PA14_58230	116.591	-0.988	0.004	-1.678	0.000	-2.525	0.000	PA14_58230	385.911	-0.680	0.007
PA14_58240	120.142	-0.155	0.786	-0.482	0.242	-0.434	0.447	PA14_58240	921.824	-1.023	0.000
PA14_58250	794.566	-0.445	0.164	-0.795	0.004	-0.397	0.301	PA14_58250	1982.417	-0.349	0.119
PA14_58260	807.127	-0.482	0.104	-1.496	0.000	-0.735	0.029	PA14_58260	1404.524	-0.192	0.451
PA14_58270	329.231	-0.633	0.070	-1.098	0.000	-0.823	0.043	PA14_58270	872.707	0.430	0.065
PA14_58290	605.735	-0.057	0.900	-0.443	0.133	0.387	0.326	PA14_58290	1599.801	-0.031	0.906
PA14_58300	939.012	-0.551	0.081	-1.211	0.000	-0.816	0.023	PA14_58300	2890.775	0.922	0.000
PA14_58320	5193.796	0.361	0.411	-0.910	0.009	0.520	0.274	PA14_58320	12260.057	1.102	0.000
PA14_58330	3290.059	-1.037	0.000	-1.550	0.000	1.986	0.000	PA14_58330	5368.422	-1.534	0.000
PA14_58350	2592.567	-0.599	0.042	-3.607	0.000	-0.098	0.821	PA14_58350	5004.355	0.631	0.062
PA14_58360	993.313	-0.441	0.204	-2.551	0.000	1.188	0.001	PA14_58360	972.095	-0.423	0.080
PA14_58375	1716.360	1.300	0.000	0.225	0.451	-1.506	0.000	PA14_58375	2179.376	0.134	0.610
PA14_58380	690.619	0.564	0.208	-0.230	0.599	0.871	0.077	PA14_58380	2134.971	-0.627	0.005
PA14_58390	29332.395	1.341	0.000	0.499	0.144	-0.060	0.912	PA14_58390	17096.035	1.596	0.000
PA14_58410	4211.045	1.564	0.000	-1.753	0.000	-2.626	0.000	PA14_58410	2133.653	1.510	0.000
PA14_58420	4009.536	0.966	0.018	-2.656	0.000	-3.292	0.000	PA14_58420	3284.474	0.453	0.052
PA14_58440	1260.170	1.324	0.000	-1.893	0.000	-3.064	0.000	PA14_58440	847.405	1.130	0.000
PA14_58450	1930.594	1.481	0.000	-1.845	0.000	-2.880	0.000	PA14_58450	970.568	0.613	0.007
PA14_58470	1515.010	1.173	0.005	-2.441	0.000	-2.912	0.000	PA14_58470	1218.155	0.944	0.031
PA14_58490	1646.940	1.437	0.000	-2.244	0.000	-2.644	0.000	PA14_58490	1438.098	0.588	0.007
PA14_58500	309.574	-0.015	0.980	2.101	0.000	0.755	0.138	PA14_58500	425.511	1.086	0.000
PA14_58510	57.254	2.101	0.000	0.982	0.030	-1.001	0.176	PA14_58510	110.944	0.056	0.868
PA14_58515	74.335	0.572	0.214	1.273	0.000	0.594	0.274	PA14_58515	219.476	0.441	0.136
PA14_58530	126.562	0.440	0.297	0.755	0.027	-0.064	0.916	PA14_58530	557.341	-0.039	0.867
PA14_58540	173.281	-0.342	0.473	-0.327	0.413	-0.308	0.577	PA14_58540	855.127	-0.847	0.000
PA14_58550	273.062	0.604	0.057	0.811	0.004	0.135	0.770	PA14_58550	1073.251	1.549	0.000
PA14_58560	245.200	-1.150	0.000	0.505	0.084	-2.602	0.000	PA14_58560	326.201	-1.396	0.000
PA14_58570	870.928	-1.909	0.000	-0.625	0.013	-2.922	0.000	PA14_58570	371.198	-0.342	0.225
PA14_58580	285.038	-0.201	0.708	0.285	0.497	0.205	0.727	PA14_58580	306.147	-0.506	0.485
PA14_58600	80.100	-1.266	0.003	-0.788	0.044	-1.803	0.001	PA14_58600	168.227	-0.675	0.223
PA14_58610	681.460	-0.327	0.480	-0.331	0.391	4.439	0.000	PA14_58610	434.140	-0.772	0.002
PA14_58620	128.893	0.149	0.757	0.640	0.054	0.140	0.796	PA14_58620	282.726	-1.635	0.000
PA14_58630	1343.497	-0.706	0.014	-0.618	0.022	-0.377	0.313	PA14_58630	3088.481	-1.332	0.000
PA14_58650	772.864	0.216	0.490	-0.300	0.242	0.675	0.027	PA14_58650	1342.546	0.406	0.077
PA14_58660	313.247	0.577	0.066	1.008	0.000	1.646	0.000	PA14_58660	901.020	0.538	0.021
PA14_58670	201.353	0.043	0.940	0.205	0.601	0.717	0.114	PA14_58670	900.673	0.075	0.760
PA14_58690	2386.733	-0.425	0.127	-0.950	0.000	0.754	0.014	PA14_58690	15468.007	-1.538	0.000
PA14_58700	352.307	0.875	0.008	1.395	0.000	0.681	0.095	PA14_58700	916.761	-0.143	0.647
PA14_58710	240.697	-0.364	0.328	-0.399	0.207	-2.654	0.000	#N/A	#N/A	#N/A	#N/A
PA14_58720	1730.640	-0.813	0.012	-1.315	0.000	-2.062	0.000	PA14_58720	4088.754	1.178	0.000

PA14_58730	9204.981	-1.941	0.000	-3.129	0.000	-6.361	0.000	PA14_58730	14949.745	0.839	0.000
PA14_58740	177.162	-0.471	0.359	-0.638	0.136	-1.107	0.040	PA14_58740	940.191	-2.442	0.000
PA14_58750	4679.156	0.414	0.166	-0.615	0.018	-1.201	0.000	PA14_58750	12792.453	-0.941	0.000
PA14_58760	3558.694	0.107	0.752	-1.425	0.000	-1.476	0.000	PA14_58760	13940.472	-0.988	0.000
PA14_58770	502.168	0.572	0.054	-0.061	0.853	-1.425	0.000	PA14_58770	2253.292	0.417	0.108
PA14_58780	348.923	0.020	0.966	-0.108	0.741	-0.693	0.063	PA14_58780	1793.516	0.312	0.273
PA14_58790	87.513	-0.160	0.766	-0.323	0.421	-0.908	0.083	PA14_58790	494.001	1.226	0.000
PA14_58800	429.579	-0.844	0.016	-0.873	0.007	-0.987	0.018	PA14_58800	1113.211	-0.746	0.003
PA14_58810	1381.658	-0.020	0.974	-0.037	0.935	1.136	0.012	PA14_58810	2608.536	-1.131	0.003
PA14_58820	63.073	-1.410	0.002	-1.469	0.000	-0.488	0.411	PA14_58820	483.330	-2.245	0.000
PA14_58830	377.758	-1.022	0.007	-0.975	0.005	0.416	0.400	PA14_58830	1638.861	-2.301	0.000
PA14_58840	1204.467	0.263	0.609	0.189	0.667	0.594	0.248	PA14_58840	3859.029	-1.437	0.000
PA14_58850	302.059	-0.010	0.984	-0.451	0.151	0.179	0.701	PA14_58850	1664.435	-1.620	0.000
PA14_58860	176.314	0.217	0.622	0.957	0.002	0.848	0.037	PA14_58860	770.136	-1.021	0.000
PA14_58870	1053.450	0.333	0.230	-0.156	0.555	0.051	0.896	PA14_58870	2474.795	-0.283	0.234
PA14_58880	177.739	0.558	0.111	0.493	0.118	0.502	0.237	PA14_58880	543.041	0.458	0.061
PA14_58890	67.868	-0.090	0.892	-0.428	0.339	1.031	0.045	PA14_58890	104.050	0.453	0.217
PA14_58900	467.883	0.248	0.470	0.532	0.046	0.811	0.015	PA14_58900	1174.598	-0.195	0.429
PA14_58910	76.550	-0.073	0.908	0.449	0.268	0.468	0.394	PA14_58910	143.717	-0.413	0.221
PA14_58920	212.387	0.324	0.639	0.060	0.925	0.354	0.639	PA14_58920	488.514	-1.634	0.000
PA14_58930	13.567	-0.643	0.477	-0.258	0.744	-0.267	0.804	PA14_58930	42.081	-0.477	0.367
PA14_58940	2.977	0.778	0.581	-0.545	0.662	0.222	0.885	PA14_58940	13.254	0.334	0.762
PA14_58950	34.974	-0.682	0.263	-0.800	0.119	-0.029	0.972	PA14_58950	82.027	-0.181	0.708
PA14_58960	8.824	-0.386	0.746	-0.494	0.595	0.560	0.622	PA14_58960	28.273	-0.457	0.487
PA14_58970	103.999	-0.362	0.432	-1.458	0.000	0.255	0.637	PA14_58970	217.442	-0.672	0.024
PA14_58980	24.122	-0.001	0.999	-1.965	0.001	-0.933	0.238	PA14_58980	81.895	-1.194	0.001
PA14_58990	109.700	-0.054	0.921	-0.737	0.031	0.226	0.660	PA14_58990	284.765	-0.761	0.004
PA14_59000	7.992	-0.681	0.526	-1.405	0.103	-0.674	0.559	PA14_59000	21.291	-0.207	0.804
PA14_59010	9.057	-0.599	0.579	-0.137	0.887	-1.222	0.266	PA14_59010	34.487	-0.810	0.152
PA14_59020	24.848	-0.411	0.580	-0.181	0.777	0.000	1.000	PA14_59020	89.568	-1.022	0.005
PA14_59030	13.834	-0.974	0.249	-1.141	0.112	1.225	0.133	PA14_59030	42.667	-0.381	0.469
PA14_59050	18.095	-0.615	0.466	-0.977	0.149	-0.033	0.974	PA14_59050	1289.392	1.069	0.000
PA14_59060	38.130	-0.651	0.296	-0.851	0.103	-0.786	0.273	PA14_59060	124.346	-0.523	0.131
PA14_59070	672.911	-0.293	0.406	-0.458	0.112	-0.052	0.911	PA14_59070	2139.836	-0.825	0.000
PA14_59090	257.631	0.006	0.989	-0.065	0.852	0.425	0.283	PA14_59090	1005.684	-0.184	0.500
PA14_59100	274.706	0.220	0.556	-0.312	0.302	0.104	0.822	PA14_59100	914.720	-0.019	0.968
PA14_59110	33.785	0.411	0.610	0.207	0.767	1.192	0.109	PA14_59110	92.367	0.418	0.286
PA14_59120	85.814	-0.488	0.266	-1.592	0.000	-1.093	0.027	PA14_59120	407.887	-0.982	0.000
PA14_59130	144.867	-0.285	0.495	-0.897	0.005	-1.231	0.004	PA14_59130	445.368	-0.242	0.370
PA14_59140	140.313	-0.169	0.731	-1.076	0.002	-1.362	0.003	PA14_59140	772.991	-0.354	0.147
PA14_59150	294.392	-1.362	0.000	-2.562	0.000	-2.535	0.000	PA14_59150	1324.335	-0.626	0.005
PA14_59160	42.137	-0.029	0.970	-0.442	0.364	-0.591	0.385	PA14_59160	141.625	-0.756	0.021
PA14_59170	434.811	-0.912	0.027	-1.953	0.000	-1.821	0.000	PA14_59170	2912.830	-2.057	0.000
PA14_59180	282.392	-0.249	0.505	-0.834	0.003	0.272	0.514	PA14_59180	1503.593	0.336	0.143
PA14_59190	4649.939	-0.274	0.440	-0.862	0.002	-0.620	0.089	PA14_59190	20442.764	-1.264	0.010
PA14_59200	356.126	0.255	0.482	0.123	0.705	0.979	0.004	PA14_59200	1096.098	0.104	0.663
PA14_59210	100.023	0.008	0.989	-0.334	0.429	2.381	0.000	PA14_59210	234.229	0.245	0.407
PA14_59220	3405.063	-1.117	0.000	-2.196	0.000	-2.049	0.000	PA14_59220	116580.662	-6.035	0.000
PA14_59230	415.748	0.830	0.040	0.387	0.332	-0.607	0.237	PA14_59230	3102.642	-1.479	0.000
PA14_59240	130.983	0.369	0.635	1.847	0.001	2.292	0.000	PA14_59240	174.620	-0.260	0.434
PA14_59250	94.960	-0.334	0.486	-1.003	0.007	-0.130	0.829	PA14_59250	185.109	-0.007	1.000
PA14_59270	86.072	0.077	0.897	0.147	0.730	0.240	0.676	PA14_59270	203.358	-0.138	0.694
PA14_59280	13.984	-0.747	0.398	-1.132	0.118	0.638	0.491	PA14_59280	39.768	-0.467	0.390
PA14_59290	39.388	-0.854	0.116	-1.354	0.004	-0.142	0.852	PA14_59290	71.862	-0.031	0.980
PA14_59310	38.006	-0.351	0.612	-0.437	0.427	0.127	0.877	PA14_59310	90.945	-0.193	0.753
PA14_59320	287.262	0.254	0.609	-0.004	0.994	0.932	0.045	PA14_59320	400.488	0.193	0.459
PA14_59340	57.536	-0.600	0.219	-0.494	0.243	0.827	0.103	PA14_59340	220.967	-1.230	0.000
PA14_59350	239.627	0.021	0.969	-0.433	0.191	0.529	0.211	PA14_59350	673.154	-1.148	0.000
PA14_59360	16.878	-0.065	0.954	-0.710	0.342	0.079	0.944	PA14_59360	68.522	-1.208	0.002

PA14_59370	540.171	-0.318	0.320	-0.543	0.039	0.352	0.329	PA14_59370	1441.924	-0.865	0.000
PA14_59380	138.722	-0.683	0.097	-1.037	0.004	1.642	0.000	PA14_59380	777.254	-2.590	0.000
PA14_59390	846.385	-1.115	0.011	-2.097	0.000	-1.850	0.000	PA14_59390	5590.879	-2.693	0.000
PA14_59400	1533.890	-0.424	0.166	-0.383	0.166	1.555	0.000	PA14_59400	2476.618	-1.805	0.000
PA14_59410	23.490	-0.185	0.835	-0.298	0.651	0.312	0.723	PA14_59410	83.393	-0.099	0.812
PA14_59430	159.348	-0.365	0.473	-2.176	0.000	1.116	0.021	PA14_59430	278.360	-1.776	0.003
PA14_59440	97.807	-0.870	0.050	-1.741	0.000	-0.605	0.272	PA14_59440	318.049	-0.770	0.004
PA14_59470	83.496	-1.196	0.009	-2.275	0.000	0.198	0.758	PA14_59470	281.579	-1.245	0.001
PA14_59480	109.985	-0.726	0.110	-1.001	0.011	0.061	0.925	PA14_59480	416.275	-0.753	0.002
PA14_59490	87.589	-0.096	0.888	-0.105	0.838	2.096	0.000	PA14_59490	204.532	-0.635	0.030
PA14_59500	73.190	-0.544	0.275	-1.322	0.001	1.468	0.002	PA14_59500	206.478	-1.010	0.000
PA14_59510	44.807	-0.256	0.717	-0.970	0.060	1.866	0.001	PA14_59510	94.455	-0.732	0.053
PA14_59520	10.605	-1.883	0.066	-1.622	0.076	0.624	0.596	PA14_59520	29.920	-0.899	0.140
PA14_59530	49.313	-0.769	0.153	-1.102	0.017	1.649	0.001	PA14_59530	104.340	-0.526	0.245
PA14_59540	133.657	-0.390	0.380	-0.483	0.189	0.491	0.307	PA14_59540	325.189	-0.115	0.677
PA14_59550	4126.796	0.993	0.000	0.595	0.012	-0.055	0.889	PA14_59550	10030.828	0.947	0.018
PA14_59560	30.341	0.711	0.282	0.342	0.579	0.159	0.863	PA14_59560	65.259	2.614	0.000
PA14_59570	7.496	0.444	0.714	0.683	0.459	0.967	0.383	PA14_59570	16.096	2.098	0.105
PA14_59580	1026.723	0.264	0.397	0.066	0.825	0.281	0.428	PA14_59580	2718.361	-0.218	0.393
PA14_59590	1352.106	-0.070	0.887	-1.238	0.000	-0.665	0.109	PA14_59590	4496.848	-1.286	0.000
PA14_59600	49.977	0.240	0.705	0.467	0.318	1.839	0.000	PA14_59600	158.571	0.570	0.070
PA14_59610	1431.563	0.132	0.708	-0.182	0.520	0.494	0.139	PA14_59610	7125.830	-1.020	0.001
PA14_59620	2706.329	-0.176	0.633	-0.400	0.159	0.475	0.192	PA14_59620	19337.311	-1.762	0.000
PA14_59630	1771.480	-0.195	0.627	-0.929	0.001	-0.192	0.672	PA14_59630	11760.533	-1.861	0.000
PA14_59640	53.033	-0.621	0.258	-0.310	0.532	0.722	0.216	PA14_59640	110.179	-0.131	0.793
PA14_59650	30.005	-0.222	0.798	0.521	0.389	1.705	0.010	PA14_59650	38.486	0.531	0.528
PA14_59660	7.382	-0.714	0.524	-1.984	0.029	0.516	0.660	PA14_59660	19.059	0.106	0.945
PA14_59670	6.937	0.109	0.939	-0.586	0.561	0.726	0.536	PA14_59670	40.458	-0.523	0.426
PA14_59680	8.647	-0.999	0.347	-1.644	0.063	-1.773	0.104	PA14_59680	40.912	-0.478	0.487
PA14_59690	149.065	-0.144	0.749	-0.593	0.063	0.541	0.193	PA14_59690	316.113	-0.296	0.311
PA14_59700	14.112	-0.231	0.823	-0.837	0.254	0.540	0.570	PA14_59700	107.318	-0.357	0.346
PA14_59710	100.457	0.130	0.797	-0.232	0.544	-1.257	0.010	PA14_59710	281.391	-1.413	0.000
PA14_59720	18.319	-0.408	0.635	-0.165	0.824	0.422	0.641	PA14_59720	38.066	0.627	0.251
PA14_59735	101.122	0.224	0.650	0.011	0.980	1.062	0.016	#N/A	#N/A	#N/A	#N/A
PA14_59750	38.762	0.116	0.876	0.121	0.830	0.665	0.298	PA14_59750	128.649	-0.052	0.960
PA14_59760	62.282	1.153	0.015	1.334	0.002	1.698	0.001	PA14_59760	146.663	0.301	0.479
PA14_59770	251.910	-0.371	0.268	-1.251	0.000	-1.626	0.000	PA14_59770	4361.248	1.629	0.000
PA14_59780	3698.940	0.029	0.938	-0.164	0.524	-1.132	0.000	PA14_59780	24577.511	1.194	0.000
PA14_59790	4500.010	-0.966	0.002	-1.652	0.000	-2.697	0.000	PA14_59790	17234.157	-0.842	0.001
PA14_59800	1164.072	0.313	0.409	0.039	0.917	0.570	0.153	PA14_59800	5447.054	-0.205	0.366
PA14_59820	52.927	0.213	0.767	-0.296	0.601	1.346	0.024	PA14_59820	242.688	0.522	0.056
PA14_59830	777.659	0.266	0.477	-0.132	0.695	0.147	0.746	PA14_59830	2016.661	-0.080	0.779
PA14_59840	49766.790	-1.318	0.000	-3.793	0.000	-3.344	0.000	PA14_59840	216276.677	-0.726	0.001
PA14_59845	1480.733	0.482	0.710	-0.179	0.874	-1.453	0.197	PA14_59845	3813.221	-2.343	0.000
PA14_59850	108.199	-0.616	0.201	-1.416	0.000	-1.461	0.008	PA14_59850	601.835	0.908	0.000
PA14_59860	3.128	0.391	0.808	-1.197	0.319	0.547	0.702	PA14_59860	20.235	1.395	0.051
PA14_59870	14.272	-0.079	0.943	-1.288	0.075	0.491	0.611	PA14_59870	84.744	1.372	0.000
PA14_59880	33.663	-0.500	0.433	-1.357	0.007	-0.708	0.324	PA14_59880	173.298	1.657	0.000
PA14_59890	22.057	0.178	0.843	-1.578	0.014	1.421	0.044	PA14_59890	136.039	-1.045	0.001
PA14_59900	58.896	-0.399	0.500	-1.387	0.002	0.579	0.344	PA14_59900	304.995	-0.591	0.099
PA14_59910	92.043	0.155	0.782	-0.256	0.553	1.789	0.000	PA14_59910	269.873	0.254	0.369
PA14_59920	81.927	-0.135	0.807	-0.192	0.645	0.312	0.567	PA14_59920	305.210	0.114	0.689
PA14_59930	52.037	-0.028	0.967	-0.746	0.076	-1.075	0.069	PA14_59930	222.265	0.394	0.227
PA14_59940	322.084	-0.163	0.695	-0.823	0.005	0.232	0.599	PA14_59940	1012.395	-0.242	0.341
PA14_59950	22.962	-1.014	0.106	-1.529	0.006	-1.090	0.152	PA14_59950	165.728	-0.287	0.403
PA14_59960	98.134	-0.194	0.716	-0.191	0.653	-0.500	0.361	PA14_59960	376.933	-1.245	0.000
PA14_59970	3502.732	1.030	0.030	-0.047	0.932	-2.209	0.000	PA14_59970	13605.127	-1.619	0.000
PA14_59980	12.228	-0.108	0.927	0.129	0.881	0.653	0.507	PA14_59980	59.646	-0.783	0.066
PA14_59990	26.329	-0.509	0.486	-0.988	0.087	0.382	0.638	PA14_59990	67.279	-1.093	0.007

PA14_60000	217.894	0.355	0.349	0.249	0.459	0.533	0.197
PA14_60010	16.198	0.591	0.521	0.429	0.586	0.568	0.575
PA14_60020	389.224	-0.012	0.981	0.124	0.749	0.125	0.814
PA14_60030	3687.504	0.093	0.857	-0.514	0.144	0.909	0.033
PA14_60040	1296.964	0.126	0.792	0.848	0.008	0.807	0.052
PA14_60050	617.470	0.447	0.147	0.348	0.217	1.354	0.000
PA14_60060	391.736	1.144	0.001	1.468	0.000	2.308	0.000
PA14_60070	3954.338	0.229	0.433	-0.172	0.503	-0.337	0.290
PA14_60080	2213.376	-0.272	0.384	-0.801	0.001	-0.453	0.178
PA14_60090	965.263	0.398	0.201	-0.319	0.263	-0.380	0.309
PA14_60100	3885.319	0.467	0.073	-0.473	0.047	-0.315	0.335
PA14_60110	3075.524	0.203	0.577	-0.351	0.226	-0.568	0.115
PA14_60120	3652.958	0.806	0.005	0.463	0.099	1.234	0.000
PA14_60130	8565.327	0.223	0.693	-1.435	0.000	-0.607	0.266
PA14_60140	664.352	1.162	0.000	0.279	0.402	0.626	0.118
PA14_60150	89.495	0.347	0.518	2.059	0.000	1.796	0.000
PA14_60160	991.979	0.881	0.007	2.973	0.000	1.627	0.000
PA14_60180	571.825	0.371	0.484	3.261	0.000	1.837	0.000
PA14_60190	12952.828	-0.546	0.019	-0.255	0.273	-2.394	0.000
PA14_60200	90.150	0.366	0.452	1.605	0.000	0.990	0.037
PA14_60210	1166.808	0.899	0.001	1.513	0.000	1.362	0.000
PA14_60230	1539.924	0.620	0.036	0.589	0.030	0.428	0.246
PA14_60240	29.101	-0.098	0.908	0.181	0.763	0.540	0.464
PA14_60250	578.069	0.129	0.775	-0.058	0.879	-0.295	0.519
PA14_60260	268.137	-0.488	0.167	-0.982	0.001	-0.764	0.057
PA14_60270	1873.321	0.347	0.380	0.268	0.444	-0.575	0.177
PA14_60280	390.646	-1.111	0.001	-1.827	0.000	1.185	0.002
PA14_60290	571.101	-0.807	0.002	-1.559	0.000	1.119	0.000
PA14_60300	1133.206	-0.441	0.191	-0.715	0.013	1.059	0.003
PA14_60310	15407.369	-0.183	0.656	-0.573	0.062	0.638	0.103
PA14_60320	1134.254	-0.247	0.401	-1.193	0.000	-0.596	0.049
PA14_60330	2822.050	0.515	0.105	1.130	0.000	0.639	0.081
PA14_60350	443.979	0.489	0.092	0.585	0.023	0.458	0.190
PA14_60360	1741.855	0.221	0.490	0.283	0.285	0.226	0.534
PA14_60370	5394.085	0.346	0.235	0.368	0.151	0.487	0.134
PA14_60380	3584.768	1.331	0.000	1.755	0.000	1.689	0.000
PA14_60390	362.861	0.813	0.030	1.612	0.000	0.991	0.022
PA14_60400	1319.959	0.345	0.747	1.527	0.044	1.359	0.146
PA14_60410	978.438	0.290	0.505	0.269	0.468	0.778	0.072
PA14_60420	2034.827	-0.234	0.482	0.005	0.989	0.128	0.752
PA14_60445	3498.011	0.665	0.018	0.817	0.002	1.196	0.000
PA14_60450	579.446	0.459	0.444	0.589	0.239	0.003	0.998
PA14_60460	17843.879	1.564	0.000	2.252	0.000	1.839	0.000
PA14_60470	801.242	0.460	0.152	1.033	0.000	-0.063	0.892
PA14_60480	105.757	-0.037	0.962	0.777	0.067	3.747	0.000
PA14_60490	2927.395	0.081	0.833	-1.549	0.000	-2.457	0.000
PA14_60500	444.275	0.017	0.972	0.387	0.186	0.766	0.031
PA14_60520	295.778	-0.851	0.010	0.274	0.393	2.797	0.000
PA14_60530	143.496	0.580	0.159	1.616	0.000	1.907	0.000
PA14_60540	211.793	-0.617	0.071	-0.792	0.008	0.844	0.025
PA14_60550	5231.288	0.421	0.176	0.192	0.526	-0.744	0.030
PA14_60560	769.955	-1.152	0.000	-2.493	0.000	-3.431	0.000
PA14_60570	9835.912	0.057	0.896	-0.921	0.001	-3.069	0.000
PA14_60580	614.834	0.216	0.548	-0.421	0.137	-1.859	0.000
PA14_60590	229.435	0.838	0.011	0.914	0.002	0.331	0.471
PA14_60600	433.468	0.859	0.035	1.467	0.000	0.781	0.108
PA14_60620	29.019	1.224	0.036	0.908	0.094	0.881	0.225
PA14_60630	243.816	0.654	0.055	0.389	0.232	0.287	0.532
PA14_60650	543.964	0.682	0.040	0.921	0.002	0.497	0.228

PA14_60000	497.049	-0.678	0.005
PA14_60010	48.922	0.169	0.812
PA14_60020	988.656	-0.152	0.564
PA14_60030	14866.329	-1.501	0.000
PA14_60040	2970.366	-0.114	0.611
PA14_60050	1984.482	-0.245	0.303
PA14_60060	1037.418	0.942	0.002
PA14_60070	13630.231	-0.149	0.547
PA14_60080	10668.225	-0.472	0.028
PA14_60090	4294.197	-0.510	0.291
PA14_60100	15215.626	1.444	0.000
PA14_60110	20068.353	0.214	0.326
PA14_60120	16128.732	-0.369	0.109
PA14_60130	60251.465	-3.493	0.000
PA14_60140	2269.362	-0.931	0.000
#N/A	#N/A	#N/A	#N/A
#N/A	#N/A	#N/A	#N/A
#N/A	#N/A	#N/A	#N/A
PA14_60190	44828.417	1.540	0.000
PA14_60200	133.925	1.193	0.000
PA14_60210	1434.597	1.393	0.000
PA14_60230	3036.946	1.049	0.000
PA14_60240	102.283	-0.326	0.406
PA14_60250	985.802	-0.283	0.258
PA14_60260	494.472	-0.859	0.066
PA14_60270	6746.247	0.129	0.608
PA14_60280	571.539	0.429	0.347
PA14_60290	1415.690	-0.440	0.061
PA14_60300	1626.863	0.128	0.570
PA14_60310	36368.256	-0.976	0.000
PA14_60320	4813.340	-0.450	0.039
PA14_60330	2862.760	0.765	0.000
PA14_60350	557.868	0.687	0.004
PA14_60360	2009.763	0.728	0.001
PA14_60370	5707.266	0.822	0.000
PA14_60380	3213.029	1.327	0.000
PA14_60390	658.913	0.170	0.556
PA14_60400	2227.443	2.537	0.000
PA14_60410	1658.718	-0.780	0.000
PA14_60420	3189.688	-0.210	0.359
PA14_60445	3803.177	0.121	0.577
PA14_60450	867.246	1.914	0.000
PA14_60460	12326.714	2.602	0.000
PA14_60470	2062.290	0.828	0.000
PA14_60480	82.341	-0.171	0.695
PA14_60490	12595.616	2.480	0.000
PA14_60500	428.072	-0.353	0.162
PA14_60520	920.289	-3.653	0.000
PA14_60530	481.472	-0.905	0.000
PA14_60540	1638.664	-3.754	0.000
PA14_60550	11160.288	-0.128	0.583
PA14_60560	5263.414	-1.431	0.000
PA14_60570	16819.457	0.651	0.073
PA14_60580	1546.188	0.900	0.000
PA14_60590	509.004	1.141	0.000
PA14_60600	737.704	0.515	0.030
#N/A	#N/A	#N/A	#N/A
PA14_60630	702.216	0.431	0.065
PA14_60650	1221.239	1.239	0.000

PA14_60660	206.987	0.610	0.085	0.731	0.019	0.794	0.051	PA14_60660	598.363	0.198	0.452
PA14_60670	61.328	0.138	0.820	-0.019	0.970	-1.107	0.063	PA14_60670	209.866	0.284	0.346
PA14_60690	4.637	-0.873	0.512	0.486	0.661	0.669	0.609	PA14_60690	12.005	1.221	0.186
PA14_60700	4371.455	-2.944	0.000	-5.811	0.000	-5.962	0.000	PA14_60700	13170.242	0.167	0.465
PA14_60710	890.181	1.570	0.000	1.992	0.000	0.910	0.006	PA14_60710	690.864	0.098	0.726
PA14_60730	226.659	-0.967	0.001	-2.953	0.000	-2.697	0.000	PA14_60730	517.616	-1.414	0.000
PA14_60750	1345.954	-2.445	0.000	-5.165	0.000	-1.300	0.000	PA14_60750	5275.934	-3.458	0.000
PA14_60760	146.044	-0.962	0.002	-1.947	0.000	-2.759	0.000	PA14_60760	388.854	-0.653	0.008
PA14_60770	221.705	-0.398	0.265	-1.033	0.000	-2.521	0.000	PA14_60770	398.528	0.336	0.205
PA14_60780	68.902	-0.396	0.429	-1.144	0.004	-1.957	0.001	PA14_60780	151.999	0.433	0.260
PA14_60790	107.137	-0.805	0.037	-1.136	0.001	-2.343	0.000	PA14_60790	545.762	-1.713	0.000
PA14_60800	3668.272	-0.014	0.974	-0.397	0.136	-0.789	0.014	PA14_60800	6432.347	-0.115	0.648
PA14_60810	351.667	-1.130	0.001	-0.383	0.263	-0.488	0.272	PA14_60810	578.500	-0.412	0.262
PA14_60820	263.454	0.722	0.033	2.129	0.000	1.574	0.000	PA14_60820	1231.729	-0.044	0.912
PA14_60830	563.711	-0.650	0.090	2.353	0.000	1.520	0.000	PA14_60830	2389.562	0.025	0.906
PA14_60850	486.634	-1.209	0.006	2.443	0.000	0.584	0.291	PA14_60850	2281.662	-1.609	0.000
PA14_60860	481.422	-0.569	0.057	0.660	0.011	-0.528	0.145	PA14_60860	2853.771	-0.706	0.001
PA14_60870	1354.579	-0.441	0.143	-0.693	0.008	1.502	0.000	PA14_60870	2575.962	0.284	0.216
PA14_60890	2190.676	0.053	0.889	0.808	0.001	0.688	0.026	PA14_60890	2704.269	0.844	0.000
PA14_60900	58.096	-0.450	0.484	-0.038	0.951	-0.152	0.850	PA14_60900	247.210	-0.722	0.007
PA14_60920	1274.118	-0.063	0.920	0.145	0.752	0.878	0.079	PA14_60920	1903.473	-0.568	0.010
PA14_60930	202.841	-0.094	0.857	-0.712	0.040	0.202	0.700	PA14_60930	426.891	-1.087	0.000
PA14_60950	1680.493	-0.673	0.025	-1.721	0.000	-0.620	0.087	PA14_60950	3058.320	-0.493	0.023
PA14_60960	2579.175	-3.520	0.000	-5.596	0.000	-3.586	0.000	PA14_60960	19266.051	-5.148	0.000
PA14_60970	89.912	-0.823	0.097	-1.946	0.000	0.096	0.891	PA14_60970	920.871	-2.148	0.000
PA14_60990	393.199	0.448	0.162	0.993	0.000	-0.146	0.744	PA14_60990	695.488	0.546	0.023
PA14_61000	251.720	-1.636	0.000	-3.063	0.000	-0.619	0.091	PA14_61000	2009.562	-2.146	0.000
PA14_61010	3263.497	-0.960	0.000	-2.123	0.000	-2.265	0.000	PA14_61010	20052.010	-1.316	0.000
PA14_61020	57.542	-0.365	0.506	-0.277	0.550	-0.283	0.667	PA14_61020	381.210	-2.448	0.000
PA14_61040	404.107	0.276	0.483	0.402	0.207	0.113	0.820	PA14_61040	3818.811	-3.289	0.000
PA14_61050	683.751	-1.642	0.000	-1.735	0.000	-2.386	0.000	PA14_61050	3412.279	-1.693	0.000
PA14_61060	223.449	0.112	0.807	-0.164	0.640	1.051	0.005	PA14_61060	1035.767	-2.249	0.000
PA14_61080	331.556	-0.057	0.891	-0.683	0.009	-0.653	0.064	PA14_61080	694.595	0.919	0.000
PA14_61090	204.839	0.338	0.357	0.064	0.859	-0.041	0.936	PA14_61090	531.205	-0.027	0.930
PA14_61110	14.958	1.230	0.135	1.632	0.019	1.652	0.058	PA14_61110	87.232	0.539	0.159
PA14_61120	364.471	1.031	0.001	1.408	0.000	0.496	0.233	PA14_61120	1410.239	0.278	0.250
PA14_61130	397.165	1.752	0.000	1.652	0.000	0.910	0.050	PA14_61130	2081.056	0.912	0.000
PA14_61140	146.121	2.007	0.000	1.689	0.000	1.409	0.001	PA14_61140	980.038	1.384	0.000
PA14_61150	904.479	1.950	0.000	1.558	0.000	0.987	0.002	PA14_61150	2537.973	1.004	0.000
PA14_61170	69.233	1.050	0.046	1.307	0.005	0.963	0.123	PA14_61170	74.711	2.518	0.000
PA14_61180	23.224	0.245	0.789	0.355	0.606	2.380	0.000	PA14_61180	61.027	-0.757	0.083
PA14_61190	907.481	-2.257	0.000	-2.336	0.000	1.102	0.000	PA14_61190	2515.595	-3.269	0.000
PA14_61200	2369.670	-2.532	0.000	-2.444	0.000	1.823	0.000	PA14_61200	2871.130	-2.402	0.000
PA14_61210	153.779	0.953	0.005	1.096	0.000	0.430	0.357	PA14_61210	222.770	1.807	0.000
PA14_61220	415.978	0.994	0.001	1.448	0.000	1.643	0.000	PA14_61220	371.085	2.278	0.000
PA14_61250	358.059	0.581	0.067	1.119	0.000	0.596	0.113	PA14_61250	311.171	1.750	0.000
PA14_61260	167.423	0.377	0.352	1.903	0.000	2.124	0.000	PA14_61260	142.393	0.529	0.165
PA14_61270	37.746	0.588	0.330	0.243	0.666	1.486	0.011	PA14_61270	259.771	-2.923	0.000
PA14_61280	273.966	-0.142	0.754	0.098	0.794	-0.922	0.028	PA14_61280	920.179	0.024	0.940
PA14_61290	856.375	-0.145	0.660	-0.138	0.612	-1.307	0.000	PA14_61290	2547.918	0.098	0.675
PA14_61300	804.763	-0.590	0.045	-2.070	0.000	-0.522	0.143	PA14_61300	3403.694	-0.897	0.000
PA14_61320	54.850	0.750	0.155	1.338	0.002	1.376	0.013	PA14_61320	140.099	1.017	0.022
PA14_61330	5.342	-0.908	0.459	-0.278	0.800	-0.204	0.884	PA14_61330	84.969	-2.996	0.015
PA14_61340	26.534	-0.190	0.822	0.129	0.843	-0.253	0.777	PA14_61340	209.399	-0.962	0.001
PA14_61350	136.652	0.350	0.428	0.207	0.597	0.318	0.537	PA14_61350	697.323	-1.021	0.000
PA14_61360	822.146	0.850	0.001	0.810	0.001	0.565	0.076	PA14_61360	1535.981	1.272	0.000
PA14_61370	65.248	1.038	0.058	1.998	0.000	1.168	0.063	PA14_61370	82.242	0.924	0.015
PA14_61380	3938.474	-3.423	0.000	-4.730	0.000	0.243	0.547	PA14_61380	6583.596	-3.676	0.000
PA14_61390	328.015	-0.717	0.037	-0.966	0.002	-1.393	0.000	PA14_61390	1597.150	-0.616	0.004

PA14_61400	1277.486	-0.067	0.874	-0.354	0.218	-0.065	0.885	PA14_61400	2709.029	-0.301	0.197
PA14_61410	120.514	-2.279	0.000	-1.575	0.000	-0.706	0.135	PA14_61410	975.313	-3.562	0.000
PA14_61430	63.173	-0.598	0.225	0.651	0.103	-1.257	0.033	PA14_61430	175.509	0.690	0.041
PA14_61440	1090.248	0.432	0.230	0.971	0.001	0.281	0.528	PA14_61440	1628.491	0.923	0.000
PA14_61450	48.637	0.612	0.296	1.314	0.004	-0.272	0.734	PA14_61450	79.260	0.626	0.120
PA14_61460	546.895	0.160	0.708	0.909	0.002	-0.222	0.632	PA14_61460	813.999	-0.112	0.647
PA14_61470	746.222	-0.186	0.618	-0.498	0.078	-0.707	0.049	PA14_61470	2174.940	-1.266	0.000
PA14_61480	405.233	-0.236	0.481	-0.670	0.009	-0.586	0.089	PA14_61480	634.451	-0.477	0.048
PA14_61500	156.999	-1.560	0.000	-2.520	0.000	0.801	0.038	PA14_61500	1626.428	-4.353	0.000
PA14_61510	3.812	-1.412	0.243	-1.534	0.143	-0.584	0.665	PA14_61510	92.189	-4.360	0.000
PA14_61520	18.867	-1.298	0.069	-2.004	0.002	-0.043	0.966	PA14_61520	157.993	-3.304	0.000
PA14_61530	8.854	-1.287	0.165	-2.079	0.011	-1.185	0.259	PA14_61530	59.319	-3.005	0.000
PA14_61540	34.813	-0.689	0.228	-1.900	0.000	0.166	0.825	PA14_61540	129.806	-1.597	0.000
PA14_61550	69.104	0.069	0.919	0.737	0.070	2.189	0.000	PA14_61550	132.286	-0.230	0.543
PA14_61560	33.867	-0.100	0.902	0.502	0.333	1.291	0.031	PA14_61560	56.597	1.068	0.013
PA14_61580	392.105	-0.330	0.401	-0.074	0.844	-0.355	0.428	PA14_61580	1883.214	-0.006	0.993
PA14_61590	302.236	-0.114	0.818	0.192	0.604	0.522	0.238	PA14_61590	1857.621	0.091	0.678
PA14_61600	486.392	0.098	0.827	-0.459	0.137	1.547	0.000	PA14_61600	3105.854	-1.152	0.000
PA14_61610	230.580	-0.287	0.440	-0.106	0.752	0.943	0.007	PA14_61610	1053.410	0.428	0.195
PA14_61620	54.309	-0.450	0.436	-0.194	0.705	0.427	0.503	PA14_61620	255.847	1.786	0.000
PA14_61640	73.848	-0.284	0.591	-0.562	0.168	0.620	0.231	PA14_61640	245.588	1.434	0.000
PA14_61650	1261.246	-0.540	0.092	-0.599	0.038	-2.570	0.000	PA14_61650	4001.047	-1.109	0.000
PA14_61660	1013.180	0.807	0.015	1.796	0.000	0.288	0.527	PA14_61660	1065.858	0.030	0.928
PA14_61670	365.214	0.612	0.065	1.318	0.000	0.751	0.050	PA14_61670	560.005	-0.144	0.553
PA14_61680	530.865	0.626	0.022	0.779	0.002	-0.471	0.181	PA14_61680	781.550	0.608	0.009
PA14_61700	1092.558	0.818	0.001	1.324	0.000	0.861	0.003	PA14_61700	1275.339	1.632	0.000
PA14_61710	1225.519	1.227	0.000	1.810	0.000	0.989	0.004	PA14_61710	3385.042	1.126	0.000
PA14_61720	1549.021	0.139	0.728	-0.256	0.407	0.013	0.977	PA14_61720	3985.003	0.470	0.030
PA14_61740	411.864	0.269	0.447	-0.115	0.723	-0.508	0.185	PA14_61740	809.354	1.099	0.000
PA14_61750	3781.162	0.595	0.061	1.963	0.000	0.472	0.224	PA14_61750	10477.459	0.941	0.000
PA14_61760	1115.068	1.857	0.000	3.817	0.000	3.066	0.000	#N/A	#N/A	#N/A	#N/A
PA14_61770	6939.380	0.957	0.000	2.580	0.000	2.074	0.000	PA14_61770	5691.395	2.497	0.000
PA14_61780	19850.421	1.010	0.002	2.040	0.000	0.906	0.023	PA14_61780	8454.850	1.960	0.000
PA14_61790	802.862	0.821	0.003	2.078	0.000	1.922	0.000	PA14_61790	715.446	2.420	0.000
PA14_61820	1414.424	0.749	0.090	1.132	0.004	0.233	0.699	PA14_61820	1194.904	2.026	0.000
PA14_61830	67.118	0.378	0.681	2.686	0.000	2.512	0.001	#N/A	#N/A	#N/A	#N/A
PA14_61840	259.756	-0.288	0.473	-0.535	0.091	-1.208	0.003	PA14_61840	2108.041	-2.177	0.000
PA14_61850	1904.877	-0.248	0.407	0.586	0.013	0.641	0.033	PA14_61850	1030.465	0.792	0.010
PA14_61860	441.682	0.278	0.394	0.354	0.190	-1.380	0.000	PA14_61860	1251.301	0.835	0.000
PA14_61870	210.369	-0.060	0.910	-0.382	0.275	-0.862	0.053	PA14_61870	818.435	0.098	0.699
PA14_61880	41.157	-0.125	0.872	0.601	0.227	0.394	0.584	PA14_61880	102.366	0.888	0.011
PA14_61890	327.101	0.602	0.073	1.139	0.000	0.940	0.012	PA14_61890	467.122	0.881	0.000
PA14_61910	26.309	-0.198	0.818	-1.127	0.059	0.220	0.805	PA14_61910	81.995	-0.564	0.125
PA14_61920	38.485	-1.150	0.025	-2.310	0.000	-1.349	0.032	PA14_61920	160.627	-1.226	0.000
PA14_61940	118.601	-1.515	0.000	-2.126	0.000	-0.324	0.508	PA14_61940	367.095	-1.844	0.000
PA14_61950	180.998	-0.471	0.185	-1.589	0.000	-3.177	0.000	PA14_61950	873.547	-0.321	0.279
PA14_61960	841.745	1.225	0.000	2.184	0.000	1.492	0.000	PA14_61960	818.767	2.627	0.000
PA14_61980	549.342	1.178	0.000	1.401	0.000	1.234	0.000	PA14_61980	290.606	1.983	0.000
PA14_61990	1389.091	0.563	0.025	0.188	0.466	-0.420	0.183	PA14_61990	1027.952	0.835	0.000
PA14_62000	1880.891	0.021	0.962	0.034	0.915	-1.069	0.001	PA14_62000	2996.586	-1.947	0.000
PA14_62010	421.293	0.076	0.848	0.725	0.004	-1.422	0.000	PA14_62010	288.104	0.255	0.345
PA14_62020	1381.143	0.190	0.645	0.630	0.039	0.642	0.104	PA14_62020	2718.402	0.052	0.844
PA14_62030	249.377	-0.045	0.927	0.285	0.383	0.525	0.196	PA14_62030	1387.244	-0.457	0.041
PA14_62040	559.294	-0.201	0.572	0.448	0.099	0.239	0.542	PA14_62040	2714.132	-0.986	0.000
PA14_62050	0.000	NA	NA	NA	NA	NA	NA	#N/A	#N/A	#N/A	#N/A
PA14_62060	913.557	4.303	0.000	-0.532	0.685	-0.208	0.893	#N/A	#N/A	#N/A	#N/A
PA14_62070	0.000	NA	NA	NA	NA	NA	NA	#N/A	#N/A	#N/A	#N/A
PA14_62080	0.000	NA	NA	NA	NA	NA	NA	#N/A	#N/A	#N/A	#N/A
PA14_62090	381.813	5.969	0.000	0.216	0.868	0.346	0.815	#N/A	#N/A	#N/A	#N/A

PA14_62100	180.726	-0.172	0.718	-0.110	0.782	-0.440	0.363	PA14_62100	673.547	-1.024	0.000
PA14_62110	350.924	-0.277	0.483	0.219	0.516	-0.158	0.741	PA14_62110	1641.031	-1.410	0.000
PA14_62120	850.155	0.180	0.581	0.648	0.007	-0.048	0.908	PA14_62120	1625.331	0.485	0.030
PA14_62130	6815.797	-0.019	0.960	-0.365	0.113	-1.897	0.000	PA14_62130	9306.847	0.233	0.298
PA14_62150	4972.067	0.474	0.136	0.412	0.155	-0.785	0.026	PA14_62150	6071.212	0.274	0.366
PA14_62160	4710.015	0.404	0.131	0.469	0.050	-0.399	0.211	PA14_62160	4843.886	0.741	0.001
PA14_62170	511.611	-0.119	0.843	-0.250	0.576	-1.594	0.001	PA14_62170	1696.260	-0.769	0.000
PA14_62180	61.666	0.165	0.833	-0.301	0.605	-1.237	0.087	PA14_62180	203.472	0.168	0.740
PA14_62190	353.452	-0.074	0.878	-0.204	0.553	-0.767	0.060	PA14_62190	763.323	0.369	0.137
PA14_62200	1523.988	0.549	0.027	1.076	0.000	0.760	0.008	PA14_62200	2322.030	1.235	0.000
PA14_62230	2108.465	-0.094	0.822	-0.157	0.619	-0.766	0.030	PA14_62230	2889.844	0.235	0.302
PA14_62240	105.673	-1.448	0.001	-2.163	0.000	1.421	0.003	PA14_62240	721.390	-4.092	0.000
PA14_62250	206.696	-0.766	0.023	-0.876	0.004	2.398	0.000	PA14_62250	1555.562	-4.557	0.000
PA14_62260	192.213	-1.377	0.000	-1.482	0.000	1.159	0.002	PA14_62260	919.964	-1.831	0.000
PA14_62270	732.013	0.315	0.376	0.842	0.003	-0.370	0.368	PA14_62270	624.420	-0.246	0.346
PA14_62280	123.408	0.134	0.810	0.755	0.038	-0.648	0.242	PA14_62280	198.557	-0.540	0.065
PA14_62290	562.139	0.157	0.697	0.810	0.003	-1.369	0.000	PA14_62290	503.822	-0.127	0.721
PA14_62300	483.990	-0.367	0.410	0.334	0.375	-0.360	0.484	PA14_62300	345.513	-0.715	0.005
PA14_62330	440.999	-1.219	0.000	-0.308	0.326	-0.174	0.704	PA14_62330	270.677	-0.142	0.656
PA14_62350	1078.513	-1.216	0.000	-0.300	0.409	0.563	0.206	PA14_62350	1243.655	-1.480	0.000
PA14_62360	234.941	1.455	0.000	1.629	0.000	1.734	0.000	PA14_62360	702.139	0.757	0.009
PA14_62370	141.048	0.562	0.213	0.821	0.029	2.143	0.000	PA14_62370	339.771	-0.683	0.008
PA14_62380	188.884	-0.450	0.340	0.829	0.025	0.866	0.070	PA14_62380	765.371	-0.236	0.331
PA14_62390	2555.717	-2.342	0.000	-2.062	0.000	-0.347	0.303	PA14_62390	9145.622	-1.209	0.000
PA14_62400	1180.779	-0.404	0.177	-0.608	0.019	-1.665	0.000	PA14_62400	4546.329	1.710	0.000
PA14_62410	761.587	0.289	0.336	0.646	0.007	1.353	0.000	PA14_62410	2274.203	0.167	0.474
PA14_62420	955.902	-0.661	0.010	-0.869	0.000	0.507	0.106	PA14_62420	3403.936	-1.023	0.000
PA14_62430	166.579	1.253	0.000	1.698	0.000	1.755	0.000	PA14_62430	350.987	0.904	0.000
PA14_62440	478.097	0.512	0.070	1.224	0.000	0.816	0.010	PA14_62440	610.263	0.965	0.000
PA14_62450	895.135	0.756	0.008	1.090	0.000	0.398	0.282	PA14_62450	839.392	0.849	0.000
PA14_62470	286.185	0.816	0.011	1.235	0.000	-0.511	0.242	PA14_62470	478.514	0.974	0.000
PA14_62480	339.522	0.533	0.081	0.504	0.068	-0.749	0.044	PA14_62480	562.006	0.743	0.002
PA14_62490	13159.977	0.080	0.855	0.345	0.256	-0.561	0.136	PA14_62490	18994.347	0.700	0.015
PA14_62510	64.849	0.186	0.809	0.145	0.814	-0.566	0.455	PA14_62510	165.661	1.375	0.000
PA14_62520	79.286	0.764	0.141	1.536	0.000	1.035	0.073	PA14_62520	381.764	-0.473	0.129
PA14_62530	2113.787	0.569	0.065	0.259	0.394	0.075	0.871	PA14_62530	5080.956	-1.266	0.000
PA14_62540	4171.134	0.753	0.010	0.412	0.155	0.361	0.351	PA14_62540	7939.766	-0.344	0.123
PA14_62560	3385.562	0.455	0.075	1.081	0.000	1.390	0.000	PA14_62560	6244.835	1.134	0.000
PA14_62570	175.365	0.339	0.509	1.872	0.000	1.349	0.004	PA14_62570	307.895	1.953	0.000
PA14_62580	595.469	-0.060	0.899	0.510	0.089	-0.238	0.591	PA14_62580	1346.251	0.635	0.030
PA14_62590	345.167	-0.334	0.288	0.286	0.294	-0.855	0.012	PA14_62590	534.004	0.529	0.027
PA14_62600	203.853	-0.468	0.335	-0.005	0.993	-0.859	0.101	PA14_62600	391.323	0.696	0.117
PA14_62620	1236.791	-0.299	0.298	-0.891	0.000	-0.902	0.002	PA14_62620	2944.223	-0.342	0.137
PA14_62630	786.209	-0.052	0.889	-0.974	0.000	0.161	0.662	PA14_62630	1908.364	-0.784	0.000
PA14_62640	138.939	0.439	0.265	-0.147	0.700	-0.063	0.911	PA14_62640	487.564	-0.250	0.328
PA14_62650	2618.223	-0.967	0.000	-1.293	0.000	-0.153	0.682	PA14_62650	8735.604	-0.792	0.000
PA14_62660	419.576	-1.429	0.000	-1.761	0.000	-0.674	0.073	PA14_62660	3584.301	-0.900	0.000
PA14_62670	45.945	0.039	0.965	-0.088	0.887	-0.367	0.643	PA14_62670	272.155	-1.155	0.001
PA14_62680	4639.268	-1.690	0.000	-2.899	0.000	-0.146	0.758	PA14_62680	4044.559	-3.716	0.000
PA14_62690	29362.304	-1.739	0.000	-3.200	0.000	1.022	0.000	PA14_62690	12504.450	-3.191	0.000
PA14_62710	19745.555	0.250	0.369	0.362	0.119	1.608	0.000	PA14_62710	9267.662	1.805	0.000
PA14_62720	13294.473	1.478	0.000	2.006	0.000	1.663	0.000	PA14_62720	5887.593	1.797	0.000
PA14_62730	16355.224	0.930	0.000	1.556	0.000	2.176	0.000	PA14_62730	12153.347	1.317	0.000
PA14_62740	1486.998	0.710	0.028	1.052	0.000	2.102	0.000	PA14_62740	1239.159	1.736	0.000
PA14_62760	58340.137	0.675	0.047	1.300	0.000	2.273	0.000	PA14_62760	23271.072	1.206	0.000
PA14_62770	27887.154	0.681	0.018	1.168	0.000	2.462	0.000	PA14_62770	14452.920	1.239	0.000
PA14_62780	18267.781	1.718	0.000	3.212	0.000	4.033	0.000	PA14_62780	9088.804	1.830	0.000
PA14_62790	1796.119	0.956	0.050	3.280	0.000	3.556	0.000	#N/A	#N/A	#N/A	#N/A
PA14_62800	2398.557	0.297	0.483	0.865	0.008	0.799	0.057	#N/A	#N/A	#N/A	#N/A

PA14_62810	8577.590	0.170	0.706	0.806	0.012	0.484	0.272	PA14_62810	16437.293	0.312	0.184
PA14_62830	9239.655	0.637	0.090	2.114	0.000	1.530	0.000	PA14_62830	12163.956	2.236	0.000
PA14_62840	4356.505	-0.072	0.851	0.566	0.025	-0.309	0.383	PA14_62840	7765.114	1.786	0.000
PA14_62850	7016.777	0.454	0.314	1.614	0.000	1.428	0.002	PA14_62850	11080.647	1.752	0.000
PA14_62860	11483.589	-0.149	0.634	0.142	0.587	0.415	0.178	PA14_62860	35466.465	0.275	0.214
PA14_62870	2847.559	0.711	0.033	1.551	0.000	0.917	0.017	PA14_62870	5653.093	1.102	0.000
PA14_62880	638.208	0.954	0.006	1.458	0.000	2.119	0.000	PA14_62880	1495.321	1.108	0.000
PA14_62890	878.259	-0.129	0.737	-0.462	0.096	0.155	0.709	PA14_62890	1183.306	0.201	0.418
PA14_62900	830.736	-0.018	0.969	-0.346	0.236	0.288	0.469	PA14_62900	1768.127	0.229	0.341
PA14_62910	10579.250	0.129	0.709	-0.347	0.183	0.715	0.022	PA14_62910	8633.606	0.091	0.694
PA14_62920	5083.367	-0.166	0.749	-0.172	0.684	1.765	0.000	PA14_62920	3290.974	1.236	0.000
PA14_62930	3629.502	-0.119	0.729	0.003	0.993	1.212	0.000	PA14_62930	5828.496	0.499	0.019
PA14_62940	1327.164	-0.455	0.151	0.719	0.008	-1.129	0.001	PA14_62940	2651.998	1.730	0.000
PA14_62960	5435.956	0.386	0.335	1.923	0.000	-0.514	0.248	PA14_62960	5148.695	1.713	0.000
PA14_62970	21451.399	0.025	0.948	0.938	0.000	-2.115	0.000	PA14_62970	42045.170	1.945	0.000
PA14_62990	9118.188	0.261	0.461	1.621	0.000	-2.043	0.000	PA14_62990	15672.014	1.527	0.000
PA14_63010	484.577	0.139	0.705	0.089	0.773	-0.425	0.246	PA14_63010	1799.891	-1.150	0.000
PA14_63020	3448.914	0.556	0.246	1.420	0.000	0.476	0.398	PA14_63020	5969.724	-0.294	0.428
PA14_63030	4262.668	0.451	0.188	0.676	0.023	0.988	0.007	PA14_63030	7908.512	0.818	0.048
PA14_63040	14.938	0.219	0.835	-0.145	0.865	-1.289	0.206	PA14_63040	72.571	-1.465	0.000
PA14_63050	1042.123	-0.374	0.220	0.024	0.941	-0.006	0.989	PA14_63050	3641.611	-1.467	0.001
PA14_63060	1218.544	0.772	0.044	1.838	0.000	0.507	0.291	PA14_63060	1785.579	1.223	0.003
PA14_63070	396.875	0.084	0.866	0.215	0.550	0.040	0.941	PA14_63070	1049.681	0.148	0.553
PA14_63080	2697.041	1.705	0.000	4.001	0.000	4.613	0.000	PA14_63080	1512.192	3.620	0.000
PA14_63090	162.727	0.824	0.130	1.364	0.003	2.404	0.000	PA14_63090	260.200	2.334	0.000
PA14_63100	478.160	0.931	0.024	1.950	0.000	2.396	0.000	PA14_63100	692.600	2.156	0.000
PA14_63110	590.480	-2.140	0.000	-3.160	0.000	-0.784	0.027	PA14_63110	1024.359	1.651	0.081
PA14_63120	949.514	-1.893	0.000	-2.846	0.000	-0.299	0.514	PA14_63120	1132.626	1.218	0.091
PA14_63130	472.883	-1.924	0.000	-2.800	0.000	-0.674	0.104	PA14_63130	1181.696	-0.429	0.250
PA14_63150	219.765	-2.034	0.000	-2.401	0.000	-0.932	0.051	PA14_63150	616.259	-0.214	0.575
PA14_63160	485.522	-1.983	0.000	-2.835	0.000	-0.761	0.020	PA14_63160	1052.802	0.617	0.412
PA14_63170	1887.540	-1.426	0.000	-1.785	0.000	-1.758	0.000	PA14_63170	4532.959	-0.708	0.001
PA14_63190	55.167	0.394	0.479	0.952	0.022	-0.106	0.888	PA14_63190	257.961	-0.209	0.470
PA14_63200	231.621	0.119	0.800	0.875	0.004	1.071	0.005	PA14_63200	728.903	-0.886	0.000
PA14_63210	354.605	-1.356	0.000	-1.067	0.000	1.634	0.000	PA14_63210	2143.193	-1.587	0.000
PA14_63220	117.312	-1.569	0.000	-1.267	0.000	-1.096	0.023	PA14_63220	687.557	0.124	0.856
PA14_63230	139.892	0.983	0.014	1.283	0.000	-0.122	0.849	PA14_63230	743.314	-0.319	0.197
PA14_63240	91.699	-0.005	0.993	-0.680	0.067	-0.200	0.729	PA14_63240	337.848	-0.583	0.028
PA14_63250	268.850	-0.928	0.017	-0.912	0.011	-0.553	0.261	PA14_63250	983.281	-0.806	0.000
PA14_63270	157.491	-1.744	0.000	-2.392	0.000	-0.541	0.204	PA14_63270	558.563	-1.319	0.000
PA14_63280	1827.610	1.008	0.000	0.643	0.019	-1.070	0.002	PA14_63280	3824.624	0.126	0.620
PA14_63290	42.005	-1.738	0.000	-4.277	0.000	0.009	0.990	PA14_63290	164.038	-2.186	0.000
PA14_63300	122.388	0.867	0.044	1.730	0.000	0.348	0.559	PA14_63300	269.950	1.234	0.000
PA14_63310	60.601	0.646	0.171	1.121	0.004	-0.204	0.771	PA14_63310	108.979	1.582	0.000
PA14_63320	57.315	0.090	0.899	0.306	0.533	-0.085	0.911	PA14_63320	223.073	-0.744	0.006
PA14_63330	81.419	0.105	0.881	0.785	0.074	0.802	0.165	PA14_63330	182.590	-0.091	0.787
PA14_63340	1841.306	0.163	0.672	-0.457	0.112	-0.906	0.010	PA14_63340	8679.309	-0.718	0.001
PA14_63350	309.165	0.325	0.394	0.090	0.804	-0.160	0.743	PA14_63350	1362.802	0.457	0.038
PA14_63360	85.827	0.100	0.859	-0.154	0.716	-1.702	0.002	PA14_63360	424.693	0.956	0.000
PA14_63370	58.698	0.086	0.910	0.014	0.981	-0.199	0.796	PA14_63370	258.005	0.869	0.001
PA14_63380	138.058	-0.230	0.578	-0.331	0.313	-0.936	0.029	PA14_63380	321.702	1.976	0.000
PA14_63390	32.501	1.116	0.073	1.309	0.016	0.568	0.492	PA14_63390	87.839	0.844	0.022
PA14_63410	224.969	0.552	0.101	1.483	0.000	0.069	0.893	PA14_63410	352.147	0.852	0.001
PA14_63420	61.669	0.763	0.094	0.896	0.024	-0.593	0.357	PA14_63420	109.742	0.819	0.119
PA14_63430	126.713	0.788	0.046	1.583	0.000	0.783	0.098	PA14_63430	222.508	0.988	0.000
PA14_63440	44.835	0.741	0.156	0.804	0.076	0.264	0.720	PA14_63440	101.638	0.041	0.895
PA14_63450	385.960	1.394	0.000	2.148	0.000	2.468	0.000	PA14_63450	467.362	1.040	0.000
PA14_63460	173.081	1.129	0.002	1.966	0.000	0.066	0.915	#N/A	#N/A	#N/A	#N/A
PA14_63470	297.729	-0.230	0.601	-0.460	0.178	-1.324	0.002	PA14_63470	1215.401	-1.211	0.000

PA14_63480	58.870	0.346	0.564	0.527	0.261	0.638	0.299	PA14_63480	205.885	-0.883	0.002
PA14_63500	48.473	0.842	0.101	1.051	0.018	0.521	0.433	PA14_63500	96.368	0.390	0.297
PA14_63520	123.981	0.447	0.304	0.452	0.226	0.364	0.490	PA14_63520	220.347	0.780	0.005
PA14_63530	340.093	1.387	0.000	1.232	0.000	-0.234	0.587	PA14_63530	672.953	0.688	0.004
PA14_63540	309.030	0.998	0.001	0.818	0.003	-0.425	0.303	PA14_63540	775.081	0.318	0.181
PA14_63550	173.116	1.222	0.000	1.204	0.000	-1.905	0.000	PA14_63550	311.431	1.274	0.000
PA14_63570	864.491	1.656	0.000	1.630	0.000	-0.662	0.071	PA14_63570	971.393	0.865	0.000
PA14_63580	1574.067	1.545	0.000	0.830	0.002	-1.381	0.000	PA14_63580	1542.601	0.351	0.140
PA14_63605	7215.906	1.447	0.000	1.361	0.000	-0.215	0.565	PA14_63605	8955.441	0.117	0.610
PA14_63620	11.582	0.437	0.679	0.979	0.202	0.985	0.315	PA14_63620	32.471	-0.266	0.661
PA14_63640	32.235	-0.989	0.194	-0.883	0.184	1.249	0.106	PA14_63640	55.452	-1.576	0.002
PA14_63650	48.082	0.429	0.500	0.489	0.346	0.819	0.204	PA14_63650	107.153	1.964	0.000
PA14_63660	133.492	0.988	0.017	1.431	0.000	-0.252	0.694	PA14_63660	250.076	1.262	0.000
PA14_63680	68.760	0.798	0.089	1.904	0.000	1.275	0.013	PA14_63680	92.568	1.291	0.000
PA14_63700	14.448	0.565	0.534	1.129	0.097	-0.237	0.841	PA14_63700	36.954	0.285	0.663
PA14_63710	6.005	-0.686	0.559	-1.434	0.124	-1.235	0.287	PA14_63710	19.610	-0.376	0.628
PA14_63720	1.363	-0.470	0.779	0.058	0.969	-0.053	0.972	PA14_63720	12.989	-1.550	0.112
PA14_63730	116.140	0.964	0.015	2.031	0.000	-0.590	0.307	PA14_63730	70.890	0.680	0.101
PA14_63740	3.190	2.082	0.096	0.616	0.634	1.061	0.431	PA14_63740	106.546	-3.284	0.001
PA14_63750	43.003	0.623	0.273	1.480	0.001	-0.741	0.324	PA14_63750	1080.473	-4.189	0.000
PA14_63770	4.270	-0.157	0.923	-1.194	0.277	-0.583	0.672	PA14_63770	244.912	-4.859	0.000
PA14_63780	27.175	1.106	0.073	1.027	0.064	-0.543	0.548	PA14_63780	1742.014	-5.078	0.000
PA14_63800	241.863	0.225	0.637	-0.229	0.567	-1.420	0.002	PA14_63800	10152.233	-5.389	0.000
PA14_63820	212.085	0.593	0.088	0.992	0.001	0.781	0.049	PA14_63820	3720.885	-4.728	0.000
PA14_63830	139.208	0.088	0.885	0.332	0.426	0.786	0.116	PA14_63830	536.646	-0.902	0.000
PA14_63840	36.625	-0.741	0.317	-0.463	0.478	0.104	0.913	PA14_63840	133.421	-1.674	0.000
PA14_63850	199.243	-0.646	0.059	-0.904	0.003	0.083	0.868	PA14_63850	829.003	-1.517	0.000
PA14_63860	9.326	-0.190	0.882	-0.441	0.633	-0.600	0.611	PA14_63860	32.974	0.324	0.705
PA14_63880	63.019	0.730	0.108	0.749	0.062	-0.125	0.861	PA14_63880	176.806	0.346	0.278
PA14_63890	38.359	0.380	0.576	0.564	0.287	-0.696	0.379	PA14_63890	66.432	-0.111	0.792
PA14_63900	200.423	-0.150	0.720	-0.317	0.313	0.234	0.596	PA14_63900	810.736	-1.156	0.000
PA14_63910	280.862	0.072	0.933	0.859	0.098	-0.027	0.975	PA14_63910	102.101	-0.021	1.000
PA14_63920	288.644	0.415	0.470	0.325	0.505	0.236	0.744	PA14_63920	157.319	-0.863	0.004
PA14_63940	547.648	0.123	0.848	0.023	0.966	-0.361	0.595	PA14_63940	383.669	-2.146	0.000
PA14_63960	1620.611	0.351	0.466	1.223	0.001	0.000	1.000	PA14_63960	1716.315	-3.931	0.000
PA14_63970	1050.156	0.809	0.138	1.256	0.008	0.295	0.683	PA14_63970	647.187	-0.344	0.237
PA14_63990	521.022	-0.582	0.045	-0.411	0.130	-0.465	0.194	PA14_63990	922.447	0.105	0.674
PA14_64000	71.506	-0.368	0.459	-0.352	0.391	0.129	0.837	PA14_64000	171.165	-0.241	0.457
PA14_64010	116.948	-0.345	0.447	-0.130	0.750	-0.267	0.624	PA14_64010	597.176	0.251	0.344
PA14_64030	1999.991	-0.965	0.000	-1.042	0.000	-0.123	0.761	PA14_64030	6386.032	-0.735	0.000
PA14_64050	1167.417	-0.948	0.004	-2.291	0.000	0.800	0.044	PA14_64050	4134.897	-2.316	0.000
PA14_64060	42.848	-0.286	0.686	0.198	0.724	2.153	0.000	PA14_64060	206.933	-1.339	0.000
PA14_64080	280.594	-0.005	0.990	0.283	0.323	-0.359	0.358	PA14_64080	477.726	0.989	0.000
PA14_64090	3170.127	0.844	0.001	1.466	0.000	0.585	0.071	PA14_64090	4174.081	1.312	0.000
PA14_64100	5208.997	0.126	0.722	-0.483	0.059	-1.709	0.000	PA14_64100	3947.273	0.817	0.026
PA14_64110	2845.465	-0.419	0.273	-0.952	0.002	-1.741	0.000	PA14_64110	1918.277	0.795	0.008
PA14_64120	37.582	0.409	0.517	0.274	0.612	0.247	0.748	PA14_64120	54.832	1.647	0.000
PA14_64140	628.518	0.847	0.017	1.181	0.000	0.128	0.810	PA14_64140	858.402	0.668	0.004
PA14_64170	754.338	0.672	0.014	0.713	0.005	0.280	0.450	PA14_64170	1371.225	0.973	0.000
PA14_64180	3638.277	0.921	0.000	1.788	0.000	1.320	0.000	PA14_64180	2894.758	1.247	0.000
PA14_64190	404.832	0.338	0.369	0.967	0.001	0.317	0.471	PA14_64190	581.360	0.963	0.000
PA14_64200	1127.320	0.714	0.022	1.062	0.000	-0.069	0.886	PA14_64200	596.762	1.370	0.000
PA14_64220	497.119	0.697	0.007	1.228	0.000	-1.198	0.000	PA14_64220	234.364	1.486	0.000
PA14_64230	1158.083	0.124	0.718	0.398	0.112	-0.361	0.286	PA14_64230	2276.825	-0.261	0.243
PA14_64240	67.068	0.314	0.577	-0.020	0.970	0.665	0.240	PA14_64240	216.531	-1.194	0.000
PA14_64260	7.692	-0.249	0.857	1.190	0.169	0.810	0.486	PA14_64260	7.449	0.502	0.713
PA14_64270	32.315	0.051	0.949	-0.641	0.225	-0.181	0.825	PA14_64270	78.745	-0.126	0.754
PA14_64280	5.182	-1.206	0.316	-0.570	0.598	-0.323	0.817	PA14_64280	16.914	0.055	1.000
PA14_64290	6.099	0.205	0.897	1.375	0.167	0.949	0.450	PA14_64290	12.560	0.999	0.297

PA14_64300	39.491	-0.025	0.977	0.203	0.731	0.338	0.662	PA14_64300	76.689	0.404	0.328
PA14_64310	12.775	0.425	0.670	0.467	0.557	0.613	0.546	PA14_64310	46.923	-0.758	0.105
PA14_64320	113.514	-0.093	0.855	-0.674	0.045	-0.926	0.043	PA14_64320	257.238	1.163	0.000
PA14_64335	107.127	-0.137	0.782	-0.483	0.169	-1.502	0.001	PA14_64335	198.295	1.142	0.000
PA14_64350	85.927	-0.844	0.041	-1.729	0.000	-2.911	0.000	PA14_64350	120.963	0.971	0.003
PA14_64360	158.149	-0.738	0.024	-1.045	0.000	-2.411	0.000	PA14_64360	180.983	0.481	0.113
PA14_64370	117.932	-1.259	0.000	-1.777	0.000	-3.635	0.000	PA14_64370	142.540	0.060	0.899
PA14_64390	608.508	-0.511	0.182	-1.129	0.000	-2.418	0.000	PA14_64390	602.720	-0.244	0.380
PA14_64400	54.989	-1.310	0.002	-1.666	0.000	-2.145	0.000	PA14_64400	90.662	-0.405	0.323
PA14_64410	750.970	0.405	0.189	1.118	0.000	0.112	0.793	PA14_64410	2532.950	-1.829	0.000
PA14_64420	39.318	1.470	0.005	1.325	0.006	0.707	0.321	PA14_64420	61.013	0.917	0.031
PA14_64430	37.084	-0.449	0.522	-0.283	0.636	-0.260	0.754	PA14_64430	60.944	0.945	0.023
PA14_64440	365.785	0.764	0.014	0.529	0.076	-0.416	0.326	PA14_64440	842.921	-0.291	0.224
PA14_64450	58.281	0.544	0.304	1.320	0.001	0.747	0.200	PA14_64450	84.373	1.221	0.001
PA14_64460	5228.110	-1.083	0.024	-1.928	0.000	0.474	0.448	PA14_64460	19079.629	-2.971	0.000
PA14_64470	176.791	-0.507	0.329	-1.231	0.003	-0.488	0.414	PA14_64470	484.258	-1.710	0.000
PA14_64480	240.123	-2.344	0.000	-3.156	0.000	-1.323	0.027	PA14_64480	2742.258	-3.021	0.000
PA14_64490	293.055	-1.385	0.000	-1.253	0.000	0.220	0.606	PA14_64490	2539.229	-3.112	0.000
PA14_64500	6510.698	0.078	0.881	-1.077	0.001	0.673	0.123	PA14_64500	11054.451	-1.652	0.000
PA14_64510	762.511	-0.514	0.050	-0.474	0.047	0.603	0.045	PA14_64510	3101.771	-0.910	0.000
PA14_64520	2056.658	-1.379	0.000	-2.369	0.000	-0.534	0.248	PA14_64520	10085.484	-2.738	0.000
PA14_64530	14.077	-1.601	0.055	-0.724	0.336	0.514	0.599	PA14_64530	93.004	-1.834	0.000
PA14_64540	63.131	1.070	0.022	1.608	0.000	1.332	0.013	PA14_64540	94.778	0.260	0.520
PA14_64550	29.931	1.340	0.037	1.534	0.007	1.588	0.027	PA14_64550	40.001	0.599	0.271
PA14_64560	6.632	0.485	0.704	0.124	0.912	-0.447	0.740	PA14_64560	26.501	-0.964	0.116
PA14_64570	76.056	-0.258	0.614	0.475	0.211	-0.435	0.439	PA14_64570	164.780	-0.077	0.866
PA14_64580	50.260	0.608	0.262	0.568	0.226	-0.537	0.453	PA14_64580	118.359	0.301	0.383
PA14_64590	87.087	-0.312	0.592	1.053	0.010	0.549	0.350	PA14_64590	137.901	0.388	0.331
PA14_64610	1581.543	0.113	0.843	2.098	0.000	2.246	0.000	PA14_64610	219.000	0.737	0.019
PA14_64620	188.282	0.408	0.363	2.189	0.000	1.828	0.000	PA14_64620	128.294	2.822	0.000
PA14_64640	209.476	1.003	0.006	2.559	0.000	0.450	0.375	PA14_64640	313.564	1.565	0.000
PA14_64650	11.025	0.373	0.705	0.084	0.925	-2.323	0.025	PA14_64650	23.651	0.254	0.779
PA14_64660	19.439	0.551	0.496	-0.060	0.939	-1.265	0.172	PA14_64660	46.157	0.520	0.312
PA14_64670	69.883	0.478	0.285	-0.271	0.518	-0.672	0.221	PA14_64670	109.695	0.541	0.119
PA14_64680	4.953	1.305	0.260	0.584	0.598	-0.989	0.444	PA14_64680	12.298	0.291	0.835
PA14_64690	44.733	1.055	0.096	1.706	0.001	2.376	0.000	PA14_64690	29.383	0.160	0.805
PA14_64700	38.056	0.002	0.999	-0.029	0.970	1.180	0.117	PA14_64700	53.236	0.731	0.103
PA14_64710	199.825	-0.245	0.541	-0.379	0.233	1.516	0.000	PA14_64710	327.826	-1.033	0.000
PA14_64720	119.528	0.880	0.019	1.199	0.000	0.871	0.053	PA14_64720	169.841	0.193	0.571
PA14_64740	143.679	0.648	0.080	0.888	0.005	0.970	0.019	PA14_64740	425.074	0.139	0.632
PA14_64750	179.468	1.174	0.007	1.639	0.000	2.266	0.000	PA14_64750	340.767	-0.178	0.524
PA14_64770	30.384	0.270	0.720	0.605	0.270	0.386	0.628	PA14_64770	58.522	0.182	0.731
PA14_64780	201.908	1.381	0.005	2.066	0.000	0.924	0.135	PA14_64780	211.182	0.667	0.020
PA14_64790	22.539	0.506	0.492	0.983	0.078	0.535	0.519	PA14_64790	58.687	0.746	0.245
PA14_64800	40.049	0.993	0.078	1.888	0.000	0.173	0.843	PA14_64800	74.018	1.382	0.000
PA14_64810	19.108	0.686	0.400	1.022	0.114	-0.678	0.505	PA14_64810	43.411	0.153	0.779
PA14_64820	473.888	0.634	0.187	1.218	0.003	-0.656	0.250	PA14_64820	596.846	0.872	0.082
PA14_64840	1754.401	0.674	0.013	0.395	0.135	-0.977	0.002	PA14_64840	2796.342	0.724	0.001
PA14_64850	22.659	0.278	0.749	0.103	0.890	0.227	0.814	PA14_64850	60.074	-0.592	0.193
PA14_64860	61.722	0.122	0.838	-0.901	0.026	0.241	0.692	PA14_64860	136.071	-0.235	0.636
PA14_64870	44.324	0.068	0.926	-0.981	0.036	0.185	0.796	PA14_64870	118.836	0.240	0.482
PA14_64880	25.487	-0.605	0.363	-1.794	0.001	0.188	0.821	PA14_64880	114.727	-0.050	0.966
PA14_64890	23.024	-0.450	0.526	-1.546	0.006	-0.324	0.699	PA14_64890	99.781	-1.165	0.001
PA14_64900	281.533	-1.443	0.000	-3.187	0.000	-1.443	0.000	PA14_64900	2317.959	-2.579	0.000
PA14_64910	78.207	0.749	0.083	-0.467	0.268	-0.634	0.275	PA14_64910	243.754	-0.825	0.002
PA14_64920	683.450	-1.465	0.000	-2.259	0.000	-0.043	0.934	PA14_64920	3162.761	-2.722	0.000
PA14_64930	142.512	-1.386	0.000	-1.988	0.000	-2.016	0.000	PA14_64930	1359.515	-0.982	0.000
PA14_64940	2363.858	-0.073	0.849	-0.277	0.307	-0.560	0.091	PA14_64940	6149.809	-1.214	0.000
PA14_64950	910.027	2.327	0.000	3.605	0.000	-0.875	0.082	PA14_64950	1014.456	1.058	0.000

PA14_64960	1511.157	1.523	0.000	2.057	0.000	0.482	0.270	PA14_64960	4029.405	1.552	0.000
PA14_64980	576.915	0.961	0.001	0.970	0.001	-0.518	0.198	PA14_64980	2079.720	2.391	0.000
PA14_64990	66.960	0.829	0.095	0.957	0.028	-0.678	0.316	PA14_64990	306.951	1.058	0.000
PA14_65000	12253.115	-1.354	0.000	-2.866	0.000	-2.871	0.000	PA14_65000	42346.511	-0.371	0.117
PA14_65010	78.223	-0.020	0.976	0.322	0.451	-2.244	0.000	PA14_65010	104.550	0.907	0.010
PA14_65030	159.170	0.041	0.936	0.577	0.058	0.210	0.660	PA14_65030	494.528	-0.302	0.237
PA14_65040	59.608	-1.560	0.000	-3.118	0.000	-0.427	0.453	PA14_65040	640.883	-3.385	0.000
PA14_65050	117.320	-0.526	0.285	-0.358	0.412	0.705	0.180	PA14_65050	332.272	-1.110	0.000
PA14_65060	206.992	-0.079	0.875	0.476	0.144	0.751	0.066	PA14_65060	461.409	-0.526	0.034
PA14_65080	1647.521	0.763	0.002	1.225	0.000	2.016	0.000	PA14_65080	1111.711	1.203	0.000
PA14_65090	246.069	-2.565	0.000	-4.066	0.000	0.696	0.074	PA14_65090	1607.926	-3.594	0.000
PA14_65110	232.592	1.325	0.000	2.323	0.000	0.763	0.101	PA14_65110	252.090	1.249	0.000
PA14_65130	1262.861	0.508	0.077	1.427	0.000	1.264	0.000	PA14_65130	1541.605	0.466	0.039
PA14_65150	900.212	-0.298	0.653	-0.081	0.894	0.148	0.849	PA14_65150	1128.040	1.926	0.000
PA14_65160	7914.690	0.786	0.009	1.202	0.000	0.993	0.005	PA14_65160	4187.920	2.510	0.000
PA14_65170	9488.806	1.068	0.001	1.494	0.000	0.838	0.035	PA14_65170	3890.237	2.397	0.000
PA14_65180	9225.942	0.683	0.043	1.055	0.000	1.192	0.002	PA14_65180	4112.340	2.957	0.000
PA14_65190	355.482	-0.490	0.187	-0.564	0.082	0.152	0.758	PA14_65190	1419.553	0.198	0.434
PA14_65200	10047.089	0.232	0.483	0.088	0.774	1.057	0.001	PA14_65200	19678.566	0.040	0.866
PA14_65210	3710.057	0.449	0.265	2.032	0.000	0.913	0.031	#N/A	#N/A	#N/A	#N/A
PA14_65220	160.090	0.191	0.749	1.180	0.003	0.086	0.900	#N/A	#N/A	#N/A	#N/A
PA14_65230	7009.938	-0.135	0.752	-0.247	0.455	1.061	0.004	PA14_65230	5079.889	0.265	0.254
PA14_65250	4149.392	0.326	0.290	0.844	0.001	2.155	0.000	PA14_65250	4586.639	0.170	0.482
PA14_65260	1377.464	0.365	0.308	0.887	0.002	2.042	0.000	PA14_65260	1994.323	0.175	0.482
PA14_65270	6313.128	-0.374	0.184	0.606	0.012	0.190	0.599	PA14_65270	8300.799	0.964	0.000
PA14_65280	15289.915	0.098	0.818	1.216	0.000	0.754	0.037	PA14_65280	19908.230	1.211	0.000
PA14_65300	13486.057	0.739	0.013	1.700	0.000	1.611	0.000	PA14_65300	22028.839	0.912	0.000
PA14_65310	20189.049	0.709	0.172	1.645	0.000	1.707	0.002	PA14_65310	21679.263	0.340	0.118
PA14_65320	12372.567	0.479	0.147	0.848	0.003	2.378	0.000	PA14_65320	29327.569	-0.265	0.406
PA14_65350	1316.011	0.164	0.611	0.448	0.064	-0.543	0.086	PA14_65350	2624.971	0.228	0.331
PA14_65370	2196.637	0.246	0.437	0.677	0.006	0.587	0.069	PA14_65370	3914.721	-0.013	0.968
PA14_65380	322.257	0.232	0.521	0.803	0.003	0.482	0.195	PA14_65380	1213.855	0.314	0.178
PA14_65390	442.865	0.433	0.175	1.208	0.000	1.024	0.002	PA14_65390	1059.912	0.482	0.033
PA14_65400	415.887	0.603	0.053	0.258	0.401	0.817	0.022	PA14_65400	748.619	-0.344	0.158
PA14_65410	614.963	-0.012	0.977	0.168	0.557	0.409	0.236	PA14_65410	1889.348	-1.128	0.000
PA14_65420	222.556	0.622	0.091	1.116	0.000	0.685	0.112	PA14_65420	667.542	0.875	0.000
PA14_65430	669.453	0.176	0.598	0.009	0.977	0.062	0.880	PA14_65430	1343.868	0.900	0.000
PA14_65450	985.580	-0.025	0.957	0.103	0.745	0.048	0.914	PA14_65450	2758.949	0.153	0.528
PA14_65470	416.303	-0.669	0.045	-0.814	0.006	-1.057	0.006	PA14_65470	1484.937	-0.469	0.036
PA14_65480	834.657	0.239	0.575	1.331	0.000	0.652	0.120	PA14_65480	1353.262	0.826	0.000
PA14_65500	270.675	-0.235	0.531	-0.037	0.916	0.013	0.977	PA14_65500	587.005	1.152	0.000
PA14_65520	1354.116	-0.202	0.660	-0.235	0.535	0.409	0.384	PA14_65520	2385.721	-0.030	0.920
PA14_65540	3577.840	0.256	0.428	0.525	0.042	0.262	0.479	PA14_65540	6089.769	1.152	0.000
PA14_65560	1322.026	0.910	0.002	1.953	0.000	1.130	0.001	PA14_65560	1710.324	1.019	0.000
PA14_65570	741.006	0.316	0.393	0.506	0.094	-0.311	0.471	PA14_65570	2111.882	0.587	0.007
PA14_65580	14.119	1.372	0.101	2.091	0.003	1.727	0.054	PA14_65580	49.915	1.892	0.000
PA14_65590	705.518	0.208	0.540	0.516	0.047	0.652	0.048	PA14_65590	1911.582	0.315	0.244
PA14_65605	5446.259	0.283	0.512	0.595	0.080	-0.789	0.065	PA14_65605	9857.075	-0.490	0.022
PA14_65630	380.544	0.452	0.134	0.697	0.007	-2.128	0.000	PA14_65630	845.883	-0.710	0.005
PA14_65640	298.573	0.047	0.913	0.019	0.955	-2.210	0.000	PA14_65640	665.728	-0.186	0.527
PA14_65660	1392.800	0.106	0.773	-0.181	0.528	-1.678	0.000	PA14_65660	2529.075	0.889	0.000
PA14_65670	458.892	0.567	0.083	0.676	0.020	-0.169	0.714	PA14_65670	1443.581	0.806	0.000
PA14_65690	114.777	-0.712	0.065	-0.106	0.788	-2.238	0.000	PA14_65690	364.688	0.179	0.513
PA14_65700	414.309	0.463	0.173	1.085	0.000	-0.436	0.300	PA14_65700	1327.068	0.197	0.400
PA14_65710	941.351	-0.124	0.757	0.118	0.717	-2.081	0.000	PA14_65710	3875.890	-0.182	0.414
PA14_65720	1108.485	0.419	0.175	0.170	0.574	0.462	0.198	PA14_65720	4001.588	0.075	0.730
PA14_65740	1580.557	-0.782	0.001	-0.761	0.001	-0.910	0.002	PA14_65740	1111.889	-0.218	0.371
PA14_65750	3276.615	0.415	0.237	0.708	0.017	-1.503	0.000	PA14_65750	3751.422	0.712	0.001
PA14_65760	67.982	0.254	0.656	0.725	0.077	-0.052	0.943	PA14_65760	183.790	0.994	0.001

PA14_65770	116.131	0.420	0.344	0.084	0.847	0.068	0.911	PA14_65770	265.419	0.241	0.415
PA14_65795	96.373	0.211	0.675	-0.126	0.766	-0.945	0.063	PA14_65795	194.048	0.740	0.010
PA14_65810	98.159	0.034	0.949	-0.127	0.742	-0.418	0.399	PA14_65810	157.698	1.229	0.000
PA14_65820	160.903	0.193	0.648	-0.212	0.543	-0.234	0.626	PA14_65820	238.464	1.633	0.000
PA14_65840	35.557	-0.363	0.589	-0.455	0.398	-0.587	0.422	PA14_65840	103.690	1.788	0.000
PA14_65850	42.133	-0.444	0.459	-0.117	0.829	0.283	0.685	PA14_65850	136.806	-0.858	0.056
PA14_65860	81.832	0.964	0.019	0.920	0.014	-0.442	0.478	PA14_65860	268.811	0.434	0.128
PA14_65870	190.985	0.850	0.084	1.339	0.002	0.106	0.884	PA14_65870	309.075	0.529	0.056
PA14_65880	101.826	0.511	0.316	0.733	0.081	-0.024	0.973	PA14_65880	241.365	1.060	0.000
PA14_65900	170.710	0.996	0.002	1.500	0.000	-0.012	0.983	PA14_65900	429.299	0.523	0.040
PA14_65920	228.085	3.026	0.000	4.225	0.000	0.607	0.277	PA14_65920	530.833	3.778	0.000
PA14_65940	175.225	1.608	0.000	1.360	0.000	-1.480	0.008	PA14_65940	702.610	3.582	0.000
PA14_65950	92.433	-0.023	0.972	-0.050	0.913	-1.337	0.011	PA14_65950	349.806	-0.067	0.873
PA14_65960	148.060	0.402	0.334	0.914	0.005	-0.342	0.512	PA14_65960	315.223	0.921	0.003
PA14_65970	31.258	0.758	0.252	1.357	0.010	0.444	0.599	PA14_65970	120.128	0.321	0.381
PA14_65990	6.017	2.103	0.041	1.458	0.137	1.452	0.217	PA14_65990	13.949	-0.813	0.370
PA14_66000	145.765	0.167	0.766	0.239	0.582	-0.575	0.296	PA14_66000	334.936	1.120	0.000
PA14_66010	74.181	0.316	0.593	0.259	0.601	-0.432	0.521	PA14_66010	148.858	0.766	0.015
PA14_66020	218.529	-0.371	0.297	-0.282	0.368	-0.139	0.763	PA14_66020	560.691	-1.068	0.000
PA14_66040	111.621	0.009	0.987	1.158	0.001	1.174	0.007	PA14_66040	342.581	0.181	0.493
PA14_66050	194.864	0.290	0.483	1.927	0.000	1.637	0.000	PA14_66050	292.168	0.190	0.498
PA14_66060	3903.643	0.611	0.083	1.032	0.001	0.745	0.067	PA14_66060	5339.390	-0.618	0.003
PA14_66080	3776.994	0.816	0.000	1.550	0.000	1.018	0.000	PA14_66080	4535.488	2.325	0.000
PA14_66090	573.390	0.319	0.310	0.937	0.000	0.776	0.016	PA14_66090	1193.845	1.398	0.000
PA14_66100	1446.150	0.945	0.000	1.421	0.000	0.580	0.085	PA14_66100	1899.235	0.491	0.026
PA14_66110	1598.803	-0.170	0.665	-0.373	0.215	-0.791	0.029	PA14_66110	5572.845	-0.944	0.000
PA14_66120	2386.405	0.114	0.794	0.048	0.898	-0.687	0.077	PA14_66120	3879.474	-0.306	0.336
PA14_66140	2419.530	0.075	0.845	0.677	0.007	-0.507	0.134	PA14_66140	2119.098	0.635	0.004
PA14_66150	1073.306	0.397	0.223	1.199	0.000	0.331	0.398	PA14_66150	1560.968	0.103	0.700
PA14_66160	978.920	0.447	0.143	1.312	0.000	0.179	0.662	PA14_66160	1249.973	0.511	0.025
PA14_66170	6939.363	0.352	0.203	0.662	0.005	-0.118	0.750	PA14_66170	8586.216	1.190	0.000
PA14_66190	991.680	0.272	0.501	0.713	0.023	-0.117	0.818	PA14_66190	2230.710	0.840	0.000
PA14_66200	1303.700	0.226	0.546	0.661	0.020	-0.539	0.158	PA14_66200	1592.478	1.388	0.000
PA14_66210	472.880	0.376	0.285	0.734	0.011	0.097	0.841	PA14_66210	1042.908	1.083	0.000
PA14_66220	795.522	0.288	0.371	0.759	0.003	-0.184	0.645	PA14_66220	1600.549	1.511	0.000
PA14_66230	2402.716	0.299	0.307	0.674	0.005	-0.592	0.060	PA14_66230	4109.471	1.919	0.000
PA14_66240	1602.988	0.559	0.055	1.087	0.000	0.034	0.938	PA14_66240	2817.060	1.135	0.000
PA14_66250	1240.793	0.586	0.066	1.500	0.000	0.601	0.112	PA14_66250	1778.428	0.247	0.322
PA14_66260	18487.481	0.640	0.163	0.871	0.030	0.384	0.504	PA14_66260	15687.157	0.779	0.000
PA14_66270	4802.485	0.877	0.007	1.360	0.000	1.510	0.000	PA14_66270	7583.830	0.977	0.000
PA14_66290	36548.056	2.557	0.000	2.108	0.000	1.031	0.000	PA14_66290	17257.182	1.651	0.000
PA14_66310	10877.669	2.166	0.000	1.907	0.000	0.513	0.071	PA14_66310	5751.129	0.538	0.011
PA14_66320	1546.653	-0.112	0.748	-0.497	0.043	0.650	0.035	PA14_66320	4734.113	0.010	0.961
PA14_66330	2159.399	0.056	0.886	-0.381	0.147	-0.331	0.348	PA14_66330	3438.274	0.101	0.653
PA14_66340	181.490	-0.230	0.577	-0.024	0.951	0.848	0.026	PA14_66340	884.327	-1.430	0.000
PA14_66350	290.305	1.133	0.002	1.715	0.000	4.187	0.000	PA14_66350	170.563	0.928	0.002
PA14_66380	358.745	0.204	0.604	0.436	0.148	-0.979	0.011	PA14_66380	1007.963	1.516	0.000
PA14_66400	2258.247	0.043	0.932	0.340	0.313	-0.483	0.257	PA14_66400	3731.151	0.545	0.012
PA14_66410	306.798	0.625	0.058	1.132	0.000	1.679	0.000	PA14_66410	1331.071	-2.885	0.000
PA14_66420	53.523	-2.602	0.000	-2.358	0.000	-1.780	0.002	PA14_66420	46.802	0.320	0.718
PA14_66440	2631.916	-1.229	0.000	-2.244	0.000	-4.130	0.000	PA14_66440	2607.095	-0.705	0.001
PA14_66450	97.708	-1.593	0.000	-3.084	0.000	-2.423	0.000	PA14_66450	2298.290	-5.199	0.000
PA14_66460	2737.899	-0.514	0.137	-2.157	0.000	-3.556	0.000	PA14_66460	12988.977	-1.441	0.000
PA14_66480	1922.516	0.555	0.077	0.932	0.001	0.995	0.004	PA14_66480	3871.584	-0.491	0.024
PA14_66490	113.505	0.663	0.093	0.547	0.128	0.475	0.340	PA14_66490	202.019	0.267	0.386
PA14_66510	470.600	0.410	0.240	0.914	0.001	3.398	0.000	PA14_66510	1700.111	-2.967	0.000
PA14_66520	26.147	0.005	0.996	0.470	0.422	0.703	0.348	PA14_66520	63.532	-0.627	0.157
PA14_66530	7.738	-1.056	0.299	-0.659	0.458	-0.245	0.845	PA14_66530	31.859	-1.518	0.006
PA14_66540	14.639	-0.725	0.405	-1.562	0.027	-0.383	0.708	PA14_66540	50.933	-1.275	0.012

PA14_66550	484.462	-0.266	0.405	-0.106	0.718	-0.035	0.934	PA14_66550	571.050	0.980	0.000
PA14_66560	704.847	0.014	0.979	-0.261	0.464	0.069	0.896	PA14_66560	941.919	-0.252	0.301
PA14_66570	2458.276	0.925	0.000	1.079	0.000	1.938	0.000	PA14_66570	1927.724	0.914	0.000
PA14_66580	2324.374	-0.005	0.989	0.063	0.818	-0.272	0.397	PA14_66580	5450.198	0.465	0.031
PA14_66600	4421.232	0.492	0.239	1.043	0.003	1.055	0.017	PA14_66600	7418.702	0.229	0.582
PA14_66610	573.289	0.146	0.681	0.095	0.750	-0.441	0.208	PA14_66610	2924.966	-0.160	0.487
PA14_66620	4563.049	-0.506	0.064	-1.191	0.000	-1.177	0.000	PA14_66620	18932.418	0.887	0.000
PA14_66630	1550.338	-0.472	0.105	-0.946	0.000	-1.200	0.000	PA14_66630	8454.019	1.499	0.000
PA14_66640	1007.053	-0.098	0.770	-0.564	0.015	-0.770	0.009	PA14_66640	4482.682	1.203	0.000
PA14_66650	680.240	-0.091	0.818	-0.783	0.002	-1.557	0.000	PA14_66650	1687.198	0.416	0.064
PA14_66660	1762.802	-0.328	0.407	-1.372	0.000	-1.399	0.000	PA14_66660	6064.894	-0.674	0.006
PA14_66670	7005.847	0.738	0.068	1.588	0.000	3.276	0.000	PA14_66670	5807.222	0.332	0.130
PA14_66680	7023.118	0.856	0.004	0.946	0.001	0.314	0.440	PA14_66680	5722.472	1.704	0.000
PA14_66690	1255.160	0.114	0.770	0.309	0.284	1.173	0.000	PA14_66690	3429.980	-1.528	0.000
PA14_66700	404.819	-0.335	0.429	0.040	0.924	0.045	0.935	PA14_66700	1968.766	-1.474	0.000
PA14_66710	1553.343	0.414	0.199	2.726	0.000	0.190	0.655	PA14_66710	1604.626	1.621	0.000
PA14_66720	414.958	0.636	0.027	0.723	0.005	0.272	0.487	PA14_66720	569.238	1.837	0.000
PA14_66750	2230.552	-0.179	0.549	-0.423	0.067	-1.182	0.000	PA14_66750	5202.524	0.291	0.192
PA14_66760	351.008	-0.156	0.666	-0.488	0.066	-0.845	0.013	PA14_66760	842.975	0.489	0.041
PA14_66770	2116.332	-0.076	0.871	0.653	0.030	-1.848	0.000	PA14_66770	7130.484	2.065	0.000
PA14_66790	1988.188	-0.452	0.110	0.300	0.259	-2.726	0.000	PA14_66790	7233.382	2.709	0.000
PA14_66800	58.999	-0.315	0.605	0.627	0.160	-1.273	0.049	PA14_66800	178.790	1.432	0.000
PA14_66820	1405.390	-0.988	0.000	-1.668	0.000	-0.512	0.112	PA14_66820	3793.344	-0.852	0.000
PA14_66830	194.118	-0.890	0.011	-1.814	0.000	-1.492	0.000	PA14_66830	566.230	-0.224	0.464
PA14_66840	885.301	-2.736	0.000	-3.557	0.000	-1.748	0.000	PA14_66840	2525.193	-2.425	0.000
PA14_66850	95.226	-2.910	0.000	-2.805	0.000	-1.769	0.000	PA14_66850	242.524	-1.756	0.000
PA14_66875	38174.078	0.252	0.666	0.130	0.800	0.923	0.092	PA14_66875	69072.743	-1.125	0.001
PA14_66880	5537.419	-0.590	0.037	-1.119	0.000	0.167	0.674	PA14_66880	22668.910	-1.659	0.000
PA14_66890	2040.597	1.372	0.086	2.154	0.002	0.492	0.630	PA14_66890	7038.647	-0.872	0.109
PA14_66900	548.659	0.134	0.728	0.578	0.033	-0.084	0.851	PA14_66900	914.279	-0.048	0.866
PA14_66910	117.698	0.003	0.996	0.027	0.950	-0.367	0.471	PA14_66910	244.008	0.140	0.680
PA14_66920	643.648	0.191	0.581	0.236	0.406	0.005	0.991	PA14_66920	730.914	1.018	0.000
PA14_66940	1905.851	0.227	0.578	0.085	0.818	0.040	0.938	PA14_66940	4278.225	-0.213	0.434
PA14_66950	1793.702	0.728	0.010	0.707	0.008	1.469	0.000	PA14_66950	4212.671	-0.013	0.981
PA14_66960	2398.412	-0.065	0.846	-0.764	0.000	-0.919	0.001	PA14_66960	5615.224	-0.167	0.494
PA14_66970	663.568	0.311	0.324	-0.113	0.707	-0.381	0.291	PA14_66970	1654.729	0.185	0.439
PA14_66980	501.427	0.566	0.060	0.773	0.004	-0.203	0.629	PA14_66980	1039.020	1.382	0.000
PA14_66990	188.643	0.977	0.002	1.418	0.000	0.040	0.943	PA14_66990	253.642	1.561	0.001
PA14_67010	341.934	0.618	0.045	0.679	0.014	-0.641	0.097	PA14_67010	453.465	0.934	0.000
PA14_67020	851.254	0.415	0.171	0.418	0.122	0.324	0.383	PA14_67020	2311.408	-0.399	0.073
PA14_67030	125.049	-0.111	0.844	-0.250	0.548	-2.676	0.000	PA14_67030	249.801	0.147	0.797
PA14_67040	297.469	0.506	0.132	0.278	0.383	-0.868	0.028	PA14_67040	413.110	0.500	0.050
PA14_67050	2416.634	-0.191	0.621	-0.307	0.319	-0.190	0.663	PA14_67050	3029.056	-0.004	1.000
PA14_67065	3340.436	0.236	0.531	0.730	0.011	-0.387	0.334	PA14_67065	3755.284	0.652	0.002
PA14_67090	6080.884	0.014	0.974	0.012	0.967	-0.185	0.597	PA14_67090	11232.837	0.133	0.567
PA14_67100	191.133	-0.193	0.633	-0.572	0.057	-1.501	0.000	PA14_67100	677.888	-0.822	0.000
PA14_67110	397.344	-0.048	0.908	-0.160	0.581	-0.912	0.007	PA14_67110	1022.942	-0.185	0.458
PA14_67120	37.905	1.085	0.062	1.424	0.004	1.359	0.037	PA14_67120	131.931	-0.833	0.010
PA14_67130	120.980	0.361	0.447	0.040	0.931	-0.200	0.740	PA14_67130	444.879	-0.283	0.286
PA14_67140	97.533	0.335	0.601	0.754	0.119	1.180	0.046	PA14_67140	354.700	-0.998	0.000
PA14_67150	63.671	-0.169	0.802	0.693	0.121	0.449	0.483	PA14_67150	749.515	-3.228	0.000
PA14_67170	121.193	0.075	0.885	0.277	0.432	-0.701	0.140	PA14_67170	307.696	0.948	0.000
PA14_67180	1178.514	0.243	0.423	-0.276	0.281	-0.729	0.018	PA14_67180	3951.259	0.861	0.000
PA14_67190	891.528	0.143	0.730	-0.139	0.682	-0.094	0.845	PA14_67190	2697.647	1.010	0.000
PA14_67200	757.433	-0.052	0.899	-0.544	0.035	-0.425	0.226	PA14_67200	3306.749	0.020	0.925
PA14_67210	1203.243	0.014	0.974	-0.701	0.005	-0.712	0.027	PA14_67210	5021.446	-0.943	0.001
PA14_67220	693.653	0.105	0.833	-0.093	0.814	0.136	0.793	PA14_67220	1252.149	-0.902	0.000
PA14_67230	286.053	-0.213	0.556	-0.442	0.115	0.395	0.291	PA14_67230	412.007	0.363	0.158
PA14_67240	246.373	-0.930	0.001	-1.721	0.000	-1.214	0.000	PA14_67240	384.073	-0.980	0.000

PA14_67250	306.932	-0.641	0.146	-0.727	0.062	-1.659	0.000	PA14_67250	397.844	-0.230	0.400
PA14_67260	125.869	-1.445	0.000	-2.390	0.000	-1.598	0.000	PA14_67260	229.573	-0.325	0.275
PA14_67270	203.930	-1.590	0.000	-2.916	0.000	-1.738	0.000	PA14_67270	420.170	-0.173	0.553
PA14_67280	426.786	-1.619	0.000	-2.944	0.000	-1.458	0.000	PA14_67280	742.478	-1.077	0.000
PA14_67300	614.817	-1.724	0.000	-2.986	0.000	-2.211	0.000	PA14_67300	721.685	-1.671	0.000
PA14_67310	160.940	-1.727	0.000	-3.935	0.000	-3.990	0.000	PA14_67310	91.520	0.101	0.849
PA14_67320	474.703	-1.696	0.000	-3.918	0.000	-3.411	0.000	PA14_67320	269.788	-0.291	0.287
PA14_67340	315.974	-1.780	0.000	-3.736	0.000	-3.795	0.000	PA14_67340	176.099	-0.480	0.106
PA14_67350	861.190	-2.289	0.000	-4.628	0.000	-4.728	0.000	PA14_67350	588.857	-2.104	0.000
PA14_67370	19.025	-2.529	0.001	-2.029	0.003	-0.567	0.550	PA14_67370	126.781	-3.860	0.000
PA14_67380	16.032	-1.045	0.175	-0.537	0.434	-0.121	0.907	PA14_67380	44.829	-0.890	0.067
PA14_67400	416.956	-1.832	0.000	-1.723	0.000	0.293	0.477	PA14_67400	1721.790	-1.889	0.000
PA14_67410	54.799	-1.508	0.000	-2.016	0.000	-1.585	0.003	PA14_67410	228.052	-0.883	0.001
PA14_67420	855.884	-1.290	0.000	-1.805	0.000	-1.918	0.000	PA14_67420	1950.104	-0.692	0.001
PA14_67440	398.439	-1.594	0.000	-2.960	0.000	-3.703	0.000	PA14_67440	707.087	-1.234	0.000
PA14_67450	756.531	-0.075	0.902	1.473	0.000	1.175	0.014	PA14_67450	2194.484	-0.351	0.137
PA14_67460	311.098	-0.368	0.261	0.168	0.579	0.131	0.758	PA14_67460	2158.301	-0.871	0.000
PA14_67470	88.076	0.091	0.880	0.719	0.053	0.230	0.698	PA14_67470	341.358	0.768	0.003
PA14_67490	1997.321	-0.132	0.728	-0.053	0.871	0.080	0.854	PA14_67490	5463.447	0.487	0.024
PA14_67500	402.165	-0.534	0.089	0.071	0.832	-0.363	0.357	PA14_67500	1981.528	-1.350	0.000
PA14_67510	800.211	-0.157	0.690	-1.547	0.000	-0.966	0.006	PA14_67510	2578.112	0.042	0.866
PA14_67520	648.303	-0.322	0.335	-0.945	0.000	-1.473	0.000	PA14_67520	3456.326	-1.886	0.000
PA14_67530	172.872	-0.561	0.121	-0.069	0.854	-0.739	0.085	PA14_67530	647.008	-0.049	0.890
PA14_67540	66.832	-0.402	0.468	-0.677	0.124	0.005	0.995	PA14_67540	373.525	-1.268	0.000
PA14_67550	38.543	-1.279	0.024	-1.793	0.000	-0.271	0.727	PA14_67550	220.073	-1.326	0.000
PA14_67560	3285.712	0.607	0.030	1.081	0.000	0.965	0.002	PA14_67560	1597.881	1.458	0.000
PA14_67580	2893.712	1.390	0.000	2.524	0.000	3.062	0.000	PA14_67580	1547.869	2.122	0.000
PA14_67600	13416.819	-0.314	0.280	0.772	0.001	-1.682	0.000	PA14_67600	30717.377	1.361	0.000
PA14_67620	15.059	-0.436	0.691	0.859	0.272	-0.517	0.653	PA14_67620	53.206	0.902	0.047
PA14_67630	328.635	0.397	0.256	0.847	0.003	0.195	0.666	PA14_67630	660.317	0.291	0.269
PA14_67640	382.008	0.198	0.563	-0.385	0.153	-1.230	0.000	PA14_67640	1485.194	-0.598	0.006
PA14_67650	100.847	-0.289	0.524	-1.168	0.001	-1.649	0.001	PA14_67650	384.289	-0.326	0.216
PA14_67670	1067.708	-0.052	0.922	1.526	0.000	-0.418	0.371	PA14_67670	1701.102	1.062	0.000
PA14_67680	646.440	-0.338	0.300	0.650	0.014	-1.657	0.000	PA14_67680	1305.803	1.421	0.000
PA14_67700	238.950	0.653	0.063	0.684	0.030	0.218	0.656	PA14_67700	529.187	-0.689	0.015
PA14_67710	65.118	0.265	0.614	0.467	0.243	0.553	0.301	PA14_67710	168.232	0.288	0.390
PA14_67720	1934.439	-0.117	0.740	-0.629	0.011	-0.878	0.004	PA14_67720	2496.400	0.225	0.345
PA14_67740	1522.691	1.054	0.000	1.188	0.000	1.314	0.000	PA14_67740	1806.621	0.966	0.000
PA14_67750	621.397	0.589	0.048	0.593	0.029	0.711	0.040	PA14_67750	1214.515	1.054	0.000
PA14_67770	1043.041	-0.089	0.827	-0.479	0.080	-1.143	0.001	PA14_67770	3028.484	-1.172	0.000
PA14_67780	28.181	-0.308	0.687	-0.199	0.751	-0.605	0.455	PA14_67780	74.322	0.341	0.453
PA14_67790	941.290	0.161	0.652	0.498	0.059	-0.255	0.508	PA14_67790	3261.769	0.364	0.101
PA14_67810	1450.696	0.142	0.674	-0.022	0.944	-1.055	0.000	PA14_67810	2747.709	0.516	0.016
PA14_67820	153.368	0.604	0.085	0.827	0.007	-1.623	0.001	PA14_67820	290.536	1.091	0.001
PA14_67830	822.417	0.056	0.896	0.186	0.542	-1.693	0.000	PA14_67830	1022.610	0.931	0.001
PA14_67840	238.299	0.030	0.949	-0.045	0.901	-1.750	0.000	PA14_67840	390.693	0.360	0.177
PA14_67850	1644.868	1.595	0.000	2.401	0.000	0.840	0.139	PA14_67850	1529.694	1.039	0.000
PA14_67860	449.088	0.892	0.002	2.122	0.000	-0.816	0.038	PA14_67860	378.735	2.081	0.000
PA14_67880	645.067	0.477	0.096	0.743	0.003	-0.875	0.009	PA14_67880	616.601	0.934	0.000
PA14_67890	518.483	0.421	0.146	0.612	0.014	-0.958	0.004	PA14_67890	589.582	0.839	0.000
PA14_67900	210.177	0.353	0.417	0.676	0.049	-0.058	0.921	PA14_67900	447.555	0.835	0.001
PA14_67920	664.993	0.335	0.309	0.312	0.280	-0.526	0.155	PA14_67920	1265.247	0.805	0.000
PA14_67930	2222.401	0.455	0.161	0.897	0.001	0.050	0.915	PA14_67930	4100.124	1.017	0.000
PA14_67940	13.091	0.924	0.282	0.305	0.716	0.091	0.940	PA14_67940	48.353	0.370	0.450
PA14_67960	109.022	0.891	0.027	0.791	0.032	-1.104	0.051	PA14_67960	322.194	1.045	0.010
PA14_67970	127.753	0.524	0.203	0.997	0.003	-0.427	0.431	PA14_67970	246.792	0.633	0.021
PA14_67975	1693.487	0.492	0.065	0.609	0.011	-0.290	0.399	PA14_67975	2759.525	0.572	0.008
PA14_67990	1390.227	0.288	0.390	0.338	0.232	-1.295	0.000	PA14_67990	1913.902	0.237	0.346
PA14_68000	531.806	0.218	0.626	-0.411	0.242	-2.370	0.000	PA14_68000	1193.973	0.019	0.933

PA14_68010	131.439	0.364	0.411	0.542	0.128	-1.365	0.007	#N/A	#N/A	#N/A	#N/A
PA14_68020	422.080	0.538	0.111	1.151	0.000	-0.464	0.274	#N/A	#N/A	#N/A	#N/A
PA14_68030	13.917	1.325	0.145	1.641	0.036	-0.254	0.847	#N/A	#N/A	#N/A	#N/A
PA14_68040	222.600	-0.206	0.626	-0.204	0.556	1.387	0.000	PA14_68040	556.177	-0.344	0.158
PA14_68050	106.008	0.299	0.533	0.871	0.013	0.020	0.974	PA14_68050	179.133	0.340	0.296
PA14_68060	5998.797	0.288	0.581	-0.666	0.100	-1.444	0.003	PA14_68060	12163.585	-0.188	0.490
PA14_68070	1277.021	0.015	0.974	-2.672	0.000	-3.099	0.000	PA14_68070	1972.064	0.333	0.128
PA14_68080	189.888	0.965	0.002	-0.975	0.001	-1.274	0.002	PA14_68080	263.055	1.340	0.000
PA14_68090	86.876	0.470	0.314	-1.932	0.000	-1.912	0.000	PA14_68090	120.337	2.372	0.000
PA14_68100	40.542	1.612	0.001	1.160	0.011	-0.088	0.918	PA14_68100	48.387	2.040	0.000
PA14_68110	180.948	2.389	0.000	2.098	0.000	0.822	0.099	PA14_68110	296.214	-0.510	0.071
PA14_68120	311.941	2.068	0.000	1.487	0.000	-0.041	0.944	PA14_68120	403.703	0.239	0.372
PA14_68130	578.595	1.868	0.000	1.075	0.001	-0.553	0.235	PA14_68130	814.174	1.294	0.000
PA14_68140	492.923	1.774	0.000	1.989	0.000	0.347	0.453	PA14_68140	546.140	1.878	0.000
PA14_68150	23.299	0.415	0.641	2.222	0.000	0.336	0.741	#N/A	#N/A	#N/A	#N/A
PA14_68170	1163.309	-0.781	0.008	-0.717	0.009	-0.722	0.043	PA14_68170	4168.265	-0.343	0.138
PA14_68190	710.507	-0.369	0.288	-0.175	0.590	-0.078	0.869	PA14_68190	2412.854	-0.466	0.038
PA14_68200	1290.287	-0.858	0.001	-0.954	0.000	-1.627	0.000	PA14_68200	3366.618	-0.404	0.077
PA14_68210	462.225	-0.759	0.014	-0.668	0.020	-1.496	0.000	PA14_68210	1065.685	-0.485	0.146
PA14_68230	953.864	0.439	0.121	0.710	0.004	0.271	0.452	PA14_68230	2471.963	0.115	0.647
PA14_68250	557.165	0.173	0.617	-0.059	0.851	-0.757	0.024	PA14_68250	1261.766	0.807	0.000
PA14_68260	2474.114	-0.744	0.005	0.131	0.650	0.341	0.331	PA14_68260	4513.796	0.228	0.321
PA14_68280	140.178	-0.552	0.205	0.500	0.172	1.010	0.022	PA14_68280	256.797	0.462	0.092
PA14_68290	255.858	-0.377	0.400	1.147	0.001	-0.396	0.444	PA14_68290	407.966	0.851	0.001
PA14_68300	27042.517	-0.271	0.553	-1.469	0.000	-2.617	0.000	PA14_68300	105286.655	0.007	0.983
PA14_68330	10837.909	-1.015	0.000	-2.320	0.000	-3.010	0.000	PA14_68330	85812.286	-0.048	0.868
PA14_68340	15545.091	-0.609	0.070	-2.095	0.000	-2.506	0.000	PA14_68340	152252.182	-0.080	0.873
PA14_68350	981.061	-1.593	0.000	-3.228	0.000	-2.824	0.000	PA14_68350	10405.857	0.169	0.542
PA14_68360	1347.626	-0.466	0.078	-1.023	0.000	-0.151	0.678	PA14_68360	2015.352	-0.351	0.131
PA14_68370	200.984	0.095	0.837	0.855	0.003	0.781	0.042	PA14_68370	393.241	-0.434	0.088
PA14_68380	360.398	0.323	0.410	0.801	0.008	0.459	0.283	PA14_68380	811.394	-0.278	0.257
PA14_68390	158.958	0.681	0.074	0.678	0.047	0.725	0.106	PA14_68390	361.598	0.297	0.271
PA14_68400	1768.095	-1.515	0.000	-1.554	0.000	-1.185	0.002	PA14_68400	8400.016	-1.427	0.000
PA14_68420	80.551	-0.361	0.457	-1.160	0.002	-0.530	0.330	PA14_68420	210.019	-0.184	0.565
PA14_68430	597.395	-2.629	0.000	-1.474	0.000	3.700	0.000	PA14_68430	101.155	0.913	0.010
PA14_68440	1219.651	-2.334	0.000	-2.247	0.000	3.097	0.000	PA14_68440	170.928	-0.108	0.766
PA14_68450	133.393	-0.318	0.565	0.628	0.130	0.990	0.053	PA14_68450	652.241	-0.604	0.011
PA14_68460	92.070	-0.320	0.545	0.490	0.228	0.718	0.163	PA14_68460	306.875	-0.027	0.997
PA14_68470	2594.450	-0.464	0.279	-1.416	0.000	-2.909	0.000	PA14_68470	6866.806	-1.163	0.000
PA14_68480	235.753	0.965	0.002	1.152	0.000	-0.140	0.779	PA14_68480	722.697	-0.012	0.931
PA14_68490	37.640	0.826	0.134	0.815	0.092	-0.126	0.883	PA14_68490	86.749	0.277	0.522
PA14_68500	366.714	1.364	0.002	1.935	0.000	0.936	0.093	PA14_68500	273.452	-0.192	0.514
PA14_68510	564.332	1.844	0.000	2.367	0.000	0.535	0.155	PA14_68510	467.449	1.069	0.000
PA14_68530	131.063	1.513	0.000	2.036	0.000	0.241	0.706	PA14_68530	219.661	0.280	0.364
PA14_68550	178.338	1.570	0.001	1.771	0.000	0.736	0.225	PA14_68550	211.304	0.479	0.328
PA14_68560	306.852	0.892	0.022	0.730	0.046	-0.117	0.846	PA14_68560	720.102	-0.253	0.392
PA14_68570	55.463	-1.247	0.030	-1.827	0.000	-1.586	0.021	PA14_68570	317.291	-0.686	0.008
PA14_68580	4043.522	0.283	0.355	0.417	0.102	-0.861	0.005	PA14_68580	5244.464	1.289	0.000
PA14_68610	399.886	0.794	0.004	1.433	0.000	0.396	0.285	PA14_68610	629.337	1.863	0.000
PA14_68620	360.795	0.546	0.144	2.199	0.000	-0.670	0.141	PA14_68620	490.329	2.094	0.000
PA14_68630	315.443	0.460	0.405	0.224	0.657	-0.642	0.288	PA14_68630	495.864	-0.469	0.222
PA14_68640	48.827	-0.661	0.248	-0.599	0.226	-0.654	0.332	PA14_68640	174.370	-0.727	0.016
PA14_68660	279.350	-0.455	0.141	-0.861	0.001	-0.999	0.004	PA14_68660	1109.059	-0.366	0.126
PA14_68670	379.662	-0.006	0.989	-0.051	0.888	-0.186	0.684	PA14_68670	793.148	0.380	0.110
PA14_68680	271.630	-0.131	0.770	0.251	0.454	-0.915	0.027	PA14_68680	585.459	0.660	0.006
PA14_68700	913.690	-0.630	0.070	-0.026	0.947	-0.145	0.765	PA14_68700	4268.110	-0.643	0.215
PA14_68710	1173.030	0.699	0.009	1.215	0.000	0.610	0.061	PA14_68710	1261.041	0.804	0.000
PA14_68720	37.881	-0.197	0.792	0.137	0.818	-1.183	0.102	PA14_68720	72.868	0.024	0.967
PA14_68730	2499.736	0.375	0.290	1.135	0.000	-0.453	0.262	PA14_68730	5775.691	0.974	0.001

PA14_68740	367.122	0.280	0.402	0.291	0.302	-0.500	0.176
PA14_68750	63.530	-1.005	0.016	-1.544	0.000	-2.437	0.000
PA14_68770	1135.989	0.670	0.179	1.042	0.016	0.320	0.618
PA14_68780	120.470	-0.877	0.013	-1.154	0.000	-0.748	0.093
PA14_68800	966.108	-1.035	0.000	-1.755	0.000	-2.546	0.000
PA14_68810	1053.531	0.792	0.004	1.224	0.000	1.636	0.000
PA14_68820	2702.119	-0.250	0.517	-0.323	0.311	0.649	0.092
PA14_68830	34.219	1.042	0.092	1.151	0.033	2.710	0.000
PA14_68840	616.544	-2.092	0.000	-2.387	0.000	-0.335	0.486
PA14_68850	322.982	-1.373	0.000	-1.842	0.000	-0.022	0.964
PA14_68860	1479.663	0.127	0.778	-0.184	0.604	-1.398	0.000
PA14_68870	3967.770	0.722	0.050	0.897	0.007	-0.387	0.414
PA14_68890	274.068	-0.539	0.114	0.695	0.015	-0.955	0.017
PA14_68900	993.888	-1.644	0.000	-1.038	0.000	-2.359	0.000
PA14_68920	177.957	-0.043	0.937	0.297	0.405	-0.304	0.546
PA14_68930	669.033	-1.530	0.000	-3.418	0.000	-0.798	0.110
PA14_68940	804.226	-2.378	0.000	-5.444	0.000	-2.827	0.000
PA14_68955	408.232	-0.145	0.684	0.240	0.378	-0.999	0.002
PA14_68970	261.079	0.262	0.478	0.392	0.185	-0.376	0.371
PA14_68980	430.208	0.152	0.704	0.033	0.927	-0.701	0.065
PA14_69000	1358.916	-0.079	0.822	-0.196	0.445	-1.065	0.000
PA14_69010	755.710	0.151	0.655	0.389	0.125	0.263	0.460
PA14_69020	6973.527	1.129	0.000	1.400	0.000	-0.012	0.978
PA14_69030	434.125	-0.087	0.865	-0.521	0.128	-1.104	0.009
PA14_69040	970.304	0.583	0.035	1.576	0.000	1.103	0.000
PA14_69050	409.907	-0.311	0.366	-0.567	0.043	-0.477	0.209
PA14_69060	570.748	-0.771	0.008	-1.429	0.000	-2.954	0.000
PA14_69070	1891.155	-0.794	0.001	-1.206	0.000	-2.911	0.000
PA14_69090	5258.814	-0.565	0.050	-1.560	0.000	-2.622	0.000
PA14_69100	367.315	-0.246	0.491	0.021	0.951	-0.945	0.008
PA14_69110	246.208	-0.408	0.268	-0.545	0.079	0.110	0.823
PA14_69130	293.159	0.576	0.100	3.102	0.000	1.043	0.006
PA14_69140	1503.834	0.658	0.041	0.718	0.015	-0.568	0.151
PA14_69150	1578.538	0.631	0.037	0.956	0.000	-0.024	0.960
PA14_69170	166.614	-0.040	0.938	1.220	0.000	1.141	0.003
PA14_69190	14066.308	0.999	0.000	1.863	0.000	2.756	0.000
PA14_69200	2338.028	-0.481	0.066	0.101	0.714	-0.821	0.005
PA14_69220	1499.386	0.263	0.378	0.270	0.292	-0.433	0.183
PA14_69230	6310.054	-0.117	0.729	-0.631	0.008	-0.025	0.950
PA14_69240	6299.358	-0.305	0.253	-0.965	0.000	0.347	0.257
PA14_69250	332.477	-0.616	0.050	-0.704	0.012	-1.154	0.002
PA14_69260	307.162	-1.209	0.001	-1.532	0.000	-1.357	0.002
PA14_69270	81.392	-0.227	0.685	0.761	0.048	0.958	0.051
PA14_69280	416.329	0.641	0.022	0.836	0.001	0.367	0.314
PA14_69300	295.181	0.463	0.145	0.762	0.005	-0.165	0.709
PA14_69310	56.314	-1.013	0.039	-0.049	0.926	-0.079	0.912
PA14_69320	942.165	0.477	0.088	0.710	0.004	0.229	0.534
PA14_69330	388.580	0.223	0.595	0.498	0.121	0.661	0.105
PA14_69340	854.876	0.490	0.061	1.123	0.000	0.381	0.238
PA14_69350	1466.334	-0.023	0.969	-0.476	0.194	-0.760	0.094
PA14_69370	4660.140	-1.244	0.000	-2.388	0.000	-2.508	0.000
PA14_69380	507.999	0.550	0.065	0.537	0.046	1.015	0.002
PA14_69390	1358.358	-0.880	0.001	-1.086	0.000	-1.673	0.000
PA14_69400	130.120	0.879	0.066	1.982	0.000	0.726	0.211
PA14_69420	769.205	-0.446	0.151	-0.439	0.114	-0.247	0.533
PA14_69430	728.269	-0.548	0.039	-0.424	0.084	-0.080	0.841
PA14_69440	964.457	0.539	0.074	1.029	0.000	0.826	0.015
PA14_69450	702.262	0.344	0.300	1.051	0.000	0.612	0.087
PA14_69470	402.747	-1.575	0.000	-1.714	0.000	-0.299	0.537

PA14_68740	941.288	0.606	0.008
PA14_68750	80.579	-0.219	0.607
PA14_68770	2110.471	0.131	0.742
PA14_68780	563.484	1.374	0.000
PA14_68800	3247.938	0.142	0.617
PA14_68810	1973.474	-0.136	0.608
PA14_68820	5824.273	-0.638	0.002
PA14_68830	82.866	0.263	0.643
PA14_68840	6241.543	-1.771	0.000
PA14_68850	1039.182	-2.225	0.000
PA14_68860	2977.352	-0.478	0.553
PA14_68870	6079.575	-0.456	0.134
PA14_68890	213.664	0.917	0.001
PA14_68900	1732.041	-0.745	0.144
PA14_68920	401.151	-0.246	0.358
PA14_68930	1897.982	-2.384	0.000
PA14_68940	1818.169	-2.123	0.000
PA14_68955	686.620	1.091	0.005
PA14_68970	492.500	0.726	0.003
PA14_68980	765.385	0.602	0.009
PA14_69000	3104.515	0.941	0.000
PA14_69010	2070.853	0.392	0.077
PA14_69020	15391.889	-0.976	0.006
PA14_69030	3534.828	-0.638	0.003
PA14_69040	1909.470	-0.120	0.609
PA14_69050	1497.547	-1.324	0.000
PA14_69060	3068.759	1.197	0.000
PA14_69070	12393.693	1.533	0.000
PA14_69090	16633.697	0.156	0.516
PA14_69100	1305.370	-0.417	0.064
PA14_69110	708.990	-0.846	0.000
PA14_69130	582.383	-0.610	0.009
PA14_69140	1486.656	0.767	0.000
PA14_69150	3287.820	0.938	0.000
PA14_69170	339.995	-0.520	0.046
PA14_69190	13459.049	2.103	0.000
PA14_69200	4889.943	0.159	0.488
PA14_69220	3762.645	0.410	0.062
PA14_69230	12625.773	0.695	0.001
PA14_69240	14784.438	-0.223	0.323
PA14_69250	711.053	1.389	0.000
PA14_69260	1705.142	-0.883	0.000
PA14_69270	189.846	0.258	0.398
PA14_69280	994.017	-0.350	0.146
PA14_69300	375.996	0.365	0.174
PA14_69310	268.287	-0.543	0.047
PA14_69320	1388.170	1.066	0.000
PA14_69330	1111.973	0.156	0.509
PA14_69340	2051.579	0.958	0.000
PA14_69350	5305.738	-1.909	0.000
PA14_69370	21864.593	-0.904	0.000
PA14_69380	1307.132	-0.282	0.235
PA14_69390	3450.955	-0.033	0.918
PA14_69400	370.498	1.754	0.000
PA14_69420	1870.457	0.543	0.013
PA14_69430	2327.636	-0.563	0.009
PA14_69440	2632.813	-0.495	0.064
PA14_69450	2195.432	-0.437	0.049
PA14_69470	3663.775	-1.517	0.000

PA14_69480	1476.475	-0.718	0.039	-0.754	0.018	-0.291	0.529	PA14_69480	8433.760	-1.421	0.000
PA14_69500	2354.209	0.617	0.024	0.990	0.000	0.424	0.216	PA14_69500	2680.441	1.232	0.000
PA14_69510	1772.558	0.127	0.749	-0.363	0.218	-0.037	0.936	PA14_69510	7093.475	-0.334	0.136
PA14_69520	1679.667	-0.194	0.565	-0.854	0.001	-0.769	0.018	PA14_69520	8283.280	-0.779	0.000
PA14_69540	98.190	0.036	0.965	1.039	0.019	0.097	0.896	PA14_69540	199.912	0.462	0.122
PA14_69550	1.782	-0.520	0.752	-0.471	0.727	-0.705	0.603	PA14_69550	125.489	0.305	0.395
PA14_69560	73.123	-1.726	0.000	-2.842	0.000	-2.351	0.000	PA14_69560	1108.579	-1.821	0.000
PA14_69570	1034.699	0.009	0.980	0.097	0.734	-0.590	0.065	PA14_69570	2591.624	0.340	0.139
PA14_69580	413.667	-0.705	0.035	-1.569	0.000	-2.163	0.000	PA14_69580	1916.445	-1.647	0.000
PA14_69590	520.422	-0.056	0.899	-0.652	0.019	-1.130	0.001	PA14_69590	1844.512	0.175	0.434
PA14_69600	1901.742	-0.871	0.002	-2.196	0.000	-0.989	0.003	PA14_69600	8910.293	-2.138	0.000
PA14_69610	824.456	0.201	0.559	0.018	0.955	0.559	0.099	PA14_69610	2092.492	0.115	0.628
PA14_69620	112.235	0.492	0.269	0.435	0.263	0.305	0.587	PA14_69620	263.273	0.515	0.062
PA14_69630	2026.665	0.368	0.392	1.507	0.000	-1.355	0.001	PA14_69630	2500.024	1.095	0.000
PA14_69640	173.720	0.802	0.106	0.970	0.027	0.907	0.112	PA14_69640	1634.185	-2.622	0.000
PA14_69660	1258.200	1.800	0.000	2.255	0.000	0.824	0.023	PA14_69660	2944.892	-0.279	0.728
PA14_69670	2144.634	0.341	0.224	0.655	0.005	-0.136	0.714	PA14_69670	3702.753	1.182	0.000
PA14_69690	820.668	0.619	0.033	0.593	0.026	-0.319	0.406	PA14_69690	1655.471	0.484	0.031
PA14_69700	489.756	0.079	0.870	0.098	0.792	-0.638	0.130	PA14_69700	1241.721	0.113	0.648
PA14_69710	582.604	0.676	0.035	0.702	0.016	0.111	0.817	PA14_69710	622.555	0.993	0.003
PA14_69720	265.973	0.223	0.535	-0.463	0.101	-0.614	0.103	PA14_69720	308.930	0.819	0.002
PA14_69740	18.771	1.141	0.112	1.472	0.016	1.258	0.121	PA14_69740	41.557	0.741	0.148
PA14_69750	480.729	-0.028	0.949	-0.039	0.907	-0.223	0.580	PA14_69750	2827.555	-0.778	0.000
PA14_69760	61.648	1.132	0.012	1.755	0.000	1.692	0.001	PA14_69760	88.820	0.652	0.076
PA14_69770	1046.891	-0.671	0.135	-0.794	0.047	-2.131	0.000	PA14_69770	5341.063	-1.448	0.000
PA14_69780	1008.500	0.214	0.648	0.544	0.125	-0.173	0.748	PA14_69780	3126.158	-0.940	0.009
PA14_69795	246.627	0.274	0.494	1.106	0.000	0.644	0.112	PA14_69795	1209.035	-0.643	0.004
PA14_69810	4680.692	-0.543	0.038	-0.719	0.002	-1.185	0.000	PA14_69810	17993.681	-0.949	0.000
PA14_69820	129.225	-0.282	0.624	0.071	0.890	-0.097	0.889	PA14_69820	346.325	-0.370	0.145
PA14_69840	281.431	-0.798	0.008	-1.024	0.000	-0.012	0.978	PA14_69840	965.425	-1.669	0.000
PA14_69850	1096.732	1.137	0.000	1.407	0.000	1.079	0.002	PA14_69850	1026.515	-0.108	0.672
PA14_69870	854.093	0.115	0.783	-0.174	0.595	-0.443	0.265	PA14_69870	1503.814	-0.297	0.215
PA14_69880	162.897	0.567	0.094	0.746	0.011	-0.480	0.288	PA14_69880	405.217	1.071	0.000
PA14_69890	16.646	0.003	0.999	0.618	0.387	0.334	0.746	PA14_69890	33.535	1.735	0.003
PA14_69900	1029.354	-0.011	0.978	0.346	0.168	-0.059	0.884	PA14_69900	2168.279	0.803	0.000
PA14_69910	1800.881	1.089	0.000	2.433	0.000	2.056	0.000	PA14_69910	1615.601	1.899	0.000
PA14_69925	297.190	0.276	0.512	1.306	0.000	0.135	0.795	PA14_69925	529.785	-1.514	0.000
PA14_69940	1361.295	0.498	0.116	0.768	0.006	0.950	0.006	PA14_69940	2509.067	1.182	0.000
PA14_69950	340.311	-1.357	0.000	-1.197	0.000	-0.043	0.933	PA14_69950	777.989	-1.217	0.000
PA14_69970	1206.757	-0.875	0.046	-0.101	0.835	-0.700	0.187	PA14_69970	1734.340	-0.493	0.207
PA14_69980	2711.570	0.443	0.153	0.860	0.001	-0.117	0.785	PA14_69980	6981.283	0.005	0.966
PA14_69990	1217.460	1.922	0.000	1.276	0.000	-0.337	0.357	PA14_69990	3994.307	1.968	0.000
PA14_70010	197.729	1.050	0.022	-0.136	0.789	-1.228	0.030	PA14_70010	3625.185	1.136	0.000
PA14_70040	4039.148	1.216	0.000	-0.174	0.574	-0.209	0.609	PA14_70040	17674.697	0.991	0.000
PA14_70050	264.092	-0.723	0.037	-1.554	0.000	-1.793	0.000	PA14_70050	1857.971	-0.840	0.000
PA14_70060	899.383	0.197	0.576	-0.497	0.067	-0.501	0.162	PA14_70060	4140.118	-1.024	0.000
PA14_70070	1763.361	-0.013	0.981	-0.241	0.562	-0.082	0.892	PA14_70070	6977.200	-1.023	0.000
PA14_70080	1851.305	-0.018	0.974	0.116	0.757	-0.545	0.204	PA14_70080	3488.745	0.507	0.046
PA14_70100	240.913	1.487	0.000	1.390	0.000	0.350	0.474	PA14_70100	417.924	1.371	0.000
PA14_70110	172.510	0.133	0.767	-0.082	0.827	-0.715	0.095	PA14_70110	391.531	-0.349	0.177
PA14_70120	73.378	0.668	0.134	1.313	0.000	1.035	0.034	PA14_70120	196.023	1.124	0.032
PA14_70140	1809.644	0.848	0.005	-0.751	0.010	-0.048	0.921	PA14_70140	4487.044	0.069	0.764
PA14_70160	485.340	1.151	0.000	-0.511	0.074	1.190	0.000	PA14_70160	1886.055	-0.449	0.042
PA14_70170	128.592	1.127	0.004	-0.083	0.858	0.351	0.532	PA14_70170	318.656	0.232	0.426
PA14_70180	431.163	0.416	0.511	0.785	0.118	-0.429	0.543	PA14_70180	2356.718	1.434	0.000
PA14_70190	3727.888	0.776	0.014	1.336	0.000	0.720	0.059	PA14_70190	5389.700	1.787	0.000
PA14_70200	627.952	-0.322	0.422	-0.672	0.036	-0.985	0.015	PA14_70200	1330.145	0.350	0.147
PA14_70220	29.145	0.194	0.851	0.656	0.357	-0.588	0.559	PA14_70220	99.772	-0.081	0.868
PA14_70230	100.798	-0.332	0.544	0.490	0.248	0.351	0.557	PA14_70230	345.441	-0.308	0.241

PA14_70240	2211.613	0.756	0.007	0.700	0.008	1.045	0.001	PA14_70240	4197.780	1.077	0.000
PA14_70260	889.048	0.694	0.012	0.370	0.175	0.201	0.610	PA14_70260	1858.608	1.302	0.000
PA14_70270	5257.118	-0.081	0.857	-0.033	0.929	0.747	0.048	PA14_70270	8791.213	0.287	0.190
PA14_70280	1724.652	-0.711	0.024	-0.875	0.002	-1.162	0.001	PA14_70280	4775.360	0.373	0.160
PA14_70290	322.062	1.499	0.000	1.649	0.000	0.125	0.800	PA14_70290	405.635	0.174	0.517
PA14_70300	104.373	0.216	0.675	0.875	0.014	1.233	0.005	PA14_70300	136.283	0.289	0.400
PA14_70310	23.332	-0.350	0.681	0.460	0.470	0.068	0.944	PA14_70310	64.130	1.055	0.009
PA14_70330	47.911	-0.299	0.666	1.385	0.002	0.766	0.235	PA14_70330	115.971	1.092	0.001
PA14_70340	10.347	-0.738	0.508	1.769	0.018	0.661	0.551	PA14_70340	11.756	2.082	0.027
PA14_70350	358.253	-0.707	0.088	-0.938	0.011	-0.350	0.513	PA14_70350	1494.735	-1.066	0.000
PA14_70360	855.099	0.285	0.394	0.747	0.004	0.377	0.307	PA14_70360	1767.337	-0.006	0.982
PA14_70370	1243.252	0.044	0.918	0.399	0.143	0.359	0.326	PA14_70370	3752.676	-0.027	0.892
PA14_70390	2724.737	0.656	0.009	1.263	0.000	0.809	0.006	PA14_70390	7388.682	0.424	0.051
PA14_70400	125.758	0.232	0.626	0.517	0.148	-0.026	0.966	PA14_70400	443.606	-0.668	0.083
PA14_70420	1313.068	0.662	0.062	1.124	0.000	0.551	0.200	PA14_70420	2903.043	0.400	0.070
PA14_70430	686.770	0.488	0.149	0.972	0.001	1.352	0.000	PA14_70430	1282.336	0.485	0.034
PA14_70440	1133.786	1.362	0.000	2.560	0.000	1.931	0.000	PA14_70440	1543.022	1.207	0.000
PA14_70450	1261.398	0.282	0.329	0.914	0.000	0.887	0.002	PA14_70450	2203.706	1.052	0.000
PA14_70470	5587.411	0.470	0.085	0.773	0.001	0.853	0.005	PA14_70470	8841.247	1.982	0.000
PA14_70480	1563.478	0.085	0.868	-0.396	0.266	-1.486	0.000	PA14_70480	2339.164	1.854	0.000
PA14_70490	1185.184	1.041	0.000	1.231	0.000	0.588	0.069	PA14_70490	1296.834	2.218	0.000
PA14_70510	20.037	-0.633	0.419	1.176	0.035	-2.131	0.023	PA14_70510	64.286	-1.079	0.009
PA14_70530	57.164	0.455	0.352	0.159	0.729	-1.106	0.068	PA14_70530	101.453	0.821	0.020
PA14_70550	196.761	0.402	0.260	0.683	0.019	-0.686	0.104	PA14_70550	282.186	0.895	0.001
PA14_70560	801.928	0.517	0.092	0.350	0.226	0.032	0.944	PA14_70560	2617.263	-0.516	0.020
PA14_70570	1200.196	0.368	0.173	0.079	0.774	-1.253	0.000	PA14_70570	1892.766	0.946	0.000
PA14_70580	1734.944	-0.165	0.612	-0.649	0.007	-0.936	0.002	PA14_70580	6887.299	-1.479	0.000
PA14_70590	790.208	-0.612	0.035	-0.377	0.172	0.200	0.614	PA14_70590	1476.406	-0.031	0.938
PA14_70600	1225.902	0.095	0.832	-0.611	0.042	-0.264	0.544	PA14_70600	2621.674	-0.192	0.471
PA14_70620	689.733	-0.190	0.548	-0.471	0.053	-1.241	0.000	PA14_70620	1799.320	-0.807	0.000
PA14_70630	991.698	-0.210	0.624	-0.717	0.025	-0.989	0.014	PA14_70630	2418.280	-1.508	0.000
PA14_70640	814.528	-0.206	0.581	-0.781	0.005	-2.782	0.000	PA14_70640	1265.481	-0.365	0.108
PA14_70650	25.283	0.780	0.224	-1.227	0.038	-0.124	0.894	PA14_70650	308.106	-2.079	0.000
PA14_70670	79.855	0.882	0.088	-1.274	0.008	-0.492	0.478	PA14_70670	579.568	-2.270	0.000
PA14_70680	15.626	0.365	0.696	-1.916	0.008	-1.429	0.134	PA14_70680	229.272	-2.405	0.000
PA14_70690	137.860	-0.672	0.084	-2.629	0.000	-1.982	0.000	PA14_70690	1726.499	-3.504	0.000
PA14_70710	550.067	-0.794	0.003	-1.296	0.000	-2.935	0.000	PA14_70710	1465.023	0.062	0.818
PA14_70720	36.504	-0.674	0.273	-0.543	0.305	-0.620	0.396	PA14_70720	90.573	0.960	0.010
PA14_70730	179.098	0.083	0.856	0.515	0.082	0.273	0.534	PA14_70730	482.661	0.387	0.125
PA14_70740	448.167	-2.210	0.000	-3.297	0.000	-0.264	0.518	PA14_70740	2367.473	-3.063	0.000
PA14_70750	335.801	0.001	0.999	-0.100	0.750	0.051	0.910	PA14_70750	987.943	-1.032	0.000
PA14_70760	114.314	-0.552	0.166	-0.789	0.021	-0.585	0.221	PA14_70760	513.557	-1.246	0.006
PA14_70770	1122.095	0.606	0.034	1.128	0.000	0.127	0.758	PA14_70770	2113.782	2.078	0.000
PA14_70780	1799.739	0.254	0.521	0.694	0.023	0.951	0.012	PA14_70780	4032.962	-0.659	0.042
PA14_70790	1303.918	-0.523	0.058	-0.673	0.006	-1.413	0.000	PA14_70790	4073.098	-0.699	0.001
PA14_70800	679.806	0.036	0.931	-0.494	0.058	-1.096	0.001	PA14_70800	4935.879	-2.535	0.000
PA14_70810	442.491	-0.077	0.913	-0.189	0.710	-1.094	0.050	PA14_70810	1890.980	-2.367	0.000
PA14_70830	332.284	-0.196	0.734	0.593	0.155	0.111	0.865	PA14_70830	3523.546	-4.643	0.000
PA14_70850	303.268	-0.319	0.413	0.187	0.587	1.044	0.005	PA14_70850	1533.430	-2.521	0.000
PA14_70860	508.040	-0.693	0.026	-0.140	0.669	0.727	0.044	PA14_70860	7321.147	-4.930	0.000
PA14_70870	0.000	NA	NA	NA	NA	NA	NA	#N/A	#N/A	#N/A	#N/A
PA14_70880	0.000	NA	NA	NA	NA	NA	NA	#N/A	#N/A	#N/A	#N/A
PA14_70890	0.000	NA	NA	NA	NA	NA	NA	#N/A	#N/A	#N/A	#N/A
PA14_70900	0.000	NA	NA	NA	NA	NA	NA	#N/A	#N/A	#N/A	#N/A
PA14_70910	0.000	NA	NA	NA	NA	NA	NA	#N/A	#N/A	#N/A	#N/A
PA14_70920	349.628	0.719	0.028	0.884	0.003	2.424	0.000	PA14_70920	387.534	0.877	0.000
PA14_70930	345.434	-1.321	0.000	-1.374	0.000	-0.911	0.009	PA14_70930	1481.257	-1.254	0.000
PA14_70940	3849.870	1.510	0.000	3.945	0.000	5.915	0.000	PA14_70940	1932.936	2.690	0.000
PA14_70950	10759.312	1.823	0.000	4.463	0.000	6.426	0.000	PA14_70950	2121.522	1.929	0.000

PA14_70970	7446.777	2.432	0.000	5.225	0.000	6.703	0.000	PA14_70970	2032.648	1.918	0.000
PA14_70980	218.520	1.349	0.000	3.852	0.000	4.044	0.000	PA14_70980	146.285	1.579	0.000
PA14_71000	312.521	-0.077	0.884	-0.790	0.019	-0.917	0.036	PA14_71000	899.248	0.257	0.499
PA14_71020	338.436	0.216	0.542	-0.128	0.680	-0.377	0.327	PA14_71020	820.296	0.358	0.162
PA14_71030	860.765	-0.226	0.519	-0.735	0.006	-0.547	0.127	PA14_71030	2544.976	-0.322	0.387
PA14_71060	174.296	0.900	0.034	0.896	0.021	1.373	0.004	PA14_71060	362.784	-1.018	0.000
PA14_71070	237.853	0.087	0.868	-0.943	0.005	-1.297	0.003	PA14_71070	572.861	-0.593	0.013
PA14_71080	228.425	0.514	0.245	0.073	0.872	-2.251	0.000	PA14_71080	788.484	-1.052	0.010
PA14_71090	169.389	1.432	0.000	2.498	0.000	-0.836	0.151	PA14_71090	234.524	1.123	0.000
PA14_71100	234.500	-1.413	0.002	-1.473	0.000	4.679	0.000	PA14_71100	27.147	-2.084	0.001
PA14_71110	2.230	0.047	0.980	1.545	0.196	-0.102	0.946	PA14_71110	3.063	0.274	1.000
PA14_71120	2.288	0.335	0.849	1.179	0.341	0.959	0.482	PA14_71120	4.572	1.650	0.328
PA14_71140	7.306	1.254	0.211	1.028	0.253	-0.058	0.967	PA14_71140	10.823	0.075	0.998
PA14_71150	24.283	-0.041	0.968	0.872	0.135	1.385	0.050	PA14_71150	24.758	0.226	0.772
PA14_71160	18.644	-0.763	0.365	0.382	0.596	0.511	0.581	PA14_71160	30.857	0.185	0.817
PA14_71170	66.199	-0.647	0.141	-0.956	0.011	-2.037	0.000	PA14_71170	114.253	-0.063	0.888
PA14_71180	21.673	-0.584	0.438	0.226	0.727	-0.133	0.889	PA14_71180	50.030	-0.224	0.640
PA14_71190	2.945	-1.104	0.414	-2.320	0.037	-1.352	0.283	PA14_71190	9.633	0.570	0.634
PA14_71200	38.025	0.456	0.475	-0.111	0.854	-0.072	0.933	PA14_71200	47.526	-0.055	0.933
PA14_71210	37.371	0.394	0.566	0.006	0.993	-1.122	0.145	PA14_71210	93.279	0.017	0.944
PA14_71220	93.730	-0.422	0.379	-0.862	0.025	-0.914	0.083	PA14_71220	337.083	-0.484	0.062
PA14_71230	17.259	-0.991	0.208	-1.725	0.011	-1.204	0.179	PA14_71230	35.273	-0.985	0.117
PA14_71240	187.521	1.026	0.001	-0.111	0.761	-0.252	0.600	PA14_71240	165.543	-1.138	0.000
PA14_71250	79.539	1.896	0.000	0.022	0.967	-0.459	0.487	PA14_71250	77.139	0.028	0.950
PA14_71260	302.838	1.302	0.000	-0.510	0.085	-1.665	0.000	PA14_71260	335.912	-0.369	0.179
PA14_71280	87.583	1.151	0.002	-0.648	0.086	-0.898	0.088	PA14_71280	93.865	-0.680	0.069
PA14_71300	13.162	1.085	0.158	-0.934	0.218	-0.777	0.455	PA14_71300	17.226	0.669	0.427
PA14_71310	12.491	1.573	0.038	-0.633	0.445	-0.573	0.613	PA14_71310	30.287	-0.126	0.925
PA14_71320	429.910	0.448	0.364	1.280	0.001	0.329	0.576	PA14_71320	1150.477	-0.345	0.239
PA14_71330	94.213	-0.274	0.646	0.299	0.528	-0.030	0.967	PA14_71330	311.098	0.554	0.037
PA14_71340	47.735	0.565	0.352	1.452	0.002	0.964	0.128	PA14_71340	94.142	1.671	0.000
PA14_71350	8.042	0.069	0.965	1.547	0.072	1.006	0.375	PA14_71350	25.072	1.639	0.009
PA14_71360	93.923	0.823	0.138	2.123	0.000	1.465	0.012	PA14_71360	111.675	1.345	0.000
PA14_71370	19.788	1.309	0.116	3.122	0.000	1.622	0.070	PA14_71370	21.526	0.896	0.199
PA14_71380	111.333	0.728	0.090	0.731	0.056	0.987	0.042	PA14_71380	719.395	-0.339	0.267
PA14_71390	226.100	-0.393	0.343	-0.832	0.013	-1.187	0.006	PA14_71390	1719.824	-0.511	0.017
PA14_71400	13.560	0.146	0.889	-0.696	0.347	-0.595	0.562	PA14_71400	58.899	-1.040	0.046
PA14_71410	217.286	0.659	0.055	-1.172	0.000	-2.206	0.000	PA14_71410	637.232	-0.737	0.025
PA14_71420	200.033	1.406	0.000	-0.460	0.198	-0.831	0.075	PA14_71420	263.344	-0.340	0.220
PA14_71430	256.554	0.244	0.563	0.914	0.003	-0.598	0.182	PA14_71430	811.080	0.537	0.023
PA14_71440	453.570	-0.188	0.651	-0.498	0.111	-1.735	0.000	PA14_71440	775.971	0.591	0.010
PA14_71450	864.003	0.684	0.072	0.354	0.336	-0.164	0.758	PA14_71450	1206.613	0.896	0.000
PA14_71460	70.983	0.773	0.092	-1.489	0.000	-1.835	0.002	PA14_71460	511.197	-1.244	0.001
PA14_71470	105.868	0.941	0.013	-1.287	0.000	-0.831	0.101	PA14_71470	122.545	-1.147	0.000
PA14_71490	64.359	1.158	0.004	-1.178	0.004	-1.739	0.004	PA14_71490	64.514	-0.979	0.018
PA14_71500	347.809	1.632	0.000	-0.460	0.128	-1.480	0.000	PA14_71500	260.543	-0.866	0.002
PA14_71510	28.712	0.718	0.217	-1.257	0.019	-1.787	0.021	PA14_71510	53.446	-0.389	0.422
PA14_71530	97.637	1.147	0.003	-0.902	0.019	-1.645	0.002	PA14_71530	141.452	-1.091	0.001
PA14_71560	274.447	1.654	0.000	-1.280	0.000	-1.074	0.006	PA14_71560	263.988	-0.879	0.001
PA14_71570	446.881	-0.077	0.844	-0.387	0.150	-0.441	0.217	PA14_71570	2197.419	-0.949	0.000
PA14_71580	480.954	-0.276	0.390	0.539	0.032	1.377	0.000	PA14_71580	1010.614	0.415	0.071
PA14_71590	242.166	-1.300	0.003	-0.984	0.019	0.166	0.801	PA14_71590	1553.954	-2.123	0.000
PA14_71600	267.496	-0.087	0.856	0.077	0.837	0.024	0.965	PA14_71600	348.076	1.409	0.000
PA14_71620	552.959	0.534	0.085	0.651	0.018	1.126	0.001	PA14_71620	670.939	1.506	0.000
PA14_71630	5574.287	-0.684	0.008	-1.757	0.000	-2.263	0.000	PA14_71630	23566.372	-0.561	0.008
PA14_71640	212.010	0.144	0.760	1.201	0.000	-0.010	0.985	PA14_71640	621.360	0.574	0.015
PA14_71650	3076.540	-2.757	0.001	-2.281	0.004	-2.213	0.020	PA14_71650	11336.344	1.416	0.003
PA14_71670	208.845	0.437	0.229	0.348	0.284	-0.545	0.211	PA14_71670	296.729	-0.059	0.890
PA14_71680	53.692	0.685	0.177	1.289	0.002	-0.712	0.314	PA14_71680	121.498	1.538	0.000

PA14_71690	58.431	0.082	0.899	-0.264	0.565	-1.236	0.040	PA14_71690	298.181	-0.717	0.006
PA14_71700	32.755	-0.567	0.340	-0.954	0.050	-1.568	0.025	PA14_71700	111.745	0.595	0.088
PA14_71710	138.133	-0.292	0.503	0.280	0.432	-0.691	0.135	PA14_71710	769.744	1.807	0.000
PA14_71720	3608.132	-0.675	0.006	-0.112	0.680	-1.585	0.000	PA14_71720	54560.641	-0.299	0.163
PA14_71740	5243.296	-0.215	0.505	-0.106	0.716	-1.700	0.000	PA14_71740	50321.800	-1.020	0.000
PA14_71750	4344.560	2.793	0.000	5.293	0.000	1.400	0.006	PA14_71750	2791.281	3.516	0.000
PA14_71760	175.911	2.004	0.000	2.329	0.000	1.897	0.000	PA14_71760	461.454	0.049	0.925
PA14_71780	5020.800	1.347	0.000	1.952	0.000	1.774	0.000	PA14_71780	5377.833	0.141	0.586
PA14_71800	569.046	-0.438	0.176	-0.128	0.691	0.112	0.801	PA14_71800	1698.886	-0.921	0.000
PA14_71820	910.656	0.404	0.192	0.827	0.001	-0.057	0.897	PA14_71820	2286.116	2.789	0.000
PA14_71830	66.406	0.585	0.289	1.270	0.004	-0.952	0.169	PA14_71830	235.000	-0.162	0.760
PA14_71840	408.627	0.198	0.600	-0.014	0.969	-0.854	0.021	PA14_71840	1097.678	0.953	0.000
PA14_71850	258.169	0.989	0.000	0.964	0.000	-0.770	0.052	PA14_71850	342.522	1.467	0.000
PA14_71870	1559.695	0.659	0.016	0.895	0.000	0.129	0.748	PA14_71870	2822.709	0.483	0.027
PA14_71880	66.044	-0.090	0.899	1.222	0.003	1.161	0.031	PA14_71880	152.749	-1.391	0.000
PA14_71890	424.435	1.129	0.001	2.006	0.000	0.225	0.675	PA14_71890	725.406	0.738	0.025
PA14_71900	5819.260	0.384	0.490	0.317	0.513	2.301	0.000	PA14_71900	45068.770	-1.522	0.005
PA14_71910	388.001	-0.174	0.695	-1.042	0.001	-1.253	0.002	PA14_71910	1212.419	-0.119	0.676
PA14_71920	405.123	0.114	0.769	-0.806	0.003	-1.634	0.000	PA14_71920	1173.050	0.007	0.990
PA14_71930	409.141	-0.057	0.896	-1.184	0.000	-1.774	0.000	PA14_71930	644.824	1.226	0.000
PA14_71940	418.972	-0.389	0.295	-1.674	0.000	-1.515	0.000	PA14_71940	730.797	1.185	0.000
PA14_71960	578.039	0.182	0.628	-1.122	0.000	-1.499	0.000	PA14_71960	1033.949	2.020	0.000
PA14_71970	917.909	0.214	0.542	-0.734	0.006	-0.684	0.050	PA14_71970	1981.467	1.890	0.000
PA14_71990	1084.557	0.041	0.920	-1.541	0.000	-1.163	0.000	PA14_71990	3807.201	1.837	0.000
PA14_72000	1402.035	0.264	0.469	-0.966	0.001	-0.754	0.039	PA14_72000	4873.680	1.080	0.000
PA14_72010	941.574	0.300	0.390	-0.800	0.004	-1.459	0.000	PA14_72010	2148.822	0.518	0.018
PA14_72020	1085.763	0.657	0.010	-0.581	0.016	-1.753	0.000	PA14_72020	1939.190	1.733	0.000
PA14_72030	158.440	0.495	0.204	-0.233	0.536	-2.081	0.000	PA14_72030	363.624	1.452	0.000
PA14_72040	356.473	0.834	0.019	-0.217	0.566	-1.911	0.000	PA14_72040	438.823	1.605	0.000
PA14_72050	439.264	0.689	0.037	-0.689	0.025	-1.746	0.000	PA14_72050	319.239	0.784	0.003
PA14_72060	224.479	0.043	0.943	-0.426	0.278	-0.684	0.171	PA14_72060	6165.144	-2.869	0.000
PA14_72070	438.810	0.053	0.920	-0.502	0.141	-1.827	0.000	PA14_72070	1280.713	-0.548	0.018
PA14_72080	106.175	0.937	0.041	1.704	0.000	1.469	0.004	PA14_72080	364.708	1.188	0.000
PA14_72090	153.242	0.730	0.062	1.287	0.000	0.723	0.120	PA14_72090	710.259	-0.199	0.685
PA14_72110	599.220	0.599	0.044	2.041	0.000	0.978	0.003	PA14_72110	1050.881	1.296	0.000
PA14_72130	118.713	0.448	0.317	-0.115	0.793	-0.927	0.078	PA14_72130	372.991	-0.205	0.553
PA14_72140	43.141	-0.506	0.405	-1.604	0.001	-1.585	0.018	PA14_72140	490.774	-0.610	0.012
PA14_72150	60.834	0.461	0.420	0.118	0.828	0.678	0.268	PA14_72150	313.281	-0.187	0.486
PA14_72170	801.787	6.114	0.000	4.934	0.000	3.096	0.000	PA14_72170	65.606	0.403	0.367
PA14_72180	630.355	6.019	0.000	4.486	0.000	1.921	0.000	PA14_72180	43.125	0.626	0.227
PA14_72200	23.624	0.976	0.184	2.434	0.000	0.401	0.688	PA14_72200	55.160	1.863	0.000
PA14_72210	125.704	0.993	0.013	2.621	0.000	0.108	0.871	PA14_72210	157.687	1.656	0.000
PA14_72220	215.145	0.011	0.984	0.913	0.007	-0.657	0.173	PA14_72220	493.135	1.143	0.000
PA14_72230	538.664	-0.857	0.002	-0.703	0.007	0.747	0.023	PA14_72230	2258.325	0.203	0.399
PA14_72250	511.361	-0.379	0.282	-0.078	0.824	-0.089	0.852	PA14_72250	1631.154	-0.303	0.194
PA14_72260	3370.415	-1.488	0.000	-2.909	0.000	-3.490	0.000	PA14_72260	20766.432	-0.744	0.000
PA14_72280	243.361	2.471	0.000	2.391	0.000	2.166	0.000	PA14_72280	113.922	0.300	0.414
PA14_72300	376.206	0.507	0.154	0.505	0.112	0.281	0.537	PA14_72300	747.635	-0.542	0.172
PA14_72320	365.555	0.282	0.470	1.444	0.000	1.400	0.000	PA14_72320	968.316	0.486	0.071
PA14_72340	741.193	0.410	0.273	0.041	0.917	1.185	0.002	PA14_72340	2002.054	2.657	0.000
PA14_72350	14.147	0.026	0.981	0.416	0.596	1.634	0.048	PA14_72350	66.424	-2.139	0.000
PA14_72360	4122.158	-1.189	0.000	-3.334	0.000	2.023	0.000	PA14_72360	1236.088	-2.209	0.000
PA14_72370	1946.910	-1.747	0.000	-4.704	0.000	0.990	0.023	PA14_72370	854.699	-2.658	0.000
PA14_72380	600.644	-0.243	0.521	-0.338	0.274	0.065	0.892	PA14_72380	3743.118	0.100	0.635
PA14_72390	849.774	-0.049	0.905	0.286	0.298	0.010	0.982	PA14_72390	1933.844	1.437	0.000
PA14_72400	326.069	0.999	0.003	1.319	0.000	0.917	0.025	PA14_72400	565.498	0.332	0.197
PA14_72410	165.470	1.121	0.003	2.546	0.000	1.697	0.000	PA14_72410	264.050	0.971	0.000
PA14_72420	1007.282	-0.365	0.229	-0.296	0.277	-0.649	0.050	PA14_72420	1864.493	0.512	0.019
PA14_72430	1092.702	0.104	0.805	0.193	0.548	0.432	0.266	PA14_72430	3962.240	-0.020	0.968

PA14_72450	4586.057	-0.132	0.720	-0.284	0.310	-0.359	0.321	PA14_72450	12445.768	-0.236	0.321
PA14_72460	3553.503	-0.553	0.050	-1.189	0.000	-0.306	0.400	PA14_72460	5484.457	0.279	0.413
PA14_72470	1388.860	0.580	0.022	1.137	0.000	1.721	0.000	PA14_72470	3112.914	0.795	0.000
PA14_72480	1803.796	-0.018	0.966	-0.271	0.314	0.420	0.214	PA14_72480	4333.493	-0.641	0.002
PA14_72490	2861.727	0.530	0.064	1.091	0.000	1.045	0.001	PA14_72490	2828.561	1.371	0.000
PA14_72500	2159.864	-1.284	0.000	-2.184	0.000	-2.164	0.000	PA14_72500	13920.674	-1.341	0.000
PA14_72510	1621.467	-0.282	0.442	-0.906	0.001	-1.395	0.000	PA14_72510	8505.040	-0.032	0.913
PA14_72520	435.199	-1.100	0.002	-1.588	0.000	-0.488	0.308	PA14_72520	2126.089	0.283	0.204
PA14_72540	5750.836	-0.935	0.000	-0.701	0.003	0.032	0.937	PA14_72540	13436.879	0.744	0.000
PA14_72550	516.832	0.174	0.668	0.874	0.002	-0.172	0.709	PA14_72550	1560.010	-1.878	0.000
PA14_72560	673.926	0.136	0.751	0.548	0.070	0.774	0.042	PA14_72560	3036.849	-2.214	0.000
PA14_72580	271.550	-0.101	0.859	0.905	0.011	0.226	0.691	PA14_72580	1124.842	-1.932	0.000
PA14_72590	264.004	0.634	0.113	1.433	0.000	0.344	0.511	PA14_72590	612.093	-0.832	0.000
PA14_72600	1067.677	1.186	0.000	1.983	0.000	2.077	0.000	PA14_72600	1515.413	1.376	0.000
PA14_72620	283.211	0.983	0.008	1.413	0.000	0.191	0.732	PA14_72620	463.383	1.904	0.000
PA14_72630	2800.928	0.957	0.039	1.551	0.000	0.327	0.601	PA14_72630	2544.148	2.319	0.000
PA14_72640	2785.628	-0.115	0.754	-0.220	0.432	-1.009	0.001	PA14_72640	3334.163	1.621	0.000
PA14_72650	346.138	4.533	0.000	3.106	0.000	2.936	0.000	PA14_72650	1083.786	5.840	0.000
PA14_72660	765.614	4.803	0.000	2.398	0.000	1.186	0.006	PA14_72660	1118.715	5.664	0.000
PA14_72690	365.521	5.146	0.000	3.360	0.000	1.843	0.000	PA14_72690	861.624	5.611	0.000
PA14_72700	572.945	5.066	0.000	3.511	0.000	2.029	0.000	PA14_72700	936.611	5.061	0.000
PA14_72710	867.921	4.696	0.000	3.652	0.000	1.179	0.013	PA14_72710	1360.120	4.917	0.000
PA14_72720	378.991	0.301	0.363	0.706	0.007	-0.171	0.685	PA14_72720	644.843	0.641	0.007
PA14_72740	282.760	0.527	0.101	1.329	0.000	0.288	0.496	PA14_72740	537.722	1.297	0.000
PA14_72750	165.487	0.006	0.989	0.242	0.505	-0.184	0.727	PA14_72750	506.101	-0.406	0.103
PA14_72760	122.990	-0.192	0.679	0.094	0.810	0.062	0.911	PA14_72760	1783.447	-2.046	0.000
PA14_72770	294.911	0.042	0.934	0.207	0.547	0.299	0.506	PA14_72770	902.720	0.721	0.055
PA14_72780	514.329	0.287	0.536	0.749	0.035	-0.333	0.523	PA14_72780	1099.964	0.778	0.001
PA14_72790	300.956	0.876	0.003	1.347	0.000	0.537	0.164	PA14_72790	716.557	1.757	0.000
PA14_72800	247.994	0.431	0.238	1.133	0.000	-0.149	0.766	PA14_72800	773.317	0.731	0.001
PA14_72810	159.309	0.081	0.878	-0.034	0.935	-0.146	0.789	PA14_72810	605.028	-1.070	0.000
PA14_72820	87.250	-0.418	0.391	-0.110	0.808	1.005	0.031	PA14_72820	483.136	-0.286	0.261
PA14_72830	9.556	-0.066	0.960	-0.102	0.915	-0.694	0.541	PA14_72830	211.858	-0.604	0.032
PA14_72840	365.630	-0.719	0.009	-0.593	0.020	-0.494	0.155	PA14_72840	1534.930	-0.651	0.003
PA14_72850	2538.811	0.920	0.033	1.126	0.004	1.121	0.024	PA14_72850	3602.935	-0.307	0.165
PA14_72870	349.010	0.635	0.049	0.989	0.000	1.300	0.000	PA14_72870	684.216	0.885	0.000
PA14_72880	516.100	1.158	0.004	1.917	0.000	2.485	0.000	PA14_72880	562.879	0.014	0.976
PA14_72890	213.267	0.102	0.828	0.670	0.026	2.030	0.000	PA14_72890	475.427	0.244	0.328
PA14_72900	2422.956	-0.255	0.654	0.545	0.216	0.645	0.248	PA14_72900	11906.805	-0.339	0.489
PA14_72920	696.586	-0.626	0.069	0.024	0.950	0.308	0.489	PA14_72920	2537.534	-0.722	0.001
PA14_72930	1964.478	-1.287	0.000	-0.887	0.000	-0.589	0.076	PA14_72930	10830.039	-0.398	0.175
PA14_72940	331.395	-0.420	0.286	0.491	0.138	-0.453	0.320	PA14_72940	1236.174	-0.580	0.008
PA14_72960	1124.042	-1.120	0.000	4.412	0.000	1.658	0.000	PA14_72960	551.794	-2.751	0.007
PA14_72970	3739.790	0.054	0.880	0.066	0.812	1.693	0.000	PA14_72970	2888.881	-1.339	0.000
PA14_72980	492.445	0.656	0.162	0.767	0.063	0.767	0.152	PA14_72980	598.686	-0.160	0.526
PA14_72990	138.255	0.408	0.337	0.882	0.008	0.432	0.379	PA14_72990	703.591	0.188	0.481
PA14_73000	453.317	0.250	0.720	1.264	0.007	0.876	0.168	PA14_73000	255.000	-1.014	0.000
PA14_73010	605.166	0.205	0.707	0.549	0.164	-0.119	0.857	PA14_73010	736.785	-1.891	0.000
PA14_73020	726.238	-0.107	0.936	1.648	0.043	0.764	0.485	PA14_73020	446.219	-4.222	0.000
PA14_73030	121.793	-0.161	0.750	-0.095	0.819	0.340	0.504	PA14_73030	379.486	-0.929	0.000
PA14_73040	84.401	0.030	0.975	0.594	0.309	0.813	0.280	PA14_73040	183.570	-2.910	0.000
PA14_73050	99.223	-0.588	0.351	0.524	0.278	-0.509	0.523	PA14_73050	122.901	-2.446	0.000
PA14_73060	52.742	0.129	0.892	-0.089	0.906	0.627	0.458	PA14_73060	68.698	-1.226	0.002
PA14_73070	228.942	-0.553	0.410	0.631	0.233	1.254	0.046	PA14_73070	138.123	-1.353	0.013
PA14_73090	308.421	0.639	0.068	-0.631	0.052	-0.441	0.330	PA14_73090	451.258	0.047	0.874
PA14_73100	45.399	0.754	0.156	-0.550	0.283	0.230	0.757	PA14_73100	72.348	-0.301	0.475
PA14_73110	221.605	-0.220	0.565	-0.891	0.002	-0.518	0.193	PA14_73110	404.566	-0.259	0.344
PA14_73120	490.618	-0.018	0.966	-0.359	0.175	-0.025	0.952	PA14_73120	1373.172	-0.535	0.017
PA14_73140	2128.144	-0.447	0.100	-0.441	0.074	-0.559	0.075	PA14_73140	17955.951	-3.433	0.000

PA14_73150	85.535	-0.350	0.535	-0.970	0.025	-1.465	0.012	PA14_73150	470.362	-0.489	0.057
PA14_73160	51.539	-0.473	0.439	0.682	0.144	0.267	0.709	PA14_73160	91.886	1.079	0.002
PA14_73170	558.578	0.094	0.834	0.222	0.500	0.022	0.965	PA14_73170	604.926	1.030	0.000
PA14_73190	194.479	-0.227	0.615	0.657	0.043	1.488	0.000	PA14_73190	300.303	0.300	0.278
PA14_73200	431.625	0.626	0.092	1.384	0.000	0.815	0.054	PA14_73200	654.536	0.391	0.114
PA14_73220	1385.246	-0.134	0.700	-0.568	0.022	-1.078	0.000	PA14_73220	2868.584	0.026	0.933
PA14_73230	1169.400	0.016	0.967	0.473	0.041	-1.640	0.000	PA14_73230	1049.939	-0.022	0.899
PA14_73240	14265.429	-0.494	0.044	-0.593	0.008	-2.118	0.000	PA14_73240	13347.574	0.297	0.176
PA14_73250	10217.462	-0.065	0.884	-0.225	0.472	-1.339	0.000	PA14_73250	8097.143	0.156	0.494
PA14_73260	24521.640	-0.275	0.385	-0.365	0.169	-1.333	0.000	PA14_73260	17493.979	-0.013	0.983
PA14_73280	12330.701	-0.360	0.333	-0.314	0.340	-1.332	0.000	PA14_73280	11290.961	0.383	0.072
PA14_73290	9650.370	-0.085	0.852	-0.003	0.994	-0.896	0.017	PA14_73290	6843.587	0.522	0.014
PA14_73300	9850.297	-0.158	0.655	0.346	0.204	-0.966	0.002	PA14_73300	4683.711	0.671	0.001
PA14_73310	2955.167	0.060	0.913	0.543	0.127	-0.093	0.869	PA14_73310	5195.728	0.910	0.000
PA14_73320	2745.732	-0.078	0.885	1.702	0.000	1.307	0.002	PA14_73320	4877.815	0.587	0.007
PA14_73330	4276.919	0.184	0.622	0.252	0.404	-0.367	0.340	PA14_73330	4742.152	0.387	0.075
PA14_73350	2989.180	0.300	0.336	0.897	0.000	0.435	0.203	PA14_73350	16331.450	-0.606	0.022
PA14_73360	1103.420	0.382	0.307	0.854	0.005	0.507	0.223	PA14_73360	12384.527	-0.789	0.024
PA14_73370	5154.481	0.708	0.005	1.574	0.000	1.705	0.000	PA14_73370	7700.139	1.242	0.000
PA14_73390	75.042	0.813	0.172	2.842	0.000	2.743	0.000	PA14_73390	102.440	-1.294	0.000
PA14_73400	550.167	0.240	0.443	0.981	0.000	0.761	0.013	PA14_73400	1110.348	0.237	0.320
PA14_73410	5741.471	0.392	0.112	1.225	0.000	1.199	0.000	PA14_73410	3163.635	2.208	0.000
PA14_73420	2176.395	0.285	0.363	1.312	0.000	1.495	0.000	PA14_73420	2089.619	2.442	0.000

1 Average normalized read count of PBS-tryptone alone or with human urine, mouse urine or instilled into mouse bladder
2 Average normalized read count of LB with and without 0.5 M urea
